# GIT2 Acts as a Potential Keystone Protein in Functional Hypothalamic Networks Associated with Age-Related Phenotypic Changes in Rats

**DOI:** 10.1371/journal.pone.0036975

**Published:** 2012-05-14

**Authors:** Wayne Chadwick, Bronwen Martin, Megan C. Chapter, Sung-Soo Park, Liyun Wang, Caitlin M. Daimon, Randall Brenneman, Stuart Maudsley

**Affiliations:** 1 Receptor Pharmacology Unit, Laboratory of Neuroscience, National Institute on Aging, National Institutes of Health, Biomedical Research Center, Baltimore, Maryland, United States of America; 2 Metabolism Unit, Laboratory of Clinical Investigation, National Institute on Aging, National Institutes of Health, Biomedical Research Center, Baltimore, Maryland, United States of America; 3 Dodson Interdisciplinary Immunotherapy Institute, Miller School of Medicine, University of Miami, Miami, Florida, United States of America; University of Wuerzburg, Germany

## Abstract

The aging process affects every tissue in the body and represents one of the most complicated and highly integrated inevitable physiological entities. The maintenance of good health during the aging process likely relies upon the coherent regulation of hormonal and neuronal communication between the central nervous system and the periphery. Evidence has demonstrated that the optimal regulation of energy usage in both these systems facilitates healthy aging. However, the proteomic effects of aging in regions of the brain vital for integrating energy balance and neuronal activity are not well understood. The hypothalamus is one of the main structures in the body responsible for sustaining an efficient interaction between energy balance and neurological activity. Therefore, a greater understanding of the effects of aging in the hypothalamus may reveal important aspects of overall organismal aging and may potentially reveal the most crucial protein factors supporting this vital signaling integration. In this study, we examined alterations in protein expression in the hypothalami of young, middle-aged, and old rats. Using novel combinatorial bioinformatics analyses, we were able to gain a better understanding of the proteomic and phenotypic changes that occur during the aging process and have potentially identified the G protein-coupled receptor/cytoskeletal-associated protein GIT2 as a vital integrator and modulator of the normal aging process.

## Introduction

The aging process is associated with an accumulation of molecular perturbations and potential damage to the body's cells, tissues, and organs. These alterations affect multiple processes related to cell survival, genomic instability, altered gene expression patterns, aberrant cellular replication, oxidative damage by reactive oxygen species (ROS), and fluctuations in protein expression and coherent protein post-translational modification [1]. Therefore, with old age and a reduced ability to cope with stress, the body becomes more prone to a variety of pathophysiologies such as neurodegeneration and metabolic syndrome. These accumulated and progressive changes in complex physiological systems such as the endocrine or central nervous system (CNS) are highly likely to be mediated by entire networks of genes and proteins rather than just one single factor. Considerable evidence suggests that both neurodegenerative diseases and pathophysiological aging processes involve a functional interplay between a series of diverse biological systems including neurological, endocrinological, sensory, and metabolic activities [2]–[7]. Many of these systems are functionally integrated together in one crucial organ – the hypothalamus. The hypothalamus is responsible for the regulation of many metabolic pathways by synthesizing and secreting numerous neurohormones that stimulate or inhibit the secretion of trophic hormones from the anterior pituitary. The hypothalamus therefore can control body temperature, thirst, hunger, fatigue, and circadian rhythms [8]. Not only does the hypothalamus act as a master trophic controller of the endocrine system, but it also possesses neuronal projections to many autonomous and higher centers of the brain [9].

As the hypothalamus forms a vital link between multiple complex physiological systems, its role in maintaining the fidelity of ‘neurometabolic’ trans-network communication during the normal or pathological aging process may be of paramount importance for gerontological scientists. In addition, by manipulating certain neuroendocrine hormones to selectively modulate hypothalamic functioning, it may be possible in the future to therapeutically regulate the aging process. For example, regulation of insulin/IGF-1 signaling, a system that strongly regulates hypothalamic function, increased the lifespan of the model *C.* *elegans* organismal system [10], [11]. Similarly, drugs such as L-dopa, which elevate hypothalamic catecholamine activity, have been shown to increase the lifespan of mice by approximately 50% [12].

The emerging appreciation of the coherent connectivity between multiple physiological systems has generated the need to develop a ‘higher-order’ level of understanding of the integration of these systems, *i.e.,* so-called ‘systems biology’. This concept of network or ‘systems’ biology, *i.e*. biological functions are mediated by strongly or weakly connected groups of genes/proteins rather than simple linear signaling pathways, can often seem too diffuse and non-specific to yield actionable data for biomedical or pharmaceutical science. Recent mathematical modeling of ‘real-world’ networks have demonstrated that, in most cases, complex network systems are not connected in a equitable and homogenous manner, but rather typically consist of at least two levels, *i.e.,* small, tightly-connected ‘sub-networks’ which are then collected together into larger constellations of groups of multiple ‘sub-networks’ [13]. From a biological standpoint, it is easy to analogize the small ‘sub-networks’ to biological programs such as kinase signaling cascades (*e.g.* mitogen-activated protein kinase cascades) or receptor signaling systems (*e.g*. insulin receptor signaling system) in different tissues, while endocrine or neuronal axes could represent the constellations of these groups of smaller ‘sub-networks’.

While the physiological output of any given gene/protein network may be an eventual function of the activity of all of the constituent components, the relative contribution (to the eventual functional output) of each gene/protein in this network is not equal [14], [15]. Hence within networks of functionally-related genes/proteins, there are likely to exist specific hubs that consist of genes/proteins that form the most important bridges, or ‘loose-connections’, between the smaller functional programs (‘sub-networks’) contained within the global network system. Such genes/proteins within a functional network are often described as keystones. These keystones profoundly enhance and facilitate rapid and facile connection between disparate parts of a network constellation and, as such, can be considered as functional ‘short-cuts’ in the complex system [13]. It has been demonstrated, using mathematical modeling of graph and network theories, that even in networks containing thousands to millions of nodes, surprisingly few (5–10) ‘short-cuts’ (keystones) are required to facilitate rapid transfer across even the largest of systems [13].

In this study, we aim to identify specific alterations in functional hypothalamic protein networks and the potential presentation of keystone network factors that occur over time in the hypothalamus. In order to achieve this goal, we examined differences in protein levels expressed in the hypothalami of young, middle-aged, and old rats using a variety of synergistic combinatorial proteomic and bioinformatic techniques. Following these unbiased mathematical approaches, we show that specific protein networks that are altered in the hypothalamus during the aging process may be primarily linked to and regulated by a small number of crucial ‘network-crossing’ keystone factors. Investigating the nature of these multidimensionally-active factors in the context of aging may allow us to greatly enhance our understanding of the normal or pathological aging process.

## Results

### Longitudinal hypothalamic protein expression patterns

**Figure 1:**
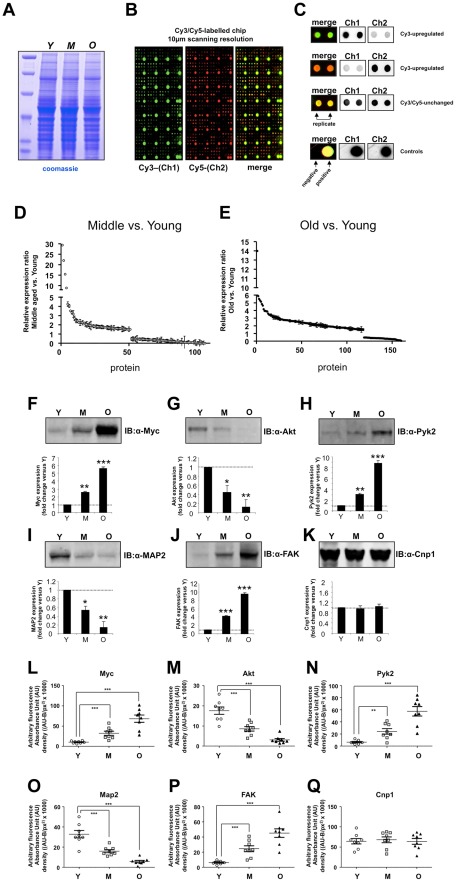
Age-related proteomic alterations in the hypothalamus. (A) Coomassie staining of pooled input hypothalami for Cy-dye labeling and hybridization with Cell Signaling antibody array: Y–young animal pool; M–middle-aged animal pool; O–old animal pool. (B) Prototypic single channel and merge images from scanned Panorama® Cell Signaling Antibody array chips. Specific antibody species are printed in duplicate across the chip. (C) Prototypic examples of protein results for factors up-regulated, down-regulated, or unchanged in either Cy3 or Cy5 channels are indicated. Positive and negative hybridization controls from the chips are also indicated. (D) Protein expression ratios (<0.5 or >1.5 ratio: middle versus young) for proteins in middle aged versus young hypothalami. Datapoints plotted represent the mean ± standard error of mean (SEM) from three separate experimental hybridizations which included Cys-Cy5 dye swaps for the samples. (E) Protein expression ratios (<0.5 or >1.5 ratio: old versus young) for proteins in old aged versus young hypothalami. Datapoints plotted represent the mean ± SEM from three separate experimental hybridizations which included Cys-Cy5 dye swaps for the samples. (F–K) Western blot validation of specifically identified proteins, from Panorama® array analysis, and their age-dependent expression trends (Y-young pool, M-middle aged pool, O-old pool). Proteins validated from pooled animal input were Myc (F), Akt-1 (G), Pyk2 (H), Map2 (I), FAK (J), and Cnp-1 (K). Data presented represents mean ± SEM from three separate blots. Statistical significance was assessed using a Student's t-test with GraphPad Prism: * = p<0.05; ** = p<0.01; *** = p<0.001. (L–Q) Expression patterns for specific proteins were also validated for each animal used as input for the Y (white circle), M (grey square), or O (black triangle) hypothalamic pools. Proteins validated from individual animal inputs were Myc (L), Akt-1 (M), Pyk2 (N), Map2 (O), FAK (P) and Cnp-1 (Q). Data on the histograms are represented as mean ± SEM from the multiple animals.

Cytoplasmic hypothalamic extracts were prepared from young (Y, 2–3 months), middle-aged (M, 10–12 months), and old (O, 24–26 months) rats (n = 8 each). Individual lysate samples were taken from each animal and then pooled together for each age group. One dimensional gel separation was performed to control for any gross proteome differences between the three tissue pools (Fig. 1A). Gel separation indicated that after coomassie staining the pool inputs did not grossly differ in their global protein content. These samples were then prepared for Panorama® Cell Signaling antibody array hybridization by labeling with Cy-3 or Cy-5 fluorescent dyes (Fig. 1B, C). Relative protein expression between middle-aged (M) or old (O) versus young (Y) animals was assessed (in triplicate) using standardized dye-swapping controls as described previously [2]. Compared to young animals, there were 50 significantly differentially expressed proteins in middle aged animals, demonstrating an M/Y expression ratio of >1.5 (p<0.05) and 55 proteins with an M/Y ratio of <0.5 (p<0.05) (Fig. 1D: Table S1). Considerably more proteins demonstrated a significant change in expression between the old and the young animal comparison: 118 proteins demonstrated an O/Y expression ratio of >1.5 (p<0.05) and 30 proteins possessed an O/Y expression ratio of <0.5 (p<0.05) (Fig. 1E: Table S2). We chose six proteins identified in all the antibody array samples (demonstrating up, down, or no change in expression regulation: Myc, Akt, Pyk2, Map2, FAK, Cnp1) to validate the initial experiments using standard western blot procedures (Fig. 1F–K). Using the pooled hypothalamic samples (Y, M, O), we validated the expression trends for each of these proteins (Myc, Pyk2, FAK: up-regulated with advanced age; Akt, Map2: down-regulated with advanced age; Cnp1: unchanged with advanced age) seen with the Panorama^®^ Cell Signaling array analysis (Tables S1, S2). In addition to standard western analysis of the input pooled hypothalamic samples, we also performed validatory western analysis on the individual animal samples (Fig. 1L–Q). Similarly to the pooled samples, we observed the following significant protein expression trends: Myc, Pyk2, FAK: up-regulated with advanced age; Akt, Map2: down-regulated with advanced age; Cnp: unchanged across age-span (Fig. 1L–Q).

**Figure 2:**
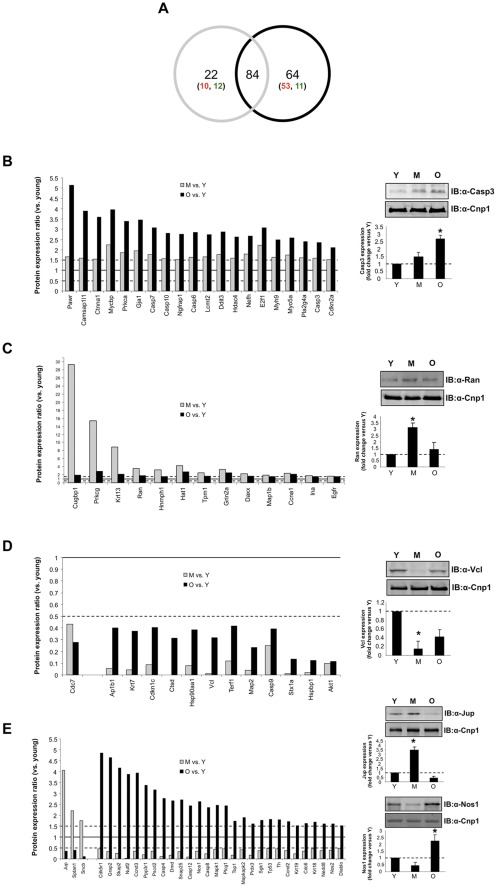
Age-dependent protein expression changes in the rat hypothalamus. (A) Venn diagram analysis of significantly-regulated (p<0.05) proteins in the middle versus young (grey circle) and the old versus young (black circle) antibody array analyses. Proteins uniquely regulated in either middle age (22) or old age (64) animals are subsequently broken down into up-regulated (red numbers) or down-regulated (green numbers) groups. The 84 commonly-regulated proteins were further dissected into different regulatory-behavior groups represented in panels B–E. For each regulation-behavior group an exemplary protein is verified from the animal hypothalamic pools (Y–young, M–middle aged, O–old). (B) Proteins up-regulated in both M (grey bars) and O (black bars) hypothalami compared to Y animals, with O ratio >M ratio. The associated validation was performed using western blot for caspase 3 (Casp3). (C) Proteins up-regulated in both M (grey bars) and O (black bars) hypothalami compared to Y animals, with M ratio >O ratio. The associated validation was performed using western blot for Ran. (D) Proteins down-regulated in both M (grey bars) and O (black bars) hypothalami compared to Y animals, with M ratio >O ratio and O ratio >M ratio. The associated validation was performed using western blot for vinculin (Vcl). (E) Proteins differentially regulated between M or O timepoints relative to Y (up-regulated at M and down-regulated at O (verified using junction plakoglobin-Jup), or down-regulated at M and up-regulated at O (verified using nitric oxide synthase 1-Nos)). For each verification, data on each histogram is represented as mean ± SEM from the multiple animal pools. Statistical significance is as follows: * = p<0.05, ** = p<0.01, *** = p<0.001.

With respect to the proteins differentially regulated between middle-aged or old animals and the young cohort, 84 proteins were found to be common between both M and O samples (Fig. 2A). In the middle-aged hypothalami there were 22 uniquely-regulated (not in O/Y) proteins (10 up-regulated, 12 down-regulated), while in the old animals there were 64 uniquely-regulated proteins (53 up-regulated, 11 down-regulated) (Fig. 2A). Among the 84 proteins commonly regulated in M/Y and O/Y, 53 proteins were similarly regulated (compared to Y animals), with 38 proteins up-regulated in M and O (Fig. 2B, C) and 15 proteins down-regulated in M and O (Figure 2D). We validated additional proteins for each of these protein subgroups: caspase 3 (Casp3, Fig. 2B), Ran (RAN, member RAS oncogene family, Fig. 2C) and vinculin (Vcl, Fig. 2D). In each of these western validations we recapitulated the expression trends observed with the antibody array (Table S3).

Of the proteins identified in both M and O samples, when compared to Y, there were 31 proteins differentially regulated between M and O samples: three proteins were up-regulated in M versus Y, yet down-regulated in O versus Y (Fig. 2E), and 28 proteins were down-regulated in M versus Y but up-regulated in O versus Y (Fig. 2E). Again, we validated individual proteins for each of these subgroups: junction plakoglobin (Jup, Fig. 2E) and nitric oxide synthase-1 (Nos-1, Fig. 2E). In each of these western validations we recapitulated the expression trends observed with the antibody array.

### Functional clustering of age-regulated hypothalamic proteins

We performed two independent types of functional protein clustering, KEGG signaling pathway analysis and GO term group clustering, to assess the potential physiological focus of the hypothalamic proteins altered with advancing age. Complete significantly-regulated protein lists (Tables S1, S2), containing proteins up- or down-regulated compared to Y animals, were used for both forms of bioinformatic analysis. KEGG pathway or GO term group clustering criteria were similar for both types of analysis, *i.e.* >2 proteins per KEGG pathway/GO term group at a p<0.05 value (hypergeometric test of significance).

**Figure 3:**
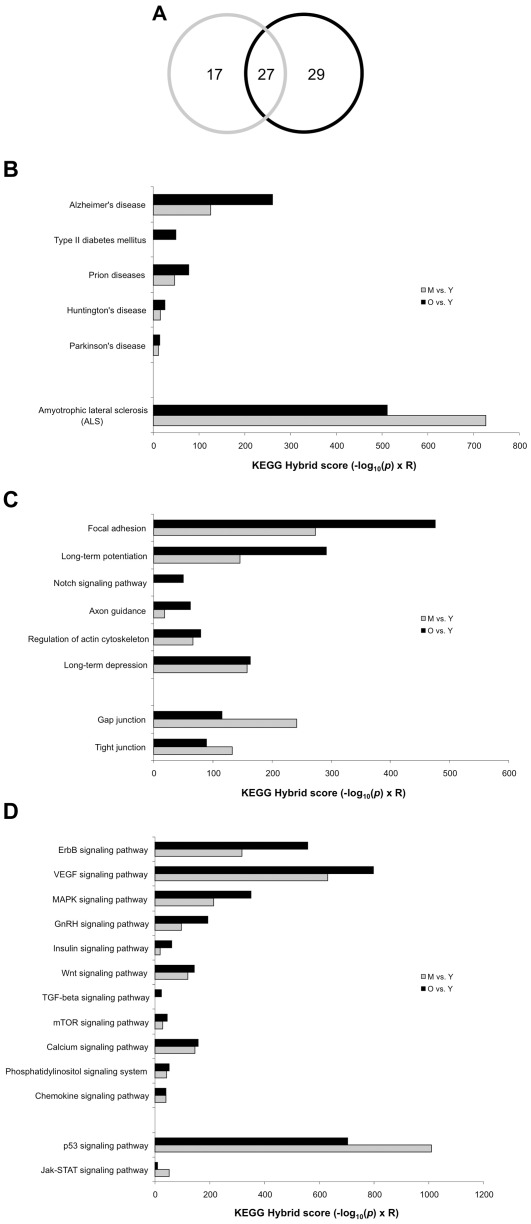
KEGG signaling pathway analysis of aging-related hypothalamic proteins. Proteins significantly regulated in middle (M) or old (O) aged animals compared to young (Y) animals were used as input data for KEGG signaling pathway population analysis. (A) Venn diagram analysis of middle-aged (grey line) and old-aged (black line) significantly-regulated KEGG pathways demonstrated 27 common KEGG terms between old and middle-aged tissues. The common (27) significantly populated pathways for middle-aged (grey bars) and old aged (black bars) animals were then rationally clustered into subgroups focused upon disease pathology (B), neurophysiological activity (C), and intermediary cellular signaling activity (D). For each significantly-populated KEGG pathway a ‘hybrid’ score was generated which represents the −log_10_ of the enrichment probability multiplied with the relative enrichment factor compared to the background proteomic expression.

Clustering of the significantly-regulated hypothalamic proteins from M or O animals resulted in the population of 44 and 56 distinct KEGG pathways, respectively (Tables S4, S5). There were 27 significantly-populated KEGG pathways that were common between the two different age profiles (M/Y and O/Y) (Fig. 3A). These commonly-populated KEGG pathways were then rationally grouped together into sets focusing upon disease pathways (Fig. 3B), neurophysiological architecture (Fig. 3C), and intermediary cell metabolism signaling pathways (Fig. 3D). Among these commonly-regulated KEGG pathways, 22 out of 27 possessed a greater hybrid score (indicating the profundity of KEGG pathway population) within the old animal datasets, demonstrating a strong age-dependent trajectory of these predicted biological functions.

GO term group clustering of the significantly-regulated hypothalamic proteins from M or O animals resulted in the population of 112 and 114 distinct GO term group terms, respectively (Tables S6, S7). There were 79 significantly-populated GO terms common between the two different age profiles (M/Y and O/Y) (Fig. S1A). Similar to the KEGG pathways, these GO term groups were grouped together into rational functional sets: cell structure/function (Fig. S1B), cell cycle control (Fig. S1C), enzyme activity (Fig. S1D), and neurophysiological architecture (Fig. S1E). Among these commonly-regulated GO term groups, 53 out of 79 possessed a greater hybrid score (indicating the profundity of GO term group population) within the old animal datasets, again demonstrating a strong age-dependent trajectory of these functions.

The KEGG functional classifications created from the primary hypothalamic protein data demonstrated the generation of multiple neurodegenerative (‘*Alzheimer's disease’*, ‘*Huntington's disease’*, ‘*Parkinson's disease’*), neurological (‘*Prion diseases’*, ‘*Amyotrophic lateral sclerosis’*) and metabolic (‘*Type II diabetes mellitus’*) disorder-related groups with advancing age (Fig. 3B). A profound age-related cytoskeletal/focal adhesion remodeling functional phenotype (‘*Focal adhesion’*, ‘*Axon guidance’*, ‘*Regulation of actin cytoskeleton’*) was also evident within the populated KEGG pathways (Fig. 3C). Such functional predictions corroborate our previous western blot and antibody array data, demonstrating a strong age-related upregulation of proteins (Pyk2, FAK) involved in organizing cytoskeletal dynamics in neuronal tissue (Fig. 1). Among the age-related KEGG signaling pathway paradigms (Fig. 3D), several convergent functional themes emerged: neurodevelopmental signaling (‘*TGF-beta signaling’*, ‘*Wnt signaling’*), excitatory calcium cell signaling (‘*Phosphatidylinositol signaling’*, ‘*Calcium signaling’*) and metabolism-based signaling (‘*VEGF signaling’*, ‘*Insulin signaling’*, ‘*Jak-STAT signaling’*) (Fig. 3D). Similar to the KEGG functional analysis, the significantly-populated GO term group clusters also revealed strong neuronal remodeling/focal adhesion/cytoskeletal aspects (Fig. S1B, S1E), cell cycle regulation (Fig. S1C), and calcium-targeted cell signaling enzyme activity (Fig. S1D) focused functionalities.

Statistical clustering of proteins into different forms of canonically-curated functional KEGG pathways or GO term groups allows for a ‘higher-order’ appreciation of their functional relationships to each other. However, many GO term groups, and especially KEGG pathways, possess considerable vagueness, content overlap, and experimental redundancy due to their historically-curated nature [16]. While these functional annotation tools may possess some drawbacks when their isolated use is relied upon, it is likely that they can still be of great importance when they are employed in a combinatorial manner with a novel form of data-mining that has demonstrated great promise for discovering previously unknown biomedical interactions, *e.g.* latent semantic indexing [2], [3], [17]–[21]. Using latent semantic indexing (LSI) of biomedical text databases, the mathematical correlations of input text terms with genes/proteins can be assessed, even if they are not present in classically curated datasets such as GO or KEGG. This allows for the discovery of novel, previously unidentified connections between significantly altered genes/proteins.

### Identification of multidimensional ‘keystone’ factors in age-related protein networks

**Figure 4:**
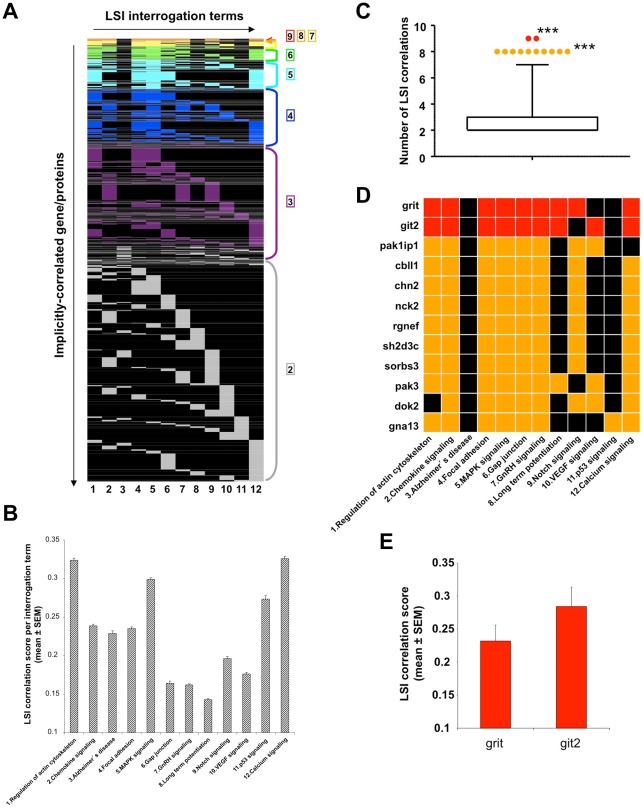
Latent semantic indexing correlations of KEGG signaling pathways terms with proteins. (A) Latent semantic indexing (LSI) interrogation matrix between input significantly-regulated KEGG signaling pathway terms. Colored blocks represent the individual LSI implicit correlation of the specific protein (vertically organized on left of heatmap: 1–2524 – see Table S20) with the respective KEGG term (1-Regulation of actin cytoskeleton, 2-Chemokine signaling, 3-Alzheimer's disease, 4-Focal adhesion, 5-MAPK signaling, 6-Gap junction, 7-GnRH signaling, 8-Long term potentiation, 9-Notch signaling, 10-VEGF signaling, 11-p53 signaling, 12-Calcium signaling). The number of KEGG signaling pathway correlations for each protein is indicated by the color of the respective heatmap blocks (9 correlations-red; 8 correlations-orange; 7 correlations-yellow; 6 correlations-green; 5 correlations-light blue; 4 correlations-dark blue; 3 correlations-purple; 2 correlations-grey). (B) Mean ± SEM for the total implicitly-correlating proteins for each of the 12 input KEGG signaling pathways. (C) Box and whisker plot with 1–99% statistical cut-offs (GraphPad Prism) of the number of specific correlations to KEGG pathways each protein possessed. Twelve proteins demonstrated a statistically-significantly greater number of KEGG pathway correlations compared to the total protein mean number of correlations (*** = p<0.001). (D) Expanded heatmap identification of specific proteins possessing a significantly greater number of KEGG pathway correlations compared to the mean number of KEGG pathway correlations for all implicit proteins. (E) Mean ± SEM of LSI correlation scores (across all 9 correlations) for Grit and GIT2.

To identify novel protein factors that may act as keystones by connecting the potentially complex series of functional networks involved in aging, we performed combinatorial LSI using multiple KEGG and GO term groups significantly populated by age-regulated hypothalamic proteins. We chose twelve KEGG signaling pathway text terms, including all functional subsets described in Fig. 3B–D, to use as input interrogation terms for a complete murine biomedical protein database (Computable Genomix, https://computablegenomix.com/geneindexer). This process yields lists of proteins that possess a quantitative LSI correlation score (cut off of >0.1 indicates at least an ‘implicit’ correlation) associated with the input text term. To maintain equality between the output lists of LSI-correlating proteins from the diverse input KEGG terms used (1-Regulation of actin cytoskeleton, 2-Chemokine signaling, 3-Alzheimer's disease, 4-Focal adhesion, 5-MAPK signaling, 6-Gap junction, 7-GnRH signaling, 8-Long term potentiation, 9-Notch signaling, 10-VEGF signaling, 11-p53 signaling, 12-Calcium signaling), we chose the top 1000 highest-scoring proteins in each case (terms 1–12). The individual protein LSI correlation scores for the input KEGG pathway terms (1–12) are listed in Tables S8, S9, S10, S11, S12, S13, S14, S15, S16, S17, S18, S19. To identify potentially multidimensional keystone factors in age-related protein patterns, we combined the LSI correlation results from the 12 input KEGG terms into a heatmap diagram. Only proteins that displayed an LSI correlation (>0.1 score) in at least two separate KEGG term outputs were used for heatmap analysis (Fig. 4A). Using a >2 KEGG pathway correlation cut-off, a matrix of 2524 proteins was generated (Table S20). The highest number of correlations (8 KEGG terms) was achieved by 2 proteins: Grit (Rho GTPase activating protein 32/Arhgap32) and GIT2 (G protein-coupled receptor kinase interacting protein 2) (Fig. 4A: Table S20). The mean LSI correlation scores for all correlated proteins, generated by the twelve input terms, were all significantly greater than 0.1, demonstrating a greater than implicit correlation for all proteins (Fig. 4B). After taking together the total number of proteins demonstrating multiple (>2) KEGG term correlations and performing a group statistical analysis, twelve were found to exist outside a 99% percentile of the mean results assuming a normal distribution (10 with 7 correlations, 2 with 8 correlations) (Fig. 4C: Box & Whiskers plot, p<0.001). These twelve proteins and their specific KEGG term correlations are highlighted in Fig. 4D. GIT2 possessed a greater mean LSI correlation score (across the 8 KEGG pathways linked to it) than Grit, despite a similar number of cross-KEGG pathway correlations (Fig. 4E). These unbiased results may suggest that cytoskeletal-organizing factors could play an important role in maintaining normal neuronal function with age in the hypothalamus.

In addition to using KEGG pathway analysis for unbiased keystone identification, we also employed GO term enrichment analysis combined with LSI to investigate the presence of multidimensional factors in hypothalamic aging. As with the KEGG pathway term-directed LSI analysis, we chose twelve significantly-populated GO terms from the four major functional GO term groups (Fig. S1) and generated a correlation heatmap for GO terms (Fig. S2A). As with the KEGG multidimensional correlation analysis, we chose the top 1000 highest-scoring and implicitly correlating proteins for each input GO term: 1-Actin filament binding, 2-Anatomical structural development, 3-Cyclin-dependent protein kinase inhibitor activity, 4-Regulation of cell proliferation, 5-Cytoskeletal protein binding, 6-Macromolecular complex, 7-Nitric oxide synthase activity, 8-Synapse, 9-Response to stress, 10-Intracellular membrane-bound organelle, 11-Regulation of programmed cell death, 12-Protein kinase activity (Fig. S2B). The LSI protein output for each of these GO term interrogation units are listed in Tables S21, S22, S23, S24, S25, S26, S27, S28, S29, S30, S31, S32. As with the LSI analysis of the input significant KEGG pathways, we employed a>2 cut-off for the number of multiply-correlating proteins identified using GO terminology input (Fig. S2C). Using this criterion, we created a matrix containing 2902 proteins (Table S33). The highest number of correlations presented by proteins was 7 and this was achieved by 9 proteins: Ccdc88a–coiled-coil domain containing 88A, Kank1–KN motif and ankyrin repeat domains 1, Pcnp–PEST proteolytic signal containing nuclear protein, Plekho1–pleckstrin homology domain containing, family O member 1, Rsu1–Ras suppressor protein 1, Tfpt–TCF3 (E2A) fusion partner, GIT2–G protein-coupled receptor interacting transcript 2, Plrg1–pleiotropic regulator 1, Zdhhc16–zinc finger, DHHC domain containing 16 (Fig. S2A: Table S33). The mean LSI correlation scores for all correlated proteins, generated by the twelve input terms, were all significantly greater than 0.1, demonstrating a greater than implicit correlation (Fig. S2B). After taking together the total number of proteins demonstrating multiple (>2) GO term correlations and performing a group statistical analysis, nine were found (all with 7 correlations) to exist outside a 99% percentile of the mean results assuming a normal distribution (Fig. S2C: Box & Whiskers plot, p<0.001). These 9 proteins and their specific GO term correlations are highlighted in Fig. S2D. Among these highlighted multidimensional proteins, the mean LSI correlation scores were relatively similar to each other (Fig. S2E). Interestingly, the only protein that demonstrated a similar presence in the most significantly correlating proteins from KEGG and GO term LSI input was GIT2. We therefore chose to validate the age-dependent changes predicted for GIT2, the highest scoring and highest LSI-correlating protein, in the hypothalamus.

### Age-dependent alterations in hypothalamic GIT2 expression

**Figure 5:**
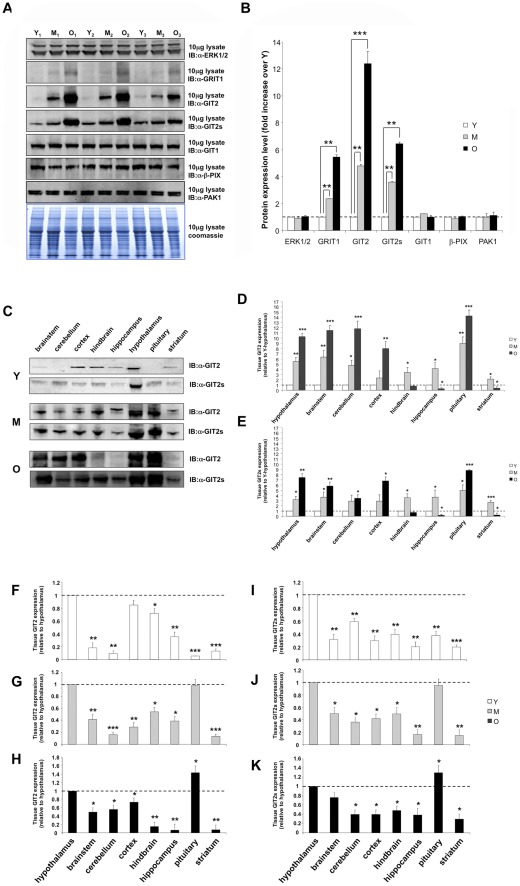
Age-dependent expression profile of GIT2 in central nervous system tissues. (A) Expression profiles across three randomly chosen hypothalamic samples (Young, Y_1_-Y_2_-Y_3_; Middle, M_1_-M_2_-M_3_; Old, O_1_-O_2_-O_3_) for ERK1/2, GRIT, GIT2, GIT2short (GIT2s), GIT1, β-PIX, and PAK1. The loading protein input control with coomassie staining of the gel is also indicated below. (B) Quantification (mean ± SEM) of age-dependent alterations in protein expression for middle aged (grey bars) or old (black bars) animals compared to the young controls (white bars). (C) Brain region-specific alterations of GIT2 and GIT2s in young (Y), middle (M), and old (O) age animals. Quantification of multi-brain region GIT2 (D) and GIT2s (E) expression across the experimental age-span (n = 10). Multiple brain region expression levels relative to hypothalamus of GIT2 in young (F), middle-aged (G), and old animals (H). Multiple brain region expression levels, relative to hypothalamus of GIT2s in young (I), middle-aged (J), and old animals (K). Statistical significance is as follows: * = p<0.05, ** = p<0.01, *** = p<0.001.

From our combinatorial bioinformatic investigation of predicted age-dependent protein expression in the hypothalamus, we decided to assess the age-dependent expression of GIT2 using standardized techniques. We assessed protein expression with specific western blots in three randomly-chosen young (Y_1_, Y_2_, Y_3_), middle-aged (M_1_, M_2_, M_3_), and old rat hypothalami (O_1_, O_2_, O_3_: Fig. 5A). We found that with similar levels of loaded protein (10 µg), the ERK1/2 expression profile was unchanged with age but the expression profile for Grit and GIT2 was strongly age-dependent (Fig. 5A–B). The age-dependent elevation of GIT2 was extremely profound while that of Grit was less strong. GIT2 often demonstrates co-expression with a shorter isoform, termed GIT2-short (GIT2s). Similar to the age-dependent increase in GIT2 hypothalamic expression, we also found a strong elevation of GIT2s with age (Fig. 5A). The GIT family of proteins consist of GIT1 and GIT2, as well as the smaller isoforms of GIT2 including GIT2s [22]. Proteins belonging to the GIT family were first identified as ADP-ribosylation factor GTPase-activating proteins. GIT proteins, and GIT1 especially, associate with signaling factors, p21-activated kinase (PAK) and PAK-interacting exchange factor (PIX), and the monomeric G proteins that control cytoskeletal remodeling, *e.g.* Rac and Cdc42, which regulate cell structure and movement [22]. However, in contrast to GIT2 and GIT2s, the levels of GIT1, β-PIX, or PAK1 did not demonstrate a significant elevation with age in the hypothalamus (Fig. 5A–B), suggesting a strong age-dependent hypothalamic functionality for GIT2. As we have seen that protein expression of GIT2 is altered during the aging process, we also investigated whether alterations in post-translational modification of GIT2 are also sensitive to aging. GIT2 functional activity has been demonstrated to be associated with its tyrosine phosphorylation [23]. We assessed the tyrosine phosphorylation status of GIT2 in normalized (for GIT2) hypothalamic lysates collected from rats of the different ages. We found a trend for age-dependent elevation of the phosphotyrosine content in hypothalamic GIT2 (Fig. S3). This increase in phosphotyrosine content may indeed be associated with the age-related elevation in kinases linked to GIT2 phosphorylation such as FAK (Fig. 1). We next assessed whether there were age-dependent changes in GIT2 and GIT2s expression across other regions of the central nervous system outside our primary hypothalamic locus.

### Age-dependent alterations in global brain GIT2 expression

Using identical levels of input protein (10 µg) we assessed the age-dependent expression profile of GIT2 and GIT2s across multiple brain regions in Y, M and O rats (Fig. 5C; relatively quantified in Fig. 5D–GIT2, 5E–GIT2s). Using identical enzyme-linked chemifluorescence exposure and phosophorimager data collection parameters, we directly compared the age-dependent expression profile of GIT2 and GIT2s in the brainstem, cerebellum, cortex, hindbrain, hippocampus, hypothalamus, pituitary, and striatum. Generally, the relative extent of GIT2 or GIT2s expression increased with the advancing age of the rats. In young animals, expression of GIT2 was almost undetectable in many brain regions, *e.g*. the brainstem, cerebellum, and pituitary, while in areas such as the cortex, hindbrain and especially the hypothalamus, high expression of GIT2 was detected (Fig. 5C). GIT2s demonstrated a generally similar expression profile to GIT2 in the young animals across brain regions. Generally, there was a progressive increase in GIT2 or GIT2s expression in the middle and old-aged animals compared to the young animal brain. Interestingly, in addition to the age-dependent alterations of the long and short isoforms of GIT2 we also noted a prominent ‘intermediate’ GIT2-sera immunoreactive band of approximately 60–62 kDa that also demonstrates an age-dependent expression alteration profile (Fig. S4). It is well known that multiple GIT2 splice forms exist, however the precise molecular structure of this intermediate form remains an interesting topic for future studies into its molecular activity. With respect specifically to the long GIT2 isoform, progressive and significant increases in expression (O>>M>>Y) were observed in the following regions: hypothalamus; brainstem; cerebellum; cortex; pituitary (Fig. 5D). In the hindbrain, hippocampus and striatum GIT2 expression was increased at the middle age time-point but then decreased with advanced age (Fig. 5D). A qualitatively similar age- and tissue-dependent expression profile was observed with GIT2s, with respect to unidirectional increases in the hypothalamus, brainstem, cerebellum, cortex, and pituitary as well as bimodal regulation in the hindbrain, hippocampus, and striatum (Fig. 5E). As our primary identification of the proteomic alterations in response to aging were identified in the hypothalamus, we then compared the age-dependent GIT2 and GIT2s expression changes in various regions of the brain relative to this important core tissue (GIT2-Fig. 5F–H; GIT2s-Fig. 5I–K). Relative to hypothalamic expression, there was a subtle difference between GIT2 and GIT2s expression across the brain regions studied, *i.e.* hypothalamic GIT2s expression was significantly higher than in all other brain regions (Fig. 5I), while GIT2 expression was nearly identical between the cortex and hypothalamus (Fig. 5F). In addition, there was more tissue quantitative variation in GIT2 compared to GIT2s when expression was measured relative to that in the hypothalamus. In the middle-aged brain tissues, a greater similarity of general GIT2 and GIT2s expression profiles were detected (Fig. 5G, 5J). However, of specific note was the profound relative elevation of pituitary expression levels of GIT2/GIT2s compared to the hypothalamus. In addition to this, the relatively strong expression of cortex and hindbrain GIT2 (relative to the hypothalamus) in the young receded in the middle-aged animals (Fig. 5G). When the oldest time point of GIT2/GIT2s expression, relative to the hypothalamus, was assessed (Fig. 5H, 5K), the most notable effect was again the successive -and statistically-significant- overexpression of pituitary GIT2/GIT2s compared to the hypothalamus. Therefore the two most consistent and profound age-related changes in GIT2 and GIT2s expression occurred in the pituitary and the hypothalamus. Both of these central nervous system organs share a common functional axis; and hence this may not be unexpected. In addition, both of these organs serve as connective nodes between the central nervous system and the peripheral endocrine systems that control major peripheral biological systems such as reproduction and global energy metabolism. We therefore next decided to investigate whether these central nervous system tissue changes in GIT2/GIT2s expression were mirrored in peripheral target organs linked to energy metabolism.

### Functional metabolic profile of aged animals

**Figure 6:**
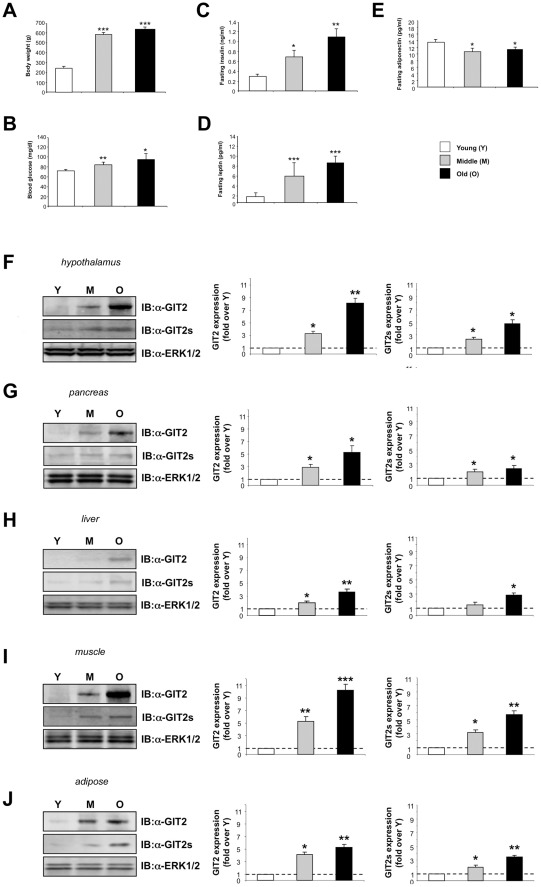
Correlation of GIT2 and GIT2s expression with metabolic phenotype changes in aging rats. (A) Age-dependent changes in mean body weight for the multiple age ranges of rats. (B) Fasting blood glucose measurements for the multiple age ranges of rats. (C) Fasting plasma insulin measurements for the multiple age ranges of rats. (D) Fasting plasma leptin measurements for the multiple age ranges of rats. (E) Fasting plasma adiponectin measurements for the multiple age ranges of rats. (F) GIT2 and GIT2s age-dependent expression in young (Y), middle-aged (M), and old rat hypothalamic extracts. The associated histograms depict the mean ± SEM of the GIT2 and GIT2s expression in middle-aged (grey bars) and old (black bars) animals relative to the young (white bars) samples. (G) GIT2 and GIT2s age-dependent expression in young (Y), middle-aged (M), and old rat whole pancreatic extracts. The associated histograms depict the mean ± SEM of the GIT2 and GIT2s expression in middle-aged (grey bars) and old (black bars) animals relative to the young (white bars) samples. (H) GIT2 and GIT2s age-dependent expression in young (Y), middle-aged (M), and old rat whole liver extracts. The associated histograms depict the mean ± SEM of the GIT2 and GIT2s expression in middle-aged (grey bars) and old (black bars) animals relative to the young (white bars) samples. (I) GIT2 and GIT2s age-dependent expression in young (Y), middle-aged (M), and old rat hind-limb skeletal muscle extracts. The associated histograms depict the mean ± SEM of the GIT2 and GIT2s expression in middle-aged (grey bars) and old (black bars) animals relative to the young (white bars) samples. (J) GIT2 and GIT2s age-dependent expression in young (Y), middle-aged (M), and old rat somatic adipose tissue extracts. The associated histograms depict the mean ± SEM of the GIT2 and GIT2s expression in middle-aged (grey bars) and old (black bars) animals relative to the young (white bars) samples. Statistical significance is as follows: * = p<0.05, ** = p<0.01, *** = p<0.001.

As the hypothalamus is closely associated with managing energy-related systems in the brain and periphery, we first assessed the circulating levels of hormones involved in energy metabolism across the age-span of the rats. We noted a progressive increase in body mass with age in the rats, however the majority of the increases in body weight primarily occurred between the young and middle-aged time point (Fig. 6A). We noted a significant and progressive increase in fasting glucose levels (Fig. 6B), fasting insulin levels (Fig. 6C), and fasting leptin levels (Fig. 6D). As expected, with the observed age-dependent increase in body mass, we observed a significant age-dependent reduction in fasting adiponectin levels (Fig. 6E). The age-span of the animals employed in this study thus demonstrated a common pattern often observed in humans across their lifetime, *i.e.,* progressive weight gain and potentially disrupted energy metabolism linked to insulin resistance. When we assessed the age-dependent expression of GIT2 and GIT2s in tissues associated with a strong energy-metabolism focus (pancreas, liver, skeletal muscle, and adipose tissue), we found a similar age-dependent trend to that seen in the hypothalamus. Strongly reminiscent of the age-dependent increases of GIT2/GIT2s in the hypothalamus (Fig. 6F), we found a progressive age-dependent increase of GIT2/GIT2s expression in the pancreas (Fig. 6G), liver (Fig. 6H), skeletal muscle (Fig. 6I) and adipose tissue (Fig. 6J). Therefore it seems that the progressive alteration in GIT2 expression in the hypothalamus is mirrored in multiple peripheral tissues associated with somatic energy metabolism.

## Discussion

In this study, we examined alterations in protein signaling networks in the hypothalamus of young, middle-aged, and old rats in order to gain a better understanding of the functional alterations that occur in the hypothalamus with aging. We have used novel combinatorial bioinformatic techniques to identify potentially pivotal factors that may control fundamental physiological processes during the aging process, *e.g.* neuronal function and survival, as well as somatic energy metabolism.

Using unbiased proteomic approaches, we identified a large number of significant alterations in proteins in the hypothalamus that link both neurological regulation and somatic metabolic status (Fig. 1–2). Our protein-centered study complements previous data generated from hypothalamic investigations. Jiang and colleagues investigated the relationship between aging and gene expression in the hypothalami and cortices of young and old BALB/c mice (2 and 22 months of age, respectively) using high-density oligonucleotide arrays. In the hypothalamus from old mice, there was an overall up-regulation of proteins involved in the mitochondrial respiratory chain, neuronal structure, and protein processing enzymes such as caspase-6 (Casp6: [24]). Caspase 6 belongs to a family of cysteine proteases involved in neuronal apoptosis and remodeling mechanisms [25], [26]. Additionally, caspase-6 also contributes to the processing of amyloid precursor protein and the deposition of Alzheimer's disease-related amyloid in the brain [27]. In accordance with this earlier study, we also observed up-regulation of Casp6 expression in the hypothalami of old rats (Tables S1, S2). Additional caspases (Casp3, 7, 8, and 12) were also found to be up-regulated in the aged rats. Expression of caspase-2 and -3 has been found to be increased in the brains of Alzheimer's patients [28], strongly implicating a role for caspases in age-related neurodegenerative disorders. In addition to alterations in caspases, we found a strong cytoskeletally-linked alteration in hypothalamic proteins, *e.g.* proline-rich tyrosine kinase 2 (Pyk2) and focal adhesion kinase (FAK) (Fig. 1). Both of these non-receptor tyrosine kinases are strongly associated with the aging process and demonstrate expression changes in response to protracted oxidative stress exposure as well as to excitoxicity, both of which are highly characteristic facets of aging [29]–[33]. While we observed a strong age-dependent elevation of both FAK and Pyk2, interestingly we found a simultaneous decrease in another protein associated with cytoskeletal function, the microtubule-associated protein 2 (Map2) (Fig. 1). Stable neuronal Map2 expression has previously been demonstrated to be altered in the aging process [34], [35] as well as neurological disorders such as Alzheimer's disease [36], [37] and Downs Syndrome [38]. It is possible therefore that with reduced cellular integrity, *e.g.* with Map2 reductions, an attempt to ameliorate this enhanced activity/expression of focal adhesion kinases (Pyk2/FAK) is effected to manage this. It is possible therefore that the expression profile of many proteins may demonstrate a complex relationship to aging or aging-related pathophysiologies, *e.g*. protein changes with time may be causative or reactive in nature [5]. With respect to this, we observed, similar to Map2, a significant age-related reduction in cellular levels of the kinase v-akt murine thymoma viral oncogene homolog 1 (Akt-1). This finding is similar to previous reports associating altered Akt expression in the aging process [39]–[42]. This kinase typically forms a crucial role in cell survival pathways associated with cell surface receptors, including G protein coupled receptors and receptor tyrosine kinases systems such as the insulin/insulin-like growth factor 1 (IGF-1) receptor [43]–[46]. However, as with the diverse nature of protein expression responses to aging/pathology, kinases such as Akt-1 are also associated with diverse activity as they also help coordinate cell survival pathways and apoptotic processes as well [47]–[49]. Therefore, our finding of reduced Akt expression in the hypothalamus with aging may represent both pathophysiological actions as well as reactive/protective ones (Fig. 1). Akt-1 forms a vital component in signaling pathways linked to somatic energy metabolism (*e.g.* insulin/IGF-1 receptor pathway); therefore it is also likely that this hypothalamic expression alteration may also be connected to the alterations in global metabolism seen in the aging rats (Fig. 6).

While significant alterations in individual proteins can generate important information concerning the *gestalt* molecular activity changes in a specific tissue with aging, the functional group clustering of these proteins is more likely to yield physiologically-relevant data. We performed two independent modes of unbiased bioinformatic clustering of the proteins that were significantly altered in the hypothalamus with increasing age. Firstly, we demonstrated using KEGG signaling pathway clustering that age-related proteomic changes in the hypothalamus were potentially associated with neurodegenerative phenotypes (Alzheimer's disease, Parkinson's disease, Huntington's disease), cognitive and neurodevelopmental function (axon guidance, long-term potentiation, Notch signaling, Wnt signaling pathway), dysglycemia (Type II diabetes mellitus, insulin signaling), and neuronal cytoskeletal remodeling (Focal adhesion, Gap junction, Regulation of actin cytoskeleton) (Fig. 3). Using a parallel GO term-based clustering process of the significantly-altered proteins detected in the aging hypothalamus, we also found similar phenotypic functional predictions. For example, GO term-based functional clustering revealed significant enrichment of GO term groups associated with regulation of subcellular organelle and protein complex regulation (Macromolecular complex, intracellular membrane-bound organelle), cellular homeostasis/stress responses (Regulation of cell proliferation, Response to stress), cell signaling kinase cascade activity (Cyclin-dependent protein kinase inhibitor activity, Nitric oxide synthase activity), and neuronal cytoskeletal development (Cytoskeletal protein binding, Synapse, Neuron projection) (Fig. S1). Therefore, in both cases we found that a strong concordance between KEGG and GO term analysis concerning age-dependent alteration of pathways linked to neuronal remodeling, cellular stress response and energy metabolism (from the molecular level, *e.g*. kinase activity, up to the somatic level, *e.g.* glucose regulation) was evident. As one of the initial goals of this study was to generate mechanisms to identify potential keystone protein factors that may integrate and control these multiple synergistic activities occurring in the aging hypothalamus, we then used a novel bioinformatic tool capable of revealing such factors, *i.e.* latent semantic indexing (LSI). LSI specifically facilitates the mathematical discovery of novel connections between distinct input textual terms and genomic/proteomic factors. In this case we employed a novel combinatorial approach to look for convergence between multiple significantly-populated KEGG and GO term groups with a whole-proteome protein reference set. If indeed important keystone proteins linking energy metabolism and neurophysiological regulation exist, it is likely that they would possess a strong cross-correlation with multiple KEGG or GO term groups generated by the direct significant protein expression data. Using LSI heatmap matrix generation, we were able to identify protein factors linked to the greatest numbers of predicted functional aspects of the aging hypothalamic protein network (Fig. 4; Fig. S2). When numerically ranking the proteins that were predicted to possess the most number of functional correlations with KEGG and GO terms, we found only one protein common to both forms of analysis: GIT2. This protein therefore could potentially possess multidimensional roles in linking neuronal and energy-regulatory functions in the aging process.

Our combinatorial informatics process allowed us to identify a potentially important keystone factor for aging, without even its initial detection in the antibody array screen. The antibody screen is a standard platform that contains functionally important signaling proteins that are evenly distributed between both multiple GO and KEGG term annotations. Therefore, the array provides a simple and transferrable platform for initial signaling investigation of complex physiological events, *e.g*. aging. Using our combinatorial informatics approach, we were able to expand the initial hypothalamic protein signature to other functionally-related proteins using a broad range of significantly-regulated KEGG or GO pathways chosen to represent all of the diverse functionally-predicted groups. In essence the phenotypic signature of the tissue, created using the array platform, can be extended out to the functional predictions (GO, KEGG and LSI) and subsequently triangulated to discover factors that may possess strong convergent roles in the specific biological process under study.

While a powerful tool, there are several mitigating limitations to the employment of LSI in biological informatics. LSI-based algorithms primarily attempt to uncover latent connections in text, *e.g*. scientific abstracts. Therefore often there is a spectrum of statistical strength in these uncovered connections, form the very strong to the tenuous. In addition, the linkage process is purely associative and does not include specific functional or regulatory information. To compensate for these issues, we have firstly used a multidimensional heatmap method, in which tenuous and randomly-linked proteins are unlikely to rank as highly as proteins with a recurrent association with multiple functional outputs (Fig. 5). For this study a representative group of both KEGG pathways and GO term groups were chosen to span all of the functional paradigms generated using the informatic prediction (Fig. 4, S1), without generating inordinately-large data streams for analysis. It is highly likely that with increasing the number of convergent LSI interrogation inputs, additional multidimensional factors may be discovered. However it may be more efficient to not force too much bias onto one LSI interrogation system, *e.g.* KEGG pathways, but rather to use a multiplexed approach in which several additional representative informatic outputs, *e.g.* GO-terms, MeSH-terms, MGI-Mammalian Phenotype data, from distinct bioinformatic sources are used for the discovery of convergent proteins in physiological pathways. In addition to these mainly technical considerations, it is always important to use standard validation processes for expression analysis, of the LSI-identified target factor. It is prudent, and physiologically important, to investigate the expression of the target protein in multiple tissues functionally related to the phenotypic profile under investigation, *e.g*. ‘*neurometabolic*’ aging (Fig. 6). Such validation is vital for discounting the potential for non-specific keystone factor discovery from informatic workflows.

The GIT2 protein was originally identified as a factor associated with connecting GPCRs to monomeric G proteins that control cytoskeletal remodeling [50]–[52]. The ability to mediate efficient neuronal remodeling is one of the most important factors in controlling both short-term memory formation and maintaining neural networks linked to both cognitive functions and stress-response processes [19], [53]–[58]. If GIT2 does indeed represent a fundamental protein in the aging/energy regulation process, then we may expect to identify age-related alterations of this factor in multiple tissues involved in neural and endocrine/energy regulatory networks. We found that in a wide variety of central nervous system tissues (the hypothalamus, brainstem, cerebellum, cortex, and pituitary), a strong unidirectional increase of GIT2 expression occurred with advancing age (Fig. 5). More complex, bi-directional age-regulated changes in GIT2 expression were seen in the hindbrain, striatum, and hippocampus (Fig. 5). The specific reasons for these varied modes of GIT2 expression control may be highly complex and will form the basis of future studies. In addition to a strong age-dependent control of GIT2 expression in the CNS, we also investigated whether GIT2 expression was elevated in peripheral tissues linked with somatic energy metabolism. Analysis of GIT2 expression in pancreatic, liver, skeletal muscle, and adipose tissue demonstrated a strong unidirectional, age-dependent elevation of GIT2 and GIT2s expression, reminiscent of that observed in the hypothalamus (Fig. 6). These age-dependent expression changes in GIT2 therefore could potentially be associated with the gross endocrine hormonal age-dependent changes that we observed in these animals, *i.e.* increasing bodyweight, increasing fasting blood glucose, insulin, and leptin levels, and decreasing adiponectin levels (Fig. 6). All of these hormonal parameters are classically associated, in humans and experimental animals, with advancing age and poor global metabolic health [59]–[67]. The exact properties of GIT2 that render it a potential keystone in the age-dependent modulation of neuronal and metabolic functional networks remain to be elucidated. It is likely that the role of GIT2 in these varied tissues may be an evolutionarily-conserved mechanism that allows the coherent integration of metabolic and sensory/cognitive functions. Some of the first fundamental aging studies using simple experimental organisms, such as *C. elegans*, strongly demonstrated the vital role of coherent energy maintenance in controlling longevity [68], [69]. Many of these proteins that were first identified to strongly control aging, *e.g.* insulin/insulin-like growth factor-1 receptor, Akt-1, and phosphatidylinositol 3-kinases (PI-3K), also possess important neuroprotective actions (vital for healthy neuronal aging) in both peripheral and central nervous tissue in many species [70]–[73]. While these proteins linking metabolic and neurological health were first identified in simple organisms like the nematode, specific genetic ablation of components of this family in adipose tissue, *i.e.* the fat-targeted insulin receptor knockout, engenders an enhanced lifespan and stress resistance in mice possessing this genotype [74]. Therefore, based upon our evidence it is possible that GIT2 could also be associated with the group of proteins linking energy metabolism with life/healthspan regulation. This hypothesis will be investigated further in future studies.

Our novel combinatorial bioinformatic approaches have allowed us to analyze potentially crucial structural components in relatively small hypothalamic protein networks that control age-related neuronal and metabolic homeostasis. Based upon our rigorous analyses, we show that endogenous physiological responses to aging may be strongly orchestrated by the expression level of the GIT2 protein. The relevance of the hypothalamic expression level of this protein to the aging process in both neuronal and energy-controlling tissues reinforces the importance of this organ in the potential future development of targeted pharmacotherapeutics designed to interdict a multitude of age-related disorders.

## Materials and Methods

### Animal care and use

Male Sprague Dawley rats were used for this study: 2–3 month old ‘*young*’ rats (n = 8); 10–12 month old ‘*middle’* aged rats (n = 8); 24–26 month old ‘*old*’ rats (n = 8). All animals were humanely euthanized using isoflurane anesthesia, and the following tissues were collected from each animal: hypothalamus, pituitary, hippocampus, cortex, striatum, cerebellum, hindbrain, brain stem, pancreas, liver, skeletal muscle, adipose tissue. All animal testing procedures were approved by the Animal Care and Use Committee of the National Institute on Aging (NIA) under NIA protocol number 293-LNS-2011. Tissues were snap frozen at −80°C following dissection. Additionally, approximately 2 mL of blood was collected from each animal into tubes coated with EDTA. Isolated hypothalami, pancreata, liver, hind-limb skeletal muscle, and subcutaneous fat from each rat were individually fractionated into four major subcellular compartments (cytoplasmic, plasma membrane, large organelles (*e.g.* mitochondria and the actin cytoskeleton) using a Q-proteome tissue fractionation kit according to the manufacturer's instructions (Qiagen Corporation, Valencia, CA). The protein concentrations for all four subcellular fractions of the hypothalami were then normalized to at least 0.5 mg/mL using a BCA protein estimation protocol according to manufacturer's instructions (ThermoScientific, Rockford, IL).

### Cell signaling antibody microarray

Preparation of protein samples, Cy3/Cy5 dye labeling (GE Healthcare, Waltham, MA), application to the Panorama^®^ Cell Signaling Array chip, and data analysis were performed as previously described [2] according to the manufacturer's instructions using the proprietary solutions and equipment provided in the kit (Sigma, St. Louis, MO). Individually-extracted cytoplasmic samples from the rats were equally pooled into three categories according to age: young (Y), middle-aged (M), and old (O). Panorama chips were performed in triplicate for the following direct comparisons: middle (M) vs. young (Y); old (O) vs. young (Y). Label swaps for the dye-sample combinations were made and the whole series of experiments repeated again (n = 3). The differential fluorescent signal intensity for each antibody spot was recorded using a multimodal phosphorimager scanning at a 10μm spot pattern (Typhoon 9410, GE Healthcare, Piscataway, NJ). In order to be considered for further analysis, the recorded expression ratios (estimated through Cy3:Cy5 fluorescence ratios) between M vs. Y or O vs. Y had to be significantly different (greater than or less than) from unity (p<0.05, non-paired Student's t-test: GraphPad Prism version 5.0, San Diego, CA). Validations of randomly selected up-regulated and down-regulated proteins identified by the microspotted antibodies were performed using standard Western blotting procedures both with the pooled and individual hypothalamic samples.

### Western blotting procedures

Specific proteins were resolved using one-dimensional gel electrophoresis. Hypothalamic cytoplasmic extracts (containing 15 µg of total protein) were run on 4–12% Bis-Tris polyacrylamide gels (Invitrogen, Carlsbad, CA) before electrotransfer to a polyvinylenedifluoride (PVDF) membrane (NEN Life Sciences, Boston, MA). PVDF membranes were blocked for one hour at room temperature in 4% non-fat milk (Santa Cruz Biotechnology, Santa Cruz, CA) before blotting. Specific antisera employed were obtained from the following sources: proline-rich tyrosine kinase 2 (Pyk2), G protein-coupled receptor kinase interacting protein 2-short form (GIT2s), focal adhesion kinase (FAK), G protein-coupled receptor kinase interacting protein 1 (GIT1) (BD Biosciences, San Jose, CA); myc, microtubule-associated protein 2 (Map2), Ran/TC4, vinculin (Vcl), nitric oxide synthase-1 (Nos1) (Santa Cruz Biotechnology, Santa Cruz, CA); Akt, caspase 3, extracellular signal-regulated kinase 1/2 (ERK1/2), p21 protein (Cdc42/Rac)-activated kinase 1 (PAK1), Rho guanine nucleotide exchange factor (GEF) 7 (β-PIX) (Cell Signaling Technology, Danvers, MA); junction plakoglobin (Jup) (Sigma Aldrich, St. Louis, MO); G protein-coupled receptor kinase interacting protein 2 (GIT2) (NeuroMab, San Jose, CA); 2′,3′-cyclic nucleotide 3′ phosphodiesterase (Cnp1) (Abnova, Walnut, CA). Detection of primary immune complexes were performed with subsequent application of a 1∶10,000 dilution of an alkaline phosphatase-conjugated, species-specific secondary antibody (Sigma Aldrich, St. Louis, MO) followed by enzyme-linked chemifluorescence exposure (GE Healthcare, Pittsburgh, PA) and digital quantification using a Typhoon 9410 phosphorimager with ImageQuant 5.2 L software (GE Healthcare, Pittsburgh, PA). Standard Coomassie staining methods were used to ensure equal protein concentrations of the SDS-PAGE gel. Gels were fixed for 30 minutes by immersion in 10% v/v glacial acetic and 30% v/v methanol. G250 Coomassie was then added for a one hour incubation period with agitation. After staining, the gel was washed in destaining solution containing 3% glacial acetic acid until the stain background was adequately removed. For GIT2 immunoprecipitation experiments, GIT2 antisera were employed (NeuroMab, San Jose, CA) with overnight incubation at 4°C before addition of 30 µL of Protein G Plus/Protein A Agarose (EMD Biosciences) for 1 hour and subsequent collection of GIT2 immunoprecipitates with low-speed centrifugation. Phosphotyrosine content of gel-resolved proteins was assessed using a 1∶1,000 dilution of anti-phosphotyrosine PY20 (Santa Cruz Biotechnology, CA) sera.

### Bioinformatics Analyses

Protein sets generated from the antibody microarray were analyzed using WebGestalt (http://bioinfo.vanderbilt.edu/webgestalt/). Significant clustering of proteins into GO (gene ontology) terms and KEGG (Kyoto encyclopedia of genes and genomes) pathway groups was performed using a hypergeometric test of significance (p<0.05). At least two independent proteins were required for significant population of specific GO groups or KEGG pathways. The relative profundity of the GO term or KEGG pathway population was measured as described previously using a ‘hybrid score’ approach [2]. Briefly, hybrid scores for GO term groups or KEGG pathways were calculated by multiplication of the negative log_10_ of the enrichment probability (P) with the enrichment ratio (R). Latent semantic indexing (LSI) bioinformatic analyses were performed using GeneIndexer (Computable Genomix LLC, https://computablegenomix.com/geneindexer) as described previously [2], [3].

### Plasma hormone analysis

Approximately 2 mL of blood was collected from each animal into Vacutainer^TM^ tubes coated with EDTA (BD Biosciences, San Jose, CA). Blood glucose concentration was measured as previously described [75]. Samples were centrifuged (3000 rpm, 30 minutes, 4°C) and plasma from each tube was collected and then stored at −80°C until used. When required, samples were thawed overnight at 4°C prior to analysis. The plasma levels of specific metabolic hormones were measured using enzyme-linked immunosorbent assays (ELISA) (Millipore, Billerica, MA). The hormones measured were insulin, leptin, and adiponectin. Each sample was run in duplicate on a 96-well plate. Analysis of quality control standards provided in the kits met expectations, validating the accuracy of the panels.

## Supporting Information

Figure S1 **Gene Ontology term analysis of aging-related hypothalamic proteins.** Proteins significantly regulated in middle (M) or old (O) aged animals compared to young (Y) animals were used as input data for Gene Ontology (GO) term population analysis. (A) Venn diagram analysis of middle-aged (grey line) and old-aged (black line) significantly-regulated GO term groups demonstrated 79 common GO terms between old and middle-aged tissues. The common (79) significantly populated pathways for middle-aged (grey bars) or old aged (black bars) animals were then rationally clustered into subgroups focused upon cell structure/function (B), cell cycle regulation (C), enzyme activity (D), and neurophysiological architecture (E). For each significantly-populated GO term group a ‘hybrid’ score was generated which represents the −log_10_ of the enrichment probability multiplied with the relative enrichment factor compared to the background proteomic expression.
(TIF)

Figure S2 **Latent semantic indexing** (**LSI**) **correlations of Gene Ontology groups with proteins.** (A) Latent semantic indexing (LSI) interrogation matrix between input significantly-regulated Gene Ontology (GO) term groups. Colored blocks represent the individual LSI implicit correlation of the specific protein (vertically organized on left of heatmap: 1–2524 – see Table S20) with the respective GO term (1-Actin filament binding, 2-Anatomical structural development, 3-Cyclin-dependent protein kinase inhibitor activity, 4-Regulation of cell proliferation, 5-Cytoskeletal protein binding, 6-Macromolecular complex, 7-Nitric oxide synthase activity, 8-Synapse, 9-Response to stress, 10-Intracellular membrane-bound organelle, 11-Regulation of programmed cell death, 12-Protein kinase activity). The number of KEGG signaling pathway correlations for each protein is indicated by the color of the respective heatmap blocks (7 correlations-yellow; 6 correlations-green; 5 correlations-light blue; 4 correlations-dark blue; 3 correlations-purple; 2 correlations-grey). (B) Mean ± SEM for the total implicitly-correlating proteins for each of the 12 input GO term groups. (C) Box and whisker plot with 1–99% statistical cut-offs (GraphPad Prism) of the number of specific correlations to GO term groups each protein possessed. Twelve proteins demonstrated a statistically-significantly greater number of GO term group correlations compared to the total protein mean number of correlations (*** = p<0.001). (D) Expanded heatmap identification of specific proteins possessing a significantly greater number of GO term group correlations compared to the mean number of GO term group correlations for all implicit proteins. (E) Mean ± SEM of LSI correlation scores (across all 7 correlations) for Ccdc88a, Kank1, Pcnp, Plekho1, Rsu1, Tfpt, GIT2, Plrg1, and Zdhhc16.
(TIF)

Figure S3 **Age-dependent alteration in GIT2 phosphotyrosine content.** Anti-GIT2 immunoprecipitates were performed on hypothalamic lysates from young (Y), middle-aged (M) or old (O) rats. The immunoprecipitates were then assessed for GIT2 content using western blot, and then based upon this normalized for a subsequent western that ensured equal GIT2 loading from the specific immunoprecipitate. This western blot was then probed with a generic anti-phosphotyrosine anti-sera (anti-P-Tyr). The associated histogram indicates GIT2 phosphotyrosine content. Data represented as mean ± SEM, n = 3, * = p<0.05).
(TIF)

Figure S4 **Age-dependent alteration in the expression of an ‘intermediate’ mass GIT2 isoform.** (A) Using specific anti-GIT2 sera, the primary species observed in the hypothalamus and other central nervous system regions were 85 kDA (1.) and 55 kDa (3.). A prominent intermediate mass GIT2 immunoreactive species was also observed and was estimated to be 60–62 kDa (2.). (B) Age-dependent expression variation of the intermediate mass (60–62kDa) GIT2 immunoreactive species across multiple regions of the central nervous system. Expression profile, relative to that in the hypothalamus, of the intermediate (60–62kDa) GIT2 immunoreactive isoform in young (C), middle-aged (D) and old (E) rats. Anti-GIT2 immunoprecipitates were performed on hypothalamic lysates from young (Y), middle-aged (M) or old (O) rats. The histograms depict intermediate GIT2 immunoreactive form expression. Data represented as mean ± SEM, n = 3, * = p<0.05, ** = p<0.01, *** = p<0.001.
(TIF)

Table S1 **Protein expression alterations in middle-aged compared to young rat hypothalamus.** Panorama® Cell Signaling Array platforms were employed to assess the relative expression ratio of individual proteins for middle-aged (M) versus young (Y) rats (M/Y). Expression ratios were calculated from triplicate experiments and the mean and standard error of the mean (SEM) for each protein demonstrating an M/Y ratio using the following criteria: ratio>1.5 and ratio<0.5.
(DOC)

Table S2 **Protein expression alterations in old compared to young rat hypothalamus.** Panorama® Cell Signaling Array platforms were employed to assess the relative expression ratio of individual proteins for old (O) versus young (Y) rats (O/Y). Expression ratios were calculated from triplicate experiments and the mean and standard error of the mean (SEM) for each protein demonstrating an O/Y ratio using the following criteria: ratio>1.5 and ratio<0.5.
(DOC)

Table S3 **Proteins significantly regulated in both middle- and old-aged rat hypothalami compared to young rats.** Panorama® Cell Signaling Array platforms were employed to assess the relative expression ratio of individual proteins for middle-aged/old (M or O) versus young (Y) rats (M/Y or O/Y). Expression ratios were calculated from triplicate experiments and the mean and standard error of the mean (SEM) for each protein demonstrating an O/Y ratio using the following criteria: ratio>1.5 and ratio<0.5.
(DOC)

Table S4 **KEGG signaling pathway enrichment for middle-aged versus young rat protein expression variation.** KEGG signaling pathway enrichment was performed using WebGestalt with the protein set significantly altered in middle aged hypothalami compared to the young controls. KEGG signaling pathway text description, enrichment factor (R), probability of enrichment (P), and the resultant hybrid score (H: −log_10_(P)×R) is represented.
(DOC)

Table S5 **KEGG signaling pathway enrichment for old versus young rat protein expression variation.** KEGG signaling pathway enrichment was performed using WebGestalt with the protein set significantly altered in old-aged hypothalami compared to the young controls. KEGG signaling pathway text description, enrichment factor (R), probability of enrichment (P), and the resultant hybrid score (H: −log_10_(P)×R) is represented.
(DOC)

Table S6 **GO-term enrichment for middle versus young rat protein expression variation.** GO-term enrichment was performed using WebGestalt with the protein set significantly altered in middle aged hypothalami compared to the young controls. Official GO-term codes, the text description of the code as well as the enrichment factor (R), probability of enrichment (P), and the resultant hybrid score (H: −log_10_(P)×R) is represented.
(DOC)

Table S7 **GO-term enrichment for old versus young protein expression variation.** GO-term enrichment was performed using WebGestalt with the protein set significantly altered in middle aged hypothalami compared to the young controls. Official GO-term codes, the text description of the code as well as the enrichment factor (R ), probability of enrichment (P), and the resultant hybrid score (H: −log_10_(P)×R) is represented.
(DOC)

Table S8 **GeneIndexer latent semantic indexing (LSI) of significantly-regulated ‘Regulation of actin cytoskeleton’ KEGG pathway.** Using the KEGG signaling pathway ‘Regulation of actin cytoskeleton’ as an input term, a list of the top 1000 implicitly-correlated (LSI correlation score >0.1) was generated using a full genome background list.
(DOC)

Table S9 **GeneIndexer latent semantic indexing (LSI) of significantly-regulated ‘Chemokine signaling’ KEGG pathway.** Using the KEGG signaling pathway ‘Chemokine signaling’ as an input term, a list of the top 1000 implicitly-correlated (LSI correlation score >0.1) was generated using a full genome background list.
(DOC)

Table S10 **GeneIndexer latent semantic indexing (LSI) of significantly-regulated ‘Alzheimer**'**s disease’ KEGG pathway.** Using the KEGG signaling pathway ‘Alzheimer's disease’ as an input term, a list of the top 1000 implicitly-correlated (LSI correlation score >0.1) was generated using a full genome background list.
(DOC)

Table S11 **GeneIndexer latent semantic indexing (LSI) of significantly-regulated ‘Focal adhesion’ KEGG pathway.** Using the KEGG signaling pathway ‘Focal adhesion’ as an input term, a list of the top 1000 implicitly-correlated LSI correlation score >0.1) was generated using a full genome background list.
(DOC)

Table S12 **GeneIndexer latent semantic indexing (LSI) of significantly-regulated ‘MAPK signaling’ KEGG pathway.** Using the KEGG signaling pathway ‘MAPK signaling’ as an input term, a list of the top 1000 implicitly-correlated (LSI correlation score >0.1) was generated using a full genome background list.
(DOC)

Table S13 **GeneIndexer latent semantic indexing (LSI) of significantly-regulated ‘Gap junction’ KEGG pathway.** Using the KEGG signaling pathway ‘Gap junction’ as an input term, a list of the top 1000 implicitly-correlated (LSI correlation score >0.1) was generated using a full genome background list.
(DOC)

Table S14 **GeneIndexer latent semantic indexing (LSI) of significantly-regulated ‘GnRH signaling’ KEGG pathway.** Using the KEGG signaling pathway ‘GnRH signaling’ as an input term, a list of the top 1000 implicitly-correlated (LSI correlation score >0.1) was generated using a full genome background list.
(DOC)

Table S15 **GeneIndexer latent semantic indexing (LSI) of significantly-regulated ‘Long term potentiation’ KEGG pathway.** Using the KEGG signaling pathway ‘Long term potentiation’ as an input term, a list of the top 1000 implicitly-correlated (LSI correlation score >0.1) was generated using a full genome background list.
(DOC)

Table S16 **GeneIndexer latent semantic indexing (LSI) of significantly-regulated ‘Notch signaling’ KEGG pathway.** Using the KEGG signaling pathway ‘Notch signaling’ as an input term, a list of the top 1000 implicitly-correlated (LSI correlation score >0.1) was generated using a full genome background list.
(DOC)

Table S17 **GeneIndexer latent semantic indexing (LSI) of significantly-regulated ‘VEGF signaling’ KEGG pathway.** Using the KEGG signaling pathway ‘VEGF signaling’ as an input term, a list of the top 1000 implicitly-correlated (LSI correlation score >0.1) was generated using a full genome background list.
(DOC)

Table S18 **GeneIndexer latent semantic indexing (LSI) of significantly-regulated ‘p53 signaling’ KEGG pathway.** Using the KEGG signaling pathway ‘p53 signaling’ as an input term, a list of the top 1000 implicitly-correlated (LSI correlation score >0.1) was generated using a full genome background list.
(DOC)

Table S19 **GeneIndexer latent semantic indexing (LSI) of significantly-regulated ‘Calcium signaling’ KEGG pathway.** Using the KEGG signaling pathway ‘Calcium signaling’ as an input term, a list of the top 1000 implicitly-correlated (LSI correlation score >0.1) was generated using a full genome background list.
(DOC)

Table S20 **GeneIndexer latent semantic indexing (LSI) correlation matrix for twelve input KEGG signaling pathway terms.** The input text terms are numerated as follows: 1-*Regulation of actin cytoskeleton*, 2-*Chemokine signaling*, 3-*Alzheimer's disease*, 4-*Focal adhesion*, 5-*MAPK signaling*, 6-*Gap junction*, 7-*GnRH signaling*, 8-*Long term potentiation*, 9-*Notch signaling*, 10-*VEGF signaling*, 11-*p53 signaling*, 12-*Calcium signaling*. An implicit correlation (LSI score >0.1) of the specific protein and the respective input KEGG terms is denoted by a 1. When no correlation occurs, a 0 is present.
(DOC)

Table S21 **GeneIndexer latent semantic indexing (LSI) of significantly-regulated ‘Actin filament binding’ GO term group.** Using the GO term group ‘Actin filament binding’ as an input term, a list of the top 1000 implicitly-correlated (LSI correlation score >0.1) was generated using a full genome background list.
(DOC)

Table S22 **GeneIndexer latent semantic indexing (LSI) of significantly-regulated ‘Anatomical structural development’ GO term group.** Using the GO term group ‘Anatomical structural development’ as an input term, a list of the top 1000 implicitly-correlated (LSI correlation score >0.1) was generated using a full genome background list.
(DOC)

Table S23 **GeneIndexer latent semantic indexing (LSI) of significantly-regulated ‘Cyclin-dependent protein kinase inhibitor activity’ GO term group.** Using the GO term group ‘Cyclin-dependent protein kinase inhibitor activity’ as an input term, a list of the top 1000 implicitly-correlated (LSI correlation score >0.1) was generated using a full genome background list.
(DOC)

Table S24 **GeneIndexer latent semantic indexing (LSI) of significantly-regulated ‘Regulation of cell proliferation’ GO term group.** Using the GO term group ‘Regulation of cell proliferation’ as an input term, a list of the top 1000 implicitly-correlated (LSI correlation score >0.1) was generated using a full genome background list.
(DOC)

Table S25 **GeneIndexer latent semantic indexing (LSI) of significantly-regulated ‘Cytoskeletal protein binding’ GO term group.** Using the GO term group ‘Cytoskeletal protein binding’ as an input term, a list of the top 1000 implicitly-correlated (LSI correlation score >0.1) was generated using a full genome background list.
(DOC)

Table S26 **GeneIndexer latent semantic indexing (LSI) of significantly-regulated ‘Macromolecular complex’ GO term group.** Using the GO term group ‘Macromolecular complex’ as an input term, a list of the top 1000 implicitly-correlated (LSI correlation score >0.1) was generated using a full genome background list.
(DOC)

Table S27 **GeneIndexer latent semantic indexing (LSI) of significantly-regulated ‘Nitric oxide synthase activity’ GO term group.** Using the GO term group ‘Nitric oxide synthase activity’ as an input term, a list of the top 1000 implicitly-correlated (LSI correlation score >0.1) was generated using a full genome background list.
(DOC)

Table S28 **GeneIndexer latent semantic indexing (LSI) of significantly-regulated ‘Synapse’ GO term group.** Using the GO term group ‘Synapse’ as an input term, a list of the top 1000 implicitly-correlated (LSI correlation score >0.1) was generated using a full genome background list.
(DOC)

Table S29 **GeneIndexer latent semantic indexing (LSI) of significantly-regulated ‘Response to stress’ GO term group.** Using the GO term group ‘Response to stress’ as an input term, a list of the top 1000 implicitly-correlated (LSI correlation score >0.1) was generated using a full genome background list.
(DOC)

Table S30 **GeneIndexer latent semantic indexing (LSI) of significantly-regulated ‘Intracellular membrane-bound organelle’ GO term group.** Using the GO term group ‘Intracellular membrane-bound organelle’ as an input term, a list of the top 1000 implicitly-correlated (LSI correlation score >0.1) was generated using a full genome background list.
(DOC)

Table S31 **GeneIndexer latent semantic indexing (LSI) of significantly-regulated ‘Regulation of programmed cell death’ GO term group.** Using the GO term group ‘Regulation of programmed cell death’ as an input term, a list of the top 1000 implicitly-correlated (LSI correlation score >0.1) was generated using a full genome background list.
(DOC)

Table S32 **GeneIndexer latent semantic indexing (LSI) of significantly-regulated ‘Protein kinase activity’ GO term group.** Using the GO term group ‘Protein kinase activity’ as an input term, a list of the top 1000 implicitly-correlated (LSI correlation score >0.1) was generated using a full genome background list.
(DOC)

Table S33 **GeneIndexer latent semantic indexing (LSI) correlation matrix for twelve input GO term groups.** The input text terms are numerated as follows: 1-*Actin filament binding*, 2-*Anatomical structural development*, 3-*Cyclin-dependent protein kinase inhibitor activity*, 4-*Regulation of cell proliferation*, 5-*Cytoskeletal protein binding*, 6-*Macromolecular complex*, 7-*Nitric oxide synthase activity*, 8-*Synapse*, 9-*Response to stress*, 10-*Intracellular membrane-bound organelle*, 11-*Regulation of programmed cell death*, 12-*Protein kinase activity*. An implicit correlation (LSI score >0.1) of the specific protein and the respective input KEGG terms is denoted by a 1. When no correlation occurs, a 0 is present.
(DOC)
