# Supplementary figures and images for: GIT2 Acts as a Potential Keystone Protein in Functional Hypothalamic Networks Associated with Age-Related Phenotypic Changes in Rats
Source: PLoS One. 2012 May 14;7(5):e36975. doi: 10.1371/journal.pone.0036975 (PMC3351446; doi:10.1371/journal.pone.0036975)

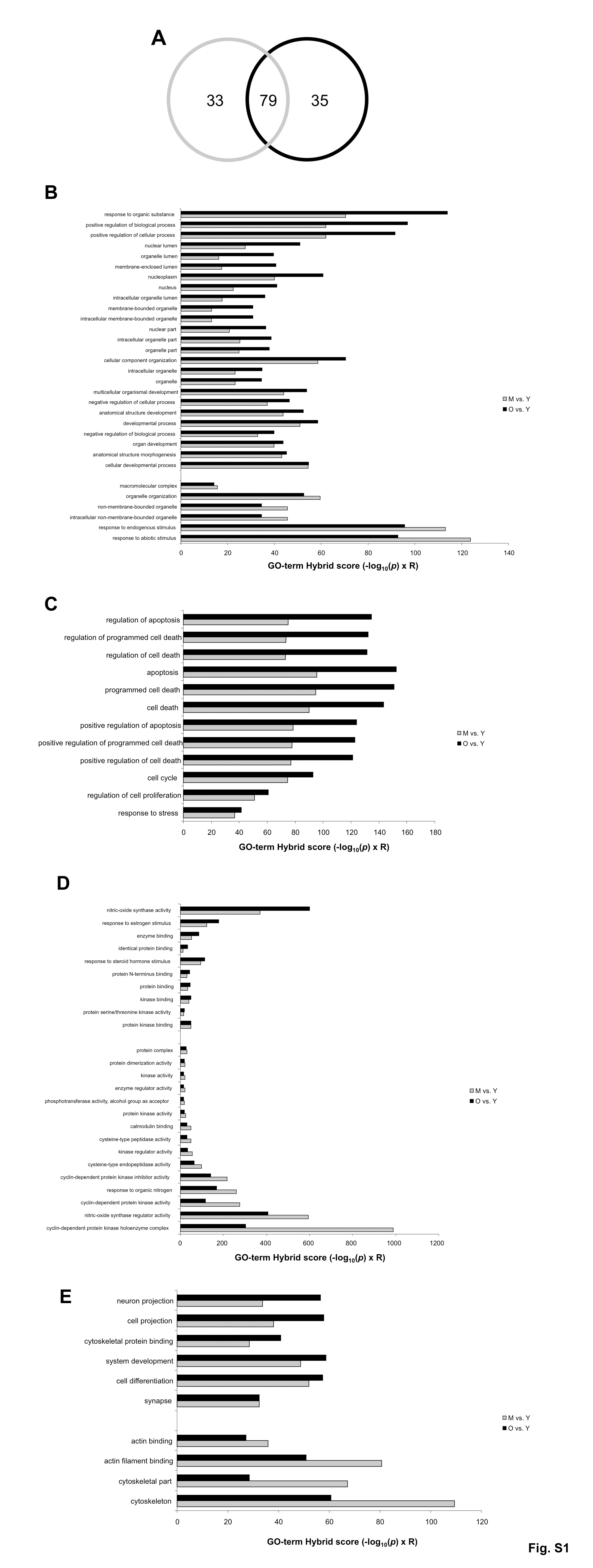

Supplement: Figure S1 — Gene Ontology term analysis of aging-related hypothalamic proteins. Proteins significantly regulated in middle (M) or old (O) aged animals compared to young (Y) animals were used as input data for Gene Ontology (GO) term population analysis. (A) Venn diagram analysis of middle-aged (grey line) and old-aged (black line) significantly-regulated GO term groups demonstrated 79 common GO terms between old and middle-aged tissues. The common (79) significantly populated pathways for middle-aged (grey bars) or old aged (black bars) animals were then rationally clustered into subgroups focused upon cell structure/function (B), cell cycle regulation (C), enzyme activity (D), and neurophysiological architecture (E). For each significantly-populated GO term group a ‘hybrid’ score was generated which represents the −log10 of the enrichment probability multiplied with the relative enrichment factor compared to the background proteomic expression. (TIF) [file pone.0036975.s001.tif]

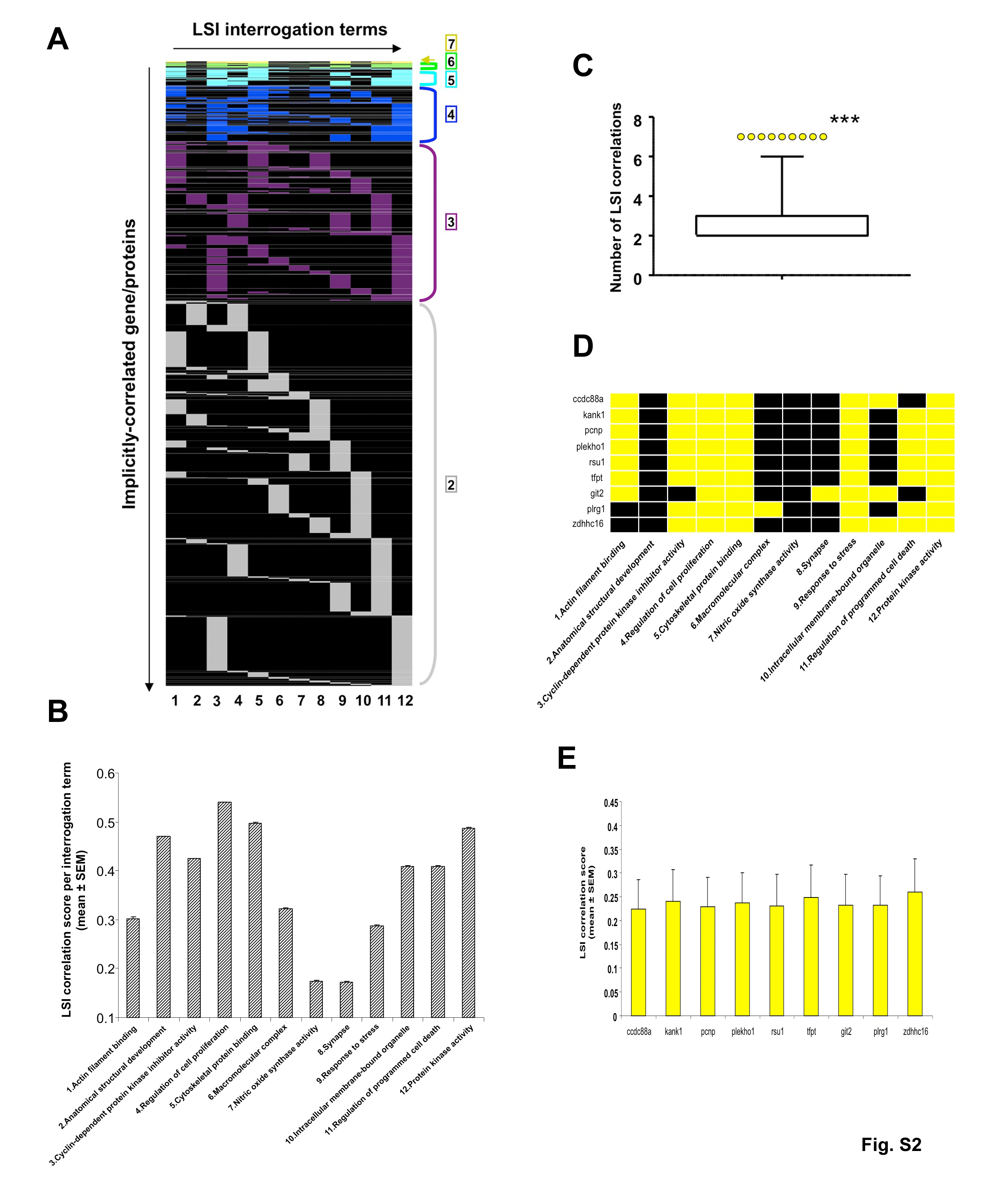

Supplement: Figure S2 — Latent semantic indexing (LSI) correlations of Gene Ontology groups with proteins. (A) Latent semantic indexing (LSI) interrogation matrix between input significantly-regulated Gene Ontology (GO) term groups. Colored blocks represent the individual LSI implicit correlation of the specific protein (vertically organized on left of heatmap: 1–2524 – see Table S20) with the respective GO term (1-Actin filament binding, 2-Anatomical structural development, 3-Cyclin-dependent protein kinase inhibitor activity, 4-Regulation of cell proliferation, 5-Cytoskeletal protein binding, 6-Macromolecular complex, 7-Nitric oxide synthase activity, 8-Synapse, 9-Response to stress, 10-Intracellular membrane-bound organelle, 11-Regulation of programmed cell death, 12-Protein kinase activity). The number of KEGG signaling pathway correlations for each protein is indicated by the color of the respective heatmap blocks (7 correlations-yellow; 6 correlations-green; 5 correlations-light blue; 4 correlations-dark blue; 3 correlations-purple; 2 correlations-grey). (B) Mean ± SEM for the total implicitly-correlating proteins for each of the 12 input GO term groups. (C) Box and whisker plot with 1–99% statistical cut-offs (GraphPad Prism) of the number of specific correlations to GO term groups each protein possessed. Twelve proteins demonstrated a statistically-significantly greater number of GO term group correlations compared to the total protein mean number of correlations (*** = p<0.001). (D) Expanded heatmap identification of specific proteins possessing a significantly greater number of GO term group correlations compared to the mean number of GO term group correlations for all implicit proteins. (E) Mean ± SEM of LSI correlation scores (across all 7 correlations) for Ccdc88a, Kank1, Pcnp, Plekho1, Rsu1, Tfpt, GIT2, Plrg1, and Zdhhc16. (TIF) [file pone.0036975.s002.tif]

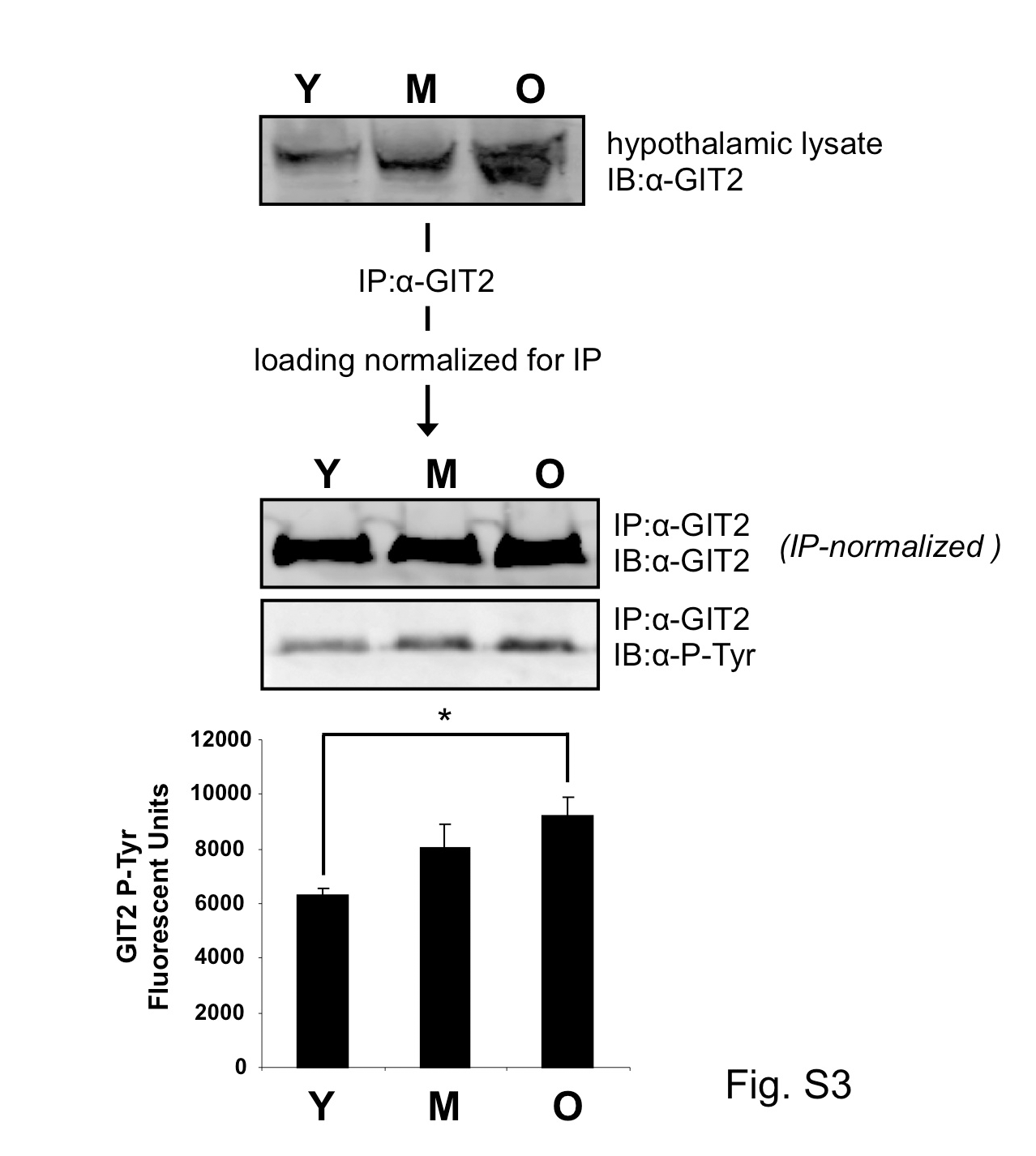

Supplement: Figure S3 — Age-dependent alteration in GIT2 phosphotyrosine content. Anti-GIT2 immunoprecipitates were performed on hypothalamic lysates from young (Y), middle-aged (M) or old (O) rats. The immunoprecipitates were then assessed for GIT2 content using western blot, and then based upon this normalized for a subsequent western that ensured equal GIT2 loading from the specific immunoprecipitate. This western blot was then probed with a generic anti-phosphotyrosine anti-sera (anti-P-Tyr). The associated histogram indicates GIT2 phosphotyrosine content. Data represented as mean ± SEM, n = 3, * = p<0.05). (TIF) [file pone.0036975.s003.tif]

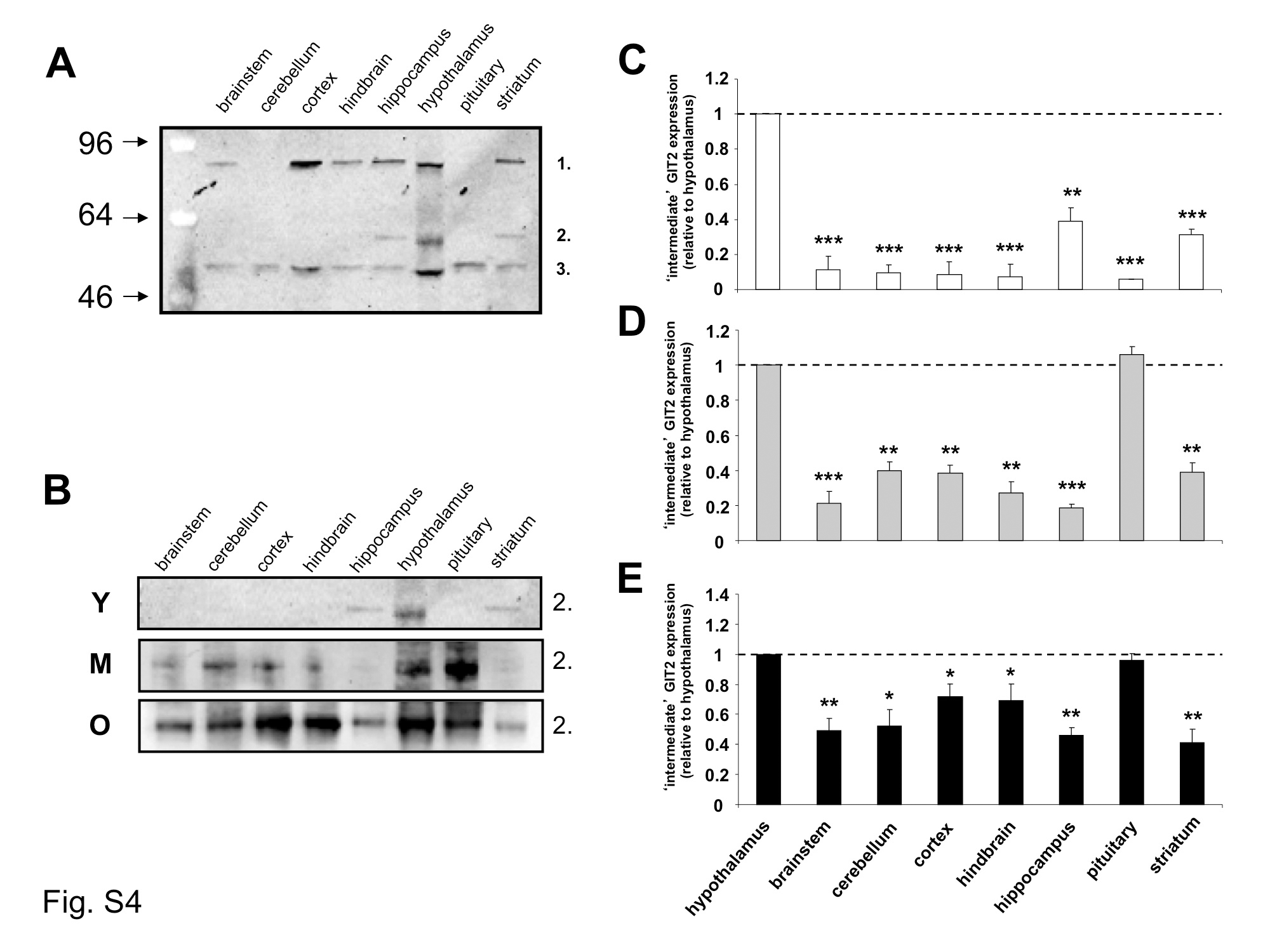

Supplement: Figure S4 — Age-dependent alteration in the expression of an ‘intermediate’ mass GIT2 isoform. (A) Using specific anti-GIT2 sera, the primary species observed in the hypothalamus and other central nervous system regions were 85 kDA (1.) and 55 kDa (3.). A prominent intermediate mass GIT2 immunoreactive species was also observed and was estimated to be 60–62 kDa (2.). (B) Age-dependent expression variation of the intermediate mass (60–62kDa) GIT2 immunoreactive species across multiple regions of the central nervous system. Expression profile, relative to that in the hypothalamus, of the intermediate (60–62kDa) GIT2 immunoreactive isoform in young (C), middle-aged (D) and old (E) rats. Anti-GIT2 immunoprecipitates were performed on hypothalamic lysates from young (Y), middle-aged (M) or old (O) rats. The histograms depict intermediate GIT2 immunoreactive form expression. Data represented as mean ± SEM, n = 3, * = p<0.05, ** = p<0.01, *** = p<0.001. (TIF) [file pone.0036975.s004.tif]
