# Supplementary material for: GIT2 Acts as a Potential Keystone Protein in Functional Hypothalamic Networks Associated with Age-Related Phenotypic Changes in Rats
Source: PLoS One. 2012 May 14;7(5):e36975. doi: 10.1371/journal.pone.0036975 (PMC3351446; doi:10.1371/journal.pone.0036975)
Supplement: Table S1 — Protein expression alterations in middle-aged compared to young rat hypothalamus. Panorama® Cell Signaling Array platforms were employed to assess the relative expression ratio of individual proteins for middle-aged (M) versus young (Y) rats (M/Y). Expression ratios were calculated from triplicate experiments and the mean and standard error of the mean (SEM) for each protein demonstrating an M/Y ratio using the following criteria: ratio>1.5 and ratio<0.5. (DOC) [file pone.0036975.s005.doc]

**Table S1. Protein expression alterations in middle-aged compared to young rat hypothalamus.** Panorama® Cell Signaling Array platforms were employed to assess the relative expression ratio of individual proteins for middle-aged (M) versus young (Y) rats (M/Y). Expression ratios were calculated from triplicate experiments and the mean and standard error of the mean (SEM) for each protein demonstrating an M/Y ratio using the following criteria: ratio> 1.5 and ratio<0.5.

| **Protein** | **Symbol** | **Mean ratio (M/Y)** | **SEM** |
| --- | --- | --- | --- |
| CUGBP, Elav-like family member 1 | Cugbp1 | 29.35 | 0.29942248 |
| cyclin D1 | Ccnd1 | 22 | 0.05941359 |
| protein kinase C, gamma | Prkcg | 15.42410714 | 0.1561084 |
| keratin 13 | Krt13 | 8.850694444 | 0.33208352 |
| histone acetyltransferase 1 | Hat1 | 4.251912568 | 0.24500222 |
| junction plakoglobin | Jup | 4.055555556 | 0.15688361 |
| RAN, member RAS oncogene family | Ran | 3.515789474 | 0.04273 |
| glutamate receptor, ionotropic, N-methyl D-aspartate 2A | Grin2a | 3.33988764 | 0.17576 |
| heterogeneous nuclear ribonucleoprotein H1 (H) | Hnrnph1 | 3.177925785 | 0.37272 |
| H3 histone, family 3B (H3.3B); H3 histone, family 3A pseudogene | H3f3a | 2.703422053 | 0.2457 |
| tropomyosin 1 (alpha) | Tpm1 | 2.452054795 | 0.22742 |
| cyclin A1 | Ccna1 | 2.417009602 | 0.1872 |
| death-domain associated protein | Daxx | 2.263484413 | 0.01172 |
| BCL2-like 1 | Bcl2l1 | 2.257785467 | 0.337 |
| c-myc binding protein | Mycbp | 2.244933921 | 0.1123 |
| E2F transcription factor 1 | E2f1 | 2.229451312 | 0.24575 |
| spectrin, alpha, non-erythrocytic 1 (alpha-fodrin) | Sptan1 | 2.207920792 | 0.017657 |
| kinesin family member 3A | Kif3a | 2.058450385 | 0.214625 |
| tubulin, beta 3 | Tubb3 | 2.048279159 | 0.3058125 |
| cyclin B1 | Ccnb1 | 1.974180735 | 0.181625 |
| gap junction protein, alpha 1, 43kDa | Gja1 | 1.947351599 | 0.347 |
| microtubule-associated protein 1B | Map1b | 1.924426451 | 0.470875 |
| death-associated protein kinase 1 | Dapk1 | 1.87469241 | 0.02207125 |
| protein kinase C, alpha | Prkca | 1.854561879 | 0.1722125 |
| cyclin-dependent kinase 4 | Cdk4 | 1.841792352 | 0.047325 |
| neurofilament, heavy polypeptide | Nefh | 1.804012346 | 0.219625 |
| internexin neuronal intermediate filament protein, alpha | Ina | 1.802415842 | 0.178075 |
| PTK2 protein tyrosine kinase 2 | Ptk2 | 1.785857474 | 0.168375 |
| DNA-damage-inducible transcript 3 | Ddit3 | 1.777063578 | 0.14 |
| caspase 7, apoptosis-related cysteine peptidase | Casp7 | 1.768816856 | 0.205875 |
| synuclein, beta | Sncb | 1.741791045 | 0.1725 |
| myosin VA (heavy chain 12, myoxin) | Myo5a | 1.740389116 | 0.045875 |
| cystatin A (stefin A) | Csta | 1.740000743 | 0.03071875 |
| cyclin-dependent kinase 7 | Cdk7 | 1.712179595 | 0.22125 |
| microtubule-associated protein 1A | Map1a | 1.666232073 | 0.208625 |
| PRKC, apoptosis, WT1, regulator | Pawr | 1.659925468 | 0.16625 |
| leucine carboxyl methyltransferase 2 | Lcmt2 | 1.652316886 | 0.3057125 |
| myosin, heavy chain 9, non-muscle | Myh9 | 1.644329533 | 0.13875 |
| caspase 6, apoptosis-related cysteine peptidase | Casp6 | 1.639203085 | 0.292345 |
| epidermal growth factor receptor | Egfr | 1.622458002 | 0.0255117 |
| phospholipase A2, group IVA (cytosolic, calcium-dependent) | Pla2g4a | 1.61913084 | 0.1267296 |
| histone deacetylase 4 | Hdac4 | 1.592067189 | 0.1735164 |
| caspase 3, apoptosis-related cysteine peptidase | Casp3 | 1.59150088 | 0.13302003 |
| calmodulin regulated spectrin-associated protein 1-l | Camsap1l1 | 1.588822173 | 0.10131 |
| caspase 10 | Casp10 | 1.560322163 | 0.3109821 |
| PTK2B protein tyrosine kinase 2 beta | Ptk2b | 1.552445245 | 0.2539197 |
| catenin (cadherin-associated protein), alpha 1 | Ctnna1 | 1.540101803 | 0.16184273 |
| cyclin-dependent kinase inhibitor 2A (melanoma, p16, inhibit | Cdkn2a | 1.528737076 | 0.10477296 |
| nerve growth factor receptor (TNFRSF16) associated protein 1 | Ngfrap1 | 1.520175648 | 0.0069075 |
| nitric oxide synthase 3 (endothelial cell) | NOS3 | 1.512259593 | 0.00398673 |
| nitric oxide synthase 1 (neuronal) | Nos1 | 0.475366768 | 0.1717 |
| diablo homolog (Drosophila) | Diablo | 0.470289984 | 0.24465 |
| cell division cycle 2 homolog (S. cerevisiae) | Cdc2 | 0.462082481 | 0.1453 |
| v-crk sarcoma virus CT10 oncogene homolog (avian)-like | Crkl | 0.460090287 | 0.2776 |
| tyrosine hydroxylase | Th | 0.457922856 | 0.3767 |
| phospholipase C, gamma 1 | Plcg1 | 0.451839024 | 0.017657 |
| keratin 18; keratin 18 pseudogene 26; keratin 18 pseudogene | Krt18 | 0.450087566 | 0.13777 |
| cyclin-dependent kinase 5, regulatory subunit 1 (p35) | Cdk5r1 | 0.449916799 | 0.03786 |
| cell division cycle 7 homolog (S. cerevisiae) | Cdc7 | 0.433267956 | 0.1757 |
| mitogen-activated protein kinase 1 | Mapk1 | 0.433166114 | 0.14246 |
| mitogen-activated protein kinase-activated protein kinase 2 | Mapkapk2 | 0.406587319 | 0.1347 |
| nitric oxide synthase 2, inducible | Nos2 | 0.393256537 | 0.112 |
| tumor protein p63 | Tp63 | 0.386579683 | 0.1647 |
| cyclin D2 | Ccnd2 | 0.382789318 | 0.138 |
| neural precursor cell expressed, developmentally down-regulated 8 | Nedd8 | 0.371847993 | 0.0367 |
| serum/glucocorticoid regulated kinase 1 | Sgk1 | 0.368400171 | 0.024575 |
| cytohesin 2 | Pscd2 | 0.362086201 | 0.177 |
| cell division cycle 6 homolog (S. cerevisiae) | Cdc6 | 0.358744395 | 0.1669 |
| tumor protein p53 | Tp53 | 0.355238168 | 0.133 |
| cyclin D3 | Ccnd3 | 0.351194078 | 0.24457 |
| keratin 8 | Krt8 | 0.340124816 | 0.111 |
| synaptosomal-associated protein, 25kDa | Snap25 | 0.324975031 | 0.233876 |
| GRB2-related adaptor protein 2 | Grap2 | 0.324632178 | 0.3427 |
| calponin 1, basic, smooth muscle | Cnn1 | 0.32172663 | 0.1142 |
| desmin | Des | 0.30968086 | 0.0277 |
| src kinase associated phosphoprotein 2 | Skap2 | 0.259589897 | 0.1376 |
| caspase 9, apoptosis-related cysteine peptidase | Casp9 | 0.250091394 | 0.1884 |
| keratin 19 | Krt19 | 0.237251551 | 0.14443 |
| RAS p21 protein activator 3 | Rasa3 | 0.228758969 | 0.11 |
| caspase 8, apoptosis-related cysteine peptidase | Casp8 | 0.223556444 | 0.337657 |
| protein phosphatase 3 (formerly 2B), regulatory subunit B | Ppp3r1 | 0.190428303 | 0.2757 |
| peroxiredoxin 3 | Prdx3 | 0.175924192 | 0.175725 |
| topoisomerase (DNA) I | Top1 | 0.175579744 | 0.11376 |
| dystrophin | Dmd | 0.158980099 | 0.0075 |
| actin, beta | Actb | 0.157210402 | 0.27327 |
| fizzy/cell division cycle 20 related 1 (Drosophila) | FZR1 | 0.154167813 | 0.0043287 |
| similar to telomeric repeat binding factor (NIMA-interacting) 1 | Terf1 | 0.118622818 | 0.176 |
| apoptosis-inducing factor, mitochondrion-associated, 1 | Aifm1 | 0.118527776 | 0.4242 |
| caspase 4, apoptosis-related cysteine peptidase | Casp4 | 0.100814649 | 0.14376 |
| v-akt murine thymoma viral oncogene homolog 1 | Akt1 | 0.099009901 | 0.677 |
| cyclin-dependent kinase 6 | Cdk6 | 0.086777392 | 0.04557 |
| cyclin-dependent kinase inhibitor 1C (p57, Kip2) | Cdkn1c | 0.086719224 | 0.147 |
| heat shock protein 90kDa alpha (cytosolic), class A member 1 | Hsp90aa1 | 0.078 | 0.2337 |
| caspase 12 (gene/pseudogene) | Casp12 | 0.073678264 | 0.10457 |
| septin 4 | Sept4 | 0.072992701 | 0.007657 |
| actin, alpha 1, skeletal muscle | Acta1 | 0.062626048 | 0.1172 |
| adaptor-related protein complex 1, beta 1 subunit | Ap1b1 | 0.056944757 | 0.257 |
| keratin 7 | Krt7 | 0.043100189 | 0.337 |
| microtubule-associated protein 2 | Map2 | 0.038731584 | 0.06687 |
| HSPA (heat shock 70kDa) binding protein, cytoplasmic cochaperone 1 | Hspbp1 | 0.019145394 | 0.1757 |
| nuclear transport factor 2 | Nutf2 | 0.017630198 | 0.37376 |
| vinculin | Vcl | 0.012884615 | 0.27575 |
| syntaxin 1A (brain) | Stx1a | 0.011863918 | 0.1765725 |
| S100 calcium binding protein A1 | S100a1 | 0.006232687 | 0.2427 |
| cathepsin D | Ctsd | 0.000356706 | 0.07575 |
