# Supplementary material for: GIT2 Acts as a Potential Keystone Protein in Functional Hypothalamic Networks Associated with Age-Related Phenotypic Changes in Rats
Source: PLoS One. 2012 May 14;7(5):e36975. doi: 10.1371/journal.pone.0036975 (PMC3351446; doi:10.1371/journal.pone.0036975)
Supplement: Table S2 — Protein expression alterations in old compared to young rat hypothalamus. Panorama® Cell Signaling Array platforms were employed to assess the relative expression ratio of individual proteins for old (O) versus young (Y) rats (O/Y). Expression ratios were calculated from triplicate experiments and the mean and standard error of the mean (SEM) for each protein demonstrating an O/Y ratio using the following criteria: ratio>1.5 and ratio<0.5. (DOC) [file pone.0036975.s006.doc]

**Table S2. Protein expression alterations in old compared to young rat hypothalamus.** Panorama® Cell Signaling Array platforms were employed to assess the relative expression ratio of individual proteins for old (O) versus young (Y) rats (O/Y). Expression ratios were calculated from triplicate experiments and the mean and standard error of the mean (SEM) for each protein demonstrating an O/Y ratio using the following criteria: ratio> 1.5 and ratio<0.5.

| **Protein** | **Symbol** | **Mean ratio (O/Y)** | **SEM** |
| --- | --- | --- | --- |
| phosphatase and tensin homolog; phosphatase and tensin homolog pseudogene 1 | Pten | 13.98511835 | 0.1 |
| p300/CBP-associated factor | Pcaf | 5.879677839 | 0.0278846 |
| B-cell CLL/lymphoma 10 | Bcl10 | 5.512415874 | 0.0172231 |
| protein kinase C, beta | Prkcb1 | 5.307805838 | 0.0167822 |
| PRKC, apoptosis, WT1, regulator | Pawr | 5.146973012 | 0.0229397 |
| cyclin-dependent kinase 5, regulatory subunit 1 (p35) | Cdk5r1 | 4.850077103 | 0.027424 |
| GRB2-related adaptor protein 2 | Grap2 | 4.639694656 | 0.075768 |
| src kinase associated phosphoprotein 2 | Skap2 | 4.172198708 | 0.0432 |
| c-myc binding protein | Mycbp | 3.943424298 | 0.034327 |
| cyclin D3 | Ccnd3 | 3.935568623 | 0.0375732 |
| nuclear transport factor 2 | Nutf2 | 3.882339344 | 0.087673 |
| calmodulin regulated spectrin-associated protein 1-like 1 | Camsap1l1 | 3.874018585 | 0.038383 |
| catenin (cadherin-associated protein), alpha 1, 102kDa | Ctnna1 | 3.59807096 | 0.075 |
| gap junction protein, alpha 1, 43kDa | Gja1 | 3.451643071 | 0.075768 |
| jumonji domain containing 6 | Jmjd6 | 3.437117059 | 0.0432 |
| protein kinase C, alpha | Prkca | 3.394471073 | 0.1742 |
| protein phosphatase 3 (formerly 2B), regulatory subunit B, alpha isoform | Ppp3r1 | 3.368665121 | 0.075 |
| cytohesin 2 | Pscd2 | 3.169937958 | 0.1375 |
| activating transcription factor 2 | Atf2 | 3.13731189 | 0.2765 |
| mitogen-activated protein kinase 8 | Mapk8 | 3.130365782 | 0.175 |
| E2F transcription factor 1 | E2f1 | 3.08573666 | 0.247 |
| nitric oxide synthase 3 (endothelial cell) | Nos3 | 3.076453046 | 0.17575 |
| caspase 7, apoptosis-related cysteine peptidase | Casp7 | 3.076101858 | 0.21 |
| estrogen receptor 1 | Esr1 | 3.033134384 | 0.2457 |
| caveolin 1, caveolae protein, 22kDa | Cav1 | 2.967396594 | 0.1757 |
| DNA-damage-inducible transcript 3 | Ddit3 | 2.864537815 | 0.175 |
| caspase 6, apoptosis-related cysteine peptidase | Casp6 | 2.851565195 | 0.0175 |
| protein kinase C, gamma | Prkcg | 2.840681149 | 0.0106636 |
| caspase 10, apoptosis-related cysteine peptidase | Casp10 | 2.81220753 | 0.0099304 |
| nerve growth factor receptor (TNFRSF16) associated protein 1 | Ngfrap1 | 2.766480626 | 0.0093309 |
| caspase 4, apoptosis-related cysteine peptidase | Casp4 | 2.762650314 | 0.007825 |
| mitogen-activated protein kinase 3 | Mapk3 | 2.749065647 | 0.0167822 |
| leucine carboxyl methyltransferase 2 | Lcmt2 | 2.740448926 | 0.0229397 |
| histone acetyltransferase 1 | Hat1 | 2.737732253 | 0.027424 |
| synaptosomal-associated protein, 25kDa | Snap25 | 2.716961954 | 0.075768 |
| neurofilament, heavy polypeptide | Nefh | 2.678499135 | 0.0432 |
| Dystrophin | Dmd | 2.661997785 | 0.1742 |
| synuclein, alpha (non A4 component of amyloid precursor) | Snca | 2.638612342 | 0.075 |
| SMAD family member 4 | Smad4 | 2.630973134 | 0.034327 |
| tumor protein p53 | Tp53 | 2.625547573 | 0.0375732 |
| histone deacetylase 4 | Hdac4 | 2.616791783 | 0.087673 |
| chondroitin sulfate N-acetylgalactosaminyltransferase 1 | Csgalnact1 | 2.61364975 | 0.038383 |
| myosin VA (heavy chain 12, myoxin) | Myo5a | 2.575497337 | 0.175 |
| Nicastrin | Ncstn | 2.56841351 | 0.075768 |
| glutamate receptor, ionotropic, N-methyl D-aspartate 2ª | Grin2a | 2.543455735 | 0.0432 |
| fizzy/cell division cycle 20 related 1 (Drosophila) | Fzr1 | 2.527327641 | 0.04273 |
| dual specificity phosphatase 1 | Dusp1 | 2.490983089 | 0.2727 |
| myosin, heavy chain 9, non-muscle | Myh9 | 2.488891018 | 0.1 |
| mitogen-activated protein kinase 1 | Mapk1 | 2.448844 | 0.187673 |
| phospholipase C, gamma 1 | Plcg1 | 2.438076416 | 0.038383 |
| caspase 12 (gene/pseudogene) | Casp12 | 2.434708875 | 0.275 |
| phospholipase A2, group IVA (cytosolic, calcium-dependent) | Pla2g4a | 2.412584103 | 0.075768 |
| catenin (cadherin-associated protein), delta 1 | Ctnnd1 | 2.38672103 | 0.0106636 |
| keratin 4 | Krt4 | 2.371991471 | 0.0099304 |
| caspase 8, apoptosis-related cysteine peptidase | Casp8 | 2.350034913 | 0.0093309 |
| caspase 3, apoptosis-related cysteine peptidase | Casp3 | 2.346262088 | 0.007825 |
| protein phosphatase 1, catalytic subunit, alpha isoform | Ppp1ca | 2.326710387 | 0.0167822 |
| cyclin B1 | Ccnb1 | 2.264555454 | 0.0229397 |
| BCL2-associated agonist of cell death | Bad | 2.20337005 | 0.274657 |
| cyclin-dependent kinase 4 | Cdk4 | 2.201548504 | 0.247 |
| histone deacetylase 5 | Hdac5 | 2.195323796 | 0.1476 |
| glutamate decarboxylase 1 (brain, 67kDa) | Gad1 | 2.181707597 | 0.1676 |
| mitogen-activated protein kinase 7 | Mapk7 | 2.162255122 | 0.007272 |
| glutamate-ammonia ligase (glutamine synthetase) | Glul | 2.154532176 | 0.172732 |
| syntrophin, alpha 1 (dystrophin-associated protein A1, 59kDa, acidic component) | Snta1 | 2.152050345 | 0.1477 |
| jun oncogene | Jun | 2.11469105 | 0.245627 |
| histone deacetylase 2 | Hdac2 | 2.112501668 | 0.3425 |
| cyclin-dependent kinase inhibitor 2A (melanoma, p16, inhibits CDK4) | Cdkn2a | 2.106704476 | 0.02737 |
| gap junction protein, beta 1, 32kDa | Gjb1 | 2.10052813 | 0.172 |
| keratin 13 | Krt13 | 2.094819794 | 0.15125 |
| cyclin A1 | Ccna1 | 2.094312434 | 0.30415 |
| stathmin 1 | Stmn1 | 2.075060793 | 0.1925 |
| catenin, beta like 1 | Ctnnbl1 | 2.037393426 | 0.2717 |
| Mdm2 p53 binding protein homolog | Mdm2 | 2.024968789 | 0.193325 |
| cell division cycle 25 homolog C (S. pombe) | Cdc25c | 1.966125966 | 0.231 |
| amyloid beta (A4) precursor protein | App | 1.940481847 | 0.27027 |
| myeloid cell leukemia sequence 1 (BCL2-related) | Mcl1 | 1.936618115 | 0.19327 |
| PTK2 protein tyrosine kinase 2 | Ptk2 | 1.930430482 | 0.1925 |
| protein kinase D | Pkd | 1.928180981 | 0.01925 |
| microtubule-associated protein tau | Mapt | 1.925039872 | 0.0117299 |
| focal adhesion kinase | Fak | 1.914526264 | 0.0109235 |
| c-abl oncogene 1, receptor tyrosine kinase | Abl1 | 1.904769359 | 0.010264 |
| calcium/calmodulin-dependent protein kinase IV | Camk4 | 1.901714835 | 0.0086075 |
| mitogen-activated protein kinase-activated protein kinase 2 | Mapkapk2 | 1.898695908 | 0.0184605 |
| CUGBP, Elav-like family member 1 | Cugbp1 | 1.884846391 | 0.0252337 |
| nuclear factor of kappa light polypeptide gene enhancer in B-cells inhibitor, alpha | Nfkbia | 1.838566438 | 0.0301664 |
| synaptotagmin I | Syt1 | 1.837193782 | 0.0833448 |
| cyclin-dependent kinase 7 | Cdk7 | 1.818057456 | 0.04752 |
| nitric oxide synthase 1 (neuronal) | Nos1 | 1.815217219 | 0.19162 |
| tyrosine hydroxylase | Th | 1.789303355 | 0.0825 |
| PTK2B protein tyrosine kinase 2 beta | Ptk2b | 1.780081335 | 0.0377597 |
| serum/glucocorticoid regulated kinase 1 | Sgk1 | 1.76959394 | 0.166 |
| Calreticulin | Calr | 1.764325734 | 0.0462885 |
| cofilin 1 (non-muscle) | Cfl1 | 1.761244851 | 0.0285904 |
| parvin, alpha | Parva | 1.752889768 | 0.0278585 |
| RAN, member RAS oncogene family | Ran | 1.752232614 | 0.0380799 |
| fibronectin 1 | Fn1 | 1.745797884 | 0.0455238 |
| topoisomerase (DNA) I | Top1 | 1.735210501 | 0.1257749 |
| cyclin D2 | Ccnd2 | 1.69895091 | 0.071712 |
| keratin 18 | Krt18 | 1.695857787 | 0.0569828 |
| cadherin 1, type 1, E-cadherin (epithelial) | Cdh1 | 1.662849025 | 0.0623715 |
| coatomer protein complex, subunit beta 1 | Copb1 | 1.660966781 | 0.1455372 |
| cell division cycle 6 homolog (S. cerevisiae) | Cdc6 | 1.628277486 | 0.231 |
| tropomyosin 1 (alpha) | Tpm1 | 1.620934435 | 0.27027 |
| death-domain associated protein | Daxx | 1.612073802 | 0.19327 |
| neural precursor cell expressed, developmentally down-regulated 8 | Nedd8 | 1.611566704 | 0.1925 |
| nitric oxide synthase 2, inducible | Nos2 | 1.611031475 | 0.01925 |
| peroxiredoxin 3 | Prdx3 | 1.601877862 | 0.0117299 |
| internexin neuronal intermediate filament protein, alpha | Ina | 1.587682136 | 0.0109235 |
| CHK1 checkpoint homolog (S. pombe) | Chek1 | 1.5848019 | 0.010264 |
| cyclin-dependent kinase inhibitor 2D (p19, inhibits CDK4) | Cdkn2d | 1.575988208 | 0.0086075 |
| cell division cycle 27 homolog (S. cerevisiae) | Cdc27 | 1.56819019 | 0.1867 |
| microtubule-associated protein 1B | Map1b | 1.558238163 | 0.176 |
| heterogeneous nuclear ribonucleoprotein H1 (H) | Hnrnph1 | 1.549651023 | 0.1727 |
| clathrin, light chain (Lca) | Clta | 1.531115642 | 0.1727 |
| keratin 19 | Krt19 | 1.521775645 | 0.3737 |
| diablo homolog (Drosophila) | Diablo | 1.517324974 | 0.1427 |
| epidermal growth factor receptor | Egfr | 1.506592684 | 0.1427 |
| cyclin-dependent kinase inhibitor 2A | CDKN2A | 0.483928939 | 0.021508 |
| apoptosis-inducing factor, mitochondrion-associated, 1 | Aifm1 | 0.48121462 | 0.0213873 |
| septin 4 | Sept4 | 0.477115935 | 0.0212052 |
| cytohesin 3 | Pscd3 | 0.462581195 | 0.0205592 |
| S100 calcium binding protein A1 | S100A1 | 0.460160922 | 0.0204516 |
| microtubule-associated protein tau | MAPT | 0.418269231 | 0.0278846 |
| similar to telomeric repeat binding factor (NIMA-interacting) 1 | Terf1 | 0.417463207 | 0.0172231 |
| tryptophan hydroxylase 1 | TPH1 | 0.412241604 | 0.0167822 |
| cyclin-dependent kinase inhibitor 1C (p57, Kip2) | Cdkn1c | 0.405557876 | 0.0229397 |
| adaptor-related protein complex 1, beta 1 subunit | Ap1b1 | 0.401404703 | 0.0213234 |
| cyclin A1 | CCNA1 | 0.399598304 | 0.0228342 |
| mitogen-activated protein kinase 8 | MAPK8 | 0.398349286 | 0.0227628 |
| caspase 9, apoptosis-related cysteine peptidase | Casp9 | 0.394507049 | 0.0225433 |
| spectrin, alpha, non-erythrocytic 1 (alpha-fodrin) | Sptan1 | 0.393756129 | 0.0225004 |
| heat shock protein 90kDa alpha (cytosolic), class A member 2 | Hsp90aa1 | 0.386746247 | 0.0220998 |
| keratin 7 | Krt7 | 0.37378571 | 0.0213592 |
| RAS p21 protein activator 3 | RASA3 | 0.361998669 | 0.0206856 |
| junction plakoglobin | Jup | 0.356574363 | 0.0203757 |
| Vinculin | Vcl | 0.316732543 | 0.018099 |
| cathepsin D | Ctsd | 0.31432394 | 0.0179614 |
| cyclin D1 | CCND1 | 0.293689362 | 0.0235567 |
| cell division cycle 7 homolog (S. cerevisiae) | Cdc7 | 0.277455929 | 0.0231747 |
| dopa decarboxylase (aromatic L-amino acid decarboxylase) | Ddc | 0.261621563 | 0.0209297 |
| microtubule-associated protein 2 | Map2 | 0.234757657 | 0.0187806 |
| S100 calcium binding protein B | S100b | 0.178431523 | 0.0142745 |
| syntaxin 1A (brain) | Stx1a | 0.133294646 | 0.0106636 |
| HSPA (heat shock 70kDa) binding protein, cytoplasmic cochaperone 1 | Hspbp1 | 0.124130308 | 0.0172231 |
| v-akt murine thymoma viral oncogene homolog 1 | Akt1 | 0.116636478 | 0.0167822 |
| synuclein, beta | Sncb | 0.097812651 | 0.0229397 |
