# Supplementary material for: GIT2 Acts as a Potential Keystone Protein in Functional Hypothalamic Networks Associated with Age-Related Phenotypic Changes in Rats
Source: PLoS One. 2012 May 14;7(5):e36975. doi: 10.1371/journal.pone.0036975 (PMC3351446; doi:10.1371/journal.pone.0036975)
Supplement: Table S3 — Proteins significantly regulated in both middle- and old-aged rat hypothalami compared to young rats. Panorama® Cell Signaling Array platforms were employed to assess the relative expression ratio of individual proteins for middle-aged/old (M or O) versus young (Y) rats (M/Y or O/Y). Expression ratios were calculated from triplicate experiments and the mean and standard error of the mean (SEM) for each protein demonstrating an O/Y ratio using the following criteria: ratio>1.5 and ratio<0.5. (DOC) [file pone.0036975.s007.doc]

**Table S3. Proteins significantly regulated in both middle- and old-aged rat hypothalami compared to young rats.** Panorama® Cell Signaling Array platforms were employed to assess the relative expression ratio of individual proteins for middle-aged/old (M or O) versus young (Y) rats (M/Y or O/Y). Expression ratios were calculated from triplicate experiments and the mean and standard error of the mean (SEM) for each protein demonstrating an O/Y ratio using the following criteria: ratio> 1.5 and ratio<0.5.

| **Common proteins in M and O vs. Y** | **Mean ratio M/Y** | **Mean ratio O/Y** |
| --- | --- | --- |
| Pawr | 1.65992547 | 5.146973 |
| Camsap1l1 | 1.58882217 | 3.874019 |
| Ctnna1 | 1.5401018 | 3.598071 |
| Mycbp | 2.24493392 | 3.943424 |
| Prkca | 1.85456188 | 3.394471 |
| Gja1 | 1.9473516 | 3.451643 |
| Casp7 | 1.76881686 | 3.076102 |
| Casp10 | 1.56032216 | 2.812208 |
| Ngfrap1 | 1.52017565 | 2.766481 |
| Casp6 | 1.63920308 | 2.851565 |
| Lcmt2 | 1.65231689 | 2.740449 |
| Ddit3 | 1.77706358 | 2.864538 |
| Hdac4 | 1.59206719 | 2.616792 |
| Nefh | 1.80401235 | 2.678499 |
| E2f1 | 2.22945131 | 3.085737 |
| Myh9 | 1.64432953 | 2.488891 |
| Myo5a | 1.74038912 | 2.575497 |
| Pla2g4a | 1.61913084 | 2.412584 |
| Casp3 | 1.59150088 | 2.346262 |
| Cdkn2a | 1.52873708 | 2.106704 |
|  |  |  |
|  |  |  |
| Cdk4 | 1.84179235 | 2.201549 |
| Ccnb1 | 1.97418073 | 2.264555 |
| Ptk2b | 1.55244525 | 1.780081 |
| Ptk2 | 1.78585747 | 1.93043 |
| Cdk7 | 1.71217959 | 1.818057 |
|  |  |  |
| Cugbp1 | 29.35 | 1.884846 |
| Prkcg | 15.4241071 | 2.840681 |
| Krt13 | 8.85069444 | 2.09482 |
| Ran | 3.51578947 | 1.752233 |
| Hnrnph1 | 3.17792578 | 1.549651 |
| Hat1 | 4.25191257 | 2.737732 |
| Tpm1 | 2.45205479 | 1.620934 |
| Grin2a | 3.33988764 | 2.543456 |
| Daxx | 2.26348441 | 1.612074 |
| Map1b | 1.92442645 | 1.558238 |
| Ccna1 | 2.4170096 | 2.094312 |
| Ina | 1.80241584 | 1.587682 |
| Egfr | 1.622458 | 1.506593 |
|  |  |  |
| Cdc7 | 0.43326796 | 0.277456 |
|  |  |  |
| Ap1b1 | 0.05694476 | 0.401405 |
| Krt7 | 0.04310019 | 0.373786 |
| Cdkn1c | 0.08671922 | 0.405558 |
| Ctsd | 0.00035671 | 0.314324 |
| Hsp90aa1 | 0.078 | 0.386746 |
| Vcl | 0.01288462 | 0.316733 |
| Terf1 | 0.11862282 | 0.417463 |
| Map2 | 0.03873158 | 0.234758 |
| Casp9 | 0.25009139 | 0.394507 |
| Stx1a | 0.01186392 | 0.133295 |
| Hspbp1 | 0.01914539 | 0.12413 |
| Akt1 | 0.0990099 | 0.116636 |
| Sept4 | 0.0729927 | 0.477116 |
| Aifm1 | 0.11852778 | 0.481215 |
|  |  |  |
| Jup | 4.05555556 | 0.356574 |
| Sptan1 | 2.20792079 | 0.393756 |
| Sncb | 1.74179104 | 0.097813 |
|  |  |  |
| Cdk5r1 | 0.4499168 | 4.850077 |
| Grap2 | 0.32463218 | 4.639695 |
| Skap2 | 0.2595899 | 4.172199 |
| Nutf2 | 0.0176302 | 3.882339 |
| Ccnd3 | 0.35119408 | 3.935569 |
| Ppp3r1 | 0.1904283 | 3.368665 |
| Pscd2 | 0.3620862 | 3.169938 |
| Casp4 | 0.10081465 | 2.76265 |
| Dmd | 0.1589801 | 2.661998 |
| Snap25 | 0.32497503 | 2.716962 |
| Casp12 | 0.07367826 | 2.434709 |
| Tp53 | 0.35523817 | 2.625548 |
| Casp8 | 0.22355644 | 2.350035 |
| Mapk1 | 0.43316611 | 2.448844 |
| Plcg1 | 0.45183902 | 2.438076 |
| Top1 | 0.17557974 | 1.735211 |
| Mapkapk2 | 0.40658732 | 1.898696 |
| Prdx3 | 0.17592419 | 1.601878 |
| Sgk1 | 0.36840017 | 1.769594 |
| Nos1 | 0.47536677 | 1.815217 |
| Th | 0.45792286 | 1.789303 |
| Ccnd2 | 0.38278932 | 1.698951 |
| Krt19 | 0.23725155 | 1.521776 |
| Cdc6 | 0.35874439 | 1.628277 |
| Krt18 | 0.45008757 | 1.695858 |
| Nedd8 | 0.37184799 | 1.611567 |
| Nos2 | 0.39325654 | 1.611031 |
| Diablo | 0.47028998 | 1.517325 |
