# Supplementary material for: GIT2 Acts as a Potential Keystone Protein in Functional Hypothalamic Networks Associated with Age-Related Phenotypic Changes in Rats
Source: PLoS One. 2012 May 14;7(5):e36975. doi: 10.1371/journal.pone.0036975 (PMC3351446; doi:10.1371/journal.pone.0036975)
Supplement: Table S4 — KEGG signaling pathway enrichment for middle-aged versus young rat protein expression variation. KEGG signaling pathway enrichment was performed using WebGestalt with the protein set significantly altered in middle aged hypothalami compared to the young controls. KEGG signaling pathway text description, enrichment factor (R), probability of enrichment (P), and the resultant hybrid score (H: −log10(P)×R) is represented. (DOC) [file pone.0036975.s008.doc]

**Table S4. KEGG signaling pathway enrichment for middle-aged versus young rat protein expression variation**. KEGG signaling pathway enrichment was performed using WebGestalt with the protein set significantly altered in Middle aged hypothalami compared to the Young controls. KEGG signaling pathway text description, enrichment factor (R), probability of enrichment (P), and the resultant hybrid score (H: -log_10_(P) x R) is represented.

| **KEGG Pathway** | **R** | **P** | **H** |
| --- | --- | --- | --- |
| Glioma | 66.41 | 6.27E-18 | 1142.4335 |
| p53 signaling pathway | 60.21 | 1.78E-17 | 1008.4922 |
| Pancreatic cancer | 55.94 | 1.00E-15 | 839.1 |
| Cell cycle | 44.1 | 1.65E-19 | 828.30896 |
| Amyotrophic lateral sclerosis (ALS) | 53.76 | 3.00E-14 | 726.98996 |
| VEGF signaling pathway | 48.24 | 8.33E-14 | 630.94809 |
| Small cell lung cancer | 39.61 | 4.82E-13 | 487.87451 |
| ErbB signaling pathway | 33.82 | 4.33E-10 | 316.67398 |
| Prostate cancer | 32.03 | 5.99E-10 | 295.39902 |
| Focal adhesion | 22.81 | 1.08E-12 | 272.9576 |
| Gap junction | 30.28 | 1.06E-08 | 241.47374 |
| Colorectal cancer | 28.63 | 1.50E-08 | 223.99851 |
| MAPK signaling pathway | 18.12 | 1.69E-12 | 213.31069 |
| Leukocyte transendothelial migration | 25.09 | 4.05E-09 | 210.56895 |
| Natural killer cell mediated cytotoxicity | 24.62 | 3.98E-08 | 182.19088 |
| Progesterone-mediated oocyte maturation | 25.09 | 3.30E-07 | 162.62049 |
| Long-term depression | 27.67 | 2.02E-06 | 157.57093 |
| Long-term potentiation | 26.13 | 2.61E-06 | 145.89318 |
| Thyroid cancer | 36.42 | 0.0001 | 145.68 |
| Calcium signaling pathway | 17.82 | 6.72E-09 | 145.63628 |
| Arrhythmogenic right ventricular cardiomyopathy (ARVC) | 25.09 | 3.03E-06 | 138.4606 |
| Tight junction | 19.66 | 1.75E-07 | 132.84187 |
| Neurotrophin signaling pathway | 19.51 | 1.78E-07 | 131.68431 |
| Alzheimer's disease | 15.05 | 4.47E-09 | 125.66287 |
| Wnt signaling pathway | 18.17 | 2.80E-07 | 119.06514 |
| GnRH signaling pathway | 19.4 | 1.03E-05 | 96.750958 |
| Dorso-ventral axis formation | 34.21 | 0.002 | 92.331764 |
| Regulation of actin cytoskeleton | 12.14 | 3.23E-06 | 66.658281 |
| NOD-like receptor signaling pathway | 17.92 | 0.0008 | 55.496627 |
| Jak-STAT signaling pathway | 12.8 | 7.47E-05 | 52.821496 |
| Prion diseases | 20.34 | 0.005 | 46.80295 |
| Vascular smooth muscle contraction | 12.98 | 0.0004 | 44.105261 |
| Phosphatidylinositol signaling system | 14.85 | 0.0015 | 41.935045 |
| Chemokine signaling pathway | 10.75 | 0.0002 | 39.763928 |
| Arginine and proline metabolism | 13.94 | 0.0097 | 28.064402 |
| mTOR signaling pathway | 13.68 | 0.01 | 27.36 |
| Notch signaling pathway | 6.69 | 0.001 | 20.07 |
| Axon guidance | 8.49 | 0.006 | 18.863496 |
| Insulin signaling pathway | 8.36 | 0.0062 | 18.455605 |
| Type II diabetes mellitus | 5.23 | 0.001 | 15.69 |
| Huntington's disease | 6.35 | 0.0043 | 15.027475 |
| TGF-beta signaling pathway | 5.13 | 0.0039 | 12.357839 |
| Parkinson's disease | 6.27 | 0.0128 | 11.867793 |
| Lysosome | 6.38 | 0.0401 | 8.9119389 |
