# Supplementary material for: GIT2 Acts as a Potential Keystone Protein in Functional Hypothalamic Networks Associated with Age-Related Phenotypic Changes in Rats
Source: PLoS One. 2012 May 14;7(5):e36975. doi: 10.1371/journal.pone.0036975 (PMC3351446; doi:10.1371/journal.pone.0036975)
Supplement: Table S5 — KEGG signaling pathway enrichment for old versus young rat protein expression variation. KEGG signaling pathway enrichment was performed using WebGestalt with the protein set significantly altered in old-aged hypothalami compared to the young controls. KEGG signaling pathway text description, enrichment factor (R), probability of enrichment (P), and the resultant hybrid score (H: −log10(P)×R) is represented. (DOC) [file pone.0036975.s009.doc]

**Table S5. KEGG signaling pathway enrichment for old versus young rat protein expression variation**. KEGG signaling pathway enrichment was performed using WebGestalt with the protein set significantly altered in old-aged hypothalami compared to the young controls. KEGG signaling pathway text description, enrichment factor (R), probability of enrichment (P), and the resultant hybrid score (H: -log_10_(P) x R) is represented.

| **KEGG pathway** | **R** | **P** | **H** |
| --- | --- | --- | --- |
| Non-small cell lung cancer | 65.85 | 2.78E-19 | 1221.9097 |
| Bladder cancer | 69 | 2.90E-14 | 934.09454 |
| VEGF signaling pathway | 47.28 | 1.31E-17 | 798.21541 |
| Pathways in cancer | 25.48 | 1.14E-31 | 788.43006 |
| Chronic myeloid leukemia | 44.43 | 2.71E-17 | 736.07317 |
| Melanoma | 46.63 | 2.69E-16 | 726.04065 |
| p53 signaling pathway | 45.39 | 3.10E-16 | 703.93709 |
| Endometrial cancer | 51.58 | 2.26E-14 | 703.85509 |
| ErbB signaling pathway | 38.25 | 2.48E-15 | 558.66222 |
| Amyotrophic lateral sclerosis (ALS) | 40.52 | 2.49E-13 | 510.706 |
| Apoptosis | 34.38 | 7.49E-15 | 485.63532 |
| Focal adhesion | 25.79 | 3.40E-19 | 476.30316 |
| MAPK signaling pathway | 19.96 | 2.47E-18 | 351.44177 |
| Long-term potentiation | 31.52 | 5.47E-10 | 291.93864 |
| Adherens junction | 30.67 | 6.59E-10 | 281.58478 |
| Alzheimer's disease | 18.15 | 4.15E-15 | 261.03243 |
| T cell receptor signaling pathway | 24.88 | 3.26E-11 | 260.91115 |
| Fc epsilon RI signaling pathway | 28.73 | 1.08E-09 | 257.60974 |
| Fc gamma R-mediated phagocytosis | 27.16 | 2.09E-08 | 208.58483 |
| Acute myeloid leukemia | 28.85 | 1.36E-07 | 198.0974 |
| GnRH signaling pathway | 23.4 | 5.29E-09 | 193.67114 |
| B cell receptor signaling pathway | 24.51 | 3.51E-08 | 182.71452 |
| Long-term depression | 25.03 | 2.96E-07 | 163.41357 |
| Calcium signaling pathway | 16.42 | 2.60E-10 | 157.38614 |
| Toll-like receptor signaling pathway | 20.9 | 1.04E-07 | 145.944 |
| Wnt signaling pathway | 17.61 | 6.17E-09 | 144.57308 |
| Gap junction | 19.56 | 1.20E-06 | 115.81121 |
| Viral myocarditis | 16.37 | 3.34E-06 | 89.64627 |
| Tight junction | 14.82 | 9.36E-07 | 89.345692 |
| Regulation of actin cytoskeleton | 11.77 | 1.70E-07 | 79.677616 |
| Prion diseases | 23 | 0.0004 | 78.15262 |
| Axon guidance | 12.8 | 1.30E-05 | 62.541525 |
| Insulin signaling pathway | 12.61 | 1.39E-05 | 61.246583 |
| TGF-beta signaling pathway | 25.13 | 0.0039 | 60.536546 |
| Phosphatidylinositol signaling system | 14.93 | 0.0003 | 52.59658 |
| Notch signaling pathway | 16.69 | 0.001 | 50.07 |
| Type II diabetes mellitus | 16.37 | 0.001 | 49.11 |
| Oocyte meiosis | 13.2 | 0.0004 | 44.852808 |
| mTOR signaling pathway | 15.47 | 0.0013 | 44.647296 |
| Alanine, aspartate and glutamate metabolism | 19.56 | 0.0054 | 44.354378 |
| Renal cell carcinoma | 15.46 | 0.0014 | 44.120861 |
| Chemokine signaling pathway | 9.73 | 5.88E-05 | 41.163959 |
| RIG-I-like receptor signaling pathway | 13.73 | 0.0017 | 38.025936 |
| Hypertrophic cardiomyopathy (HCM) | 13.28 | 0.0019 | 36.138152 |
| Tyrosine metabolism | 16.69 | 0.0072 | 35.761121 |
| Adipocytokine signaling pathway | 12.7 | 0.0021 | 34.007815 |
| Dilated cardiomyopathy | 12.41 | 0.0023 | 32.740958 |
| SNARE interactions in vesicular transport | 14.93 | 0.0088 | 30.688874 |
| Melanogenesis | 11.64 | 0.0026 | 30.08971 |
| Huntington's disease | 7.18 | 0.0003 | 25.294269 |
| Inositol phosphate metabolism | 9.95 | 0.0178 | 17.408321 |
| Parkinson's disease | 6.3 | 0.0045 | 14.784761 |
| Ubiquitin mediated proteolysis | 6.6 | 0.0114 | 12.824428 |
| Jak-STAT signaling pathway | 5.79 | 0.0161 | 10.382478 |
| Endocytosis | 3.9 | 0.0428 | 5.3373693 |
| Metabolic pathways | 2.45 | 0.0088 | 5.0360175 |
