# Supplementary material for: GIT2 Acts as a Potential Keystone Protein in Functional Hypothalamic Networks Associated with Age-Related Phenotypic Changes in Rats
Source: PLoS One. 2012 May 14;7(5):e36975. doi: 10.1371/journal.pone.0036975 (PMC3351446; doi:10.1371/journal.pone.0036975)
Supplement: Table S6 — GO-term enrichment for middle versus young rat protein expression variation. GO-term enrichment was performed using WebGestalt with the protein set significantly altered in middle aged hypothalami compared to the young controls. Official GO-term codes, the text description of the code as well as the enrichment factor (R), probability of enrichment (P), and the resultant hybrid score (H: −log10(P)×R) is represented. (DOC) [file pone.0036975.s010.doc]

**Table S6. GO-term enrichment for middle versus young rat protein expression variation**. GO-term enrichment was performed using WebGestalt with the protein set significantly altered in middle aged hypothalami compared to the young controls. Official GO-term codes, the text description of the code as well as the enrichment factor (R), probability of enrichment (P), and the resultant hybrid score (H: -log_10_(P) x R) is represented.

| **GO term code** | **GO term description** | **R** | **P** | **H** |
| --- | --- | --- | --- | --- |
| GO:0000307 | cyclin-dependent protein kinase holoenzyme complex | 87.34 | 4.83E-12 | 988.3441 |
| GO:0030235 | nitric-oxide synthase regulator activity | 122.79 | 1.42E-05 | 595.2505 |
| GO:0043423 | 3-phosphoinositide-dependent protein kinase binding | 109.15 | 0.0004 | 370.8852 |
| GO:0004517 | nitric-oxide synthase activity | 109.15 | 0.0004 | 370.8852 |
| GO:0004693 | cyclin-dependent protein kinase activity | 48.15 | 1.82E-06 | 276.3776 |
| GO:0043034 | costamere | 49.91 | 3.73E-06 | 270.926 |
| GO:0010243 | response to organic nitrogen | 19.69 | 5.25E-14 | 261.4801 |
| GO:0043008 | ATP-dependent protein binding | 81.86 | 0.0008 | 253.5131 |
| GO:0004861 | cyclin-dependent protein kinase inhibitor activity | 54.57 | 0.0001 | 218.28 |
| GO:0014704 | intercalated disc | 39.93 | 9.60E-06 | 200.3579 |
| GO:0051325 | interphase | 19.02 | 2.89E-10 | 181.4337 |
| GO:0016538 | cyclin-dependent protein kinase regulator activity | 38.52 | 2.27E-05 | 178.8859 |
| GO:0042641 | actomyosin | 27.22 | 3.72E-07 | 175.0098 |
| GO:0010035 | response to inorganic substance | 11.76 | 1.38E-11 | 127.715 |
| GO:0009628 | response to abiotic stimulus | 8.84 | 9.71E-15 | 123.873 |
| GO:0043627 | response to estrogen stimulus | 13 | 4.75E-10 | 121.203 |
| GO:0030018 | Z disc | 20.89 | 1.72E-06 | 120.4198 |
| GO:0005829 | cytosol | 5.86 | 3.18E-21 | 120.1158 |
| GO:0009719 | response to endogenous stimulus | 7.41 | 5.57E-16 | 113.0332 |
| GO:0010038 | response to metal ion | 11.83 | 2.86E-10 | 112.9012 |
| GO:0043292 | contractile fiber | 14.26 | 1.55E-08 | 111.3659 |
| GO:0044449 | contractile fiber part | 15.14 | 5.06E-08 | 110.4592 |
| GO:0005856 | cytoskeleton | 6.13 | 1.47E-18 | 109.3143 |
| GO:0043028 | caspase regulator activity | 30.7 | 0.0004 | 104.3168 |
| GO:0030017 | sarcomere | 15.56 | 2.31E-07 | 103.2622 |
| GO:0004197 | cysteine-type endopeptidase activity | 17.91 | 3.21E-06 | 98.38849 |
| GO:00390291 | protein serine/threonine kinase inhibitor activity | 28.89 | 0.0004 | 98.16649 |
| GO:0030016 | myofibril | 13.89 | 1.00E-07 | 97.23 |
| GO:0048545 | response to steroid hormone stimulus | 9.12 | 2.91E-11 | 96.0893 |
| GO:0006915 | apoptosis | 5.93 | 7.22E-17 | 95.71887 |
| GO:0012501 | programmed cell death | 5.87 | 7.82E-17 | 94.54688 |
| GO:0008219 | cell death | 5.68 | 1.52E-16 | 89.84713 |
| GO:0016265 | death | 5.65 | 1.55E-16 | 89.32463 |
| GO:0031674 | I band | 16.95 | 5.50E-06 | 89.15085 |
| GO:0005882 | intermediate filament | 12.71 | 1.96E-07 | 85.25543 |
| GO:0022403 | cell cycle phase | 8.74 | 2.10E-10 | 84.5838 |
| GO:0045111 | intermediate filament cytoskeleton | 12.36 | 2.34E-07 | 81.95649 |
| GO:0051015 | actin filament binding | 18.6 | 4.67E-05 | 80.55071 |
| GO:0048468 | cell development | 5.48 | 3.54E-15 | 79.19146 |
| GO:0043065 | positive regulation of apoptosis | 7.8 | 8.74E-11 | 78.45621 |
| GO:0043068 | positive regulation of programmed cell death | 7.76 | 9.08E-11 | 77.92525 |
| GO:0010942 | positive regulation of cell death | 7.69 | 1.02E-10 | 76.83386 |
| GO:0042981 | regulation of apoptosis | 5.85 | 1.45E-13 | 75.106 |
| GO:0007049 | cell cycle | 6.23 | 1.04E-12 | 74.65388 |
| GO:0043067 | regulation of programmed cell death | 5.78 | 1.90E-13 | 73.5288 |
| GO:0010941 | regulation of cell death | 5.75 | 1.94E-13 | 73.09514 |
| GO:0022402 | cell cycle process | 7.19 | 9.08E-11 | 72.20136 |
| GO:0010033 | response to organic substance | 5.22 | 3.16E-14 | 70.47163 |
| GO:0019887 | protein kinase regulator activity | 15.11 | 2.22E-05 | 70.31661 |
| GO:0044430 | cytoskeletal part | 5.76 | 2.20E-12 | 67.14765 |
| GO:0009725 | response to hormone stimulus | 6.32 | 8.74E-11 | 63.56965 |
| GO:0048518 | positive regulation of biological process | 3.9 | 1.20E-16 | 62.09119 |
| GO:0048522 | positive regulation of cellular process | 4.05 | 4.85E-16 | 62.02275 |
| GO:0006996 | organelle organization | 4.68 | 1.94E-13 | 59.49309 |
| GO:0016043 | cellular component organization | 3.62 | 7.22E-17 | 58.4321 |
| GO:0019207 | kinase regulator activity | 12.76 | 4.82E-05 | 55.08432 |
| GO:0048869 | cellular developmental process | 3.89 | 1.07E-14 | 54.3457 |
| GO:0019899 | enzyme binding | 6.41 | 4.59E-09 | 53.44778 |
| GO:0030154 | cell differentiation | 3.91 | 5.35E-14 | 51.89214 |
| GO:0032502 | developmental process | 3.15 | 7.22E-17 | 50.84561 |
| GO:0042127 | regulation of cell proliferation | 5.3 | 2.60E-10 | 50.80064 |
| GO:0005516 | calmodulin binding | 11.84 | 7.15E-05 | 49.08502 |
| GO:0048731 | system development | 3.37 | 3.55E-15 | 48.69573 |
| GO:0008234 | cysteine-type peptidase activity | 10.71 | 2.84E-05 | 48.69496 |
| GO:0019901 | protein kinase binding | 9.45 | 8.71E-06 | 47.81683 |
| GO:0043232 | intracellular non-membrane-bounded organelle | 3.34 | 2.16E-14 | 45.64292 |
| GO:0043228 | non-membrane-bounded organelle | 3.34 | 2.16E-14 | 45.64292 |
| GO:0007275 | multicellular organismal development | 3.14 | 9.71E-15 | 44.00013 |
| GO:0048856 | anatomical structure development | 3.2 | 2.18E-14 | 43.71694 |
| GO:0009653 | anatomical structure morphogenesis | 4.07 | 2.54E-11 | 43.12233 |
| GO:0005654 | nucleoplasm | 4.82 | 4.76E-09 | 40.11393 |
| GO:0048513 | organ development | 3.45 | 2.97E-12 | 39.76899 |
| GO:0019900 | kinase binding | 8.23 | 1.56E-05 | 39.56058 |
| GO:0042995 | cell projection | 4.68 | 7.74E-09 | 37.96069 |
| GO:0048523 | negative regulation of cellular process | 3.66 | 7.64E-11 | 37.02788 |
| GO:0006950 | response to stress | 3.58 | 6.16E-11 | 36.5533 |
| GO:0005737 | cytoplasm | 2.05 | 2.30E-18 | 36.15846 |
| GO:0003779 | actin binding | 7.41 | 1.42E-05 | 35.92154 |
| GO:0005515 | protein binding | 1.98 | 8.44E-18 | 33.80584 |
| GO:0043005 | neuron projection | 5.37 | 5.15E-07 | 33.7676 |
| GO:0048519 | negative regulation of biological process | 3.4 | 2.24E-10 | 32.80916 |
| GO:0045202 | synapse | 5.66 | 1.96E-06 | 32.30583 |
| GO:0047485 | protein N-terminus binding | 9.75 | 0.0008 | 30.19487 |
| GO:0043234 | protein complex | 2.96 | 1.18E-10 | 29.38723 |
| GO:0008092 | cytoskeletal protein binding | 5.63 | 8.71E-06 | 28.4877 |
| GO:0031981 | nuclear lumen | 3.64 | 2.87E-08 | 27.45331 |
| GO:0044444 | cytoplasmic part | 2.19 | 2.93E-13 | 27.44756 |
| GO:0000267 | cell fraction | 3.75 | 6.80E-08 | 26.87809 |
| GO:0044424 | intracellular part | 1.64 | 1.35E-16 | 26.02625 |
| GO:0004672 | protein kinase activity | 4.65 | 3.21E-06 | 25.54475 |
| GO:0005622 | intracellular | 1.56 | 4.52E-17 | 25.49798 |
| GO:0044446 | intracellular organelle part | 2.43 | 3.79E-11 | 25.32391 |
| GO:0044422 | organelle part | 2.41 | 4.89E-11 | 24.84877 |
| GO:0043229 | intracellular organelle | 1.76 | 6.44E-14 | 23.21636 |
| GO:0043226 | organelle | 1.76 | 7.20E-14 | 23.13109 |
| GO:0005634 | nucleus | 2.34 | 2.72E-10 | 22.38311 |
| GO:0044428 | nuclear part | 3.09 | 1.84E-07 | 20.81171 |
| GO:0016301 | kinase activity | 3.94 | 5.45E-06 | 20.7386 |
| GO:0046983 | protein dimerization activity | 4.41 | 2.29E-05 | 20.46313 |
| GO:0030234 | enzyme regulator activity | 4.18 | 1.45E-05 | 20.22548 |
| GO:0016773 | phosphotransferase activity, alcohol group as acceptor | 3.93 | 1.45E-05 | 19.01582 |
| GO:0070013 | intracellular organelle lumen | 2.97 | 1.20E-06 | 17.58483 |
| GO:0031974 | membrane-enclosed lumen | 2.91 | 9.70E-07 | 17.49849 |
| GO:0016772 | transferase activity, transferring phosphorus-containing groups | 3.46 | 1.59E-05 | 16.60317 |
| GO:0043233 | organelle lumen | 2.87 | 2.08E-06 | 16.30716 |
| GO:0032991 | macromolecular complex | 2.28 | 1.40E-07 | 15.62683 |
| GO:0004674 | protein serine/threonine kinase activity | 4.21 | 0.0003 | 14.83132 |
| GO:0030554 | adenyl nucleotide binding | 2.88 | 1.56E-05 | 13.8438 |
| GO:0042802 | identical protein binding | 3.66 | 0.0002 | 13.53823 |
| GO:0001883 | purine nucleoside binding | 2.83 | 1.87E-05 | 13.38069 |
| GO:0001882 | nucleoside binding | 2.81 | 2.05E-05 | 13.17397 |
| GO:0043231 | intracellular membrane-bounded organelle | 1.7 | 2.05E-08 | 13.07002 |
| GO:0043227 | membrane-bounded organelle | 1.7 | 2.14E-08 | 13.0383 |
| GO:0017076 | purine nucleotide binding | 2.66 | 1.49E-05 | 12.83932 |
| GO:0005488 | binding | 1.36 | 1.44E-09 | 12.02463 |
| GO:0005524 | ATP binding | 2.71 | 0.0001 | 10.84 |
| GO:0032559 | adenyl ribonucleotide binding | 2.66 | 0.0001 | 10.64 |
| GO:0032555 | purine ribonucleotide binding | 2.48 | 0.0001 | 9.92 |
| GO:0032553 | ribonucleotide binding | 2.48 | 0.0001 | 9.92 |
| GO:0000166 | nucleotide binding | 2.3 | 7.91E-05 | 9.434194 |
