# Supplementary material for: GIT2 Acts as a Potential Keystone Protein in Functional Hypothalamic Networks Associated with Age-Related Phenotypic Changes in Rats
Source: PLoS One. 2012 May 14;7(5):e36975. doi: 10.1371/journal.pone.0036975 (PMC3351446; doi:10.1371/journal.pone.0036975)
Supplement: Table S7 — GO-term enrichment for old versus young protein expression variation. GO-term enrichment was performed using WebGestalt with the protein set significantly altered in middle aged hypothalami compared to the young controls. Official GO-term codes, the text description of the code as well as the enrichment factor (R ), probability of enrichment (P), and the resultant hybrid score (H: −log10(P)×R) is represented. (DOC) [file pone.0036975.s011.doc]

**Table S7. GO-term enrichment for old versus young protein expression variation**. GO-term enrichment was performed using WebGestalt with the protein set significantly altered in middle aged hypothalami compared to the young controls. Official GO-term codes, the text description of the code as well as the enrichment factor (R ), probability of enrichment (P), and the resultant hybrid score (H: -log_10_(P) x R) is represented.

| **GO term code** | **GO term description** | **R** | **P** | **H** |
| --- | --- | --- | --- | --- |
| GO:0004517 | nitric-oxide synthase activity | 121.18 | 1.14E-05 | 599.0043 |
| GO:0034617 | tetrahydrobiopterin binding | 121.18 | 0.0004 | 411.7624 |
| GO:0030235 | nitric-oxide synthase regulator activity | 90.89 | 3.24E-05 | 408.0466 |
| GO:0042153 | RPTP-like protein binding | 72.71 | 5.64E-05 | 308.9245 |
| GO:0000307 | cyclin-dependent protein kinase holoenzyme complex | 45.92 | 2.62E-07 | 302.2316 |
| GO:0048156 | tau protein binding | 51.93 | 0.0002 | 192.0875 |
| GO:0005829 | cytosol | 6.02 | 9.47E-31 | 180.7424 |
| GO:0043627 | response to estrogen stimulus | 13.35 | 5.85E-14 | 176.6585 |
| GO:0010243 | response to organic nitrogen | 14.61 | 2.70E-12 | 169.0178 |
| GO:0006915 | apoptosis | 6.24 | 3.58E-25 | 152.5438 |
| GO:0012501 | programmed cell death | 6.17 | 3.58E-25 | 150.8325 |
| GO:0043204 | perikaryon | 20.04 | 3.46E-08 | 149.5169 |
| GO:0008219 | cell death | 5.97 | 1.00E-24 | 143.28 |
| GO:0016265 | death | 5.94 | 1.00E-24 | 142.56 |
| GO:0004861 | cyclin-dependent protein kinase inhibitor activity | 40.39 | 0.0003 | 142.2891 |
| GO:0042981 | regulation of apoptosis | 6.43 | 1.09E-21 | 134.7893 |
| GO:0043067 | regulation of programmed cell death | 6.35 | 1.55E-21 | 132.1414 |
| GO:0010941 | regulation of cell death | 6.33 | 1.62E-21 | 131.6038 |
| GO:0004707 | MAP kinase activity | 30.3 | 7.04E-05 | 125.8185 |
| GO:0043065 | positive regulation of apoptosis | 8.22 | 8.69E-16 | 123.8013 |
| GO:0016504 | peptidase activator activity | 36.35 | 0.0004 | 123.5151 |
| GO:0043068 | positive regulation of programmed cell death | 8.18 | 9.54E-16 | 122.8673 |
| GO:0010942 | positive regulation of cell death | 8.11 | 1.13E-15 | 121.2195 |
| GO:0051726 | regulation of cell cycle | 9.27 | 2.87E-13 | 116.2654 |
| GO:0004693 | cyclin-dependent protein kinase activity | 28.51 | 8.76E-05 | 115.6792 |
| GO:0010033 | response to organic substance | 5.52 | 2.22E-21 | 114.0081 |
| GO:0048545 | response to steroid hormone stimulus | 8.65 | 7.28E-14 | 113.6426 |
| GO:0005913 | cell-cell adherens junction | 18.89 | 3.34E-06 | 103.4464 |
| GO:0048518 | positive regulation of biological process | 4.04 | 1.00E-24 | 96.96 |
| GO:0009719 | response to endogenous stimulus | 6.26 | 5.15E-16 | 95.70409 |
| GO:0007049 | cell cycle | 6.05 | 4.43E-16 | 92.88926 |
| GO:0009628 | response to abiotic stimulus | 7.11 | 9.01E-14 | 92.75191 |
| GO:0010181 | FMN binding | 27.96 | 0.0005 | 92.2968 |
| GO:0048522 | positive regulation of cellular process | 4.14 | 7.54E-23 | 91.58768 |
| GO:0019899 | enzyme binding | 6.75 | 2.34E-13 | 85.25779 |
| GO:0016043 | cellular component organization | 3.46 | 4.20E-21 | 70.50356 |
| GO:0065009 | regulation of molecular function | 4.91 | 2.38E-14 | 66.89101 |
| GO:0004197 | cysteine-type endopeptidase activity | 13.25 | 1.75E-05 | 63.02975 |
| GO:0030424 | axon | 8.04 | 1.50E-08 | 62.90423 |
| GO:0019903 | protein phosphatase binding | 15.54 | 0.0001 | 62.16 |
| GO:0022607 | cellular component assembly | 4.75 | 1.33E-13 | 61.1617 |
| GO:0048468 | cell development | 4.54 | 3.48E-14 | 61.10123 |
| GO:0042127 | regulation of cell proliferation | 5.08 | 1.02E-12 | 60.91631 |
| GO:0005654 | nucleoplasm | 4.99 | 6.36E-13 | 60.86075 |
| GO:0005856 | cytoskeleton | 4.51 | 3.54E-14 | 60.664 |
| GO:0048306 | calcium-dependent protein binding | 17.31 | 0.0004 | 58.81834 |
| GO:0005912 | adherens junction | 9.42 | 5.82E-07 | 58.73443 |
| GO:0048731 | system development | 3.25 | 9.07E-19 | 58.63778 |
| GO:0032502 | developmental process | 2.99 | 2.81E-20 | 58.45837 |
| GO:0042995 | cell projection | 4.85 | 1.15E-12 | 57.90562 |
| GO:0030154 | cell differentiation | 3.68 | 2.69E-16 | 57.29851 |
| GO:0043005 | neuron projection | 5.8 | 1.81E-10 | 56.50546 |
| GO:0044085 | cellular component biogenesis | 4.43 | 3.84E-13 | 55.00141 |
| GO:0048869 | cellular developmental process | 3.55 | 4.43E-16 | 54.50527 |
| GO:0007275 | multicellular organismal development | 3.04 | 2.07E-18 | 53.75945 |
| GO:0070161 | anchoring junction | 8.89 | 9.80E-07 | 53.418 |
| GO:0006996 | organelle organization | 4.08 | 1.38E-13 | 52.46929 |
| GO:0048856 | anatomical structure development | 3.09 | 1.20E-17 | 52.28533 |
| GO:0019902 | phosphatase binding | 14.09 | 0.0002 | 52.11849 |
| GO:0005625 | soluble fraction | 6.39 | 8.97E-09 | 51.42166 |
| GO:0031981 | nuclear lumen | 4.02 | 2.06E-13 | 50.99825 |
| GO:0051015 | actin filament binding | 13.77 | 0.0002 | 50.93482 |
| GO:0043687 | post-translational protein modification | 3.85 | 8.02E-14 | 50.41893 |
| GO:0051239 | regulation of multicellular organismal process | 4.11 | 1.33E-12 | 48.81097 |
| GO:0019900 | kinase binding | 8.12 | 9.88E-07 | 48.76257 |
| GO:0019901 | protein kinase binding | 8.54 | 1.96E-06 | 48.74413 |
| GO:0048471 | perinuclear region of cytoplasm | 6.8 | 1.16E-07 | 47.16169 |
| GO:0048523 | negative regulation of cellular process | 3.57 | 9.64E-14 | 46.46684 |
| GO:0009653 | anatomical structure morphogenesis | 3.75 | 8.77E-13 | 45.21375 |
| GO:0005515 | protein binding | 1.96 | 2.00E-23 | 44.48998 |
| GO:0048513 | organ development | 3.25 | 3.63E-14 | 43.6803 |
| GO:0047485 | protein N-terminus binding | 10.1 | 5.84E-05 | 42.75923 |
| GO:0006950 | response to stress | 3.39 | 5.29E-13 | 41.61749 |
| GO:0005634 | nucleus | 2.52 | 5.01E-17 | 41.07641 |
| GO:0008092 | cytoskeletal protein binding | 5.77 | 8.40E-08 | 40.82691 |
| GO:0031974 | membrane-enclosed lumen | 3.42 | 1.32E-12 | 40.62764 |
| GO:0010843 | promoter binding | 11.88 | 0.0004 | 40.36753 |
| GO:0048519 | negative regulation of biological process | 3.29 | 7.40E-13 | 39.91023 |
| GO:0000149 | SNARE binding | 13.1 | 0.0009 | 39.89942 |
| GO:0043233 | organelle lumen | 3.42 | 2.72E-12 | 39.55377 |
| GO:0005737 | cytoplasm | 1.97 | 1.05E-20 | 39.35826 |
| GO:0044212 | DNA regulatory region binding | 11.43 | 0.0004 | 38.83845 |
| GO:0044446 | intracellular organelle part | 2.49 | 3.37E-16 | 38.5262 |
| GO:0044422 | organelle part | 2.47 | 4.93E-16 | 37.80867 |
| GO:0044428 | nuclear part | 3.33 | 1.26E-11 | 36.29577 |
| GO:0070013 | intracellular organelle lumen | 3.36 | 1.92E-11 | 36.00811 |
| GO:0043229 | intracellular organelle | 1.79 | 4.16E-20 | 34.69182 |
| GO:0043226 | organelle | 1.78 | 4.77E-20 | 34.39224 |
| GO:0043232 | intracellular non-membrane-bounded organelle | 2.83 | 7.07E-13 | 34.38614 |
| GO:0043228 | non-membrane-bounded organelle | 2.83 | 7.07E-13 | 34.38614 |
| GO:0044444 | cytoplasmic part | 2.15 | 1.11E-16 | 34.30256 |
| GO:0000267 | cell fraction | 3.68 | 4.85E-10 | 34.27647 |
| GO:0016597 | amino acid binding | 9.57 | 0.0003 | 33.71395 |
| GO:0019207 | kinase regulator activity | 9.44 | 0.0003 | 33.25598 |
| GO:0042802 | identical protein binding | 4.59 | 8.40E-08 | 32.47756 |
| GO:0044424 | intracellular part | 1.62 | 1.05E-20 | 32.36567 |
| GO:0045202 | synapse | 5.13 | 4.92E-07 | 32.36022 |
| GO:0043231 | intracellular membrane-bounded organelle | 1.88 | 4.00E-17 | 30.82813 |
| GO:0043227 | membrane-bounded organelle | 1.88 | 4.12E-17 | 30.80399 |
| GO:0005622 | intracellular | 1.54 | 4.16E-20 | 29.8466 |
| GO:0005516 | calmodulin binding | 8.76 | 0.0004 | 29.76595 |
| GO:0008234 | cysteine-type peptidase activity | 7.93 | 0.0002 | 29.33283 |
| GO:0043234 | protein complex | 2.71 | 1.93E-11 | 29.03614 |
| GO:0044430 | cytoskeletal part | 3.91 | 5.08E-08 | 28.52007 |
| GO:0003779 | actin binding | 6.03 | 3.24E-05 | 27.07141 |
| GO:0044463 | cell projection part | 5.42 | 1.06E-05 | 26.96284 |
| GO:0043025 | cell soma | 5.42 | 1.06E-05 | 26.96284 |
| GO:0005626 | insoluble fraction | 3.53 | 3.18E-07 | 22.93643 |
| GO:0019904 | protein domain specific binding | 4.73 | 4.53E-05 | 20.54666 |
| GO:0004672 | protein kinase activity | 3.84 | 1.07E-05 | 19.08717 |
| GO:0004674 | protein serine/threonine kinase activity | 4.25 | 3.40E-05 | 18.99121 |
| GO:0046983 | protein dimerization activity | 3.96 | 2.24E-05 | 18.41302 |
| GO:0016301 | kinase activity | 3.38 | 1.07E-05 | 16.80068 |
| GO:0032403 | protein complex binding | 4.96 | 0.0005 | 16.37311 |
| GO:0030234 | enzyme regulator activity | 3.48 | 4.53E-05 | 15.11678 |
| GO:0032991 | macromolecular complex | 2.08 | 1.62E-07 | 14.12421 |
| GO:0016773 | phosphotransferase activity, alcohol group as acceptor | 3.25 | 5.64E-05 | 13.80834 |
| GO:0005488 | binding | 1.34 | 7.56E-11 | 13.56278 |
| GO:0016772 | transferase activity, transferring phosphorus-containing groups | 2.97 | 4.53E-05 | 12.90139 |
| GO:0005886 | plasma membrane | 2.12 | 1.76E-06 | 12.19951 |
