# Supplementary material for: GIT2 Acts as a Potential Keystone Protein in Functional Hypothalamic Networks Associated with Age-Related Phenotypic Changes in Rats
Source: PLoS One. 2012 May 14;7(5):e36975. doi: 10.1371/journal.pone.0036975 (PMC3351446; doi:10.1371/journal.pone.0036975)
Supplement: Table S8 — GeneIndexer latent semantic indexing (LSI) of significantly-regulated ‘Regulation of actin cytoskeleton’ KEGG pathway. Using the KEGG signaling pathway ‘Regulation of actin cytoskeleton’ as an input term, a list of the top 1000 implicitly-correlated (LSI correlation score >0.1) was generated using a full genome background list. (DOC) [file pone.0036975.s012.doc]

**Table S8. GeneIndexer latent semantic indexing (LSI) of significantly-regulated ‘Regulation of actin cytoskeleton’ KEGG pathway.** Using the KEGG signaling pathway ‘Regulation of actin cytoskeleton’ as an input term, a list of the top 1000 implicitly-correlated (LSI correlation score >0.1) was generated using a full genome background list.

| ***Regulation of actin cytoskeleton*** |  |
| --- | --- |
|  |  |
| **Protein Symbol** | **LSI correlation score** |
| coro2b | 0.716 |
| 2310014h01rik | 0.707 |
| lrrc16a | 0.698 |
| 2610204m08rik | 0.686 |
| 1700007i08rik | 0.66 |
| arpc4 | 0.64 |
| arpc3 | 0.63 |
| twf1 | 0.617 |
| diap3 | 0.606 |
| bc060632 | 0.603 |
| nckipsd | 0.602 |
| arpc2 | 0.602 |
| stk35 | 0.598 |
| eps8l2 | 0.593 |
| lima1 | 0.593 |
| coro1b | 0.592 |
| rhof | 0.584 |
| baiap2l1 | 0.581 |
| actr3 | 0.58 |
| arpc5 | 0.579 |
| evl | 0.577 |
| eps8l3 | 0.575 |
| svil | 0.574 |
| twf2 | 0.574 |
| tmod3 | 0.572 |
| actr2 | 0.567 |
| kptn | 0.567 |
| fnbp1l | 0.566 |
| capzb | 0.566 |
| fhod1 | 0.565 |
| vstm2l | 0.565 |
| fmnl1 | 0.561 |
| ssh1 | 0.557 |
| eps8l1 | 0.557 |
| au040829 | 0.557 |
| ssh2 | 0.555 |
| arpm1 | 0.55 |
| pfn1 | 0.549 |
| mtss1 | 0.547 |
| cfl2 | 0.546 |
| pfn2 | 0.545 |
| ssh3 | 0.541 |
| loc384848 | 0.541 |
| wipf2 | 0.537 |
| afap1 | 0.532 |
| arpc1b | 0.532 |
| coro1c | 0.532 |
| arhgap17 | 0.531 |
| arpc1a | 0.531 |
| enah | 0.53 |
| wasf3 | 0.528 |
| kif9 | 0.526 |
| nckap1l | 0.525 |
| wasl | 0.523 |
| baiap2 | 0.522 |
| wasf2 | 0.521 |
| tesk2 | 0.521 |
| daam1 | 0.519 |
| palld | 0.518 |
| pip5kl1 | 0.518 |
| wipf3 | 0.517 |
| diap1 | 0.516 |
| spire1 | 0.515 |
| dbn1 | 0.514 |
| wipf1 | 0.514 |
| ppp1r12c | 0.512 |
| actrt2 | 0.51 |
| cdc42ep5 | 0.506 |
| vasp | 0.504 |
| wasf1 | 0.503 |
| zyx | 0.501 |
| cdc42ep1 | 0.5 |
| a930037g23rik | 0.497 |
| shroom1 | 0.497 |
| actrt1 | 0.496 |
| memo1 | 0.496 |
| arhgap24 | 0.493 |
| actg1 | 0.492 |
| raph1 | 0.492 |
| dbnl | 0.49 |
| parvb | 0.487 |
| limk2 | 0.486 |
| shroom4 | 0.486 |
| 2810003c17rik | 0.485 |
| iqgap3 | 0.485 |
| cdgap | 0.484 |
| plek2 | 0.484 |
| arhgap9 | 0.483 |
| apbb1ip | 0.483 |
| capza1 | 0.483 |
| nckap1 | 0.481 |
| cdc42ep4 | 0.481 |
| arhgap21 | 0.481 |
| cfl1 | 0.481 |
| frmpd4 | 0.479 |
| abi1 | 0.479 |
| fblim1 | 0.477 |
| tesk1 | 0.474 |
| abra | 0.472 |
| triobp | 0.471 |
| macf1 | 0.471 |
| xpo6 | 0.47 |
| rab35 | 0.47 |
| d8ertd82e | 0.469 |
| layn | 0.468 |
| myo10 | 0.467 |
| mprip | 0.466 |
| cdc42ep2 | 0.465 |
| rhpn1 | 0.463 |
| dstn | 0.462 |
| klhl20 | 0.461 |
| ablim3 | 0.461 |
| rhod | 0.46 |
| actr10 | 0.459 |
| fnbp1 | 0.458 |
| srgap3 | 0.458 |
| parva | 0.457 |
| pfn4 | 0.456 |
| rhot1 | 0.455 |
| parvg | 0.455 |
| whdc1 | 0.453 |
| ablim2 | 0.453 |
| cdc42ep3 | 0.452 |
| acta1 | 0.448 |
| tln2 | 0.448 |
| diap2 | 0.447 |
| cttn | 0.447 |
| klhl17 | 0.445 |
| wdr1 | 0.444 |
| arhgef2 | 0.443 |
| rnd1 | 0.443 |
| actr3b | 0.442 |
| 3110043j09rik | 0.44 |
| klhl2 | 0.439 |
| myo7b | 0.439 |
| cnn3 | 0.439 |
| arhgap12 | 0.438 |
| lasp1 | 0.438 |
| rhot2 | 0.437 |
| abi2 | 0.434 |
| shroom2 | 0.434 |
| actr1b | 0.433 |
| pip5k1c | 0.432 |
| ablim1 | 0.432 |
| 4831426i19rik | 0.431 |
| cdc42bpa | 0.431 |
| tpm4 | 0.43 |
| pfn3 | 0.429 |
| plekhg6 | 0.429 |
| orf19 | 0.429 |
| d10wsu52e | 0.429 |
| rnd2 | 0.427 |
| pip5k1b | 0.426 |
| cald1 | 0.426 |
| nexn | 0.425 |
| 9030409g11rik | 0.424 |
| vcl | 0.423 |
| rnd3 | 0.423 |
| myo1e | 0.423 |
| ccin | 0.423 |
| ubxd5 | 0.42 |
| actn1 | 0.42 |
| myo1a | 0.419 |
| iqub | 0.419 |
| centd1 | 0.418 |
| pmv23 | 0.417 |
| cap1 | 0.416 |
| iqgap2 | 0.416 |
| fermt2 | 0.416 |
| spata13 | 0.416 |
| abi3 | 0.416 |
| fgd4 | 0.415 |
| tmod4 | 0.415 |
| rhobtb1 | 0.415 |
| elmo2 | 0.415 |
| cdc42se1 | 0.414 |
| rhpn2 | 0.414 |
| limk1 | 0.414 |
| mylc2b | 0.414 |
| 11-Sep | 0.413 |
| pacsin2 | 0.412 |
| trip10 | 0.412 |
| arhgap4 | 0.411 |
| spire2 | 0.41 |
| iqgap1 | 0.409 |
| actl7a | 0.409 |
| bin3 | 0.408 |
| tln1 | 0.407 |
| smek1 | 0.407 |
| fmnl2 | 0.406 |
| centd3 | 0.406 |
| nebl | 0.406 |
| pacsin1 | 0.405 |
| anln | 0.405 |
| cnn2 | 0.405 |
| coro2a | 0.405 |
| rp23-157o10.7 | 0.405 |
| scin | 0.405 |
| rhoq | 0.403 |
| myo1b | 0.403 |
| capg | 0.402 |
| 7-Sep | 0.401 |
| srgap2 | 0.4 |
| rac3 | 0.4 |
| cyfip2 | 0.4 |
| was | 0.399 |
| pstpip1 | 0.399 |
| fhod3 | 0.399 |
| ssx2ip | 0.399 |
| dock6 | 0.397 |
| pdlim1 | 0.396 |
| kank1 | 0.396 |
| arhgef4 | 0.396 |
| eps8 | 0.395 |
| d13mit91 | 0.395 |
| espn | 0.394 |
| nck2 | 0.394 |
| arhgap26 | 0.393 |
| ai662250 | 0.392 |
| stard13 | 0.392 |
| arhgef19 | 0.392 |
| pfdn2 | 0.391 |
| actn4 | 0.391 |
| epb4.9 | 0.39 |
| cit | 0.39 |
| fchsd1 | 0.39 |
| sorbs3 | 0.389 |
| pip5k1a | 0.389 |
| cyfip1 | 0.388 |
| cdc42bpb | 0.388 |
| sh3bp1 | 0.387 |
| rhoj | 0.387 |
| sorbs2 | 0.386 |
| rhou | 0.386 |
| ai427122 | 0.384 |
| fchsd2 | 0.384 |
| scyl3 | 0.383 |
| synpo | 0.383 |
| pak4 | 0.383 |
| gsn | 0.382 |
| cnpy2 | 0.382 |
| fgd3 | 0.382 |
| tiam2 | 0.381 |
| rgnef | 0.381 |
| nrap | 0.381 |
| pstpip2 | 0.38 |
| ppm1f | 0.38 |
| pls3 | 0.38 |
| tmod1 | 0.379 |
| rhog | 0.378 |
| tes | 0.377 |
| cnksr3 | 0.377 |
| pgm5 | 0.377 |
| d10ertd610e | 0.377 |
| swap70 | 0.375 |
| shank2 | 0.374 |
| bc067047 | 0.373 |
| rhov | 0.372 |
| tpm3 | 0.372 |
| kbtbd10 | 0.37 |
| 4631416l12rik | 0.37 |
| dnm3 | 0.37 |
| usp6nl | 0.369 |
| 2-Sep | 0.369 |
| iapls3-31 | 0.368 |
| iapt11 | 0.368 |
| arhgap10 | 0.368 |
| dnm2 | 0.367 |
| arhgap25 | 0.367 |
| epb4.1l2 | 0.367 |
| farp2 | 0.366 |
| grlf1 | 0.365 |
| myo1f | 0.365 |
| plec1 | 0.364 |
| xirp1 | 0.364 |
| raver1 | 0.364 |
| fer | 0.363 |
| rai14 | 0.363 |
| phactr3 | 0.361 |
| actn2 | 0.36 |
| phldb2 | 0.36 |
| itsn2 | 0.36 |
| 6-Sep | 0.36 |
| rtkn | 0.359 |
| iapls1-19 | 0.359 |
| def6 | 0.358 |
| shroom3 | 0.358 |
| jub | 0.358 |
| prdm12 | 0.357 |
| trio | 0.356 |
| trip6 | 0.356 |
| clic5 | 0.356 |
| mpmv19 | 0.355 |
| arhgef15 | 0.354 |
| ptpn14 | 0.354 |
| elmo1 | 0.354 |
| e130112l23rik | 0.354 |
| arhgef11 | 0.353 |
| dctn4 | 0.352 |
| fgd1 | 0.352 |
| syx1 | 0.352 |
| syx2 | 0.352 |
| 4930506m07rik | 0.352 |
| arl8a | 0.351 |
| nrk | 0.35 |
| shisa4 | 0.35 |
| mkl2 | 0.35 |
| cbll1 | 0.349 |
| fmn2 | 0.348 |
| nudt16l1 | 0.348 |
| arhgef7 | 0.348 |
| lcp1 | 0.348 |
| cap2 | 0.347 |
| arhgef6 | 0.347 |
| rdx | 0.347 |
| synpo2 | 0.346 |
| micall2 | 0.346 |
| actr6 | 0.345 |
| ppp1r9a | 0.345 |
| flnc | 0.345 |
| ktn1 | 0.345 |
| arhgdig | 0.345 |
| spna2 | 0.344 |
| cgnl1 | 0.344 |
| 6720456b07rik | 0.344 |
| filip1 | 0.343 |
| lpp | 0.343 |
| mcf2l | 0.342 |
| actc1 | 0.342 |
| phactr1 | 0.341 |
| 10-Sep | 0.34 |
| d930005d10rik | 0.34 |
| git1 | 0.34 |
| fscn1 | 0.339 |
| net1 | 0.339 |
| fbf1 | 0.339 |
| pdlim3 | 0.339 |
| actr1a | 0.338 |
| txndc9 | 0.338 |
| msn | 0.338 |
| dock10 | 0.338 |
| hcls1 | 0.338 |
| dock4 | 0.337 |
| tns1 | 0.337 |
| ppp1r9b | 0.337 |
| myo18a | 0.337 |
| arhgap5 | 0.337 |
| amot | 0.337 |
| nrbp1 | 0.336 |
| lpxn | 0.336 |
| snx26 | 0.335 |
| tpm2 | 0.335 |
| epb4.1l1 | 0.335 |
| myo3a | 0.335 |
| coro1a | 0.335 |
| fscn3 | 0.334 |
| myo5c | 0.334 |
| sorbs1 | 0.332 |
| fmn1 | 0.332 |
| myh10 | 0.331 |
| tmod2 | 0.331 |
| hip1r | 0.331 |
| pdlim4 | 0.331 |
| kalrn | 0.331 |
| rap1gds1 | 0.33 |
| arhgef9 | 0.33 |
| racgap1 | 0.33 |
| ddef1 | 0.329 |
| flii | 0.329 |
| actg2 | 0.329 |
| myo16 | 0.328 |
| 1700009n14rik | 0.327 |
| srgap1 | 0.327 |
| myo1c | 0.327 |
| mapre1 | 0.326 |
| psd | 0.326 |
| tiam1 | 0.326 |
| dock1 | 0.326 |
| arhgap22 | 0.326 |
| arhgdia | 0.325 |
| synj2 | 0.325 |
| smtnl1 | 0.325 |
| cd2ap | 0.325 |
| snx9 | 0.324 |
| sh3pxd2a | 0.323 |
| myl9 | 0.323 |
| arhgef12 | 0.322 |
| centg3 | 0.322 |
| plekhg5 | 0.322 |
| arhgap1 | 0.321 |
| mkl1 | 0.321 |
| flnb | 0.321 |
| xirp2 | 0.321 |
| slk | 0.32 |
| av249152 | 0.319 |
| ppp1r12a | 0.318 |
| mylk | 0.318 |
| plek | 0.318 |
| exoc1 | 0.317 |
| vil1 | 0.316 |
| avil | 0.316 |
| tnik | 0.316 |
| clasp2 | 0.316 |
| arhgdib | 0.316 |
| clip1 | 0.316 |
| lmod1 | 0.316 |
| rab3ip | 0.316 |
| dnm1 | 0.316 |
| exoc2 | 0.315 |
| ngef | 0.315 |
| arhgef18 | 0.315 |
| abl2 | 0.314 |
| mical3 | 0.314 |
| tnk2 | 0.314 |
| p42pop | 0.314 |
| tbcb | 0.314 |
| eif3k | 0.313 |
| d930014e17rik | 0.313 |
| gas7 | 0.313 |
| mpp1 | 0.312 |
| nck1 | 0.311 |
| arhgap15 | 0.311 |
| ptpn18 | 0.311 |
| pkn1 | 0.31 |
| phactr2 | 0.31 |
| elmo3 | 0.309 |
| ddn | 0.309 |
| grid2ip | 0.309 |
| marcks | 0.309 |
| psd4 | 0.309 |
| flna | 0.309 |
| arl8b | 0.309 |
| mical1 | 0.308 |
| gas2l2 | 0.308 |
| farp1 | 0.308 |
| fat1 | 0.308 |
| ncdn | 0.307 |
| cotl1 | 0.307 |
| shank3 | 0.307 |
| rhobtb3 | 0.307 |
| ficd | 0.306 |
| coro7 | 0.306 |
| 1-Sep | 0.306 |
| apold1 | 0.306 |
| pdlim2 | 0.306 |
| arhgap27 | 0.306 |
| ect2 | 0.305 |
| pak1ip1 | 0.305 |
| rab8b | 0.305 |
| lmo7 | 0.305 |
| rab12 | 0.305 |
| depdc2 | 0.305 |
| wtip | 0.305 |
| tbc1d10a | 0.304 |
| clasp1 | 0.304 |
| frmd6 | 0.304 |
| mcf2 | 0.304 |
| cttnbp2 | 0.304 |
| dlc1 | 0.303 |
| pacsin3 | 0.303 |
| arhgef1 | 0.303 |
| spnb3 | 0.303 |
| spnb5 | 0.303 |
| amotl1 | 0.303 |
| itsn1 | 0.302 |
| dmn | 0.301 |
| mtap1a | 0.301 |
| ldb2 | 0.301 |
| pdlim7 | 0.301 |
| cnn1 | 0.3 |
| ezr | 0.3 |
| cdc42se2 | 0.3 |
| git2 | 0.3 |
| d4mit356 | 0.3 |
| d4mit208 | 0.3 |
| gmfg | 0.3 |
| syne1 | 0.3 |
| rock2 | 0.299 |
| vezt | 0.299 |
| mylip | 0.299 |
| rufy1 | 0.299 |
| fgd2 | 0.299 |
| rock1 | 0.299 |
| kndc1 | 0.299 |
| iqsec1 | 0.299 |
| dock2 | 0.299 |
| ehbp1 | 0.298 |
| epb4.1 | 0.298 |
| pdxp | 0.297 |
| smtn | 0.297 |
| rhbdd2 | 0.297 |
| hepacam | 0.297 |
| magi1 | 0.296 |
| kif15 | 0.296 |
| ttc9 | 0.296 |
| rcsd1 | 0.296 |
| rhoh | 0.296 |
| plekho1 | 0.296 |
| spnb2 | 0.295 |
| fhl3 | 0.295 |
| pak7 | 0.294 |
| mkln1 | 0.294 |
| lims1 | 0.294 |
| mllt4 | 0.294 |
| gem | 0.294 |
| arhgap6 | 0.294 |
| myo9b | 0.293 |
| ppp1r12b | 0.293 |
| ralgps2 | 0.293 |
| centa1 | 0.292 |
| ppfia1 | 0.292 |
| arfip2 | 0.292 |
| klhl5 | 0.292 |
| arhgap29 | 0.291 |
| myh14 | 0.291 |
| scrib | 0.291 |
| pib5pa | 0.291 |
| dock9 | 0.29 |
| rp23-100c5.8 | 0.29 |
| myh9 | 0.29 |
| capza2 | 0.29 |
| kif13b | 0.29 |
| gdpd2 | 0.289 |
| lsp1 | 0.289 |
| actn3 | 0.289 |
| d2mit491 | 0.289 |
| eg629121 | 0.289 |
| clip3 | 0.288 |
| tbcd | 0.288 |
| basp1 | 0.288 |
| myo6 | 0.287 |
| rhoc | 0.287 |
| myo3b | 0.287 |
| neb | 0.287 |
| smpx | 0.286 |
| pkp4 | 0.286 |
| rhob | 0.286 |
| ppp1r14a | 0.286 |
| tuba4a | 0.285 |
| cep250 | 0.285 |
| ptpn12 | 0.285 |
| sync | 0.285 |
| itpka | 0.285 |
| chn2 | 0.285 |
| pfdn1 | 0.285 |
| akap12 | 0.285 |
| ccdc88c | 0.285 |
| sh2d3c | 0.285 |
| bin2 | 0.285 |
| vav3 | 0.284 |
| ddef2 | 0.284 |
| dgkz | 0.284 |
| 1110012m11rik | 0.284 |
| nol6 | 0.284 |
| plxnb1 | 0.284 |
| dock11 | 0.284 |
| micall1 | 0.284 |
| med28 | 0.284 |
| tns4 | 0.284 |
| syne2 | 0.283 |
| enc1 | 0.283 |
| clic4 | 0.283 |
| ralb | 0.283 |
| 1700129i15rik | 0.283 |
| loc100034363 | 0.283 |
| 4930488e11rik | 0.283 |
| loc666244 | 0.283 |
| dnmbp | 0.283 |
| tmsb4x | 0.283 |
| tgfb1i1 | 0.283 |
| kank2 | 0.283 |
| myl6b | 0.282 |
| ehd2 | 0.281 |
| actb | 0.281 |
| myom1 | 0.281 |
| centd2 | 0.281 |
| mybph | 0.281 |
| unc45b | 0.28 |
| pak3 | 0.28 |
| tpm1 | 0.279 |
| mylk2 | 0.279 |
| ccdc88a | 0.278 |
| fermt1 | 0.277 |
| vav2 | 0.277 |
| add2 | 0.276 |
| riok3 | 0.276 |
| klhdc1 | 0.276 |
| sh3kbp1 | 0.276 |
| tns3 | 0.276 |
| 12-Sep | 0.275 |
| arl6ip6 | 0.275 |
| d11ertd49e | 0.274 |
| bc004728 | 0.274 |
| lims2 | 0.274 |
| exoc4 | 0.274 |
| 5830411g16rik | 0.274 |
| d13mit249 | 0.273 |
| pak2 | 0.273 |
| arhgef10 | 0.273 |
| ttl | 0.272 |
| pard3 | 0.272 |
| kank4 | 0.272 |
| kank3 | 0.272 |
| pkn3 | 0.271 |
| eppk1 | 0.271 |
| inf2 | 0.271 |
| obscn | 0.271 |
| clta | 0.271 |
| strn4 | 0.271 |
| pkn2 | 0.27 |
| myh2 | 0.27 |
| reps1 | 0.27 |
| ldb3 | 0.27 |
| gripap1 | 0.269 |
| fkbp15 | 0.269 |
| phactr4 | 0.269 |
| chn1 | 0.269 |
| ctnna1 | 0.269 |
| ddefl1 | 0.269 |
| palm | 0.268 |
| pfdn4 | 0.268 |
| pip4k2a | 0.268 |
| fyb | 0.268 |
| ptpn20 | 0.267 |
| rab13 | 0.267 |
| trim55 | 0.267 |
| ptpn21 | 0.267 |
| shank1 | 0.267 |
| amph | 0.267 |
| osbpl3 | 0.267 |
| unc45a | 0.267 |
| pmv19 | 0.266 |
| mtap1s | 0.266 |
| pxn | 0.265 |
| gas2l1 | 0.264 |
| plxnc1 | 0.264 |
| myrip | 0.264 |
| tbca | 0.264 |
| sdcbp | 0.263 |
| sipa1l1 | 0.263 |
| maea | 0.263 |
| ahnak | 0.263 |
| sipa1 | 0.263 |
| rras | 0.262 |
| myo1h | 0.262 |
| nostrin | 0.262 |
| ints4 | 0.262 |
| 6430548m08rik | 0.262 |
| stmn3 | 0.262 |
| dctn1 | 0.262 |
| rsu1 | 0.261 |
| unc84b | 0.261 |
| myom2 | 0.261 |
| amotl2 | 0.261 |
| exoc7 | 0.261 |
| clic3 | 0.261 |
| plekhb1 | 0.261 |
| tubb5 | 0.26 |
| c130038g02rik | 0.26 |
| map2k1ip1 | 0.26 |
| pscd2 | 0.26 |
| tubb2a | 0.26 |
| dpysl2 | 0.26 |
| lmod2 | 0.259 |
| rac2 | 0.259 |
| ermn | 0.259 |
| plekha2 | 0.259 |
| iqsec2 | 0.259 |
| capza3 | 0.258 |
| ppm1e | 0.258 |
| ilk | 0.258 |
| rgl3 | 0.258 |
| tuba8 | 0.257 |
| plekha7 | 0.257 |
| nf2 | 0.256 |
| marcksl1 | 0.256 |
| arf6 | 0.256 |
| gphn | 0.256 |
| fsd1 | 0.256 |
| ctnnd1 | 0.256 |
| efha1 | 0.256 |
| magi2 | 0.256 |
| mylk3 | 0.256 |
| pscdbp | 0.256 |
| ppl | 0.255 |
| ppp1r14b | 0.255 |
| slc9a3r2 | 0.255 |
| saps1 | 0.255 |
| mobkl3 | 0.255 |
| rab11fip3 | 0.255 |
| ftcd | 0.255 |
| rala | 0.255 |
| grit | 0.254 |
| ccpg1 | 0.254 |
| rab21 | 0.254 |
| eml3 | 0.253 |
| snx18 | 0.253 |
| myo9a | 0.253 |
| synj1 | 0.253 |
| abp1 | 0.253 |
| schip1 | 0.253 |
| myom3 | 0.253 |
| prkcbp1 | 0.252 |
| iba1 | 0.252 |
| rapgef2 | 0.252 |
| mtap7 | 0.252 |
| mast1 | 0.252 |
| shbdp1 | 0.252 |
| ostf1 | 0.252 |
| unc84a | 0.251 |
| a130090k04rik | 0.251 |
| atp6v1e1 | 0.251 |
| loc641201 | 0.251 |
| mirn145 | 0.251 |
| wdr44 | 0.251 |
| rab11fip2 | 0.25 |
| tuba1b | 0.249 |
| tubgcp6 | 0.249 |
| tjap1 | 0.249 |
| itgb1bp1 | 0.249 |
| dapp1 | 0.249 |
| ivns1abp | 0.249 |
| rab11fip1 | 0.249 |
| rab8a | 0.249 |
| myo15b | 0.248 |
| mpp5 | 0.248 |
| ptprq | 0.248 |
| cib1 | 0.248 |
| nedd9 | 0.248 |
| lrrc4b | 0.248 |
| mycbp2 | 0.248 |
| fert2 | 0.248 |
| dock8 | 0.248 |
| pag1 | 0.248 |
| tm4sf5 | 0.248 |
| myh4 | 0.248 |
| podxl | 0.248 |
| frmd4b | 0.248 |
| rps4x | 0.247 |
| tbkbp1 | 0.247 |
| pscd1 | 0.247 |
| kirrel | 0.247 |
| lrrfip1 | 0.247 |
| pdlim5 | 0.246 |
| synj2bp | 0.246 |
| actl6b | 0.246 |
| actr8 | 0.246 |
| ctnnal1 | 0.246 |
| tubg1 | 0.246 |
| 1700016k13rik | 0.246 |
| 9-Sep | 0.246 |
| eml5 | 0.246 |
| erbb2ip | 0.245 |
| clic1 | 0.245 |
| rab34 | 0.245 |
| tubg2 | 0.245 |
| wdr68 | 0.245 |
| pcyt1b | 0.245 |
| dnmlp1 | 0.245 |
| dixdc1 | 0.245 |
| centg1 | 0.245 |
| 1110006o17rik | 0.245 |
| dgkg | 0.244 |
| pard6b | 0.244 |
| trim39 | 0.244 |
| pak6 | 0.244 |
| tchp | 0.244 |
| rab11b | 0.244 |
| nphs1 | 0.244 |
| abr | 0.244 |
| cgn | 0.244 |
| klhl1 | 0.244 |
| myl6 | 0.244 |
| cdc42l2 | 0.243 |
| cdc42l1 | 0.243 |
| cdc42l3 | 0.243 |
| bin1 | 0.243 |
| ctnna2 | 0.243 |
| d10mit109 | 0.243 |
| spnb1 | 0.243 |
| arhgef10l | 0.243 |
| igsf5 | 0.243 |
| epb4.1l4b | 0.242 |
| sacm1l | 0.242 |
| rph3a | 0.242 |
| pcdhgc3 | 0.242 |
| homer3 | 0.241 |
| crip2 | 0.241 |
| tppp | 0.241 |
| akap9 | 0.241 |
| nisch | 0.241 |
| garnl4 | 0.241 |
| pard6a | 0.24 |
| epb4.1l3 | 0.24 |
| gmip | 0.24 |
| pard6g | 0.24 |
| skap2 | 0.24 |
| ehd1 | 0.239 |
| taok1 | 0.239 |
| dpysl3 | 0.239 |
| emo1 | 0.238 |
| pi4k2a | 0.238 |
| gipc1 | 0.238 |
| pcnp | 0.238 |
| sh3rf1 | 0.238 |
| stk10 | 0.238 |
| ddx47 | 0.238 |
| myot | 0.238 |
| tuba1a | 0.237 |
| dync1i1 | 0.237 |
| myo5b | 0.237 |
| ctnnd2 | 0.237 |
| slc9a5 | 0.237 |
| grasp | 0.237 |
| eif6 | 0.237 |
| tubb2c | 0.237 |
| vill | 0.237 |
| bcar3 | 0.237 |
| rod1 | 0.237 |
| tjp2 | 0.237 |
| spef1 | 0.237 |
| katna1 | 0.237 |
| katnb1 | 0.236 |
| skap1 | 0.236 |
| sec14l1 | 0.236 |
| crocc | 0.236 |
| rasa4 | 0.236 |
| mib2 | 0.236 |
| poldip2 | 0.236 |
| camk2n2 | 0.236 |
| nup85 | 0.236 |
| inadl | 0.235 |
| mirn143 | 0.235 |
| a230067g21rik | 0.235 |
| pik3r4 | 0.235 |
| exoc8 | 0.235 |
| rasgrf2 | 0.234 |
| tbcel | 0.234 |
| centb2 | 0.234 |
| snx33 | 0.234 |
| fermt3 | 0.233 |
| rp23-136k12.4 | 0.233 |
| tubb2b | 0.233 |
| abi3bp | 0.232 |
| rab9b | 0.232 |
| rap1a | 0.232 |
| strn | 0.232 |
| pik3c2b | 0.232 |
| mapre3 | 0.232 |
| b230339m05rik | 0.232 |
| cntnap1 | 0.232 |
| cmya5 | 0.232 |
| rnf6 | 0.232 |
| micalcl | 0.231 |
| sh3gl2 | 0.231 |
| bves | 0.231 |
| cnksr1 | 0.231 |
| mtap4 | 0.231 |
| igfn1 | 0.231 |
| spred2 | 0.231 |
| obsl1 | 0.231 |
| add3 | 0.231 |
| strn3 | 0.231 |
| dst | 0.231 |
| gdi1 | 0.231 |
| rin1 | 0.23 |
| myoz3 | 0.23 |
| spna1 | 0.23 |
| dimt1 | 0.23 |
| ank3 | 0.23 |
| 4933407c03rik | 0.23 |
| evpl | 0.23 |
| bcar1 | 0.23 |
| ralgds | 0.23 |
| sh3gl3 | 0.229 |
| csrp2 | 0.229 |
| d11mit116 | 0.229 |
| centb1 | 0.229 |
| nek3 | 0.229 |
| pi4kb | 0.229 |
| pak1 | 0.229 |
| fmnl3 | 0.229 |
| gdi2 | 0.229 |
| tenc1 | 0.229 |
| 6330417g02rik | 0.228 |
| arl2 | 0.228 |
| dlgap1 | 0.228 |
| cd302 | 0.228 |
| troap | 0.228 |
| mras | 0.228 |
| ptprm | 0.228 |
| pscd3 | 0.228 |
| sdk1 | 0.228 |
| dctn2 | 0.228 |
| bicd2 | 0.228 |
| mpp2 | 0.228 |
| pknox2 | 0.228 |
| ppfia4 | 0.228 |
| ttc3 | 0.228 |
| saps3 | 0.227 |
| gas8 | 0.227 |
| sntb2 | 0.227 |
| cobl | 0.227 |
| whrn | 0.227 |
| stmn4 | 0.227 |
| spred1 | 0.227 |
| mtap1b | 0.227 |
| khdrbs2 | 0.227 |
| ankrd28 | 0.226 |
| bicd1 | 0.226 |
| dlgap3 | 0.226 |
| lrrc7 | 0.226 |
| rufy3 | 0.226 |
| reps2 | 0.226 |
| a930041i02rik | 0.226 |
| mypn | 0.226 |
| ppp1r16b | 0.226 |
| diras2 | 0.226 |
| astt1 | 0.226 |
| astt2 | 0.226 |
| astt3 | 0.226 |
| epb4.1l5 | 0.225 |
| 4-Sep | 0.225 |
| arhgef3 | 0.225 |
| dynll2 | 0.225 |
| rit2 | 0.225 |
| gna13 | 0.225 |
| ralgps1 | 0.225 |
| pvrl3 | 0.225 |
| sh3gl1 | 0.225 |
| 8030451f13rik | 0.224 |
| cabyr | 0.224 |
| mtpn | 0.224 |
| slc4a1ap | 0.224 |
| alpk1 | 0.224 |
| homer2 | 0.224 |
| spnb4 | 0.224 |
| exoc5 | 0.224 |
| akap13 | 0.224 |
| gna12 | 0.223 |
| pitpnm1 | 0.223 |
| trappc9 | 0.223 |
| utrn | 0.223 |
| dock3 | 0.223 |
| palm2-akap2 | 0.223 |
| raver2 | 0.222 |
| zfyve27 | 0.222 |
| jund2 | 0.222 |
| stom | 0.221 |
| cct6b | 0.221 |
| arhgef17 | 0.221 |
| ropn1 | 0.221 |
| rap2a | 0.221 |
| 2310039e09rik | 0.221 |
| trappc4 | 0.221 |
| sema4g | 0.221 |
| ottmusg00000022462 | 0.22 |
| rab37 | 0.22 |
| ube2q2 | 0.22 |
| tfpt | 0.22 |
| ehd4 | 0.22 |
| daam2 | 0.22 |
| rab25 | 0.22 |
| ocrl | 0.22 |
| plce1 | 0.219 |
| itgb1bp2 | 0.219 |
| ptk7 | 0.219 |
| rab4a | 0.219 |
| ranbp10 | 0.219 |
| aamp | 0.219 |
| bnip2 | 0.219 |
| fbxo10 | 0.219 |
| sestd1 | 0.219 |
| akap5 | 0.218 |
| sh2b2 | 0.218 |
| ipp | 0.218 |
| llgl1 | 0.218 |
| zfp185 | 0.218 |
| dapk3 | 0.218 |
| tg(krt14-cre)1efu | 0.218 |
| ermap | 0.218 |
| bcnp1 | 0.218 |
| ajap1 | 0.218 |
| tagln2 | 0.218 |
| zfp639 | 0.218 |
| dync1li2 | 0.217 |
| srf | 0.217 |
| flot2 | 0.217 |
| myo18b | 0.217 |
| mapre2 | 0.217 |
| tagln3 | 0.217 |
| gda | 0.217 |
| inppl1 | 0.217 |
| megf11 | 0.217 |
| rin2 | 0.217 |
| ptpn23 | 0.216 |
| cnpl | 0.216 |
| mettl7b | 0.216 |
| kif23 | 0.216 |
| akap2 | 0.216 |
