# Supplementary material for: GIT2 Acts as a Potential Keystone Protein in Functional Hypothalamic Networks Associated with Age-Related Phenotypic Changes in Rats
Source: PLoS One. 2012 May 14;7(5):e36975. doi: 10.1371/journal.pone.0036975 (PMC3351446; doi:10.1371/journal.pone.0036975)
Supplement: Table S9 — GeneIndexer latent semantic indexing (LSI) of significantly-regulated ‘Chemokine signaling’ KEGG pathway. Using the KEGG signaling pathway ‘Chemokine signaling’ as an input term, a list of the top 1000 implicitly-correlated (LSI correlation score >0.1) was generated using a full genome background list. (DOC) [file pone.0036975.s013.doc]

**Table S9. GeneIndexer latent semantic indexing (LSI) of significantly-regulated ‘Chemokine signaling’ KEGG pathway.** Using the KEGG signaling pathway ‘Chemokine signaling’ as an input term, a list of the top 1000 implicitly-correlated (LSI correlation score >0.1) was generated using a full genome background list.

| ***Chemokine signaling*** |  |
| --- | --- |
|  |  |
| **Protein Symbol** | **LSI correlation score** |
| ccrl1 | 0.653 |
| oaf | 0.595 |
| d9mit36 | 0.585 |
| xcr1 | 0.576 |
| ccrl2 | 0.571 |
| ccr10 | 0.566 |
| ccbp2 | 0.564 |
| ccl12 | 0.564 |
| ccr1l1 | 0.561 |
| ccl19 | 0.555 |
| ccl9 | 0.542 |
| ccr8 | 0.541 |
| ccl7 | 0.54 |
| ccl21c | 0.53 |
| loc100042493 | 0.518 |
| rgs13 | 0.516 |
| ccl21b | 0.516 |
| ccl22 | 0.516 |
| ccl1 | 0.515 |
| ccl28 | 0.515 |
| ccr4 | 0.513 |
| cxcr5 | 0.509 |
| cxcr6 | 0.501 |
| ccl27 | 0.493 |
| ccr6 | 0.491 |
| ccl25 | 0.488 |
| cxcl13 | 0.482 |
| ccl8 | 0.482 |
| ccl20 | 0.478 |
| cxcl11 | 0.468 |
| ccr3 | 0.466 |
| ccr7 | 0.464 |
| gpr15 | 0.464 |
| ccl17 | 0.463 |
| rgs1 | 0.462 |
| ccl16-ps | 0.462 |
| ccr9 | 0.457 |
| cxcl3 | 0.455 |
| ccr1 | 0.455 |
| xcl1 | 0.453 |
| ccl6 | 0.453 |
| cxcl15 | 0.45 |
| iapls3-29 | 0.45 |
| cxcl9 | 0.443 |
| gpr84 | 0.44 |
| cxcr3 | 0.433 |
| ccl4 | 0.431 |
| ccl24 | 0.423 |
| lyzl6 | 0.422 |
| il8ra | 0.412 |
| cxcl5 | 0.412 |
| cxcr7 | 0.409 |
| dock2 | 0.404 |
| ccl11 | 0.402 |
| cmklr1 | 0.4 |
| cx3cl1-rs1 | 0.395 |
| ccl26 | 0.394 |
| gpr33 | 0.39 |
| cxcl14 | 0.377 |
| cxcl2 | 0.377 |
| s1pr4 | 0.372 |
| zc3h12a | 0.371 |
| pik3r6 | 0.37 |
| cx3cl1 | 0.37 |
| act1 | 0.363 |
| cxcl16 | 0.363 |
| rgs16 | 0.358 |
| ccl3 | 0.356 |
| il8rb | 0.355 |
| cxcl10 | 0.353 |
| defb14 | 0.351 |
| il17re | 0.351 |
| fpr2 | 0.35 |
| fpr3 | 0.349 |
| loc100045000 | 0.349 |
| ccr2 | 0.349 |
| cxcl1 | 0.348 |
| cx3cr1 | 0.344 |
| s1pr5 | 0.339 |
| akirin1 | 0.339 |
| hrh4 | 0.338 |
| gpr31c | 0.336 |
| zc3h12b | 0.333 |
| rgs18 | 0.332 |
| fpr1 | 0.33 |
| cxcl17 | 0.329 |
| cklf | 0.328 |
| gpr44 | 0.325 |
| d5mit369 | 0.324 |
| s1pr1 | 0.323 |
| sash3 | 0.323 |
| card10 | 0.32 |
| il34 | 0.317 |
| gna14 | 0.316 |
| darc | 0.315 |
| ebi2 | 0.312 |
| ltb4r1 | 0.312 |
| rarres2 | 0.31 |
| tslp | 0.309 |
| d11mit119 | 0.309 |
| cd180 | 0.309 |
| tg(lck-cre)i57jxm | 0.308 |
| fpr-rs6 | 0.307 |
| loc100048461 | 0.305 |
| il25 | 0.304 |
| adrbk2 | 0.304 |
| ly6c1 | 0.303 |
| ppbp | 0.303 |
| gpr18 | 0.303 |
| s1pr3 | 0.301 |
| hspa14 | 0.3 |
| madcam1 | 0.296 |
| il31 | 0.294 |
| tlr5 | 0.294 |
| ccdc100 | 0.293 |
| lrrfip2 | 0.292 |
| gnai2 | 0.29 |
| nup85 | 0.29 |
| pf4 | 0.29 |
| nrp | 0.29 |
| xmv41 | 0.289 |
| lrrc19 | 0.289 |
| trem3 | 0.288 |
| il33 | 0.287 |
| zc3h12c | 0.286 |
| ecsit | 0.284 |
| ffar2 | 0.284 |
| 6330403m23rik | 0.283 |
| rgs3 | 0.283 |
| gnai1 | 0.281 |
| socs5 | 0.281 |
| s1pr2 | 0.279 |
| zfp414 | 0.278 |
| gnai3 | 0.278 |
| il20 | 0.278 |
| tnfsf18 | 0.278 |
| p2ry14 | 0.278 |
| tnip3 | 0.277 |
| trafd1 | 0.277 |
| mirn147 | 0.276 |
| sh2d3c | 0.275 |
| ccl5 | 0.275 |
| ksr2 | 0.274 |
| sigirr | 0.274 |
| sarm1 | 0.274 |
| lax1 | 0.273 |
| oscar | 0.273 |
| ccdc88c | 0.273 |
| pik3cd | 0.272 |
| gpr132 | 0.272 |
| spred2 | 0.271 |
| wdr34 | 0.271 |
| ltbr | 0.271 |
| clec4e | 0.27 |
| gna15 | 0.27 |
| p2ry6 | 0.269 |
| paqr6 | 0.269 |
| paqr9 | 0.269 |
| bc067047 | 0.269 |
| ticam2 | 0.268 |
| zc3h12d | 0.268 |
| il17d | 0.268 |
| il17rc | 0.268 |
| tnfrsf12a | 0.267 |
| gpr4 | 0.266 |
| il17ra | 0.266 |
| lpar3 | 0.265 |
| psma5 | 0.265 |
| zbtb1 | 0.265 |
| arr3 | 0.264 |
| crlf2 | 0.264 |
| gna13 | 0.264 |
| grk6 | 0.262 |
| bc037156 | 0.262 |
| fzd2 | 0.261 |
| arhgef1 | 0.261 |
| mcpt3 | 0.261 |
| sharpin | 0.261 |
| il16 | 0.261 |
| stap2 | 0.261 |
| retnla | 0.261 |
| ltb4r2 | 0.261 |
| prkd2 | 0.261 |
| sphk2 | 0.261 |
| chi3l3 | 0.26 |
| loc546644 | 0.26 |
| card9 | 0.26 |
| sh2d2a | 0.26 |
| gpbar1 | 0.259 |
| tnfsf14 | 0.259 |
| card11 | 0.258 |
| 4732429d16rik | 0.258 |
| il31ra | 0.258 |
| irgq | 0.258 |
| afap1l2 | 0.258 |
| irak3 | 0.257 |
| a530064d06rik | 0.257 |
| traf7 | 0.257 |
| nfkbiz | 0.257 |
| lztfl1 | 0.256 |
| mesdc1 | 0.255 |
| dok3 | 0.255 |
| dusp16 | 0.255 |
| crtam | 0.255 |
| mapkap1 | 0.255 |
| 5430435g22rik | 0.254 |
| pscdbp | 0.254 |
| ptafr | 0.254 |
| itgb7 | 0.254 |
| sbno2 | 0.254 |
| dok2 | 0.253 |
| irak4 | 0.253 |
| tnfaip8l2 | 0.253 |
| gcsfis | 0.253 |
| ppil5 | 0.252 |
| zfp641 | 0.252 |
| plekhm3 | 0.252 |
| spred1 | 0.251 |
| rgs10 | 0.251 |
| aida | 0.251 |
| gpr55 | 0.251 |
| nrk | 0.251 |
| irak2 | 0.251 |
| lpar4 | 0.25 |
| d9mit19 | 0.25 |
| tollip | 0.25 |
| tnfsf12 | 0.25 |
| card14 | 0.25 |
| caprin2 | 0.25 |
| bcl10 | 0.25 |
| clec4b2 | 0.249 |
| gpr17 | 0.249 |
| creb3 | 0.249 |
| anks1 | 0.248 |
| hebp1 | 0.248 |
| itgae | 0.248 |
| clnk | 0.247 |
| cd300a | 0.247 |
| iba1 | 0.246 |
| map4k4 | 0.246 |
| gmfb | 0.246 |
| lgr6 | 0.246 |
| lsp1 | 0.245 |
| asb15 | 0.245 |
| socs7 | 0.245 |
| rgs17 | 0.245 |
| peli2 | 0.245 |
| map4k5 | 0.244 |
| nanos1 | 0.244 |
| itk | 0.244 |
| gm944 | 0.244 |
| rhod | 0.244 |
| clec7a | 0.243 |
| adrbk1 | 0.243 |
| eg232801 | 0.243 |
| lime1 | 0.243 |
| sh2d4a | 0.243 |
| ccrn4l | 0.243 |
| rgs14 | 0.243 |
| rassf5 | 0.242 |
| pik3r5 | 0.242 |
| pdlim2 | 0.242 |
| cnksr1 | 0.242 |
| sucnr1 | 0.242 |
| tg(tek-cre)12flv | 0.242 |
| tirap | 0.241 |
| stk10 | 0.241 |
| il20ra | 0.241 |
| rtp1 | 0.241 |
| osbpl9 | 0.241 |
| zranb1 | 0.241 |
| nsmaf | 0.24 |
| skap1 | 0.24 |
| fndc1 | 0.24 |
| d4mit286 | 0.24 |
| ltb | 0.24 |
| akirin2 | 0.24 |
| peli1 | 0.239 |
| d10mit161 | 0.239 |
| fem1a | 0.239 |
| cd300lb | 0.239 |
| ffar3 | 0.239 |
| dact1 | 0.239 |
| il17f | 0.239 |
| tlr8 | 0.239 |
| sirpb1 | 0.239 |
| sgpp2 | 0.238 |
| hsh2d | 0.238 |
| tpst1 | 0.238 |
| fzd8 | 0.238 |
| dapp1 | 0.238 |
| chia | 0.237 |
| rasgrp2 | 0.237 |
| pglyrp2 | 0.237 |
| hes3 | 0.237 |
| af251705 | 0.237 |
| 4932409i22rik | 0.237 |
| eg244911 | 0.237 |
| 3300001a09rik | 0.237 |
| irak1bp1 | 0.237 |
| mrc1 | 0.237 |
| map4k2 | 0.237 |
| fpr-rs3 | 0.236 |
| slamf8 | 0.236 |
| socs4 | 0.236 |
| tnfrsf21 | 0.236 |
| camp | 0.236 |
| ankrd6 | 0.236 |
| ripk2 | 0.236 |
| 3930401k13rik | 0.236 |
| pik3ap1 | 0.236 |
| wdr26 | 0.235 |
| trem2 | 0.235 |
| gpr68 | 0.235 |
| socs6 | 0.235 |
| shoc2 | 0.235 |
| tm7sf4 | 0.235 |
| tspan12 | 0.235 |
| cd5l | 0.235 |
| lpar5 | 0.235 |
| shc4 | 0.234 |
| zcchc11 | 0.234 |
| ripk4 | 0.234 |
| dok1 | 0.234 |
| rspo2 | 0.234 |
| tnfrsf25 | 0.233 |
| slfn2 | 0.233 |
| nlrp12 | 0.233 |
| epha10 | 0.233 |
| sit1 | 0.233 |
| txk | 0.232 |
| pik3cb | 0.232 |
| trat1 | 0.232 |
| treml2 | 0.232 |
| fpr-rs4 | 0.232 |
| cd207 | 0.232 |
| gpr6 | 0.232 |
| tpst2 | 0.232 |
| spon2 | 0.232 |
| cxxc4 | 0.231 |
| defb2 | 0.231 |
| lpxn | 0.231 |
| gpr1 | 0.231 |
| rspo3 | 0.231 |
| trip6 | 0.231 |
| defb6 | 0.231 |
| klf2 | 0.231 |
| tmed1 | 0.231 |
| traf3ip2 | 0.23 |
| otud7b | 0.23 |
| tas2r107 | 0.23 |
| fzd5 | 0.23 |
| peli3 | 0.23 |
| d4mit237a | 0.23 |
| trem1 | 0.229 |
| gpr83 | 0.229 |
| sgpp1 | 0.229 |
| lyz2 | 0.229 |
| rps6ka4 | 0.229 |
| ifna1 | 0.229 |
| arrb2 | 0.229 |
| rnf138 | 0.229 |
| asb3 | 0.229 |
| loc641201 | 0.229 |
| grk5 | 0.228 |
| trim30 | 0.228 |
| defb8 | 0.228 |
| dusp14 | 0.228 |
| tmem9 | 0.228 |
| lpar1 | 0.228 |
| shisa2 | 0.228 |
| sla2 | 0.228 |
| trib2 | 0.228 |
| shcbp1 | 0.227 |
| ric8 | 0.227 |
| plxnc1 | 0.227 |
| emr4 | 0.227 |
| rgs12 | 0.227 |
| tnfsf15 | 0.227 |
| 9130404d14rik | 0.226 |
| tlr11 | 0.226 |
| gpr139 | 0.226 |
| irg1 | 0.226 |
| marco | 0.226 |
| tifab | 0.226 |
| olfr677 | 0.226 |
| olfr661 | 0.226 |
| olfr547 | 0.226 |
| mirn146 | 0.226 |
| lpar2 | 0.226 |
| tbc1d10c | 0.225 |
| rhoh | 0.225 |
| lna1 | 0.225 |
| rasgrp1 | 0.225 |
| gipc3 | 0.225 |
| trappc9 | 0.225 |
| scye1 | 0.224 |
| nlk | 0.224 |
| olfr985 | 0.224 |
| rac2 | 0.224 |
| samhd1 | 0.224 |
| nfkbid | 0.224 |
| map4k1 | 0.224 |
| zbed3 | 0.224 |
| tnfsf8 | 0.224 |
| rgs20 | 0.224 |
| lrrfip1 | 0.224 |
| dgka | 0.223 |
| gpr65 | 0.223 |
| il27 | 0.223 |
| cd83 | 0.223 |
| c5ar1 | 0.223 |
| tlr13 | 0.223 |
| fgr | 0.223 |
| map3k14 | 0.223 |
| bmx | 0.223 |
| map3k7ip3 | 0.222 |
| sprr2b | 0.222 |
| tlr12 | 0.222 |
| tmem11 | 0.222 |
| lphn3 | 0.222 |
| chn2 | 0.222 |
| tifa | 0.222 |
| fzd7 | 0.222 |
| bc023829 | 0.222 |
| ubash3b | 0.221 |
| phlppl | 0.221 |
| sirpa | 0.221 |
| cmtm8 | 0.221 |
| aif1 | 0.221 |
| clec5a | 0.221 |
| d1mit508 | 0.22 |
| ccdc88a | 0.22 |
| osmr | 0.22 |
| c3ar1 | 0.22 |
| icam2 | 0.22 |
| ptk7 | 0.22 |
| lzic | 0.22 |
| gipc2 | 0.22 |
| stap1 | 0.22 |
| timd2 | 0.22 |
| jak3 | 0.22 |
| dok4 | 0.219 |
| grit | 0.219 |
| il27ra | 0.219 |
| cdkl2 | 0.219 |
| daam2 | 0.219 |
| pik3c2b | 0.219 |
| cbll1 | 0.219 |
| aplnr | 0.219 |
| 381484 | 0.218 |
| cd300lf | 0.218 |
| mrgprb2 | 0.218 |
| il20rb | 0.218 |
| fyb | 0.218 |
| dact2 | 0.218 |
| rasal1 | 0.218 |
| 9130404h23rik | 0.218 |
| ly96 | 0.218 |
| centd3 | 0.218 |
| il17rd | 0.218 |
| stk4 | 0.218 |
| arrb1 | 0.218 |
| olfr553 | 0.218 |
| olfr557 | 0.218 |
| olfr461 | 0.218 |
| olfr556 | 0.218 |
| swap70 | 0.217 |
| tnip2 | 0.217 |
| il22ra1 | 0.217 |
| ptk2b | 0.217 |
| tlr3 | 0.217 |
| d930005d10rik | 0.217 |
| cblb | 0.217 |
| sphk1 | 0.217 |
| ripk3 | 0.217 |
| il22 | 0.217 |
| stk40 | 0.217 |
| sema4d | 0.217 |
| c330002i19rik | 0.217 |
| tlr1 | 0.216 |
| plekha2 | 0.216 |
| gna12 | 0.216 |
| chi3l4 | 0.216 |
| tnfrsf14 | 0.216 |
| dkk4 | 0.216 |
| rtp4 | 0.216 |
| eg627648 | 0.215 |
| cd200r1 | 0.215 |
| mlf2 | 0.215 |
| map2k1ip1 | 0.215 |
| kremen2 | 0.215 |
| 2610018g03rik | 0.215 |
| map3k8 | 0.215 |
| itgb1bp1 | 0.215 |
| lilrb3 | 0.215 |
| map3k3 | 0.215 |
| cd69 | 0.215 |
| tg(dvl2)7gsb | 0.215 |
| il17rb | 0.215 |
| mapk15 | 0.215 |
| fzd6 | 0.215 |
| osm | 0.215 |
| ducm1 | 0.214 |
| gab3 | 0.214 |
| dtx4 | 0.214 |
| timd4 | 0.214 |
| sorbs3 | 0.214 |
| gpr142 | 0.214 |
| dusp10 | 0.214 |
| th1l | 0.214 |
| relt | 0.214 |
| rgs5 | 0.214 |
| tnfsf9 | 0.214 |
| rftn1 | 0.214 |
| plxnd1 | 0.214 |
| spred3 | 0.214 |
| ppm1m | 0.214 |
| cxcl12 | 0.213 |
| rgs22 | 0.213 |
| nfatc2ip | 0.213 |
| mobkl1a | 0.213 |
| olfr705 | 0.213 |
| tlr7 | 0.213 |
| rgs8 | 0.213 |
| d19mit104 | 0.213 |
| 9830130m13rik | 0.213 |
| arhgef12 | 0.213 |
| rnf125 | 0.213 |
| olfr937 | 0.213 |
| retnlb | 0.213 |
| jmjd6 | 0.213 |
| eg668725 | 0.213 |
| gpr3 | 0.213 |
| 4933407c03rik | 0.212 |
| ms4a4b | 0.212 |
| il28a | 0.212 |
| zfp383 | 0.212 |
| rasd1 | 0.212 |
| hsp86-ps1 | 0.212 |
| nkap | 0.212 |
| ptgdr | 0.212 |
| sh2b3 | 0.212 |
| pstpip2 | 0.212 |
| mapkbp1 | 0.212 |
| dpp4 | 0.212 |
| rorc | 0.212 |
| ifi47 | 0.212 |
| dice2 | 0.211 |
| pik3c2g | 0.211 |
| dullard | 0.211 |
| zbp1 | 0.211 |
| sema7a | 0.211 |
| fzd10 | 0.211 |
| itgb2l | 0.211 |
| il24 | 0.211 |
| tmem173 | 0.211 |
| fstl1 | 0.211 |
| rgs19 | 0.211 |
| sell | 0.211 |
| trib1 | 0.211 |
| tec | 0.211 |
| pitpnm3 | 0.211 |
| cnpy3 | 0.211 |
| pik3r2 | 0.211 |
| taok3 | 0.211 |
| irak1 | 0.211 |
| rap2a | 0.21 |
| nfam1 | 0.21 |
| nxn | 0.21 |
| plce1 | 0.21 |
| fzd1 | 0.21 |
| dice1 | 0.21 |
| clec9a | 0.21 |
| nkd1 | 0.21 |
| spn | 0.21 |
| il18r1 | 0.21 |
| dll4 | 0.209 |
| krtap8-1 | 0.209 |
| e430004n04rik | 0.209 |
| card6 | 0.209 |
| lgals9 | 0.209 |
| map3k2 | 0.209 |
| gpr35 | 0.209 |
| rtkn | 0.209 |
| malt1 | 0.209 |
| lair1 | 0.209 |
| samsn1 | 0.209 |
| glrx3 | 0.209 |
| emv20 | 0.208 |
| skap2 | 0.208 |
| flii | 0.208 |
| stk38l | 0.208 |
| tnfrsf18 | 0.208 |
| matk | 0.208 |
| tbkbp1 | 0.208 |
| bcar3 | 0.208 |
| sh2d1b2 | 0.208 |
| hck | 0.208 |
| clec4n | 0.207 |
| ubash3a | 0.207 |
| srms | 0.207 |
| chid1 | 0.207 |
| d5mit20 | 0.207 |
| tlr6 | 0.207 |
| nlk-ps1 | 0.207 |
| d2mit444 | 0.207 |
| mgl2 | 0.207 |
| gpr97 | 0.207 |
| arrdc2 | 0.207 |
| ror2 | 0.207 |
| gnao1 | 0.207 |
| sh3rf1 | 0.206 |
| treml4 | 0.206 |
| ifnk | 0.206 |
| il19 | 0.206 |
| mesdc2 | 0.206 |
| mirn155 | 0.206 |
| il15ra | 0.206 |
| ksr1 | 0.206 |
| slk | 0.205 |
| gulp1 | 0.205 |
| dpp8 | 0.205 |
| fzd4 | 0.205 |
| tln2 | 0.205 |
| dvl2 | 0.205 |
| camk1d | 0.205 |
| tnk1 | 0.205 |
| gigyf1 | 0.205 |
| dok5 | 0.205 |
| tax1bp1 | 0.205 |
| bcl11b | 0.205 |
| klf13 | 0.205 |
| tank | 0.205 |
| ticam1 | 0.204 |
| klhl6 | 0.204 |
| tnfaip8 | 0.204 |
| akt3 | 0.204 |
| siglec15 | 0.204 |
| 100043861 | 0.204 |
| rgs7bp | 0.204 |
| stat2 | 0.204 |
| tbk1 | 0.204 |
| smpd2 | 0.204 |
| cd6 | 0.204 |
| msn | 0.204 |
| il1rap | 0.204 |
| mapk10 | 0.204 |
| trim8 | 0.204 |
| itgb3bp | 0.203 |
| grap2 | 0.203 |
| tcf7 | 0.203 |
| fcrl6 | 0.203 |
| depdc6 | 0.203 |
| arhgef15 | 0.203 |
| map2k3 | 0.203 |
| tsc22d3 | 0.203 |
| gpr20 | 0.203 |
| litaf | 0.203 |
| ccdc50 | 0.203 |
| relb | 0.203 |
| cd97 | 0.203 |
| dkk2 | 0.203 |
| il9r | 0.203 |
| cdh22 | 0.203 |
| nlrp2 | 0.203 |
| asb6 | 0.202 |
| avil | 0.202 |
| smok2a | 0.202 |
| il1rapl2 | 0.202 |
| gpr156 | 0.202 |
| il1rl1 | 0.202 |
| etv3 | 0.202 |
| 2310008h04rik | 0.202 |
| lilrb4 | 0.202 |
| traf5 | 0.202 |
| cd19 | 0.202 |
| sdc4 | 0.202 |
| ottmusg00000008540 | 0.201 |
| pag1 | 0.201 |
| nrarp | 0.201 |
| magi3 | 0.201 |
| il17c | 0.201 |
| map3k7ip1 | 0.201 |
| mst1 | 0.201 |
| mapkapk2 | 0.201 |
| gpr22 | 0.201 |
| dvl3 | 0.201 |
| sh3bp2 | 0.201 |
| stab1 | 0.201 |
| rasgrp3 | 0.201 |
| cnpy4 | 0.201 |
| s100a7a | 0.201 |
| cd163l1 | 0.201 |
| spry3 | 0.201 |
| sdcbp | 0.2 |
| ensmusg00000071552 | 0.2 |
| selplg | 0.2 |
| amica1 | 0.2 |
| fbxw5 | 0.2 |
| itch | 0.2 |
| rap1gap | 0.2 |
| il3ra | 0.2 |
| ysk4 | 0.2 |
| ubg | 0.2 |
| nod1 | 0.2 |
| rspo1 | 0.2 |
| lag3 | 0.2 |
| il11ra2 | 0.2 |
| mrc2 | 0.2 |
| aebp1 | 0.2 |
| dtx1 | 0.2 |
| ralb | 0.2 |
| slamf1 | 0.2 |
| vangl1 | 0.199 |
| fer | 0.199 |
| nrbp1 | 0.199 |
| def6 | 0.199 |
| frs3 | 0.199 |
| lrp4 | 0.199 |
| rapgefl1 | 0.199 |
| igtp | 0.199 |
| prokr1 | 0.199 |
| rqcd1 | 0.198 |
| rgs11 | 0.198 |
| nenf | 0.198 |
| prkd3 | 0.198 |
| cd164 | 0.198 |
| grlf1 | 0.198 |
| tnik | 0.198 |
| traf4 | 0.198 |
| wsb2 | 0.198 |
| wnt9b | 0.198 |
| ebi3 | 0.198 |
| fzd3 | 0.198 |
| d11mit331 | 0.198 |
| inpp5d | 0.198 |
| rgs2 | 0.198 |
| map4k3 | 0.198 |
| spry4 | 0.198 |
| sh3bp5 | 0.198 |
| ryk | 0.198 |
| gpsm1 | 0.198 |
| dact3 | 0.198 |
| mapk4 | 0.197 |
| plcb3 | 0.197 |
| lrrc2 | 0.197 |
| ptprcap | 0.197 |
| itgad | 0.197 |
| mapk11 | 0.197 |
| d2mit281 | 0.197 |
| cigs1 | 0.197 |
| ulk2 | 0.197 |
| nkrf | 0.197 |
| gde1 | 0.197 |
| socs1 | 0.197 |
| loc627060 | 0.197 |
| ryk-rs1 | 0.197 |
| ripk5 | 0.197 |
| crim2 | 0.196 |
| olfr73 | 0.196 |
| dnajc14 | 0.196 |
| siglech | 0.196 |
| spopl | 0.196 |
| ott | 0.196 |
| smpd3 | 0.196 |
| smpd4 | 0.196 |
| dusp6 | 0.196 |
| fut7 | 0.196 |
| cd200 | 0.196 |
| gna11 | 0.196 |
| lect2 | 0.196 |
| ric8b | 0.195 |
| irf4 | 0.195 |
| cmtm3 | 0.195 |
| dixdc1 | 0.195 |
| gps2 | 0.195 |
| phlpp | 0.195 |
| d8mit155 | 0.195 |
| dusp22 | 0.195 |
| arhgap5 | 0.195 |
| nkx2-3 | 0.195 |
| cish | 0.195 |
| cd209b | 0.195 |
| jub | 0.195 |
| akap13 | 0.194 |
| wnt5a | 0.194 |
| mmd | 0.194 |
| nedd9 | 0.194 |
| arhgap26 | 0.194 |
| jam2 | 0.194 |
| chst4 | 0.194 |
| siglec5 | 0.194 |
| memo1 | 0.194 |
| rgs7 | 0.194 |
| appl1 | 0.194 |
| 1110006o17rik | 0.194 |
| eg383927 | 0.194 |
| gpr63 | 0.194 |
| abl2 | 0.193 |
| cd72 | 0.193 |
| tg(erbb2)1jek | 0.193 |
| oxgr1 | 0.193 |
| tg(il1a)1.1tsk | 0.193 |
| iigp2 | 0.193 |
| usp14 | 0.193 |
| intu | 0.193 |
| fuz | 0.193 |
| disp2 | 0.193 |
| clec1b | 0.193 |
| cd200r4 | 0.193 |
| cxcr4 | 0.193 |
| zfp46 | 0.193 |
| wnt11 | 0.193 |
| wnt2b | 0.193 |
| elmo1 | 0.193 |
| sgpl1 | 0.193 |
| ikbke | 0.193 |
| ror1 | 0.193 |
| rap1b | 0.193 |
| pglyrp4 | 0.193 |
| vmn2r122 | 0.193 |
| l1md-a5 | 0.193 |
| itgal | 0.192 |
| fcer1a | 0.192 |
| il1f6 | 0.192 |
| itga4 | 0.192 |
| mapk8ip2 | 0.192 |
| rin1 | 0.192 |
| dusp4 | 0.192 |
| tnfrsf19 | 0.192 |
| tbx21 | 0.192 |
| ilkap | 0.192 |
| gnaq | 0.192 |
| cyld | 0.192 |
| grem2 | 0.192 |
| lgr5 | 0.192 |
| olfr410 | 0.192 |
| gpr126 | 0.192 |
| tg(il1a)1.2tsk | 0.191 |
| zfyve9 | 0.191 |
| ifnab | 0.191 |
| lrp6 | 0.191 |
| gpr151 | 0.191 |
| paqr3 | 0.191 |
| elf4 | 0.191 |
| vav2 | 0.191 |
| myo18a | 0.191 |
| mprip | 0.191 |
| taok2 | 0.191 |
| epha1 | 0.19 |
| pygo1 | 0.19 |
| rps6ka5 | 0.19 |
| lins2 | 0.19 |
| coro1a | 0.19 |
| pick2 | 0.19 |
| pick5 | 0.19 |
| pick3 | 0.19 |
| pick4 | 0.19 |
| cebpe | 0.19 |
| rxfp4 | 0.19 |
| d6mit296 | 0.19 |
| d11mit215 | 0.19 |
| tas2r136 | 0.19 |
| il1f8 | 0.19 |
| cc2d1b | 0.19 |
| wnt8a | 0.19 |
| il21r | 0.19 |
| rb1cc1 | 0.19 |
| stk17b | 0.19 |
| afap1 | 0.19 |
| ptpn7 | 0.19 |
| gprin1 | 0.19 |
| gbp2 | 0.19 |
| ranbp10 | 0.19 |
| dub1 | 0.19 |
| ly86 | 0.19 |
| pak1ip1 | 0.19 |
| rgnef | 0.189 |
| wdr1 | 0.189 |
| tns3 | 0.189 |
| gpr120 | 0.189 |
| rapgef3 | 0.189 |
| cysltr2 | 0.189 |
| rp23-157o10.7 | 0.189 |
| cblc | 0.189 |
| zfp322a | 0.189 |
| icos | 0.189 |
| cd74 | 0.189 |
| olfr63 | 0.189 |
| vav3 | 0.189 |
| gper | 0.189 |
| cd27 | 0.189 |
| igfbp7 | 0.189 |
| 1700009n14rik | 0.189 |
| nck2 | 0.189 |
| nlrx1 | 0.189 |
| map3k11 | 0.189 |
| olfr71 | 0.188 |
| sphkap | 0.188 |
| gpr110 | 0.188 |
| olfr544 | 0.188 |
| il21 | 0.188 |
| ddr1 | 0.188 |
| dub2 | 0.188 |
| gab1 | 0.188 |
| tg(notch1)a5rko | 0.188 |
| tg(notch1)a7rko | 0.188 |
| kremen1 | 0.188 |
| cd300lg | 0.188 |
| pglyrp3 | 0.188 |
| d10ertd610e | 0.188 |
| gpr87 | 0.188 |
| pea15b | 0.187 |
| gprin2 | 0.187 |
| sla | 0.187 |
| thoc5 | 0.187 |
| btla | 0.187 |
| otud5 | 0.187 |
| v2r1 | 0.187 |
| vmn2r3 | 0.187 |
| fermt3 | 0.187 |
| spsb2 | 0.187 |
| clec12a | 0.187 |
| tg(krt5-cre)1tak | 0.187 |
| racgap1 | 0.187 |
| pear1 | 0.187 |
| bc | 0.187 |
| plcb2 | 0.187 |
| epha8 | 0.187 |
| traf3ip1 | 0.187 |
| lrrn3 | 0.187 |
| flt3l | 0.186 |
| 6330417g02rik | 0.186 |
| olfr3 | 0.186 |
| olfr1310 | 0.186 |
| ifitm3 | 0.186 |
| cd38 | 0.186 |
| rasa4 | 0.186 |
| git2 | 0.186 |
| csf3 | 0.186 |
| itgax | 0.186 |
| akt1s1 | 0.186 |
| cd160 | 0.186 |
| dgkz | 0.186 |
| rspo4 | 0.186 |
| zfand6 | 0.186 |
| sp9 | 0.186 |
| stk24 | 0.186 |
| v1rf2 | 0.186 |
| v1rf3 | 0.186 |
| d16mit73 | 0.186 |
| fzd9 | 0.186 |
| fcr | 0.186 |
| il1f9 | 0.186 |
| ottmusg00000005723 | 0.186 |
| cxxc5 | 0.186 |
| apold1 | 0.186 |
| maml3 | 0.185 |
| traf6 | 0.185 |
| rgs9bp | 0.185 |
| nle1 | 0.185 |
| mapk8ip3 | 0.185 |
| akap12 | 0.185 |
| mast3 | 0.185 |
| 1110018j18rik | 0.185 |
| asc1 | 0.185 |
| cnot8 | 0.185 |
| irf7 | 0.185 |
| d3ertd302e | 0.185 |
| irf8 | 0.185 |
| sema6d | 0.185 |
| vasn | 0.185 |
| cd276 | 0.185 |
| brap | 0.185 |
| il9 | 0.185 |
| centg3 | 0.185 |
| adarb2 | 0.184 |
| cd22 | 0.184 |
| lphn1 | 0.184 |
| arhgef11 | 0.184 |
| tg(ctnnb1)1efu | 0.184 |
| enpp2 | 0.184 |
| jakmip1 | 0.184 |
| irs4 | 0.184 |
| d16mit15 | 0.184 |
| d6mit328 | 0.184 |
| rit1 | 0.184 |
| 1200009o22rik | 0.184 |
| tlr9 | 0.184 |
| bcl2l12 | 0.184 |
| gpr37l1 | 0.184 |
| mrgprh | 0.184 |
| mast2 | 0.184 |
| dock1 | 0.184 |
| dgkb | 0.184 |
| wnt7b | 0.184 |
| rras2 | 0.184 |
| tbl1xr1 | 0.184 |
| nr4a3 | 0.183 |
