# Supplementary material for: GIT2 Acts as a Potential Keystone Protein in Functional Hypothalamic Networks Associated with Age-Related Phenotypic Changes in Rats
Source: PLoS One. 2012 May 14;7(5):e36975. doi: 10.1371/journal.pone.0036975 (PMC3351446; doi:10.1371/journal.pone.0036975)
Supplement: Table S10 — GeneIndexer latent semantic indexing (LSI) of significantly-regulated ‘Alzheimer's disease’ KEGG pathway. Using the KEGG signaling pathway ‘Alzheimer's disease’ as an input term, a list of the top 1000 implicitly-correlated (LSI correlation score >0.1) was generated using a full genome background list. (DOC) [file pone.0036975.s014.doc]

**Table S10. GeneIndexer latent semantic indexing (LSI) of significantly-regulated ‘Alzheimer’s disease’ KEGG pathway.** Using the KEGG signaling pathway ‘Alzheimer’s disease’ as an input term, a list of the top 1000 implicitly-correlated (LSI correlation score >0.1) was generated using a full genome background list.

| ***Alzheimer's disease*** |  |
| --- | --- |
|  |  |
| **Protein Symbol** | **LSI correlation score** |
| sfrs12ip1 | 0.772 |
| mirn106b | 0.767 |
| 2810048g17rik | 0.759 |
| gapdh-ps13 | 0.682 |
| azdm3 | 0.676 |
| appd2 | 0.676 |
| appd1 | 0.676 |
| col25a1 | 0.665 |
| mirn29a | 0.663 |
| lrrtm3 | 0.655 |
| tm2d2 | 0.647 |
| tg(apoe)1vln | 0.642 |
| dock3 | 0.61 |
| pid2 | 0.607 |
| d16h21s52 | 0.6 |
| bhlhb9 | 0.599 |
| apbb3 | 0.596 |
| itm2b | 0.588 |
| zfand2b | 0.586 |
| bc039210 | 0.571 |
| clstn2 | 0.565 |
| bace2 | 0.563 |
| d9mit285 | 0.552 |
| tm2d1 | 0.552 |
| tauph | 0.541 |
| bri3 | 0.536 |
| 4933407p14rik | 0.525 |
| apbb2 | 0.521 |
| mettl2 | 0.521 |
| atp8b4 | 0.517 |
| utp11l | 0.516 |
| tm2d3 | 0.514 |
| rnf146 | 0.513 |
| hectd2 | 0.511 |
| sorl1 | 0.507 |
| clstn3 | 0.503 |
| clstn1 | 0.497 |
| slc30a6 | 0.496 |
| gbrln | 0.495 |
| mirn17 | 0.493 |
| ch25h | 0.482 |
| aph1b | 0.481 |
| mnotch | 0.481 |
| cyp46a1 | 0.475 |
| apba2 | 0.472 |
| aplp1 | 0.47 |
| aph1c | 0.467 |
| bace1 | 0.465 |
| mirn20a | 0.463 |
| mirn29b-1 | 0.463 |
| ece2 | 0.461 |
| snx33 | 0.459 |
| ttbk2 | 0.456 |
| dnmbp | 0.455 |
| ormdl1 | 0.454 |
| bc038822 | 0.452 |
| 3110056o03rik | 0.451 |
| alph1 | 0.442 |
| apbb1 | 0.44 |
| aph1a | 0.437 |
| d7mit150 | 0.433 |
| d7mit222 | 0.433 |
| psenen | 0.433 |
| ubqln1 | 0.433 |
| 1810043g02rik | 0.43 |
| aplp2 | 0.423 |
| ptpla | 0.421 |
| necab3 | 0.42 |
| d17h21s56 | 0.417 |
| ncstn | 0.417 |
| sppl3 | 0.415 |
| rtn3 | 0.414 |
| ttbk1 | 0.413 |
| eg622509 | 0.412 |
| 1700081l11rik | 0.409 |
| anks1b | 0.406 |
| blmh | 0.406 |
| adamts17 | 0.402 |
| lrrc15 | 0.397 |
| apba3 | 0.396 |
| dhcr24 | 0.395 |
| sh3pxd2a | 0.393 |
| tapbpl | 0.387 |
| tmed10 | 0.386 |
| rabgap1l | 0.382 |
| ide | 0.381 |
| 2700078k21rik | 0.379 |
| tomm40 | 0.379 |
| d7mit91 | 0.376 |
| 2010106g01rik | 0.374 |
| klc1 | 0.373 |
| appbp2 | 0.371 |
| apba1 | 0.369 |
| lgi3 | 0.366 |
| wac | 0.362 |
| acad8 | 0.36 |
| hsd17b10 | 0.355 |
| nae1 | 0.355 |
| psmd8 | 0.355 |
| ctnna3 | 0.35 |
| snx17 | 0.346 |
| tagap | 0.346 |
| pank4 | 0.345 |
| mapk8ip1 | 0.341 |
| atp5j2 | 0.34 |
| pank3 | 0.339 |
| rer1 | 0.339 |
| tardbp | 0.338 |
| spon1 | 0.335 |
| dlst | 0.334 |
| aatf | 0.332 |
| fibrs1 | 0.331 |
| fibrs2 | 0.331 |
| fibrs3 | 0.331 |
| cml4 | 0.33 |
| apod | 0.328 |
| cdkl1 | 0.324 |
| d5mit151 | 0.323 |
| d5mit110 | 0.323 |
| slc30a7 | 0.323 |
| vps26a | 0.321 |
| nptxr | 0.321 |
| thop1 | 0.321 |
| gnd | 0.32 |
| sncb | 0.32 |
| abca2 | 0.32 |
| itm2a | 0.318 |
| h13 | 0.316 |
| t(16c3-4;17a2)65dn | 0.315 |
| mme | 0.315 |
| serpini1 | 0.314 |
| psen2 | 0.314 |
| pin1 | 0.313 |
| nptx1 | 0.31 |
| qser1 | 0.305 |
| cox10 | 0.305 |
| atp13a2 | 0.304 |
| slc30a3 | 0.302 |
| napsa | 0.3 |
| pank2 | 0.3 |
| a2m | 0.298 |
| serpina3n | 0.298 |
| klk6 | 0.297 |
| hadhb | 0.296 |
| bptf | 0.294 |
| ncln | 0.293 |
| sorcs1 | 0.292 |
| golm1 | 0.29 |
| grinl1a | 0.29 |
| cdcp2 | 0.29 |
| cbara1 | 0.288 |
| dyrk1a | 0.286 |
| pmv32 | 0.286 |
| mpmv17 | 0.286 |
| slc30a1 | 0.281 |
| tppp | 0.281 |
| atp8 | 0.281 |
| slc30a5 | 0.279 |
| slc30a4 | 0.278 |
| tbl3 | 0.278 |
| sncaip | 0.278 |
| chmp2b | 0.278 |
| bicc1 | 0.278 |
| nxnl1 | 0.277 |
| htra2 | 0.277 |
| wwc1 | 0.276 |
| fpr2 | 0.276 |
| sdf2l1 | 0.276 |
| pitrm1 | 0.276 |
| ece1 | 0.276 |
| ctsd | 0.275 |
| usp24 | 0.274 |
| apcs | 0.274 |
| lrpap1 | 0.273 |
| klhl7 | 0.273 |
| d16mit140 | 0.272 |
| tmem108 | 0.271 |
| d7mit281 | 0.271 |
| d16h21s16 | 0.27 |
| fggy | 0.27 |
| mark1 | 0.27 |
| gigyf2 | 0.269 |
| selm | 0.269 |
| eae12 | 0.265 |
| eae11 | 0.265 |
| eae4 | 0.265 |
| eae7 | 0.265 |
| eae13 | 0.265 |
| cdk5r1 | 0.264 |
| cst3 | 0.264 |
| bche | 0.264 |
| d18mit164 | 0.262 |
| pawr | 0.262 |
| gga1 | 0.261 |
| tet1 | 0.261 |
| mrpl10 | 0.261 |
| tcfcp2 | 0.26 |
| ottmusg00000007332 | 0.26 |
| ubxd2 | 0.258 |
| gsto2 | 0.258 |
| padi2 | 0.257 |
| herpud1 | 0.257 |
| mnek1c | 0.257 |
| mnek1a | 0.257 |
| mnek1b | 0.257 |
| lgh | 0.256 |
| d13mit249 | 0.256 |
| zfp294 | 0.255 |
| mapk8ip2 | 0.254 |
| slc7a11 | 0.254 |
| lipa | 0.254 |
| rcan1 | 0.253 |
| dpysl2 | 0.253 |
| kcnip3 | 0.251 |
| eae2 | 0.25 |
| flot2 | 0.25 |
| adam10 | 0.249 |
| dmrtb1 | 0.249 |
| d10mit199 | 0.248 |
| d10mit200 | 0.248 |
| drp2 | 0.248 |
| t(2;10)67gso | 0.248 |
| astn2 | 0.247 |
| abca12 | 0.245 |
| ube2k | 0.245 |
| trim11 | 0.245 |
| xpnpep2 | 0.244 |
| aw209491 | 0.244 |
| mrpl32 | 0.244 |
| pgk1-ps1 | 0.244 |
| speg | 0.244 |
| prrg4 | 0.244 |
| dars2 | 0.244 |
| nucb1 | 0.244 |
| tppp3 | 0.243 |
| dld | 0.242 |
| cyp2g1 | 0.242 |
| pctk3 | 0.242 |
| d19mit65 | 0.242 |
| ube2m | 0.241 |
| gapdh | 0.24 |
| d11mit262 | 0.239 |
| tmevd5 | 0.239 |
| psrs1 | 0.239 |
| cdk5 | 0.239 |
| tnfaip1 | 0.239 |
| crb2 | 0.239 |
| lrp8 | 0.239 |
| saa-ps | 0.238 |
| d18mit110 | 0.237 |
| homer3 | 0.237 |
| rtn2 | 0.237 |
| gsto1 | 0.237 |
| soat1 | 0.236 |
| tg(apoc1)1lmh | 0.236 |
| apoc1 | 0.236 |
| ottmusg00000005148 | 0.236 |
| chat | 0.236 |
| ndufb8 | 0.235 |
| cntn3 | 0.235 |
| rb(16.17)32lub | 0.235 |
| rb(16.17)32lub | 0.235 |
| d18mit65 | 0.234 |
| chgb | 0.233 |
| d18mit66 | 0.233 |
| lingo1 | 0.232 |
| becn1 | 0.232 |
| pcmt1 | 0.232 |
| epm2a | 0.232 |
| d11mit245 | 0.232 |
| 4-Sep | 0.231 |
| klhl1as | 0.231 |
| pnma5 | 0.23 |
| rtn4r | 0.23 |
| nomo1 | 0.23 |
| synpr | 0.229 |
| 3-Sep | 0.229 |
| vps13a | 0.228 |
| d16mit58 | 0.228 |
| opa3 | 0.228 |
| tdo2 | 0.228 |
| sqstm1 | 0.227 |
| olfr222 | 0.227 |
| ppp1r10 | 0.227 |
| rencd | 0.227 |
| mptp1 | 0.226 |
| htr6 | 0.226 |
| trav8d-1 | 0.226 |
| grn | 0.226 |
| uchl1 | 0.225 |
| adnp | 0.225 |
| psmc6 | 0.225 |
| gan | 0.225 |
| slc5a7 | 0.225 |
| efhd2 | 0.225 |
| mopkd1 | 0.225 |
| mopkd2 | 0.225 |
| cpy | 0.225 |
| hrtfm1 | 0.224 |
| hrtfm2 | 0.224 |
| xmv2 | 0.224 |
| kctd7 | 0.224 |
| eae8 | 0.224 |
| ttpa | 0.224 |
| phkg1 | 0.223 |
| rnf19a | 0.222 |
| nhlrc1 | 0.222 |
| anks6 | 0.222 |
| ubxd8 | 0.221 |
| rb(6.16)24lub | 0.221 |
| rb(6.16)24lub | 0.221 |
| gmppb | 0.221 |
| homer2 | 0.221 |
| dbn1 | 0.221 |
| d230025d16rik | 0.221 |
| spast | 0.221 |
| 4921504e06rik | 0.22 |
| 4930579e17rik | 0.22 |
| d18mit146 | 0.22 |
| amigo2 | 0.22 |
| lrrk1 | 0.219 |
| hspb8 | 0.219 |
| kif11 | 0.219 |
| ina | 0.219 |
| exf | 0.218 |
| hpcal1 | 0.218 |
| mt3 | 0.218 |
| dpml | 0.218 |
| rtn4 | 0.217 |
| olr1 | 0.217 |
| nd1 | 0.217 |
| abcg1 | 0.216 |
| eno1 | 0.216 |
| atxn2 | 0.216 |
| gsk3a | 0.216 |
| casp6 | 0.215 |
| d10mit178 | 0.215 |
| loc100046187 | 0.214 |
| setx | 0.214 |
| rmcs5 | 0.214 |
| rb(16.17)7bnr | 0.214 |
| jckm2 | 0.214 |
| rb(16.17)7bnr | 0.214 |
| bc023892 | 0.213 |
| lrp1 | 0.213 |
| lrp1b | 0.213 |
| ranbp9 | 0.212 |
| d10mit46 | 0.212 |
| npc2 | 0.211 |
| rtn1 | 0.211 |
| tnfrsf21 | 0.211 |
| prdm10 | 0.211 |
| rnf103 | 0.211 |
| nucb2 | 0.211 |
| centb5 | 0.21 |
| gvhd4 | 0.21 |
| d4mit226 | 0.21 |
| gvhd5 | 0.21 |
| csnk1d | 0.21 |
| atxn10 | 0.21 |
| d10mit102 | 0.21 |
| yaa2 | 0.21 |
| yaa3 | 0.21 |
| yaa4 | 0.21 |
| cox3 | 0.209 |
| gga3 | 0.209 |
| sncg | 0.209 |
| spon2 | 0.209 |
| park7 | 0.209 |
| atxn7 | 0.209 |
| trpc4ap | 0.209 |
| acad11 | 0.208 |
| tgm1 | 0.208 |
| naaa | 0.208 |
| 2210016l21rik | 0.208 |
| nbwa1 | 0.208 |
| nbwa2 | 0.208 |
| 668253 | 0.208 |
| tmem90a | 0.208 |
| adamts10 | 0.208 |
| aw121567 | 0.208 |
| spg7 | 0.207 |
| zfp750 | 0.207 |
| lrrk2 | 0.207 |
| gbe1 | 0.207 |
| spg3a | 0.207 |
| chrna7 | 0.207 |
| foxo3 | 0.207 |
| fhdc1 | 0.207 |
| 5330431n19rik | 0.207 |
| elsgp4 | 0.206 |
| jckm1 | 0.206 |
| d10mit164 | 0.206 |
| d7mit261 | 0.206 |
| clu | 0.206 |
| sar1b | 0.206 |
| vldlr | 0.206 |
| bc037112 | 0.205 |
| loc627060 | 0.205 |
| als2 | 0.205 |
| hs1bp3 | 0.205 |
| atxn1l | 0.205 |
| d4mit261 | 0.204 |
| 6030443o07rik | 0.204 |
| cln5 | 0.204 |
| renf2 | 0.204 |
| gpc1 | 0.204 |
| atn1 | 0.204 |
| htatip | 0.204 |
| rb(11.16)2h | 0.204 |
| rb(11.16)2h | 0.204 |
| 4932414j04rik | 0.203 |
| d10mit96 | 0.203 |
| d10mit135 | 0.203 |
| timm8b | 0.203 |
| atxn3 | 0.203 |
| nba3 | 0.203 |
| xmv26 | 0.203 |
| cln8 | 0.203 |
| mfsd8 | 0.202 |
| sacs | 0.202 |
| abcd1 | 0.202 |
| numb | 0.202 |
| pcsk1n | 0.202 |
| pacrg | 0.201 |
| ttr | 0.201 |
| alg5 | 0.201 |
| cc2d2a | 0.201 |
| glo1 | 0.201 |
| mmachc | 0.201 |
| syp | 0.201 |
| rpgrip1l | 0.201 |
| crbn | 0.2 |
| ndufv1 | 0.2 |
| tmevd2 | 0.2 |
| otud1 | 0.2 |
| d11mit116 | 0.2 |
| d19mit11.1 | 0.2 |
| jph3 | 0.2 |
| atp2c1 | 0.2 |
| gne | 0.2 |
| eae26 | 0.199 |
| lmr13 | 0.199 |
| lmr14 | 0.199 |
| lmr15 | 0.199 |
| d2mit283 | 0.199 |
| nus1 | 0.199 |
| cntn5 | 0.199 |
| bbs7 | 0.199 |
| npc1 | 0.199 |
| itm2c | 0.199 |
| mapk10 | 0.198 |
| zfyve26 | 0.198 |
| dbndd2 | 0.198 |
| ldlrap1 | 0.198 |
| tmevd3 | 0.198 |
| tmevd4 | 0.198 |
| d10mit70 | 0.198 |
| kif1a | 0.198 |
| apof | 0.197 |
| 1-Sep | 0.197 |
| rpl36 | 0.197 |
| rabep1 | 0.197 |
| usp14 | 0.196 |
| renf1 | 0.196 |
| slco2a1 | 0.196 |
| hspa13 | 0.196 |
| mlrrq | 0.196 |
| gdap1 | 0.196 |
| priap1 | 0.196 |
| l2hgdh | 0.196 |
| gm967 | 0.196 |
| myocd | 0.195 |
| d2mit51 | 0.195 |
| apon | 0.195 |
| trem2 | 0.195 |
| serf2 | 0.195 |
| a230106n23rik | 0.195 |
| gcdh | 0.195 |
| rends | 0.194 |
| bbs5 | 0.194 |
| aoc3 | 0.194 |
| numbl | 0.194 |
| st6gal1 | 0.194 |
| iapp | 0.194 |
| ak7 | 0.194 |
| ubqln2 | 0.194 |
| ccdc28b | 0.194 |
| wld | 0.194 |
| d10mit114 | 0.194 |
| picalm | 0.193 |
| ndufb3 | 0.193 |
| d7mit40 | 0.193 |
| slc27a2 | 0.193 |
| spink5 | 0.193 |
| cln6 | 0.193 |
| wasf1 | 0.193 |
| ltnr1 | 0.192 |
| ltnr2 | 0.192 |
| cdk5r2 | 0.192 |
| apoc2 | 0.192 |
| ngb | 0.192 |
| mgat3 | 0.192 |
| wrb | 0.192 |
| dab1 | 0.192 |
| gm22 | 0.191 |
| ndufa11 | 0.191 |
| slc17a5 | 0.191 |
| ccs | 0.191 |
| fbxw7 | 0.191 |
| stub1 | 0.191 |
| serf2-ps | 0.191 |
| sgsh | 0.191 |
| naip1 | 0.191 |
| ll | 0.191 |
| sunc1 | 0.191 |
| sod1m | 0.191 |
| bcl7b | 0.19 |
| d10mit54 | 0.19 |
| d10mit255 | 0.19 |
| ndufv2 | 0.19 |
| plekhg4 | 0.19 |
| mlc1 | 0.19 |
| hmgcs2 | 0.19 |
| abcg4 | 0.19 |
| btbd9 | 0.189 |
| manba | 0.189 |
| gmfb | 0.189 |
| rb(12.14)8rma | 0.189 |
| pdxp | 0.189 |
| usp40 | 0.189 |
| bc023814 | 0.189 |
| cmn3 | 0.189 |
| cmn2 | 0.189 |
| cmn1 | 0.189 |
| psmb5 | 0.188 |
| ckds | 0.188 |
| ncapd2 | 0.188 |
| reep1 | 0.188 |
| nptx2 | 0.188 |
| cirh1a | 0.188 |
| tfam | 0.188 |
| tmem67 | 0.188 |
| d10mit174 | 0.187 |
| mpdk2 | 0.187 |
| mpdk6 | 0.187 |
| mpdk8 | 0.187 |
| mpdk1 | 0.187 |
| mpdk5 | 0.187 |
| mpdk7 | 0.187 |
| mpdk3 | 0.187 |
| mpdk4 | 0.187 |
| gvhd2 | 0.187 |
| gvhd1 | 0.187 |
| efhc1 | 0.187 |
| loc677222 | 0.187 |
| ahi1 | 0.187 |
| odam | 0.187 |
| serf1 | 0.187 |
| neu1 | 0.187 |
| rmcs2 | 0.187 |
| nd5 | 0.187 |
| amigo1 | 0.187 |
| amigo3 | 0.187 |
| ttc12 | 0.186 |
| klk7 | 0.186 |
| d7mit98 | 0.186 |
| ptpn5 | 0.186 |
| mtch1 | 0.186 |
| prdt3 | 0.186 |
| klhl1 | 0.186 |
| kcnc4 | 0.186 |
| park2 | 0.185 |
| saa2 | 0.185 |
| pdpv | 0.185 |
| hap1 | 0.185 |
| cndp2 | 0.185 |
| ppp2r4 | 0.184 |
| ubiad1 | 0.184 |
| gvhd3 | 0.184 |
| nphp1 | 0.184 |
| laq1 | 0.184 |
| 2-Sep | 0.184 |
| ctnnd2 | 0.184 |
| klc2 | 0.184 |
| nphp4 | 0.184 |
| il10rb | 0.184 |
| gt | 0.183 |
| eae24 | 0.183 |
| idd9.2 | 0.183 |
| ple | 0.182 |
| bbs2 | 0.182 |
| psmc1 | 0.182 |
| 9930022n03rik | 0.182 |
| zc3h15 | 0.182 |
| vas1 | 0.182 |
| orch6 | 0.182 |
| epd1 | 0.182 |
| d16mit79 | 0.182 |
| d16mit130 | 0.182 |
| d11mit177 | 0.182 |
| abbp4 | 0.182 |
| hmgn1-rs7 | 0.182 |
| scat | 0.182 |
| bbs10 | 0.182 |
| dnajb2 | 0.182 |
| eif2b2 | 0.181 |
| d19mit63 | 0.181 |
| d10mit140 | 0.181 |
| atp6 | 0.181 |
| clic1 | 0.181 |
| gba | 0.181 |
| aaas | 0.181 |
| cln3 | 0.181 |
| sel1l | 0.181 |
| peo1 | 0.18 |
| cd5l | 0.18 |
| pank1 | 0.18 |
| saa4 | 0.18 |
| cox11 | 0.18 |
| ubb | 0.18 |
| mwfh | 0.18 |
| sec63 | 0.18 |
| d8mit100 | 0.18 |
| fgf20 | 0.18 |
| wl | 0.18 |
| psen1 | 0.179 |
| nd3 | 0.179 |
| gsn | 0.179 |
| naglu | 0.179 |
| xk | 0.179 |
| d14mit66 | 0.179 |
| abd1 | 0.179 |
| gm1123 | 0.179 |
| eef2k | 0.179 |
| ilvbl | 0.179 |
| aabpr | 0.179 |
| d17mit115 | 0.179 |
| tmc6 | 0.179 |
| naaladl2 | 0.179 |
| htr4 | 0.178 |
| spink4 | 0.178 |
| rdh14 | 0.178 |
| d7mit227 | 0.178 |
| nphp3 | 0.178 |
| xpnpep3 | 0.178 |
| nba1 | 0.178 |
| c4bp | 0.178 |
| hyls1 | 0.178 |
| ppt1 | 0.178 |
| b830045n13rik | 0.178 |
| capn1 | 0.178 |
| vcp | 0.178 |
| 2310035c23rik | 0.178 |
| hspa12a | 0.178 |
| cyp4f39 | 0.178 |
| sials | 0.178 |
| ndufs3 | 0.178 |
| eae3 | 0.178 |
| crlf1 | 0.178 |
| ireb2 | 0.177 |
| aadat | 0.177 |
| cbs | 0.177 |
| ndufa12 | 0.177 |
| dxmod | 0.177 |
| dxnph | 0.177 |
| epi | 0.177 |
| jb | 0.177 |
| bag1 | 0.177 |
| pnma1 | 0.177 |
| d15mit100 | 0.177 |
| nckap1 | 0.177 |
| gga2 | 0.177 |
| phyhip | 0.177 |
| c1r | 0.177 |
| cia8 | 0.177 |
| bbs1 | 0.176 |
| scrg1 | 0.176 |
| shc3 | 0.176 |
| eg260347 | 0.176 |
| cyp4v3 | 0.176 |
| saa1 | 0.176 |
| spon1l | 0.176 |
| gasa2 | 0.176 |
| gasa1 | 0.176 |
| t(12;16)1cje | 0.176 |
| ddit4 | 0.176 |
| agln1 | 0.175 |
| elovl4 | 0.175 |
| ctse | 0.175 |
| bat1a | 0.175 |
| clpp | 0.175 |
| rbm20 | 0.175 |
| s100a16 | 0.175 |
| apbb1ip | 0.175 |
| sil1 | 0.174 |
| coq9 | 0.174 |
| phb | 0.174 |
| nxnl2 | 0.174 |
| rb(16.17)8lub | 0.174 |
| rb(16.17)8lub | 0.174 |
| lix1 | 0.174 |
| nek8 | 0.174 |
| rapop4 | 0.174 |
| mott1 | 0.174 |
| d17mit60 | 0.174 |
| pink1 | 0.174 |
| 2810046l04rik | 0.174 |
| b230206f22rik | 0.174 |
| lsg1 | 0.174 |
| prkcsh | 0.174 |
| crb1 | 0.174 |
| luzp2 | 0.173 |
| dpp6 | 0.173 |
| prnd | 0.173 |
| ppt2 | 0.173 |
| slc4a11 | 0.173 |
| plekhh2 | 0.173 |
| bcs1l | 0.173 |
| agln3 | 0.173 |
| agln2 | 0.173 |
| agln4 | 0.173 |
| nd2 | 0.173 |
| atxn1 | 0.172 |
| cp | 0.172 |
| ache | 0.172 |
| cox1 | 0.172 |
| ctnnbip1 | 0.172 |
| pqbp1 | 0.172 |
| vsnl1 | 0.172 |
| rnaseh2c | 0.172 |
| enpep | 0.172 |
| cstb | 0.172 |
| psors1c2 | 0.171 |
| eg628705 | 0.171 |
| creld1 | 0.171 |
| cd68 | 0.171 |
| d16mit103 | 0.171 |
| icam5 | 0.171 |
| b9d1 | 0.171 |
| syvn1 | 0.171 |
| sh3tc1 | 0.171 |
| ppif | 0.171 |
| nradd | 0.171 |
| vps13c | 0.171 |
| prdx1 | 0.171 |
| d1nds1 | 0.171 |
| palm2-akap2 | 0.171 |
| kif5b | 0.171 |
| gpr37 | 0.171 |
| tmc8 | 0.17 |
| shpk | 0.17 |
| ttc8 | 0.17 |
| d2mit285 | 0.17 |
| d16mit64 | 0.17 |
| anxty | 0.17 |
| d4mit127 | 0.17 |
| krt24 | 0.17 |
| cntn4 | 0.17 |
| idd17 | 0.17 |
| d8mit231 | 0.17 |
| lxn | 0.17 |
| aldh16a1 | 0.17 |
| atg9a | 0.17 |
| pcdh19 | 0.169 |
| timm8a1 | 0.169 |
| cisd2 | 0.169 |
| ugcg | 0.169 |
| mks1 | 0.169 |
| h47 | 0.169 |
| cep290 | 0.169 |
| tor1b | 0.169 |
| tmevd1 | 0.169 |
| idd10 | 0.169 |
| pnma3 | 0.169 |
| reg2 | 0.168 |
| fri | 0.168 |
| mark2 | 0.168 |
| cox6c | 0.168 |
| mcoln1 | 0.168 |
| tbce | 0.168 |
| d5mit233 | 0.168 |
| saa | 0.168 |
| chrna4 | 0.168 |
| pkhd1l1 | 0.168 |
| del(7)tyr<c-3h>-p | 0.168 |
| mkks | 0.168 |
| lbt1 | 0.168 |
| d12mit11 | 0.168 |
| aod1 | 0.167 |
| ppp2r2b | 0.167 |
| evc2 | 0.167 |
| d11mit294 | 0.167 |
| sles4 | 0.167 |
| sles3 | 0.167 |
| atp5a1 | 0.167 |
| apom | 0.167 |
| tcra-v7 | 0.167 |
| cib1 | 0.167 |
| d15mit12 | 0.167 |
| igan4 | 0.167 |
| igan2 | 0.167 |
| igan1 | 0.167 |
| igan3 | 0.167 |
| d10mit179 | 0.167 |
| tmem43 | 0.167 |
| ndufs2 | 0.167 |
| aspa | 0.167 |
| sar | 0.167 |
| d2mit176 | 0.167 |
| d2mit83 | 0.167 |
| d2mit151 | 0.167 |
| rasgef1a | 0.166 |
| ctns | 0.166 |
| 5-Sep | 0.166 |
| nefh | 0.166 |
| sh3bgr | 0.166 |
| stk32b | 0.166 |
| tbl2 | 0.166 |
| zfp169 | 0.166 |
| lars2 | 0.166 |
| d10mit145 | 0.166 |
| d10mit71 | 0.166 |
| d5mit338 | 0.166 |
| d2hgdh | 0.166 |
| lmr5 | 0.166 |
| madd | 0.166 |
| clec16a | 0.166 |
| capsl | 0.165 |
| idd9.1 | 0.165 |
| s100b | 0.165 |
| hpt | 0.165 |
| nipa1 | 0.165 |
| rps6kb2 | 0.165 |
| dxhxs676 | 0.165 |
| ndufb4 | 0.165 |
| casp12 | 0.165 |
| tesb | 0.165 |
| iqcb1 | 0.165 |
| abcd2 | 0.164 |
| c1qa | 0.164 |
| d4mit70 | 0.164 |
| 6530404n21rik | 0.164 |
| mapt | 0.164 |
| 5730427n09rik | 0.164 |
| d3mit244 | 0.164 |
| 6430537h07rik | 0.164 |
| 4921509b22rik | 0.164 |
| chrna3 | 0.164 |
| hmgcr | 0.164 |
| derms | 0.164 |
| hspa9 | 0.164 |
| etfa | 0.164 |
| c1qb | 0.164 |
| dpysl5 | 0.164 |
| hspa12b | 0.164 |
| ighmbp2 | 0.164 |
| trp53bp2 | 0.164 |
| d5mit179 | 0.164 |
| d11mit122 | 0.164 |
| nnmt | 0.164 |
| slc1a2 | 0.164 |
| atcay | 0.164 |
| d3mit12 | 0.163 |
| mtap1b | 0.163 |
| maob | 0.163 |
| spm1 | 0.163 |
| aem3 | 0.163 |
| flad1 | 0.163 |
| ckb | 0.163 |
| coq7 | 0.163 |
| gnptab | 0.163 |
| rnaseh2b | 0.163 |
| hmgb1-rs6 | 0.163 |
| d13mit76 | 0.163 |
| htra1 | 0.163 |
| scyl1bp1 | 0.163 |
| mccc2 | 0.162 |
| eif2b5 | 0.162 |
| grk5 | 0.162 |
| fm | 0.162 |
| ofd1 | 0.162 |
| birc1-rs1 | 0.162 |
| kif2b | 0.162 |
| il1r2 | 0.162 |
| prdt1 | 0.162 |
| hgsnat | 0.162 |
| d14mit105 | 0.161 |
| sfxn5 | 0.161 |
| bbs4 | 0.161 |
| elsgp1 | 0.161 |
| cerkl | 0.161 |
| flnb | 0.161 |
| dgcr2 | 0.161 |
| zfp715 | 0.161 |
| tpp1 | 0.161 |
| pter | 0.161 |
| gasa3 | 0.161 |
| gasa4 | 0.161 |
| etfb | 0.161 |
| flot1 | 0.161 |
| phyh | 0.161 |
| ndufs7 | 0.161 |
| d11mit360 | 0.161 |
| kdsr | 0.161 |
| wars | 0.161 |
| klhdc8b | 0.16 |
| 4833418a01rik | 0.16 |
| capn5 | 0.16 |
| cfd | 0.16 |
| efemp1 | 0.16 |
| pnma2 | 0.16 |
| d18mit37 | 0.16 |
| d3mit75 | 0.16 |
| d3mit175 | 0.16 |
| fdps | 0.16 |
| dxmit117 | 0.16 |
| d9mit297 | 0.16 |
| gab2 | 0.16 |
| afg3l2 | 0.16 |
| pon3 | 0.16 |
| cia6 | 0.16 |
| nd6 | 0.16 |
| il31 | 0.16 |
| d17jcs98 | 0.159 |
| 4930506m07rik | 0.159 |
| c1qtnf5 | 0.159 |
| d11mit143 | 0.159 |
| ad | 0.159 |
| d10mit267 | 0.159 |
| tmem70 | 0.159 |
| pgia9 | 0.159 |
| pgia8 | 0.159 |
| ard1 | 0.159 |
| glul | 0.159 |
| wrmod1 | 0.159 |
| zdhhc8 | 0.159 |
| mccc1 | 0.159 |
| arpp19 | 0.159 |
| 3110002h16rik | 0.159 |
| d11mit179 | 0.158 |
| idd12 | 0.158 |
| d1mit538 | 0.158 |
| cri | 0.158 |
| etfdh | 0.158 |
| msra | 0.158 |
| d1mit45 | 0.158 |
| dopey2 | 0.158 |
| osbp2 | 0.158 |
| ftl1 | 0.158 |
| 1110038b12rik | 0.157 |
| c1galt1c1 | 0.157 |
| klhl8 | 0.157 |
| slc25a15 | 0.157 |
| asbb2 | 0.157 |
| ptgds | 0.157 |
| crhq1 | 0.157 |
| wd | 0.157 |
| ath13 | 0.157 |
| cia40 | 0.157 |
| pregq2 | 0.157 |
| d7mit67 | 0.157 |
| d4mit148 | 0.157 |
| gutl1 | 0.157 |
| hspb2 | 0.157 |
| fxc1 | 0.157 |
| psma6 | 0.157 |
| d5mit254 | 0.157 |
| rars2 | 0.157 |
| ndufb11 | 0.157 |
| col18a1 | 0.157 |
| ccbl2 | 0.156 |
| d1mit403 | 0.156 |
| efhc2 | 0.156 |
| tm | 0.156 |
| cox7b2 | 0.156 |
| d6mit14 | 0.156 |
| slc36a1 | 0.156 |
| ctss | 0.156 |
| armetl1 | 0.156 |
| 4930455b06rik | 0.156 |
| sbf2 | 0.156 |
| adam9 | 0.156 |
| aga | 0.156 |
| arl13b | 0.156 |
| ril2 | 0.156 |
| ril3 | 0.156 |
| slitrk1 | 0.156 |
| d4mit19 | 0.156 |
| tuba1b | 0.156 |
| lca5 | 0.156 |
| ndufa7 | 0.156 |
| 6430704m03rik | 0.156 |
| dnaic2 | 0.155 |
| 1110012l19rik | 0.155 |
| d1mit274 | 0.155 |
| ager | 0.155 |
| klhl14 | 0.155 |
| ssial1 | 0.155 |
| ppp1r3a | 0.155 |
