# Supplementary material for: GIT2 Acts as a Potential Keystone Protein in Functional Hypothalamic Networks Associated with Age-Related Phenotypic Changes in Rats
Source: PLoS One. 2012 May 14;7(5):e36975. doi: 10.1371/journal.pone.0036975 (PMC3351446; doi:10.1371/journal.pone.0036975)
Supplement: Table S11 — GeneIndexer latent semantic indexing (LSI) of significantly-regulated ‘Focal adhesion’ KEGG pathway. Using the KEGG signaling pathway ‘Focal adhesion’ as an input term, a list of the top 1000 implicitly-correlated LSI correlation score >0.1) was generated using a full genome background list. (DOC) [file pone.0036975.s015.doc]

**Table S11. GeneIndexer latent semantic indexing (LSI) of significantly-regulated ‘Focal adhesion’ KEGG pathway.** Using the KEGG signaling pathway ‘Focal adhesion’ as an input term, a list of the top 1000 implicitly-correlated (LSI correlation score >0.1) was generated using a full genome background list.

| ***Focal adhesion*** |  |
| --- | --- |
|  |  |
| **Protein Symbol** | **LSI correlation score** |
| nudt16l1 | 0.691 |
| d10wsu52e | 0.639 |
| parvg | 0.532 |
| parva | 0.507 |
| tns1 | 0.494 |
| fblim1 | 0.488 |
| parvb | 0.487 |
| fermt2 | 0.487 |
| lpxn | 0.474 |
| cbll1 | 0.47 |
| tln2 | 0.459 |
| tm4sf5 | 0.447 |
| pxn | 0.444 |
| amica1 | 0.443 |
| layn | 0.44 |
| tln1 | 0.44 |
| vcl | 0.439 |
| fer | 0.436 |
| lims2 | 0.435 |
| lims1 | 0.433 |
| coro2b | 0.427 |
| itgb1bp1 | 0.427 |
| itgb1bp3 | 0.423 |
| vit | 0.417 |
| fermt3 | 0.408 |
| fermt1 | 0.406 |
| git1 | 0.406 |
| d930005d10rik | 0.403 |
| nexn | 0.398 |
| zyx | 0.397 |
| grlf1 | 0.396 |
| apbb1ip | 0.393 |
| rp23-157o10.7 | 0.393 |
| npnt | 0.392 |
| bcar1 | 0.391 |
| tgfb1i1 | 0.39 |
| actn1 | 0.39 |
| cass4 | 0.388 |
| tns4 | 0.385 |
| nedd9 | 0.384 |
| dock1 | 0.384 |
| ddef1 | 0.382 |
| git2 | 0.381 |
| cadm2 | 0.378 |
| rwdd4a | 0.377 |
| edil3 | 0.377 |
| sorbs3 | 0.371 |
| arhgap26 | 0.371 |
| ilk | 0.371 |
| ssx2ip | 0.369 |
| dimt1 | 0.367 |
| pvrl3 | 0.366 |
| trip6 | 0.364 |
| actn4 | 0.364 |
| ptpn12 | 0.36 |
| itga8 | 0.359 |
| arhgap9 | 0.358 |
| rhbdd2 | 0.358 |
| iqsec1 | 0.358 |
| ptprm | 0.355 |
| arhgap21 | 0.355 |
| cdh18 | 0.354 |
| d9sut1e | 0.352 |
| lima1 | 0.35 |
| tns3 | 0.349 |
| palld | 0.349 |
| ptpn14 | 0.349 |
| itgb8 | 0.348 |
| lpp | 0.345 |
| svil | 0.345 |
| thsd1 | 0.345 |
| d0wfb1e | 0.345 |
| d15nds1 | 0.345 |
| cercam | 0.344 |
| kifc4b | 0.344 |
| 2810004i08rik | 0.344 |
| actl7a | 0.343 |
| lrrc4b | 0.343 |
| fndc4 | 0.34 |
| jub | 0.34 |
| raver1 | 0.339 |
| mcam | 0.339 |
| efs | 0.338 |
| arhgef7 | 0.337 |
| arhgef6 | 0.337 |
| stard13 | 0.333 |
| u06147 | 0.333 |
| ssh2 | 0.333 |
| hepacam | 0.333 |
| nck2 | 0.331 |
| pcdha4 | 0.331 |
| ddef2 | 0.33 |
| b4galnt3 | 0.33 |
| sh2d3c | 0.329 |
| mllt4 | 0.327 |
| cd151 | 0.326 |
| arhgap24 | 0.326 |
| cdh24 | 0.325 |
| cd300lg | 0.324 |
| pvrl4 | 0.324 |
| clec3a | 0.324 |
| pvr | 0.324 |
| jam2 | 0.323 |
| cadm3 | 0.323 |
| tenc1 | 0.323 |
| bc004728 | 0.323 |
| tg(itga5)0844fmw | 0.322 |
| bcar3 | 0.322 |
| pcdh12 | 0.321 |
| jam3 | 0.321 |
| centd1 | 0.32 |
| pdlim2 | 0.32 |
| pvrl1 | 0.32 |
| cadm4 | 0.319 |
| tes | 0.319 |
| tesk1 | 0.318 |
| arhgap5 | 0.316 |
| ptk2b | 0.316 |
| cml2 | 0.316 |
| esam1 | 0.316 |
| megf11 | 0.314 |
| cdcp1 | 0.314 |
| f11r | 0.314 |
| rac3 | 0.312 |
| flrt2 | 0.312 |
| igsf5 | 0.311 |
| gpr124 | 0.31 |
| rhod | 0.31 |
| flrt1 | 0.309 |
| rsu1 | 0.308 |
| icam4 | 0.308 |
| centd3 | 0.307 |
| sorbs1 | 0.307 |
| tg(itgb1)0840fmw | 0.307 |
| bcnp1 | 0.307 |
| lgals8 | 0.306 |
| itga9 | 0.306 |
| rras | 0.305 |
| arpc2 | 0.305 |
| klhl20 | 0.304 |
| tg(itga2)1070fmw | 0.304 |
| ppfia1 | 0.303 |
| ninj2 | 0.303 |
| cml1 | 0.303 |
| cml5 | 0.303 |
| actn2 | 0.302 |
| vasp | 0.302 |
| elmo2 | 0.302 |
| crk | 0.301 |
| shisa4 | 0.301 |
| itgb5 | 0.301 |
| cdh12 | 0.3 |
| enah | 0.299 |
| ajap1 | 0.298 |
| sorbs2 | 0.297 |
| igsf11 | 0.297 |
| tg(krt14-cre)1efu | 0.297 |
| ptprh | 0.297 |
| rhou | 0.297 |
| fndc3b | 0.296 |
| mgat5b | 0.296 |
| elmo1 | 0.296 |
| plxnc1 | 0.295 |
| nrcam | 0.294 |
| itgb6 | 0.294 |
| ptprk | 0.293 |
| itga10 | 0.293 |
| evl | 0.293 |
| icam2 | 0.293 |
| aoc3-rs | 0.292 |
| 9030409g11rik | 0.291 |
| iqgap1 | 0.291 |
| itgb1bp2 | 0.291 |
| memo1 | 0.291 |
| pcdha3 | 0.29 |
| raph1 | 0.29 |
| ctnna2 | 0.29 |
| ctnnal1 | 0.289 |
| elmo3 | 0.289 |
| afap1 | 0.289 |
| ptpn23 | 0.288 |
| itgb7 | 0.288 |
| fat1 | 0.287 |
| sdk1 | 0.287 |
| stk35 | 0.286 |
| cd2ap | 0.286 |
| ctnnd1 | 0.285 |
| rnd3 | 0.285 |
| lmo7 | 0.285 |
| vasn | 0.285 |
| mical1 | 0.285 |
| flnb | 0.284 |
| d10mit161 | 0.284 |
| pcdh20 | 0.284 |
| b3gnt6 | 0.284 |
| itga1 | 0.284 |
| iqgap3 | 0.283 |
| icam5 | 0.282 |
| vezt | 0.282 |
| grit | 0.282 |
| nck1 | 0.281 |
| ppap2b | 0.281 |
| col22a1 | 0.281 |
| podxl | 0.28 |
| arhgap10 | 0.279 |
| abi2 | 0.278 |
| tpm4 | 0.278 |
| pkp4 | 0.276 |
| e230028l10rik | 0.276 |
| ctnna1 | 0.276 |
| cadm1 | 0.276 |
| bcam | 0.276 |
| nfasc | 0.275 |
| cdc42ep1 | 0.275 |
| pcdhga12 | 0.275 |
| sdcbp | 0.274 |
| madcam1 | 0.274 |
| cttn | 0.273 |
| vtn | 0.273 |
| pcdhgc3 | 0.272 |
| lasp1 | 0.272 |
| hmcn1 | 0.272 |
| arpc4 | 0.271 |
| dchs1 | 0.271 |
| rapgef6 | 0.271 |
| scrib | 0.271 |
| chl1 | 0.271 |
| tspan9 | 0.27 |
| sirpa | 0.27 |
| spon2 | 0.27 |
| crkl | 0.27 |
| ptprf | 0.27 |
| pak4 | 0.269 |
| b4galt7 | 0.269 |
| ssh3 | 0.269 |
| pcdhgb4 | 0.269 |
| gripap1 | 0.268 |
| wtip | 0.268 |
| adam23 | 0.268 |
| tspan6 | 0.268 |
| col16a1 | 0.268 |
| rnf181 | 0.267 |
| rgnef | 0.267 |
| lcp1 | 0.266 |
| arpc1a | 0.266 |
| pvrl2 | 0.266 |
| ptprt | 0.266 |
| sdk2 | 0.266 |
| tesk2 | 0.265 |
| lrit1 | 0.265 |
| eg226654 | 0.265 |
| rapgef1 | 0.265 |
| cd47 | 0.265 |
| cdh5 | 0.265 |
| bai1 | 0.264 |
| iqgap2 | 0.264 |
| dock2 | 0.264 |
| adam15 | 0.263 |
| ptprb | 0.263 |
| skap2 | 0.262 |
| flrt3 | 0.261 |
| itga3 | 0.261 |
| pip5k1c | 0.261 |
| myo10 | 0.261 |
| magi1 | 0.261 |
| cgnl1 | 0.261 |
| mpzl1 | 0.26 |
| amot | 0.26 |
| gm784 | 0.26 |
| cntn1 | 0.259 |
| rap1b | 0.259 |
| angptl6 | 0.258 |
| csk | 0.258 |
| clca5 | 0.258 |
| egfl6 | 0.258 |
| kirrel | 0.258 |
| trio | 0.258 |
| tnn | 0.258 |
| pcdh9 | 0.257 |
| mdga1 | 0.257 |
| cd96 | 0.257 |
| emilin1 | 0.256 |
| vav2 | 0.256 |
| rap1gap | 0.256 |
| flna | 0.255 |
| mpzl2 | 0.255 |
| swap70 | 0.255 |
| sh3pxd2a | 0.254 |
| tmem204 | 0.254 |
| rap1a | 0.254 |
| ddefl1 | 0.253 |
| adam22 | 0.253 |
| abl2 | 0.253 |
| pgm5 | 0.253 |
| slk | 0.253 |
| fert2 | 0.253 |
| cib3 | 0.252 |
| synpo | 0.252 |
| lamc3 | 0.252 |
| itgad | 0.252 |
| jup | 0.252 |
| rhog | 0.251 |
| rhof | 0.251 |
| apold1 | 0.251 |
| flnc | 0.251 |
| rplag | 0.251 |
| d13mit260 | 0.251 |
| dok1 | 0.25 |
| cib1 | 0.25 |
| skap1 | 0.25 |
| igsf8 | 0.25 |
| itga7 | 0.25 |
| d15mit14 | 0.25 |
| ankrd28 | 0.249 |
| cntnap1 | 0.249 |
| cdh2 | 0.249 |
| garnl4 | 0.249 |
| dsg2 | 0.249 |
| pcdh10 | 0.248 |
| alcam | 0.248 |
| xmv19 | 0.248 |
| prtg | 0.248 |
| ndufb7 | 0.247 |
| lama4 | 0.247 |
| cspg4 | 0.247 |
| sdc4 | 0.247 |
| slc3a2 | 0.247 |
| cdh16 | 0.247 |
| dscaml1 | 0.246 |
| myh9 | 0.246 |
| avil | 0.246 |
| cd24c | 0.246 |
| cd24b | 0.246 |
| asah3l | 0.246 |
| ptprj | 0.246 |
| shroom4 | 0.246 |
| mp | 0.246 |
| ssh1 | 0.246 |
| pscd1 | 0.246 |
| tpbg | 0.245 |
| cdh17 | 0.245 |
| cdh13 | 0.245 |
| tspan1 | 0.245 |
| dsc2 | 0.245 |
| dlc1 | 0.244 |
| xmv16 | 0.244 |
| cirbp-rs2 | 0.243 |
| limk2 | 0.243 |
| mgat5 | 0.243 |
| sipa1 | 0.243 |
| rap1gds1 | 0.243 |
| tspan4 | 0.243 |
| rasgrp2 | 0.242 |
| hnt | 0.242 |
| ceacam1 | 0.242 |
| tspan3 | 0.242 |
| cdc42ep5 | 0.242 |
| rere | 0.242 |
| pdlim1 | 0.242 |
| prl2c4 | 0.241 |
| mia3 | 0.24 |
| aamp | 0.24 |
| pkp3 | 0.24 |
| l3mbtl4 | 0.24 |
| rapgef2 | 0.24 |
| fbln7 | 0.24 |
| lamb2 | 0.24 |
| itgb2l | 0.239 |
| mpp6 | 0.239 |
| itga5 | 0.239 |
| cdh11 | 0.239 |
| rnd1 | 0.239 |
| itga4 | 0.239 |
| gldn | 0.238 |
| ociad1 | 0.238 |
| antxr1 | 0.238 |
| gnb2l1 | 0.238 |
| rasa1 | 0.237 |
| ctnnd2 | 0.237 |
| zfp639 | 0.237 |
| fndc1 | 0.237 |
| sema7a | 0.237 |
| cdh3 | 0.236 |
| poldip2 | 0.236 |
| pstpip1 | 0.236 |
| zfp414 | 0.235 |
| amigo1 | 0.235 |
| amigo3 | 0.235 |
| cml3 | 0.235 |
| nphp1 | 0.235 |
| mgat3 | 0.235 |
| tnk2 | 0.235 |
| pag1 | 0.235 |
| limk1 | 0.235 |
| adam3 | 0.234 |
| pdlim4 | 0.234 |
| arhgef4 | 0.234 |
| frem3 | 0.234 |
| dbn1 | 0.234 |
| pcdh18 | 0.234 |
| astn1 | 0.233 |
| cdc42ep4 | 0.233 |
| ptpru | 0.233 |
| yes1 | 0.233 |
| rpsa | 0.233 |
| adam28 | 0.233 |
| abi1 | 0.232 |
| dsc3 | 0.232 |
| snx20 | 0.232 |
| ddr1 | 0.232 |
| h36 | 0.232 |
| sdcbp2 | 0.232 |
| ptprz1 | 0.231 |
| dsp | 0.231 |
| plxnb1 | 0.231 |
| itga11 | 0.231 |
| au040829 | 0.231 |
| ttc9 | 0.23 |
| sh3kbp1 | 0.23 |
| cfl1 | 0.23 |
| mkl2 | 0.23 |
| amigo2 | 0.23 |
| diap1 | 0.23 |
| grb7 | 0.229 |
| thy1 | 0.229 |
| rhpn1 | 0.228 |
| ptpn21 | 0.228 |
| col13a1 | 0.228 |
| arpc5 | 0.228 |
| vav3 | 0.228 |
| rtkn | 0.228 |
| pkp1 | 0.228 |
| pfn1 | 0.227 |
| 9030425e11rik | 0.227 |
| plek2 | 0.227 |
| nphp4 | 0.227 |
| nphs1 | 0.227 |
| cd82 | 0.227 |
| itgb4 | 0.226 |
| ilkap | 0.226 |
| epha1 | 0.226 |
| cd24a | 0.225 |
| cdh15 | 0.225 |
| arf6 | 0.225 |
| nckap1 | 0.225 |
| fhl3 | 0.225 |
| 4930506m07rik | 0.225 |
| adam2 | 0.224 |
| epha2 | 0.224 |
| capn2 | 0.224 |
| adam34 | 0.224 |
| actr3 | 0.224 |
| cd9 | 0.224 |
| chst10 | 0.224 |
| tmod3 | 0.224 |
| rhoc | 0.223 |
| nf2 | 0.223 |
| tnr | 0.223 |
| ptpn18 | 0.223 |
| cdh9 | 0.222 |
| cdc42ep2 | 0.222 |
| emp2 | 0.222 |
| cdh4 | 0.222 |
| wasf2 | 0.222 |
| itgae | 0.222 |
| sparcl1 | 0.222 |
| shroom1 | 0.221 |
| art5 | 0.221 |
| fyb | 0.221 |
| b3gnt3 | 0.221 |
| wasf3 | 0.221 |
| srgap3 | 0.221 |
| fscn1 | 0.221 |
| cxadr | 0.221 |
| arpc3 | 0.22 |
| csmd2 | 0.22 |
| nckipsd | 0.22 |
| plec1 | 0.22 |
| abi3 | 0.22 |
| amotl1 | 0.219 |
| postn | 0.219 |
| tg(jup)4pac | 0.219 |
| tg(jup)21pac | 0.219 |
| tg(jup)9pac | 0.219 |
| tg(jup)45pac | 0.219 |
| dock3 | 0.219 |
| cd248 | 0.219 |
| lrrc7 | 0.219 |
| myom1 | 0.219 |
| micall2 | 0.219 |
| 2010109i03rik | 0.219 |
| d15mit13 | 0.219 |
| b4galt1 | 0.219 |
| pard3 | 0.219 |
| ccl25 | 0.218 |
| d18mit22 | 0.218 |
| d18mit92 | 0.218 |
| d18mit198 | 0.218 |
| cdh22 | 0.218 |
| cdh10 | 0.218 |
| d9mit264 | 0.218 |
| negr1 | 0.217 |
| rnd2 | 0.216 |
| mkln1 | 0.216 |
| ppp1r12c | 0.216 |
| lypd3 | 0.216 |
| itgb3bp | 0.216 |
| khdrbs2 | 0.215 |
| zan | 0.215 |
| pmv23 | 0.215 |
| fgr | 0.215 |
| dsc1 | 0.215 |
| tgfbi | 0.214 |
| ostf1 | 0.214 |
| prune | 0.214 |
| sema4d | 0.214 |
| 1-Sep | 0.214 |
| cd97 | 0.214 |
| actn3 | 0.214 |
| dstn | 0.213 |
| racgap1 | 0.213 |
| scyl3 | 0.213 |
| arvcf | 0.213 |
| fhl2 | 0.213 |
| 3110043j09rik | 0.212 |
| spata13 | 0.212 |
| arpc1b | 0.212 |
| rhob | 0.212 |
| itga6 | 0.212 |
| loc384848 | 0.212 |
| cntn6 | 0.212 |
| nrap | 0.212 |
| cd99 | 0.212 |
| tro | 0.212 |
| akap12 | 0.211 |
| dsg1b | 0.211 |
| dock4 | 0.211 |
| tinag | 0.211 |
| 4930500o05rik | 0.211 |
| gsn | 0.211 |
| nebl | 0.211 |
| d10mit114 | 0.211 |
| dsg1a | 0.211 |
| cyr61 | 0.21 |
| amotl2 | 0.21 |
| kptn | 0.21 |
| flii | 0.21 |
| pak1ip1 | 0.21 |
| centg1 | 0.209 |
| stard8 | 0.209 |
| col8a1 | 0.209 |
| arhgef15 | 0.209 |
| epb4.1l3 | 0.209 |
| 1110006o17rik | 0.209 |
| samd1 | 0.209 |
| nanos1 | 0.209 |
| mirn328 | 0.209 |
| arhgap17 | 0.209 |
| ctnna3 | 0.208 |
| pk | 0.208 |
| mupcdh | 0.208 |
| map2k1ip1 | 0.208 |
| stap2 | 0.208 |
| mprip | 0.208 |
| crtam | 0.207 |
| loc641201 | 0.207 |
| tiam1 | 0.207 |
| arhgap22 | 0.207 |
| mfge8 | 0.207 |
| efna1 | 0.207 |
| cald1 | 0.207 |
| fat4 | 0.207 |
| srgap2 | 0.206 |
| fhod1 | 0.206 |
| mmrn2 | 0.206 |
| dsg4 | 0.206 |
| smoc2 | 0.206 |
| cd93 | 0.206 |
| lama5 | 0.206 |
| adam12 | 0.206 |
| vsig1 | 0.206 |
| cntnap2 | 0.206 |
| kank1 | 0.206 |
| epb4.9 | 0.206 |
| msn | 0.205 |
| mmrn1 | 0.205 |
| 2310014h01rik | 0.205 |
| plek | 0.205 |
| rock1 | 0.205 |
| arhgef12 | 0.205 |
| ptprq | 0.205 |
| actr2 | 0.205 |
| lsp1 | 0.204 |
| pcdhga3 | 0.204 |
| ezr | 0.204 |
| cea | 0.204 |
| l1cam | 0.204 |
| erbb2ip | 0.204 |
| treml1 | 0.204 |
| klf8 | 0.204 |
| ptpra | 0.204 |
| cntn4 | 0.203 |
| dok2 | 0.203 |
| ncan | 0.203 |
| pak3 | 0.203 |
| 2810003c17rik | 0.203 |
| dchs2 | 0.202 |
| emcn | 0.202 |
| coro1b | 0.202 |
| xirp1 | 0.202 |
| srpx2 | 0.202 |
| cdgap | 0.201 |
| pak1 | 0.201 |
| kbtbd10 | 0.201 |
| pcdh7 | 0.201 |
| bves | 0.201 |
| 4831426i19rik | 0.201 |
| krit1 | 0.201 |
| mpzl3 | 0.201 |
| limd1 | 0.2 |
| sdc2 | 0.2 |
| ptprd | 0.2 |
| bsg | 0.2 |
| mpp5 | 0.2 |
| magi2 | 0.199 |
| capns1 | 0.199 |
| tcam1 | 0.199 |
| st6galnac1 | 0.199 |
| cdc42ep3 | 0.199 |
| pvcan2 | 0.199 |
| pvcan1 | 0.199 |
| pvcan3 | 0.199 |
| osbpl3 | 0.198 |
| ptk7 | 0.198 |
| frem2 | 0.198 |
| 6720456b07rik | 0.198 |
| frem1 | 0.198 |
| col4a6 | 0.198 |
| ppm1f | 0.197 |
| rgma | 0.197 |
| cntn5 | 0.197 |
| olfm4 | 0.197 |
| nphs2 | 0.197 |
| adam9 | 0.197 |
| rai14 | 0.197 |
| ai427122 | 0.197 |
| dag1 | 0.197 |
| macf1 | 0.197 |
| st8sia4 | 0.197 |
| arhgdig | 0.197 |
| tnc | 0.197 |
| podxl2 | 0.197 |
| pik3r6 | 0.197 |
| lrg1 | 0.196 |
| fxyd5 | 0.196 |
| ank3 | 0.196 |
| neu3 | 0.196 |
| cnn2 | 0.196 |
| hmmr | 0.196 |
| arhgef1 | 0.196 |
| gpr56 | 0.196 |
| icam1-rs1 | 0.195 |
| pkd1 | 0.195 |
| fmnl1 | 0.195 |
| syx1 | 0.195 |
| syx2 | 0.195 |
| d8ertd82e | 0.195 |
| itgbl1 | 0.195 |
| esm1 | 0.195 |
| ablim1 | 0.195 |
| mylk | 0.195 |
| magi3 | 0.194 |
| dsg3 | 0.194 |
| plekhg5 | 0.194 |
| ppp1r12b | 0.194 |
| arhgap4 | 0.194 |
| ceacam3 | 0.194 |
| cnpy2 | 0.194 |
| glycam1 | 0.193 |
| syngap1 | 0.193 |
| wipf1 | 0.193 |
| rock2 | 0.193 |
| chn2 | 0.193 |
| ccm2 | 0.193 |
| triobp | 0.193 |
| gpc2 | 0.193 |
| fap | 0.193 |
| pfn2 | 0.192 |
| exoc2 | 0.192 |
| cdc42se1 | 0.192 |
| loc100042493 | 0.192 |
| sspo | 0.192 |
| rdx | 0.192 |
| itgal | 0.192 |
| krtap8-1 | 0.192 |
| spock1 | 0.191 |
| rassf5 | 0.191 |
| diap3 | 0.191 |
| adamtsl1 | 0.191 |
| loxl2 | 0.191 |
| map4k4 | 0.191 |
| dab2 | 0.191 |
| nov | 0.191 |
| filip1 | 0.19 |
| actg1 | 0.19 |
| dscam | 0.19 |
| cd302 | 0.19 |
| cd226 | 0.19 |
| mpp1 | 0.19 |
| bc067047 | 0.19 |
| mpp2 | 0.19 |
| chad | 0.189 |
| wasf1 | 0.189 |
| b3gnt1 | 0.189 |
| mfap4 | 0.189 |
| chst1 | 0.189 |
| zkscan3 | 0.189 |
| myh10 | 0.188 |
| ninj1 | 0.188 |
| iqcb1 | 0.188 |
| ncam2 | 0.188 |
| xirp2 | 0.188 |
| eps8 | 0.188 |
| itgav | 0.188 |
| thsd3 | 0.188 |
| fras1 | 0.188 |
| pdlim7 | 0.188 |
| cask | 0.188 |
| scube1 | 0.187 |
| cd53 | 0.187 |
| gal3st2 | 0.187 |
| cntn3 | 0.187 |
| cdc42bpa | 0.187 |
| ephb1 | 0.187 |
| d10ertd140e | 0.187 |
| mkl1 | 0.186 |
| itga2 | 0.186 |
| 9130404d14rik | 0.186 |
| pcnp | 0.186 |
| vnn3 | 0.186 |
| cd63 | 0.186 |
| ly30 | 0.186 |
| col8a2 | 0.186 |
| acp1 | 0.185 |
| spn | 0.185 |
| dbnl | 0.185 |
| shroom2 | 0.185 |
| lgals1 | 0.185 |
| daam1 | 0.185 |
| fut7 | 0.185 |
| pstpip2 | 0.185 |
| net1 | 0.185 |
| pkp2 | 0.185 |
| siglec1 | 0.185 |
| plxnd1 | 0.184 |
| epha8 | 0.184 |
| nat8l | 0.184 |
| vill | 0.184 |
| adamtsl3 | 0.184 |
| ptrh2 | 0.184 |
| mmp24 | 0.184 |
| gca | 0.183 |
| ptpro | 0.183 |
| col4a3 | 0.183 |
| adam8 | 0.183 |
| d8mit336 | 0.183 |
| d8mit96 | 0.183 |
| cd164 | 0.183 |
| emv20 | 0.183 |
| gna13 | 0.183 |
| gpatch8 | 0.183 |
| col17a1 | 0.182 |
| lgals3bp | 0.182 |
| kif9 | 0.182 |
| itgax | 0.182 |
| arhgap1 | 0.182 |
| cdh6 | 0.182 |
| tmem123 | 0.182 |
| adam26a | 0.182 |
| bag3 | 0.182 |
| camk2n2 | 0.182 |
| muc13 | 0.182 |
| ptpn20 | 0.182 |
| fbln5 | 0.182 |
| ptpn1 | 0.181 |
| rb1cc1 | 0.181 |
| tm4sf1 | 0.181 |
| celsr1 | 0.181 |
| scn2b | 0.181 |
| capzb | 0.181 |
| rap2a | 0.181 |
| fut4-ps1 | 0.181 |
| lrrc15 | 0.18 |
| fbf1 | 0.18 |
| farp2 | 0.18 |
| gp6 | 0.18 |
| gpr4 | 0.18 |
| tg(krt5-cre)1tak | 0.18 |
| gm1123 | 0.18 |
| klhl2 | 0.18 |
| lrfn5 | 0.18 |
| mras | 0.18 |
| ccl21c | 0.179 |
| pi4k2a | 0.179 |
| pcdh19 | 0.179 |
| ceacam10 | 0.179 |
| clec1b | 0.179 |
| rod1 | 0.179 |
| nt5e | 0.179 |
| ccr10 | 0.178 |
| adam1a | 0.178 |
| nlgn1 | 0.178 |
| arhgdib | 0.178 |
| fchsd2 | 0.178 |
| ddr2 | 0.178 |
| coro1c | 0.178 |
| tiam2 | 0.178 |
| ubxd5 | 0.178 |
| speer4d | 0.178 |
| mertk | 0.178 |
| capn1 | 0.177 |
| plvap | 0.177 |
| chst4 | 0.177 |
| dmn | 0.177 |
| cgn | 0.177 |
| epb4.1l4b | 0.177 |
| adrm1 | 0.177 |
| lum | 0.176 |
| rhobtb1 | 0.176 |
| prkd1 | 0.176 |
| pf | 0.176 |
| slc35c1 | 0.176 |
| shb | 0.176 |
| mtss1 | 0.176 |
| gpr125 | 0.175 |
| fndc5 | 0.175 |
| snai2 | 0.175 |
| capg | 0.175 |
| pcdhga1 | 0.175 |
| tg(ctnnb1)1efu | 0.175 |
| sh3bp1 | 0.175 |
| stab1 | 0.175 |
| lox | 0.175 |
| gcnt3 | 0.175 |
| rac2 | 0.175 |
| d8mit339 | 0.175 |
| 1200014j11rik | 0.175 |
| d6mit252 | 0.174 |
| bst1 | 0.174 |
| farp1 | 0.174 |
| sparc | 0.174 |
| pscdbp | 0.174 |
| pcdha12 | 0.174 |
| adam21 | 0.174 |
| tiaf2 | 0.174 |
| lgals9 | 0.174 |
| olfm3 | 0.173 |
| clca1 | 0.173 |
| stab2 | 0.173 |
| d6wsu176e | 0.173 |
| shbdp1 | 0.173 |
| st8sia2 | 0.173 |
| pdpn | 0.173 |
| sema4a | 0.173 |
| adam11 | 0.173 |
| kirrel2 | 0.173 |
| emb | 0.173 |
| 1110012m11rik | 0.172 |
| tnxb | 0.172 |
| ppp1r14b | 0.172 |
| col19a1 | 0.172 |
| sdc1 | 0.172 |
| d7mit178 | 0.172 |
| ppfia2 | 0.172 |
| ptk2 | 0.172 |
| ermn | 0.172 |
| plce1 | 0.172 |
| mmp16 | 0.171 |
| muc10 | 0.171 |
| adam5 | 0.171 |
| rassf3 | 0.171 |
| podn | 0.171 |
| twf1 | 0.171 |
| igsf9 | 0.171 |
| strn4 | 0.171 |
| col4a4 | 0.171 |
| gp5 | 0.171 |
| crb3 | 0.171 |
| rhov | 0.171 |
| twf2 | 0.17 |
| nlgn3 | 0.17 |
| fat3 | 0.17 |
| baiap2 | 0.17 |
| arhgdia | 0.17 |
| enpp2 | 0.17 |
| lgtn | 0.17 |
| ecm2 | 0.17 |
| antxr2 | 0.17 |
| dusp26 | 0.17 |
| rhobtb3 | 0.17 |
| ppp1r12a | 0.17 |
| plekhg6 | 0.169 |
| tsta3 | 0.169 |
| plxdc1 | 0.169 |
| robo4 | 0.169 |
| epb4.1l2 | 0.169 |
| arf5 | 0.169 |
| mag | 0.169 |
| hspg2 | 0.169 |
| synj2 | 0.169 |
| pak2 | 0.169 |
| bc060632 | 0.169 |
| ablim3 | 0.168 |
| ranbp9 | 0.168 |
| myo1f | 0.168 |
| bmx | 0.168 |
| plaur | 0.168 |
| cd99l2 | 0.168 |
| lrrc16a | 0.168 |
| chst2 | 0.168 |
| synpo2 | 0.168 |
| ptp4a3 | 0.168 |
| fbln1 | 0.168 |
| matk | 0.168 |
| d0h4s114 | 0.168 |
| eps8l1 | 0.168 |
| smpx | 0.168 |
| st3gal4 | 0.167 |
| kirrel3 | 0.167 |
| nckap1l | 0.167 |
| ifaprc2 | 0.167 |
| epb4.1l5 | 0.167 |
| arhgap15 | 0.167 |
| zpbp | 0.167 |
| shc4 | 0.167 |
| cntn2 | 0.167 |
| adam24 | 0.167 |
| fsd1 | 0.167 |
| tmem25 | 0.167 |
| coro2a | 0.167 |
| fhl1 | 0.167 |
| ssfa2 | 0.167 |
| dnm2 | 0.167 |
| tsc1 | 0.166 |
| 4631416l12rik | 0.166 |
| srgap1 | 0.166 |
| gp1bb | 0.166 |
| nek8 | 0.166 |
| ntn4 | 0.166 |
| axl | 0.166 |
| st6gal1 | 0.166 |
| tspan2 | 0.166 |
| hck | 0.166 |
| nid1 | 0.166 |
| cthrc1 | 0.166 |
| tinagl | 0.165 |
| mia1 | 0.165 |
| calr | 0.165 |
| epn3 | 0.165 |
| cib4 | 0.165 |
| rapgef3 | 0.165 |
| marcks | 0.165 |
| osgep | 0.165 |
| adam19 | 0.165 |
| spnb2 | 0.165 |
| ccl21b | 0.165 |
| dock6 | 0.164 |
| mfi2 | 0.164 |
| mrc2 | 0.164 |
| cx3cl1 | 0.164 |
| ai662250 | 0.164 |
| tjp2 | 0.164 |
| neo1 | 0.164 |
| nlgn2 | 0.164 |
| centg3 | 0.164 |
| fut8 | 0.164 |
| frmpd4 | 0.164 |
| adam1b | 0.164 |
| nrxn2 | 0.164 |
| ephb2 | 0.164 |
| def6 | 0.164 |
| col14a1 | 0.164 |
| efnb1 | 0.164 |
| eif6 | 0.164 |
| lpar2 | 0.164 |
| coro7 | 0.164 |
| tnfrsf12a | 0.163 |
| rab21 | 0.163 |
| zbtb8a | 0.163 |
| lpar1 | 0.163 |
| chn1 | 0.163 |
| col4a5 | 0.163 |
| cxcl16 | 0.163 |
| gna12 | 0.163 |
| 9-Mar | 0.162 |
| col18a1 | 0.162 |
| was | 0.162 |
| d10ertd610e | 0.162 |
| a130090k04rik | 0.162 |
| mpp7 | 0.162 |
