# Supplementary material for: GIT2 Acts as a Potential Keystone Protein in Functional Hypothalamic Networks Associated with Age-Related Phenotypic Changes in Rats
Source: PLoS One. 2012 May 14;7(5):e36975. doi: 10.1371/journal.pone.0036975 (PMC3351446; doi:10.1371/journal.pone.0036975)
Supplement: Table S12 — GeneIndexer latent semantic indexing (LSI) of significantly-regulated ‘MAPK signaling’ KEGG pathway. Using the KEGG signaling pathway ‘MAPK signaling’ as an input term, a list of the top 1000 implicitly-correlated (LSI correlation score >0.1) was generated using a full genome background list. (DOC) [file pone.0036975.s016.doc]

**Table S12. GeneIndexer latent semantic indexing (LSI) of significantly-regulated ‘MAPK signaling’ KEGG pathway.** Using the KEGG signaling pathway ‘MAPK signaling’ as an input term, a list of the top 1000 implicitly-correlated (LSI correlation score >0.1) was generated using a full genome background list.

| ***MAPK signaling*** |  |
| --- | --- |
|  |  |
| **Protein Symbol** | **LSI correlation score** |
| nudt16l1 | 0.675 |
| d10wsu52e | 0.593 |
| fer | 0.577 |
| cbll1 | 0.574 |
| parva | 0.568 |
| lpxn | 0.56 |
| tns1 | 0.542 |
| parvb | 0.533 |
| itgb1bp1 | 0.526 |
| tesk1 | 0.521 |
| parvg | 0.517 |
| arhgap26 | 0.508 |
| sorbs3 | 0.508 |
| rp23-157o10.7 | 0.49 |
| tln2 | 0.489 |
| fermt2 | 0.487 |
| fblim1 | 0.482 |
| d0wfb1e | 0.481 |
| itgb1bp3 | 0.48 |
| tesk2 | 0.479 |
| sh2d3c | 0.477 |
| lims1 | 0.477 |
| ptpn14 | 0.476 |
| stk35 | 0.472 |
| grlf1 | 0.468 |
| lims2 | 0.462 |
| slk | 0.457 |
| nedd9 | 0.451 |
| trip6 | 0.451 |
| arhgap21 | 0.449 |
| ptprh | 0.444 |
| jub | 0.443 |
| zyx | 0.443 |
| afap1 | 0.443 |
| tln1 | 0.442 |
| dimt1 | 0.442 |
| layn | 0.439 |
| stk24 | 0.437 |
| pxn | 0.435 |
| ptpn12 | 0.435 |
| tm4sf5 | 0.431 |
| ssh2 | 0.43 |
| edk | 0.43 |
| centd3 | 0.429 |
| ddef1 | 0.428 |
| coro2b | 0.428 |
| vcl | 0.426 |
| efs | 0.426 |
| git2 | 0.425 |
| arhgap10 | 0.425 |
| dock1 | 0.424 |
| tgfb1i1 | 0.422 |
| d15nds1 | 0.421 |
| sorbs2 | 0.42 |
| bcar1 | 0.417 |
| amica1 | 0.417 |
| ptpn23 | 0.416 |
| nck2 | 0.414 |
| arhgef6 | 0.413 |
| ilk | 0.412 |
| rsu1 | 0.412 |
| afap1l2 | 0.412 |
| ilkap | 0.411 |
| arhgap5 | 0.411 |
| tns3 | 0.411 |
| bcar3 | 0.411 |
| actn1 | 0.411 |
| 9130404d14rik | 0.411 |
| ptprm | 0.41 |
| d930005d10rik | 0.407 |
| ptpn21 | 0.406 |
| pdlim2 | 0.404 |
| lima1 | 0.402 |
| limk2 | 0.401 |
| cdcp1 | 0.401 |
| map2k1ip1 | 0.4 |
| fermt1 | 0.4 |
| tns4 | 0.4 |
| memo1 | 0.398 |
| apbb1ip | 0.397 |
| fermt3 | 0.396 |
| svil | 0.396 |
| ppfia1 | 0.393 |
| ptprk | 0.391 |
| dusp26 | 0.39 |
| fert2 | 0.39 |
| thsd1 | 0.389 |
| shc4 | 0.389 |
| arhgef7 | 0.388 |
| pak1ip1 | 0.388 |
| ptpn18 | 0.388 |
| pak4 | 0.388 |
| ssh3 | 0.388 |
| skap2 | 0.387 |
| mpzl1 | 0.386 |
| ppp1r12c | 0.385 |
| grit | 0.383 |
| d1mit508 | 0.383 |
| rnd3 | 0.383 |
| rgnef | 0.382 |
| 2610018g03rik | 0.382 |
| mapk15 | 0.382 |
| rcsd1 | 0.382 |
| rhou | 0.381 |
| palld | 0.38 |
| rhod | 0.379 |
| edil3 | 0.379 |
| actn4 | 0.378 |
| skap1 | 0.378 |
| npnt | 0.375 |
| arhgap9 | 0.375 |
| b4galnt3 | 0.375 |
| pag1 | 0.375 |
| ppm1f | 0.374 |
| sorbs1 | 0.373 |
| ssh1 | 0.371 |
| itgb8 | 0.37 |
| abi2 | 0.369 |
| ddef2 | 0.369 |
| khdrbs2 | 0.368 |
| lasp1 | 0.368 |
| lrrc4b | 0.367 |
| itga8 | 0.367 |
| dok1 | 0.366 |
| ankrd28 | 0.366 |
| mllt4 | 0.365 |
| tnik | 0.365 |
| lpp | 0.365 |
| kifc4b | 0.365 |
| 2810004i08rik | 0.365 |
| ppp1r14b | 0.365 |
| elmo2 | 0.364 |
| nexn | 0.363 |
| rwdd4a | 0.363 |
| rac3 | 0.363 |
| abl2 | 0.363 |
| iqsec1 | 0.362 |
| gripap1 | 0.362 |
| stap2 | 0.362 |
| cdc2b | 0.361 |
| ly6g6f | 0.36 |
| cadm2 | 0.36 |
| anks1 | 0.359 |
| arhgef15 | 0.359 |
| rras | 0.358 |
| elmo1 | 0.358 |
| pip5k1c | 0.357 |
| vit | 0.357 |
| tnk2 | 0.357 |
| ppm1e | 0.357 |
| tenc1 | 0.356 |
| igsf5 | 0.355 |
| camk2n2 | 0.354 |
| itgb1bp2 | 0.353 |
| garnl4 | 0.353 |
| pvrl3 | 0.352 |
| grb7 | 0.352 |
| rap1gds1 | 0.352 |
| vasp | 0.352 |
| plxnc1 | 0.351 |
| nrk | 0.351 |
| arhgap24 | 0.351 |
| limk1 | 0.349 |
| twf2 | 0.349 |
| raver1 | 0.349 |
| sh3pxd2a | 0.349 |
| trio | 0.349 |
| cdc42bpa | 0.348 |
| mical1 | 0.348 |
| rhbdd2 | 0.348 |
| sdcbp | 0.348 |
| plek | 0.347 |
| stk10 | 0.347 |
| klhl20 | 0.347 |
| sbk1 | 0.347 |
| ssx2ip | 0.347 |
| pstpip1 | 0.346 |
| pcdh12 | 0.346 |
| map4k4 | 0.346 |
| ptk2b | 0.346 |
| mprip | 0.345 |
| centg1 | 0.345 |
| rap1gap | 0.344 |
| dok2 | 0.343 |
| ppp1r12b | 0.343 |
| mcam | 0.343 |
| cdgap | 0.343 |
| stard13 | 0.342 |
| ptprf | 0.341 |
| nck1 | 0.341 |
| bai1 | 0.341 |
| t(11;19)42h | 0.341 |
| ptprb | 0.341 |
| bcnp1 | 0.34 |
| lcp1 | 0.34 |
| loc641201 | 0.339 |
| tg(krt14-cre)1efu | 0.339 |
| abi3 | 0.339 |
| rapgef1 | 0.338 |
| sh3bp5 | 0.338 |
| ptprj | 0.337 |
| ctnnal1 | 0.337 |
| centd1 | 0.337 |
| iqgap1 | 0.337 |
| epha8 | 0.337 |
| apold1 | 0.337 |
| avil | 0.336 |
| cdc42ep1 | 0.336 |
| yes1 | 0.336 |
| ppfibp1 | 0.336 |
| dok4 | 0.336 |
| cass4 | 0.335 |
| cnpy2 | 0.335 |
| stk38l | 0.335 |
| rhpn1 | 0.335 |
| gmfg | 0.335 |
| actn2 | 0.335 |
| pkn3 | 0.334 |
| dok3 | 0.334 |
| d1mit58 | 0.334 |
| mkln1 | 0.334 |
| ppap2b | 0.333 |
| ptprt | 0.333 |
| akap12 | 0.333 |
| shd | 0.333 |
| she | 0.333 |
| c330002i19rik | 0.332 |
| arpc2 | 0.332 |
| mtmr15 | 0.331 |
| pick2 | 0.331 |
| pick5 | 0.331 |
| pick3 | 0.331 |
| pick4 | 0.331 |
| rasgrp2 | 0.33 |
| megf11 | 0.33 |
| matk | 0.33 |
| rap1b | 0.329 |
| mgat5b | 0.329 |
| iqgap3 | 0.329 |
| coro1b | 0.328 |
| cd2ap | 0.328 |
| ajap1 | 0.328 |
| cd151 | 0.328 |
| racgap1 | 0.328 |
| plek2 | 0.328 |
| chn2 | 0.328 |
| flnb | 0.328 |
| d15mit13 | 0.327 |
| centg3 | 0.327 |
| swap70 | 0.327 |
| dlc1 | 0.327 |
| itga9 | 0.327 |
| crk | 0.327 |
| ysk4 | 0.327 |
| angptl6 | 0.326 |
| evl | 0.326 |
| pcdha4 | 0.326 |
| pak7 | 0.326 |
| 3930401k13rik | 0.326 |
| magi1 | 0.325 |
| smek1 | 0.325 |
| tspan9 | 0.325 |
| iqgap2 | 0.325 |
| rapgef2 | 0.325 |
| f11r | 0.324 |
| gpr124 | 0.324 |
| bmx | 0.324 |
| dyrk1c | 0.324 |
| mink1 | 0.324 |
| taok1 | 0.323 |
| cd24c | 0.323 |
| cd24b | 0.323 |
| hepacam | 0.323 |
| srms | 0.323 |
| a630047e20rik | 0.322 |
| csk | 0.322 |
| gnb2l1 | 0.322 |
| itgb5 | 0.321 |
| prkd2 | 0.321 |
| flrt1 | 0.32 |
| lgals8 | 0.32 |
| actl7a | 0.32 |
| esam1 | 0.32 |
| jam2 | 0.32 |
| lmo7 | 0.32 |
| flrt2 | 0.32 |
| vasn | 0.32 |
| rasa1 | 0.32 |
| kirrel | 0.32 |
| cib1 | 0.319 |
| dapp1 | 0.319 |
| elmo3 | 0.318 |
| gm944 | 0.318 |
| vav2 | 0.318 |
| ptk6 | 0.317 |
| pvr | 0.317 |
| cfl1 | 0.316 |
| jam3 | 0.316 |
| fhod1 | 0.316 |
| pak3 | 0.316 |
| sh3kbp1 | 0.316 |
| syngap1 | 0.315 |
| rtkn | 0.315 |
| sirpa | 0.315 |
| enah | 0.315 |
| ctnnd1 | 0.315 |
| ulk2 | 0.314 |
| ripk5 | 0.313 |
| ngef | 0.313 |
| taok2 | 0.313 |
| snx26 | 0.312 |
| d9sut1e | 0.312 |
| shf | 0.312 |
| rhog | 0.312 |
| crkl | 0.311 |
| magi3 | 0.311 |
| pmv23 | 0.311 |
| tpm4 | 0.311 |
| map3k9 | 0.311 |
| u06147 | 0.311 |
| lmtk3 | 0.311 |
| sipa1 | 0.311 |
| icam4 | 0.311 |
| ddr1 | 0.31 |
| cercam | 0.31 |
| dbnl | 0.31 |
| pak6 | 0.31 |
| tg(itga5)0844fmw | 0.31 |
| rhov | 0.31 |
| itgb6 | 0.31 |
| kank1 | 0.31 |
| xmv19 | 0.309 |
| shb | 0.309 |
| cald1 | 0.309 |
| ptk7 | 0.309 |
| abi1 | 0.309 |
| mpp6 | 0.308 |
| cdh18 | 0.308 |
| 9030409g11rik | 0.308 |
| cdc42bpb | 0.308 |
| rnd1 | 0.307 |
| 1110012m11rik | 0.307 |
| cttn | 0.307 |
| ptpru | 0.307 |
| cadm3 | 0.307 |
| pkn1 | 0.307 |
| nagk | 0.306 |
| 1110006o17rik | 0.306 |
| flnc | 0.306 |
| micalcl | 0.306 |
| pdlim7 | 0.306 |
| cml2 | 0.306 |
| cd300lg | 0.306 |
| ick | 0.305 |
| eps8 | 0.305 |
| 3110043j09rik | 0.305 |
| itgb3bp | 0.305 |
| epha1 | 0.305 |
| pvrl1 | 0.304 |
| dock2 | 0.304 |
| nek3 | 0.304 |
| pscd1 | 0.304 |
| pvrl4 | 0.303 |
| cfl2 | 0.303 |
| pip5kl1 | 0.303 |
| amot | 0.303 |
| plxnb1 | 0.302 |
| d15mit14 | 0.302 |
| arhgef4 | 0.302 |
| tes | 0.302 |
| mobkl1b | 0.301 |
| raph1 | 0.301 |
| cdc42ep5 | 0.301 |
| d2mit316 | 0.301 |
| fhl3 | 0.301 |
| emo1 | 0.3 |
| nckipsd | 0.3 |
| fndc4 | 0.3 |
| map4k3 | 0.3 |
| itga1 | 0.299 |
| bc004728 | 0.299 |
| flii | 0.299 |
| erbb2ip | 0.298 |
| rb1cc1 | 0.298 |
| cirbp-rs2 | 0.298 |
| cadm4 | 0.298 |
| ptprr | 0.297 |
| ppp1r14a | 0.297 |
| flna | 0.297 |
| c230081a13rik | 0.297 |
| pdik1l | 0.296 |
| ppp1r12a | 0.296 |
| riok3 | 0.296 |
| wnk2 | 0.296 |
| pcdhgc3 | 0.295 |
| mapk8ip3 | 0.295 |
| fat1 | 0.295 |
| rap1a | 0.295 |
| bc067047 | 0.294 |
| stk4 | 0.294 |
| cdc42se1 | 0.294 |
| mobkl1a | 0.294 |
| ranbp9 | 0.294 |
| cml1 | 0.294 |
| cml5 | 0.294 |
| spred2 | 0.293 |
| ptrh2 | 0.293 |
| phlppl | 0.293 |
| scrib | 0.293 |
| pkn2 | 0.293 |
| pdlim4 | 0.293 |
| ptpra | 0.292 |
| bcam | 0.292 |
| hcls1 | 0.292 |
| ttc9 | 0.291 |
| stk16 | 0.291 |
| adam22 | 0.291 |
| rin1 | 0.291 |
| arhgef12 | 0.291 |
| smok2a | 0.29 |
| cdh5 | 0.29 |
| cdc42ep2 | 0.29 |
| nckap1 | 0.29 |
| tg(itgb1)0840fmw | 0.29 |
| ctnna2 | 0.29 |
| fyb | 0.29 |
| rasa3 | 0.29 |
| usp6nl | 0.289 |
| itgb7 | 0.289 |
| btbd10 | 0.289 |
| ptprq | 0.289 |
| tiam1 | 0.289 |
| ilk-rs | 0.289 |
| myo10 | 0.289 |
| icam2 | 0.288 |
| mylk | 0.288 |
| prune | 0.288 |
| rap2a | 0.288 |
| camkv | 0.288 |
| lsp1 | 0.288 |
| chl1 | 0.288 |
| synpo | 0.287 |
| 1-Sep | 0.287 |
| pik3r6 | 0.287 |
| pdlim1 | 0.287 |
| prkd1 | 0.287 |
| prkx | 0.287 |
| ptpro | 0.287 |
| nrbp1 | 0.286 |
| 1810043h04rik | 0.286 |
| pip5k1b | 0.286 |
| phlpp | 0.286 |
| mark4 | 0.286 |
| ctnna1 | 0.286 |
| tfg | 0.286 |
| itga10 | 0.286 |
| rapgef6 | 0.285 |
| b4galt7 | 0.285 |
| cdc42ep4 | 0.285 |
| fndc3b | 0.285 |
| nrcam | 0.285 |
| ostf1 | 0.285 |
| mapk6 | 0.285 |
| cnksr1 | 0.285 |
| tiam2 | 0.285 |
| epha2 | 0.285 |
| igsf11 | 0.285 |
| slc3a2 | 0.284 |
| arpc5 | 0.284 |
| dusp16 | 0.284 |
| stk25 | 0.284 |
| icam5 | 0.284 |
| twf1 | 0.284 |
| ptpn20 | 0.284 |
| vtn | 0.284 |
| net1 | 0.283 |
| ddr2 | 0.283 |
| pik3c2b | 0.283 |
| mast1 | 0.283 |
| brsk1 | 0.283 |
| sdc4 | 0.283 |
| clec3a | 0.283 |
| pi4k2a | 0.283 |
| ppp1r14d | 0.282 |
| mlkl | 0.282 |
| efna1 | 0.282 |
| smoc2 | 0.282 |
| nphp1 | 0.282 |
| sema7a | 0.282 |
| ryk-rs1 | 0.282 |
| ptpdc1 | 0.282 |
| adam15 | 0.282 |
| aatk | 0.282 |
| egfl6 | 0.281 |
| rnf181 | 0.281 |
| arhgap1 | 0.281 |
| ink76 | 0.281 |
| ptprz1 | 0.28 |
| ckt2 | 0.28 |
| cdh12 | 0.28 |
| rhoc | 0.28 |
| pard3 | 0.28 |
| nisch | 0.28 |
| zc3hc1 | 0.28 |
| styk1 | 0.28 |
| dgkg | 0.28 |
| iph1 | 0.28 |
| pstpip2 | 0.279 |
| e230028l10rik | 0.279 |
| tnk1 | 0.279 |
| chn1 | 0.279 |
| cdkl2 | 0.279 |
| vav3 | 0.279 |
| clca5 | 0.279 |
| caskin2 | 0.278 |
| ddefl1 | 0.278 |
| mapk4 | 0.278 |
| lrrc7 | 0.278 |
| pkp4 | 0.278 |
| diap1 | 0.277 |
| cd6 | 0.277 |
| farp2 | 0.277 |
| map4k1 | 0.277 |
| dstn | 0.277 |
| nuak1 | 0.277 |
| in(17)1t | 0.277 |
| rassf5 | 0.277 |
| itga7 | 0.276 |
| ptpn5 | 0.276 |
| magi2 | 0.276 |
| rock2 | 0.276 |
| mylk2 | 0.276 |
| itga3 | 0.276 |
| rell1 | 0.276 |
| rell2 | 0.276 |
| ulk1 | 0.276 |
| cadm1 | 0.276 |
| cdh24 | 0.276 |
| podxl | 0.276 |
| epb4.9 | 0.275 |
| ninj2 | 0.275 |
| pitpnm3 | 0.275 |
| flrt3 | 0.275 |
| acp1 | 0.275 |
| myh9 | 0.275 |
| mpp2 | 0.275 |
| fgr | 0.275 |
| cib3 | 0.275 |
| nuak2 | 0.275 |
| tyro3-rs1 | 0.275 |
| arhgef1 | 0.275 |
| poldip2 | 0.275 |
| spred1 | 0.275 |
| pim3 | 0.274 |
| brsk2 | 0.274 |
| marcks | 0.274 |
| frs2 | 0.274 |
| arhgap4 | 0.274 |
| gkap1 | 0.274 |
| sdk1 | 0.274 |
| nrp | 0.274 |
| ptpmt1 | 0.274 |
| tssk3 | 0.274 |
| neu3 | 0.273 |
| sh3rf1 | 0.273 |
| eps8l2 | 0.273 |
| sav1 | 0.273 |
| ptpn7 | 0.273 |
| gulp1 | 0.272 |
| stap1 | 0.272 |
| pcnp | 0.272 |
| nf2 | 0.272 |
| ptpn4 | 0.272 |
| rhob | 0.272 |
| pcdh18 | 0.272 |
| vezt | 0.272 |
| ranbp10 | 0.272 |
| styxl1 | 0.271 |
| mras | 0.271 |
| nme6 | 0.271 |
| als2cr2 | 0.271 |
| 1700009n14rik | 0.271 |
| marcksl1 | 0.271 |
| arpc4 | 0.271 |
| mapk7 | 0.271 |
| ptprcap | 0.271 |
| ctnnd2 | 0.271 |
| mpp1 | 0.271 |
| plce1 | 0.27 |
| shisa4 | 0.27 |
| cabyr | 0.27 |
| jund2 | 0.27 |
| sh2d2a | 0.269 |
| mkl2 | 0.269 |
| aamp | 0.269 |
| scyl3 | 0.269 |
| ceacam1 | 0.269 |
| caskin1 | 0.269 |
| plekha2 | 0.269 |
| mast4 | 0.269 |
| plec1 | 0.269 |
| tyro3 | 0.269 |
| shcbp1 | 0.268 |
| pcdh20 | 0.268 |
| frs3 | 0.268 |
| ccm2 | 0.268 |
| dgkk | 0.267 |
| ppp1r1c | 0.267 |
| ak5 | 0.267 |
| cd96 | 0.267 |
| nek9 | 0.267 |
| ccdc88a | 0.267 |
| myom1 | 0.267 |
| mgat5 | 0.267 |
| d8ertd82e | 0.267 |
| shroom4 | 0.267 |
| med28 | 0.267 |
| pgm5 | 0.267 |
| tnn | 0.267 |
| antxr1 | 0.267 |
| bc060632 | 0.267 |
| ptprd | 0.266 |
| sh2b3 | 0.266 |
| dusp2 | 0.266 |
| jup | 0.266 |
| nphs1 | 0.266 |
| kank2 | 0.266 |
| ppp2r5b | 0.266 |
| arhgef2 | 0.266 |
| pak2 | 0.266 |
| cd47 | 0.265 |
| ppp2r2c | 0.265 |
| mark3 | 0.265 |
| shoc2 | 0.265 |
| rbks | 0.265 |
| cntn1 | 0.265 |
| cdc42ep3 | 0.264 |
| spon2 | 0.264 |
| bc010304 | 0.264 |
| gem | 0.264 |
| cnksr2 | 0.264 |
| ripk4 | 0.264 |
| cit | 0.264 |
| sema4d | 0.264 |
| cspg4 | 0.264 |
| smok2b | 0.264 |
| smok3a | 0.264 |
| smok3b | 0.264 |
| spata13 | 0.263 |
| spred3 | 0.263 |
| rnd2 | 0.263 |
| wasf2 | 0.263 |
| pfn1 | 0.263 |
| nfasc | 0.263 |
| tro | 0.263 |
| adam23 | 0.263 |
| rgma | 0.263 |
| rufy2 | 0.262 |
| fndc1 | 0.262 |
| them4 | 0.262 |
| alcam | 0.262 |
| dgkz | 0.262 |
| synpo2 | 0.262 |
| ephb1 | 0.262 |
| rhpn2 | 0.262 |
| rasgrp3 | 0.262 |
| frk | 0.262 |
| a230067g21rik | 0.262 |
| npcd | 0.261 |
| amigo2 | 0.261 |
| aoc3-rs | 0.261 |
| loc436194 | 0.261 |
| snx20 | 0.261 |
| eps8l3 | 0.261 |
| pvrl2 | 0.261 |
| mapkapk5 | 0.261 |
| stk3 | 0.261 |
| nme7 | 0.261 |
| plekhm3 | 0.261 |
| b3gnt6 | 0.261 |
| arhgap17 | 0.261 |
| plekhg5 | 0.26 |
| camk2n1 | 0.26 |
| zfp414 | 0.26 |
| gpr4 | 0.26 |
| ptpn13 | 0.26 |
| centa1 | 0.26 |
| cdh16 | 0.26 |
| mark2 | 0.26 |
| asah3l | 0.26 |
| asb15 | 0.26 |
| cgnl1 | 0.26 |
| lrrc4 | 0.26 |
| ccdc88c | 0.26 |
| pcdha3 | 0.26 |
| taok3 | 0.259 |
| wtip | 0.259 |
| gab1 | 0.259 |
| mtss1 | 0.259 |
| dapk3 | 0.259 |
| inppl1 | 0.259 |
| 1110008f13rik | 0.259 |
| clk2 | 0.259 |
| ropn1 | 0.259 |
| fscb | 0.259 |
| srgap3 | 0.258 |
| wee2 | 0.258 |
| amotl2 | 0.258 |
| cpne3 | 0.258 |
| plekhg6 | 0.258 |
| cdh13 | 0.258 |
| fchsd2 | 0.258 |
| wasf3 | 0.258 |
| ppm1a | 0.258 |
| psd | 0.258 |
| alpk2 | 0.257 |
| dbn1 | 0.257 |
| dab2 | 0.257 |
| sdcbp2 | 0.257 |
| nphp4 | 0.257 |
| axl | 0.257 |
| tg(itga2)1070fmw | 0.257 |
| sh3bp1 | 0.257 |
| sh2b2 | 0.257 |
| git1 | 0.257 |
| mgat3 | 0.256 |
| rplag | 0.256 |
| d13mit260 | 0.256 |
| dock4 | 0.256 |
| mertk | 0.256 |
| tspan3 | 0.256 |
| dusp18 | 0.256 |
| madcam1 | 0.256 |
| igsf8 | 0.255 |
| rapgef3 | 0.255 |
| pbk | 0.255 |
| 4930500o05rik | 0.255 |
| b230120h23rik | 0.255 |
| rock1 | 0.255 |
| amigo1 | 0.255 |
| amigo3 | 0.255 |
| tspan1 | 0.255 |
| hisppd1 | 0.255 |
| dusp6 | 0.255 |
| limd1 | 0.254 |
| rhof | 0.254 |
| pfn2 | 0.254 |
| mapkapk2 | 0.254 |
| itgb4 | 0.254 |
| dusp7 | 0.254 |
| osbpl3 | 0.254 |
| trp53rk | 0.254 |
| ksr2 | 0.254 |
| fscn1 | 0.254 |
| dgka | 0.254 |
| sirpb1 | 0.254 |
| cd99 | 0.254 |
| mpp5 | 0.254 |
| cask | 0.254 |
| dock3 | 0.254 |
| daam1 | 0.253 |
| lax1 | 0.253 |
| fes | 0.253 |
| dchs1 | 0.253 |
| arhgap29 | 0.253 |
| amotl1 | 0.253 |
| fhl2 | 0.253 |
| hmmr | 0.253 |
| pik3ap1 | 0.253 |
| rasal1 | 0.253 |
| ficd | 0.253 |
| pea15b | 0.253 |
| tspan15 | 0.252 |
| rere | 0.252 |
| bag3 | 0.252 |
| klhl2 | 0.252 |
| vill | 0.252 |
| dennd3 | 0.252 |
| camk1d | 0.252 |
| eps8l1 | 0.252 |
| map4k5 | 0.252 |
| dusp3 | 0.252 |
| map3k12 | 0.252 |
| tspan6 | 0.252 |
| ryk-ps1 | 0.251 |
| lime1 | 0.251 |
| rapgefl1 | 0.251 |
| tssk2 | 0.251 |
| iapls3-28 | 0.251 |
| itga5 | 0.251 |
| grb14 | 0.25 |
| phldb2 | 0.25 |
| cdk5r2 | 0.25 |
| thy1 | 0.25 |
| cdh2 | 0.25 |
| ptpre | 0.25 |
| ablim1 | 0.25 |
| synj2 | 0.25 |
| shroom1 | 0.25 |
| mobkl3 | 0.25 |
| tg(krt5-cre)1tak | 0.249 |
| actn3 | 0.249 |
| sla2 | 0.249 |
| cd24a | 0.249 |
| mast2 | 0.249 |
| dusp23 | 0.249 |
| pik3c2a | 0.248 |
| ai427122 | 0.248 |
| d10ertd610e | 0.248 |
| depdc2 | 0.248 |
| mkl1 | 0.248 |
| map3k13 | 0.248 |
| reps2 | 0.248 |
| dlgap4 | 0.248 |
| adk | 0.248 |
| msn | 0.248 |
| dusp4 | 0.247 |
| amph | 0.247 |
| eg226654 | 0.247 |
| epha6 | 0.247 |
| dusp22 | 0.247 |
| d10mit161 | 0.247 |
| sdk2 | 0.247 |
| lama4 | 0.247 |
| syx1 | 0.247 |
| syx2 | 0.247 |
| cdh3 | 0.246 |
| ahnak | 0.246 |
| eif6 | 0.246 |
| eef2k | 0.246 |
| map3k11 | 0.245 |
| lrit1 | 0.245 |
| coro7 | 0.245 |
| emilin1 | 0.245 |
| arhgdib | 0.245 |
| rpsa | 0.245 |
| ptpn3 | 0.245 |
| shc3 | 0.245 |
| adam24 | 0.244 |
| mdga1 | 0.244 |
| gsn | 0.244 |
| ppapdc1 | 0.244 |
| pip5k1a | 0.244 |
| nme2 | 0.244 |
| centd2 | 0.244 |
| tom1l1 | 0.244 |
| shc2 | 0.244 |
| nol1 | 0.244 |
| sh2d4a | 0.244 |
| krit1 | 0.244 |
| lamc3 | 0.244 |
| tmem204 | 0.244 |
| baiap2 | 0.244 |
| clec1b | 0.243 |
| 4930506m07rik | 0.243 |
| stk38 | 0.243 |
| prl2c4 | 0.243 |
| trip10 | 0.243 |
| il17rd | 0.243 |
| smtnl1 | 0.243 |
| pak1 | 0.243 |
| stk22s1 | 0.243 |
| itgad | 0.243 |
| cntnap1 | 0.243 |
| ltk | 0.243 |
| e030049g20rik | 0.243 |
| cd53 | 0.242 |
| sos2 | 0.242 |
| b230339m05rik | 0.242 |
| d11mit116 | 0.242 |
| ezr | 0.242 |
| akt1s1 | 0.242 |
| plekho1 | 0.242 |
| ect2 | 0.242 |
| dpysl3 | 0.242 |
| nek6 | 0.242 |
| map3k10 | 0.241 |
| arhgap22 | 0.241 |
| nebl | 0.241 |
| melk | 0.241 |
| itga11 | 0.241 |
| rasgrp1 | 0.241 |
| gm784 | 0.241 |
| ppm1b | 0.241 |
| ccrk | 0.241 |
| myom2 | 0.24 |
| ddx47 | 0.24 |
| ralgps2 | 0.24 |
| ceacam3 | 0.24 |
| mpzl2 | 0.24 |
| arpc1a | 0.24 |
| capn2 | 0.24 |
| zfp639 | 0.24 |
| arhgdia | 0.24 |
| dusp5 | 0.24 |
| uhmk1 | 0.24 |
| capzb | 0.24 |
| ablim3 | 0.24 |
| mcf2 | 0.24 |
| gigyf1 | 0.24 |
| dyrk1b | 0.239 |
| paqr3 | 0.239 |
| cep250 | 0.239 |
| d930014e17rik | 0.239 |
| mak | 0.239 |
| zfand6 | 0.239 |
| ralgds | 0.239 |
| rasgrf1 | 0.239 |
| actr3 | 0.239 |
| pip4k2b | 0.239 |
| pscdbp | 0.239 |
| myh10 | 0.239 |
| postn | 0.239 |
| rhot2 | 0.238 |
| arhgdig | 0.238 |
| cnn2 | 0.238 |
| 9030425e11rik | 0.238 |
| bnip2 | 0.238 |
| triobp | 0.238 |
| coro1c | 0.238 |
| nek8 | 0.238 |
| epha3 | 0.238 |
| arhgef11 | 0.238 |
| lats1 | 0.238 |
| dusp9 | 0.238 |
| itpka | 0.237 |
| mapbpip | 0.237 |
| gltscr2 | 0.237 |
| stk40 | 0.237 |
| stard8 | 0.237 |
| dsg2 | 0.237 |
| cd82 | 0.237 |
| mcf2l | 0.237 |
| dusp10 | 0.237 |
| myo18a | 0.237 |
| lamb2 | 0.237 |
| tssk6 | 0.237 |
| hsh2d | 0.237 |
| 2010109i03rik | 0.237 |
| rasa4 | 0.237 |
| dusp14 | 0.236 |
| rusc1 | 0.236 |
| srgap2 | 0.236 |
| ephb2 | 0.236 |
| h36 | 0.236 |
| d12mit143 | 0.236 |
| ctdspl | 0.236 |
| 2310014h01rik | 0.236 |
| pcdh10 | 0.236 |
| uck1 | 0.236 |
| dusp19 | 0.235 |
| st5 | 0.235 |
| ksr1 | 0.235 |
| rdx | 0.235 |
| ephb6 | 0.235 |
| gipc2 | 0.235 |
| pcdhga12 | 0.235 |
| exoc2 | 0.235 |
| ralb | 0.235 |
| tssk4 | 0.234 |
| tiprl | 0.234 |
| dscaml1 | 0.234 |
| rps6ka2 | 0.234 |
| pear1 | 0.234 |
| mia3 | 0.234 |
| tssk1 | 0.234 |
| mtmr12 | 0.234 |
| d0h4s114 | 0.234 |
| col22a1 | 0.234 |
| nek11 | 0.234 |
| arf6 | 0.234 |
| 2610019a05rik | 0.233 |
| mapk11 | 0.233 |
| lrrn3 | 0.233 |
| adam28 | 0.233 |
| dyrk2 | 0.233 |
| astn1 | 0.233 |
| 6330500d04rik | 0.233 |
| def6 | 0.233 |
| kndc1 | 0.233 |
| spry3 | 0.233 |
| cdh15 | 0.233 |
| riok1 | 0.233 |
| adam12 | 0.233 |
| mylk3 | 0.233 |
| cyr61 | 0.232 |
| pik3cb | 0.232 |
| cmpk2 | 0.232 |
| gna13 | 0.232 |
| arfip2 | 0.232 |
| sh2b1 | 0.232 |
| pik3r3 | 0.232 |
| cdkl1 | 0.232 |
| clk1 | 0.232 |
| epb4.1l2 | 0.232 |
| itga4 | 0.232 |
| prkrir | 0.232 |
| tmod3 | 0.232 |
| map4k2 | 0.232 |
| umpk-ps | 0.232 |
