# Supplementary material for: GIT2 Acts as a Potential Keystone Protein in Functional Hypothalamic Networks Associated with Age-Related Phenotypic Changes in Rats
Source: PLoS One. 2012 May 14;7(5):e36975. doi: 10.1371/journal.pone.0036975 (PMC3351446; doi:10.1371/journal.pone.0036975)
Supplement: Table S13 — GeneIndexer latent semantic indexing (LSI) of significantly-regulated ‘Gap junction’ KEGG pathway. Using the KEGG signaling pathway ‘Gap junction’ as an input term, a list of the top 1000 implicitly-correlated (LSI correlation score >0.1) was generated using a full genome background list. (DOC) [file pone.0036975.s017.doc]

**Table S13. GeneIndexer latent semantic indexing (LSI) of significantly-regulated ‘Gap junction’ KEGG pathway.** Using the KEGG signaling pathway ‘Gap junction’ as an input term, a list of the top 1000 implicitly-correlated (LSI correlation score >0.1) was generated using a full genome background list.

| ***Gap junction*** |  |
| --- | --- |
|  |  |
| **Symbol** | **LSI correlation score** |
| d3tu51 | 0.561 |
| gjd4 | 0.554 |
| gja10 | 0.541 |
| gjd3 | 0.531 |
| gjb5 | 0.528 |
| gja6 | 0.521 |
| panx3 | 0.518 |
| gje1 | 0.513 |
| gjc1 | 0.505 |
| c030046e11rik | 0.502 |
| gjc2 | 0.501 |
| gjb4 | 0.485 |
| gja3 | 0.467 |
| tjp3 | 0.465 |
| elmod1 | 0.443 |
| sgsm3 | 0.438 |
| gjb3 | 0.43 |
| 1700006a11rik | 0.423 |
| gjb1 | 0.417 |
| panx2 | 0.416 |
| gja8 | 0.415 |
| gjd2 | 0.411 |
| cgn | 0.399 |
| tjp1 | 0.392 |
| tjp2 | 0.39 |
| centd1 | 0.39 |
| ebo | 0.389 |
| gja5 | 0.384 |
| micall1 | 0.382 |
| cldn17 | 0.379 |
| gja4 | 0.376 |
| gjb6 | 0.367 |
| cgnl1 | 0.366 |
| cldn14 | 0.366 |
| elmod2 | 0.358 |
| panx1 | 0.353 |
| rab13 | 0.353 |
| centb2 | 0.353 |
| mtv14 | 0.344 |
| d3mit39 | 0.343 |
| d3mit76 | 0.343 |
| d3mit74 | 0.343 |
| d3mit233 | 0.343 |
| tmem204 | 0.341 |
| ocln | 0.337 |
| micall2 | 0.337 |
| cldn5 | 0.329 |
| dmxl2 | 0.328 |
| arhgap17 | 0.325 |
| gjb2 | 0.324 |
| cldn9 | 0.324 |
| tjap1 | 0.319 |
| b230339m05rik | 0.309 |
| cldn10 | 0.308 |
| cldn19 | 0.306 |
| v1ra5 | 0.306 |
| cldn12 | 0.305 |
| centg2 | 0.304 |
| arfgap3 | 0.304 |
| cldn15 | 0.302 |
| pkp2 | 0.3 |
| igsf5 | 0.3 |
| marveld2 | 0.298 |
| mpdz | 0.295 |
| trim23 | 0.294 |
| arfgap1 | 0.294 |
| rasa2 | 0.293 |
| centd2 | 0.292 |
| cldn1 | 0.289 |
| loc667655 | 0.289 |
| cldn8 | 0.288 |
| rmnd5a | 0.288 |
| wdr7 | 0.281 |
| smap2 | 0.28 |
| tagap1 | 0.277 |
| arhgap10 | 0.277 |
| ddef2 | 0.276 |
| a230067g21rik | 0.276 |
| tbc1d30 | 0.274 |
| inadl | 0.273 |
| plekha7 | 0.27 |
| ctnna1 | 0.27 |
| arf5 | 0.269 |
| pkp4 | 0.264 |
| rab20 | 0.263 |
| sympk | 0.261 |
| rasa3 | 0.26 |
| amotl1 | 0.259 |
| ma | 0.257 |
| mllt4 | 0.255 |
| arhgap21 | 0.254 |
| ighd1-1 | 0.253 |
| cldn6 | 0.253 |
| epb4.1l5 | 0.252 |
| copz1 | 0.251 |
| ddefl1 | 0.251 |
| rab43 | 0.25 |
| abr | 0.25 |
| gm1123 | 0.25 |
| cldn2 | 0.249 |
| arhgap9 | 0.247 |
| gen1 | 0.246 |
| centd3 | 0.245 |
| cldn13 | 0.245 |
| centb1 | 0.243 |
| 2310057j16rik | 0.242 |
| lth1 | 0.242 |
| arf1 | 0.242 |
| gbp1 | 0.242 |
| 3110043j09rik | 0.242 |
| dsp | 0.242 |
| tbc1d25 | 0.241 |
| rab8b | 0.241 |
| git2 | 0.241 |
| sh3bp1 | 0.241 |
| bc065397 | 0.24 |
| zfyve1 | 0.238 |
| pard6b | 0.238 |
| ctnna3 | 0.237 |
| gja1 | 0.237 |
| ddef1 | 0.237 |
| arhgap26 | 0.237 |
| cntnap1 | 0.237 |
| arf6 | 0.236 |
| arhgap29 | 0.236 |
| ctnna2 | 0.236 |
| cdgap | 0.235 |
| arcn1 | 0.235 |
| arhgap5 | 0.233 |
| rnd2 | 0.232 |
| arfgap2 | 0.231 |
| mpp5 | 0.231 |
| arhgap1 | 0.231 |
| snx26 | 0.23 |
| tbc1d5 | 0.23 |
| cldn11 | 0.229 |
| arhgap20 | 0.229 |
| rapgef6 | 0.228 |
| jam2 | 0.227 |
| cope | 0.227 |
| scoc | 0.227 |
| rapgef2 | 0.226 |
| mirn206 | 0.226 |
| rasa4 | 0.226 |
| grit | 0.225 |
| rhod | 0.224 |
| pard3 | 0.224 |
| zfp280b | 0.223 |
| zfp279 | 0.223 |
| copb2 | 0.223 |
| arl3 | 0.222 |
| rnd1 | 0.222 |
| copg | 0.221 |
| col22a1 | 0.221 |
| mon2 | 0.22 |
| tcrb-v16 | 0.22 |
| tcrb-v5.2 | 0.22 |
| rasa1 | 0.219 |
| iqsec1 | 0.219 |
| cldn7 | 0.218 |
| rhof | 0.218 |
| f11r | 0.218 |
| dock6 | 0.217 |
| loc677654 | 0.216 |
| arhgap24 | 0.215 |
| rab3gap2 | 0.215 |
| cdh5 | 0.214 |
| rgnef | 0.214 |
| arhgap15 | 0.214 |
| git1 | 0.213 |
| cldn4 | 0.213 |
| crb3 | 0.213 |
| amot | 0.212 |
| herc1 | 0.212 |
| tmem47 | 0.211 |
| cdc42ep4 | 0.21 |
| d10ertd610e | 0.21 |
| vezt | 0.21 |
| rgs14 | 0.21 |
| cldn3 | 0.21 |
| arfgef1 | 0.21 |
| cdc42ep5 | 0.209 |
| rin2 | 0.209 |
| jup | 0.206 |
| esam1 | 0.206 |
| rgl1 | 0.206 |
| arf3 | 0.205 |
| ralgps2 | 0.205 |
| arhgap12 | 0.205 |
| arvcf | 0.205 |
| arl2 | 0.204 |
| rala | 0.204 |
| arhgap4 | 0.204 |
| pard6g | 0.204 |
| dsc1 | 0.204 |
| grlf1 | 0.203 |
| arfrp1 | 0.203 |
| arl1 | 0.203 |
| tbc1d15 | 0.203 |
| copz2 | 0.203 |
| dsc2 | 0.203 |
| csda | 0.203 |
| copb1 | 0.202 |
| rab3gap1 | 0.202 |
| iqgap1 | 0.201 |
| rab34 | 0.2 |
| ralgps1 | 0.2 |
| bc016423 | 0.199 |
| diras2 | 0.199 |
| cldn18 | 0.198 |
| mpp7 | 0.198 |
| cdc42ep3 | 0.198 |
| pscd4 | 0.197 |
| amotl2 | 0.197 |
| arhgap27 | 0.197 |
| copa | 0.196 |
| dsg2 | 0.196 |
| arhgef7 | 0.196 |
| cdh11 | 0.196 |
| rap1a | 0.194 |
| magi1 | 0.194 |
| dock11 | 0.194 |
| gdi1 | 0.194 |
| gbf1 | 0.193 |
| arl2bp | 0.193 |
| arl5b | 0.193 |
| pscd2 | 0.193 |
| gapvd1 | 0.193 |
| racgap1 | 0.193 |
| arhgef1 | 0.192 |
| ubn1 | 0.192 |
| arl4c | 0.192 |
| kalrn | 0.192 |
| rab8a | 0.192 |
| nefl | 0.191 |
| jam3 | 0.191 |
| centg3 | 0.19 |
| sipa1 | 0.19 |
| loc100040794 | 0.19 |
| stard13 | 0.19 |
| pscd1 | 0.19 |
| ssx2ip | 0.189 |
| erbb2ip | 0.189 |
| 9030409g11rik | 0.189 |
| ottmusg00000002180 | 0.189 |
| arhgef12 | 0.188 |
| rabgef1 | 0.188 |
| rab1b | 0.188 |
| trio | 0.188 |
| psd2 | 0.187 |
| agrn | 0.187 |
| dsg1a | 0.187 |
| kbtbd11 | 0.187 |
| rnd3 | 0.186 |
| arl6ip6 | 0.186 |
| ctnnd1 | 0.186 |
| a130090k04rik | 0.185 |
| fgd4 | 0.184 |
| rgs19 | 0.184 |
| cdh2 | 0.184 |
| arf2 | 0.184 |
| arf4 | 0.183 |
| musk | 0.183 |
| rheb | 0.182 |
| mras | 0.182 |
| rab35 | 0.182 |
| prkcz2 | 0.182 |
| rab5a | 0.182 |
| dok7 | 0.182 |
| arl5a | 0.182 |
| cbll1 | 0.181 |
| rap2a | 0.181 |
| arhgdig | 0.18 |
| rhoq | 0.18 |
| rras | 0.18 |
| cdc42ep2 | 0.18 |
| rapsn | 0.18 |
| dsc3 | 0.18 |
| rgs16 | 0.179 |
| bves | 0.179 |
| 9030425e11rik | 0.179 |
| rabgap1 | 0.178 |
| ctnnd2 | 0.178 |
| isoc2a | 0.178 |
| rab12 | 0.178 |
| nefh | 0.178 |
| rap1gap | 0.178 |
| igl-2 | 0.177 |
| centa1 | 0.177 |
| arfip1 | 0.177 |
| scrib | 0.176 |
| rab1 | 0.176 |
| tbc1d20 | 0.175 |
| pcdha3 | 0.175 |
| rhou | 0.175 |
| pcdh12 | 0.175 |
| kif13b | 0.175 |
| chn2 | 0.174 |
| syx1 | 0.174 |
| syx2 | 0.174 |
| rab6ip1 | 0.174 |
| shroom2 | 0.174 |
| arl4d | 0.174 |
| ankrd44 | 0.173 |
| pgam2 | 0.173 |
| rraga | 0.173 |
| amica1 | 0.173 |
| shroom4 | 0.173 |
| rgs10 | 0.173 |
| bhlhb8 | 0.172 |
| arl4a | 0.172 |
| fgd3 | 0.172 |
| vcl | 0.172 |
| ralb | 0.171 |
| rab28 | 0.171 |
| arhgef10 | 0.171 |
| kdelr1 | 0.17 |
| smap1 | 0.17 |
| exoc2 | 0.17 |
| tsc1 | 0.169 |
| hmcn1 | 0.169 |
| pscd3 | 0.169 |
| rin3 | 0.169 |
| cdc42ep1 | 0.169 |
| rab24 | 0.169 |
| rabep1 | 0.169 |
| arfip2 | 0.168 |
| pvrl3 | 0.168 |
| pard3b | 0.168 |
| bnip2 | 0.168 |
| rac3 | 0.168 |
| myo9a | 0.168 |
| arhgef6 | 0.168 |
| pard6a | 0.167 |
| cldn16 | 0.167 |
| rab40c | 0.167 |
| tbc1d10a | 0.167 |
| rab11fip3 | 0.167 |
| cxadr | 0.167 |
| mpz | 0.166 |
| mcf2 | 0.166 |
| rragb | 0.166 |
| snx13 | 0.166 |
| rgs20 | 0.166 |
| pkp1 | 0.165 |
| centg1 | 0.165 |
| shroom1 | 0.165 |
| rab21 | 0.165 |
| arl6 | 0.165 |
| rab3il1 | 0.164 |
| rabif | 0.164 |
| rgs17 | 0.164 |
| gem | 0.164 |
| fchsd2 | 0.164 |
| iqsec2 | 0.163 |
| ermn | 0.163 |
| arhgef18 | 0.163 |
| mcf2l | 0.163 |
| cdh15 | 0.163 |
| sipa1l1 | 0.163 |
| plekhg2 | 0.163 |
| iqgap2 | 0.163 |
| ralgds | 0.162 |
| iqgap3 | 0.162 |
| fgd2 | 0.162 |
| gmip | 0.162 |
| cdh24 | 0.162 |
| chn1 | 0.162 |
| psd4 | 0.162 |
| dchs1 | 0.162 |
| igkv10-96 | 0.161 |
| rasl11a | 0.161 |
| dock9 | 0.161 |
| rasal1 | 0.161 |
| plekhg5 | 0.161 |
| lgl | 0.161 |
| dnmbp | 0.16 |
| arhgdia | 0.16 |
| pak3 | 0.16 |
| rab5b | 0.16 |
| ajap1 | 0.16 |
| tiam1 | 0.16 |
| magi2 | 0.16 |
| rab11fip4 | 0.159 |
| rras2 | 0.159 |
| phf5a | 0.159 |
| rgs12 | 0.158 |
| iqsec3 | 0.158 |
| rab2a | 0.158 |
| dbn1 | 0.157 |
| tbcd | 0.157 |
| gna13 | 0.157 |
| chrne | 0.157 |
| spna2 | 0.157 |
| rgs8 | 0.156 |
| rrad | 0.156 |
| rhog | 0.156 |
| rab10 | 0.156 |
| d8ertd82e | 0.156 |
| cdh10 | 0.156 |
| ankrd27 | 0.156 |
| nf1 | 0.156 |
| csk | 0.156 |
| farp2 | 0.155 |
| arfgef2 | 0.155 |
| gdi2 | 0.155 |
| rap1gds1 | 0.155 |
| dock10 | 0.155 |
| rundc3a | 0.155 |
| rusc2 | 0.155 |
| lnx1 | 0.155 |
| rgs11 | 0.155 |
| rit1 | 0.154 |
| arl6ip2 | 0.154 |
| arhgef2 | 0.154 |
| rtkn | 0.153 |
| ect2 | 0.153 |
| rlf | 0.153 |
| magi3 | 0.153 |
| arl9 | 0.153 |
| arl10 | 0.153 |
| rgs7 | 0.153 |
| tsc2 | 0.153 |
| rab3a | 0.153 |
| nrxn3 | 0.153 |
| yif1a | 0.152 |
| farp1 | 0.152 |
| rragc | 0.152 |
| d3mit284 | 0.152 |
| prkcc | 0.152 |
| tbc1d10c | 0.151 |
| arhgef10l | 0.151 |
| pxn | 0.151 |
| rab33b | 0.151 |
| chrna1 | 0.15 |
| stard8 | 0.15 |
| gna12 | 0.15 |
| ap3s2 | 0.15 |
| rgl3 | 0.15 |
| plxnb1 | 0.15 |
| rab4a | 0.149 |
| pmp22 | 0.149 |
| 4930511j11rik | 0.148 |
| tiam2 | 0.148 |
| arhgap6 | 0.148 |
| arhgef11 | 0.148 |
| nrxn2 | 0.148 |
| arhgap22 | 0.148 |
| rap2b | 0.148 |
| ubqln4 | 0.148 |
| rgs1 | 0.147 |
| fgd1 | 0.147 |
| rhobtb3 | 0.147 |
| nefm | 0.147 |
| hsd3b | 0.147 |
| plekhg6 | 0.146 |
| rgl2 | 0.146 |
| rabac1 | 0.146 |
| arhgap19 | 0.146 |
| d3mit43 | 0.146 |
| myom2 | 0.146 |
| pkn3 | 0.145 |
| gnao1 | 0.145 |
| shroom3 | 0.145 |
| diras1 | 0.145 |
| prx | 0.145 |
| sp4 | 0.145 |
| prl7d1 | 0.145 |
| 5730596k20rik | 0.144 |
| colq | 0.144 |
| btbd12 | 0.144 |
| ap3s1 | 0.144 |
| arhgef9 | 0.143 |
| arl13b | 0.143 |
| mpmv1 | 0.143 |
| rit2 | 0.142 |
| itsn1 | 0.142 |
| cask | 0.142 |
| chrnd | 0.142 |
| dock4 | 0.142 |
| rgs3 | 0.142 |
| gnaz | 0.142 |
| gcna1 | 0.141 |
| pde6d | 0.141 |
| rgs4 | 0.141 |
| rgs9bp | 0.14 |
| gnai1 | 0.14 |
| pkp3 | 0.14 |
| ank3 | 0.14 |
| dsg1b | 0.14 |
| ric8 | 0.14 |
| rap2c | 0.14 |
| gnai3 | 0.14 |
| rasgrf1 | 0.14 |
| arhgef19 | 0.14 |
| chrng | 0.14 |
| frmd4b | 0.139 |
| tcrd-v4 | 0.138 |
| mtmr2 | 0.138 |
| rhoc | 0.138 |
| tcrb-v7 | 0.138 |
| gga1 | 0.138 |
| frmpd4 | 0.138 |
| srgap3 | 0.138 |
| psd | 0.137 |
| sarb | 0.137 |
| dopey1 | 0.137 |
| 2210009g21rik | 0.137 |
| rab9 | 0.137 |
| rapgef3 | 0.137 |
| arhgdib | 0.137 |
| 4631416l12rik | 0.137 |
| poll | 0.137 |
| om | 0.137 |
| des | 0.137 |
| mynn | 0.136 |
| arl8a | 0.136 |
| dsg3 | 0.136 |
| ptk2b | 0.136 |
| igl-v3 | 0.136 |
| yes1 | 0.136 |
| arhgef3 | 0.136 |
| tbx5 | 0.136 |
| rab6 | 0.136 |
| rab3b | 0.136 |
| ttc1 | 0.135 |
| usp6nl | 0.135 |
| garnl4 | 0.135 |
| syngap1 | 0.135 |
| plekha2 | 0.135 |
| myl2 | 0.135 |
| myo9b | 0.135 |
| ric8b | 0.135 |
| rab6b | 0.134 |
| kif9 | 0.134 |
| exoc4 | 0.134 |
| rhpn1 | 0.134 |
| rem2 | 0.134 |
| jph1 | 0.133 |
| nf2 | 0.133 |
| rhov | 0.133 |
| chrnb1 | 0.133 |
| gpsm3 | 0.133 |
| syp | 0.133 |
| gna14 | 0.133 |
| phldb2 | 0.133 |
| mip | 0.133 |
| rhebl1 | 0.133 |
| cdh18 | 0.133 |
| gnb5 | 0.133 |
| klhl20 | 0.132 |
| net1 | 0.132 |
| jph2 | 0.132 |
| rab22a | 0.132 |
| cntnap2 | 0.132 |
| pvalb | 0.132 |
| myo1f | 0.132 |
| rab30 | 0.132 |
| plce1 | 0.132 |
| tnk2 | 0.132 |
| rasgrf2 | 0.132 |
| gnaq | 0.131 |
| cadm2 | 0.131 |
| g3bp1 | 0.131 |
| xirp2 | 0.131 |
| depdc2 | 0.131 |
| rab11b | 0.131 |
| 6-Sep | 0.131 |
| rhoj | 0.131 |
| pld4 | 0.13 |
| gna11 | 0.13 |
| arhgef15 | 0.13 |
| tmed2 | 0.13 |
| tbc1d7 | 0.13 |
| tg(krt14-cre)1efu | 0.13 |
| rasgef1b | 0.13 |
| pvrl1 | 0.13 |
| gprin3 | 0.13 |
| gbp2 | 0.13 |
| rab5c | 0.129 |
| sorbs3 | 0.129 |
| zfp286 | 0.129 |
| ng23 | 0.129 |
| nhs | 0.129 |
| rbj | 0.129 |
| nlgn3 | 0.129 |
| dnaja3 | 0.129 |
| nrxn1 | 0.129 |
| plekha8 | 0.128 |
| cnksr2 | 0.128 |
| pgm5 | 0.128 |
| rapgefl1 | 0.128 |
| rgs5 | 0.128 |
| cdc42l2 | 0.128 |
| cdc42l1 | 0.128 |
| cdc42l3 | 0.128 |
| sergef | 0.128 |
| dtna | 0.128 |
| cdc42 | 0.128 |
| rhobtb1 | 0.128 |
| rap1b | 0.127 |
| rgs6 | 0.127 |
| copg2 | 0.127 |
| osgin2 | 0.127 |
| kirrel | 0.127 |
| cdh12 | 0.127 |
| calb1 | 0.127 |
| ngef | 0.127 |
| rapgef5 | 0.127 |
| rnr1 | 0.126 |
| crk | 0.126 |
| nfasc | 0.126 |
| rab3c | 0.126 |
| bcar1 | 0.125 |
| spnb4 | 0.125 |
| sdcbp | 0.125 |
| cntfr | 0.125 |
| fat2 | 0.125 |
| arhgap25 | 0.125 |
| pak1 | 0.125 |
| dsg1c | 0.125 |
| rangrf | 0.125 |
| nck1 | 0.124 |
| sync | 0.124 |
| rab14 | 0.124 |
| ccpg1 | 0.124 |
| cdh3 | 0.124 |
| angpt4 | 0.124 |
| gcc2 | 0.124 |
| pld1 | 0.123 |
| rab11a | 0.123 |
| utrn | 0.123 |
| gga3 | 0.123 |
| mpp2 | 0.123 |
| nov | 0.123 |
| rabggtb | 0.123 |
| ap4e1 | 0.123 |
| gprin1 | 0.123 |
| pclo | 0.123 |
| e130112l23rik | 0.123 |
| rab9b | 0.123 |
| lima1 | 0.123 |
| d13mit91 | 0.123 |
| mnx1 | 0.123 |
| slc2a1 | 0.123 |
| d1ertd57e | 0.123 |
| sar1a | 0.122 |
| elmo1 | 0.122 |
| ai507611 | 0.122 |
| ottmusg00000005491 | 0.122 |
| 4933417m04rik | 0.122 |
| 4921509e07rik | 0.122 |
| 1700021p22rik | 0.122 |
| cit | 0.122 |
| pip5k1c | 0.122 |
| brd8 | 0.122 |
| pscdbp | 0.122 |
| rgs9 | 0.122 |
| pcdhga3 | 0.121 |
| fbxo8 | 0.121 |
| d3mit101 | 0.121 |
| zfp2 | 0.121 |
| pdzd2 | 0.121 |
| pak2 | 0.121 |
| exoc1 | 0.12 |
| erp29 | 0.12 |
| rgs2 | 0.12 |
| gng2 | 0.12 |
| rab15 | 0.12 |
| gna15 | 0.12 |
| gbp3 | 0.12 |
| mp | 0.119 |
| sorbs1 | 0.119 |
| pak1ip1 | 0.119 |
| bcar3 | 0.119 |
| exoc5 | 0.119 |
| tcrd-v | 0.119 |
| cdh4 | 0.119 |
| rsu1 | 0.119 |
| rhob | 0.119 |
| bc067047 | 0.119 |
| col13a1 | 0.119 |
| rdx | 0.118 |
| dock1 | 0.118 |
| zbtb33 | 0.118 |
| ptprm | 0.118 |
| lmo7 | 0.118 |
| au040829 | 0.118 |
| golga4 | 0.118 |
| opalin | 0.118 |
| plekha3 | 0.118 |
| grin2c | 0.117 |
| xirp1 | 0.117 |
| pak4 | 0.117 |
| mpp4 | 0.117 |
| mpp6 | 0.117 |
| gga2 | 0.117 |
| hmgn1-rs15 | 0.117 |
| mtv54 | 0.117 |
| pde6h | 0.117 |
| tcrb-v10 | 0.117 |
| tcrb-v15 | 0.117 |
| dgkg | 0.117 |
| foxj2 | 0.117 |
| pcdha4 | 0.117 |
| gpsm2 | 0.117 |
| syn1 | 0.117 |
| polb | 0.116 |
| dst | 0.116 |
| exoc6b | 0.116 |
| tcrd-v2 | 0.116 |
| prkcb1 | 0.116 |
| zbtb12 | 0.116 |
| gnai2 | 0.116 |
| vav2 | 0.116 |
| rab3d | 0.116 |
| rin1 | 0.116 |
| bv | 0.116 |
| plcb1 | 0.116 |
| shank3 | 0.116 |
| nlgn1 | 0.116 |
| rangap1 | 0.115 |
| rcbtb2 | 0.115 |
| sypl2 | 0.115 |
| abl2 | 0.115 |
| lin7a | 0.115 |
| tbc1d4 | 0.115 |
| neuna60 | 0.115 |
| chat | 0.115 |
| myl7 | 0.115 |
| tcrg-v | 0.115 |
| gimap6 | 0.115 |
| gpsm1 | 0.115 |
| zfyve20 | 0.115 |
| fen1 | 0.115 |
| fes | 0.114 |
| pfkp | 0.114 |
| pvrl4 | 0.114 |
| rims3 | 0.114 |
| polm | 0.114 |
| gvin1 | 0.114 |
| nbea | 0.114 |
| dock5 | 0.114 |
| pdzrn3 | 0.113 |
| cdh9 | 0.113 |
| 1100001e04rik | 0.113 |
| lrrc7 | 0.113 |
| snta1 | 0.113 |
| cnksr1 | 0.113 |
| akap13 | 0.113 |
| shank2 | 0.113 |
| gm1077 | 0.113 |
| sh2d3c | 0.113 |
| nrap | 0.113 |
| arl14 | 0.113 |
| rragd | 0.113 |
| mapk7 | 0.113 |
| plcb3 | 0.113 |
| vapa | 0.113 |
| tbx3 | 0.113 |
| rapgef1 | 0.113 |
| arl8b | 0.113 |
| wdr44 | 0.113 |
| gprin2 | 0.112 |
| zfp106 | 0.112 |
| rab7 | 0.112 |
| wtip | 0.112 |
| eea1 | 0.112 |
| a630055g03rik | 0.112 |
| ap3m1 | 0.112 |
| csnk1d | 0.112 |
| calb2 | 0.112 |
| hck | 0.112 |
| pcp2 | 0.112 |
| fbf1 | 0.112 |
| d930005d10rik | 0.112 |
| mical1 | 0.111 |
| d7mit357 | 0.111 |
| tcrd-v7 | 0.111 |
| rac2 | 0.111 |
| nexn | 0.111 |
| scn5a | 0.11 |
| arhgef4 | 0.11 |
| cd2ap | 0.11 |
| ubxd5 | 0.11 |
| tcrb-v1 | 0.11 |
| strn4 | 0.11 |
| smpx | 0.11 |
| prkci | 0.11 |
| caskin2 | 0.11 |
| 11-Sep | 0.11 |
| ptpn14 | 0.109 |
| arpc2 | 0.109 |
| pik3r1 | 0.109 |
| arhgef17 | 0.109 |
| tbc1d1 | 0.109 |
| sv2a | 0.109 |
| tcrd-v6 | 0.109 |
| gnat1 | 0.109 |
| sec31a | 0.109 |
| elmo2 | 0.109 |
| rab7l1 | 0.109 |
| ren1 | 0.109 |
| d4mit339 | 0.109 |
| dok2 | 0.108 |
| sh3kbp1 | 0.108 |
| ralbp1 | 0.108 |
| dlg3 | 0.108 |
| pld2 | 0.108 |
| golga7 | 0.108 |
| fgf8 | 0.108 |
| srgap2 | 0.108 |
| nphs1 | 0.108 |
| cdh16 | 0.108 |
| baf | 0.108 |
| tbx2 | 0.108 |
| ocrl | 0.108 |
| lin7c | 0.108 |
| bcr | 0.107 |
| gipc1 | 0.107 |
| d3mit244 | 0.107 |
| rufy1 | 0.107 |
| cdc42se1 | 0.107 |
| nlgn2 | 0.107 |
| macf1 | 0.107 |
| ahnak | 0.107 |
| dok1 | 0.107 |
| eps8 | 0.107 |
| dlc1 | 0.107 |
| srprb | 0.107 |
| cdc42bpb | 0.107 |
| dlgap1 | 0.107 |
| ms10s | 0.107 |
| ppl | 0.106 |
| gba | 0.106 |
| gal3st1 | 0.106 |
| pcdhgb4 | 0.106 |
| pcdh9 | 0.106 |
| ap3b2 | 0.106 |
| jsrp1 | 0.106 |
| rgs7bp | 0.106 |
| p2rx7 | 0.106 |
| rmi1 | 0.106 |
| rph3a | 0.106 |
| zfp639 | 0.106 |
| obscn | 0.105 |
| apold1 | 0.105 |
| eme1 | 0.105 |
| a630098a13rik | 0.105 |
| myom1 | 0.105 |
| caskin1 | 0.105 |
| dmpk | 0.105 |
| eg665419 | 0.104 |
| tubb2a | 0.104 |
| gap43 | 0.104 |
| plcb4 | 0.104 |
| ranbp1 | 0.104 |
| rab3ip | 0.104 |
| ank2 | 0.104 |
| rem1 | 0.104 |
| shc4 | 0.104 |
| def6 | 0.103 |
| mylk3 | 0.103 |
| nipsnap1 | 0.103 |
| rhoa | 0.103 |
| gnb1 | 0.103 |
| plcg1 | 0.103 |
| cdh8 | 0.103 |
| mag | 0.103 |
| grasp | 0.103 |
| pdgfrb | 0.103 |
| 4933428g20rik | 0.102 |
| cercam | 0.102 |
| trpt1 | 0.102 |
| gng4 | 0.102 |
| nol8 | 0.102 |
| ctnnal1 | 0.102 |
| cttn | 0.102 |
| kif20a | 0.102 |
| cltc | 0.102 |
| egf | 0.102 |
| fchsd1 | 0.102 |
| spata13 | 0.102 |
| src | 0.101 |
| mneu | 0.101 |
| emx1 | 0.101 |
| als2cl | 0.101 |
| fat1 | 0.101 |
| gng5 | 0.101 |
| gcc1 | 0.101 |
| plxnd1 | 0.101 |
| mus81 | 0.101 |
| tax1bp3 | 0.101 |
| d3mit137 | 0.101 |
| jund | 0.101 |
| fert2 | 0.101 |
| ywhae | 0.101 |
| d6kcc1 | 0.101 |
| ficd | 0.101 |
| ezr | 0.101 |
| prkaca | 0.101 |
| d19mit17 | 0.101 |
| celsr1 | 0.101 |
| plxnb3 | 0.101 |
| blm | 0.101 |
| gimap7 | 0.101 |
| baiap2 | 0.101 |
| capzb | 0.101 |
| chm | 0.101 |
| rab17 | 0.101 |
| mif-ps9 | 0.101 |
| sdk2 | 0.101 |
| scn1b | 0.101 |
| d1mit388 | 0.101 |
| rab11fip2 | 0.101 |
| lig3 | 0.101 |
| ras | 0.101 |
| eif5 | 0.101 |
| fyn | 0.101 |
| krit1 | 0.1 |
| litaf | 0.1 |
| pklr | 0.1 |
| rhot1 | 0.1 |
| nol1 | 0.1 |
| d1mit538 | 0.1 |
| gimap9 | 0.1 |
| epha2 | 0.1 |
| dep | 0.1 |
| exoc3 | 0.1 |
| ywhab | 0.1 |
| lyn | 0.1 |
| d14mit156 | 0.1 |
| p2rx1 | 0.1 |
| ins1 | 0.1 |
| gimap8 | 0.1 |
| pvrl2 | 0.1 |
| 2-Sep | 0.1 |
| efna1 | 0.1 |
| lim2 | 0.1 |
| uso1 | 0.1 |
| d12mit191 | 0.1 |
| spnb2 | 0.1 |
| ocm | 0.1 |
| unc84b | 0.1 |
| mpp3 | 0.1 |
| als2 | 0.1 |
| cntn5 | 0.1 |
| rab33a | 0.1 |
| gng3 | 0.1 |
| ap1g1 | 0.1 |
| rbm35a | 0.1 |
| lin7b | 0.1 |
| gnl2 | 0.1 |
| anxa6 | 0.1 |
| schip1 | 0.1 |
| chml | 0.1 |
| dlgap4 | 0.1 |
| epb4.9 | 0.1 |
| ophn1 | 0.1 |
| lama5 | 0.1 |
| eg226654 | 0.1 |
| sntb2 | 0.1 |
| begain | 0.1 |
| syne1 | 0.1 |
| icmt | 0.1 |
| rhpn2 | 0.1 |
| d15mit72 | 0.1 |
| b230208h17rik | 0.1 |
| dlgap3 | 0.1 |
| pde6g | 0.1 |
| rasgrp3 | 0.1 |
| dlg1 | 0.1 |
| gnb2 | 0.1 |
| nck2 | 0.1 |
| poli | 0.1 |
| rab31 | 0.1 |
| rhoh | 0.1 |
| cdc42bpa | 0.1 |
| d10wsu52e | 0.1 |
| sema4f | 0.1 |
| upf3b | 0.1 |
| nutf2-ps1 | 0.1 |
| bin3 | 0.1 |
| ptpn13 | 0.1 |
| gdf9 | 0.1 |
| zfp1 | 0.1 |
| ooep | 0.1 |
| d1mit103 | 0.1 |
| sec16a | 0.1 |
| gnl3l | 0.1 |
| khdrbs1 | 0.1 |
| exoc7 | 0.1 |
| grin1 | 0.1 |
| golga2 | 0.1 |
| msh5 | 0.1 |
| rimbp2 | 0.1 |
| ddn | 0.1 |
| erc2 | 0.1 |
| 9930111j21rik | 0.1 |
| aqp10p | 0.1 |
