# Supplementary material for: GIT2 Acts as a Potential Keystone Protein in Functional Hypothalamic Networks Associated with Age-Related Phenotypic Changes in Rats
Source: PLoS One. 2012 May 14;7(5):e36975. doi: 10.1371/journal.pone.0036975 (PMC3351446; doi:10.1371/journal.pone.0036975)
Supplement: Table S14 — GeneIndexer latent semantic indexing (LSI) of significantly-regulated ‘GnRH signaling’ KEGG pathway. Using the KEGG signaling pathway ‘GnRH signaling’ as an input term, a list of the top 1000 implicitly-correlated (LSI correlation score >0.1) was generated using a full genome background list. (DOC) [file pone.0036975.s018.doc]

**Table S14. GeneIndexer latent semantic indexing (LSI) of significantly-regulated ‘GnRH signaling’ KEGG pathway.** Using the KEGG signaling pathway ‘GnRH signaling’ as an input term, a list of the top 1000 implicitly-correlated (LSI correlation score >0.1) was generated using a full genome background list.

| ***GnRH signaling*** |  |
| --- | --- |
|  |  |
| **Protein Symbol** | **LSI correlation score** |
| gnrh1 | 0.547 |
| kiss1r | 0.529 |
| gnrhr | 0.523 |
| kiss1 | 0.478 |
| 6430527g18rik | 0.438 |
| fshb | 0.427 |
| v1ra2 | 0.423 |
| lhb | 0.423 |
| prokr2 | 0.421 |
| npvf | 0.388 |
| nelf | 0.377 |
| taf6l | 0.364 |
| v1ra1 | 0.357 |
| tra2a | 0.347 |
| prok2 | 0.345 |
| cga | 0.305 |
| aes | 0.304 |
| galp | 0.292 |
| fst | 0.288 |
| d15wsu169e | 0.282 |
| tle3 | 0.279 |
| tle2 | 0.278 |
| tle4 | 0.276 |
| lgr6 | 0.274 |
| pitx1 | 0.273 |
| maged1 | 0.272 |
| tpcn1 | 0.272 |
| magel2 | 0.268 |
| lhcgr | 0.267 |
| fshr | 0.261 |
| inha | 0.258 |
| tle1 | 0.258 |
| bmp15 | 0.252 |
| gpha2 | 0.25 |
| prokr1 | 0.249 |
| a230065h16rik | 0.248 |
| qrfp | 0.247 |
| inhbb | 0.247 |
| slc46a3 | 0.244 |
| tyro3 | 0.241 |
| d19mit104 | 0.241 |
| inhba | 0.239 |
| dlx2 | 0.239 |
| nms | 0.238 |
| igsf1 | 0.238 |
| dlx1 | 0.237 |
| scg2 | 0.235 |
| tg(fst)5zuk | 0.234 |
| ebf2 | 0.232 |
| dlx5 | 0.232 |
| nbl1 | 0.231 |
| pik3ap1 | 0.23 |
| inhbc | 0.226 |
| pcdha7 | 0.226 |
| pcdha10 | 0.226 |
| narg1 | 0.226 |
| 1110005a23rik | 0.226 |
| acvr2a | 0.224 |
| gphb5 | 0.22 |
| msx1 | 0.219 |
| hipk3 | 0.217 |
| v1rf2 | 0.216 |
| v1rf3 | 0.216 |
| v2r1 | 0.215 |
| vmn2r3 | 0.215 |
| pou2f1 | 0.214 |
| galr2 | 0.213 |
| dlx6 | 0.212 |
| utp14b | 0.212 |
| gm944 | 0.212 |
| vmn2r122 | 0.211 |
| axl | 0.211 |
| mtnr1a | 0.209 |
| rln3 | 0.209 |
| nell2 | 0.209 |
| pcdha12 | 0.207 |
| gabarapl1 | 0.206 |
| fstl3 | 0.205 |
| rabep2 | 0.205 |
| gal | 0.205 |
| cnpy1 | 0.205 |
| bambi-ps1 | 0.205 |
| msx2 | 0.205 |
| adcyap1 | 0.204 |
| dgkz | 0.203 |
| ottmusg00000005714 | 0.203 |
| amh | 0.202 |
| v1rb1 | 0.202 |
| nr0b1 | 0.202 |
| grem2 | 0.201 |
| lhx3 | 0.201 |
| paqr6 | 0.2 |
| paqr9 | 0.2 |
| ppp2r5d | 0.2 |
| rgs3 | 0.199 |
| gln3-1 | 0.199 |
| gdf9 | 0.198 |
| pmv11 | 0.198 |
| acvr1b | 0.198 |
| zfp709 | 0.197 |
| dlx6os1 | 0.197 |
| barx1 | 0.197 |
| rgs10 | 0.197 |
| nr5a1 | 0.197 |
| tg(fst)9zuk | 0.197 |
| tg(fst)10zuk | 0.197 |
| tg(fst)4zuk | 0.197 |
| tg(fst)7zuk | 0.197 |
| il17rd | 0.197 |
| gna11 | 0.196 |
| mtnr1b | 0.196 |
| pgr | 0.196 |
| anks1 | 0.196 |
| tmeff1 | 0.196 |
| epha5 | 0.195 |
| galr1 | 0.194 |
| olfr74 | 0.194 |
| lrp8 | 0.194 |
| ndn | 0.194 |
| trhr | 0.194 |
| pcdha5 | 0.193 |
| pcdha11 | 0.193 |
| au041133 | 0.193 |
| prok1 | 0.193 |
| afap1l2 | 0.193 |
| prph | 0.193 |
| rxfp1 | 0.193 |
| gpr103 | 0.192 |
| wdr26 | 0.191 |
| lrrfip2 | 0.191 |
| pcolce2 | 0.191 |
| msx1as | 0.19 |
| lpxn | 0.19 |
| gnaq | 0.189 |
| msx3 | 0.188 |
| vmn2r1 | 0.188 |
| egr4 | 0.188 |
| dlx3 | 0.188 |
| trhr2 | 0.188 |
| amhr2 | 0.187 |
| gpr151 | 0.187 |
| mertk | 0.187 |
| rgs13 | 0.187 |
| fosb | 0.187 |
| c330002i19rik | 0.186 |
| ift122 | 0.186 |
| nr5a2 | 0.186 |
| gper | 0.185 |
| camk2b | 0.185 |
| pcdha6 | 0.185 |
| lef1 | 0.185 |
| fezf1 | 0.185 |
| dok3 | 0.185 |
| galr3 | 0.184 |
| lyrm1 | 0.184 |
| ctf2 | 0.184 |
| shoc2 | 0.184 |
| ankrd6 | 0.183 |
| iapls3-10 | 0.183 |
| 1110032a04rik | 0.183 |
| rasd1 | 0.183 |
| pelp1 | 0.183 |
| bambi | 0.183 |
| rybp | 0.182 |
| ebf1 | 0.182 |
| paqr5 | 0.182 |
| gpr101 | 0.182 |
| lgr5 | 0.181 |
| v2r8 | 0.181 |
| sp9 | 0.181 |
| lzic | 0.181 |
| rnf138 | 0.18 |
| tac2 | 0.18 |
| rspo2 | 0.18 |
| d4mit304 | 0.18 |
| pcp4l1 | 0.18 |
| sorbs3 | 0.18 |
| crim2 | 0.18 |
| olfr63 | 0.18 |
| foxl2 | 0.179 |
| prlh | 0.179 |
| synj2bp | 0.179 |
| nr4a1 | 0.179 |
| tex261 | 0.179 |
| 4933433p14rik | 0.179 |
| sbno1 | 0.179 |
| zfp641 | 0.179 |
| sbno2 | 0.178 |
| aida | 0.178 |
| grr | 0.178 |
| acvr2b | 0.178 |
| v1rl1 | 0.177 |
| zfp653 | 0.177 |
| phlpp | 0.177 |
| rgs16 | 0.177 |
| olfr1 | 0.177 |
| 1190002h23rik | 0.177 |
| 9130404d14rik | 0.177 |
| lhx6 | 0.176 |
| dab1 | 0.176 |
| shf | 0.176 |
| dpy19l4 | 0.176 |
| b3gnt2 | 0.176 |
| nrip2 | 0.176 |
| d1mit508 | 0.175 |
| nmu | 0.175 |
| grit | 0.175 |
| sh2d3c | 0.175 |
| pak1ip1 | 0.175 |
| olfr937 | 0.175 |
| npw | 0.174 |
| stap1 | 0.174 |
| map4k1 | 0.174 |
| cmtm8 | 0.174 |
| spry4 | 0.174 |
| insl3 | 0.174 |
| olfr281 | 0.174 |
| olfr282 | 0.174 |
| olfr124 | 0.174 |
| olfr168 | 0.174 |
| cnksr1 | 0.174 |
| nkap | 0.173 |
| zbed3 | 0.173 |
| akirin1 | 0.173 |
| tubb-rs | 0.173 |
| c130060k24rik | 0.173 |
| ereg | 0.173 |
| lax1 | 0.173 |
| ddx54 | 0.172 |
| socs4 | 0.172 |
| hnl | 0.172 |
| otx1 | 0.172 |
| sh2d4a | 0.172 |
| il34 | 0.172 |
| ccdc88c | 0.171 |
| gprasp1 | 0.171 |
| thop1 | 0.171 |
| bsx | 0.17 |
| rgs1 | 0.17 |
| matk | 0.17 |
| zc3h15 | 0.169 |
| shc4 | 0.169 |
| cd300lb | 0.169 |
| fzd2 | 0.169 |
| eg546896 | 0.169 |
| asb15 | 0.169 |
| lifr | 0.169 |
| prkd2 | 0.169 |
| hsh2d | 0.168 |
| nmur2 | 0.168 |
| vmn2r26 | 0.168 |
| tg(msx2)1rem | 0.168 |
| sostdc1 | 0.168 |
| caprin2 | 0.168 |
| sash3 | 0.168 |
| sh3rf1 | 0.168 |
| olfr3 | 0.168 |
| gpr4 | 0.168 |
| clcf1 | 0.168 |
| ror2 | 0.168 |
| gpr3 | 0.168 |
| ptpn18 | 0.167 |
| kremen2 | 0.167 |
| zfyve9 | 0.167 |
| egr1 | 0.167 |
| gsx1 | 0.167 |
| dapp1 | 0.167 |
| cxxc4 | 0.167 |
| kremen1 | 0.167 |
| olfr985 | 0.167 |
| gpr139 | 0.166 |
| mapk8ip2 | 0.166 |
| prop1 | 0.166 |
| spred2 | 0.166 |
| iba1 | 0.166 |
| mrgpre | 0.165 |
| ppil5 | 0.165 |
| dkk4 | 0.165 |
| sla2 | 0.165 |
| olfr14 | 0.165 |
| olfr13 | 0.165 |
| olfr144 | 0.165 |
| olfr143 | 0.165 |
| olfr19 | 0.165 |
| akirin2 | 0.165 |
| hist2h2be | 0.165 |
| tshb | 0.165 |
| insl5 | 0.165 |
| dullard | 0.165 |
| lrp4 | 0.164 |
| stap2 | 0.164 |
| appl2 | 0.164 |
| mapk15 | 0.164 |
| dlx6as | 0.164 |
| rgs17 | 0.163 |
| rtp1 | 0.163 |
| hisppd1 | 0.163 |
| nol3 | 0.163 |
| dlx1as | 0.163 |
| olfr2 | 0.163 |
| d4mit46 | 0.163 |
| acvr1c | 0.163 |
| rspo3 | 0.163 |
| axin2 | 0.163 |
| crhbp | 0.162 |
| gna14 | 0.162 |
| stk38l | 0.162 |
| nab2 | 0.162 |
| egr3 | 0.162 |
| fstl1 | 0.162 |
| csnk1g2 | 0.162 |
| dok4 | 0.162 |
| paqr3 | 0.162 |
| dusp4 | 0.161 |
| mobkl1b | 0.161 |
| e030049g20rik | 0.161 |
| a530064d06rik | 0.161 |
| 2610018g03rik | 0.161 |
| ierepo2 | 0.161 |
| nmur1 | 0.161 |
| olfr705 | 0.161 |
| gpr120 | 0.161 |
| loc641201 | 0.161 |
| acvr1 | 0.161 |
| rxfp3 | 0.161 |
| frs3 | 0.161 |
| fgfr1 | 0.161 |
| rasa3 | 0.161 |
| sh2d1b2 | 0.161 |
| d4mit219 | 0.161 |
| bbaa23 | 0.161 |
| bbaa18 | 0.161 |
| camk1g | 0.161 |
| spen | 0.16 |
| gpr156 | 0.16 |
| toe1 | 0.16 |
| 9830130m13rik | 0.16 |
| stk24 | 0.16 |
| tshz1 | 0.16 |
| csnk1e | 0.16 |
| zfp423 | 0.16 |
| gigyf1 | 0.159 |
| pitpnm3 | 0.159 |
| asb3 | 0.159 |
| dact1 | 0.159 |
| pou3f3 | 0.159 |
| zfp335 | 0.159 |
| foxh1 | 0.159 |
| snx26 | 0.159 |
| zfp383 | 0.159 |
| appl1 | 0.159 |
| rasd2 | 0.159 |
| fgf8 | 0.159 |
| irg1 | 0.159 |
| tacr3 | 0.159 |
| sh3bp5 | 0.159 |
| ccdc100 | 0.159 |
| sp8 | 0.158 |
| rit2 | 0.158 |
| pea15b | 0.158 |
| ulk2 | 0.158 |
| per1 | 0.158 |
| olfr151 | 0.158 |
| 1110012m11rik | 0.158 |
| npcd | 0.158 |
| pcdh18 | 0.158 |
| ppyr1 | 0.158 |
| srms | 0.158 |
| spry3 | 0.158 |
| faim | 0.158 |
| pou3f1 | 0.158 |
| oxtr | 0.158 |
| rad54l2 | 0.158 |
| rps6ka4 | 0.158 |
| grin1 | 0.157 |
| gpr6 | 0.157 |
| kifap3 | 0.157 |
| edar | 0.157 |
| krt80 | 0.157 |
| 6430517e21rik | 0.157 |
| fgfbp3 | 0.157 |
| pip4k2b | 0.157 |
| mesdc1 | 0.157 |
| afap1 | 0.157 |
| fzd6 | 0.157 |
| gde1 | 0.157 |
| paqr8 | 0.157 |
| gad1 | 0.157 |
| ubash3b | 0.157 |
| olfr1359 | 0.157 |
| olfr153 | 0.156 |
| ccdc50 | 0.156 |
| dusp14 | 0.156 |
| nupr1 | 0.156 |
| dusp16 | 0.156 |
| zdhhc16 | 0.156 |
| mib2 | 0.156 |
| fzd5 | 0.156 |
| tsc22d4 | 0.156 |
| adcyap1r1 | 0.156 |
| rftn1 | 0.156 |
| map2k1ip1 | 0.156 |
| rxfp2 | 0.156 |
| cpz | 0.156 |
| rhov | 0.156 |
| ilkap | 0.156 |
| dlx4 | 0.156 |
| pth2 | 0.156 |
| dub1 | 0.156 |
| dgka | 0.156 |
| itpr1 | 0.156 |
| rhox5 | 0.155 |
| npb | 0.155 |
| inhbe | 0.155 |
| shisa2 | 0.155 |
| zdhhc7 | 0.155 |
| nlk | 0.155 |
| phb | 0.155 |
| ror1 | 0.155 |
| grin3b | 0.155 |
| ppp2r5b | 0.155 |
| cnga2 | 0.155 |
| sorbs2 | 0.155 |
| tnk1 | 0.154 |
| paqr7 | 0.154 |
| mapkbp1 | 0.154 |
| vipr2 | 0.154 |
| smarca1 | 0.154 |
| sfrs9 | 0.154 |
| dgkk | 0.154 |
| bc042720 | 0.153 |
| shc2 | 0.153 |
| cst8 | 0.153 |
| zcchc12 | 0.153 |
| lime1 | 0.153 |
| spopl | 0.153 |
| 3930401k13rik | 0.153 |
| klri1 | 0.153 |
| sgsm1 | 0.153 |
| nkd1 | 0.153 |
| nps | 0.153 |
| pomc-ps1 | 0.153 |
| gata5 | 0.153 |
| fosl2 | 0.153 |
| eg630579 | 0.152 |
| fgf17 | 0.152 |
| dub2 | 0.152 |
| dkk2 | 0.152 |
| v1rb2 | 0.152 |
| npy1r | 0.152 |
| il17re | 0.152 |
| map3k13 | 0.152 |
| star | 0.152 |
| rbm4b | 0.152 |
| plxnb1 | 0.152 |
| bmx | 0.152 |
| tshz3 | 0.152 |
| ripk5 | 0.152 |
| rgs18 | 0.152 |
| wnt10a | 0.152 |
| nab1 | 0.151 |
| plekha2 | 0.151 |
| snx6 | 0.151 |
| lhx2 | 0.151 |
| sarm1 | 0.151 |
| plcb3 | 0.151 |
| nrk | 0.151 |
| med28 | 0.151 |
| dbx1 | 0.151 |
| vldlr | 0.151 |
| mageh1 | 0.151 |
| lrrc19 | 0.151 |
| cbll1 | 0.151 |
| epb4.1l4b | 0.151 |
| dok2 | 0.151 |
| nxn | 0.151 |
| tob1 | 0.151 |
| grb14 | 0.15 |
| arhgef15 | 0.15 |
| rp23-157o10.7 | 0.15 |
| pak6 | 0.15 |
| nfam1 | 0.15 |
| shcbp1 | 0.15 |
| siglec15 | 0.15 |
| dbpht2 | 0.15 |
| sirpb1 | 0.15 |
| pi4k2b | 0.15 |
| grin3a | 0.15 |
| sh2d2a | 0.15 |
| rnf111 | 0.15 |
| otx2 | 0.15 |
| grin2b | 0.15 |
| olfr459 | 0.15 |
| zfyve16 | 0.15 |
| reln | 0.149 |
| tnip2 | 0.149 |
| clec12b | 0.149 |
| lhx4 | 0.149 |
| mpzl1 | 0.149 |
| cxxc5 | 0.149 |
| anxa5 | 0.149 |
| map3k9 | 0.149 |
| map4k5 | 0.149 |
| nle1 | 0.149 |
| ntsr2 | 0.149 |
| mirn9-1 | 0.149 |
| ly6g6f | 0.149 |
| arhgap10 | 0.149 |
| spata5 | 0.149 |
| enc1 | 0.149 |
| traf7 | 0.149 |
| nisch | 0.149 |
| rufy2 | 0.149 |
| gab3 | 0.149 |
| gna15 | 0.149 |
| rnf130 | 0.148 |
| trappc9 | 0.148 |
| pag1 | 0.148 |
| gas6 | 0.148 |
| plekhm3 | 0.148 |
| smad6 | 0.148 |
| olfr677 | 0.148 |
| olfr661 | 0.148 |
| olfr547 | 0.148 |
| tgfbr3 | 0.148 |
| rasal1 | 0.148 |
| usp6nl | 0.148 |
| olfr1168 | 0.148 |
| olfr642 | 0.148 |
| olfr164 | 0.148 |
| stc1 | 0.148 |
| ebi2 | 0.148 |
| hcls1 | 0.148 |
| styk1 | 0.148 |
| rps6ka5 | 0.148 |
| lrrfip1 | 0.148 |
| syngap1 | 0.148 |
| spred3 | 0.148 |
| dear1 | 0.148 |
| fanca | 0.148 |
| olfr1507 | 0.148 |
| centg3 | 0.148 |
| zmynd19 | 0.148 |
| mobkl1a | 0.148 |
| pik3r3 | 0.148 |
| mrgprb2 | 0.147 |
| lhx8 | 0.147 |
| stam | 0.147 |
| esr2 | 0.147 |
| pdyn | 0.147 |
| b830045n13rik | 0.147 |
| batf3 | 0.147 |
| npff | 0.147 |
| map3k12 | 0.147 |
| spred1 | 0.147 |
| trafd1 | 0.147 |
| plcl2 | 0.147 |
| zfp503 | 0.147 |
| epha3 | 0.147 |
| nrarp | 0.147 |
| wnt5b | 0.147 |
| xmv24 | 0.146 |
| akr1b7 | 0.146 |
| foxg1 | 0.146 |
| nr4a3 | 0.146 |
| ducm1 | 0.146 |
| mapk4 | 0.146 |
| cmtm3 | 0.146 |
| abi2 | 0.146 |
| lrrn3 | 0.146 |
| ptpn14 | 0.146 |
| olfr1508 | 0.146 |
| olfr1509 | 0.146 |
| kank2 | 0.146 |
| wnt2b | 0.146 |
| tbc1d10c | 0.146 |
| gpr37l1 | 0.146 |
| rorb | 0.146 |
| olfr73 | 0.146 |
| aa407270 | 0.146 |
| disp2 | 0.146 |
| itpkb | 0.146 |
| rgs2 | 0.146 |
| necab2 | 0.145 |
| tbr1 | 0.145 |
| skap2 | 0.145 |
| grin2c | 0.145 |
| wnt7b | 0.145 |
| gpr142 | 0.145 |
| npbwr1 | 0.145 |
| pde4d | 0.145 |
| ksr1 | 0.145 |
| med23 | 0.145 |
| nr1d2 | 0.145 |
| rin1 | 0.145 |
| rhbdl3 | 0.145 |
| ptk2b | 0.145 |
| olfr544 | 0.145 |
| stambpl1 | 0.145 |
| pja2 | 0.145 |
| git1 | 0.145 |
| sema6d | 0.145 |
| cdkl2 | 0.145 |
| vasn | 0.145 |
| tg(wnt3)7gsb | 0.145 |
| 6330417g02rik | 0.145 |
| nr2c2 | 0.144 |
| nrbp1 | 0.144 |
| ric8 | 0.144 |
| centa1 | 0.144 |
| bex6 | 0.144 |
| socs5 | 0.144 |
| gprin1 | 0.144 |
| arhgef6 | 0.144 |
| edk | 0.144 |
| prlhr | 0.144 |
| spry2 | 0.144 |
| ankrd28 | 0.144 |
| asb4 | 0.144 |
| 1500001a10rik | 0.144 |
| cd320 | 0.144 |
| nfya | 0.144 |
| gpr50 | 0.144 |
| kcnip3 | 0.144 |
| scamp4 | 0.144 |
| act1 | 0.144 |
| dgkb | 0.143 |
| ksr2 | 0.143 |
| mchr1 | 0.143 |
| bmper | 0.143 |
| ak3l1 | 0.143 |
| reps2 | 0.143 |
| npffr2 | 0.143 |
| olfr410 | 0.143 |
| stk3 | 0.143 |
| snx18 | 0.143 |
| map4k3 | 0.143 |
| dusp5 | 0.143 |
| relt | 0.143 |
| ppp2r2d | 0.143 |
| ipmk | 0.143 |
| pak7 | 0.143 |
| klri2 | 0.142 |
| arhgap26 | 0.142 |
| ecsit | 0.142 |
| itpk1 | 0.142 |
| sema4d | 0.142 |
| gpr111 | 0.142 |
| gpr128 | 0.142 |
| bc023829 | 0.142 |
| jakmip1 | 0.142 |
| fgf12 | 0.142 |
| clec4b1 | 0.142 |
| camk2n2 | 0.142 |
| stk40 | 0.142 |
| ysk4 | 0.142 |
| rusc1 | 0.142 |
| glrx3 | 0.142 |
| cdc2b | 0.142 |
| med6 | 0.142 |
| vgot2 | 0.141 |
| ppp2r5a | 0.141 |
| spry1 | 0.141 |
| ptk7 | 0.141 |
| hesx1 | 0.141 |
| spop | 0.141 |
| fzd8 | 0.141 |
| il31ra | 0.141 |
| tnfrsf19 | 0.141 |
| bc063749 | 0.141 |
| rax | 0.141 |
| cuedc2 | 0.141 |
| dixdc1 | 0.141 |
| olfr140 | 0.141 |
| olfr71 | 0.141 |
| prrx1 | 0.141 |
| sav1 | 0.141 |
| 1110006o17rik | 0.141 |
| erbb2ip | 0.141 |
| fosl1 | 0.141 |
| lrp6 | 0.14 |
| grin2a | 0.14 |
| abi3 | 0.14 |
| taok3 | 0.14 |
| fzd7 | 0.14 |
| ythdc1 | 0.14 |
| flrt1 | 0.14 |
| olfr16 | 0.14 |
| pth2r | 0.14 |
| plcb1 | 0.14 |
| npffr1 | 0.14 |
| gpr68 | 0.14 |
| tnfrsf12a | 0.14 |
| fzd1 | 0.14 |
| jdp2 | 0.14 |
| phlppl | 0.14 |
| gps1 | 0.14 |
| mapkap1 | 0.14 |
| emx2 | 0.14 |
| shb | 0.139 |
| map3k10 | 0.139 |
| zranb1 | 0.139 |
| strn | 0.139 |
| hcrtr1 | 0.139 |
| wdr34 | 0.139 |
| tshz2 | 0.139 |
| rgs5 | 0.139 |
| mrvi1 | 0.139 |
| stmn2 | 0.139 |
| pde8a | 0.139 |
| irf2bp1 | 0.139 |
| rspo1 | 0.139 |
| fgf9 | 0.139 |
| pstpip1 | 0.139 |
| pitpnm2 | 0.139 |
| ptprr | 0.139 |
| ergic2 | 0.139 |
| gulp1 | 0.139 |
| gbx2 | 0.139 |
| frat2 | 0.139 |
| hisppd2a | 0.139 |
| tgfb1i1 | 0.139 |
| pitx2 | 0.139 |
| traip | 0.139 |
| rasgrf2 | 0.139 |
| mirn7b | 0.139 |
| plcl1 | 0.139 |
| dusp6 | 0.139 |
| spsb2 | 0.139 |
| d5mit148 | 0.138 |
| chst8 | 0.138 |
| cnksr2 | 0.138 |
| npy2r | 0.138 |
| ihpk3 | 0.138 |
| rnd1 | 0.138 |
| ghrh | 0.138 |
| per2 | 0.138 |
| pip4k2c | 0.138 |
| b3gnt1 | 0.138 |
| cartpt | 0.138 |
| rgs8 | 0.138 |
| rtp4 | 0.138 |
| akap6 | 0.138 |
| ripk4 | 0.138 |
| bc018242 | 0.138 |
| vmn2r65 | 0.138 |
| vmn2r111 | 0.138 |
| eg619517 | 0.138 |
| vmn2r112 | 0.138 |
| rgs12 | 0.138 |
| plekho1 | 0.138 |
| ubash3a | 0.138 |
| eps8 | 0.138 |
| clnk | 0.138 |
| gpr83 | 0.138 |
| mirn147 | 0.138 |
| kap | 0.138 |
| pear1 | 0.138 |
| lmtk3 | 0.138 |
| olfr37 | 0.138 |
| nhlh2 | 0.138 |
| csk | 0.137 |
| nuak1 | 0.137 |
| tnik | 0.137 |
| fgf18 | 0.137 |
| flrt2 | 0.137 |
| prpf19 | 0.137 |
| nrbp2 | 0.137 |
| btg3 | 0.137 |
| fem1a | 0.137 |
| tesk1 | 0.137 |
| pdlim2 | 0.137 |
| sit1 | 0.137 |
| nck2 | 0.137 |
| svet1 | 0.137 |
| camk2n1 | 0.137 |
| rgs20 | 0.137 |
| tcf7 | 0.137 |
| ripk3 | 0.137 |
| tmem9 | 0.137 |
| grk5 | 0.137 |
| cntfr | 0.137 |
| plxnc1 | 0.137 |
| jund | 0.137 |
| otud5 | 0.137 |
| dbnl | 0.137 |
| cxcl3 | 0.137 |
| ihpk2 | 0.137 |
| pcdhgb1 | 0.136 |
| gucy2d | 0.136 |
| tnk2 | 0.136 |
| sla | 0.136 |
| tbx3 | 0.136 |
| trip6 | 0.136 |
| fezf2 | 0.136 |
| khdrbs2 | 0.136 |
| otp | 0.136 |
| bcar3 | 0.136 |
| trh | 0.136 |
| ric8b | 0.136 |
| rgnef | 0.136 |
| socs7 | 0.136 |
| cblc | 0.136 |
| tmem173 | 0.136 |
| itgb3bp | 0.136 |
| aatk | 0.136 |
| olfr256 | 0.136 |
| map3k11 | 0.136 |
| emo1 | 0.136 |
| rxfp4 | 0.136 |
| smpd3 | 0.136 |
| l1md-a5 | 0.136 |
| clec2g | 0.136 |
| atf1 | 0.136 |
| ptprz1 | 0.136 |
| brms1 | 0.136 |
| sgsm2 | 0.135 |
| dusp7 | 0.135 |
| nsmaf | 0.135 |
| ebf4 | 0.135 |
| npy | 0.135 |
| cby | 0.135 |
| rsu1 | 0.135 |
| mtus1 | 0.135 |
| gadd45g | 0.135 |
| 0610011l14rik | 0.135 |
| plce1 | 0.135 |
| card14 | 0.135 |
| igfbp5-ip | 0.135 |
| fzd10 | 0.135 |
| tank | 0.135 |
| vgot1 | 0.135 |
| mprip | 0.135 |
| sbk1 | 0.135 |
| mirn9-2 | 0.135 |
| mirn9-3 | 0.135 |
| strn3 | 0.135 |
| smurf1 | 0.135 |
| bmp10 | 0.135 |
| gna13 | 0.135 |
| rell1 | 0.135 |
| rell2 | 0.135 |
| tnfaip8l2 | 0.135 |
| map3k2 | 0.135 |
| fiz1 | 0.135 |
| dok1 | 0.135 |
| ngef | 0.135 |
| sh3bp2 | 0.135 |
| d17mit181 | 0.135 |
| nr2c2ap | 0.134 |
| pygo1 | 0.134 |
| mdga1 | 0.134 |
| gripap1 | 0.134 |
| cdgap | 0.134 |
| card6 | 0.134 |
| centg1 | 0.134 |
| asb6 | 0.134 |
| tro | 0.134 |
| hand2 | 0.134 |
| irs4 | 0.134 |
| arntl | 0.134 |
| crebzf | 0.134 |
| rasgrp3 | 0.134 |
| stk25 | 0.134 |
| lrig1 | 0.134 |
| slfn2 | 0.134 |
| ascc1 | 0.134 |
| sfrs7 | 0.134 |
| gpr39 | 0.134 |
| dedd | 0.134 |
| notum | 0.133 |
| spsb1 | 0.133 |
| cenpi | 0.133 |
| gas1 | 0.133 |
| chrdl1 | 0.133 |
| nrp | 0.133 |
| chac1 | 0.133 |
| gpbar1 | 0.133 |
| wdfy2 | 0.133 |
| stk4 | 0.133 |
| kank1 | 0.133 |
| sgpp1 | 0.133 |
| blk | 0.133 |
| pip4k2a | 0.133 |
| 2900073g15rik | 0.133 |
| daam2 | 0.133 |
| khdc1b | 0.133 |
| stk16 | 0.133 |
| prickle1 | 0.133 |
| anx | 0.133 |
| skap1 | 0.133 |
| vwce | 0.133 |
| cdh22 | 0.133 |
| lif | 0.133 |
| akt1s1 | 0.133 |
| nfil3 | 0.133 |
| sucnr1 | 0.132 |
| s1pr4 | 0.132 |
| bag4 | 0.132 |
| clk4 | 0.132 |
| stam2 | 0.132 |
| 6330500d04rik | 0.132 |
| hey1 | 0.132 |
| avil | 0.132 |
| rgs7bp | 0.132 |
| pik3c2g | 0.132 |
| dyrk1c | 0.132 |
| itgb1bp1 | 0.132 |
| ptpn5 | 0.132 |
| sh2b2 | 0.132 |
| dusp2 | 0.132 |
| fzd9 | 0.132 |
| them4 | 0.132 |
| tatdn2 | 0.132 |
| chn2 | 0.132 |
| arhgap21 | 0.132 |
| taar9 | 0.132 |
| ppp2r3c | 0.132 |
| klrb1f | 0.132 |
| dvl3 | 0.132 |
| cd300lf | 0.132 |
| nudt3 | 0.132 |
| dgkq | 0.132 |
| ottmusg00000008540 | 0.132 |
| trim33 | 0.132 |
| taok2 | 0.132 |
| arhgef7 | 0.132 |
| 4933407c03rik | 0.132 |
| git2 | 0.132 |
| gprc6a | 0.132 |
| errfi1 | 0.132 |
| tg(alb1-cre)1rck | 0.132 |
| klf9 | 0.132 |
| agrp | 0.132 |
| ranbp10 | 0.132 |
| pak1 | 0.132 |
| clec4b2 | 0.132 |
| tnfrsf25 | 0.131 |
| olfr160 | 0.131 |
| plch2 | 0.131 |
| six3 | 0.131 |
| ncdn | 0.131 |
| axin1 | 0.131 |
| cc2d1a | 0.131 |
| centd3 | 0.131 |
| il20ra | 0.131 |
| dub4 | 0.131 |
| ecel1 | 0.131 |
| camk2g | 0.131 |
| 1110007c09rik | 0.131 |
| adcy1 | 0.131 |
| otud7b | 0.131 |
| ier5 | 0.131 |
| tiprl | 0.131 |
| gpr65 | 0.131 |
| dph5 | 0.131 |
| gpr20 | 0.131 |
| prkg2 | 0.131 |
| camk1d | 0.131 |
| nlrp6 | 0.131 |
| dusp26 | 0.13 |
| lats1 | 0.13 |
| sh2b3 | 0.13 |
| numbl | 0.13 |
| map4k4 | 0.13 |
| rhou | 0.13 |
| mobkl3 | 0.13 |
| grin2d | 0.13 |
| fpr-rs3 | 0.13 |
| sh2d1b1 | 0.13 |
| ptchd3 | 0.13 |
| ulk1 | 0.13 |
| 9430023l20rik | 0.13 |
| snf1lk | 0.13 |
| hcst | 0.13 |
| rgmb | 0.13 |
| leprot | 0.13 |
| cd200r4 | 0.13 |
| ghsr | 0.13 |
| pik3c2b | 0.13 |
| frs2 | 0.13 |
| stambp | 0.13 |
| pak3 | 0.13 |
| zfp322a | 0.13 |
| e130309f12rik | 0.13 |
| trim11 | 0.13 |
| tnip3 | 0.13 |
| stk10 | 0.13 |
| cnot8 | 0.13 |
| mark4 | 0.13 |
| osmr | 0.13 |
| zmiz1 | 0.13 |
| olfr43 | 0.129 |
| clk1 | 0.129 |
| plcd1 | 0.129 |
| abl2 | 0.129 |
| rgs6 | 0.129 |
| tcf3 | 0.129 |
| rqcd1 | 0.129 |
| thoc5 | 0.129 |
