# Supplementary material for: GIT2 Acts as a Potential Keystone Protein in Functional Hypothalamic Networks Associated with Age-Related Phenotypic Changes in Rats
Source: PLoS One. 2012 May 14;7(5):e36975. doi: 10.1371/journal.pone.0036975 (PMC3351446; doi:10.1371/journal.pone.0036975)
Supplement: Table S15 — GeneIndexer latent semantic indexing (LSI) of significantly-regulated ‘Long term potentiation’ KEGG pathway. Using the KEGG signaling pathway ‘Long term potentiation’ as an input term, a list of the top 1000 implicitly-correlated (LSI correlation score >0.1) was generated using a full genome background list. (DOC) [file pone.0036975.s019.doc]

**Table S15. GeneIndexer latent semantic indexing (LSI) of significantly-regulated ‘Long term potentiation’ KEGG pathway.** Using the KEGG signaling pathway ‘Long term potentiation’ as an input term, a list of the top 1000 implicitly-correlated (LSI correlation score >0.1) was generated using a full genome background list.

| ***Long term potentiation*** |  |
| --- | --- |
|  |  |
| **Protein Symbol** | **LSI correlation score** |
| ottmusg00000014994 | 0.411 |
| adcy8 | 0.351 |
| gria1 | 0.34 |
| gria2 | 0.332 |
| arc | 0.327 |
| adcy1 | 0.323 |
| grik1 | 0.32 |
| grik4 | 0.311 |
| lrrn4 | 0.308 |
| gria3 | 0.304 |
| d12mit171 | 0.303 |
| grik2 | 0.301 |
| akap5 | 0.296 |
| syngap1 | 0.292 |
| rims1 | 0.291 |
| gria4 | 0.285 |
| syt4 | 0.282 |
| nrgn | 0.277 |
| grik5 | 0.276 |
| camk2a | 0.275 |
| camk2b | 0.275 |
| grik3 | 0.273 |
| grm1 | 0.268 |
| grin1 | 0.267 |
| grm5 | 0.263 |
| homer1 | 0.261 |
| grin2b | 0.258 |
| grm2 | 0.258 |
| cacng8 | 0.257 |
| loc667655 | 0.25 |
| grin2a | 0.25 |
| cpne6 | 0.248 |
| eg622509 | 0.247 |
| grm7 | 0.245 |
| pick1 | 0.244 |
| camkk2 | 0.243 |
| grin2c | 0.238 |
| dagla | 0.236 |
| d8mit77 | 0.234 |
| git2 | 0.234 |
| syn2 | 0.232 |
| camk4 | 0.23 |
| trim2 | 0.23 |
| cnih3 | 0.229 |
| cacng2 | 0.229 |
| syn1 | 0.229 |
| plcb4 | 0.228 |
| kcnn2 | 0.226 |
| rims2 | 0.224 |
| unc13c | 0.224 |
| grin3a | 0.224 |
| skts5 | 0.223 |
| grip2 | 0.222 |
| prkcc | 0.222 |
| rab3a | 0.22 |
| vsnl1 | 0.22 |
| chrm1 | 0.22 |
| loc677282 | 0.218 |
| lrncs1 | 0.218 |
| lrnx8 | 0.218 |
| grin2d | 0.218 |
| zswim6 | 0.217 |
| plcb1 | 0.217 |
| ryr3 | 0.216 |
| camk2g | 0.215 |
| unc13a | 0.214 |
| bc018242 | 0.214 |
| daglb | 0.214 |
| accn2 | 0.214 |
| srr | 0.213 |
| klk8 | 0.213 |
| grm3 | 0.212 |
| slc30a3 | 0.211 |
| adcy5 | 0.211 |
| prl7d1 | 0.21 |
| t(12;16)1cje | 0.21 |
| homer2 | 0.21 |
| egr3 | 0.21 |
| chrm4 | 0.209 |
| rasgrf1 | 0.208 |
| gucy1a2 | 0.208 |
| d13mit94 | 0.207 |
| prkar1b | 0.206 |
| cnga2 | 0.206 |
| t(12;16)1cje | 0.205 |
| nsg1 | 0.205 |
| jph4 | 0.205 |
| camk1 | 0.204 |
| slc6a9 | 0.204 |
| unc13b | 0.204 |
| itpr1 | 0.204 |
| grid2ip | 0.204 |
| grm4 | 0.203 |
| l9rl1 | 0.203 |
| pde10a | 0.203 |
| cacna1d | 0.202 |
| ppp1r1b | 0.202 |
| egr1 | 0.201 |
| nptx2 | 0.201 |
| camkk1 | 0.201 |
| prkacb | 0.2 |
| cacna1c | 0.199 |
| itpka | 0.199 |
| ppp1r9a | 0.199 |
| pde4b | 0.198 |
| cplx2 | 0.198 |
| ppp1r1a | 0.198 |
| fosb | 0.198 |
| cabp1 | 0.197 |
| chrm2 | 0.197 |
| adcy2 | 0.195 |
| mtap6 | 0.195 |
| nptx1 | 0.195 |
| dlg4 | 0.194 |
| crhbp | 0.194 |
| pde4a | 0.194 |
| accn1 | 0.194 |
| gnaq | 0.193 |
| cnga4 | 0.193 |
| gabbr1 | 0.193 |
| neto1 | 0.192 |
| prl2c2 | 0.192 |
| grin3b | 0.192 |
| adcy4 | 0.191 |
| grm8 | 0.191 |
| grip1 | 0.191 |
| gcm1 | 0.19 |
| au041133 | 0.189 |
| glud2 | 0.189 |
| hrh1 | 0.189 |
| fgf14 | 0.189 |
| rgs2 | 0.189 |
| 5930434b04rik | 0.188 |
| prl3d1 | 0.188 |
| rasgrf2 | 0.188 |
| epb4.1l1 | 0.187 |
| prl3b1 | 0.187 |
| grina | 0.187 |
| pde1b | 0.187 |
| chrm3 | 0.186 |
| htr4 | 0.186 |
| chrm5 | 0.186 |
| cpeb1 | 0.186 |
| igsf9b | 0.184 |
| prkg1 | 0.184 |
| gna11 | 0.183 |
| pclo | 0.183 |
| cacna1e | 0.183 |
| adcy6 | 0.183 |
| trpc5 | 0.183 |
| crhr1 | 0.183 |
| caps2 | 0.182 |
| doc2a | 0.182 |
| klhl24 | 0.182 |
| splrn2 | 0.181 |
| ofca3 | 0.181 |
| ofca2 | 0.181 |
| ofca4 | 0.181 |
| ofca1 | 0.181 |
| splrn1 | 0.181 |
| homer3 | 0.18 |
| tex261 | 0.18 |
| ddo | 0.18 |
| d2mit266 | 0.18 |
| prkar2b | 0.18 |
| prl7a2 | 0.179 |
| lrnx7 | 0.179 |
| lrnx9 | 0.179 |
| lrnx10 | 0.179 |
| hsd11b2 | 0.178 |
| syn3 | 0.178 |
| galr1 | 0.178 |
| itpr3 | 0.178 |
| prr7 | 0.177 |
| rapgef3 | 0.177 |
| rph3a | 0.175 |
| camk2d | 0.175 |
| stox1 | 0.175 |
| oxtr | 0.175 |
| shank1 | 0.174 |
| s100b | 0.174 |
| cacna2d1 | 0.174 |
| hpca | 0.174 |
| hsd11b1 | 0.173 |
| pde2a | 0.173 |
| adcy7 | 0.173 |
| aa407270 | 0.173 |
| bsn | 0.173 |
| syt1 | 0.172 |
| gabrd | 0.172 |
| ntrk2 | 0.172 |
| crh | 0.171 |
| ntan1 | 0.171 |
| itpr2 | 0.171 |
| grid2 | 0.171 |
| erc2 | 0.171 |
| hrh3 | 0.17 |
| psg29 | 0.17 |
| psg22 | 0.17 |
| nlgn1 | 0.17 |
| neurod6 | 0.169 |
| cacna1b | 0.169 |
| vgf | 0.169 |
| rab3c | 0.169 |
| htr7 | 0.168 |
| bc1 | 0.168 |
| gap43 | 0.168 |
| pde5a | 0.168 |
| tpbpa | 0.168 |
| pde3a | 0.168 |
| grid1 | 0.168 |
| freq | 0.168 |
| gabrb1 | 0.168 |
| plac1 | 0.167 |
| prkg2 | 0.167 |
| neurod2 | 0.167 |
| pnoc | 0.167 |
| adcy3 | 0.166 |
| pappa2 | 0.166 |
| gucy1a3 | 0.166 |
| prss12 | 0.166 |
| oprl1 | 0.165 |
| slc8a2 | 0.165 |
| creb1 | 0.165 |
| grinl1a | 0.165 |
| trpc6 | 0.165 |
| fmr1 | 0.165 |
| ppp3ca | 0.164 |
| marcks | 0.164 |
| p2rx4 | 0.164 |
| p2rx3 | 0.164 |
| caln1 | 0.163 |
| cacng5 | 0.163 |
| prl4a1 | 0.163 |
| aal | 0.163 |
| syt12 | 0.163 |
| pibf1 | 0.162 |
| camk1g | 0.162 |
| ncam1 | 0.162 |
| kcnip3 | 0.162 |
| drd1a | 0.161 |
| crhr2 | 0.161 |
| ppp1r9b | 0.161 |
| prl6a1 | 0.161 |
| nsf | 0.161 |
| aap | 0.161 |
| drd5 | 0.161 |
| icam5 | 0.161 |
| ppfia4 | 0.161 |
| gal | 0.161 |
| st8sia4 | 0.161 |
| cplx4 | 0.16 |
| akap7 | 0.16 |
| el1 | 0.16 |
| lnpep | 0.16 |
| syngr1 | 0.16 |
| gabra5 | 0.16 |
| hpcal4 | 0.16 |
| adcy9 | 0.16 |
| cadps2 | 0.16 |
| nell2 | 0.159 |
| cfcd | 0.159 |
| pde1c | 0.159 |
| chl1 | 0.159 |
| fyn | 0.159 |
| cbln1 | 0.159 |
| efhb | 0.158 |
| cacng7 | 0.158 |
| doc2g | 0.158 |
| lrp8 | 0.157 |
| rims4 | 0.157 |
| nrn1 | 0.157 |
| oxt | 0.157 |
| chrna7 | 0.157 |
| hmox2 | 0.157 |
| d3bwg0562e | 0.156 |
| psg19 | 0.156 |
| gabrb3 | 0.156 |
| 5730528l13rik | 0.156 |
| p2rx2 | 0.156 |
| nps | 0.156 |
| rapgef4 | 0.156 |
| pappa | 0.156 |
| ppp3r1 | 0.155 |
| hpgd | 0.155 |
| crem | 0.155 |
| kcnma1 | 0.155 |
| pde4d | 0.155 |
| prl2a1 | 0.154 |
| ptgs1 | 0.154 |
| camk2n1 | 0.154 |
| s100g | 0.154 |
| cbln3 | 0.154 |
| ucn | 0.154 |
| gabra3 | 0.153 |
| trpc4 | 0.153 |
| ryr1 | 0.153 |
| ppp3r2 | 0.153 |
| pde4c | 0.152 |
| cacnb3 | 0.152 |
| prl8a2 | 0.152 |
| gabra1 | 0.152 |
| slc5a7 | 0.152 |
| caly | 0.152 |
| pgr | 0.152 |
| gabbr2 | 0.152 |
| prl7b1 | 0.151 |
| ephb1 | 0.151 |
| nlgn2 | 0.151 |
| ophn1 | 0.151 |
| ceacam9 | 0.151 |
| pde3b | 0.151 |
| dmxl2 | 0.151 |
| raver2 | 0.15 |
| adnp | 0.15 |
| sbp | 0.15 |
| prkcb1 | 0.15 |
| shank3 | 0.15 |
| cplx1 | 0.149 |
| ntf5 | 0.149 |
| trpv6 | 0.149 |
| pde9a | 0.149 |
| rims3 | 0.149 |
| trpv4 | 0.149 |
| d19ertd386e | 0.149 |
| trpm4 | 0.149 |
| rab3b | 0.148 |
| prkcz | 0.148 |
| nrxn3 | 0.148 |
| adarb1 | 0.147 |
| sipa1l1 | 0.147 |
| dlgap4 | 0.147 |
| slc8a3 | 0.147 |
| nr3c2 | 0.147 |
| cnn3 | 0.147 |
| trpc3 | 0.146 |
| cript | 0.146 |
| glp1r | 0.146 |
| cacng3 | 0.146 |
| klhl17 | 0.146 |
| ntf3 | 0.146 |
| pde1a | 0.146 |
| nd | 0.146 |
| tnr | 0.146 |
| efnb3 | 0.146 |
| trpv1 | 0.146 |
| snapin | 0.145 |
| kiss1 | 0.145 |
| cacna1g | 0.145 |
| frct1 | 0.145 |
| frct3 | 0.145 |
| frct4 | 0.145 |
| frct2 | 0.145 |
| frct5 | 0.145 |
| mylk2 | 0.145 |
| oprs1 | 0.145 |
| ppp3cc | 0.145 |
| prl5a1 | 0.145 |
| kcnn3 | 0.145 |
| prl3c1 | 0.145 |
| rab3gap1 | 0.145 |
| cm | 0.144 |
| sytl3 | 0.144 |
| kcnk2 | 0.144 |
| avpr1b | 0.144 |
| sbnt1 | 0.144 |
| elnt | 0.144 |
| sbnt2 | 0.144 |
| trpc7 | 0.144 |
| rimbp2 | 0.144 |
| pde8b | 0.144 |
| cacng4 | 0.143 |
| dlgap2 | 0.143 |
| rgs9 | 0.143 |
| adcyap1 | 0.143 |
| ryr2 | 0.143 |
| nova2 | 0.143 |
| hrh2 | 0.143 |
| cabp5 | 0.143 |
| dao1 | 0.143 |
| adrb1 | 0.143 |
| tmem38b | 0.142 |
| jph3 | 0.142 |
| crbn | 0.142 |
| plat | 0.142 |
| reln | 0.142 |
| l1cam | 0.142 |
| hdc | 0.141 |
| adora2a | 0.141 |
| mecp2 | 0.141 |
| bdnf | 0.141 |
| p2ry1 | 0.141 |
| pnck | 0.141 |
| gjd2 | 0.14 |
| emx1 | 0.14 |
| cabp7 | 0.14 |
| gabra4 | 0.14 |
| srd5a1 | 0.139 |
| pde7a | 0.139 |
| rgs4 | 0.139 |
| nr3c1 | 0.139 |
| dautb1 | 0.139 |
| app | 0.139 |
| adora1 | 0.139 |
| lrrc4b | 0.139 |
| psg21 | 0.139 |
| klk1b8 | 0.139 |
| p2rx1 | 0.139 |
| syt9 | 0.139 |
| gabra6 | 0.139 |
| ppp1r14a | 0.139 |
| lrnx11 | 0.138 |
| lrnx12 | 0.138 |
| gnal | 0.138 |
| ier5 | 0.138 |
| gabrg2 | 0.138 |
| crebbp | 0.138 |
| 2900073g15rik | 0.138 |
| axtrb1 | 0.138 |
| axtrb5 | 0.138 |
| axtrb3 | 0.138 |
| axtlm | 0.138 |
| axtrb2 | 0.138 |
| axtrb4 | 0.138 |
| plk2 | 0.138 |
| aadat | 0.138 |
| adcyap1r1 | 0.138 |
| slc8a1 | 0.138 |
| trappc4 | 0.137 |
| bc046331 | 0.137 |
| lrrc7 | 0.136 |
| hld | 0.136 |
| slc38a4 | 0.136 |
| bzrap1 | 0.136 |
| igfbp1 | 0.136 |
| syt10 | 0.136 |
| star | 0.135 |
| gng7 | 0.135 |
| gip | 0.135 |
| galr2 | 0.135 |
| dclk3 | 0.135 |
| htr3a | 0.135 |
| kcnk9 | 0.135 |
| gcg | 0.135 |
| kcnn1 | 0.135 |
| gabrr1 | 0.135 |
| pla2g4a | 0.135 |
| cplx3 | 0.135 |
| psen1 | 0.134 |
| prkar2a | 0.134 |
| wdr7 | 0.134 |
| cacna1h | 0.134 |
| slc12a5 | 0.134 |
| map6d1 | 0.134 |
| synpo | 0.134 |
| etn1 | 0.134 |
| cyp2e1 | 0.133 |
| mctp1 | 0.133 |
| htt | 0.133 |
| pcdhga3 | 0.133 |
| fkbp1a | 0.133 |
| junb | 0.133 |
| gabrb2 | 0.133 |
| trpa1 | 0.132 |
| smtnl1 | 0.132 |
| kif17 | 0.132 |
| slc18a3 | 0.132 |
| camk2n2 | 0.132 |
| cacna1i | 0.132 |
| igfbp4 | 0.132 |
| slc38a2 | 0.132 |
| cpeb4 | 0.131 |
| arhgef9 | 0.131 |
| nab1 | 0.131 |
| dclk2 | 0.131 |
| kcnk3 | 0.131 |
| rab3d | 0.131 |
| atf1 | 0.131 |
| pvalb | 0.131 |
| ptafr | 0.131 |
| d5mit274 | 0.131 |
| gucy1b3 | 0.13 |
| ppfia3 | 0.13 |
| ptpn5 | 0.13 |
| rxra | 0.13 |
| h2-m3 | 0.13 |
| efnb2 | 0.13 |
| gnb2l1 | 0.13 |
| dgkb | 0.13 |
| cd38 | 0.13 |
| plcb3 | 0.13 |
| chrnb2 | 0.13 |
| cabp4 | 0.13 |
| prkar1a | 0.13 |
| rcan1 | 0.13 |
| kiss1r | 0.13 |
| pde11a | 0.13 |
| kcnd2 | 0.13 |
| prl7a1 | 0.129 |
| accn3 | 0.129 |
| hcn1 | 0.129 |
| plcz1 | 0.129 |
| cadps | 0.129 |
| ephb2 | 0.129 |
| trpc2 | 0.129 |
| atp2b3 | 0.128 |
| slc17a7 | 0.128 |
| sri | 0.128 |
| paep | 0.128 |
| d1mit181 | 0.128 |
| trpm8 | 0.128 |
| dbn1 | 0.128 |
| nrxn1 | 0.128 |
| trpc1 | 0.128 |
| cnih2 | 0.128 |
| kalrn | 0.128 |
| dclk1 | 0.128 |
| rasd2 | 0.128 |
| trpm5 | 0.127 |
| abcg2 | 0.127 |
| cacnb1 | 0.127 |
| jph1 | 0.127 |
| c330019g07rik | 0.127 |
| tmub1 | 0.127 |
| st8sia2 | 0.127 |
| kcnmb4 | 0.127 |
| nab2 | 0.126 |
| adm | 0.126 |
| opfa | 0.126 |
| p2rx7 | 0.126 |
| mtap2 | 0.126 |
| lsg1 | 0.126 |
| cacng1 | 0.126 |
| phmr | 0.126 |
| slc30a1 | 0.126 |
| slc1a2 | 0.126 |
| nr4a1 | 0.126 |
| klra15 | 0.126 |
| dlg2 | 0.126 |
| gprin1 | 0.126 |
| indo | 0.126 |
| clic3 | 0.126 |
| kcnt2 | 0.125 |
| tmod2 | 0.125 |
| prkaca | 0.125 |
| faah | 0.125 |
| adora2b | 0.125 |
| trpm2 | 0.125 |
| pgf | 0.125 |
| slc17a6 | 0.125 |
| ptgfr | 0.125 |
| xmv24 | 0.125 |
| dpy19l4 | 0.125 |
| serpina12 | 0.125 |
| slc32a1 | 0.124 |
| ddn | 0.124 |
| camk1d | 0.124 |
| eif2ak4 | 0.124 |
| prl2c3 | 0.124 |
| edn2 | 0.124 |
| rb(2.8)2lub | 0.124 |
| syt5 | 0.124 |
| nrxn2 | 0.124 |
| mmp9 | 0.124 |
| grm6 | 0.124 |
| tg(cag-egfp)d4nagy | 0.124 |
| cacnb2 | 0.123 |
| cnga1 | 0.123 |
| dlgap3 | 0.123 |
| mas1 | 0.123 |
| gipr | 0.123 |
| trpm3 | 0.123 |
| akap6 | 0.123 |
| t(6;13)6ad | 0.123 |
| aff2 | 0.123 |
| ucn3 | 0.123 |
| fgf22 | 0.123 |
| mirn184 | 0.123 |
| prl8a9 | 0.123 |
| ptges | 0.122 |
| pak3 | 0.122 |
| erbb4 | 0.122 |
| mrvi1 | 0.122 |
| glra1 | 0.122 |
| atp2b2 | 0.122 |
| gnai2 | 0.122 |
| syt2 | 0.122 |
| gabrg1 | 0.122 |
| klf9 | 0.122 |
| ric8 | 0.122 |
| rln1 | 0.122 |
| 6330500d04rik | 0.122 |
| cacna1s | 0.121 |
| ppfia2 | 0.121 |
| dtnbp1 | 0.121 |
| rab3gap2 | 0.121 |
| axtex | 0.121 |
| mtnr1a | 0.121 |
| d930020e02rik | 0.121 |
| syt11 | 0.121 |
| rln3 | 0.121 |
| hras1 | 0.121 |
| znrf2 | 0.121 |
| pon1 | 0.121 |
| cbln2 | 0.12 |
| ascl2 | 0.12 |
| lcn4 | 0.12 |
| rps6ka5 | 0.12 |
| shc2 | 0.12 |
| efnb1 | 0.12 |
| prl8a8 | 0.12 |
| pde8a | 0.12 |
| fgf7 | 0.12 |
| mrpl20 | 0.12 |
| gm52 | 0.12 |
| bc063749 | 0.12 |
| p2ry13 | 0.12 |
| grpr | 0.119 |
| dlk1 | 0.119 |
| trpv5 | 0.119 |
| tg(nes-rtta)306rvs | 0.119 |
| ada | 0.119 |
| npw | 0.119 |
| psg23 | 0.119 |
| mest | 0.119 |
| glra3 | 0.119 |
| 4933434i06rik | 0.118 |
| kcnk10 | 0.118 |
| tsga14 | 0.118 |
| t(4;12)47h | 0.118 |
| t(4;12)47h | 0.118 |
| adrb2 | 0.118 |
| rtl1 | 0.118 |
| v1ra5 | 0.118 |
| rtn4rl2 | 0.118 |
| atp2b1 | 0.118 |
| cpeb3 | 0.118 |
| t(4;6)77h | 0.118 |
| kcnmb1 | 0.118 |
| inhbb | 0.118 |
| nlgn3 | 0.118 |
| rph3al | 0.117 |
| nfatc4 | 0.117 |
| rgs10 | 0.117 |
| akr1c18 | 0.117 |
| gnaz | 0.117 |
| crcp | 0.117 |
| ube3a | 0.117 |
| rem1 | 0.117 |
| fgfr1 | 0.117 |
| rasa1 | 0.117 |
| hsd3b5 | 0.117 |
| gad1 | 0.117 |
| cabp2 | 0.116 |
| ucn2 | 0.116 |
| syt8 | 0.116 |
| syp | 0.116 |
| tprgl | 0.116 |
| syt7 | 0.116 |
| prl2b1 | 0.116 |
| igfbp2 | 0.116 |
| disc1 | 0.116 |
| t(6;13)6ad | 0.116 |
| t(4;6)77h | 0.116 |
| grit | 0.116 |
| rxrg | 0.116 |
| prnp | 0.115 |
| ptn | 0.115 |
| wwc1 | 0.115 |
| f2r | 0.115 |
| rapgefl1 | 0.115 |
| fosl2 | 0.115 |
| ptgr2 | 0.115 |
| inhba | 0.115 |
| pcdh8 | 0.115 |
| cox5a | 0.115 |
| calb1 | 0.115 |
| mctp2 | 0.115 |
| mchr1 | 0.115 |
| esr2 | 0.115 |
| pdyn | 0.115 |
| ntrk1 | 0.114 |
| rasd1 | 0.114 |
| add2 | 0.114 |
| slc1a1 | 0.114 |
| car8 | 0.114 |
| d17mit44 | 0.114 |
| rnf39 | 0.114 |
| sytl2 | 0.114 |
| cav1 | 0.114 |
| nr4a3 | 0.114 |
| 1110032a03rik | 0.114 |
| lrfn4 | 0.114 |
| lrfn3 | 0.114 |
| csf2 | 0.114 |
| atp2b4 | 0.114 |
| ccnl1 | 0.113 |
| scgn | 0.113 |
| omp | 0.113 |
| cib1 | 0.113 |
| cib4 | 0.113 |
| stoml1 | 0.113 |
| trpm7 | 0.113 |
| il1r1 | 0.113 |
| s100a1 | 0.113 |
| fkbp1b | 0.113 |
| slc25a12 | 0.113 |
| gdi1 | 0.113 |
| necab1 | 0.113 |
| rab3il1 | 0.112 |
| af357425 | 0.112 |
| af357428 | 0.112 |
| af357426 | 0.112 |
| pde7b | 0.112 |
| snap25 | 0.112 |
| rb(4.12)9bnr | 0.112 |
| rb(4.12)9bnr | 0.112 |
| rb(6.12)3sic | 0.112 |
| rb(6.12)3sic | 0.112 |
| il1rn | 0.112 |
| ppfia1 | 0.112 |
| t(7;15)9h | 0.112 |
| t(7;15)9h | 0.112 |
| irg1 | 0.112 |
| sqstm1 | 0.112 |
| ffar1 | 0.112 |
| dab1 | 0.112 |
| gphn | 0.112 |
| gucy2d | 0.112 |
| cyp21a2-ps | 0.112 |
| scn11a | 0.112 |
| slc6a1 | 0.112 |
| arhgap18 | 0.112 |
| rsrc1 | 0.112 |
| nptn | 0.112 |
| zbtb20 | 0.111 |
| carg1 | 0.111 |
| syt6 | 0.111 |
| anxa4 | 0.111 |
| npff | 0.111 |
| ntng2 | 0.111 |
| plcd1 | 0.111 |
| rasa3 | 0.111 |
| htr1b | 0.111 |
| doc2b | 0.111 |
| atf3 | 0.111 |
| tpcn1 | 0.111 |
| erc1 | 0.111 |
| ptgs2 | 0.111 |
| slc39a9 | 0.111 |
| ghrl | 0.111 |
| prokr1 | 0.111 |
| insr | 0.111 |
| f2rl3 | 0.111 |
| atf4 | 0.111 |
| cnr1 | 0.111 |
| lt1 | 0.11 |
| adcy10 | 0.11 |
| epha6 | 0.11 |
| grk5 | 0.11 |
| prss29 | 0.11 |
| pik3c3 | 0.11 |
| kcnc3 | 0.11 |
| slc2a3 | 0.11 |
| fos | 0.11 |
| trdn | 0.11 |
| dlg3 | 0.11 |
| lyn | 0.11 |
| pon2 | 0.11 |
| trpv3 | 0.11 |
| il1b | 0.11 |
| clstn2 | 0.11 |
| hsd3b4 | 0.11 |
| sez6 | 0.11 |
| glp2r | 0.11 |
| cebpd | 0.11 |
| rasgrp3 | 0.11 |
| nos3 | 0.109 |
| gnas | 0.109 |
| atp10a | 0.109 |
| p2rx6 | 0.109 |
| tro | 0.109 |
| chst10 | 0.109 |
| rps15a | 0.109 |
| rxfp1 | 0.109 |
| fst | 0.109 |
| lif | 0.109 |
| prl2c5 | 0.109 |
| rgs8 | 0.109 |
| axtav | 0.109 |
| nos1 | 0.109 |
| stmn2 | 0.109 |
| ptprz1 | 0.109 |
| gabrr2 | 0.109 |
| gnao1 | 0.109 |
| rap1a | 0.109 |
| ctla2b | 0.109 |
| gad2 | 0.109 |
| slc24a3 | 0.109 |
| plcd2 | 0.109 |
| ppp3cb | 0.108 |
| csk | 0.108 |
| egr4 | 0.108 |
| olfr805 | 0.108 |
| 9330133o14rik | 0.108 |
| gdf15 | 0.108 |
| rasgrp2 | 0.108 |
| 4833403i15rik | 0.108 |
| calm1 | 0.108 |
| ephb3 | 0.108 |
| akr1c14 | 0.108 |
| prok1 | 0.108 |
| inha | 0.108 |
| ntsr1 | 0.108 |
| uchl3 | 0.108 |
| f2rl2 | 0.108 |
| vldlr | 0.108 |
| pla2g6 | 0.108 |
| tnfsf10 | 0.108 |
| panx1 | 0.107 |
| elk1 | 0.107 |
| epha4 | 0.107 |
| nfatc3 | 0.107 |
| sv2c | 0.107 |
| tram2 | 0.107 |
| t(16c3-4;17a2)65dn | 0.107 |
| socs3 | 0.107 |
| p2ry2 | 0.107 |
| grasp | 0.107 |
| rabggta | 0.107 |
| eno2 | 0.107 |
| igsf9 | 0.107 |
| cxcl5 | 0.107 |
| lhb | 0.107 |
| slc6a12 | 0.107 |
| zdhhc3 | 0.107 |
| plcd3 | 0.107 |
| rps6ka4 | 0.107 |
| ncoa2 | 0.107 |
| arpp21 | 0.107 |
| nmu | 0.107 |
| ctsq | 0.107 |
| t(2;4)1go | 0.107 |
| syt3 | 0.106 |
| phlda2 | 0.106 |
| drd2 | 0.106 |
| tacr3 | 0.106 |
| foxp2 | 0.106 |
| tmem37 | 0.106 |
| 6430527g18rik | 0.106 |
| spred1 | 0.106 |
| amigo2 | 0.106 |
| cit | 0.106 |
| rap1gap | 0.106 |
| ntrk3 | 0.106 |
| nrg1 | 0.106 |
| itga5 | 0.106 |
| cnrip1 | 0.106 |
| lrfn2 | 0.106 |
| dgkz | 0.106 |
| sv2b | 0.106 |
| prkcd | 0.105 |
| dmd | 0.105 |
| cacng6 | 0.105 |
| gripap1 | 0.105 |
| mprf | 0.105 |
| cdk5r2 | 0.105 |
| pcp4 | 0.105 |
| avpr1a | 0.105 |
| pth2 | 0.105 |
| frap1 | 0.105 |
| chrna9 | 0.105 |
| abcc8 | 0.105 |
| pthlh | 0.105 |
| catsper1 | 0.105 |
| slc6a7 | 0.105 |
| 8030462n17rik | 0.104 |
| rnf165 | 0.104 |
| c030003d03rik | 0.104 |
| t(2;8)26h | 0.104 |
| calca | 0.104 |
| snca | 0.104 |
| slc38a1 | 0.104 |
| 3-Sep | 0.104 |
| hsd3b6 | 0.104 |
| sv2a | 0.104 |
| scn10a | 0.104 |
| catsper2 | 0.104 |
| mapk14 | 0.104 |
| neto2 | 0.104 |
| anxa5 | 0.104 |
| fosl1 | 0.104 |
| astn1 | 0.104 |
| sct | 0.104 |
| glrb | 0.104 |
| t(x;4)7rl | 0.104 |
| t(x;4)8rl | 0.104 |
| t(x;4)8rl | 0.104 |
| t(x;4)7rl | 0.104 |
| iap2 | 0.104 |
| slc1a6 | 0.104 |
| fgfr2 | 0.104 |
| dbndd1 | 0.103 |
| kcnj4 | 0.103 |
| eif4ebp2 | 0.103 |
| src | 0.103 |
| ngf | 0.103 |
| slc24a6 | 0.103 |
| rap1b | 0.103 |
| mtap1b | 0.103 |
| npy2r | 0.103 |
| csf1 | 0.103 |
| sftpb | 0.103 |
| rcn2 | 0.103 |
| nmur2 | 0.103 |
| eg627648 | 0.103 |
| fstl3 | 0.103 |
| csf3 | 0.103 |
| il1 | 0.103 |
| sftpa1 | 0.103 |
| fgf1 | 0.103 |
| adrbk1 | 0.103 |
| mapk3 | 0.103 |
| gsbs | 0.103 |
| zfp211 | 0.102 |
| gabra2 | 0.102 |
| ptpra | 0.102 |
| rua | 0.102 |
| arg1 | 0.102 |
| gnat3 | 0.102 |
| pik3r1 | 0.102 |
| cyp11b1 | 0.102 |
| glud1 | 0.102 |
| gnai1 | 0.102 |
| t(2;8)2wa | 0.102 |
| bdkrb1 | 0.102 |
| uchl1 | 0.101 |
| rara | 0.101 |
| nf1 | 0.101 |
| prkca | 0.101 |
| akap8 | 0.101 |
| rap2a | 0.101 |
| hpcal1 | 0.101 |
| peg3 | 0.101 |
| atf2 | 0.101 |
| ppif | 0.101 |
| prlh | 0.101 |
| ttpa | 0.101 |
| acvr2a | 0.101 |
| trpv2 | 0.101 |
| hsd3b2 | 0.101 |
| tspo | 0.101 |
| zdbf2 | 0.101 |
| cacna1f | 0.101 |
| ckmt1 | 0.101 |
| nfatc1 | 0.101 |
| asph | 0.101 |
| klk1b5 | 0.101 |
| cnn1 | 0.101 |
| ghsr | 0.101 |
| ptgir | 0.101 |
| cyr61 | 0.101 |
| serpina6 | 0.101 |
| kcnq2 | 0.1 |
| stim1 | 0.1 |
| peg13 | 0.1 |
| amigo1 | 0.1 |
| amigo3 | 0.1 |
| cald1 | 0.1 |
| begain | 0.1 |
| esr1 | 0.1 |
| htr1a | 0.1 |
| kmo | 0.1 |
| plcl1 | 0.1 |
| cyp11b2 | 0.1 |
| hsd17b2 | 0.1 |
| nr4a2 | 0.1 |
| mrgprd | 0.1 |
| mdlk1 | 0.1 |
| d7mit267 | 0.1 |
| si5lq6 | 0.1 |
| nmbr | 0.1 |
| sps | 0.1 |
| lrfn1 | 0.1 |
| ckr | 0.1 |
| gpr182 | 0.1 |
| znrf1 | 0.1 |
| t(2;8)26h | 0.1 |
| chrna10 | 0.1 |
| igfbp3 | 0.1 |
| cyp11a1 | 0.1 |
| accn4 | 0.1 |
| gper | 0.1 |
| oprm1 | 0.1 |
| cacnb4 | 0.1 |
| pln | 0.1 |
| dxmit128 | 0.1 |
| dxmit126 | 0.1 |
| chat | 0.1 |
| calm5 | 0.1 |
