# Supplementary material for: GIT2 Acts as a Potential Keystone Protein in Functional Hypothalamic Networks Associated with Age-Related Phenotypic Changes in Rats
Source: PLoS One. 2012 May 14;7(5):e36975. doi: 10.1371/journal.pone.0036975 (PMC3351446; doi:10.1371/journal.pone.0036975)
Supplement: Table S16 — GeneIndexer latent semantic indexing (LSI) of significantly-regulated ‘Notch signaling’ KEGG pathway. Using the KEGG signaling pathway ‘Notch signaling’ as an input term, a list of the top 1000 implicitly-correlated (LSI correlation score >0.1) was generated using a full genome background list. (DOC) [file pone.0036975.s020.doc]

**Table S16. GeneIndexer latent semantic indexing (LSI) of significantly-regulated ‘Notch signaling’ KEGG pathway.** Using the KEGG signaling pathway ‘Notch signaling’ as an input term, a list of the top 1000 implicitly-correlated (LSI correlation score >0.1) was generated using a full genome background list.

| ***Notch signaling*** |  |
| --- | --- |
|  |  |
| **Protein Symbol** | **LSI correlation score** |
| csl | 0.673 |
| nle1 | 0.662 |
| rfng | 0.654 |
| tg(notch1)a3rko | 0.639 |
| tg(notch1)a4rko | 0.639 |
| eg240055 | 0.631 |
| mfng | 0.631 |
| maml3 | 0.628 |
| dll3 | 0.595 |
| dll1 | 0.593 |
| heyl | 0.585 |
| tg(notch1)a5rko | 0.584 |
| tg(notch1)a7rko | 0.584 |
| hes7 | 0.578 |
| ftl1-rs4 | 0.571 |
| hes2 | 0.554 |
| nrarp | 0.554 |
| hes5 | 0.553 |
| lfng | 0.549 |
| mnotch | 0.547 |
| dtx3 | 0.522 |
| maml1 | 0.515 |
| dtx4 | 0.514 |
| hes1 | 0.512 |
| jag2 | 0.511 |
| hey1 | 0.511 |
| mesp2 | 0.503 |
| ripply2 | 0.503 |
| hes6 | 0.497 |
| jag1 | 0.491 |
| dtx2 | 0.491 |
| notch2 | 0.489 |
| hes3 | 0.488 |
| dtx1 | 0.483 |
| hey2 | 0.476 |
| helt | 0.471 |
| dll4 | 0.47 |
| pofut1 | 0.47 |
| uncx | 0.468 |
| cc2d1b | 0.465 |
| numbl | 0.459 |
| d12mit196 | 0.455 |
| cnksr3 | 0.452 |
| msgn1 | 0.449 |
| neurl2 | 0.446 |
| notch3 | 0.439 |
| aph1c | 0.437 |
| rbpj | 0.422 |
| sbno2 | 0.415 |
| ccdc89 | 0.414 |
| ott | 0.405 |
| notch4 | 0.397 |
| d2mit34 | 0.391 |
| spen | 0.389 |
| numb | 0.388 |
| tcf15 | 0.387 |
| pofut2 | 0.383 |
| d12mit133 | 0.381 |
| rbpjl | 0.378 |
| tle3 | 0.378 |
| ripply1 | 0.369 |
| maml2 | 0.367 |
| d7mit67 | 0.367 |
| mib2 | 0.367 |
| tle2 | 0.364 |
| pdzrn4 | 0.36 |
| mib1 | 0.358 |
| tbx6 | 0.356 |
| ncln | 0.355 |
| dner | 0.354 |
| mettl2 | 0.35 |
| tbx18 | 0.35 |
| jsr | 0.346 |
| neurog1 | 0.346 |
| tle1 | 0.345 |
| st18 | 0.345 |
| mesp1 | 0.341 |
| zfp64 | 0.339 |
| 9530020o07rik | 0.337 |
| gm1968 | 0.337 |
| tle4 | 0.333 |
| alph1 | 0.333 |
| neurl | 0.331 |
| nkap | 0.331 |
| mfap5 | 0.328 |
| sash3 | 0.326 |
| tsta3 | 0.312 |
| 2010106g01rik | 0.312 |
| amy | 0.31 |
| tg(wapnotch4)10rnc | 0.309 |
| neurod4 | 0.304 |
| sbno1 | 0.304 |
| rh | 0.303 |
| dlk1 | 0.303 |
| aph1b | 0.301 |
| aph1a | 0.301 |
| ptf1a | 0.299 |
| adamts17 | 0.295 |
| neurog2 | 0.292 |
| asb11 | 0.29 |
| atoh1 | 0.287 |
| 3110056o03rik | 0.287 |
| notch1 | 0.283 |
| dact1 | 0.279 |
| tg(mmtv-notch4)3rnc | 0.279 |
| aida | 0.279 |
| pear1 | 0.278 |
| tectb | 0.278 |
| lnx2 | 0.277 |
| aes | 0.277 |
| sppl3 | 0.276 |
| nomo1 | 0.276 |
| spry4 | 0.275 |
| klhl6 | 0.274 |
| ncstn | 0.273 |
| ripply3 | 0.273 |
| neurog3 | 0.272 |
| pcdh8 | 0.272 |
| aloxe3 | 0.269 |
| mesdc1 | 0.267 |
| mamld1 | 0.264 |
| etv2 | 0.263 |
| psenen | 0.262 |
| rhbdl3 | 0.259 |
| shisa2 | 0.258 |
| adam10 | 0.257 |
| pdzd4 | 0.255 |
| ascl1 | 0.255 |
| 1110018m03rik | 0.255 |
| d19mit104 | 0.252 |
| stmn2 | 0.252 |
| sp9 | 0.251 |
| 1700023b02rik | 0.25 |
| dact3 | 0.25 |
| ankrd6 | 0.25 |
| cc2d1a | 0.249 |
| fbxw7 | 0.249 |
| d10mit62 | 0.249 |
| d5mit247 | 0.247 |
| d5mit292 | 0.247 |
| nkd1 | 0.246 |
| foxg1 | 0.246 |
| psen2 | 0.245 |
| 4933407p14rik | 0.245 |
| lnx1 | 0.245 |
| tg(tek-cre)12flv | 0.244 |
| eomes | 0.243 |
| snw1 | 0.242 |
| msi1 | 0.242 |
| kctd11 | 0.24 |
| magi3 | 0.24 |
| rspo3 | 0.239 |
| ptcra | 0.239 |
| wdr12 | 0.238 |
| neurod5 | 0.238 |
| ccdc88c | 0.238 |
| axin2 | 0.235 |
| cxxc4 | 0.235 |
| tcf3 | 0.235 |
| zfp488 | 0.234 |
| wnt11 | 0.231 |
| dact2 | 0.231 |
| kremen2 | 0.228 |
| mfap2 | 0.228 |
| irx4 | 0.225 |
| mirn9-1 | 0.225 |
| moxd2 | 0.225 |
| egfl7 | 0.224 |
| lins2 | 0.224 |
| tg(dvl2)7gsb | 0.223 |
| efnb2 | 0.223 |
| lrrfip2 | 0.223 |
| sox1 | 0.223 |
| lincr | 0.223 |
| cer1 | 0.222 |
| rnf138 | 0.221 |
| etv3 | 0.221 |
| gm944 | 0.221 |
| itch | 0.22 |
| daam2 | 0.22 |
| tspan12 | 0.22 |
| try5 | 0.219 |
| tbc1d10c | 0.219 |
| mesdc2 | 0.219 |
| crb2 | 0.219 |
| rp23-136k12.4 | 0.219 |
| anks1 | 0.219 |
| zbed3 | 0.219 |
| tg(ctnnb1)1efu | 0.217 |
| rbm15 | 0.217 |
| myt1l | 0.217 |
| rspo2 | 0.216 |
| sel1l | 0.216 |
| mirn200b | 0.216 |
| magi2 | 0.215 |
| fem1a | 0.215 |
| meox1 | 0.214 |
| neurod1 | 0.214 |
| fzd2 | 0.214 |
| tat | 0.213 |
| ephb4 | 0.213 |
| shoc2 | 0.212 |
| ly76 | 0.211 |
| 2010317e24rik | 0.211 |
| intu | 0.211 |
| fuz | 0.211 |
| b3galtl | 0.211 |
| ormdl1 | 0.211 |
| rras | 0.211 |
| hand1 | 0.209 |
| cntn6 | 0.209 |
| magi1 | 0.209 |
| socs4 | 0.208 |
| dock3 | 0.208 |
| nlk-ps1 | 0.208 |
| d2mit444 | 0.208 |
| vgll3 | 0.208 |
| tcf7 | 0.207 |
| ifaprc2 | 0.206 |
| lefty1 | 0.205 |
| iapls3-41 | 0.205 |
| tbl1xr1 | 0.205 |
| fat2 | 0.205 |
| lzic | 0.205 |
| spata5 | 0.204 |
| mirn146 | 0.204 |
| scube2 | 0.203 |
| dkk4 | 0.203 |
| epha4 | 0.203 |
| tbl1x | 0.203 |
| 4933433p14rik | 0.202 |
| fzd8 | 0.202 |
| nodal | 0.202 |
| il17rd | 0.201 |
| plekhm3 | 0.201 |
| sh2d2a | 0.201 |
| spib | 0.201 |
| smo | 0.201 |
| fabp7 | 0.201 |
| crtc1 | 0.201 |
| cnpy1 | 0.201 |
| apbb2 | 0.2 |
| nradd | 0.2 |
| fzd6 | 0.2 |
| pcdhb1 | 0.199 |
| pcdhb4 | 0.199 |
| 9830130m13rik | 0.199 |
| hand2 | 0.198 |
| wnt2b | 0.198 |
| grem1 | 0.198 |
| tmed10 | 0.197 |
| d12sut2e | 0.197 |
| spopl | 0.197 |
| nlk | 0.196 |
| twist2 | 0.196 |
| fzd5 | 0.196 |
| pdzrn3 | 0.196 |
| crb3 | 0.196 |
| irx3 | 0.196 |
| arhgef15 | 0.196 |
| spred3 | 0.195 |
| mirn124a-2 | 0.195 |
| neurod6 | 0.195 |
| ccdc100 | 0.195 |
| cdon | 0.195 |
| a530064d06rik | 0.195 |
| disp1 | 0.194 |
| svet1 | 0.194 |
| apcdd1 | 0.194 |
| tmem9 | 0.194 |
| dusp4 | 0.194 |
| rhbdl2 | 0.194 |
| mirn29b-2 | 0.194 |
| rnf128 | 0.194 |
| spred2 | 0.194 |
| ikzf1 | 0.193 |
| wnt8a | 0.193 |
| dixdc1 | 0.193 |
| fzd7 | 0.193 |
| tg(wnt3)7gsb | 0.193 |
| scrt1 | 0.192 |
| lrp4 | 0.192 |
| myt1 | 0.192 |
| crlf2 | 0.192 |
| tmed2 | 0.192 |
| cby | 0.191 |
| pygo1 | 0.191 |
| pcdhga1 | 0.191 |
| lef1 | 0.191 |
| h13 | 0.19 |
| heg1 | 0.19 |
| tg(krt5-cre)1tak | 0.19 |
| mirn124a-1 | 0.19 |
| sp8 | 0.189 |
| pax1 | 0.189 |
| ephb2 | 0.189 |
| il17re | 0.188 |
| socs5 | 0.188 |
| ephb3 | 0.188 |
| psen1 | 0.188 |
| asb15 | 0.188 |
| ferd3l | 0.188 |
| dvl3 | 0.188 |
| kremen1 | 0.188 |
| 1700009n14rik | 0.188 |
| dand5 | 0.188 |
| nkd2 | 0.188 |
| ppp2r2d | 0.187 |
| nxn | 0.187 |
| htatip | 0.187 |
| spry3 | 0.187 |
| edar | 0.186 |
| bmp10 | 0.186 |
| amy2-2 | 0.186 |
| cfc1 | 0.185 |
| sh2d3c | 0.185 |
| mirn124a-3 | 0.185 |
| lrp6 | 0.185 |
| prr16 | 0.185 |
| d10jhu81e | 0.185 |
| lefty2 | 0.184 |
| foxn4 | 0.184 |
| adam9 | 0.184 |
| il34 | 0.184 |
| d10mit109 | 0.184 |
| rnf111 | 0.184 |
| e430004n04rik | 0.184 |
| efnb1 | 0.184 |
| trim71 | 0.183 |
| caprin2 | 0.183 |
| cnksr1 | 0.183 |
| rgs5 | 0.183 |
| mtap2 | 0.183 |
| bhlhb3 | 0.183 |
| disp2 | 0.183 |
| snx5 | 0.183 |
| sufu | 0.182 |
| ubqln2 | 0.182 |
| 9130404h23rik | 0.182 |
| mirn196a-1 | 0.182 |
| mirn196a-2 | 0.182 |
| flrt2 | 0.182 |
| tcf12 | 0.182 |
| apbb1 | 0.182 |
| yap1 | 0.182 |
| wdr26 | 0.181 |
| d930005d10rik | 0.181 |
| nhlh1 | 0.181 |
| flrt1 | 0.181 |
| fzd1 | 0.181 |
| efnb3 | 0.181 |
| dok3 | 0.181 |
| rspo4 | 0.18 |
| rtp4 | 0.18 |
| tg(lef1)1efu | 0.18 |
| dvl2 | 0.18 |
| prox1 | 0.18 |
| ovol1 | 0.18 |
| d8mit98 | 0.18 |
| d8mit297 | 0.18 |
| 4933411k20rik | 0.18 |
| atoh7 | 0.18 |
| foxh1 | 0.18 |
| smarcd3 | 0.18 |
| d17mit7 | 0.179 |
| d2mit434 | 0.179 |
| frat2 | 0.179 |
| 5730410i19rik | 0.179 |
| d12mit141 | 0.179 |
| lax1 | 0.179 |
| cend1 | 0.179 |
| iba1 | 0.179 |
| nkx2-2 | 0.179 |
| sit1 | 0.179 |
| lgr5 | 0.179 |
| shcbp1 | 0.179 |
| dtx3l | 0.179 |
| sh2b3 | 0.179 |
| axin1 | 0.178 |
| lrrtm3 | 0.178 |
| ebf1 | 0.178 |
| e030049g20rik | 0.178 |
| trim33 | 0.178 |
| tcf4 | 0.178 |
| appl2 | 0.178 |
| dullard | 0.178 |
| ksr2 | 0.178 |
| sdcbp | 0.178 |
| loc667882 | 0.178 |
| mirn24-2 | 0.177 |
| cbfa2t2 | 0.177 |
| hif1an | 0.177 |
| rb(4.12)9bnr | 0.177 |
| rb(4.12)9bnr | 0.177 |
| rb(6.12)3sic | 0.177 |
| rb(6.12)3sic | 0.177 |
| mirn24-1 | 0.176 |
| gpr177 | 0.176 |
| ptk7 | 0.176 |
| ptch2 | 0.176 |
| wwp1 | 0.175 |
| ppil5 | 0.175 |
| msc | 0.175 |
| ascl3 | 0.175 |
| zfp641 | 0.174 |
| zfyve9 | 0.174 |
| rspo1 | 0.174 |
| fjx1 | 0.174 |
| ubash3b | 0.174 |
| scube3 | 0.174 |
| spred1 | 0.174 |
| crim2 | 0.173 |
| ncor2 | 0.173 |
| barhl2 | 0.173 |
| pik3ip1 | 0.173 |
| cdc42bpa | 0.173 |
| sox2 | 0.173 |
| mdfi | 0.173 |
| tubb3 | 0.173 |
| tagln | 0.172 |
| 2700049a03rik | 0.172 |
| kif27 | 0.172 |
| tinagl | 0.171 |
| spry1 | 0.171 |
| fbxl6 | 0.171 |
| stap1 | 0.171 |
| cul4b | 0.171 |
| depdc6 | 0.171 |
| asb3 | 0.171 |
| gata3 | 0.171 |
| nes | 0.171 |
| akirin1 | 0.171 |
| bambi | 0.17 |
| boc | 0.17 |
| ptchd3 | 0.17 |
| metrn | 0.17 |
| foxc2 | 0.17 |
| dvl1 | 0.169 |
| snai2 | 0.169 |
| mirn1-2 | 0.169 |
| daam1 | 0.169 |
| alx4 | 0.168 |
| wsb2 | 0.168 |
| d2mit41 | 0.168 |
| c330002i19rik | 0.168 |
| sp5 | 0.167 |
| lyve1 | 0.167 |
| speg | 0.167 |
| socs6 | 0.167 |
| shc4 | 0.167 |
| sox17 | 0.167 |
| lgr6 | 0.167 |
| lime1 | 0.167 |
| mirn17 | 0.167 |
| ncor1 | 0.167 |
| prickle1 | 0.167 |
| scx | 0.167 |
| cd19 | 0.167 |
| gsk3a | 0.167 |
| ecsit | 0.166 |
| tm2d1 | 0.166 |
| pycr2 | 0.166 |
| fzd9 | 0.166 |
| zfp383 | 0.166 |
| ripk4 | 0.166 |
| notum | 0.166 |
| cby1 | 0.166 |
| sh2d4a | 0.166 |
| olig2 | 0.166 |
| spry2 | 0.165 |
| zranb1 | 0.165 |
| fzd3 | 0.165 |
| d10ertd610e | 0.165 |
| socs7 | 0.165 |
| fzd4 | 0.165 |
| eeld2 | 0.165 |
| eeld1 | 0.165 |
| trim11 | 0.165 |
| clstn3 | 0.165 |
| dab1 | 0.165 |
| appl1 | 0.165 |
| tal1 | 0.165 |
| ilkap | 0.164 |
| hesx1 | 0.164 |
| tdgf1 | 0.164 |
| tmeff1 | 0.164 |
| adam12 | 0.164 |
| tcf21 | 0.164 |
| glis2 | 0.164 |
| ror2 | 0.164 |
| gli2 | 0.164 |
| churc1 | 0.164 |
| vangl1 | 0.164 |
| 3632451o06rik | 0.164 |
| klhl30 | 0.164 |
| tcfe2a | 0.163 |
| svep1 | 0.163 |
| irx2 | 0.163 |
| skp1a | 0.163 |
| ppp2r5c | 0.163 |
| foxd2 | 0.163 |
| mirn23b | 0.163 |
| zmiz1 | 0.163 |
| ctnnd2 | 0.163 |
| adam19 | 0.163 |
| d12mit280 | 0.163 |
| neuna60 | 0.163 |
| trappc9 | 0.163 |
| dub1 | 0.162 |
| shf | 0.162 |
| myl7 | 0.162 |
| fgf17 | 0.162 |
| act1 | 0.162 |
| sap30 | 0.162 |
| pak1ip1 | 0.162 |
| sap130 | 0.162 |
| mapk8ip2 | 0.162 |
| tbc1d15 | 0.162 |
| trat1 | 0.161 |
| wnt3a | 0.161 |
| vasn | 0.161 |
| fzd10 | 0.161 |
| stk40 | 0.161 |
| mirn181a-2 | 0.161 |
| epgn | 0.161 |
| calcoco1 | 0.161 |
| tcrb-v12 | 0.161 |
| irak2 | 0.161 |
| ephb6 | 0.161 |
| bhlhb8 | 0.16 |
| map4k5 | 0.16 |
| edil3 | 0.16 |
| mirn29b-1 | 0.16 |
| myocd | 0.16 |
| rgnef | 0.16 |
| bmx | 0.16 |
| cbll1 | 0.16 |
| ikzf3 | 0.16 |
| dok2 | 0.16 |
| epha1 | 0.16 |
| bambi-ps1 | 0.16 |
| bcl9l | 0.16 |
| apba2 | 0.16 |
| cbfa2t3 | 0.16 |
| lgl | 0.16 |
| tax1bp3 | 0.16 |
| tnk1 | 0.159 |
| dkk2 | 0.159 |
| arhgef4 | 0.159 |
| lrrfip1 | 0.159 |
| lrp8 | 0.158 |
| tg(krt14-cre)1efu | 0.158 |
| nrxn2 | 0.158 |
| pygo2 | 0.158 |
| snx13 | 0.158 |
| zfyve16 | 0.158 |
| stap2 | 0.158 |
| foxd1 | 0.158 |
| wdr68 | 0.158 |
| loc641201 | 0.158 |
| bhlhb2 | 0.158 |
| nkx3-2 | 0.157 |
| pcdha7 | 0.157 |
| pcdha10 | 0.157 |
| gipc2 | 0.157 |
| mesd | 0.157 |
| gdf11 | 0.157 |
| acta2 | 0.157 |
| tas2r136 | 0.157 |
| ccdc50 | 0.157 |
| ubqln1 | 0.157 |
| rnf41 | 0.157 |
| ppp2r5b | 0.157 |
| isl1 | 0.156 |
| d2mit436 | 0.156 |
| tbx4 | 0.156 |
| tbx1 | 0.156 |
| akirin2 | 0.156 |
| mirn20a | 0.156 |
| atoh8 | 0.156 |
| tom1l1 | 0.156 |
| pik3ap1 | 0.156 |
| gli1 | 0.156 |
| map2k1ip1 | 0.156 |
| irx1 | 0.156 |
| cd3d | 0.156 |
| 1500001a10rik | 0.156 |
| bloc1s1 | 0.155 |
| acvr1c | 0.155 |
| jub | 0.155 |
| gdf1 | 0.155 |
| snd1 | 0.155 |
| gcm1 | 0.155 |
| phlppl | 0.155 |
| 1110012m11rik | 0.155 |
| v2r8 | 0.155 |
| sla2 | 0.155 |
| gas1 | 0.154 |
| l1md-a5 | 0.154 |
| gfi1 | 0.154 |
| scube1 | 0.154 |
| akt1s1 | 0.154 |
| barx1 | 0.154 |
| frs2 | 0.154 |
| erbb2ip | 0.154 |
| thnsl2 | 0.154 |
| lin28 | 0.154 |
| dusp9 | 0.154 |
| dub2 | 0.154 |
| meox2 | 0.154 |
| 5430433g21rik | 0.153 |
| ngef | 0.153 |
| 4933407c03rik | 0.153 |
| rin1 | 0.153 |
| dok4 | 0.153 |
| rtkn | 0.153 |
| bhlhb5 | 0.153 |
| cd3g | 0.153 |
| tbx3 | 0.153 |
| gdf3 | 0.153 |
| rbm19 | 0.153 |
| mirn21 | 0.153 |
| cnksr2 | 0.153 |
| crtc3 | 0.153 |
| clnk | 0.153 |
| ephb1 | 0.153 |
| ryk | 0.152 |
| shb | 0.152 |
| wnt10b | 0.152 |
| tbr1 | 0.152 |
| dyrk1a | 0.152 |
| slc35d2 | 0.152 |
| gng13 | 0.152 |
| dusp14 | 0.152 |
| bace1 | 0.152 |
| plekhg5 | 0.152 |
| uxs1 | 0.152 |
| retnla | 0.152 |
| noc3l | 0.152 |
| aup1 | 0.151 |
| cul1 | 0.151 |
| tead4 | 0.151 |
| ly6g6f | 0.151 |
| rb(8.12)5bnr | 0.151 |
| usp6nl | 0.151 |
| flot2 | 0.151 |
| txk | 0.151 |
| grem2 | 0.151 |
| hivep3 | 0.151 |
| pak4 | 0.151 |
| tbx20 | 0.151 |
| hsh2d | 0.151 |
| apbb3 | 0.151 |
| spop | 0.151 |
| id4 | 0.151 |
| scrt2 | 0.151 |
| nr2e1 | 0.151 |
| tle6 | 0.15 |
| anks1b | 0.15 |
| stk36 | 0.15 |
| clec4b1 | 0.15 |
| lzts2 | 0.15 |
| csnk1e | 0.15 |
| tnfrsf21 | 0.15 |
| cd3e | 0.15 |
| mirn181a-1 | 0.15 |
| sox4 | 0.15 |
| map4k4 | 0.15 |
| epha10 | 0.15 |
| apba1 | 0.15 |
| mtap2k | 0.15 |
| nenf | 0.15 |
| epha8 | 0.149 |
| smok2a | 0.149 |
| nrxn3 | 0.149 |
| gde1 | 0.149 |
| zc3h8 | 0.149 |
| bmper | 0.149 |
| d11bwg0517e | 0.149 |
| asb6 | 0.149 |
| wsb1 | 0.149 |
| cpz | 0.149 |
| kndc1 | 0.149 |
| slfn2 | 0.149 |
| npn2 | 0.149 |
| porcn | 0.148 |
| chd8 | 0.148 |
| tmem181 | 0.148 |
| gcn5l2 | 0.148 |
| foxl1 | 0.148 |
| mapk8ip1 | 0.148 |
| 5830411n06rik | 0.148 |
| ksr1 | 0.148 |
| rgs13 | 0.148 |
| adam15 | 0.148 |
| chn2 | 0.148 |
| paqr6 | 0.148 |
| paqr9 | 0.148 |
| map4k1 | 0.148 |
| tbx5 | 0.148 |
| vmn2r122 | 0.148 |
| foxc1 | 0.148 |
| pak6 | 0.148 |
| pcaf | 0.148 |
| gipc3 | 0.148 |
| rbm9 | 0.148 |
| iapls3-10 | 0.148 |
| sh3pxd2a | 0.147 |
| cxxc5 | 0.147 |
| pax5 | 0.147 |
| wnt8b | 0.147 |
| cntfr | 0.147 |
| barhl1 | 0.147 |
| sh3rf1 | 0.147 |
| pcdh18 | 0.147 |
| dpy19l4 | 0.147 |
| ralgps1 | 0.147 |
| pcdhgb1 | 0.147 |
| homer3 | 0.147 |
| 1110006o17rik | 0.147 |
| gm397 | 0.147 |
| bq559217 | 0.147 |
| nck2 | 0.147 |
| neurod2 | 0.147 |
| 9130404d14rik | 0.147 |
| mirnlet7c-1 | 0.147 |
| mirnlet7c-2 | 0.147 |
| eid2 | 0.146 |
| lrrtm4 | 0.146 |
| lrrtm2 | 0.146 |
| appbp2 | 0.146 |
| irx5 | 0.146 |
| gprin1 | 0.146 |
| adam17 | 0.146 |
| ndac | 0.146 |
| tcrbe | 0.146 |
| pcdha5 | 0.146 |
| pcdha11 | 0.146 |
| zfp191 | 0.146 |
| pea15b | 0.146 |
| smarcc1 | 0.146 |
| dok6 | 0.146 |
| ikzf4 | 0.146 |
| runx1t1 | 0.146 |
| pitx2 | 0.146 |
| sorbs3 | 0.146 |
| fem1c | 0.146 |
| zcchc11 | 0.146 |
| stx19 | 0.146 |
| frs3 | 0.145 |
| tg(nes-rtta)306rvs | 0.145 |
| grit | 0.145 |
| kif7 | 0.145 |
| d10mit1 | 0.145 |
| mirn155 | 0.145 |
| wnt10a | 0.145 |
| rftn1 | 0.145 |
| 2610018g03rik | 0.145 |
| wnt2 | 0.145 |
| rnf125 | 0.145 |
| gsc | 0.145 |
| nepn | 0.145 |
| rorc | 0.145 |
| gigyf1 | 0.145 |
| arrdc2 | 0.145 |
| btrc | 0.145 |
| celsr1 | 0.145 |
| creg1 | 0.144 |
| lmo4 | 0.144 |
| mdfic | 0.144 |
| asb2 | 0.144 |
| zfp414 | 0.144 |
| lrrn3 | 0.144 |
| map3k12 | 0.144 |
| tm2d2 | 0.144 |
| traf7 | 0.144 |
| nov | 0.144 |
| zfp503 | 0.144 |
| aplp1 | 0.144 |
| angptl6 | 0.144 |
| ppp2r5e | 0.144 |
| olfr3 | 0.144 |
| dhh | 0.144 |
| olfr63 | 0.144 |
| lamb1-2 | 0.144 |
| rhov | 0.144 |
| tnfrsf19 | 0.144 |
| arl8a | 0.144 |
| rapgef2 | 0.144 |
| wnt5a | 0.144 |
| ppp2r5a | 0.143 |
| cntn1 | 0.143 |
| trafd1 | 0.143 |
| zfp74 | 0.143 |
| pag1 | 0.143 |
| itgb1bp3 | 0.143 |
| tmem149 | 0.143 |
| stam | 0.143 |
| ppapdc3 | 0.143 |
| siglec15 | 0.143 |
| ubash3a | 0.143 |
| jarid2 | 0.143 |
| afap1l2 | 0.143 |
| tcf25 | 0.143 |
| egfl6 | 0.143 |
| aplp2 | 0.143 |
| ube2a | 0.143 |
| tg(zfp38)d1htz | 0.143 |
| tg(zfp38)y7htz | 0.143 |
| tg(zfp38)b8htz | 0.143 |
| tg(zfp38)a4htz | 0.143 |
| trib1 | 0.142 |
| d230025d16rik | 0.142 |
| ptch1 | 0.142 |
| grap | 0.142 |
| megf9 | 0.142 |
| dice1 | 0.142 |
| krt17 | 0.142 |
| vgll2 | 0.142 |
| snx33 | 0.142 |
| 4933407n01rik | 0.142 |
| npdc1 | 0.142 |
| tax1bp1 | 0.142 |
| klk1b4 | 0.142 |
| lrig1 | 0.142 |
| wif1 | 0.142 |
| apc2 | 0.141 |
| irx6 | 0.141 |
| 3930401k13rik | 0.141 |
| insc | 0.141 |
| ulk2 | 0.141 |
| ifitm3 | 0.141 |
| sh2b2 | 0.141 |
| sox7 | 0.141 |
| rcor2 | 0.141 |
| wnt7a | 0.141 |
| nrp | 0.141 |
| fgf4 | 0.141 |
| cblc | 0.141 |
| tie1 | 0.141 |
| cnih | 0.141 |
| sgsm1 | 0.141 |
| irf2bp1 | 0.141 |
| npcd | 0.141 |
| mxd1 | 0.141 |
| isl2 | 0.141 |
| otud7b | 0.141 |
| ebf2 | 0.14 |
| tbx2 | 0.14 |
| tcfap4 | 0.14 |
| smyd1 | 0.14 |
| fbxw5 | 0.14 |
| itga8 | 0.14 |
| fgf15 | 0.14 |
| fgf10 | 0.14 |
| zbtb7b | 0.14 |
| hhip | 0.14 |
| cfd | 0.14 |
| smurf1 | 0.14 |
| d7mit285 | 0.14 |
| d7mit134 | 0.14 |
| tead3 | 0.14 |
| nbl1 | 0.14 |
| lrrc19 | 0.14 |
| rasd1 | 0.14 |
| mirn126 | 0.14 |
| prox2 | 0.14 |
| dusp6 | 0.14 |
| loc232077 | 0.14 |
| mirn93 | 0.14 |
| elf1 | 0.14 |
| ptchd2 | 0.139 |
| ab030242 | 0.139 |
| lbx1 | 0.139 |
| ube2b-rs1 | 0.139 |
| batf | 0.139 |
| iqgap3 | 0.139 |
| rim4 | 0.139 |
| kif3a | 0.139 |
| rtp1 | 0.139 |
| mirn142 | 0.139 |
| mirn328 | 0.139 |
| rab23 | 0.139 |
| mirn203 | 0.139 |
| ly6a | 0.139 |
| sh2b1 | 0.139 |
| mirn143 | 0.139 |
| asb9 | 0.139 |
| cmtm3 | 0.139 |
| en1 | 0.139 |
| il20rb | 0.139 |
| reck | 0.139 |
| eg628779 | 0.139 |
| herpud1 | 0.139 |
| nrk | 0.139 |
| depdc2 | 0.139 |
| cdgap | 0.138 |
| rit2 | 0.138 |
| mirn877 | 0.138 |
| snip1 | 0.138 |
| rhebl1 | 0.138 |
| ubr1 | 0.138 |
| glrx3 | 0.138 |
| cdc40 | 0.138 |
| itsn2 | 0.138 |
| skap1 | 0.138 |
| zc3h12a | 0.138 |
| frat1 | 0.138 |
| dub4 | 0.138 |
| ppp1r16b | 0.138 |
| 2410018c17rik | 0.138 |
| pias4 | 0.138 |
| cdx4 | 0.138 |
| tg(lck-cre)i57jxm | 0.138 |
| ms4a4b | 0.138 |
| wnt4 | 0.138 |
| lmx1a | 0.138 |
| tcrd-c | 0.137 |
| wnt9a | 0.137 |
| vangl2 | 0.137 |
| d4mit304 | 0.137 |
| gpr149 | 0.137 |
| wdr34 | 0.137 |
| mirn206 | 0.137 |
| invs | 0.137 |
| ankrd28 | 0.137 |
| plekha2 | 0.137 |
| mirn145 | 0.137 |
| rgs3 | 0.137 |
| wwtr1 | 0.137 |
| mv | 0.137 |
| cdc42bpb | 0.137 |
| rapgefl1 | 0.137 |
| grb14 | 0.137 |
| smurf2 | 0.137 |
| hdac9 | 0.137 |
| mirn200a | 0.137 |
| nkx6-1 | 0.137 |
| ph | 0.137 |
| elf5 | 0.137 |
| sin3a | 0.137 |
| itgb8 | 0.137 |
| pou3f3 | 0.137 |
| wnt3 | 0.137 |
| mirn1-1 | 0.136 |
| nr2f2 | 0.136 |
| gpsm1 | 0.136 |
| twist1 | 0.136 |
| zfyve28 | 0.136 |
| cm | 0.136 |
| 9430023l20rik | 0.136 |
| nfkbid | 0.136 |
| phlpp | 0.136 |
| commd6 | 0.136 |
| arrdc3 | 0.136 |
| olig1 | 0.136 |
| d6mit296 | 0.136 |
| d11mit215 | 0.136 |
| map4k3 | 0.136 |
| rit1 | 0.136 |
| cd300lb | 0.136 |
| gab3 | 0.136 |
| ppp1r12c | 0.136 |
| phf12 | 0.136 |
| trim14 | 0.136 |
| otud1 | 0.136 |
| dock4 | 0.136 |
| tmem204 | 0.136 |
| diras1 | 0.136 |
| lx | 0.136 |
| gpr124 | 0.136 |
| arid3b | 0.136 |
| ambra1 | 0.136 |
| megf6 | 0.136 |
| mark4 | 0.136 |
| wnt6 | 0.136 |
| uchl5 | 0.135 |
| mapbpip | 0.135 |
| krt19 | 0.135 |
| stub1 | 0.135 |
| mirn221 | 0.135 |
| mirn222 | 0.135 |
| kank1 | 0.135 |
| 5430435g22rik | 0.135 |
| paqr3 | 0.135 |
| dapp1 | 0.135 |
| vezf1 | 0.135 |
| trp2 | 0.135 |
| nedd9 | 0.135 |
| dnahc11 | 0.135 |
| mirn106b | 0.135 |
| rcan1 | 0.135 |
| ccar1 | 0.135 |
| hhat | 0.135 |
| golga2 | 0.135 |
| snai1 | 0.135 |
| mtch1 | 0.135 |
| cmtm8 | 0.135 |
| loc434156 | 0.135 |
| mapk4 | 0.135 |
