# Supplementary material for: GIT2 Acts as a Potential Keystone Protein in Functional Hypothalamic Networks Associated with Age-Related Phenotypic Changes in Rats
Source: PLoS One. 2012 May 14;7(5):e36975. doi: 10.1371/journal.pone.0036975 (PMC3351446; doi:10.1371/journal.pone.0036975)
Supplement: Table S17 — GeneIndexer latent semantic indexing (LSI) of significantly-regulated ‘VEGF signaling’ KEGG pathway. Using the KEGG signaling pathway ‘VEGF signaling’ as an input term, a list of the top 1000 implicitly-correlated (LSI correlation score >0.1) was generated using a full genome background list. (DOC) [file pone.0036975.s021.doc]

**Table S17. GeneIndexer latent semantic indexing (LSI) of significantly-regulated ‘VEGF signaling’ KEGG pathway.** Using the KEGG signaling pathway ‘VEGF signaling’ as an input term, a list of the top 1000 implicitly-correlated (LSI correlation score >0.1) was generated using a full genome background list.

| ***VEGF signaling*** |  |
| --- | --- |
|  |  |
| **Protein Symbol** | **LSI correlation score** |
| vash2 | 0.591 |
| mirn126 | 0.566 |
| bai2 | 0.55 |
| vash1 | 0.514 |
| mmtg2 | 0.504 |
| mmtg1 | 0.504 |
| d4mit348 | 0.504 |
| vegfb | 0.451 |
| rasip1 | 0.45 |
| sema3g | 0.441 |
| prok1 | 0.44 |
| ang2 | 0.434 |
| bai3 | 0.433 |
| pigf | 0.424 |
| nrarp | 0.424 |
| lamb1-2 | 0.411 |
| wars2 | 0.41 |
| klhl20 | 0.409 |
| pgf | 0.409 |
| git2 | 0.402 |
| vezf1 | 0.4 |
| apold1 | 0.396 |
| angpt4 | 0.394 |
| map3k6 | 0.387 |
| nus1 | 0.381 |
| paip2 | 0.38 |
| figf | 0.375 |
| nrp2 | 0.369 |
| cxcl17 | 0.369 |
| flt4 | 0.368 |
| esm1 | 0.366 |
| card10 | 0.365 |
| angptl6 | 0.363 |
| nrp1 | 0.355 |
| tm4sf5 | 0.355 |
| edil3 | 0.354 |
| prokr1 | 0.351 |
| angptl1 | 0.348 |
| vegfc | 0.341 |
| plvap | 0.339 |
| amotl2 | 0.337 |
| plxnd1 | 0.337 |
| aggf1 | 0.333 |
| emcn | 0.332 |
| dll4 | 0.331 |
| 1110006o17rik | 0.331 |
| tie1 | 0.327 |
| egfl7 | 0.32 |
| plxna1 | 0.317 |
| ang4 | 0.311 |
| ccbe1 | 0.309 |
| shb | 0.309 |
| il17rc | 0.308 |
| zfp36l1 | 0.308 |
| sema3f | 0.307 |
| sema3a | 0.306 |
| hif3a | 0.304 |
| arhgap22 | 0.303 |
| metrn | 0.302 |
| mmtg3 | 0.3 |
| flt1 | 0.296 |
| fibp | 0.294 |
| fgfbp3 | 0.292 |
| amot | 0.292 |
| sema4a | 0.292 |
| ang | 0.291 |
| angpt2 | 0.29 |
| amotl1 | 0.288 |
| tsp2 | 0.288 |
| lyve1 | 0.288 |
| gpr4 | 0.288 |
| d4mit214 | 0.287 |
| sh2d2a | 0.287 |
| sema3d | 0.284 |
| rgs5 | 0.284 |
| zfp36l2 | 0.284 |
| smoc2 | 0.283 |
| prokr2 | 0.281 |
| serpinf1 | 0.277 |
| sema3c | 0.275 |
| ptk7 | 0.272 |
| tinagl | 0.269 |
| plxna3 | 0.269 |
| col18a1 | 0.268 |
| bmx | 0.267 |
| pik3r6 | 0.267 |
| ccdc88a | 0.267 |
| hif1an | 0.267 |
| gpr124 | 0.265 |
| rcan2 | 0.264 |
| spry4 | 0.263 |
| scye1 | 0.263 |
| lect1 | 0.263 |
| egln1 | 0.262 |
| sema3b | 0.261 |
| csda | 0.261 |
| plxdc1 | 0.261 |
| epas1 | 0.26 |
| dear1 | 0.26 |
| serpina4-ps1 | 0.26 |
| angpt1 | 0.26 |
| retnla | 0.258 |
| tymp | 0.257 |
| plxna2 | 0.257 |
| pdgfd | 0.256 |
| vasn | 0.255 |
| eng | 0.254 |
| 1500041n16rik | 0.254 |
| tek | 0.253 |
| shc2 | 0.253 |
| pfkfb4 | 0.252 |
| egln3 | 0.252 |
| ddx47 | 0.252 |
| angptl2 | 0.251 |
| fer1l3 | 0.251 |
| ephb4 | 0.251 |
| sema6d | 0.25 |
| sema3e | 0.25 |
| sucnr1 | 0.249 |
| rasgrp3 | 0.248 |
| egln2 | 0.248 |
| yes1 | 0.248 |
| plekhg5 | 0.248 |
| vbp1 | 0.247 |
| robo4 | 0.246 |
| nudt6 | 0.246 |
| tnmd | 0.245 |
| tead4 | 0.245 |
| tg(krt5-cre)1tak | 0.245 |
| il20 | 0.244 |
| ddit4 | 0.244 |
| bai1 | 0.244 |
| cdh5 | 0.243 |
| narfl | 0.243 |
| pik3c2a | 0.242 |
| hsp86-ps2 | 0.241 |
| elk3 | 0.241 |
| rhod | 0.24 |
| prok2 | 0.24 |
| fes | 0.24 |
| rcan1 | 0.239 |
| 4932409i22rik | 0.239 |
| eg244911 | 0.239 |
| 3300001a09rik | 0.239 |
| nol7 | 0.238 |
| gdf2 | 0.238 |
| hdac7 | 0.237 |
| tg(tek-cre)12flv | 0.235 |
| chrdl1 | 0.234 |
| prdm6 | 0.233 |
| cyr61 | 0.233 |
| pcdh12 | 0.233 |
| spred2 | 0.232 |
| megf10 | 0.232 |
| nr4a3 | 0.231 |
| cd248 | 0.231 |
| aamp | 0.23 |
| adamts8 | 0.229 |
| prkd1 | 0.229 |
| pdgfc | 0.229 |
| sema4d | 0.229 |
| tctex1d4 | 0.228 |
| stk16 | 0.227 |
| rspo3 | 0.226 |
| cdc42ep2 | 0.226 |
| acbd6 | 0.225 |
| socs4 | 0.225 |
| hdgf | 0.224 |
| prkd2 | 0.223 |
| 8430408g22rik | 0.223 |
| elf2 | 0.223 |
| dcbld2 | 0.223 |
| fzd4 | 0.222 |
| sphk2 | 0.221 |
| setd3 | 0.221 |
| stra13 | 0.221 |
| ripk4 | 0.219 |
| 2010001j22rik | 0.218 |
| prl7d1 | 0.218 |
| sema4f | 0.218 |
| lpar3 | 0.218 |
| sphkap | 0.217 |
| lbh | 0.217 |
| zc3h12a | 0.217 |
| mirn199a-1 | 0.216 |
| hexim2 | 0.216 |
| scube3 | 0.215 |
| acvrl1 | 0.214 |
| plxdc2 | 0.214 |
| l1md-a5 | 0.214 |
| usp20 | 0.213 |
| rbm3 | 0.213 |
| mapk13 | 0.213 |
| thbs2 | 0.212 |
| pak1ip1 | 0.211 |
| esam1 | 0.211 |
| plxna4 | 0.211 |
| car9 | 0.21 |
| adamts1 | 0.209 |
| grlf1 | 0.209 |
| bud31 | 0.209 |
| racgap1 | 0.209 |
| plxnb1 | 0.209 |
| ptprb | 0.208 |
| ptp4a3 | 0.208 |
| zc3h10 | 0.208 |
| ptpn23 | 0.207 |
| id1 | 0.207 |
| ddr2 | 0.207 |
| fgfbp1 | 0.206 |
| s1pr3 | 0.206 |
| elavl1 | 0.206 |
| spry3 | 0.206 |
| cited2 | 0.205 |
| adamts12 | 0.205 |
| glmn | 0.205 |
| gm944 | 0.204 |
| antxr1 | 0.204 |
| narg1 | 0.204 |
| spred1 | 0.203 |
| dstn | 0.202 |
| nab2 | 0.202 |
| thpo | 0.201 |
| efnb2 | 0.2 |
| maz | 0.2 |
| notch4 | 0.2 |
| il17rd | 0.2 |
| pfkfb3 | 0.199 |
| fgfrl1 | 0.199 |
| arnt | 0.199 |
| gab1 | 0.199 |
| mapk7 | 0.199 |
| prox1 | 0.199 |
| crtc2 | 0.198 |
| klf2 | 0.198 |
| 2310008h04rik | 0.198 |
| ntn4 | 0.198 |
| psg23 | 0.197 |
| cpeb2 | 0.197 |
| bhlhb2 | 0.197 |
| ankrd37 | 0.197 |
| iba1 | 0.197 |
| zfyve9 | 0.197 |
| gipc1 | 0.197 |
| sphk1 | 0.196 |
| scube2 | 0.196 |
| ltb4r2 | 0.195 |
| hpse | 0.195 |
| efna1 | 0.195 |
| spry2 | 0.194 |
| anxa3 | 0.194 |
| adora2b | 0.194 |
| clec11a | 0.194 |
| stc1 | 0.194 |
| akap12 | 0.194 |
| dusp1 | 0.194 |
| med28 | 0.193 |
| tspan12 | 0.192 |
| zfand6 | 0.192 |
| lpar2 | 0.192 |
| col4a3bp | 0.192 |
| baiap3 | 0.191 |
| pik3cb | 0.191 |
| angptl4 | 0.191 |
| fzd7 | 0.191 |
| tnfsf12 | 0.191 |
| hey1 | 0.19 |
| sema6b | 0.19 |
| frs2 | 0.19 |
| egfl8 | 0.19 |
| cirbp | 0.189 |
| tsve | 0.189 |
| tsv | 0.189 |
| cdh13 | 0.189 |
| vav2 | 0.189 |
| 1190002h23rik | 0.188 |
| clic4 | 0.188 |
| tktl1 | 0.188 |
| ceacam1 | 0.188 |
| stab1 | 0.188 |
| hey2 | 0.187 |
| plxnb3 | 0.187 |
| prl2c4 | 0.187 |
| grb10 | 0.187 |
| col8a1 | 0.187 |
| egr3 | 0.187 |
| arhgap1 | 0.187 |
| ptn | 0.186 |
| epha2 | 0.186 |
| polr2g | 0.186 |
| sema5b | 0.185 |
| ets1 | 0.185 |
| pf4 | 0.185 |
| etv2 | 0.184 |
| dok2 | 0.184 |
| cyp2c44 | 0.184 |
| v1rb2 | 0.184 |
| cd34 | 0.184 |
| plxnc1 | 0.184 |
| loc546644 | 0.184 |
| 4933406e20rik | 0.184 |
| anks1 | 0.183 |
| malt1 | 0.183 |
| id3 | 0.183 |
| itgb5 | 0.183 |
| 3632451o06rik | 0.182 |
| klhl30 | 0.182 |
| ppp1r16b | 0.182 |
| tmem204 | 0.182 |
| pdgfb | 0.182 |
| cul2 | 0.181 |
| gpr180 | 0.181 |
| dgka | 0.181 |
| akirin2 | 0.18 |
| rbm10 | 0.18 |
| plxnb2 | 0.18 |
| ctgf | 0.18 |
| bcl10 | 0.18 |
| rps6kb1 | 0.18 |
| pbrgcsf1 | 0.179 |
| sh2b3 | 0.179 |
| nck1 | 0.179 |
| s1pr2 | 0.178 |
| fert2 | 0.178 |
| tel13q | 0.177 |
| axl | 0.177 |
| sulf2 | 0.176 |
| shc4 | 0.176 |
| foxc2 | 0.176 |
| thsd1 | 0.176 |
| thbs1 | 0.176 |
| sema4b | 0.176 |
| ddah1 | 0.176 |
| ldha | 0.175 |
| zfp36 | 0.175 |
| pdk1 | 0.175 |
| ero1l | 0.175 |
| hbegf | 0.175 |
| d0h4s114 | 0.174 |
| mfap5 | 0.174 |
| rasa1 | 0.174 |
| arhgef17 | 0.174 |
| rpl32 | 0.173 |
| ptpro | 0.173 |
| ard1 | 0.173 |
| mapk11 | 0.172 |
| fgf16 | 0.172 |
| ilf3 | 0.172 |
| bhlhb3 | 0.172 |
| scube1 | 0.172 |
| zeb1 | 0.171 |
| rhbdd2 | 0.171 |
| gtf2i | 0.171 |
| syngap1 | 0.171 |
| s1pr1 | 0.17 |
| sars | 0.17 |
| ilk | 0.17 |
| gas6 | 0.17 |
| ing4 | 0.17 |
| gmfg | 0.17 |
| heg1 | 0.17 |
| pttg1 | 0.17 |
| agtrap | 0.169 |
| 0610011l14rik | 0.169 |
| 6330500d04rik | 0.168 |
| apln | 0.168 |
| hk2 | 0.168 |
| gpr125 | 0.168 |
| rhoc | 0.168 |
| pdpn | 0.168 |
| klk1b4 | 0.168 |
| khdrbs3 | 0.168 |
| ppard | 0.168 |
| sox18 | 0.167 |
| sema4c | 0.167 |
| kdr | 0.167 |
| tnip2 | 0.167 |
| fgf18 | 0.167 |
| sema7a | 0.167 |
| snai1 | 0.167 |
| serpinb13 | 0.167 |
| nrn1 | 0.167 |
| aoc3-rs | 0.167 |
| zkscan3 | 0.167 |
| pdgfa | 0.166 |
| nkap | 0.166 |
| s100a13 | 0.166 |
| gipc2 | 0.165 |
| 1110012m11rik | 0.165 |
| aplnr | 0.165 |
| siah2 | 0.165 |
| ptprj | 0.165 |
| rit1 | 0.165 |
| 2610207i05rik | 0.165 |
| eif2c2 | 0.165 |
| tnfrsf12a | 0.165 |
| ptger1 | 0.164 |
| lax1 | 0.164 |
| arhgef15 | 0.164 |
| gpc1 | 0.164 |
| sema4g | 0.164 |
| pdgfit2 | 0.164 |
| pdgfit1 | 0.164 |
| col15a1 | 0.163 |
| rras | 0.163 |
| cxcl14 | 0.163 |
| wnt2 | 0.163 |
| sstr1 | 0.163 |
| tns3 | 0.163 |
| tmsb10 | 0.163 |
| bmp10 | 0.163 |
| lims1 | 0.163 |
| trim25 | 0.163 |
| col8a2 | 0.162 |
| sema6a | 0.162 |
| csf2rb2 | 0.162 |
| nrp | 0.162 |
| gprc5b | 0.162 |
| arhgap24 | 0.162 |
| vhl | 0.162 |
| areg | 0.162 |
| ptger4 | 0.162 |
| csf1r | 0.162 |
| tceb2 | 0.162 |
| plcb3 | 0.162 |
| adamts7 | 0.162 |
| pfkfb2 | 0.162 |
| nr4a1 | 0.161 |
| rap1b | 0.161 |
| cops5 | 0.161 |
| grb14 | 0.161 |
| arnt2 | 0.161 |
| iqgap1 | 0.161 |
| lrrc4 | 0.161 |
| psma7 | 0.161 |
| ccdc72 | 0.16 |
| igfbp7 | 0.16 |
| tfpi2 | 0.16 |
| smarca1 | 0.16 |
| gab2 | 0.16 |
| atp6ap2 | 0.16 |
| junb | 0.16 |
| dcun1d1 | 0.16 |
| sgpp1 | 0.16 |
| zmiz1 | 0.16 |
| grb7 | 0.16 |
| abi3bp | 0.159 |
| fzd2 | 0.159 |
| ccm2 | 0.159 |
| mvwf | 0.159 |
| sla2 | 0.159 |
| car12 | 0.159 |
| chac1 | 0.159 |
| wfdc1 | 0.159 |
| dok3 | 0.158 |
| cd2ap | 0.158 |
| pcdhb1 | 0.158 |
| pcdhb4 | 0.158 |
| d9mit115 | 0.158 |
| d9mit76 | 0.158 |
| mmp11 | 0.158 |
| il34 | 0.158 |
| eltd1 | 0.158 |
| fgf22 | 0.158 |
| raet1d | 0.157 |
| serpinb5 | 0.157 |
| noc3l | 0.157 |
| sema6c | 0.157 |
| gabpb1 | 0.156 |
| erap1 | 0.156 |
| tceb3 | 0.156 |
| st3gal5 | 0.156 |
| shcbp1 | 0.156 |
| foxm1 | 0.155 |
| bmper | 0.155 |
| cxcl1 | 0.155 |
| ddah2 | 0.155 |
| crim1 | 0.155 |
| depdc6 | 0.155 |
| nfam1 | 0.155 |
| fgf5 | 0.155 |
| tceb1 | 0.154 |
| gucy1b3 | 0.154 |
| prkab1 | 0.153 |
| fgf7 | 0.153 |
| ptpn2 | 0.153 |
| lcp1 | 0.153 |
| afap1l2 | 0.152 |
| pik3ca | 0.152 |
| hdac5 | 0.152 |
| cnn1 | 0.152 |
| dusp16 | 0.152 |
| dok4 | 0.152 |
| ppp1r14b | 0.152 |
| lpar4 | 0.152 |
| fstl1 | 0.152 |
| ctnnbip1 | 0.152 |
| zfp444 | 0.152 |
| ccdc88c | 0.152 |
| sulf1 | 0.152 |
| hexim1 | 0.151 |
| spred3 | 0.151 |
| hnrnpl | 0.151 |
| nov | 0.151 |
| spry1 | 0.151 |
| atf4 | 0.151 |
| ptger2 | 0.151 |
| fndc1 | 0.151 |
| nln | 0.151 |
| sstr2 | 0.151 |
| ereg | 0.151 |
| akt3 | 0.15 |
| shc3 | 0.15 |
| dnm2 | 0.15 |
| ppapdc3 | 0.15 |
| psg29 | 0.15 |
| psg22 | 0.15 |
| map2k2 | 0.15 |
| tmsb4x | 0.15 |
| gna11 | 0.15 |
| zbtb7c | 0.15 |
| 2310056p07rik | 0.15 |
| jag2 | 0.15 |
| cul5 | 0.149 |
| epha1 | 0.149 |
| frs3 | 0.149 |
| mapkap1 | 0.149 |
| slc2a1 | 0.149 |
| dapp1 | 0.149 |
| bsg | 0.148 |
| gpr55 | 0.148 |
| sp4 | 0.148 |
| mpzl1 | 0.148 |
| tom1l1 | 0.148 |
| cldn5 | 0.148 |
| errfi1 | 0.148 |
| pdcd10 | 0.148 |
| epor | 0.148 |
| slc2a3 | 0.148 |
| cx3cl1 | 0.148 |
| tbc1d10c | 0.148 |
| gpr6 | 0.148 |
| jam3 | 0.148 |
| mirn21 | 0.147 |
| hipk2 | 0.147 |
| lpar1 | 0.147 |
| zc3h15 | 0.147 |
| 4921505c17rik | 0.147 |
| efemp1 | 0.147 |
| shoc2 | 0.147 |
| ddost | 0.147 |
| reck | 0.147 |
| rwdd3 | 0.147 |
| stam | 0.147 |
| ptafr | 0.147 |
| eif4e | 0.146 |
| s1pr5 | 0.146 |
| mmrn2 | 0.146 |
| sema5a | 0.146 |
| tgfbr3 | 0.146 |
| v2r8 | 0.146 |
| adora3 | 0.146 |
| pthlh | 0.146 |
| fgfr1 | 0.146 |
| klf5 | 0.146 |
| ddt | 0.146 |
| chn2 | 0.145 |
| mirn145 | 0.145 |
| mia1 | 0.145 |
| csk | 0.145 |
| tnfaip8 | 0.145 |
| alox12 | 0.145 |
| syx1 | 0.145 |
| syx2 | 0.145 |
| tbxa2r | 0.145 |
| zhx2 | 0.145 |
| itgb8 | 0.145 |
| osbpl9 | 0.145 |
| mmp10 | 0.144 |
| gnb2l1 | 0.144 |
| stap2 | 0.144 |
| fbln5 | 0.144 |
| ndph | 0.144 |
| rnd1 | 0.144 |
| dusp6 | 0.144 |
| ptk2b | 0.144 |
| anpep | 0.144 |
| asb15 | 0.143 |
| pdpk1 | 0.143 |
| jam2 | 0.143 |
| apol7c | 0.143 |
| fgf9 | 0.143 |
| oraov1 | 0.143 |
| 4932417h02rik | 0.143 |
| nox4 | 0.143 |
| pdgfrb | 0.143 |
| cmtm8 | 0.143 |
| hnrnpk | 0.142 |
| mmp7 | 0.142 |
| dnajb9 | 0.142 |
| 2310016c08rik | 0.142 |
| siah1a | 0.142 |
| oit1 | 0.142 |
| pak3 | 0.142 |
| iapls3-5 | 0.142 |
| fgf1 | 0.142 |
| map2k1ip1 | 0.141 |
| hsh2d | 0.141 |
| plekhm3 | 0.141 |
| mark2 | 0.141 |
| sparc | 0.141 |
| itga9 | 0.141 |
| centg3 | 0.141 |
| prom1 | 0.141 |
| etv4 | 0.141 |
| sh2d3c | 0.141 |
| csf3 | 0.141 |
| vmn2r122 | 0.141 |
| tg(krt14-cre)1efu | 0.141 |
| pik3r5 | 0.14 |
| fhl2 | 0.14 |
| tnfsf15 | 0.14 |
| tal1 | 0.14 |
| pld2 | 0.14 |
| ephb2 | 0.14 |
| tbc1d8 | 0.14 |
| sh2d4a | 0.14 |
| zfp641 | 0.14 |
| gpbar1 | 0.14 |
| dicer1 | 0.14 |
| prkd3 | 0.14 |
| jag1 | 0.14 |
| synpo | 0.14 |
| mmp14 | 0.14 |
| d18mit18 | 0.14 |
| d17mit51 | 0.14 |
| rps6ka5 | 0.139 |
| olr1 | 0.139 |
| fgf10 | 0.139 |
| enpp2 | 0.139 |
| cxcl2 | 0.139 |
| rasa3 | 0.139 |
| creg1 | 0.139 |
| ets2 | 0.139 |
| ror2 | 0.139 |
| ptpn14 | 0.138 |
| pik3c3 | 0.138 |
| stap1 | 0.138 |
| fgfr4 | 0.138 |
| klhdc2 | 0.138 |
| angptl7 | 0.138 |
| atoh8 | 0.138 |
| gucy1a3 | 0.138 |
| cd300lg | 0.138 |
| pdlim7 | 0.138 |
| farp2 | 0.138 |
| gbp2 | 0.138 |
| itgb1bp1 | 0.138 |
| bnip3 | 0.138 |
| ephb1 | 0.137 |
| rapgef3 | 0.137 |
| rbm39 | 0.137 |
| nphs1 | 0.137 |
| hand1 | 0.137 |
| cblc | 0.137 |
| zfp383 | 0.137 |
| pear1 | 0.137 |
| edf1 | 0.137 |
| gata2 | 0.137 |
| ift74 | 0.137 |
| pthr1 | 0.137 |
| tgfbr1 | 0.137 |
| dok1 | 0.137 |
| prkaa2 | 0.137 |
| ptprm | 0.137 |
| nutf2 | 0.137 |
| gpr126 | 0.136 |
| bc048355 | 0.136 |
| gucy2d | 0.136 |
| dusp14 | 0.136 |
| ptger3 | 0.136 |
| 1200015f23rik | 0.136 |
| epgn | 0.136 |
| esrra | 0.136 |
| plcg1 | 0.136 |
| tgfbrap1 | 0.136 |
| ddit4l | 0.136 |
| pak1 | 0.135 |
| csrp2 | 0.135 |
| dpysl5 | 0.135 |
| tagln | 0.135 |
| hspg2 | 0.135 |
| emr1 | 0.135 |
| thbd | 0.135 |
| ppp1r13b | 0.135 |
| ndst1 | 0.135 |
| tmbim1 | 0.135 |
| mirn143 | 0.134 |
| rasd1 | 0.134 |
| adam15 | 0.134 |
| yars | 0.134 |
| grhl3 | 0.134 |
| id2 | 0.134 |
| epo | 0.134 |
| fgf13 | 0.134 |
| map3k3 | 0.134 |
| f3 | 0.134 |
| dnajc3 | 0.134 |
| prl2c2 | 0.134 |
| lrp6 | 0.134 |
| mdk | 0.134 |
| pik3c2b | 0.133 |
| nox1 | 0.133 |
| adm2 | 0.133 |
| tnk1 | 0.133 |
| zfp418 | 0.133 |
| atg9b | 0.133 |
| lpxn | 0.133 |
| scg2 | 0.133 |
| itgb4 | 0.133 |
| il3ra | 0.133 |
| eif4ebp1 | 0.133 |
| nr2e1 | 0.133 |
| tabw2 | 0.133 |
| cirbp-rs2 | 0.133 |
| sart1 | 0.132 |
| fgf2 | 0.132 |
| cbll1 | 0.132 |
| trip6 | 0.132 |
| pdlim2 | 0.132 |
| fosr | 0.132 |
| has3 | 0.132 |
| shisa2 | 0.132 |
| csf1 | 0.131 |
| nck2 | 0.131 |
| cbl | 0.131 |
| ngef | 0.131 |
| dusp5 | 0.131 |
| wwtr1 | 0.131 |
| gata6 | 0.131 |
| trib3 | 0.131 |
| ierepo2 | 0.131 |
| col10a1 | 0.131 |
| arhgef4 | 0.131 |
| pold4 | 0.131 |
| dkk4 | 0.131 |
| plce1 | 0.131 |
| hr | 0.131 |
| asb4 | 0.131 |
| paep | 0.131 |
| fasn | 0.131 |
| arhgef7 | 0.131 |
| cd40 | 0.131 |
| pak4 | 0.13 |
| ubash3b | 0.13 |
| tcfap2a | 0.13 |
| map4k1 | 0.13 |
| cxcl5 | 0.13 |
| s1pr4 | 0.13 |
| skap1 | 0.13 |
| stmn1 | 0.13 |
| ksr2 | 0.13 |
| rgs4 | 0.13 |
| mst1r | 0.13 |
| pdcd2l | 0.13 |
| tnc | 0.13 |
| tcf7 | 0.13 |
| magea2 | 0.13 |
| pik3r2 | 0.13 |
| wars | 0.13 |
| parva | 0.13 |
| adora2a | 0.13 |
| il17re | 0.129 |
| vldlr | 0.129 |
| wnt11 | 0.129 |
| wtap | 0.129 |
| wnt7b | 0.129 |
| metap2 | 0.129 |
| prkaa1 | 0.129 |
| heyl | 0.129 |
| ndrg1 | 0.129 |
| ube2s | 0.129 |
| fosb | 0.129 |
| postn | 0.129 |
| d19mit53 | 0.129 |
| frap1 | 0.129 |
| itga5 | 0.129 |
| ey2 | 0.129 |
| gdf9 | 0.129 |
| stam2 | 0.129 |
| tfg | 0.129 |
| appl1 | 0.129 |
| akirin1 | 0.128 |
| nfatc1 | 0.128 |
| phip | 0.128 |
| pgk1 | 0.128 |
| gipc3 | 0.128 |
| khdrbs2 | 0.128 |
| fbln1 | 0.128 |
| itgav | 0.128 |
| tnik | 0.128 |
| rock2 | 0.128 |
| cpeb1 | 0.128 |
| sla | 0.128 |
| notch3 | 0.128 |
| sos1 | 0.128 |
| foxc1 | 0.128 |
| slc7a5 | 0.128 |
| foxd1 | 0.128 |
| rock1 | 0.128 |
| mif | 0.128 |
| e030049g20rik | 0.128 |
| crmp1 | 0.128 |
| fer | 0.127 |
| stx19 | 0.127 |
| wdr26 | 0.127 |
| il6ra | 0.127 |
| phlppl | 0.127 |
| tg(k6odctr)55tgo | 0.127 |
| ythdc1 | 0.127 |
| grr | 0.127 |
| rit2 | 0.127 |
| ihh | 0.127 |
| ckap4 | 0.127 |
| gigyf1 | 0.127 |
| fzd5 | 0.127 |
| pik3ip1 | 0.127 |
| slmap | 0.127 |
| bey | 0.127 |
| gper | 0.127 |
| txnip | 0.127 |
| c330002i19rik | 0.127 |
| dusp4 | 0.127 |
| cxcl16 | 0.127 |
| stab2 | 0.127 |
| map2k1 | 0.127 |
| stk24 | 0.127 |
| dock7 | 0.127 |
| fzd1 | 0.127 |
| rps6kb2 | 0.126 |
| zfp446 | 0.126 |
| arhgap5 | 0.126 |
| shc1 | 0.126 |
| dkk3 | 0.126 |
| card14 | 0.126 |
| angptl3 | 0.126 |
| scpep1 | 0.126 |
| kifc4b | 0.126 |
| 2810004i08rik | 0.126 |
| trib1 | 0.126 |
| npn2 | 0.126 |
| map2k5 | 0.126 |
| npas1 | 0.126 |
| sh3bp2 | 0.126 |
| tgfb1i1 | 0.126 |
| osm | 0.125 |
| hhex | 0.125 |
| tsp1 | 0.125 |
| ruvbl1 | 0.125 |
| dll1 | 0.125 |
| hdac4 | 0.125 |
| vegfa | 0.125 |
| map4k4 | 0.125 |
| sorbs3 | 0.125 |
| ednra | 0.125 |
| os9 | 0.125 |
| map2k3 | 0.125 |
| pkig | 0.125 |
| olfr17 | 0.125 |
| prl2c5 | 0.125 |
| gng4 | 0.125 |
| gzf1 | 0.125 |
| mapk15 | 0.125 |
| smyd2 | 0.125 |
| rnasen | 0.125 |
| lime1 | 0.125 |
| vip | 0.125 |
| sh3kbp1 | 0.125 |
| 9830130m13rik | 0.124 |
| akt1s1 | 0.124 |
| sh2b2 | 0.124 |
| rtkn | 0.124 |
| ryk-rs1 | 0.124 |
| fgfr1op | 0.124 |
| ogfr | 0.124 |
| mta1 | 0.124 |
| sdc4 | 0.124 |
| mcam | 0.124 |
| zfp322a | 0.124 |
| slfn1 | 0.124 |
| ly6g6f | 0.124 |
| ptgir | 0.124 |
| aebp1 | 0.124 |
| cxcr7 | 0.124 |
| zfp74 | 0.124 |
| sorbs2 | 0.124 |
| jund | 0.124 |
| gcm1 | 0.124 |
| nudcd1 | 0.124 |
| ciapin1 | 0.124 |
| cdkn1c | 0.124 |
| snf1lk | 0.124 |
| kcmf1 | 0.124 |
| tnxb | 0.124 |
| podxl2 | 0.124 |
| ebag9 | 0.123 |
| lrrfip1 | 0.123 |
| ssh1 | 0.123 |
| lmnb1 | 0.123 |
| nuak1 | 0.123 |
| tnfaip2 | 0.123 |
| msrvh1 | 0.123 |
| pik3r3 | 0.123 |
| pitpnm3 | 0.123 |
| bcas3 | 0.123 |
| ctsl | 0.123 |
| mpl | 0.123 |
| gbl | 0.123 |
| prl3d1 | 0.123 |
| elavl3 | 0.123 |
| akt2 | 0.123 |
| yap1 | 0.123 |
| scarf1 | 0.123 |
| raet1a | 0.123 |
| gpr132 | 0.123 |
| ptgis | 0.123 |
| olfr1310 | 0.123 |
| nsmaf | 0.123 |
| birc5 | 0.123 |
| cnih2 | 0.122 |
| taok3 | 0.122 |
| phlpp | 0.122 |
| uhmk1 | 0.122 |
| spata5 | 0.122 |
| usp6nl | 0.122 |
| cthrc1 | 0.122 |
| zcchc11 | 0.122 |
| grap | 0.122 |
| cc2d1a | 0.122 |
| pik3c2g | 0.122 |
| dnaja3 | 0.122 |
| rgs13 | 0.122 |
| zfp692 | 0.122 |
| fosl1 | 0.122 |
| ldhal6b | 0.122 |
| snx26 | 0.122 |
| mmp21 | 0.122 |
| dgkz | 0.121 |
| mirn16-2 | 0.121 |
| mmp19 | 0.121 |
| meox2 | 0.121 |
| phlda1 | 0.121 |
| ybx1 | 0.121 |
| tjp2 | 0.121 |
| rbmx | 0.121 |
| hgf | 0.121 |
| atf3 | 0.121 |
| tg(hgf)1paus | 0.121 |
| araf | 0.121 |
| nfil3 | 0.121 |
| magi1 | 0.121 |
| adm | 0.121 |
| sdc2 | 0.121 |
| cd97 | 0.121 |
| vav3 | 0.121 |
| fkbp15 | 0.121 |
| 4732474o15rik | 0.121 |
| nfatc2 | 0.12 |
| hemgn | 0.12 |
| loc641201 | 0.12 |
| sat2 | 0.12 |
| csf2rb | 0.12 |
| limk1 | 0.12 |
| gab3 | 0.12 |
| acta2 | 0.12 |
| trim33 | 0.12 |
| hgs | 0.12 |
| thsd3 | 0.12 |
| pecam1 | 0.12 |
| sox7 | 0.12 |
| inhba | 0.12 |
| clnk | 0.12 |
| aif1 | 0.12 |
| dact1 | 0.12 |
| fosl2 | 0.12 |
| smad6 | 0.12 |
| wc | 0.12 |
| cd55 | 0.12 |
| rrm2 | 0.12 |
| zc3h12b | 0.12 |
| inppl1 | 0.12 |
| lin28 | 0.12 |
| nosip | 0.12 |
| fbln2 | 0.119 |
| trip11 | 0.119 |
| dok5 | 0.119 |
| tec | 0.119 |
| gdf10 | 0.119 |
| mirn1-2 | 0.119 |
| grem1 | 0.119 |
