# Supplementary material for: GIT2 Acts as a Potential Keystone Protein in Functional Hypothalamic Networks Associated with Age-Related Phenotypic Changes in Rats
Source: PLoS One. 2012 May 14;7(5):e36975. doi: 10.1371/journal.pone.0036975 (PMC3351446; doi:10.1371/journal.pone.0036975)
Supplement: Table S18 — GeneIndexer latent semantic indexing (LSI) of significantly-regulated ‘p53 signaling’ KEGG pathway. Using the KEGG signaling pathway ‘p53 signaling’ as an input term, a list of the top 1000 implicitly-correlated (LSI correlation score >0.1) was generated using a full genome background list. (DOC) [file pone.0036975.s022.doc]

**Table S18. GeneIndexer latent semantic indexing (LSI) of significantly-regulated ‘p53 signaling’ KEGG pathway.** Using the KEGG signaling pathway ‘p53 signaling’ as an input term, a list of the top 1000 implicitly-correlated (LSI correlation score >0.1) was generated using a full genome background list.

| ***p53 signaling*** |  |
| --- | --- |
|  |  |
| **Protein Symbol** | **LSI correlation score** |
| zfyve21 | 0.88 |
| zfp420 | 0.872 |
| glipr1l1 | 0.866 |
| tprkb | 0.856 |
| af366264 | 0.849 |
| fbxo42 | 0.848 |
| mirn34b | 0.827 |
| mirn34c | 0.827 |
| rps27l | 0.815 |
| cgref1 | 0.795 |
| ttc5 | 0.792 |
| hipk4 | 0.791 |
| motp1 | 0.777 |
| 1200009f10rik | 0.774 |
| tg(trp53r172l)4491jmr | 0.738 |
| mdm4-ps | 0.727 |
| zbtb2 | 0.725 |
| 5730403b10rik | 0.721 |
| pdrg1 | 0.721 |
| isg20l1 | 0.717 |
| triap1 | 0.712 |
| slc37a3 | 0.709 |
| rnf144b | 0.703 |
| mrpl11 | 0.7 |
| tbrg1 | 0.694 |
| d1mit531 | 0.694 |
| d11mit332 | 0.694 |
| ottmusg00000000421 | 0.69 |
| strm | 0.684 |
| gtse1 | 0.683 |
| cdkn2aip | 0.682 |
| ankrd11 | 0.68 |
| jmy | 0.674 |
| mp53d3 | 0.664 |
| mp53d2 | 0.664 |
| mp53d1 | 0.664 |
| sesn1 | 0.659 |
| 4632434i11rik | 0.657 |
| 1200002n14rik | 0.656 |
| rpl26 | 0.654 |
| noc2l | 0.652 |
| d3mit139 | 0.65 |
| carf | 0.649 |
| cgrrf1 | 0.647 |
| mirn34a | 0.64 |
| znhit1 | 0.639 |
| slc11a1-rs1 | 0.637 |
| tmem55b | 0.637 |
| fbxo45 | 0.635 |
| ei24 | 0.633 |
| g3bp2 | 0.62 |
| mtbp | 0.618 |
| rbm38 | 0.614 |
| aa408296 | 0.611 |
| ahcyl2 | 0.605 |
| dbpht1 | 0.598 |
| trp53i11 | 0.598 |
| loc100034726 | 0.596 |
| mrpl41 | 0.596 |
| tmem77 | 0.595 |
| ppp1r13b | 0.595 |
| glipr1l2 | 0.592 |
| zbtb4 | 0.588 |
| sertad3 | 0.587 |
| trp53rk | 0.583 |
| magea4 | 0.575 |
| eef1e1 | 0.569 |
| shisa5 | 0.565 |
| lypd1 | 0.561 |
| siah1b | 0.56 |
| rps19bp1 | 0.557 |
| zfp346 | 0.557 |
| 9630033f20rik | 0.556 |
| trp53-ps | 0.555 |
| commd7 | 0.546 |
| perp | 0.543 |
| d11mit90 | 0.543 |
| lrdd | 0.543 |
| dbpht2 | 0.542 |
| morc3 | 0.542 |
| sertad2 | 0.54 |
| rpl11 | 0.54 |
| rnf34 | 0.535 |
| msl2l1 | 0.526 |
| banp | 0.525 |
| pxdn | 0.522 |
| trp53inp1 | 0.52 |
| zmiz2 | 0.517 |
| fkbp3 | 0.512 |
| sesn2 | 0.512 |
| cxxc5 | 0.512 |
| zmat3 | 0.502 |
| wfdc5 | 0.5 |
| zfp385a | 0.499 |
| glipr1 | 0.498 |
| parc | 0.498 |
| rpl23 | 0.495 |
| tsp7 | 0.494 |
| tsp4 | 0.494 |
| tsp5 | 0.494 |
| tsp6 | 0.494 |
| trp53bp2 | 0.494 |
| gramd4 | 0.494 |
| rffl | 0.493 |
| bc063263 | 0.491 |
| unc5d | 0.49 |
| ing5 | 0.489 |
| wdr79 | 0.486 |
| cables2 | 0.48 |
| rprm | 0.48 |
| kras1-ps | 0.479 |
| d11mit165 | 0.478 |
| aifm2 | 0.478 |
| ccdc85b | 0.478 |
| pcbp4 | 0.475 |
| tada3l | 0.469 |
| cul7 | 0.464 |
| ing2 | 0.463 |
| rchy1 | 0.46 |
| prodh2 | 0.458 |
| zbtb5 | 0.457 |
| tmem16j | 0.456 |
| ccar1 | 0.456 |
| rai12 | 0.452 |
| letmd1 | 0.445 |
| loh2 | 0.444 |
| pgct1 | 0.442 |
| taf9b | 0.441 |
| tg(trp53r172h)8512jmr | 0.441 |
| ethe1 | 0.437 |
| ccng1 | 0.437 |
| ing3 | 0.435 |
| ep400 | 0.431 |
| sertad1 | 0.429 |
| rbbp6 | 0.429 |
| d19mit123 | 0.426 |
| hecw1 | 0.426 |
| lcmt2 | 0.425 |
| rrm2b | 0.425 |
| ing1 | 0.42 |
| gpr87 | 0.42 |
| rpl13a | 0.419 |
| tnfrsf23 | 0.415 |
| ppm1d | 0.414 |
| smyd2 | 0.414 |
| rfwd2 | 0.412 |
| vrk2 | 0.412 |
| plrg1 | 0.412 |
| huwe1 | 0.412 |
| ifi205 | 0.411 |
| blcap | 0.411 |
| mdm4 | 0.41 |
| topors | 0.41 |
| ppp1r13l | 0.409 |
| usp7 | 0.407 |
| supt7l | 0.405 |
| cabc1 | 0.404 |
| ubqln4 | 0.398 |
| vrk1 | 0.397 |
| rasal2 | 0.396 |
| mkrn1 | 0.396 |
| rapop1 | 0.395 |
| tsp3 | 0.394 |
| rps7 | 0.394 |
| zmiz1 | 0.391 |
| rreb1 | 0.388 |
| usp5 | 0.388 |
| wn | 0.386 |
| ppfibp1 | 0.386 |
| cyfip2 | 0.382 |
| shkbp1 | 0.382 |
| psmd10 | 0.379 |
| dmtf1 | 0.378 |
| psmd13 | 0.372 |
| phlda3 | 0.369 |
| vps53 | 0.368 |
| cdk5rap3 | 0.366 |
| pja1 | 0.366 |
| hipk1 | 0.365 |
| usp2 | 0.365 |
| psrc1 | 0.365 |
| impad1 | 0.363 |
| tg(nes-rtta)306rvs | 0.363 |
| steap3 | 0.363 |
| bccip | 0.363 |
| ing4 | 0.362 |
| d11mit285 | 0.361 |
| e4f1 | 0.36 |
| sephs1 | 0.359 |
| osgin1 | 0.359 |
| tada2l | 0.358 |
| taf9 | 0.358 |
| chd8 | 0.357 |
| hipk2 | 0.357 |
| dtl | 0.356 |
| ube2l3-ps1 | 0.354 |
| d1mit19 | 0.353 |
| gadd45gip1 | 0.351 |
| maged2 | 0.351 |
| ccng2 | 0.35 |
| fbxw8 | 0.348 |
| gnl3 | 0.348 |
| efhd1 | 0.347 |
| rpl5 | 0.345 |
| 6030408c04rik | 0.345 |
| tprg | 0.344 |
| d16mit87 | 0.344 |
| 2610301g19rik | 0.344 |
| taf1c | 0.34 |
| plac8 | 0.339 |
| cradd | 0.339 |
| usp28 | 0.338 |
| ciao1 | 0.338 |
| gas2 | 0.338 |
| plk4 | 0.337 |
| pfdn5 | 0.337 |
| gps2 | 0.337 |
| mirn101a | 0.335 |
| ddb2 | 0.332 |
| lats2 | 0.332 |
| cops3 | 0.332 |
| bai1 | 0.33 |
| mcts1 | 0.33 |
| d2mit147 | 0.33 |
| suds3 | 0.33 |
| taf1b | 0.329 |
| ifi202b | 0.328 |
| ube4a | 0.328 |
| arid3a | 0.327 |
| cks2 | 0.327 |
| anxa10 | 0.327 |
| bc060632 | 0.327 |
| loh1 | 0.326 |
| ankrd42 | 0.326 |
| zfp148 | 0.326 |
| ube2d3 | 0.326 |
| yeats4 | 0.326 |
| mrpl13 | 0.323 |
| fbxo11 | 0.323 |
| siva1 | 0.322 |
| taf3 | 0.321 |
| rps20 | 0.321 |
| ppp1r10 | 0.32 |
| mlf1 | 0.319 |
| cdca4 | 0.319 |
| rims3 | 0.318 |
| gadd45a | 0.318 |
| sall2 | 0.318 |
| mastr | 0.317 |
| tfpt | 0.317 |
| hecw2 | 0.317 |
| prkcdbp | 0.316 |
| apitd1 | 0.315 |
| tceal1 | 0.314 |
| siah2 | 0.314 |
| d11mit22 | 0.313 |
| magi3 | 0.312 |
| rbmx | 0.312 |
| dfna5h | 0.311 |
| hepacam | 0.311 |
| dph1 | 0.31 |
| wdr12 | 0.308 |
| rad23a | 0.308 |
| dyrk2 | 0.308 |
| psme3 | 0.306 |
| bop1 | 0.306 |
| setd2 | 0.305 |
| rybp | 0.305 |
| lrrc15 | 0.304 |
| gadd45g | 0.304 |
| pes1 | 0.304 |
| jtv1 | 0.302 |
| plk2 | 0.301 |
| siah1a | 0.301 |
| trp53inp2 | 0.301 |
| rpl32 | 0.3 |
| rbm10 | 0.3 |
| taf1a | 0.3 |
| gprc5a | 0.299 |
| ifi204 | 0.298 |
| phc2 | 0.298 |
| sirt7 | 0.297 |
| ppp2r5e | 0.297 |
| trrap | 0.296 |
| nol1 | 0.296 |
| mrpl23 | 0.296 |
| g3bp1 | 0.295 |
| bre | 0.295 |
| aatf | 0.294 |
| olig3 | 0.294 |
| camk2n2 | 0.294 |
| stra13 | 0.294 |
| tspyl2 | 0.293 |
| cnksr3 | 0.293 |
| ldoc1 | 0.29 |
| foxo6 | 0.29 |
| hic1 | 0.289 |
| myd116 | 0.288 |
| smpd4 | 0.287 |
| bat3 | 0.286 |
| traf4 | 0.286 |
| cables1 | 0.285 |
| d4wsu114e | 0.284 |
| c330027c09rik | 0.284 |
| rps6ka6 | 0.284 |
| nat13 | 0.283 |
| htatip2 | 0.283 |
| sfn | 0.282 |
| cabin1 | 0.282 |
| htatip | 0.282 |
| ube4b | 0.282 |
| ell | 0.281 |
| gas1 | 0.281 |
| cdkn3 | 0.28 |
| dusp5 | 0.279 |
| nfyc | 0.279 |
| d12mit263 | 0.278 |
| btg3 | 0.277 |
| supt3h | 0.276 |
| plk3 | 0.275 |
| birc6 | 0.274 |
| setd7 | 0.274 |
| fkbpl | 0.274 |
| zbtb17 | 0.273 |
| pmaip1 | 0.273 |
| ube2d2 | 0.273 |
| cbx7 | 0.273 |
| crnn | 0.272 |
| dhcr24 | 0.272 |
| prkrir | 0.271 |
| antxr1 | 0.271 |
| dtymk | 0.27 |
| arl8b | 0.27 |
| gadd45b | 0.27 |
| tg(wnt1)1hev | 0.269 |
| plekho1 | 0.269 |
| pmm1 | 0.267 |
| eid1 | 0.267 |
| wwp1 | 0.267 |
| ddit4l | 0.267 |
| 2610207i05rik | 0.267 |
| ppp5c | 0.266 |
| strap | 0.266 |
| bbc3 | 0.265 |
| hbxip | 0.265 |
| ngdn | 0.265 |
| cse1l | 0.265 |
| cebpz | 0.264 |
| trp53bp1 | 0.264 |
| gtf2e1 | 0.264 |
| nol3 | 0.264 |
| gps1 | 0.263 |
| mll3 | 0.263 |
| csnk1a1 | 0.262 |
| ras | 0.262 |
| gmcl1 | 0.262 |
| elf4 | 0.261 |
| reep5 | 0.26 |
| myst3 | 0.26 |
| dusp2 | 0.259 |
| senp8 | 0.258 |
| eg628324 | 0.258 |
| wwox | 0.257 |
| taf5 | 0.257 |
| trp53i13 | 0.256 |
| ube3a | 0.256 |
| ier3 | 0.256 |
| senp3 | 0.256 |
| cacybp | 0.255 |
| brf1 | 0.255 |
| spdya | 0.254 |
| ppp2r5a | 0.254 |
| thg1l | 0.254 |
| mib1 | 0.253 |
| rpl22 | 0.252 |
| mirn203 | 0.251 |
| camk2n1 | 0.251 |
| mettl9 | 0.251 |
| supt16h | 0.25 |
| yap1 | 0.25 |
| rbck1 | 0.25 |
| tg(krt14-cre)1efu | 0.249 |
| ube2l3 | 0.248 |
| zfp36l2 | 0.248 |
| plagl1 | 0.248 |
| fbxw7 | 0.248 |
| mnt | 0.247 |
| ube2l6 | 0.247 |
| ctdsp1 | 0.247 |
| cdc14a | 0.246 |
| sirt6 | 0.246 |
| sub1 | 0.246 |
| hace1 | 0.246 |
| ppp2r2b | 0.246 |
| mirn184 | 0.246 |
| nfyb | 0.246 |
| ppp2r5c | 0.245 |
| taf6 | 0.244 |
| d11mit271 | 0.244 |
| bcorl1 | 0.244 |
| ssrp1 | 0.244 |
| bard1 | 0.244 |
| acin1 | 0.243 |
| btg2 | 0.243 |
| uimc1 | 0.243 |
| ifrd1 | 0.242 |
| wnt9b | 0.242 |
| 1190002h23rik | 0.242 |
| cul4a | 0.242 |
| hnrnpul1 | 0.242 |
| plagl2 | 0.241 |
| myst1 | 0.241 |
| smarcd1 | 0.241 |
| rab25 | 0.241 |
| bnip3l | 0.24 |
| peg3 | 0.239 |
| rpn2 | 0.239 |
| cdk2ap1 | 0.239 |
| wisp2 | 0.239 |
| tnip1 | 0.239 |
| ksr1 | 0.238 |
| fbxo31 | 0.238 |
| foxo3 | 0.238 |
| rnf20 | 0.238 |
| tnk1 | 0.237 |
| snip1 | 0.237 |
| ptp4a1 | 0.237 |
| syvn1 | 0.236 |
| telo2 | 0.236 |
| dut | 0.236 |
| ddb1 | 0.235 |
| ube2a | 0.235 |
| coq2 | 0.235 |
| vrk3 | 0.234 |
| atmin | 0.234 |
| emi1 | 0.233 |
| cep164 | 0.233 |
| ube2d1 | 0.233 |
| aven | 0.232 |
| ptprv | 0.232 |
| mta2 | 0.232 |
| cirbp | 0.232 |
| myst2 | 0.232 |
| lasp1 | 0.232 |
| rb1cc1 | 0.231 |
| macrod1 | 0.231 |
| aldh4a1 | 0.231 |
| cdc14b | 0.231 |
| apbb2 | 0.231 |
| rbbp8 | 0.23 |
| iqcb1 | 0.23 |
| hspa9 | 0.23 |
| pogk | 0.229 |
| setd8 | 0.229 |
| ahcyl1 | 0.229 |
| clspn | 0.229 |
| ifitm1 | 0.229 |
| s100a6 | 0.229 |
| stk4 | 0.229 |
| maml1 | 0.228 |
| arhgef2 | 0.228 |
| ndn | 0.228 |
| nat5 | 0.227 |
| bok | 0.226 |
| ppp2r1a | 0.226 |
| itch | 0.226 |
| maged1 | 0.226 |
| sipa1l1 | 0.226 |
| prc1 | 0.226 |
| tpp2 | 0.226 |
| rplp1 | 0.225 |
| aof2 | 0.225 |
| il24 | 0.225 |
| daxx | 0.225 |
| mnat1 | 0.225 |
| tg(k6odctr)55tgo | 0.224 |
| ssbp2 | 0.224 |
| mdc1 | 0.224 |
| eda2r | 0.224 |
| pias4 | 0.223 |
| bcl11b | 0.223 |
| prim1 | 0.223 |
| bclaf1 | 0.223 |
| dapk3 | 0.223 |
| usp11 | 0.222 |
| maf1 | 0.222 |
| pbk | 0.221 |
| rps27 | 0.221 |
| pecr | 0.221 |
| coasy | 0.221 |
| unc5a | 0.221 |
| fdxr | 0.221 |
| dek | 0.22 |
| gtf2e2 | 0.22 |
| topbp1 | 0.22 |
| 6330569m22rik | 0.22 |
| d330017j20rik | 0.22 |
| arl2 | 0.22 |
| nap1l1 | 0.22 |
| thoc1 | 0.22 |
| cops5 | 0.219 |
| zbtb38 | 0.219 |
| nqo2 | 0.218 |
| cops2 | 0.218 |
| dpm1 | 0.218 |
| sirt5 | 0.218 |
| sp100 | 0.217 |
| ndrg1 | 0.217 |
| hist1h1c | 0.217 |
| rps9 | 0.217 |
| 1500019g21rik | 0.217 |
| rrm2 | 0.217 |
| trp73 | 0.217 |
| clic4 | 0.217 |
| casp2 | 0.217 |
| gdf15 | 0.216 |
| zmynd11 | 0.216 |
| d12ertd647e | 0.216 |
| lats1 | 0.216 |
| clca5 | 0.216 |
| pias2 | 0.216 |
| ubqln2 | 0.216 |
| taf10 | 0.216 |
| eef2 | 0.216 |
| pdcd5 | 0.216 |
| btg4 | 0.215 |
| spink7 | 0.214 |
| taf1 | 0.214 |
| dclre1a | 0.213 |
| ubtf | 0.213 |
| ppm1a | 0.212 |
| st7 | 0.212 |
| ttk | 0.212 |
| nupr1 | 0.212 |
| psmd9 | 0.211 |
| twist2 | 0.211 |
| chd1l | 0.211 |
| sdcbp | 0.211 |
| mad2l1bp | 0.211 |
| efemp2 | 0.21 |
| dapk1 | 0.209 |
| c1d | 0.209 |
| b4galt2 | 0.209 |
| gtf2a2 | 0.208 |
| hagh | 0.208 |
| ciapin1 | 0.208 |
| klf4 | 0.208 |
| gsta4 | 0.208 |
| nfya | 0.208 |
| tmem158 | 0.207 |
| dnaja3 | 0.207 |
| d4mit204 | 0.207 |
| carp1 | 0.206 |
| cdc42ep2 | 0.206 |
| brcc3 | 0.206 |
| cops8 | 0.206 |
| scn3b | 0.206 |
| fbxo38 | 0.205 |
| zfp110 | 0.205 |
| cdk8 | 0.205 |
| pkmyt1 | 0.205 |
| slc6a6 | 0.205 |
| paxip1 | 0.204 |
| s100a4 | 0.204 |
| axin1 | 0.204 |
| pdzd2 | 0.204 |
| s100a11 | 0.204 |
| bnip3 | 0.204 |
| 6620401k05rik | 0.204 |
| bik | 0.204 |
| 2810417h13rik | 0.204 |
| ncl | 0.203 |
| ckap2 | 0.203 |
| mdm3 | 0.202 |
| gna12 | 0.202 |
| ube2k | 0.202 |
| rrm1 | 0.202 |
| sulf2 | 0.201 |
| uba6 | 0.201 |
| endog | 0.201 |
| ddit4 | 0.201 |
| abi3bp | 0.201 |
| zmym4 | 0.201 |
| 1110008f13rik | 0.2 |
| serpinb5 | 0.2 |
| bc039210 | 0.2 |
| csnk1d | 0.2 |
| tprgl | 0.199 |
| ndrg2 | 0.199 |
| nfic | 0.199 |
| rrn3 | 0.199 |
| atf3 | 0.199 |
| ube2n | 0.199 |
| tcfap2c | 0.199 |
| prodh | 0.198 |
| shfm1 | 0.198 |
| dbc1 | 0.198 |
| hint1 | 0.198 |
| cdc25c | 0.198 |
| tegt | 0.198 |
| 5830417c01rik | 0.198 |
| tacc3 | 0.198 |
| chek2 | 0.198 |
| fem1a | 0.197 |
| ube2e3 | 0.197 |
| ppp2r2a | 0.197 |
| pml | 0.197 |
| rnd3 | 0.196 |
| pin1 | 0.196 |
| irs3 | 0.196 |
| prima1 | 0.195 |
| taf8 | 0.195 |
| max | 0.194 |
| smarcc1 | 0.194 |
| ptchd2 | 0.194 |
| hist2h4 | 0.194 |
| d4mit310 | 0.194 |
| wee1 | 0.193 |
| stk40 | 0.193 |
| hnrnpk | 0.193 |
| klf6 | 0.192 |
| meg3 | 0.192 |
| pias1 | 0.192 |
| mirn199a-1 | 0.192 |
| tbx2 | 0.192 |
| mad1l1 | 0.192 |
| ybx1 | 0.191 |
| e2f3 | 0.191 |
| asah1 | 0.191 |
| rbm3 | 0.191 |
| lgals7 | 0.191 |
| dnajb4 | 0.191 |
| tbx3 | 0.191 |
| cct5 | 0.191 |
| ppp2r4 | 0.191 |
| ddx5 | 0.191 |
| tsg101 | 0.19 |
| ppp1r1c | 0.19 |
| ect2 | 0.19 |
| ssbp1 | 0.19 |
| cks1b | 0.19 |
| pcaf | 0.19 |
| mapkapk5 | 0.19 |
| zfp523 | 0.19 |
| fbxl10 | 0.189 |
| utp14b | 0.189 |
| gzmk | 0.189 |
| pctr2 | 0.188 |
| nmi | 0.188 |
| nsmce4a | 0.188 |
| cul5 | 0.188 |
| numb | 0.187 |
| tmub1 | 0.187 |
| rev3l | 0.186 |
| tacc2 | 0.186 |
| cpeb1 | 0.186 |
| eif5a | 0.186 |
| pdcd4 | 0.186 |
| dact2 | 0.185 |
| acd | 0.185 |
| uhrf1 | 0.185 |
| nuak1 | 0.185 |
| chek1 | 0.185 |
| ywhag | 0.184 |
| hnrnpu | 0.184 |
| s100a14 | 0.184 |
| gtf2a1 | 0.184 |
| pcid2 | 0.184 |
| moap1 | 0.183 |
| baiap2l1 | 0.183 |
| prkra | 0.183 |
| kin | 0.183 |
| cad | 0.182 |
| chfr | 0.182 |
| sirt2 | 0.182 |
| nubp1 | 0.182 |
| eif3s10 | 0.182 |
| ercc3 | 0.182 |
| apc2 | 0.181 |
| mycs | 0.181 |
| rad51ap1 | 0.181 |
| ddx17 | 0.181 |
| nfx1 | 0.181 |
| cdkn2d | 0.18 |
| foxn3 | 0.18 |
| bag1 | 0.18 |
| mus81 | 0.18 |
| atf5 | 0.18 |
| zswim2 | 0.18 |
| nedd8 | 0.18 |
| myo18b | 0.18 |
| creg1 | 0.18 |
| rgs3 | 0.18 |
| fbxo6 | 0.179 |
| itpk1 | 0.179 |
| ddx18 | 0.179 |
| pttg1 | 0.179 |
| tsp50 | 0.179 |
| cdc6 | 0.179 |
| gna13 | 0.178 |
| mtss1 | 0.178 |
| top3b | 0.178 |
| mapkapk2 | 0.178 |
| cdc25a | 0.177 |
| hist2h2ac | 0.177 |
| lrrc4 | 0.177 |
| pcnp | 0.177 |
| bap1 | 0.177 |
| rnf7 | 0.177 |
| ncam2 | 0.177 |
| senp1 | 0.177 |
| cdkn2c | 0.176 |
| ube2q2 | 0.176 |
| pum2 | 0.176 |
| in(x)1h | 0.176 |
| kank1 | 0.176 |
| foxo4 | 0.176 |
| cyc1 | 0.176 |
| wbp2 | 0.175 |
| tom1l2 | 0.175 |
| e2f2 | 0.175 |
| sox4 | 0.175 |
| cugbp2 | 0.175 |
| 2900092e17rik | 0.175 |
| hnrnpc | 0.175 |
| ppp2r5b | 0.175 |
| rassf5 | 0.174 |
| unc5b | 0.174 |
| axin2 | 0.174 |
| exosc9 | 0.174 |
| anp32a | 0.174 |
| fhl2 | 0.174 |
| olfr412 | 0.174 |
| npm1 | 0.174 |
| tmbim4 | 0.173 |
| eif2a | 0.173 |
| tfdp1 | 0.173 |
| tep1 | 0.173 |
| bhlhb2 | 0.173 |
| vtcn1 | 0.173 |
| phb | 0.172 |
| bc005685 | 0.171 |
| mvp | 0.171 |
| skil | 0.171 |
| thrsp | 0.171 |
| ctbp1 | 0.171 |
| hmgb2 | 0.171 |
| ubr5 | 0.171 |
| ftmt | 0.171 |
| rnaseh2a | 0.17 |
| ccnl2 | 0.17 |
| wsb1 | 0.17 |
| padi4 | 0.17 |
| scrib | 0.17 |
| ikzf2 | 0.17 |
| ard1 | 0.17 |
| nov | 0.17 |
| arf1 | 0.169 |
| xaf1 | 0.169 |
| nfia | 0.169 |
| fbxo5 | 0.169 |
| mll5 | 0.169 |
| ubd | 0.169 |
| btrc | 0.168 |
| dpp7 | 0.168 |
| atr | 0.168 |
| mobkl1b | 0.168 |
| ccnk | 0.168 |
| eps8l1 | 0.168 |
| ccnh | 0.168 |
| ankrd2 | 0.168 |
| ss18 | 0.168 |
| cdca8 | 0.168 |
| e2f4 | 0.168 |
| dguok | 0.168 |
| ppm1g | 0.167 |
| sumo3 | 0.167 |
| rem2 | 0.167 |
| ebna1bp2 | 0.167 |
| hax1 | 0.167 |
| zfp384 | 0.166 |
| mycn | 0.166 |
| rbl2 | 0.166 |
| baiap2 | 0.166 |
| bnipl | 0.166 |
| ube2e1 | 0.166 |
| ccna1 | 0.166 |
| ctsq | 0.165 |
| tg(bcl2l1)2cbt | 0.165 |
| rnf8 | 0.165 |
| gltscr2 | 0.165 |
| bat4 | 0.165 |
| stub1 | 0.165 |
| snf1lk | 0.164 |
| ptbp2 | 0.164 |
| ai462493 | 0.164 |
| rhbdd3 | 0.164 |
| aifm1 | 0.164 |
| cnot7 | 0.164 |
| neurod6 | 0.164 |
| trp63 | 0.164 |
| prep | 0.164 |
| hsph1 | 0.164 |
| apaf1 | 0.163 |
| dffa | 0.163 |
| aldh18a1 | 0.163 |
| s100a1 | 0.163 |
| pa2g4 | 0.163 |
| trim28 | 0.163 |
| bub1 | 0.163 |
| hdgf | 0.163 |
| zkscan3 | 0.162 |
| snai2 | 0.162 |
| aprt | 0.162 |
| rad23b | 0.162 |
| pim3 | 0.162 |
| nae1 | 0.162 |
| bin1 | 0.162 |
| stk11 | 0.162 |
| sav1 | 0.161 |
| sumo2 | 0.161 |
| cited1 | 0.161 |
| hrk | 0.161 |
| casp6 | 0.161 |
| eg435755 | 0.161 |
| tmem85 | 0.161 |
| mapk10 | 0.161 |
| tspan13 | 0.161 |
| bcl9l | 0.161 |
| zdhhc16 | 0.16 |
| pou4f1 | 0.16 |
| mapk11 | 0.16 |
| nbl1 | 0.16 |
| ptrh2 | 0.16 |
| ctbp2 | 0.16 |
| shf | 0.16 |
| anxa7 | 0.16 |
| mapk6 | 0.159 |
| n4bp1 | 0.159 |
| sco2 | 0.159 |
| dab2ip | 0.158 |
| p4ha2 | 0.158 |
| smyd4 | 0.158 |
| tg(mmtvneu)202mul | 0.158 |
| anxa6 | 0.158 |
| itm2b | 0.158 |
| sema3b | 0.158 |
| hdac1-ps | 0.158 |
| ppp2cb | 0.158 |
| rab35 | 0.158 |
| d15ertd621e | 0.158 |
| rcn2 | 0.158 |
| spin2 | 0.158 |
| rapop4 | 0.157 |
| aurka | 0.157 |
| cdk7 | 0.157 |
| mad2l1 | 0.157 |
| gbp1 | 0.157 |
| taf12 | 0.157 |
| mina | 0.157 |
| smurf2 | 0.157 |
| dido1 | 0.157 |
| serpinh1 | 0.156 |
| tsp1 | 0.156 |
| dmc1 | 0.156 |
| csnk1e | 0.156 |
| metap2 | 0.156 |
| ppp2ca | 0.156 |
| rgs16 | 0.155 |
| cpne7 | 0.155 |
| 2810002n01rik | 0.155 |
| bphl | 0.155 |
| cald1 | 0.155 |
| dclre1c | 0.155 |
| mbd4 | 0.155 |
| nkx3-1 | 0.155 |
| 4931417g12rik | 0.155 |
| prkab1 | 0.155 |
| nlk | 0.155 |
| mdh1 | 0.155 |
| myct1 | 0.155 |
| mrps11 | 0.154 |
| scara3 | 0.154 |
| irx5 | 0.154 |
| dclre1b | 0.154 |
| hist4h4 | 0.154 |
| brap | 0.154 |
| tbl1x | 0.154 |
| hes6 | 0.154 |
| sgpl1 | 0.154 |
| ccni | 0.153 |
| gtf3a | 0.153 |
| brms1 | 0.153 |
| bai3 | 0.153 |
| bcl2l12 | 0.153 |
| sirt1 | 0.153 |
| smarca2 | 0.153 |
| 2310056p07rik | 0.153 |
| ptma | 0.153 |
| wdr26 | 0.153 |
| pbrm1 | 0.153 |
| carm1 | 0.153 |
| mtdh | 0.152 |
| gstp2 | 0.152 |
| smarcb1 | 0.152 |
| pcgf2 | 0.152 |
| dennd4a | 0.152 |
| rhox3a | 0.152 |
| rhox2a | 0.152 |
| gcn5l2 | 0.152 |
| tmem102 | 0.152 |
| sharpin | 0.152 |
| nme3 | 0.151 |
| bc048355 | 0.151 |
| tnfrsf10b | 0.151 |
| stag1 | 0.151 |
| rbm5 | 0.151 |
| irf5 | 0.151 |
| gm2a | 0.151 |
| cdc7 | 0.151 |
| rxfp1 | 0.15 |
| tlsr4 | 0.15 |
| tlsr5 | 0.15 |
| rnf167 | 0.15 |
| prlh | 0.15 |
| npm3 | 0.15 |
| tec | 0.15 |
| stk3 | 0.15 |
| h2afx | 0.15 |
| h3f3a | 0.15 |
| rbl1 | 0.15 |
| ak1 | 0.15 |
| set | 0.15 |
| hoxa5 | 0.15 |
| zc3h10 | 0.15 |
| dvl2 | 0.149 |
| taf7 | 0.149 |
| cstf1 | 0.149 |
| phf17 | 0.149 |
| cmtm5 | 0.149 |
| zdhhc2 | 0.149 |
| ikzf3 | 0.149 |
| d9mit24 | 0.149 |
| cmtm8 | 0.149 |
| tceal3 | 0.149 |
| tob1 | 0.149 |
| magea9 | 0.148 |
| mapre3 | 0.148 |
| tcfap2a | 0.148 |
| alk | 0.148 |
| psmc5 | 0.148 |
| yy1 | 0.148 |
| rhbdd1 | 0.148 |
| arid1a | 0.148 |
| cited2 | 0.148 |
| rps19 | 0.148 |
| ripk5 | 0.148 |
| gpnmb | 0.148 |
| ubb | 0.148 |
| hus1 | 0.148 |
| ruvbl2 | 0.148 |
| nme1 | 0.147 |
| stmn1 | 0.147 |
| klf5 | 0.147 |
| stx6 | 0.147 |
| pla2r1 | 0.147 |
| nit1 | 0.147 |
| nap1l4 | 0.147 |
| trim32 | 0.147 |
| olig1 | 0.147 |
| tmem97 | 0.147 |
| drg1 | 0.146 |
| recql4 | 0.146 |
| rsl1d1 | 0.146 |
| terc | 0.146 |
| hmmr | 0.146 |
| pi4k2b | 0.146 |
| sugt1 | 0.146 |
| 5430437p03rik | 0.146 |
| trim69 | 0.146 |
| btbd14b | 0.146 |
| tmed4 | 0.145 |
| taf5l | 0.145 |
| sirt3 | 0.145 |
| dusp6 | 0.145 |
| tg(bcl2)1tsk | 0.145 |
| dcun1d1 | 0.145 |
| plekhf1 | 0.145 |
| jmjd2c | 0.145 |
| lig4 | 0.145 |
| c230052i12rik | 0.145 |
| nat10 | 0.145 |
| ankrd32 | 0.145 |
| ctnnbip1 | 0.144 |
| 2010109i03rik | 0.144 |
| s100b | 0.144 |
| top1 | 0.144 |
| ly76 | 0.144 |
| tmem132e | 0.143 |
| 6720467c03rik | 0.143 |
