# Supplementary material for: GIT2 Acts as a Potential Keystone Protein in Functional Hypothalamic Networks Associated with Age-Related Phenotypic Changes in Rats
Source: PLoS One. 2012 May 14;7(5):e36975. doi: 10.1371/journal.pone.0036975 (PMC3351446; doi:10.1371/journal.pone.0036975)
Supplement: Table S19 — GeneIndexer latent semantic indexing (LSI) of significantly-regulated ‘Calcium signaling’ KEGG pathway. Using the KEGG signaling pathway ‘Calcium signaling’ as an input term, a list of the top 1000 implicitly-correlated (LSI correlation score >0.1) was generated using a full genome background list. (DOC) [file pone.0036975.s023.doc]

**Table S19. GeneIndexer latent semantic indexing (LSI) of significantly-regulated ‘Calcium signaling’ KEGG pathway.** Using the KEGG signaling pathway ‘Calcium signaling’ as an input term, a list of the top 1000 implicitly-correlated (LSI correlation score >0.1) was generated using a full genome background list.

| ***Calcium signaling*** |  |
| --- | --- |
|  |  |
| **Protein Symbol** | **LSI correlation score** |
| d1mit508 | 0.761 |
| edk | 0.732 |
| mtmr15 | 0.689 |
| d1mit58 | 0.646 |
| ly6g6f | 0.644 |
| afap1l2 | 0.636 |
| cdc2b | 0.632 |
| sh3bp5 | 0.588 |
| srms | 0.585 |
| ryk-rs1 | 0.583 |
| gm944 | 0.583 |
| t(11;19)42h | 0.566 |
| ptpn18 | 0.565 |
| ink76 | 0.563 |
| shd | 0.559 |
| she | 0.559 |
| a630047e20rik | 0.547 |
| fer | 0.546 |
| shc4 | 0.54 |
| rcsd1 | 0.536 |
| dyrk1c | 0.534 |
| ptpn21 | 0.532 |
| dok3 | 0.528 |
| shf | 0.519 |
| stk24 | 0.519 |
| c230081a13rik | 0.509 |
| ryk-ps1 | 0.504 |
| anks1 | 0.503 |
| dapp1 | 0.496 |
| ysk4 | 0.496 |
| 1110012m11rik | 0.494 |
| ptprh | 0.492 |
| mapk15 | 0.49 |
| stap1 | 0.486 |
| sbk1 | 0.486 |
| styxl1 | 0.485 |
| ick | 0.484 |
| ptprr | 0.482 |
| rufy2 | 0.482 |
| iapls3-28 | 0.481 |
| fgfr3-ps | 0.479 |
| lax1 | 0.479 |
| sla2 | 0.478 |
| sh2d3c | 0.473 |
| dok4 | 0.472 |
| alpk2 | 0.471 |
| 3930401k13rik | 0.471 |
| matk | 0.469 |
| pick2 | 0.466 |
| pick5 | 0.466 |
| pick3 | 0.466 |
| pick4 | 0.466 |
| ptpn23 | 0.466 |
| ptpn4 | 0.464 |
| ptpn15 | 0.463 |
| emo1 | 0.463 |
| afap1 | 0.462 |
| efs | 0.462 |
| khdrbs2 | 0.461 |
| pag1 | 0.46 |
| camkv | 0.457 |
| bc | 0.457 |
| tyro3-rs1 | 0.456 |
| dennd3 | 0.456 |
| ptpn7 | 0.456 |
| usp6nl | 0.452 |
| dok2 | 0.451 |
| cbll1 | 0.451 |
| grb2-ps1 | 0.448 |
| stk38l | 0.448 |
| e030049g20rik | 0.448 |
| map3k9 | 0.447 |
| skap2 | 0.446 |
| hcls1 | 0.444 |
| tesk1 | 0.444 |
| cdkl2 | 0.443 |
| d2mit316 | 0.443 |
| ptk6 | 0.443 |
| dusp26 | 0.443 |
| lmtk3 | 0.442 |
| dusp11 | 0.442 |
| ptpre | 0.438 |
| frs3 | 0.438 |
| shcbp1 | 0.438 |
| dusp18 | 0.435 |
| rbks | 0.435 |
| frk | 0.435 |
| ltk | 0.435 |
| map4k3 | 0.434 |
| stap2 | 0.434 |
| ptpn12 | 0.434 |
| iph1 | 0.433 |
| d0wfb1e | 0.433 |
| pstpip1 | 0.432 |
| c330002i19rik | 0.432 |
| mpzl1 | 0.432 |
| mmv15 | 0.431 |
| pmv47 | 0.431 |
| pmv51 | 0.431 |
| mapk4 | 0.431 |
| ubash3a | 0.431 |
| tssk3 | 0.43 |
| snx26 | 0.43 |
| skap1 | 0.43 |
| clybl | 0.429 |
| sorbs3 | 0.429 |
| tnk1 | 0.429 |
| dusp2 | 0.428 |
| ubash3b | 0.428 |
| in(17)1t | 0.428 |
| stam | 0.426 |
| nrk | 0.426 |
| mlkl | 0.426 |
| ripk5 | 0.425 |
| gigyf1 | 0.424 |
| tom1l1 | 0.424 |
| tfg | 0.424 |
| ptpn3 | 0.423 |
| sh2d4a | 0.423 |
| aatk | 0.423 |
| bmx | 0.423 |
| ibtk | 0.422 |
| zc3hc1 | 0.422 |
| epha8 | 0.421 |
| fert2 | 0.421 |
| ptpn20 | 0.42 |
| dusp7 | 0.419 |
| mobkl1a | 0.419 |
| 1810043h04rik | 0.417 |
| dok1 | 0.417 |
| clk2 | 0.417 |
| arhgap26 | 0.416 |
| sh2d2a | 0.416 |
| sh2b2 | 0.416 |
| lpxn | 0.414 |
| dusp16 | 0.414 |
| 9130404d14rik | 0.414 |
| cblc | 0.414 |
| dusp5 | 0.414 |
| ptpdc1 | 0.412 |
| clk1 | 0.412 |
| ppm1e | 0.412 |
| map4k1 | 0.412 |
| lime1 | 0.412 |
| ptpn5 | 0.411 |
| ptprk | 0.411 |
| dusp23 | 0.41 |
| stk10 | 0.41 |
| slk | 0.41 |
| ak5 | 0.409 |
| shb | 0.407 |
| sla | 0.407 |
| shc2 | 0.404 |
| pik3ap1 | 0.404 |
| grb7 | 0.403 |
| phlppl | 0.403 |
| tnik | 0.403 |
| mapk6 | 0.402 |
| ptpn14 | 0.402 |
| micalcl | 0.402 |
| wee2 | 0.401 |
| 2610018g03rik | 0.401 |
| dusp3 | 0.401 |
| map3k13 | 0.4 |
| npcd | 0.4 |
| rell1 | 0.4 |
| rell2 | 0.4 |
| jund2 | 0.399 |
| hsh2d | 0.399 |
| sorbs2 | 0.399 |
| blk | 0.398 |
| dok5 | 0.398 |
| taok2 | 0.398 |
| map3k12 | 0.398 |
| grb14 | 0.397 |
| sh2d1b2 | 0.397 |
| dyrk2 | 0.396 |
| smok2a | 0.395 |
| abl2 | 0.394 |
| stam2 | 0.393 |
| dusp22 | 0.392 |
| ptpmt1 | 0.392 |
| d15mit13 | 0.392 |
| grap | 0.392 |
| ptpn9 | 0.39 |
| tnk2 | 0.39 |
| pkn2 | 0.39 |
| loc641201 | 0.39 |
| abi3 | 0.389 |
| dusp14 | 0.389 |
| frs2 | 0.389 |
| dusp19 | 0.387 |
| tec | 0.387 |
| pitpnm3 | 0.386 |
| ppfibp1 | 0.385 |
| spred3 | 0.385 |
| hisppd1 | 0.385 |
| ulk2 | 0.385 |
| ranbp10 | 0.384 |
| d5mit202 | 0.384 |
| map2k1ip1 | 0.384 |
| tesk2 | 0.384 |
| styk1 | 0.383 |
| dyrk4 | 0.383 |
| dusp4 | 0.383 |
| centd3 | 0.382 |
| ptprj | 0.382 |
| pik3r3 | 0.381 |
| dbnl | 0.381 |
| ngef | 0.38 |
| ptprb | 0.38 |
| tssk4 | 0.379 |
| shbdp1 | 0.379 |
| ptpro | 0.379 |
| ptpra | 0.378 |
| ptprcap | 0.378 |
| rp23-157o10.7 | 0.378 |
| nek3 | 0.377 |
| arhgap10 | 0.377 |
| taok1 | 0.377 |
| sirpb1 | 0.377 |
| 6330417g02rik | 0.376 |
| txk | 0.376 |
| fscb | 0.375 |
| sit1 | 0.375 |
| rbmxrt | 0.375 |
| trp53rk | 0.374 |
| hrbl | 0.374 |
| bc010304 | 0.374 |
| sh3pxd2a | 0.374 |
| rftn1 | 0.373 |
| mast4 | 0.373 |
| sav1 | 0.373 |
| map3k10 | 0.372 |
| phlpp | 0.372 |
| ptprf | 0.371 |
| sh3bp2 | 0.37 |
| d7mit151 | 0.37 |
| ptpn2 | 0.37 |
| shc3 | 0.37 |
| dgkk | 0.369 |
| dusp8 | 0.369 |
| twf2 | 0.368 |
| pip5kl1 | 0.368 |
| epha10 | 0.368 |
| gkap1 | 0.368 |
| nudt3 | 0.367 |
| mobkl1b | 0.367 |
| pstpip2 | 0.367 |
| socs4 | 0.367 |
| mark3 | 0.367 |
| nck2 | 0.366 |
| rasa3 | 0.366 |
| smok2b | 0.366 |
| smok3a | 0.366 |
| smok3b | 0.366 |
| tyro3 | 0.366 |
| csnk1g1 | 0.365 |
| loc436194 | 0.365 |
| plekha2 | 0.365 |
| d5mit90 | 0.364 |
| mak | 0.364 |
| stk3 | 0.363 |
| khdrbs1 | 0.363 |
| riok2 | 0.363 |
| phip | 0.363 |
| d1mit316 | 0.362 |
| odc-rs9 | 0.362 |
| rusc1 | 0.362 |
| ppp1r14d | 0.362 |
| pik3c2b | 0.361 |
| 0610011l14rik | 0.361 |
| dusp13 | 0.361 |
| ilkap | 0.361 |
| sh2b3 | 0.361 |
| dusp10 | 0.36 |
| irs4 | 0.36 |
| dyrk3 | 0.359 |
| tns1 | 0.359 |
| dusp9 | 0.359 |
| fert1 | 0.359 |
| gmfg | 0.358 |
| ostf1 | 0.358 |
| stk35 | 0.357 |
| taok3 | 0.357 |
| shoc2 | 0.357 |
| pkn3 | 0.357 |
| mapk8ip2 | 0.357 |
| pip4k2b | 0.357 |
| asb15 | 0.357 |
| gab3 | 0.356 |
| sh2b1 | 0.356 |
| ythdc1 | 0.355 |
| fgfr1op | 0.355 |
| tob2 | 0.354 |
| thoc5 | 0.354 |
| arhgef15 | 0.354 |
| sh2d1b1 | 0.353 |
| rin1 | 0.353 |
| dgkg | 0.353 |
| mtmr12 | 0.352 |
| sh3kbp1 | 0.352 |
| umpk-ps | 0.352 |
| ptpru | 0.352 |
| d11mit109 | 0.351 |
| d11mit205 | 0.351 |
| riok1 | 0.351 |
| yes1 | 0.351 |
| prpf4b | 0.351 |
| vrk3 | 0.35 |
| uck1 | 0.35 |
| eps8 | 0.349 |
| cabyr | 0.349 |
| spry3 | 0.349 |
| slmap | 0.349 |
| mtmr14 | 0.348 |
| prkd2 | 0.347 |
| abi2 | 0.347 |
| csnk1g3 | 0.346 |
| srpk2 | 0.345 |
| wdfy2 | 0.345 |
| nagk | 0.345 |
| fes | 0.344 |
| ptprm | 0.344 |
| ptprd | 0.344 |
| ryk | 0.344 |
| tssk2 | 0.343 |
| stk25 | 0.343 |
| wnk2 | 0.342 |
| olfr749 | 0.342 |
| mapbpip | 0.342 |
| styx | 0.341 |
| mapkapk5 | 0.341 |
| ppfia1 | 0.341 |
| nme6 | 0.341 |
| carhsp1 | 0.341 |
| spred2 | 0.34 |
| mobkl3 | 0.34 |
| slamf8 | 0.34 |
| stk38 | 0.34 |
| usp8 | 0.339 |
| ppp2r5b | 0.339 |
| ckt2 | 0.339 |
| cmpk2 | 0.339 |
| ptprt | 0.339 |
| cdcp1 | 0.338 |
| cdkl1 | 0.338 |
| pdik1l | 0.338 |
| il17rd | 0.338 |
| stk22s1 | 0.336 |
| cmpk1 | 0.336 |
| cnksr1 | 0.336 |
| itk | 0.335 |
| grb10 | 0.335 |
| mark4 | 0.334 |
| ttll1 | 0.334 |
| ttll4 | 0.334 |
| ccdc50 | 0.334 |
| cep68 | 0.334 |
| phpt1 | 0.334 |
| yod1 | 0.334 |
| cd300e | 0.334 |
| rasa1 | 0.333 |
| fnbp4 | 0.333 |
| plek | 0.333 |
| dbndd2 | 0.332 |
| csnk1g2 | 0.332 |
| ppp1r14b | 0.332 |
| arhgap5 | 0.331 |
| nek4 | 0.331 |
| acp1 | 0.331 |
| nek11 | 0.33 |
| oxsr1 | 0.33 |
| nek9 | 0.33 |
| socs6 | 0.33 |
| ptpn13 | 0.33 |
| mtap2k | 0.33 |
| clnk | 0.33 |
| ttbk2 | 0.329 |
| b230120h23rik | 0.329 |
| cd6 | 0.329 |
| nek6 | 0.329 |
| eef2k | 0.328 |
| nrbp1 | 0.328 |
| nuak1 | 0.328 |
| inppl1 | 0.327 |
| sos2 | 0.327 |
| pip4k2c | 0.327 |
| ptprs | 0.327 |
| dusp15 | 0.326 |
| clk3 | 0.326 |
| tlk2 | 0.326 |
| lyn-ps1 | 0.326 |
| fyb | 0.326 |
| melk | 0.326 |
| bc033915 | 0.326 |
| tssk1 | 0.326 |
| sfrs16 | 0.326 |
| mknk2 | 0.325 |
| insrr | 0.325 |
| sh3rf1 | 0.324 |
| ihpk1 | 0.324 |
| tns3 | 0.324 |
| rapgef1 | 0.324 |
| ptprq | 0.324 |
| 1110007c09rik | 0.324 |
| grap2 | 0.323 |
| map4k2 | 0.323 |
| ctps2 | 0.323 |
| snx6 | 0.323 |
| ptplb | 0.323 |
| map3k11 | 0.322 |
| mast1 | 0.322 |
| trat1 | 0.322 |
| pdap1 | 0.322 |
| centg3 | 0.321 |
| ephb6 | 0.321 |
| cpne3 | 0.321 |
| mapk12 | 0.32 |
| zmym2 | 0.32 |
| ankrd28 | 0.319 |
| map4k5 | 0.319 |
| tiprl | 0.319 |
| d15nds1 | 0.319 |
| sh3gl2 | 0.319 |
| bcar3 | 0.319 |
| ppp1r12c | 0.319 |
| map4k4 | 0.319 |
| spred1 | 0.318 |
| centg1 | 0.318 |
| ppp2r5d | 0.318 |
| them4 | 0.318 |
| cdc2l5 | 0.318 |
| ssfa3 | 0.318 |
| als2cr2 | 0.318 |
| d2mit4 | 0.318 |
| dusp6 | 0.317 |
| stk16 | 0.317 |
| pik3c2a | 0.317 |
| itpk1 | 0.317 |
| chn2 | 0.317 |
| ttbk1 | 0.316 |
| wdr68 | 0.316 |
| arhgap21 | 0.315 |
| ierepo2 | 0.315 |
| lmtk2 | 0.315 |
| pilrb1 | 0.315 |
| nedd9 | 0.314 |
| pskh1 | 0.314 |
| zfp641 | 0.314 |
| sh3bp4 | 0.314 |
| dgka | 0.314 |
| pak6 | 0.314 |
| dusp21 | 0.314 |
| prkrir | 0.314 |
| snf1lk | 0.313 |
| sh3bp1 | 0.313 |
| avil | 0.313 |
| ulk1 | 0.313 |
| ror1 | 0.313 |
| hnrnph2 | 0.313 |
| 9830130m13rik | 0.313 |
| csk | 0.313 |
| e130304f04rik | 0.313 |
| asb6 | 0.313 |
| mapk8ip3 | 0.312 |
| prkd3 | 0.312 |
| brsk1 | 0.312 |
| crkrs | 0.312 |
| mapkbp1 | 0.312 |
| epha3 | 0.311 |
| dok6 | 0.311 |
| akap4 | 0.311 |
| nrp | 0.311 |
| ddr2 | 0.311 |
| gab1 | 0.311 |
| lrrn3 | 0.311 |
| mtmr3 | 0.311 |
| ripk4 | 0.311 |
| dgkd | 0.31 |
| stk4 | 0.31 |
| nol1 | 0.31 |
| ddef1 | 0.309 |
| dub1 | 0.309 |
| gbas | 0.309 |
| abi1 | 0.309 |
| memo1 | 0.308 |
| spsb2 | 0.308 |
| nck1 | 0.308 |
| dusp12 | 0.307 |
| ihpk3 | 0.307 |
| st5 | 0.307 |
| amph | 0.307 |
| mark1 | 0.306 |
| grlf1 | 0.306 |
| irs3 | 0.306 |
| kank2 | 0.306 |
| snx9 | 0.306 |
| card14 | 0.306 |
| mtmr11 | 0.306 |
| mtmr10 | 0.306 |
| rasa2 | 0.306 |
| iapls3-10 | 0.306 |
| akap12 | 0.305 |
| ppp1r16a | 0.305 |
| sorbs1 | 0.305 |
| pdlim7 | 0.305 |
| nme7 | 0.305 |
| akap11 | 0.305 |
| pbk | 0.305 |
| bc042720 | 0.305 |
| cd300lb | 0.304 |
| d4mit29 | 0.304 |
| arhgap27 | 0.304 |
| mink1 | 0.304 |
| mapkapk3 | 0.304 |
| eps15l1 | 0.303 |
| ppp2r2c | 0.303 |
| ppm1a | 0.303 |
| lrrc25 | 0.303 |
| trip10 | 0.302 |
| mapkap1 | 0.302 |
| dapk3 | 0.302 |
| mast2 | 0.302 |
| dak | 0.302 |
| mtmr7 | 0.302 |
| khdrbs3 | 0.302 |
| blnk | 0.302 |
| ppm1j | 0.302 |
| pea15b | 0.301 |
| axl | 0.301 |
| stk40 | 0.3 |
| cdkl3 | 0.3 |
| pear1 | 0.3 |
| crkl | 0.3 |
| tk-ps2 | 0.299 |
| cmtm8 | 0.299 |
| mknk1 | 0.299 |
| cdc42bpa | 0.299 |
| ppp1r14c | 0.299 |
| zfp692 | 0.299 |
| 2310014g06rik | 0.299 |
| dyrk1b | 0.299 |
| rasal1 | 0.298 |
| brsk2 | 0.298 |
| cdkn3 | 0.298 |
| rps6ka4 | 0.298 |
| araf | 0.298 |
| ppp1r1c | 0.298 |
| tob1 | 0.298 |
| ctps | 0.298 |
| clk4 | 0.298 |
| pcbd2 | 0.298 |
| akap3 | 0.298 |
| plekhm3 | 0.297 |
| 1110008f13rik | 0.297 |
| ppm1b | 0.297 |
| epha1 | 0.297 |
| pstk | 0.297 |
| alpk1 | 0.296 |
| plekho1 | 0.296 |
| epgn | 0.296 |
| tbkbp1 | 0.296 |
| pask | 0.296 |
| ppme1 | 0.295 |
| sash3 | 0.295 |
| aak1 | 0.295 |
| ppm1f | 0.295 |
| map3k2 | 0.295 |
| vrk2 | 0.294 |
| lat2 | 0.294 |
| gulp1 | 0.293 |
| zdhhc16 | 0.293 |
| pak1ip1 | 0.293 |
| limk2 | 0.293 |
| mast3 | 0.293 |
| akap6 | 0.293 |
| hisppd2a | 0.293 |
| pmv23 | 0.293 |
| tssk6 | 0.292 |
| epha6 | 0.292 |
| ai462493 | 0.292 |
| zc3h15 | 0.292 |
| mical1 | 0.292 |
| a630042l21rik | 0.292 |
| agk | 0.291 |
| cfl2 | 0.29 |
| 1700009n14rik | 0.29 |
| stard10 | 0.29 |
| pik3r2 | 0.29 |
| srpk1 | 0.29 |
| pctk2 | 0.29 |
| aabpr | 0.29 |
| med28 | 0.29 |
| grit | 0.289 |
| dner | 0.289 |
| bcar1 | 0.289 |
| asb3 | 0.288 |
| ccrk | 0.287 |
| ranbp9 | 0.287 |
| d4mit112 | 0.287 |
| spry2 | 0.286 |
| efna1 | 0.286 |
| dub2 | 0.286 |
| magi3 | 0.286 |
| ephb1 | 0.286 |
| 381484 | 0.286 |
| cdv3 | 0.286 |
| wac | 0.285 |
| ipmk | 0.285 |
| pitpnb | 0.285 |
| lair1 | 0.285 |
| ppapdc1 | 0.285 |
| ppp1r12b | 0.285 |
| ksr2 | 0.285 |
| sgk2 | 0.284 |
| dgkq | 0.284 |
| stk39 | 0.284 |
| phkg1 | 0.284 |
| tubgcp4 | 0.284 |
| snx18 | 0.284 |
| errfi1 | 0.283 |
| 5033414k04rik | 0.283 |
| guk1 | 0.283 |
| cdk3 | 0.282 |
| pkmyt1 | 0.282 |
| stambp | 0.282 |
| gmfb | 0.282 |
| nlk | 0.282 |
| crk | 0.282 |
| mapk13 | 0.282 |
| unc119 | 0.282 |
| cdc2l1 | 0.281 |
| snrk | 0.281 |
| sh3gl3 | 0.281 |
| pctk3 | 0.281 |
| uhmk1 | 0.281 |
| gtf2i | 0.28 |
| ssh1 | 0.28 |
| dolk | 0.28 |
| d4mit145 | 0.28 |
| plek2 | 0.28 |
| pip5k1c | 0.28 |
| a530064d06rik | 0.279 |
| synj1 | 0.279 |
| ppp2r4 | 0.279 |
| cdgap | 0.279 |
| ptk7 | 0.279 |
| eps15 | 0.279 |
| clip3 | 0.279 |
| map3k6 | 0.279 |
| trip6 | 0.278 |
| clec4b2 | 0.278 |
| fiz1 | 0.278 |
| frat2 | 0.278 |
| pip5k3 | 0.278 |
| pim3 | 0.278 |
| lasp1 | 0.278 |
| dgke | 0.277 |
| ube2r2 | 0.277 |
| socs5 | 0.277 |
| es14 | 0.277 |
| clec4b1 | 0.277 |
| camk2n2 | 0.277 |
| cd300lf | 0.277 |
| ublcp1 | 0.277 |
| xmv19 | 0.277 |
| reps2 | 0.277 |
| nek10 | 0.276 |
| dgkb | 0.276 |
| pitpnm2 | 0.276 |
| cdc37 | 0.276 |
| gnb2l1 | 0.276 |
| ros1 | 0.276 |
| tspan15 | 0.276 |
| eps8l2 | 0.275 |
| ksr1 | 0.275 |
| reps1 | 0.275 |
| nuak2 | 0.275 |
| lcp2 | 0.275 |
| ssh3 | 0.275 |
| scyl2 | 0.275 |
| dimt1 | 0.275 |
| clec1b | 0.274 |
| ficd | 0.274 |
| camk2n1 | 0.274 |
| sh3gl1 | 0.274 |
| socs7 | 0.274 |
| slamf6 | 0.274 |
| mapk8ip1 | 0.274 |
| pctk1 | 0.274 |
| mertk | 0.274 |
| ptprg | 0.274 |
| map2k5 | 0.274 |
| syngap1 | 0.273 |
| ror2 | 0.273 |
| pak7 | 0.273 |
| dok7 | 0.273 |
| akt1s1 | 0.273 |
| ppp1r15b | 0.273 |
| tic1 | 0.273 |
| rgnef | 0.272 |
| tmed8 | 0.272 |
| pip4k2a | 0.272 |
| ptprz1 | 0.272 |
| fgr | 0.272 |
| ccdc134 | 0.272 |
| vav2 | 0.272 |
| caskin2 | 0.272 |
| rs | 0.271 |
| gak | 0.271 |
| cdc42bpb | 0.271 |
| itgb1bp1 | 0.271 |
| tyk2 | 0.271 |
| bc060632 | 0.271 |
| hipk3 | 0.271 |
| pik3r5 | 0.271 |
| dlgap4 | 0.27 |
| pkia | 0.27 |
| 2610019a05rik | 0.27 |
| akap1 | 0.27 |
| gsbs | 0.27 |
| ms4a3 | 0.27 |
| yap1 | 0.27 |
| map3k4 | 0.27 |
| ppm1m | 0.27 |
| pi4k2b | 0.27 |
| ccdc6 | 0.27 |
| a230067g21rik | 0.27 |
| gp49a | 0.269 |
| pip5k1b | 0.269 |
| chchd3 | 0.269 |
| pkn1 | 0.269 |
| itfg1 | 0.269 |
| zfp383 | 0.269 |
| chn1 | 0.269 |
| hgs | 0.269 |
| 3110043j09rik | 0.268 |
| rps6ka2 | 0.268 |
| brp13 | 0.268 |
| galk2 | 0.268 |
| arpp21 | 0.268 |
| rhou | 0.268 |
| mtss1 | 0.268 |
| wdr26 | 0.268 |
| alk | 0.268 |
| dnm3 | 0.268 |
| zcchc8 | 0.268 |
| dyrk1a | 0.267 |
| prkch | 0.267 |
| mapk7 | 0.267 |
| caskin1 | 0.267 |
| epha2 | 0.267 |
| cd84 | 0.267 |
| rsu1 | 0.266 |
| rps6ka3 | 0.266 |
| jub | 0.266 |
| sdpr | 0.266 |
| zfp622 | 0.266 |
| pi4ka | 0.265 |
| spry1 | 0.265 |
| ptpn1 | 0.265 |
| cnksr2 | 0.265 |
| itpkb | 0.265 |
| coro7 | 0.265 |
| spg21 | 0.265 |
| 2700078k21rik | 0.265 |
| sirpa | 0.265 |
| ppm1h | 0.265 |
| gab2 | 0.265 |
| rbm16 | 0.265 |
| cab39 | 0.265 |
| sdcbp | 0.264 |
| bckdk | 0.264 |
| cdc42bpg | 0.264 |
| stk17b | 0.264 |
| prkrip1 | 0.264 |
| nek7 | 0.263 |
| scamp3 | 0.263 |
| d5mit175 | 0.263 |
| fastk | 0.263 |
| ptp4a1 | 0.263 |
| spry4 | 0.263 |
| cdc2l6 | 0.263 |
| eps8l3 | 0.263 |
| arhgef6 | 0.262 |
| rps6kb2 | 0.262 |
| mtmr6 | 0.262 |
| akap8 | 0.262 |
| coro1b | 0.262 |
| wnk1 | 0.262 |
| csf2rb2 | 0.262 |
| fry | 0.262 |
| eml4 | 0.262 |
| d2mit58 | 0.262 |
| kndc1 | 0.261 |
| vrk1 | 0.261 |
| rhov | 0.261 |
| ddr1 | 0.261 |
| zfand3 | 0.261 |
| ralgps2 | 0.261 |
| pdcd6ip | 0.261 |
| ppig | 0.261 |
| rp23-136k12.4 | 0.26 |
| nudt16l1 | 0.26 |
| ppp1r2 | 0.26 |
| mapk11 | 0.26 |
| smek1 | 0.26 |
| 4833426j09rik | 0.26 |
| plscr1 | 0.26 |
| cblb | 0.26 |
| spag9 | 0.26 |
| hck | 0.26 |
| d5mit274 | 0.26 |
| musk | 0.26 |
| pdk2 | 0.259 |
| hsp86-ps2 | 0.259 |
| pdcl | 0.259 |
| ropn1 | 0.259 |
| adk | 0.259 |
| spnb5 | 0.259 |
| nckipsd | 0.259 |
| fuk | 0.259 |
| enpp4 | 0.259 |
| 2610207i05rik | 0.258 |
| ihpk2 | 0.258 |
| loc667882 | 0.258 |
| rps6ka5 | 0.258 |
| elk4 | 0.258 |
| nenf | 0.258 |
| ppm1k | 0.258 |
| jdp2 | 0.258 |
| csf2rb | 0.257 |
| twf1 | 0.257 |
| ctdspl | 0.257 |
| d7mit15 | 0.257 |
| git2 | 0.257 |
| synj2 | 0.257 |
| ssh2 | 0.257 |
| rasgrp3 | 0.257 |
| zfyve27 | 0.257 |
| pcnp | 0.257 |
| cad | 0.257 |
| zfyve16 | 0.257 |
| cnpy2 | 0.257 |
| igbp1 | 0.257 |
| hs1bp3 | 0.256 |
| ppp2r5a | 0.256 |
| tmprss13 | 0.256 |
| camk1g | 0.256 |
| itgb1bp3 | 0.256 |
| ephb2 | 0.256 |
| hnrnpk | 0.255 |
| wee1 | 0.255 |
| zbed3 | 0.255 |
| grasp | 0.255 |
| ppp4c | 0.255 |
| trio | 0.255 |
| krt27 | 0.255 |
| dnm1 | 0.255 |
| rpl36a | 0.255 |
| ccdc88c | 0.255 |
| samsn1 | 0.254 |
| itpka | 0.254 |
| cct2 | 0.254 |
| ccdc19 | 0.254 |
| cd79b | 0.254 |
| uck2 | 0.254 |
| phkg2 | 0.254 |
| appl1 | 0.253 |
| plk3 | 0.253 |
| rufy1 | 0.253 |
| arhgap1 | 0.253 |
| rabep2 | 0.253 |
| camkk1 | 0.253 |
| glrx3 | 0.253 |
| limk1 | 0.253 |
| pak4 | 0.253 |
| tln2 | 0.253 |
| nme3 | 0.253 |
| clcf1 | 0.253 |
| cdk10 | 0.253 |
| ddef2 | 0.252 |
| slamf1 | 0.252 |
| epha5 | 0.252 |
| pacsin1 | 0.252 |
| olfr1359 | 0.252 |
| paqr3 | 0.252 |
| ppp1r14a | 0.252 |
| pi4k2a | 0.252 |
| d11mit208 | 0.252 |
| ctdsp2 | 0.252 |
| wbp2 | 0.251 |
| cd72 | 0.251 |
| ccdc88a | 0.251 |
| myo18a | 0.251 |
| kif20b | 0.251 |
| eif3b | 0.251 |
| kank1 | 0.251 |
| kifc4b | 0.251 |
| 2810004i08rik | 0.251 |
| g3bp1 | 0.251 |
| efnb1 | 0.25 |
| nisch | 0.25 |
| prkd1 | 0.25 |
| plcg2 | 0.25 |
| cd163l1 | 0.25 |
| sh3d19 | 0.25 |
| rps6ka6 | 0.25 |
| spsb1 | 0.249 |
| mcf2l | 0.249 |
| zyx | 0.249 |
| lats1 | 0.249 |
| klri1 | 0.249 |
| mrvi1 | 0.249 |
| pdk1 | 0.248 |
| d5nds2 | 0.248 |
| lrig1 | 0.248 |
| zfp259 | 0.248 |
| gcn1l1 | 0.248 |
| npm3-ps1 | 0.248 |
| cspg5 | 0.248 |
| ttyh2 | 0.248 |
| il17re | 0.248 |
| rnf41 | 0.247 |
| hist1h1b | 0.247 |
| trib2 | 0.247 |
| snf1lk2 | 0.247 |
| prkx | 0.247 |
| centg2 | 0.247 |
| centa1 | 0.247 |
| dtymk | 0.246 |
| mbip | 0.246 |
| parva | 0.246 |
| camk2g | 0.246 |
| rngtt | 0.246 |
| bnip2 | 0.246 |
| asb9 | 0.246 |
| bc032265 | 0.246 |
| marcksl1 | 0.246 |
| dock1 | 0.246 |
| eif2ak1 | 0.246 |
| ppm1g | 0.246 |
| lat | 0.245 |
| erbb2ip | 0.245 |
| bag2 | 0.245 |
| ylpm1 | 0.245 |
| ilk-rs | 0.245 |
| inpp4a | 0.245 |
| cables1 | 0.245 |
| slamf9 | 0.245 |
| syngr2 | 0.245 |
| glyctk | 0.245 |
| 1700007e06rik | 0.245 |
| cd2ap | 0.244 |
| habp4 | 0.244 |
| kirrel | 0.244 |
| brwd3 | 0.244 |
| wbp1 | 0.244 |
| mos | 0.243 |
| ptprv | 0.243 |
| 1110006o17rik | 0.243 |
| tgfb1i1 | 0.243 |
| 4933433p14rik | 0.243 |
| nme4 | 0.243 |
| pip5k1a | 0.243 |
| d6mit296 | 0.243 |
| d11mit215 | 0.243 |
| rp9 | 0.242 |
| pik3c2g | 0.242 |
| trib1 | 0.242 |
| wbp2nl | 0.242 |
| tpte | 0.242 |
| pak3 | 0.242 |
| fgf22 | 0.242 |
| hmga1-rs1 | 0.242 |
| sos1 | 0.242 |
| nckap1 | 0.241 |
| mkln1 | 0.241 |
| cep170 | 0.241 |
| btbd10 | 0.241 |
| gem | 0.241 |
| elf2 | 0.241 |
| cdc25b | 0.241 |
| 1500005k14rik | 0.241 |
| pdk3 | 0.241 |
| cald1 | 0.241 |
| tg(erbb2)1jek | 0.24 |
| wnk3 | 0.24 |
| jak1 | 0.24 |
| lcp1 | 0.24 |
| akt3 | 0.24 |
| rhbdl1 | 0.24 |
| riok3 | 0.24 |
| cish | 0.24 |
| cdc5l | 0.24 |
| cops4 | 0.24 |
| plcl2 | 0.239 |
| card11 | 0.239 |
| rasgrf1 | 0.239 |
