# Supplementary material for: GIT2 Acts as a Potential Keystone Protein in Functional Hypothalamic Networks Associated with Age-Related Phenotypic Changes in Rats
Source: PLoS One. 2012 May 14;7(5):e36975. doi: 10.1371/journal.pone.0036975 (PMC3351446; doi:10.1371/journal.pone.0036975)
Supplement: Table S22 — GeneIndexer latent semantic indexing (LSI) of significantly-regulated ‘Anatomical structural development’ GO term group. Using the GO term group ‘Anatomical structural development’ as an input term, a list of the top 1000 implicitly-correlated (LSI correlation score >0.1) was generated using a full genome background list. (DOC) [file pone.0036975.s026.doc]

**Table S22. GeneIndexer latent semantic indexing (LSI) of significantly-regulated ‘Anatomical structural development’ GO term group.** Using the GO term group ‘Anatomical structural development’ as an input term, a list of the top 1000 implicitly-correlated (LSI correlation score >0.1) was generated using a full genome background list.

| ***Anatomical structure development*** |  |
| --- | --- |
|  |  |
| **Protein Symbol** | **LSI correlation score** |
| ebf4 | 0.597 |
| 1190005f20rik | 0.588 |
| foxi2 | 0.587 |
| lingo2 | 0.578 |
| bc072620 | 0.578 |
| tcstv1 | 0.576 |
| zscan4f | 0.576 |
| unkh | 0.576 |
| unkl | 0.576 |
| tg(zfp38)d1htz | 0.575 |
| tg(zfp38)y7htz | 0.575 |
| tg(zfp38)b8htz | 0.575 |
| tg(zfp38)a4htz | 0.575 |
| tmem16k | 0.574 |
| slfn7 | 0.572 |
| slfn14 | 0.572 |
| slfn6 | 0.572 |
| bzw2 | 0.57 |
| mab21l2 | 0.569 |
| six3os1 | 0.566 |
| ebf2 | 0.564 |
| prox2 | 0.563 |
| slfn9 | 0.562 |
| slfn4 | 0.562 |
| tmem16h | 0.561 |
| neurl | 0.561 |
| obox2 | 0.559 |
| obox5 | 0.559 |
| obox6 | 0.559 |
| obox1 | 0.559 |
| obox4 | 0.559 |
| punc | 0.559 |
| fjx1 | 0.555 |
| dmrta1 | 0.555 |
| prrxl1 | 0.555 |
| dnm3os | 0.554 |
| abhd14a | 0.554 |
| mirn214 | 0.554 |
| zscan21 | 0.553 |
| olfml3 | 0.55 |
| lingo4 | 0.55 |
| veph1 | 0.549 |
| mirn182 | 0.549 |
| celsr3 | 0.548 |
| cntnap5a | 0.547 |
| csrnp3 | 0.547 |
| dopey2 | 0.546 |
| c230078m14rik | 0.546 |
| obox3 | 0.545 |
| rttn | 0.543 |
| lrrtm4 | 0.543 |
| lrrtm2 | 0.543 |
| lhx8 | 0.542 |
| nrbp2 | 0.542 |
| mirn449a | 0.541 |
| cntnap5c | 0.541 |
| kif26b | 0.54 |
| igsf9 | 0.538 |
| foxb1 | 0.538 |
| 1110007l15rik | 0.538 |
| am | 0.537 |
| narg2 | 0.537 |
| irx6 | 0.537 |
| msi2 | 0.537 |
| dxmit65 | 0.537 |
| mirn199a-2 | 0.537 |
| cdh20 | 0.537 |
| tmem16f | 0.537 |
| prr15 | 0.537 |
| v2r8 | 0.536 |
| tmem141 | 0.535 |
| d2wsu32e | 0.535 |
| mirn125b-1 | 0.535 |
| mirn125b-2 | 0.535 |
| rhox13 | 0.535 |
| trim71 | 0.535 |
| olfr459 | 0.534 |
| dmrta2 | 0.533 |
| ifitm7 | 0.532 |
| ifitm6 | 0.532 |
| pcdhac2 | 0.532 |
| zfp503 | 0.532 |
| ovol2 | 0.531 |
| slfn3 | 0.531 |
| zfp54 | 0.531 |
| fat3 | 0.53 |
| 2610110g12rik | 0.53 |
| foxj2 | 0.53 |
| mospd3 | 0.529 |
| foxj3 | 0.528 |
| lbx2 | 0.527 |
| bc029169 | 0.525 |
| foxs1 | 0.525 |
| dmbx1 | 0.525 |
| mea | 0.525 |
| xm_359419 | 0.524 |
| mrps25 | 0.523 |
| mirn125a | 0.522 |
| 1700021k02rik | 0.522 |
| zfp326 | 0.522 |
| dmrtc1c | 0.522 |
| dmrtc1b | 0.522 |
| dmrtc1a | 0.522 |
| 3110032g18rik | 0.522 |
| mirnlet7c-1 | 0.521 |
| mirnlet7c-2 | 0.521 |
| ebf3 | 0.52 |
| gsc2 | 0.52 |
| zfp462 | 0.519 |
| dmrt3 | 0.519 |
| d16ertd6e | 0.519 |
| mkx | 0.519 |
| tcfap2d | 0.518 |
| zfp2 | 0.518 |
| pcp4l1 | 0.518 |
| ferd3l | 0.518 |
| arid3b | 0.518 |
| pelo | 0.518 |
| rhox8 | 0.518 |
| 1500005k14rik | 0.518 |
| rhox9 | 0.516 |
| vmn2r122 | 0.516 |
| d8mit284 | 0.516 |
| lrrn1 | 0.516 |
| phf15 | 0.515 |
| sii6 | 0.515 |
| mirn183 | 0.515 |
| islr2 | 0.515 |
| d7mit326 | 0.514 |
| celsr1 | 0.513 |
| e130309f12rik | 0.513 |
| sorcs2 | 0.513 |
| zfp40 | 0.513 |
| myg1 | 0.513 |
| defb50 | 0.513 |
| hmx2 | 0.513 |
| rhox6 | 0.512 |
| nxph1 | 0.512 |
| luzp1 | 0.512 |
| ensmusg00000079376 | 0.511 |
| umodl1 | 0.511 |
| 6430517e21rik | 0.51 |
| eg624866 | 0.51 |
| zfp51 | 0.51 |
| mns1 | 0.51 |
| wdr13 | 0.51 |
| emid1 | 0.509 |
| slfn5 | 0.509 |
| siah1-ps1 | 0.508 |
| siah1-ps2 | 0.508 |
| cphx | 0.508 |
| odz2 | 0.508 |
| mirn721 | 0.507 |
| nav1 | 0.507 |
| 1500001a10rik | 0.507 |
| cdh7 | 0.507 |
| nrn1l | 0.507 |
| csrnp2 | 0.507 |
| nr2e1 | 0.507 |
| kbtbd7 | 0.507 |
| 4933436h12rik | 0.507 |
| 2410018m08rik | 0.507 |
| mirn202 | 0.506 |
| mirn137 | 0.506 |
| mirn503 | 0.506 |
| mirn674 | 0.506 |
| mirn31 | 0.505 |
| rimbp3 | 0.505 |
| slc46a3 | 0.505 |
| zfp358 | 0.505 |
| mirn96 | 0.504 |
| fezf2 | 0.504 |
| foxd2 | 0.504 |
| tdrd5 | 0.503 |
| tcfap2e | 0.503 |
| hmx1 | 0.503 |
| foxn4 | 0.503 |
| bc018242 | 0.503 |
| 9130005n14rik | 0.502 |
| dmrtc2 | 0.502 |
| crip3 | 0.502 |
| ubap2 | 0.502 |
| d16ertd472e | 0.502 |
| gtsf1 | 0.501 |
| rbm45 | 0.501 |
| fstl4 | 0.501 |
| ovol3 | 0.5 |
| ube2b-rs1 | 0.5 |
| rhox4b | 0.5 |
| 1110032e23rik | 0.5 |
| olfr157 | 0.499 |
| d15mit40 | 0.499 |
| glp1 | 0.499 |
| nell2 | 0.499 |
| rn18s | 0.499 |
| ccdc115 | 0.498 |
| d17mit181 | 0.498 |
| loc672284 | 0.498 |
| iapls3-10 | 0.497 |
| d11mit139 | 0.497 |
| lrrn2 | 0.497 |
| bhlhb5 | 0.497 |
| d15mit198 | 0.496 |
| rn18s-rs4 | 0.496 |
| barhl1 | 0.496 |
| tshz2 | 0.496 |
| sdk2 | 0.496 |
| sui1-rs3 | 0.496 |
| sui1-rs2 | 0.496 |
| sui1-rs4 | 0.496 |
| zfp191 | 0.496 |
| rnf112 | 0.495 |
| zscan10 | 0.495 |
| gatad1 | 0.495 |
| tw | 0.495 |
| zfp37 | 0.494 |
| 1110017d15rik | 0.494 |
| v1ra5 | 0.494 |
| dpy19l4 | 0.494 |
| pcp | 0.494 |
| pcdh1 | 0.494 |
| oc90 | 0.493 |
| ttc3 | 0.493 |
| lrrtm1 | 0.493 |
| tmem16c | 0.493 |
| mak10 | 0.493 |
| foxp4 | 0.493 |
| ifitm5 | 0.493 |
| scrt2 | 0.493 |
| ncdn | 0.493 |
| fign | 0.492 |
| zfp27 | 0.492 |
| zfhx2 | 0.492 |
| sebox | 0.492 |
| 1810053b01rik | 0.491 |
| smarcad1 | 0.491 |
| d6mit252 | 0.491 |
| gpr85 | 0.491 |
| cdadc1 | 0.491 |
| iapt15 | 0.491 |
| l4rn2 | 0.49 |
| gm705 | 0.49 |
| bc003993 | 0.49 |
| esx1 | 0.489 |
| olfr1359 | 0.489 |
| dscam | 0.489 |
| pum2 | 0.488 |
| zfp322a | 0.488 |
| crim1 | 0.488 |
| lrrc10 | 0.488 |
| mybl1 | 0.487 |
| dzip1 | 0.487 |
| armcx2 | 0.487 |
| dmrt2 | 0.487 |
| ncam2 | 0.487 |
| nkx1-2 | 0.487 |
| rn18s-rs3 | 0.486 |
| arid3c | 0.486 |
| emilin3 | 0.486 |
| bsx | 0.485 |
| 1810007m14rik | 0.485 |
| 4932438h23rik | 0.485 |
| agbl1 | 0.485 |
| dkkl1 | 0.485 |
| cux2 | 0.485 |
| commd3 | 0.485 |
| ovol1 | 0.485 |
| ay339874 | 0.484 |
| c81412 | 0.484 |
| hmx3 | 0.484 |
| midn | 0.484 |
| pcdhb1 | 0.484 |
| pcdhb4 | 0.484 |
| nav3 | 0.484 |
| rfxdc2 | 0.484 |
| gpr161 | 0.484 |
| usp42 | 0.484 |
| mirn181a-1 | 0.483 |
| mirn181a-2 | 0.483 |
| v2r9 | 0.483 |
| pou5f2 | 0.483 |
| yb1a | 0.483 |
| plac9 | 0.483 |
| popdc3 | 0.482 |
| nkx6-2 | 0.482 |
| mageb4 | 0.482 |
| gtig1 | 0.482 |
| mirn24-2 | 0.482 |
| d8mit98 | 0.482 |
| d8mit297 | 0.482 |
| 4933411k20rik | 0.482 |
| pcdh20 | 0.481 |
| zscan2 | 0.481 |
| asxl3 | 0.481 |
| foxd4 | 0.481 |
| pum1 | 0.481 |
| zic1 | 0.481 |
| myt1l | 0.481 |
| grhl3 | 0.48 |
| spata6 | 0.48 |
| pou6f2 | 0.48 |
| rnu1a2 | 0.48 |
| zfp354b | 0.48 |
| tgif2lx | 0.48 |
| dmrtb1 | 0.48 |
| dpf1 | 0.48 |
| ribc2 | 0.48 |
| onecut3 | 0.48 |
| hdgfrp3 | 0.479 |
| mrpl40 | 0.479 |
| foxf2 | 0.479 |
| prr16 | 0.479 |
| popdc2 | 0.479 |
| gm1673 | 0.479 |
| 1700029j07rik | 0.479 |
| py | 0.479 |
| gabpb2 | 0.478 |
| lhx5 | 0.478 |
| 8030494b02rik | 0.478 |
| mospd2 | 0.478 |
| mospd1 | 0.478 |
| bahcc1 | 0.478 |
| 9630031f12rik | 0.478 |
| mpzl2 | 0.478 |
| dscaml1 | 0.478 |
| mirn652 | 0.478 |
| mirn410 | 0.478 |
| mirn500 | 0.478 |
| cdh8 | 0.477 |
| oas1c | 0.477 |
| slitrk6 | 0.477 |
| lamr2 | 0.477 |
| lgl | 0.477 |
| tshz1 | 0.477 |
| pabpnl1 | 0.477 |
| fbxl14 | 0.476 |
| odz3 | 0.476 |
| 2410018c17rik | 0.476 |
| olfr156 | 0.476 |
| olfr155 | 0.476 |
| fank1 | 0.476 |
| foxl2os | 0.475 |
| agbl3 | 0.475 |
| irx3 | 0.475 |
| zfp352 | 0.475 |
| cobl | 0.475 |
| d2mit434 | 0.475 |
| hoxc | 0.475 |
| fbxo15 | 0.475 |
| tg(lef1)1efu | 0.474 |
| shisa2 | 0.474 |
| tshz3 | 0.474 |
| d4mit74 | 0.474 |
| 1600029d21rik | 0.474 |
| vax2 | 0.474 |
| thumpd3 | 0.474 |
| aard | 0.474 |
| nav2 | 0.474 |
| 1700011f03rik | 0.474 |
| lrrc8a | 0.474 |
| astn1 | 0.474 |
| fltn | 0.473 |
| nope | 0.473 |
| tulp3 | 0.473 |
| rb(1.18)10rma | 0.473 |
| d8mit138 | 0.473 |
| rb(16.17)8lub | 0.473 |
| rb(16.17)8lub | 0.473 |
| otor | 0.473 |
| kirrel3 | 0.473 |
| zar1 | 0.473 |
| satb2 | 0.473 |
| otp | 0.473 |
| arhgef5 | 0.472 |
| wal | 0.472 |
| cyl | 0.472 |
| tyro3-rs1 | 0.472 |
| heg1 | 0.472 |
| zbtb20 | 0.472 |
| morc1 | 0.472 |
| 1700008g05rik | 0.471 |
| egfl6 | 0.471 |
| nxph3 | 0.471 |
| ottmusg00000016644 | 0.471 |
| cdh22 | 0.471 |
| hil1 | 0.471 |
| dppa5-ps7 | 0.471 |
| dppa5-ps5 | 0.471 |
| dppa5-ps3 | 0.471 |
| dppa5-ps2 | 0.471 |
| dppa5-ps6 | 0.471 |
| dppa5-ps4 | 0.471 |
| dppa5-ps1 | 0.471 |
| c2cd3 | 0.47 |
| mdga1 | 0.47 |
| brunol6 | 0.47 |
| neurod2 | 0.47 |
| bex2 | 0.47 |
| adnp2 | 0.47 |
| krtap13 | 0.47 |
| olfr73 | 0.47 |
| drr1 | 0.47 |
| drr2 | 0.47 |
| ss18l1 | 0.47 |
| spata22 | 0.47 |
| d16mit108 | 0.47 |
| zfp422 | 0.47 |
| ay223547 | 0.47 |
| zc3h8 | 0.47 |
| fem1c | 0.469 |
| lmx1a | 0.469 |
| sp6 | 0.469 |
| zfp28 | 0.469 |
| sall3 | 0.469 |
| grhl1 | 0.469 |
| sh3bgrl | 0.469 |
| klk1b27 | 0.469 |
| d5mit62 | 0.469 |
| 9530002b09rik | 0.469 |
| tmem57 | 0.469 |
| zfp35 | 0.469 |
| in(10)7h | 0.469 |
| olfm1 | 0.468 |
| lrfn5 | 0.468 |
| hydin | 0.468 |
| vax1 | 0.468 |
| npas4 | 0.468 |
| zfp97 | 0.468 |
| dchs2 | 0.468 |
| cobll1 | 0.468 |
| a630095e13rik | 0.468 |
| spire2 | 0.467 |
| acsm4 | 0.467 |
| ntng2 | 0.467 |
| dxggc1e | 0.467 |
| tex19.1 | 0.467 |
| eg653016 | 0.467 |
| shox2 | 0.467 |
| zfp94 | 0.466 |
| gpr149 | 0.466 |
| ooep | 0.466 |
| tmem121 | 0.466 |
| lsamp | 0.466 |
| b020018g12rik | 0.466 |
| b160f9t | 0.466 |
| b437a17t | 0.466 |
| y301c7l | 0.466 |
| rgmb | 0.466 |
| 1700047i17rik1 | 0.466 |
| cnih | 0.466 |
| bc017158 | 0.466 |
| brwd1 | 0.466 |
| ecd | 0.466 |
| phf7 | 0.466 |
| pgia6 | 0.466 |
| gramd1a | 0.466 |
| irx2 | 0.465 |
| mirn93 | 0.465 |
| npas1 | 0.465 |
| fndc3b | 0.465 |
| olfr7 | 0.465 |
| t(5;12)31h | 0.465 |
| tbx15 | 0.465 |
| npn2 | 0.465 |
| mpmv4 | 0.465 |
| gsx1 | 0.465 |
| foxn2 | 0.464 |
| mmrn2 | 0.464 |
| d16mit59 | 0.464 |
| pmv42 | 0.464 |
| at | 0.464 |
| itm2c | 0.464 |
| 1110032a03rik | 0.464 |
| wiz | 0.464 |
| rpl24 | 0.464 |
| etl4 | 0.464 |
| cpu3 | 0.463 |
| cend1 | 0.463 |
| ubap2l | 0.463 |
| 2410004a20rik | 0.463 |
| smyd1 | 0.463 |
| odz4 | 0.463 |
| nrn1 | 0.463 |
| emid2 | 0.463 |
| serpinb6d | 0.463 |
| ottmusg00000000720 | 0.463 |
| serpinb6c | 0.463 |
| gsdmd | 0.462 |
| zfp640 | 0.462 |
| nkx2-3 | 0.462 |
| lrrc8c | 0.462 |
| padi6 | 0.462 |
| olfr11 | 0.462 |
| meig1 | 0.462 |
| emb | 0.462 |
| eml5 | 0.462 |
| phc3 | 0.461 |
| tsc22d4 | 0.461 |
| mltr4 | 0.461 |
| bc004044 | 0.461 |
| pcdha@ | 0.461 |
| lhx4 | 0.461 |
| ctxn1 | 0.461 |
| foxe3 | 0.461 |
| prdm10 | 0.461 |
| cni-rs2 | 0.461 |
| ror1 | 0.461 |
| lcn8 | 0.461 |
| tmem131 | 0.46 |
| olfr1507 | 0.46 |
| nrsn1 | 0.46 |
| tspyl-ps | 0.46 |
| ppfibp2 | 0.46 |
| flrt3 | 0.46 |
| osr2 | 0.46 |
| chd2 | 0.459 |
| axud1 | 0.459 |
| ctla2a | 0.459 |
| cbln4 | 0.459 |
| gdf7 | 0.459 |
| zkscan6 | 0.458 |
| pcp4 | 0.458 |
| 4833424o15rik | 0.458 |
| cecr2 | 0.458 |
| msx3 | 0.458 |
| kndc1 | 0.458 |
| zfp125 | 0.458 |
| zfp62 | 0.458 |
| chpst | 0.458 |
| lrit1 | 0.458 |
| tcf25 | 0.458 |
| hn1 | 0.458 |
| bc037156 | 0.458 |
| 9530020o07rik | 0.458 |
| gm1968 | 0.458 |
| d10mit148 | 0.458 |
| d16mit135 | 0.458 |
| zfp58 | 0.458 |
| iqch | 0.457 |
| mirn295 | 0.457 |
| d13sut1e | 0.457 |
| tex14 | 0.457 |
| cml3 | 0.457 |
| homez | 0.457 |
| sohlh2 | 0.457 |
| fez2 | 0.457 |
| scrt1 | 0.457 |
| ints1 | 0.457 |
| ptplb | 0.457 |
| grhl2 | 0.457 |
| wo | 0.456 |
| tekt1 | 0.456 |
| 0610037d15rik | 0.456 |
| gt10 | 0.456 |
| cst13 | 0.456 |
| cep63 | 0.456 |
| vmo1 | 0.456 |
| zfp423 | 0.456 |
| mirn130a | 0.456 |
| calr2 | 0.456 |
| 1700065i16rik | 0.456 |
| aqr | 0.456 |
| tdpoz1 | 0.455 |
| fndc3a | 0.455 |
| hapln4 | 0.455 |
| slfn8 | 0.455 |
| glis2 | 0.455 |
| neto1 | 0.455 |
| olfr151 | 0.454 |
| trim14 | 0.454 |
| palm2 | 0.454 |
| nrp | 0.454 |
| mettl9 | 0.454 |
| fezf1 | 0.454 |
| nobox | 0.454 |
| lcorl | 0.454 |
| six6 | 0.454 |
| mirn124a-2 | 0.454 |
| pbp2 | 0.454 |
| mynn | 0.454 |
| sp8 | 0.454 |
| il11ra2 | 0.454 |
| fbxw15 | 0.454 |
| nhlh1 | 0.453 |
| larp1 | 0.453 |
| fyco1 | 0.453 |
| osr1 | 0.453 |
| mylpf | 0.453 |
| zfhx4 | 0.453 |
| d6mit164 | 0.453 |
| sh3d2c-ps1 | 0.453 |
| tcea3 | 0.452 |
| bex4 | 0.452 |
| aym1 | 0.452 |
| sspo | 0.452 |
| adam4 | 0.452 |
| cntn3 | 0.452 |
| olfr1168 | 0.452 |
| olfr642 | 0.452 |
| olfr164 | 0.452 |
| olfr37 | 0.452 |
| mirn24-1 | 0.451 |
| eh | 0.451 |
| bves | 0.451 |
| cpxm1 | 0.451 |
| cst9 | 0.451 |
| hormad1 | 0.451 |
| pf | 0.451 |
| dmrt1 | 0.451 |
| ngrn | 0.451 |
| yy2 | 0.451 |
| pcdh8 | 0.451 |
| reep6 | 0.451 |
| bs | 0.451 |
| tbx20 | 0.451 |
| loc100040223 | 0.451 |
| rpl32-ps | 0.451 |
| d9mit263 | 0.451 |
| glis3 | 0.45 |
| sp5 | 0.45 |
| pcp2 | 0.45 |
| tg.ttr-1 | 0.45 |
| fbxl16 | 0.45 |
| 1110033m05rik | 0.45 |
| tg(krt2-9)1grog | 0.449 |
| tg(wnt3)7gsb | 0.449 |
| klk1b5 | 0.449 |
| fndc4 | 0.449 |
| loc100041323 | 0.449 |
| tbx10 | 0.449 |
| exma | 0.449 |
| mirn16-2 | 0.449 |
| rbm27 | 0.449 |
| ryk | 0.449 |
| solh | 0.449 |
| nxph2 | 0.449 |
| lyrm1 | 0.448 |
| phf16 | 0.448 |
| d6mm5e | 0.448 |
| xlr | 0.448 |
| cby | 0.448 |
| agbl2 | 0.448 |
| asxl2 | 0.448 |
| dach2 | 0.448 |
| utp3 | 0.448 |
| casz1 | 0.448 |
| prelid1 | 0.448 |
| ypel1 | 0.448 |
| olfr14 | 0.448 |
| olfr13 | 0.448 |
| olfr144 | 0.448 |
| olfr143 | 0.448 |
| irx1 | 0.447 |
| ssbp3 | 0.447 |
| dxertd573e | 0.447 |
| bex6 | 0.447 |
| zic4 | 0.447 |
| d9mit264 | 0.447 |
| mirn124a-1 | 0.447 |
| efhd1 | 0.447 |
| tagln3 | 0.447 |
| figla | 0.447 |
| zkscan5 | 0.447 |
| mif-ps3 | 0.447 |
| d7mit65 | 0.446 |
| helz | 0.446 |
| ottmusg00000004461 | 0.446 |
| mirn9-1 | 0.446 |
| sema5b | 0.446 |
| d8mit198 | 0.446 |
| eeld2 | 0.446 |
| eeld1 | 0.446 |
| rfesd | 0.446 |
| gtlf3a | 0.446 |
| gtlf3b | 0.446 |
| d17mit9 | 0.446 |
| dullard | 0.446 |
| zfp830 | 0.446 |
| v1ra1 | 0.446 |
| mrpl10 | 0.445 |
| cstl1 | 0.445 |
| lcn4 | 0.445 |
| zfp60 | 0.445 |
| sbk1 | 0.445 |
| 4933409k07rik | 0.445 |
| c030029h13rik | 0.445 |
| tgif2 | 0.445 |
| dxmit48 | 0.445 |
| pou6f1 | 0.445 |
| irx5 | 0.445 |
| klhl6 | 0.445 |
| cbln3 | 0.444 |
| ly6h | 0.444 |
| bmp8a | 0.444 |
| smu1 | 0.444 |
| tll1 | 0.444 |
| slc33a1 | 0.444 |
| zic2 | 0.444 |
| gm1110 | 0.444 |
| emilin2 | 0.444 |
| rfx3 | 0.444 |
| fndc5 | 0.444 |
| hopx | 0.444 |
| slfn10 | 0.444 |
| slc35c2 | 0.444 |
| d3mit250 | 0.444 |
| lxn | 0.443 |
| ltv1 | 0.443 |
| gpc6 | 0.443 |
| nkx6-3 | 0.443 |
| flrt2 | 0.443 |
| pcdhgb2 | 0.443 |
| pcdhgc5 | 0.443 |
| nenf | 0.443 |
| tekt3 | 0.443 |
| cbln2 | 0.443 |
| matn4 | 0.443 |
| aw548124 | 0.443 |
| lenep | 0.443 |
| dpf2 | 0.442 |
| mirn106a | 0.442 |
| d11mit51 | 0.442 |
| glis1 | 0.442 |
| sfmbt2 | 0.442 |
| epha5 | 0.442 |
| nhlh2 | 0.442 |
| 2010317e24rik | 0.442 |
| tsx | 0.442 |
| ph | 0.441 |
| bmp8b | 0.441 |
| zfp449 | 0.441 |
| olfr1508 | 0.441 |
| olfr1509 | 0.441 |
| eg546896 | 0.441 |
| omp | 0.441 |
| pdcl2 | 0.441 |
| spata20 | 0.441 |
| d9citb3 | 0.441 |
| dxmit52 | 0.441 |
| loc232077 | 0.441 |
| barhl2 | 0.441 |
| br | 0.441 |
| pmfbp1 | 0.441 |
| zfp354c | 0.44 |
| cdh6 | 0.44 |
| hnt | 0.44 |
| olfr160 | 0.44 |
| olt | 0.44 |
| eg621324 | 0.44 |
| ms15-7 | 0.44 |
| rmst | 0.44 |
| tg(bmp4)6blh | 0.44 |
| olfr17 | 0.44 |
| theg | 0.44 |
| rhox1 | 0.44 |
| lgr4 | 0.44 |
| spag4l | 0.44 |
| d5mit63 | 0.44 |
| ms15-3 | 0.44 |
| d17leh525 | 0.44 |
| sparcl1 | 0.439 |
| 3-Sep | 0.439 |
| tmem50b | 0.439 |
| ikzf2 | 0.439 |
| ptpla | 0.439 |
| tbx4 | 0.439 |
| wnt8b | 0.439 |
| arid5b | 0.439 |
| gprc5b | 0.439 |
| slitrk3 | 0.439 |
| slitrk5 | 0.439 |
| gpr97 | 0.439 |
| piwil4 | 0.439 |
| d11mit109 | 0.439 |
| d11mit205 | 0.439 |
| ottmusg00000003456 | 0.439 |
| ttll3 | 0.439 |
| wbscr17 | 0.439 |
| tlx2 | 0.439 |
| frem1 | 0.439 |
| 1700057k13rik | 0.439 |
| ptchd3 | 0.439 |
| loc100047997 | 0.439 |
| rnf6 | 0.439 |
| zfp353 | 0.439 |
| sgp2 | 0.438 |
| d19ertd386e | 0.438 |
| nkx2-4 | 0.438 |
| pcdhga12 | 0.438 |
| 1700016d06rik | 0.438 |
| mro | 0.438 |
| mpmv8 | 0.438 |
| 4931433a01rik | 0.438 |
| agbl5 | 0.438 |
| slitrk2 | 0.438 |
| slitrk4 | 0.438 |
| gm22 | 0.438 |
| cml1 | 0.438 |
| cml5 | 0.438 |
| alpk3 | 0.438 |
| morf4l1 | 0.437 |
| 1110004f10rik | 0.437 |
| d10mit181 | 0.437 |
| dtx4 | 0.437 |
| tead2 | 0.437 |
| asz1 | 0.437 |
| krtap14 | 0.437 |
| flrt1 | 0.437 |
| gtl3 | 0.437 |
| hop | 0.437 |
| fgf16 | 0.437 |
| bld | 0.437 |
| mtf2 | 0.437 |
| d4mit117 | 0.437 |
| etv2 | 0.437 |
| dbx1 | 0.437 |
| dxmit143 | 0.437 |
| frem2 | 0.437 |
| 4930467e23rik | 0.437 |
| thap11 | 0.437 |
| megf9 | 0.437 |
| gm52 | 0.437 |
| mirn205 | 0.436 |
| dnajb3 | 0.436 |
| tdpoz2 | 0.436 |
| tdpoz4 | 0.436 |
| tdpoz5 | 0.436 |
| loc100043188 | 0.436 |
| tdpoz3 | 0.436 |
| li | 0.436 |
| mirn16-1 | 0.436 |
| thcr | 0.436 |
| b230215l15rik | 0.436 |
| tox2 | 0.436 |
| dcpp1 | 0.436 |
| d11mit117 | 0.436 |
| gbx1 | 0.435 |
| cml2 | 0.435 |
| olfr281 | 0.435 |
| olfr282 | 0.435 |
| olfr124 | 0.435 |
| olfr168 | 0.435 |
| prl2c5 | 0.435 |
| pcnx | 0.435 |
| cdh10 | 0.435 |
| zfp418 | 0.435 |
| lgi2 | 0.435 |
| hoxc13 | 0.435 |
| tbx22 | 0.435 |
| d7mit56 | 0.435 |
| arid5a | 0.435 |
| spic | 0.435 |
| sox12 | 0.435 |
| mustn1 | 0.435 |
| svet1 | 0.435 |
| gas5 | 0.435 |
| zfp113 | 0.435 |
| dxmit57 | 0.434 |
| mtap2k | 0.434 |
| mirn124a-3 | 0.434 |
| foxd3 | 0.434 |
| c330019g07rik | 0.434 |
| zfp536 | 0.434 |
| phtf2 | 0.434 |
| olfr159 | 0.434 |
| olfr70 | 0.434 |
| adamts20 | 0.434 |
| rnu1b1 | 0.434 |
| mirn196a-1 | 0.434 |
| mirn196a-2 | 0.434 |
| atcay | 0.434 |
| rax | 0.434 |
| prickle2 | 0.434 |
| mirn200b | 0.433 |
| tor1b | 0.433 |
| atoh7 | 0.433 |
| 4933424b01rik | 0.433 |
| spsb4 | 0.433 |
| egfl7 | 0.433 |
| jarid2 | 0.433 |
| lrrc8d | 0.433 |
| lrrc8b | 0.433 |
| lrrc8e | 0.433 |
| d1mit172 | 0.433 |
| olfr16 | 0.433 |
| gpr139 | 0.433 |
| ncaph2 | 0.432 |
| pcdh17 | 0.432 |
| m-01983 | 0.432 |
| iapt12 | 0.432 |
| znrf1 | 0.432 |
| setd3 | 0.432 |
| zfp131 | 0.432 |
| irx4 | 0.432 |
| zscan12 | 0.432 |
| hoxb3os | 0.432 |
| gmcl1 | 0.432 |
| odz1 | 0.432 |
| zap1 | 0.432 |
| gprc5d | 0.432 |
| pou3f4 | 0.432 |
| nkx2-9 | 0.431 |
| noto | 0.431 |
| l2 | 0.431 |
| msx1as | 0.431 |
| mirn17 | 0.431 |
| gm414 | 0.431 |
| ccdc67 | 0.431 |
| c530043g21rik | 0.431 |
| mirn29b-2 | 0.431 |
| 2310037i24rik | 0.431 |
| dbx2 | 0.431 |
| gpc5 | 0.431 |
| pptcs2 | 0.431 |
| tera | 0.431 |
| pcdha4 | 0.431 |
| dnd1 | 0.43 |
| dppa3 | 0.43 |
| txndc2 | 0.43 |
| nkx2-6 | 0.43 |
| fbxw16 | 0.43 |
| zfp74 | 0.43 |
| gsdmc3 | 0.43 |
| gsdmc2 | 0.43 |
| gsdmc4 | 0.43 |
| tug1 | 0.43 |
| d15mit65 | 0.43 |
| dxmit139 | 0.43 |
| plxdc2 | 0.43 |
| fev | 0.43 |
| sema6c | 0.43 |
| mpmv2 | 0.43 |
| tex19.2 | 0.43 |
| hoxa11os | 0.429 |
| mirn23b | 0.429 |
| hmgb3 | 0.429 |
| hoxc11 | 0.429 |
| ankrd36 | 0.429 |
| rhbdl1 | 0.429 |
| avil | 0.429 |
| 6430411k18rik | 0.429 |
| piwil2 | 0.429 |
| spata5 | 0.429 |
| tch | 0.429 |
| mpzl3 | 0.429 |
| necab2 | 0.429 |
| rshl2b | 0.429 |
| zfp354a | 0.429 |
| bnc2 | 0.428 |
| klk1b9 | 0.428 |
| xlr2 | 0.428 |
| ylr | 0.428 |
| neurod6 | 0.428 |
| gpc2 | 0.428 |
| zic3 | 0.428 |
| sall2 | 0.428 |
| gdf10 | 0.428 |
| hsp70-ps1 | 0.428 |
| ascl4 | 0.428 |
| bex1 | 0.428 |
| scmh1 | 0.428 |
| olfr63 | 0.428 |
| spz1 | 0.428 |
| hist1h2ag | 0.428 |
| cpa6 | 0.428 |
| scube1 | 0.427 |
| ifrd2 | 0.427 |
| sez6 | 0.427 |
| 2810474o19rik | 0.427 |
| sh3bgr | 0.427 |
| xmv16 | 0.427 |
| zfp212 | 0.427 |
| cnih2 | 0.427 |
| alx1 | 0.427 |
| fat2 | 0.427 |
| usp22 | 0.427 |
| hoxd3os | 0.426 |
| tagln2 | 0.426 |
| wi-16786 | 0.426 |
| zfp219 | 0.426 |
| clmn | 0.426 |
| snai3 | 0.426 |
| tg(amy1c)9257mm | 0.426 |
| plxnd1 | 0.426 |
| d17mit7 | 0.426 |
| rnf166 | 0.426 |
| ifitm2 | 0.426 |
| nr6a1 | 0.426 |
| d5nds2 | 0.426 |
| 5430433g21rik | 0.426 |
| klk1b21 | 0.426 |
| mnm | 0.426 |
| gcm1 | 0.426 |
| zfp207 | 0.426 |
| sox21 | 0.426 |
| lim2 | 0.426 |
| spock1 | 0.426 |
| ceacam10 | 0.426 |
| man2a2 | 0.426 |
| in(10)17rk-p | 0.425 |
