# Supplementary material for: GIT2 Acts as a Potential Keystone Protein in Functional Hypothalamic Networks Associated with Age-Related Phenotypic Changes in Rats
Source: PLoS One. 2012 May 14;7(5):e36975. doi: 10.1371/journal.pone.0036975 (PMC3351446; doi:10.1371/journal.pone.0036975)
Supplement: Table S23 — GeneIndexer latent semantic indexing (LSI) of significantly-regulated ‘Cyclin-dependent protein kinase inhibitor activity’ GO term group. Using the GO term group ‘Cyclin-dependent protein kinase inhibitor activity’ as an input term, a list of the top 1000 implicitly-correlated (LSI correlation score >0.1) was generated using a full genome background list. (DOC) [file pone.0036975.s027.doc]

**Table S23. GeneIndexer latent semantic indexing (LSI) of significantly-regulated ‘Cyclin-dependent protein kinase inhibitor activity’ GO term group.** Using the GO term group ‘Cyclin-dependent protein kinase inhibitor activity’ as an input term, a list of the top 1000 implicitly-correlated (LSI correlation score >0.1) was generated using a full genome background list.

| ***Cyclin-dependent protein kinase inhibitor activity*** |  |
| --- | --- |
|  |  |
| **Protein Symbol** | **LSI correlation score** |
| cdk3 | 0.679 |
| edk | 0.677 |
| ccrk | 0.666 |
| wee2 | 0.652 |
| mobkl1a | 0.626 |
| nek4 | 0.626 |
| pftk2 | 0.625 |
| cdkn3 | 0.624 |
| spdya | 0.616 |
| scaper | 0.615 |
| stk38l | 0.612 |
| cdk10 | 0.608 |
| cdc2l5 | 0.607 |
| pskh1 | 0.599 |
| ppp1r1c | 0.599 |
| stk24 | 0.596 |
| nek11 | 0.589 |
| dyrk1b | 0.586 |
| nek5 | 0.586 |
| ak5 | 0.585 |
| tob2 | 0.585 |
| dyrk1c | 0.583 |
| ripk5 | 0.583 |
| rbks | 0.574 |
| cdc2l6 | 0.574 |
| pkmyt1 | 0.574 |
| camk2n1 | 0.573 |
| ick | 0.572 |
| uhmk1 | 0.571 |
| camkv | 0.568 |
| spdyb | 0.567 |
| ysk4 | 0.567 |
| crkrs | 0.567 |
| pick2 | 0.565 |
| pick5 | 0.565 |
| pick3 | 0.565 |
| pick4 | 0.565 |
| rcsd1 | 0.564 |
| cdkl1 | 0.563 |
| mobkl1b | 0.563 |
| sbk1 | 0.561 |
| pctk3 | 0.56 |
| ubac1 | 0.56 |
| pftk1 | 0.558 |
| tor | 0.558 |
| ccne2 | 0.558 |
| tssk3 | 0.557 |
| cdc2b | 0.556 |
| hisppd1 | 0.556 |
| ccnc | 0.555 |
| dyrk2 | 0.551 |
| ppp1r14d | 0.55 |
| a630047e20rik | 0.549 |
| cdk7 | 0.548 |
| nek9 | 0.545 |
| cdk8 | 0.545 |
| sfrs16 | 0.544 |
| ctps2 | 0.544 |
| pctk2 | 0.54 |
| sav1 | 0.54 |
| mnat1 | 0.539 |
| ccnk | 0.539 |
| wdr6 | 0.538 |
| riok2 | 0.538 |
| cmpk1 | 0.537 |
| 3930401k13rik | 0.536 |
| 2610018g03rik | 0.535 |
| cpne3 | 0.535 |
| cdk5rap1 | 0.534 |
| stk38 | 0.533 |
| nrk | 0.533 |
| ilkap | 0.533 |
| nme6 | 0.532 |
| pctk1 | 0.532 |
| mapk6 | 0.531 |
| tnik | 0.531 |
| nek6 | 0.531 |
| melk | 0.531 |
| ccnf | 0.529 |
| camk2n2 | 0.529 |
| fbxo7 | 0.526 |
| afap1l2 | 0.525 |
| rnf123 | 0.524 |
| taok1 | 0.522 |
| trp53rk | 0.521 |
| mapk4 | 0.52 |
| mast4 | 0.52 |
| t(11;19)42h | 0.52 |
| vrk1 | 0.52 |
| cdk5rap3 | 0.519 |
| csnk1g1 | 0.519 |
| ppm1b | 0.518 |
| d2mit316 | 0.517 |
| ms4a3 | 0.517 |
| cdca3 | 0.516 |
| ppm1e | 0.516 |
| c230081a13rik | 0.516 |
| cdc2l1 | 0.515 |
| tlk2 | 0.515 |
| itpk1 | 0.514 |
| cmpk2 | 0.514 |
| gmfg | 0.512 |
| mark3 | 0.511 |
| stk3 | 0.51 |
| ppme1 | 0.507 |
| mapk15 | 0.506 |
| ccnl1 | 0.506 |
| asb15 | 0.506 |
| zfp622 | 0.506 |
| ppm1a | 0.506 |
| hexim2 | 0.506 |
| ccnl2 | 0.506 |
| wee1 | 0.505 |
| pbk | 0.504 |
| eef2k | 0.504 |
| dusp26 | 0.504 |
| cdkl3 | 0.504 |
| sh3bp5 | 0.503 |
| akap8 | 0.503 |
| usp19 | 0.503 |
| ppp1r14b | 0.503 |
| srpk2 | 0.502 |
| sertad1 | 0.501 |
| dtymk | 0.501 |
| rell1 | 0.501 |
| rell2 | 0.501 |
| phlppl | 0.5 |
| b230120h23rik | 0.499 |
| csnk1g3 | 0.499 |
| ccni | 0.499 |
| tesk1 | 0.499 |
| cdc7 | 0.498 |
| zc3hc1 | 0.498 |
| slk | 0.496 |
| mizf | 0.496 |
| cks1b | 0.495 |
| ptpdc1 | 0.495 |
| dusp23 | 0.495 |
| 2810452k22rik | 0.495 |
| nudt3 | 0.495 |
| taok2 | 0.494 |
| tesk2 | 0.494 |
| them4 | 0.493 |
| stk16 | 0.493 |
| dusp3 | 0.493 |
| ttbk1 | 0.493 |
| riok1 | 0.492 |
| phlpp | 0.492 |
| uck1 | 0.49 |
| pim3 | 0.489 |
| cnot6l | 0.489 |
| dusp18 | 0.488 |
| cables1 | 0.487 |
| 9130404d14rik | 0.487 |
| ctps | 0.487 |
| ppp2r5d | 0.486 |
| nek3 | 0.486 |
| map3k9 | 0.485 |
| mark4 | 0.485 |
| 1700026l06rik | 0.485 |
| ccng2 | 0.485 |
| cdc5l | 0.485 |
| ppm1j | 0.485 |
| tssk4 | 0.484 |
| clk2 | 0.483 |
| cdkn2d | 0.482 |
| ccnb3 | 0.481 |
| fuk | 0.481 |
| stk10 | 0.48 |
| cks2 | 0.48 |
| aatk | 0.479 |
| ppp2r3a | 0.479 |
| ipmk | 0.479 |
| npat | 0.479 |
| vrk3 | 0.479 |
| ai842396 | 0.478 |
| dolk | 0.478 |
| nek7 | 0.478 |
| ihpk1 | 0.478 |
| dbf4 | 0.478 |
| galk2 | 0.477 |
| alpk2 | 0.477 |
| pkn2 | 0.477 |
| dusp2 | 0.476 |
| bc030867 | 0.476 |
| glt8d3 | 0.476 |
| prpf4b | 0.476 |
| syf2 | 0.475 |
| 2700078k21rik | 0.475 |
| phpt1 | 0.475 |
| cdk5rap2 | 0.475 |
| cdc14a | 0.475 |
| cdk2ap2 | 0.474 |
| umpk-ps | 0.473 |
| npdc1 | 0.473 |
| dusp11 | 0.473 |
| ppp2r5b | 0.472 |
| prkrir | 0.472 |
| mapkapk3 | 0.472 |
| brsk1 | 0.471 |
| micalcl | 0.471 |
| ctdspl | 0.47 |
| agk | 0.469 |
| ppm1f | 0.469 |
| clk1 | 0.469 |
| wnk2 | 0.469 |
| dgkk | 0.469 |
| cdk6 | 0.468 |
| spred3 | 0.468 |
| snrk | 0.468 |
| ccpg1 | 0.468 |
| ppp1r2 | 0.467 |
| mak | 0.467 |
| nme7 | 0.467 |
| pdik1l | 0.466 |
| map3k13 | 0.466 |
| pkia | 0.466 |
| cdk5r2 | 0.466 |
| dusp19 | 0.466 |
| ulk2 | 0.466 |
| ccnt2 | 0.465 |
| ppm1g | 0.465 |
| ppp2r2c | 0.465 |
| wdr68 | 0.465 |
| 1110008f13rik | 0.465 |
| plekhm3 | 0.465 |
| pkn3 | 0.464 |
| ccny | 0.464 |
| tob1 | 0.464 |
| mrpl20 | 0.464 |
| dusp4 | 0.464 |
| znhit1 | 0.462 |
| 1110007c09rik | 0.462 |
| brsk2 | 0.462 |
| tiprl | 0.462 |
| pstk | 0.461 |
| ckt2 | 0.461 |
| nagk | 0.461 |
| cops6 | 0.461 |
| pea15b | 0.461 |
| zcchc8 | 0.46 |
| dusp22 | 0.46 |
| clk3 | 0.46 |
| dusp16 | 0.46 |
| vrk2 | 0.46 |
| nuak1 | 0.459 |
| hisppd2a | 0.459 |
| prkd3 | 0.458 |
| bc010304 | 0.458 |
| ccnh | 0.458 |
| d1mit508 | 0.458 |
| clk4 | 0.458 |
| stk25 | 0.458 |
| d11mit109 | 0.458 |
| d11mit205 | 0.458 |
| bc033915 | 0.457 |
| cdc37 | 0.457 |
| rex2 | 0.457 |
| mobkl3 | 0.456 |
| map4k3 | 0.456 |
| cdc14b | 0.455 |
| bc032265 | 0.455 |
| gps1 | 0.455 |
| zfp641 | 0.455 |
| gkap1 | 0.455 |
| ccna1 | 0.454 |
| stk40 | 0.454 |
| lats1 | 0.454 |
| mlkl | 0.454 |
| mknk2 | 0.454 |
| cdkl2 | 0.454 |
| lmtk3 | 0.452 |
| tssk1 | 0.451 |
| sdpr | 0.45 |
| taok3 | 0.45 |
| dak | 0.45 |
| ptpmt1 | 0.449 |
| ppp1r14c | 0.449 |
| ly6g6f | 0.449 |
| cnot2 | 0.448 |
| dusp7 | 0.447 |
| ink76 | 0.447 |
| plk2 | 0.447 |
| dusp5 | 0.447 |
| stk4 | 0.447 |
| cdc34 | 0.446 |
| nadk | 0.446 |
| shc4 | 0.446 |
| ppp6c | 0.446 |
| carhsp1 | 0.446 |
| stard10 | 0.446 |
| zmym6 | 0.445 |
| ryk-rs1 | 0.445 |
| ppp2r4 | 0.445 |
| dgkg | 0.445 |
| rn7sk | 0.445 |
| mapkapk5 | 0.445 |
| rngtt | 0.444 |
| itfg1 | 0.444 |
| cdc42bpg | 0.443 |
| ccnj | 0.443 |
| ottmusg00000022410 | 0.443 |
| ksr2 | 0.443 |
| cep170 | 0.442 |
| prkd2 | 0.441 |
| zfp46 | 0.441 |
| mapk12 | 0.441 |
| dapk3 | 0.441 |
| cdc25b | 0.44 |
| dyrk3 | 0.44 |
| shf | 0.44 |
| plk3 | 0.439 |
| papolg | 0.439 |
| cdca2 | 0.438 |
| zfp383 | 0.438 |
| myt1 | 0.438 |
| ptprr | 0.438 |
| hist1h1b | 0.438 |
| riok3 | 0.437 |
| rbmxrt | 0.437 |
| plk-ps1 | 0.437 |
| dctn6 | 0.437 |
| zfand6 | 0.436 |
| d1mit58 | 0.436 |
| uck2 | 0.436 |
| pkn1 | 0.435 |
| ccng1 | 0.435 |
| 1190002h23rik | 0.435 |
| akap11 | 0.435 |
| tnk1 | 0.434 |
| cnpy2 | 0.434 |
| cad | 0.434 |
| ihpk2 | 0.434 |
| mybl2 | 0.433 |
| map2k1ip1 | 0.433 |
| nuak2 | 0.432 |
| cdc25a | 0.432 |
| pkib | 0.432 |
| csnk1g2 | 0.432 |
| ppm1h | 0.432 |
| gcn1l1 | 0.432 |
| snf1lk | 0.432 |
| dok3 | 0.431 |
| dyrk4 | 0.431 |
| eif3f | 0.431 |
| ublcp1 | 0.431 |
| cdc6 | 0.43 |
| twf2 | 0.43 |
| loc667882 | 0.43 |
| tspan15 | 0.43 |
| slfn1 | 0.429 |
| ripk4 | 0.429 |
| plk4 | 0.429 |
| als2cr2 | 0.428 |
| styk1 | 0.428 |
| ppp1r14a | 0.428 |
| ctdsp2 | 0.428 |
| sec14l4 | 0.428 |
| arpp21 | 0.428 |
| ebna1bp2 | 0.428 |
| map3k12 | 0.427 |
| ube2r2 | 0.427 |
| pkig | 0.427 |
| gsbs | 0.427 |
| 1810043h04rik | 0.427 |
| pip4k2c | 0.426 |
| iapls3-28 | 0.426 |
| hexim1 | 0.426 |
| ptchd2 | 0.426 |
| mtmr15 | 0.426 |
| 0610007c21rik | 0.426 |
| plekho1 | 0.426 |
| cdc23 | 0.426 |
| inpp5a | 0.426 |
| ai462493 | 0.425 |
| guk1 | 0.425 |
| cops4 | 0.425 |
| ppp1r8 | 0.425 |
| pip4k2b | 0.425 |
| anapc5 | 0.424 |
| wdr26 | 0.424 |
| mcm4 | 0.424 |
| dok4 | 0.424 |
| limk2 | 0.424 |
| ylpm1 | 0.423 |
| psmd9 | 0.423 |
| zfp692 | 0.423 |
| srpk1 | 0.423 |
| znhit4 | 0.423 |
| thtpa | 0.423 |
| pip5kl1 | 0.423 |
| shoc2 | 0.423 |
| rnf7 | 0.423 |
| sh3rf1 | 0.423 |
| fbxo5 | 0.423 |
| dgkq | 0.423 |
| anks1 | 0.423 |
| cdc27 | 0.423 |
| centg1 | 0.422 |
| mtap2k | 0.422 |
| st5 | 0.422 |
| ppp1r16a | 0.422 |
| dusp9 | 0.422 |
| ppm1k | 0.422 |
| bckdk | 0.421 |
| pak1ip1 | 0.421 |
| lin9 | 0.42 |
| anapc4 | 0.42 |
| ccdc134 | 0.42 |
| 6720463m24rik | 0.42 |
| smok2a | 0.42 |
| ccdc19 | 0.42 |
| supt5h | 0.42 |
| ppp4c | 0.419 |
| styxl1 | 0.419 |
| fbxo4 | 0.419 |
| 4632434i11rik | 0.419 |
| tspyl2 | 0.419 |
| cnot3 | 0.419 |
| dbndd2 | 0.419 |
| pola2 | 0.419 |
| ptpn23 | 0.419 |
| akap6 | 0.419 |
| dusp15 | 0.418 |
| mtmr6 | 0.418 |
| cct7 | 0.418 |
| cct2 | 0.418 |
| phip | 0.418 |
| ctdp1 | 0.418 |
| fzr1 | 0.418 |
| fbxo43 | 0.417 |
| mast2 | 0.417 |
| cdt1 | 0.417 |
| zdhhc16 | 0.416 |
| nme3 | 0.416 |
| dgkb | 0.416 |
| nrbp1 | 0.416 |
| gadd45gip1 | 0.416 |
| dusp14 | 0.416 |
| dgkd | 0.416 |
| med21 | 0.415 |
| ptpn7 | 0.415 |
| 2610207i05rik | 0.415 |
| lrrc4 | 0.415 |
| loc436194 | 0.415 |
| mark1 | 0.415 |
| hipk4 | 0.415 |
| rqcd1 | 0.415 |
| d1mit316 | 0.414 |
| chchd3 | 0.414 |
| ppapdc2 | 0.414 |
| mirn221 | 0.414 |
| mirn222 | 0.414 |
| srms | 0.413 |
| dusp10 | 0.413 |
| dub2 | 0.413 |
| nucks1 | 0.413 |
| caprin1 | 0.413 |
| lass5 | 0.413 |
| dtd1 | 0.412 |
| nlk | 0.412 |
| tmprss13 | 0.412 |
| cdk9 | 0.412 |
| mycbp | 0.412 |
| cdk2ap1 | 0.412 |
| 4833426j09rik | 0.412 |
| ttk | 0.412 |
| ciz1 | 0.412 |
| mdfic | 0.412 |
| ppp2r5e | 0.411 |
| tssk2 | 0.411 |
| ctdsp1 | 0.411 |
| gmfb | 0.411 |
| arpp19 | 0.411 |
| ptpn21 | 0.41 |
| hint1 | 0.41 |
| stk22s1 | 0.409 |
| ccndbp1 | 0.409 |
| ppp4r1 | 0.409 |
| cops8 | 0.409 |
| map4k2 | 0.409 |
| tppp | 0.409 |
| trerf1 | 0.409 |
| cdkn2c | 0.409 |
| sphkap | 0.409 |
| dmtf1 | 0.409 |
| eef2 | 0.408 |
| rasa3 | 0.408 |
| etnk1 | 0.408 |
| eef1g | 0.408 |
| stk35 | 0.407 |
| orc1l | 0.407 |
| cerk | 0.407 |
| ptpn18 | 0.407 |
| fbxo31 | 0.407 |
| mapkap1 | 0.407 |
| ryk-ps1 | 0.406 |
| depdc6 | 0.406 |
| tsen2 | 0.406 |
| spin1 | 0.406 |
| pdap1 | 0.406 |
| rp9 | 0.405 |
| atf7 | 0.405 |
| cab39 | 0.405 |
| akap8l | 0.405 |
| glrx3 | 0.405 |
| usp6nl | 0.405 |
| map3k6 | 0.405 |
| pak7 | 0.405 |
| ppm1m | 0.405 |
| gm944 | 0.405 |
| gmnn | 0.404 |
| e130304f04rik | 0.404 |
| pitpnb | 0.404 |
| clp1 | 0.404 |
| itpka | 0.404 |
| erf | 0.404 |
| nkap | 0.404 |
| c330002i19rik | 0.403 |
| ptk6 | 0.403 |
| btbd10 | 0.403 |
| ppapdc1 | 0.403 |
| ppp2r5a | 0.403 |
| rrn3 | 0.402 |
| aurkaip1 | 0.402 |
| pi4k2b | 0.402 |
| elp2 | 0.402 |
| pak6 | 0.402 |
| frat2 | 0.402 |
| frk | 0.402 |
| cdc25c | 0.402 |
| tlk1 | 0.402 |
| bzw1 | 0.402 |
| mknk1 | 0.402 |
| fkbpl | 0.401 |
| ube2q2 | 0.401 |
| cspp1 | 0.401 |
| orc2l | 0.401 |
| ptpn14 | 0.401 |
| prkrip1 | 0.401 |
| rnd3 | 0.4 |
| tfpt | 0.4 |
| itpkc | 0.4 |
| rb1cc1 | 0.4 |
| d0wfb1e | 0.4 |
| shd | 0.399 |
| she | 0.399 |
| pask | 0.399 |
| dgka | 0.399 |
| rufy2 | 0.399 |
| gltscr2 | 0.399 |
| cdc42bpa | 0.399 |
| pcbd2 | 0.398 |
| akt1s1 | 0.398 |
| prkx | 0.398 |
| ptprh | 0.398 |
| phkg1 | 0.398 |
| alpk1 | 0.398 |
| cops7a | 0.398 |
| centg3 | 0.398 |
| ppp5c | 0.397 |
| matk | 0.397 |
| ppp1r10 | 0.397 |
| uhrf2 | 0.397 |
| lrrn3 | 0.397 |
| cdc45l | 0.397 |
| mast3 | 0.397 |
| d11mit208 | 0.397 |
| ccnb2 | 0.397 |
| dus2l | 0.397 |
| dusp13 | 0.396 |
| tcea3 | 0.396 |
| mast1 | 0.396 |
| map3k10 | 0.396 |
| lats2 | 0.396 |
| cib2 | 0.396 |
| zfp655 | 0.396 |
| ppp2r2a | 0.396 |
| cnot8 | 0.395 |
| jdp2 | 0.395 |
| dapp1 | 0.395 |
| rps6kc1 | 0.395 |
| pik3c2a | 0.395 |
| 2610019a05rik | 0.395 |
| d2ertd391e | 0.395 |
| trim41 | 0.395 |
| pde7b | 0.395 |
| ppp1r7 | 0.395 |
| slfn2 | 0.395 |
| ptpn3 | 0.394 |
| degs1 | 0.394 |
| ppp1r12c | 0.394 |
| dusp6 | 0.394 |
| lmtk2 | 0.394 |
| pdcl | 0.394 |
| 6330569m22rik | 0.394 |
| d330017j20rik | 0.394 |
| eif3eip | 0.394 |
| phkg2 | 0.394 |
| clybl | 0.394 |
| dusp12 | 0.394 |
| ppig | 0.394 |
| rasal1 | 0.394 |
| tfdp2 | 0.394 |
| trim39 | 0.394 |
| dgkz | 0.394 |
| cops2 | 0.394 |
| c80913 | 0.393 |
| spry3 | 0.393 |
| dgke | 0.393 |
| camk1 | 0.393 |
| nenf | 0.393 |
| rsu1 | 0.393 |
| camk1d | 0.393 |
| ppp1r15b | 0.393 |
| pdcl3 | 0.393 |
| mcm3 | 0.392 |
| hipk3 | 0.392 |
| polr2f | 0.392 |
| inpp4a | 0.392 |
| cpne6 | 0.392 |
| pdk3 | 0.392 |
| d12ertd647e | 0.392 |
| g3bp1 | 0.391 |
| ccnd3 | 0.391 |
| cnot7 | 0.391 |
| eif3i | 0.391 |
| ankrd28 | 0.391 |
| oip5 | 0.391 |
| ppp1r12b | 0.391 |
| ttbk2 | 0.391 |
| kank2 | 0.391 |
| 1700009n14rik | 0.391 |
| ulk1 | 0.391 |
| habp4 | 0.391 |
| pib5pa | 0.391 |
| camkk1 | 0.391 |
| slbp | 0.39 |
| tdrd7 | 0.39 |
| spred2 | 0.39 |
| arhgap10 | 0.389 |
| oxsr1 | 0.389 |
| dub1a | 0.389 |
| arhgap26 | 0.389 |
| rps6ka4 | 0.389 |
| mcm10 | 0.389 |
| nek2 | 0.389 |
| fbxw5 | 0.389 |
| znf512b | 0.389 |
| 9430023l20rik | 0.388 |
| stk33 | 0.388 |
| mcm7 | 0.388 |
| cdk5r1 | 0.388 |
| plek | 0.388 |
| shcbp1 | 0.388 |
| cnot10 | 0.388 |
| polr1e | 0.388 |
| gys2 | 0.387 |
| ankrd17 | 0.387 |
| cyb561d2 | 0.387 |
| ythdc1 | 0.387 |
| gadd45g | 0.387 |
| gak | 0.387 |
| zfp445 | 0.387 |
| ccna2 | 0.387 |
| rbm16 | 0.387 |
| fbxw11 | 0.386 |
| ppm1l | 0.386 |
| nme4 | 0.386 |
| mettl1 | 0.386 |
| stap2 | 0.386 |
| rhbdl1 | 0.386 |
| pcnp | 0.386 |
| khdrbs2 | 0.385 |
| metap1 | 0.385 |
| lcmt2 | 0.385 |
| supt4h1 | 0.385 |
| sugt1 | 0.385 |
| ccnb1ip1 | 0.385 |
| eif3k | 0.385 |
| skp1a | 0.384 |
| coro1b | 0.384 |
| ptrh2 | 0.384 |
| pdxk | 0.384 |
| ldoc1 | 0.384 |
| ptpn4 | 0.383 |
| rpl37 | 0.383 |
| med6 | 0.383 |
| tut1 | 0.383 |
| tk-ps2 | 0.383 |
| pfkfb3 | 0.383 |
| mapkbp1 | 0.383 |
| ksr1 | 0.383 |
| zfp458 | 0.383 |
| foxm1 | 0.383 |
| zfyve27 | 0.383 |
| ppp1r1a | 0.383 |
| lcmt1 | 0.382 |
| glyctk | 0.382 |
| rplp2 | 0.382 |
| ppm1d | 0.382 |
| kndc1 | 0.382 |
| d930014e17rik | 0.382 |
| cdca7l | 0.382 |
| thoc5 | 0.382 |
| rasa2 | 0.382 |
| trib2 | 0.382 |
| eif5 | 0.381 |
| dusp21 | 0.381 |
| erh | 0.381 |
| d5mit274 | 0.381 |
| eif2ak1 | 0.381 |
| ssh1 | 0.381 |
| pgam5 | 0.381 |
| dub1 | 0.381 |
| aatf | 0.381 |
| cds1 | 0.381 |
| dyrk1a | 0.381 |
| dusp8 | 0.38 |
| eif2s2 | 0.38 |
| smek1 | 0.38 |
| caprin2 | 0.38 |
| tssk6 | 0.38 |
| fgfr1op | 0.38 |
| 1110006o17rik | 0.38 |
| a230067g21rik | 0.38 |
| rcbtb1 | 0.38 |
| mapk13 | 0.38 |
| pank1 | 0.38 |
| ppap2c | 0.379 |
| tbrg4 | 0.379 |
| cdk4 | 0.379 |
| sf3b1 | 0.378 |
| metap2 | 0.378 |
| ssh3 | 0.378 |
| afap1 | 0.378 |
| bc021395 | 0.378 |
| plekha2 | 0.377 |
| ccnd2 | 0.377 |
| mcm2 | 0.377 |
| poldip3 | 0.377 |
| camk1g | 0.377 |
| med28 | 0.377 |
| rpap3 | 0.377 |
| csnk1a1 | 0.377 |
| psmd11 | 0.377 |
| cops5 | 0.377 |
| th1l | 0.377 |
| wdr4 | 0.377 |
| 1700007e06rik | 0.377 |
| mtmr3 | 0.376 |
| 2300002d11rik | 0.376 |
| itpkb | 0.376 |
| cdv3 | 0.376 |
| dgkh | 0.376 |
| kank1 | 0.376 |
| nanp | 0.376 |
| kif20b | 0.376 |
| atpif1 | 0.376 |
| ibtk | 0.376 |
| sertad2 | 0.376 |
| mkln1 | 0.375 |
| tfg | 0.375 |
| psmd8 | 0.375 |
| hmga1-rs1 | 0.375 |
| dhps | 0.375 |
| rasgrp3 | 0.375 |
| mylk3 | 0.375 |
| ippk | 0.375 |
| nrp | 0.375 |
| ranbp10 | 0.375 |
| btg1 | 0.375 |
| fry | 0.375 |
| bag2 | 0.375 |
| chn2 | 0.375 |
| dpp7 | 0.374 |
| tyro3-rs1 | 0.374 |
| btg3 | 0.374 |
| pim2 | 0.374 |
| ndrg2 | 0.374 |
| psma3 | 0.374 |
| rps15a | 0.374 |
| spin2 | 0.374 |
| ccar1 | 0.374 |
| jund2 | 0.374 |
| orc6l | 0.374 |
| e4f1 | 0.374 |
| anapc7 | 0.374 |
| fbxl20 | 0.374 |
| mapbpip | 0.374 |
| cnksr1 | 0.373 |
| rp23-136k12.4 | 0.373 |
| psmd14 | 0.373 |
| htatsf1 | 0.373 |
| psmf1 | 0.373 |
| ccnt1 | 0.373 |
| psmc6 | 0.373 |
| dguok | 0.373 |
| fbxo38 | 0.373 |
| gem | 0.373 |
| eef1b2 | 0.373 |
| rps6ka2 | 0.373 |
| ssrp1 | 0.373 |
| ier5 | 0.372 |
| dck | 0.372 |
| dedd | 0.372 |
| rps6kb2 | 0.372 |
| cul1 | 0.372 |
| brd4 | 0.372 |
| wdfy2 | 0.372 |
| grb14 | 0.372 |
| tnip2 | 0.372 |
| ptpn5 | 0.372 |
| pdxp | 0.371 |
| il17rd | 0.371 |
| eif2ak4 | 0.371 |
| sh2d3c | 0.371 |
| map4k5 | 0.371 |
| kis2 | 0.371 |
| enpp4 | 0.371 |
| nt5c1a | 0.371 |
| ier2 | 0.371 |
| zbtb4 | 0.371 |
| adk | 0.371 |
| mcm5 | 0.371 |
| mcts1 | 0.371 |
| ubash3a | 0.371 |
| snx26 | 0.37 |
| ppp1r16b | 0.37 |
| clspn | 0.37 |
| csnk1d | 0.37 |
| nob1 | 0.37 |
| ccpn-ps | 0.37 |
| ppat | 0.37 |
| plrg1 | 0.37 |
| emi1 | 0.37 |
| qars | 0.37 |
| map4k4 | 0.369 |
| tom1l1 | 0.369 |
| rps6ka6 | 0.369 |
| tcea2 | 0.369 |
| gsg2 | 0.369 |
| inpp4b | 0.369 |
| 6330503k22rik | 0.369 |
| clip3 | 0.369 |
| cdgap | 0.369 |
| klhl2 | 0.369 |
| tnni3k | 0.369 |
| d11mit84 | 0.369 |
| thg1l | 0.368 |
| rps5 | 0.368 |
| mrpl41 | 0.368 |
| fert2 | 0.368 |
| pdk2 | 0.368 |
| rchy1 | 0.368 |
| pag1 | 0.368 |
| akap3 | 0.368 |
| ranbp9 | 0.368 |
| tmem33 | 0.368 |
| eif3g | 0.367 |
| evi5 | 0.367 |
| map3k11 | 0.367 |
| mtmr7 | 0.367 |
| hsh2d | 0.367 |
| fastk | 0.367 |
| brap | 0.367 |
| ccne1 | 0.367 |
| ddx18 | 0.366 |
| bmx | 0.366 |
| ppp2r3c | 0.366 |
| sertad3 | 0.366 |
| pi4kb | 0.366 |
| frag1 | 0.366 |
| rap1gds1 | 0.366 |
| pdk1 | 0.366 |
| cdk5 | 0.366 |
| 4932425i24rik | 0.366 |
| ppfibp1 | 0.366 |
| papola | 0.366 |
| cnot6 | 0.365 |
| tsen54 | 0.365 |
| sorbs3 | 0.365 |
| mcrs1 | 0.365 |
| magea4 | 0.365 |
| smoc2 | 0.365 |
| ppap2a | 0.364 |
| plaa | 0.364 |
| ppapdc1a | 0.364 |
| sgpp1 | 0.364 |
| uxs1 | 0.364 |
| trib1 | 0.364 |
| stk39 | 0.364 |
| txnl1 | 0.364 |
| 1110002b05rik | 0.364 |
| igbp1 | 0.364 |
| tprkb | 0.364 |
| mat2b | 0.364 |
| bpnt1 | 0.364 |
| stmn3 | 0.363 |
| btg4 | 0.363 |
| cul4b | 0.363 |
| cirbp-rs3 | 0.363 |
| cirbp-rs1 | 0.363 |
| eapp | 0.363 |
| 4933424b01rik | 0.363 |
| rin1 | 0.363 |
| mprip | 0.363 |
| sfrs4 | 0.363 |
| mrvi1 | 0.362 |
| sec16b | 0.362 |
| ubash3b | 0.362 |
| ube2cbp | 0.362 |
| naif1 | 0.362 |
| ptpn9 | 0.362 |
| map4k1 | 0.362 |
| 2310033p09rik | 0.362 |
| spp2 | 0.362 |
| eif6 | 0.362 |
| cfl2 | 0.361 |
| mcm6 | 0.361 |
| cops3 | 0.361 |
| rhov | 0.361 |
| phospho2 | 0.361 |
| akap4 | 0.361 |
| 2500003m10rik | 0.361 |
| itgb1bp1 | 0.361 |
| ect2 | 0.361 |
| mink1 | 0.361 |
| nap1l4 | 0.361 |
| mapk11 | 0.361 |
| snx6 | 0.36 |
| nsmce4a | 0.36 |
| paqr3 | 0.36 |
| cdc16 | 0.36 |
| syt17 | 0.36 |
| map3k7ip3 | 0.36 |
| sass6 | 0.36 |
| card14 | 0.36 |
| bc016495 | 0.359 |
| map3k2 | 0.359 |
| ptp4a1 | 0.359 |
| gulp1 | 0.359 |
| eg664805 | 0.359 |
| mbc2 | 0.359 |
| akap14 | 0.359 |
| 4930579c15rik | 0.359 |
| nol1 | 0.359 |
| ptplad1 | 0.359 |
| lmln | 0.359 |
| prcc | 0.359 |
| 6720467c03rik | 0.358 |
| chka | 0.358 |
| pa2g4 | 0.358 |
| d4mit112 | 0.358 |
| myd116 | 0.358 |
| cdc42bpb | 0.358 |
| centa1 | 0.357 |
| mov10l1 | 0.357 |
| hist1h2ae | 0.357 |
| zfp446 | 0.357 |
| spred1 | 0.357 |
| txnl4b | 0.357 |
| mpzl1 | 0.357 |
| a530050d06rik | 0.357 |
| anapc2 | 0.357 |
| gm1866 | 0.357 |
| dapk2 | 0.357 |
| pik3r3 | 0.356 |
| saps2 | 0.356 |
| pgam1 | 0.356 |
| pnck | 0.356 |
| osbpl9 | 0.356 |
| ppp3r2 | 0.356 |
| ccdc88a | 0.356 |
| pcif1 | 0.356 |
| gas2 | 0.356 |
| exosc8 | 0.356 |
| akap12 | 0.356 |
| hbp1 | 0.356 |
| cnot1 | 0.356 |
| racgap1 | 0.356 |
| cct4 | 0.356 |
| prc1 | 0.356 |
| crebzf | 0.356 |
| bnip2 | 0.356 |
| mtap4 | 0.356 |
| jub | 0.356 |
| abtb1 | 0.356 |
| fignl1 | 0.355 |
| sh2d2a | 0.355 |
| smpd2 | 0.355 |
| centd3 | 0.355 |
| acly | 0.355 |
| pea15a | 0.355 |
| ccdc50 | 0.355 |
| uchl5 | 0.355 |
| ivns1abp | 0.355 |
| ihpk3 | 0.355 |
| cirbp | 0.355 |
| ppp1r12a | 0.355 |
