# Supplementary material for: GIT2 Acts as a Potential Keystone Protein in Functional Hypothalamic Networks Associated with Age-Related Phenotypic Changes in Rats
Source: PLoS One. 2012 May 14;7(5):e36975. doi: 10.1371/journal.pone.0036975 (PMC3351446; doi:10.1371/journal.pone.0036975)
Supplement: Table S24 — GeneIndexer latent semantic indexing (LSI) of significantly-regulated ‘Regulation of cell proliferation’ GO term group. Using the GO term group ‘Regulation of cell proliferation’ as an input term, a list of the top 1000 implicitly-correlated (LSI correlation score >0.1) was generated using a full genome background list. (DOC) [file pone.0036975.s028.doc]

**Table S24. GeneIndexer latent semantic indexing (LSI) of significantly-regulated ‘Regulation of cell proliferation’ GO term group.** Using the GO term group ‘Regulation of cell proliferation’ as an input term, a list of the top 1000 implicitly- correlated (LSI correlation score >0.1) was generated using a full genome background list.

| ***Regulation of cell proliferation*** |  |
| --- | --- |
|  |  |
| **Protein Symbol** | **LSI correlation score** |
| olfml1 | 0.731 |
| spin2 | 0.729 |
| trp53i13 | 0.721 |
| ddx18 | 0.715 |
| 6720467c03rik | 0.712 |
| ccdc7 | 0.706 |
| cirbp-rs3 | 0.701 |
| cirbp-rs1 | 0.701 |
| 5830417c01rik | 0.697 |
| med29 | 0.68 |
| slfn9 | 0.677 |
| slfn4 | 0.677 |
| erdr1 | 0.677 |
| slfn7 | 0.674 |
| slfn14 | 0.674 |
| slfn6 | 0.674 |
| carp1 | 0.674 |
| mirn221 | 0.67 |
| mirn222 | 0.67 |
| setd3 | 0.662 |
| bc048355 | 0.662 |
| gas4 | 0.661 |
| slfn5 | 0.66 |
| slfn8 | 0.66 |
| slfn10 | 0.66 |
| 2500003m10rik | 0.66 |
| igfbp5-ip | 0.657 |
| thap11 | 0.655 |
| ay036118 | 0.648 |
| rogdi | 0.648 |
| arid3b | 0.647 |
| slfn1 | 0.647 |
| wdr26 | 0.645 |
| etnmg1 | 0.643 |
| tcstv1 | 0.641 |
| zscan4f | 0.641 |
| rai12 | 0.641 |
| gm347 | 0.641 |
| btbd7 | 0.64 |
| ldoc1 | 0.64 |
| cdca4 | 0.638 |
| cirbp-rs2 | 0.637 |
| a930041i02rik | 0.635 |
| tnk1 | 0.635 |
| tmem22 | 0.635 |
| kcmf1 | 0.633 |
| osgin1 | 0.633 |
| bc030867 | 0.631 |
| glt8d3 | 0.631 |
| ottmusg00000008540 | 0.631 |
| trim14 | 0.628 |
| d15ertd621e | 0.628 |
| depdc1a | 0.628 |
| 2610528e23rik | 0.627 |
| btg3 | 0.627 |
| 2300002d11rik | 0.626 |
| styk1 | 0.625 |
| zfp612 | 0.624 |
| ccdc72 | 0.623 |
| toe1 | 0.622 |
| ergic2 | 0.62 |
| akirin2 | 0.619 |
| bc029169 | 0.618 |
| smyd4 | 0.617 |
| 1110001a07rik | 0.617 |
| mobkl1b | 0.616 |
| ptchd2 | 0.616 |
| mirn34a | 0.615 |
| slfn3 | 0.614 |
| abi3bp | 0.613 |
| slfn2 | 0.613 |
| 0610007c21rik | 0.613 |
| caprin2 | 0.612 |
| dice1 | 0.611 |
| ppp1r1c | 0.611 |
| fignl1 | 0.61 |
| ay074887 | 0.609 |
| npdc1 | 0.608 |
| 1700047i17rik1 | 0.607 |
| zfp322a | 0.606 |
| api5 | 0.606 |
| pptc7 | 0.605 |
| 2310047d13rik | 0.605 |
| tnfaip8 | 0.605 |
| zfp449 | 0.603 |
| axud1 | 0.603 |
| mirn150 | 0.603 |
| myct1 | 0.603 |
| etnk1 | 0.603 |
| rtkn2 | 0.602 |
| ppan | 0.602 |
| st18 | 0.602 |
| eaf2 | 0.601 |
| eg621324 | 0.601 |
| gas5 | 0.601 |
| mirn9-1 | 0.601 |
| mageh1 | 0.601 |
| helz | 0.6 |
| naif1 | 0.6 |
| asah3l | 0.6 |
| 1110005a23rik | 0.6 |
| mrp63 | 0.599 |
| ccdc86 | 0.598 |
| gas2l1 | 0.596 |
| tpt1p | 0.596 |
| btg4 | 0.596 |
| thg1l | 0.595 |
| tbc1d2 | 0.595 |
| zc3h8 | 0.595 |
| actr8 | 0.595 |
| rpl15 | 0.595 |
| lzts2 | 0.595 |
| zc3h10 | 0.594 |
| sertad3 | 0.594 |
| spred3 | 0.594 |
| lypd1 | 0.594 |
| ccng2 | 0.594 |
| epb4.1l4b | 0.593 |
| rrp15 | 0.593 |
| ars2 | 0.593 |
| 1190002h23rik | 0.593 |
| ppm1h | 0.593 |
| zfp383 | 0.593 |
| 2010005j08rik | 0.593 |
| rps28 | 0.592 |
| nrbp2 | 0.592 |
| pdcd2l | 0.592 |
| mirn106a | 0.592 |
| sertad2 | 0.591 |
| fbxo31 | 0.591 |
| tmem184c | 0.591 |
| trim16 | 0.59 |
| rqcd1 | 0.589 |
| zfp689 | 0.589 |
| lrrn1 | 0.589 |
| sltm | 0.589 |
| commd5 | 0.588 |
| sav1 | 0.588 |
| mobkl1a | 0.587 |
| tfpt | 0.587 |
| lyb7 | 0.586 |
| slc25a33 | 0.586 |
| cmtm8 | 0.586 |
| 9830130m13rik | 0.586 |
| sif3 | 0.585 |
| sif1 | 0.585 |
| sif2 | 0.585 |
| ccpg1 | 0.585 |
| c330027c09rik | 0.585 |
| bbx | 0.584 |
| 1110006o17rik | 0.584 |
| phf19 | 0.583 |
| rps29 | 0.583 |
| tob2 | 0.583 |
| mcts1 | 0.583 |
| mrfap1 | 0.583 |
| wdr16 | 0.582 |
| ncym1 | 0.582 |
| lrig3 | 0.582 |
| heca | 0.582 |
| 2810417h13rik | 0.582 |
| tcf25 | 0.582 |
| klhdc2 | 0.581 |
| lgals12 | 0.581 |
| pbk | 0.58 |
| cab39l | 0.58 |
| d3ertd300e | 0.58 |
| d19mit75 | 0.58 |
| znhit4 | 0.579 |
| cluap1 | 0.579 |
| pcdh24 | 0.579 |
| vwce | 0.577 |
| creg1 | 0.577 |
| dcbld2 | 0.577 |
| gprc5a | 0.577 |
| mycs | 0.577 |
| nenf | 0.577 |
| mterfd3 | 0.577 |
| nme3 | 0.576 |
| melk | 0.576 |
| d14mit116 | 0.576 |
| lrrc8c | 0.575 |
| cep76 | 0.575 |
| tusc4 | 0.574 |
| e2f7 | 0.574 |
| cnot6l | 0.574 |
| grwd1 | 0.574 |
| gramd4 | 0.574 |
| bcl6b | 0.574 |
| pinc | 0.573 |
| d530049i02rik | 0.573 |
| ier3ip1 | 0.573 |
| tg(tgfa)1efu | 0.573 |
| ecd | 0.573 |
| ypel3 | 0.573 |
| rsu1 | 0.572 |
| kctd11 | 0.572 |
| csrnp2 | 0.572 |
| kank4 | 0.572 |
| kank3 | 0.572 |
| gins1 | 0.572 |
| 4932432k03rik | 0.572 |
| rhbdl1 | 0.572 |
| gadd45gip1 | 0.571 |
| bc005685 | 0.571 |
| cdk2ap2 | 0.571 |
| rp23-143a14.5 | 0.571 |
| zkscan3 | 0.571 |
| ebna1bp2 | 0.571 |
| ube2q2 | 0.571 |
| ogfr | 0.571 |
| adnp2 | 0.569 |
| ndrg4 | 0.569 |
| ccndbp1 | 0.568 |
| eral1 | 0.568 |
| ddx47 | 0.568 |
| dph1 | 0.568 |
| gnl3 | 0.567 |
| cgrrf1 | 0.567 |
| tmem85 | 0.567 |
| wdr6 | 0.567 |
| plac8 | 0.567 |
| bc021395 | 0.567 |
| kank1 | 0.567 |
| 1810011o10rik | 0.567 |
| smoc2 | 0.567 |
| plekhm3 | 0.567 |
| pes1 | 0.566 |
| zfp641 | 0.566 |
| hbp1 | 0.566 |
| lin28b | 0.566 |
| pxdn | 0.566 |
| ppapdc1 | 0.565 |
| pelo | 0.565 |
| diras1 | 0.565 |
| tbrg4 | 0.565 |
| eg637021 | 0.564 |
| nip7 | 0.564 |
| sbno1 | 0.564 |
| csrnp3 | 0.564 |
| lrrc4 | 0.564 |
| rpl38 | 0.564 |
| ifi205 | 0.564 |
| nol1 | 0.564 |
| mpb1l | 0.563 |
| shcbp1 | 0.563 |
| eif2c5 | 0.563 |
| mycbpap | 0.563 |
| emp1 | 0.563 |
| asb11 | 0.563 |
| tmem45a | 0.563 |
| spin1 | 0.563 |
| 4930500o05rik | 0.563 |
| lyrm1 | 0.562 |
| hilda | 0.562 |
| serinc3 | 0.562 |
| hrasls | 0.562 |
| st5 | 0.562 |
| tspan3 | 0.562 |
| fndc3b | 0.561 |
| sdccag1 | 0.561 |
| ier2 | 0.561 |
| prdm14 | 0.561 |
| cxcl17 | 0.561 |
| 4933424b01rik | 0.561 |
| svep1 | 0.561 |
| mirn21 | 0.56 |
| pttg1ip | 0.56 |
| nol7 | 0.56 |
| prl2c3 | 0.56 |
| trim71 | 0.56 |
| kank2 | 0.56 |
| cdca7 | 0.56 |
| myg1 | 0.56 |
| spata5 | 0.56 |
| 2310056p07rik | 0.559 |
| rbbp6 | 0.559 |
| mirn181a-2 | 0.559 |
| mirn7b | 0.559 |
| zfp191 | 0.558 |
| dnm3os | 0.558 |
| zfp326 | 0.558 |
| zfp418 | 0.558 |
| laptm5 | 0.558 |
| sdpr | 0.557 |
| armcx2 | 0.557 |
| e4f1 | 0.557 |
| nat14 | 0.557 |
| prdm4 | 0.556 |
| klhl6 | 0.556 |
| phlda1 | 0.556 |
| sh2d4a | 0.556 |
| trpd52l3 | 0.556 |
| phf13 | 0.555 |
| trim47 | 0.555 |
| gltscr2 | 0.555 |
| atp13a3 | 0.555 |
| eg668489 | 0.555 |
| cenpm | 0.555 |
| 2610036l11rik | 0.555 |
| ms4a3 | 0.555 |
| mirn181a-1 | 0.555 |
| zc3h15 | 0.555 |
| anp32b | 0.555 |
| anks1 | 0.554 |
| dbndd2 | 0.554 |
| gabpb2 | 0.554 |
| 2410016o06rik | 0.554 |
| specc1 | 0.554 |
| klhl2 | 0.554 |
| ccar1 | 0.554 |
| zscan10 | 0.554 |
| sdccag3 | 0.553 |
| samsn1 | 0.553 |
| tacc2 | 0.553 |
| mtus1 | 0.553 |
| etv3 | 0.553 |
| med28 | 0.553 |
| v165-d-j-c mu | 0.553 |
| lce3c | 0.553 |
| apitd1 | 0.553 |
| arid3c | 0.553 |
| gm397 | 0.553 |
| bq559217 | 0.553 |
| zfp113 | 0.552 |
| mon1b | 0.552 |
| pdcl3 | 0.552 |
| rnf43 | 0.552 |
| cks2 | 0.551 |
| casz1 | 0.551 |
| nek4 | 0.551 |
| cmtm5 | 0.551 |
| tspyl2 | 0.551 |
| hemgn | 0.551 |
| dullard | 0.55 |
| hunk | 0.55 |
| lats1 | 0.55 |
| 9130213b05rik | 0.549 |
| syf2 | 0.549 |
| brd3 | 0.549 |
| bzw1 | 0.549 |
| pim3 | 0.549 |
| dub2 | 0.548 |
| erf | 0.548 |
| myadm | 0.548 |
| rbm45 | 0.548 |
| abtb1 | 0.548 |
| cdk2ap1 | 0.547 |
| hist1h2ag | 0.547 |
| ifitm1 | 0.547 |
| sectm1a | 0.547 |
| sectm1b | 0.547 |
| wn | 0.547 |
| tis | 0.547 |
| 1700126l10rik | 0.546 |
| eapp | 0.546 |
| tusc2 | 0.546 |
| mllt11 | 0.546 |
| drg1 | 0.546 |
| ccdc80 | 0.546 |
| gm885 | 0.546 |
| rsrc2 | 0.546 |
| tmem8 | 0.545 |
| scube2 | 0.545 |
| rtp3 | 0.545 |
| loc667882 | 0.545 |
| rybp | 0.544 |
| ensmusg00000079376 | 0.543 |
| zfp445 | 0.543 |
| zfp46 | 0.543 |
| e2f8 | 0.542 |
| ifi9.5 | 0.542 |
| 2610110g12rik | 0.542 |
| eg653016 | 0.542 |
| b3gnt8 | 0.542 |
| apcdd1 | 0.541 |
| rhox3a | 0.541 |
| rhox2a | 0.541 |
| rnf130 | 0.541 |
| 4732474o15rik | 0.541 |
| d17mit181 | 0.541 |
| bmyc | 0.541 |
| pik3ip1 | 0.54 |
| nat13 | 0.54 |
| pgcp | 0.54 |
| 2010100o12rik | 0.54 |
| ssbp2 | 0.54 |
| b230208h17rik | 0.54 |
| 2010109i03rik | 0.54 |
| nkap | 0.54 |
| tm4sf5 | 0.54 |
| morf4l2 | 0.54 |
| mak10 | 0.539 |
| apold1 | 0.539 |
| dhx32 | 0.539 |
| scara3 | 0.539 |
| scpro5 | 0.539 |
| ints6 | 0.539 |
| dok4 | 0.539 |
| ttc9 | 0.539 |
| caprin1 | 0.538 |
| rerg | 0.538 |
| 6330500d04rik | 0.538 |
| aw146242 | 0.538 |
| fgfr1op | 0.538 |
| pcid2 | 0.538 |
| dapl1 | 0.537 |
| wfdc1 | 0.537 |
| phf16 | 0.537 |
| rhox4b | 0.537 |
| chchd8 | 0.537 |
| zfp74 | 0.536 |
| tsc22d4 | 0.536 |
| mrpl36 | 0.536 |
| eras | 0.536 |
| glipr1 | 0.536 |
| narg2 | 0.536 |
| nek5 | 0.536 |
| cdc2l5 | 0.536 |
| tpd52l2 | 0.536 |
| bop1 | 0.536 |
| ensmusg00000071552 | 0.535 |
| ociad1 | 0.535 |
| steap3 | 0.535 |
| ciapin1 | 0.535 |
| ddx54 | 0.535 |
| lats2 | 0.535 |
| prdm6 | 0.535 |
| rbms3 | 0.535 |
| wtap | 0.534 |
| bcas3 | 0.534 |
| trp53rk | 0.534 |
| tmem123 | 0.534 |
| agr3 | 0.533 |
| ard1b | 0.533 |
| gm1549 | 0.533 |
| trim35 | 0.533 |
| epb4.1l4a | 0.533 |
| ssfa2 | 0.533 |
| hrasls3 | 0.533 |
| prl2c4 | 0.533 |
| sh3rf3 | 0.533 |
| lrrc8a | 0.533 |
| nfam1 | 0.532 |
| tsc22d1 | 0.532 |
| tmem158 | 0.532 |
| mrps25 | 0.532 |
| cd8mts2 | 0.532 |
| cnih | 0.532 |
| mirn26a-1 | 0.532 |
| mxi1-rs1 | 0.532 |
| uxt | 0.532 |
| rhbdd3 | 0.532 |
| stk38l | 0.532 |
| mia2 | 0.532 |
| banp | 0.532 |
| rb1cc1 | 0.531 |
| zfp97 | 0.531 |
| bc016579 | 0.531 |
| psmg2 | 0.531 |
| fgfr3-ps | 0.531 |
| 6430517e21rik | 0.531 |
| phlpp | 0.531 |
| spred2 | 0.531 |
| zfp622 | 0.531 |
| b020018g12rik | 0.531 |
| tob1 | 0.531 |
| zmiz1 | 0.531 |
| ankrd17 | 0.531 |
| 1110014j01rik | 0.53 |
| bnipl | 0.53 |
| pygo2 | 0.53 |
| rod1 | 0.53 |
| mtbp | 0.53 |
| rhobtb2 | 0.53 |
| nit1 | 0.53 |
| klf8 | 0.53 |
| rbbp9 | 0.53 |
| mirn23b | 0.53 |
| sertad1 | 0.53 |
| nr2c2ap | 0.529 |
| zfp58 | 0.529 |
| ktelc1 | 0.529 |
| mxd4 | 0.529 |
| smpdl3a | 0.529 |
| cxxc5 | 0.529 |
| dlgap5 | 0.529 |
| arl6ip1 | 0.529 |
| oip5 | 0.529 |
| rrp1b | 0.529 |
| ippk | 0.528 |
| thsd1 | 0.528 |
| spdya | 0.528 |
| glis1 | 0.528 |
| lyar | 0.528 |
| pltr9 | 0.528 |
| rab37 | 0.528 |
| egfl7 | 0.528 |
| ola1 | 0.528 |
| zfp260 | 0.528 |
| podn | 0.527 |
| asb15 | 0.527 |
| nek3 | 0.527 |
| sdad1 | 0.527 |
| zfhx2 | 0.527 |
| ift74 | 0.527 |
| akirin1 | 0.527 |
| thoc1 | 0.527 |
| rbm35a | 0.527 |
| shisa5 | 0.527 |
| gtlf3a | 0.527 |
| gtlf3b | 0.527 |
| nsun2 | 0.527 |
| gdpd2 | 0.526 |
| tmem54 | 0.526 |
| dub3 | 0.526 |
| mxd3 | 0.526 |
| nudcd1 | 0.526 |
| zfp42-ps1 | 0.526 |
| tex2 | 0.526 |
| tg(dvl2)7gsb | 0.525 |
| eg240921 | 0.525 |
| tpd52 | 0.525 |
| cdr2 | 0.525 |
| dppa5-ps7 | 0.525 |
| dppa5-ps5 | 0.525 |
| dppa5-ps3 | 0.525 |
| dppa5-ps2 | 0.525 |
| dppa5-ps6 | 0.525 |
| dppa5-ps4 | 0.525 |
| dppa5-ps1 | 0.525 |
| 4930420k17rik | 0.525 |
| chpt1 | 0.525 |
| rps6ka6 | 0.525 |
| magmas | 0.524 |
| stra13 | 0.524 |
| ptchd3 | 0.524 |
| 6330569m22rik | 0.524 |
| d330017j20rik | 0.524 |
| prr16 | 0.524 |
| tns3 | 0.524 |
| lmln | 0.524 |
| nlrc3 | 0.524 |
| zbtb4 | 0.524 |
| nudt6 | 0.524 |
| tyro3-rs1 | 0.523 |
| wdr12 | 0.523 |
| ehf | 0.523 |
| ptrh2 | 0.523 |
| eg435755 | 0.523 |
| lrig2 | 0.523 |
| d15mit198 | 0.523 |
| mina | 0.523 |
| tg(zfp38)d1htz | 0.523 |
| tg(zfp38)y7htz | 0.523 |
| tg(zfp38)b8htz | 0.523 |
| tg(zfp38)a4htz | 0.523 |
| hspa9-ps1 | 0.523 |
| depdc6 | 0.523 |
| slc35c2 | 0.523 |
| hepacam | 0.523 |
| cnot7 | 0.523 |
| mageb2 | 0.523 |
| 1700081d17rik | 0.522 |
| emo1 | 0.522 |
| zfp91 | 0.522 |
| gdpd5 | 0.522 |
| frag1 | 0.522 |
| tfdp2 | 0.522 |
| cnot10 | 0.522 |
| mettl3 | 0.522 |
| zfp319 | 0.522 |
| ptov1 | 0.522 |
| mark4 | 0.522 |
| hmgb3 | 0.522 |
| oraov1 | 0.522 |
| samd9l | 0.522 |
| plk2 | 0.522 |
| mirn202 | 0.521 |
| mirn137 | 0.521 |
| mirn503 | 0.521 |
| mirn674 | 0.521 |
| btbd10 | 0.521 |
| gsdmd | 0.521 |
| cdk10 | 0.521 |
| letmd1 | 0.521 |
| mybl1 | 0.521 |
| wisp2 | 0.521 |
| tpt1-ps1 | 0.521 |
| wfdc5 | 0.521 |
| tg(bmp4)6blh | 0.521 |
| noc3l | 0.521 |
| cdv3 | 0.521 |
| hivep2 | 0.521 |
| 2610018g03rik | 0.521 |
| ptpn23 | 0.52 |
| rpl35a | 0.52 |
| rsl1d1 | 0.52 |
| arid3a | 0.52 |
| prl2c5 | 0.52 |
| nme6 | 0.52 |
| lyb3 | 0.52 |
| cnot2 | 0.52 |
| cyl | 0.52 |
| bc010304 | 0.52 |
| zfp212 | 0.52 |
| ppp2r3c | 0.519 |
| trim8 | 0.519 |
| lrig1 | 0.519 |
| grhl3 | 0.519 |
| rbm27 | 0.519 |
| ai427122 | 0.518 |
| mirn155 | 0.518 |
| tcfl5 | 0.518 |
| zfp703 | 0.518 |
| spz1 | 0.518 |
| ptpn14 | 0.518 |
| rnu5g | 0.518 |
| mrpl3 | 0.518 |
| rbm5 | 0.518 |
| rasip1 | 0.517 |
| mageb5 | 0.517 |
| camk1d | 0.517 |
| pea15b | 0.517 |
| cc2d1b | 0.517 |
| cml3 | 0.517 |
| nat5 | 0.517 |
| zfp26 | 0.516 |
| pptcs2 | 0.516 |
| tera | 0.516 |
| ilkap | 0.516 |
| rnf6 | 0.516 |
| tmub1 | 0.516 |
| sap30bp | 0.516 |
| 6030408c04rik | 0.516 |
| trp53i11 | 0.516 |
| denr | 0.516 |
| tpd52l1 | 0.516 |
| efhd2 | 0.516 |
| btg1 | 0.516 |
| camk2n2 | 0.516 |
| xrra1 | 0.515 |
| zfp458 | 0.515 |
| d8mit336 | 0.515 |
| d8mit96 | 0.515 |
| tex15 | 0.515 |
| epsti1 | 0.515 |
| siah1b | 0.515 |
| plrg1 | 0.515 |
| morf4l1 | 0.515 |
| dusp26 | 0.514 |
| mustn1 | 0.514 |
| lmyc2 | 0.514 |
| bcas2 | 0.514 |
| nrp | 0.514 |
| cnot3 | 0.514 |
| ppapdc3 | 0.514 |
| il17rd | 0.514 |
| lin28 | 0.514 |
| zfp382 | 0.514 |
| muc15 | 0.513 |
| dusp3 | 0.513 |
| ik | 0.513 |
| ddefl1 | 0.513 |
| c530043g21rik | 0.513 |
| pnpt1 | 0.513 |
| zfp60 | 0.513 |
| mtap2k | 0.513 |
| trap1a | 0.513 |
| def6 | 0.513 |
| mirn93 | 0.513 |
| bc063263 | 0.513 |
| 1200015f23rik | 0.512 |
| n4bp2l2 | 0.512 |
| dap | 0.512 |
| batf | 0.512 |
| 381484 | 0.512 |
| anp32c | 0.512 |
| rbm6 | 0.512 |
| mov10l1 | 0.512 |
| e2f6 | 0.512 |
| hist4 | 0.512 |
| dtd1 | 0.512 |
| ms4a7 | 0.512 |
| stk16 | 0.511 |
| plk4 | 0.511 |
| sh3bgrl | 0.511 |
| dpf2 | 0.511 |
| zfp521 | 0.511 |
| ccng1 | 0.511 |
| sipa1 | 0.511 |
| cdk5rap3 | 0.511 |
| efhd1 | 0.511 |
| gas1 | 0.511 |
| cdkn3 | 0.511 |
| lgr5 | 0.511 |
| rreb1 | 0.511 |
| 1810015c04rik | 0.511 |
| ep400 | 0.511 |
| mrpl13 | 0.51 |
| pds5b | 0.51 |
| 100039796 | 0.51 |
| rsf1 | 0.51 |
| tg(wnt3)7gsb | 0.51 |
| trim45 | 0.51 |
| ebf3 | 0.51 |
| if3 | 0.51 |
| 2310016c08rik | 0.51 |
| myt1 | 0.51 |
| phc3 | 0.51 |
| tg(rv-cyclin)29dlh | 0.51 |
| piwil2 | 0.51 |
| mirn24-1 | 0.51 |
| tmem131 | 0.51 |
| zfp385a | 0.51 |
| tpd52-ps | 0.51 |
| sall2 | 0.51 |
| wdr75 | 0.509 |
| mirn27b | 0.509 |
| tmeff1 | 0.509 |
| ccdc85b | 0.509 |
| ablim3 | 0.509 |
| pcnp | 0.509 |
| fgf16 | 0.508 |
| mat2b | 0.508 |
| ccnj | 0.508 |
| ottmusg00000022410 | 0.508 |
| ier5 | 0.508 |
| cnot8 | 0.508 |
| enc1 | 0.508 |
| hsh2d | 0.508 |
| zfp830 | 0.507 |
| mirn16-2 | 0.507 |
| trib2 | 0.507 |
| ifitm5 | 0.507 |
| smarcad1 | 0.507 |
| gas2l2 | 0.507 |
| rbm19 | 0.507 |
| 5830457o10rik | 0.507 |
| a130040m12rik | 0.506 |
| ei24 | 0.506 |
| pcbp4 | 0.506 |
| ifi202b | 0.506 |
| gmfg | 0.506 |
| tg(nes-rtta)306rvs | 0.506 |
| cd248 | 0.506 |
| 4632434i11rik | 0.506 |
| usp22 | 0.506 |
| tm4sf4 | 0.506 |
| pmepa1 | 0.506 |
| zdhhc16 | 0.506 |
| sbf1 | 0.506 |
| mmd | 0.506 |
| hn1 | 0.506 |
| spata17 | 0.506 |
| fbxo38 | 0.506 |
| d9sut1e | 0.506 |
| tspan13 | 0.506 |
| sec16b | 0.506 |
| trim33 | 0.505 |
| brd7 | 0.505 |
| 1700021f07rik | 0.505 |
| mcrs1 | 0.505 |
| rufy2 | 0.505 |
| mnt | 0.505 |
| trerf1 | 0.505 |
| cytl1 | 0.505 |
| sp5 | 0.505 |
| 2200002d01rik | 0.505 |
| nanos1 | 0.505 |
| dixdc1 | 0.504 |
| dub1 | 0.504 |
| arhgef17 | 0.504 |
| d11mit77 | 0.504 |
| lzic | 0.504 |
| tsku | 0.504 |
| uhrf2 | 0.504 |
| lzts1 | 0.504 |
| treml2 | 0.504 |
| ncaph2 | 0.503 |
| mylc2pl | 0.503 |
| armcx1 | 0.503 |
| vezf1 | 0.503 |
| 1810009o10rik | 0.503 |
| a230083h22rik | 0.503 |
| gpr87 | 0.503 |
| vrk3 | 0.503 |
| gimap9 | 0.503 |
| 6720460f02rik | 0.502 |
| lancl2 | 0.502 |
| dmtf1 | 0.502 |
| pdlim2 | 0.502 |
| pwp1 | 0.502 |
| spic | 0.502 |
| ccpn-ps | 0.502 |
| sidt1 | 0.502 |
| stard13 | 0.501 |
| slc35a4 | 0.501 |
| mirn20a | 0.501 |
| gpr149 | 0.501 |
| mcm3ap | 0.501 |
| zfp185 | 0.501 |
| plk-ps1 | 0.501 |
| tspan2 | 0.501 |
| dusp5 | 0.501 |
| dppa2 | 0.501 |
| pkn3 | 0.501 |
| g0s2 | 0.5 |
| prkcdbp | 0.5 |
| mirn124a-1 | 0.5 |
| 2010110p09rik | 0.5 |
| rasl11b | 0.5 |
| ifitm2 | 0.5 |
| gas2 | 0.5 |
| bc037156 | 0.5 |
| fcrlb | 0.5 |
| aw125753 | 0.5 |
| mtf2 | 0.499 |
| rbm38 | 0.499 |
| egfl6 | 0.499 |
| zfp639 | 0.499 |
| tnfaip2 | 0.499 |
| fezf1 | 0.499 |
| vasn | 0.499 |
| gadd45g | 0.499 |
| 1100001g20rik | 0.499 |
| prcc | 0.499 |
| tg(foxn1)6jlb | 0.499 |
| tg(foxn1)1jlb | 0.499 |
| ccdc88a | 0.499 |
| timd2 | 0.499 |
| rbm15 | 0.499 |
| them4 | 0.499 |
| hemt1 | 0.498 |
| gml | 0.498 |
| wsb2 | 0.498 |
| dtx3l | 0.498 |
| ngp | 0.498 |
| scaper | 0.498 |
| zbtb32 | 0.498 |
| rps27 | 0.498 |
| spdef | 0.498 |
| chac1 | 0.498 |
| prap1 | 0.498 |
| chpst | 0.498 |
| eg433968 | 0.498 |
| larp6 | 0.497 |
| dnajc2 | 0.497 |
| thyn1 | 0.497 |
| tiaf2 | 0.497 |
| ivns1abp | 0.497 |
| bcar3 | 0.497 |
| loh11cr2a | 0.497 |
| kifc4b | 0.497 |
| 2810004i08rik | 0.497 |
| eif3h | 0.497 |
| arid5a | 0.497 |
| tcta | 0.497 |
| lims2 | 0.497 |
| maf1 | 0.497 |
| gpr124 | 0.497 |
| lrp12 | 0.497 |
| piwil1 | 0.497 |
| dpy19l4 | 0.497 |
| ccdc115 | 0.497 |
| limd1 | 0.497 |
| adi1 | 0.497 |
| 2010011i20rik | 0.496 |
| 4930432k21rik | 0.496 |
| gpr65 | 0.496 |
| crebzf | 0.496 |
| tcea3 | 0.496 |
| mirn16-1 | 0.496 |
| sh3bgrl3 | 0.496 |
| rbak | 0.496 |
| l3mbtl2 | 0.496 |
| fbxo15 | 0.496 |
| cnpy2 | 0.496 |
| ppp1r16b | 0.496 |
| tacc3 | 0.496 |
| tcfap4 | 0.496 |
| cirbp | 0.496 |
| yaf2 | 0.496 |
| mirn124a-2 | 0.496 |
| aw146020 | 0.495 |
| gsdma1 | 0.495 |
| narg1 | 0.495 |
| glcci1 | 0.495 |
| hormad1 | 0.495 |
| ly6e | 0.495 |
| dido1 | 0.495 |
| fat3 | 0.495 |
| atp8a2 | 0.495 |
| ddx39 | 0.495 |
| plekho1 | 0.495 |
| uhmk1 | 0.495 |
| cnot1 | 0.495 |
| steap2 | 0.495 |
| aifm2 | 0.495 |
| gfi1b | 0.495 |
| mirn328 | 0.495 |
| 1200002n14rik | 0.494 |
| higd1a | 0.494 |
| g3bp1 | 0.494 |
| rbm3 | 0.494 |
| d6wsu176e | 0.494 |
| mizf | 0.494 |
| tg(wapnotch4)10rnc | 0.494 |
| ttc4 | 0.494 |
| llgl2 | 0.494 |
| btbd14b | 0.494 |
| expi | 0.494 |
| ambra1 | 0.493 |
| spsb1 | 0.493 |
| ddx56 | 0.493 |
| mirnlet7g | 0.493 |
| ndufa13 | 0.493 |
| degs1 | 0.493 |
| arhgap8 | 0.493 |
| d11mit139 | 0.493 |
| ggnbp2 | 0.493 |
| rcbtb1 | 0.493 |
| gimap1 | 0.493 |
| ddx19a | 0.493 |
| mpeg1 | 0.493 |
| sash1 | 0.493 |
| blcap | 0.493 |
| loc100041323 | 0.492 |
| arhgap22 | 0.492 |
| slc37a3 | 0.492 |
| tmsb10 | 0.492 |
| igsf10 | 0.492 |
| ptpn3 | 0.492 |
| zfp263 | 0.492 |
| agbl2 | 0.492 |
| plxdc1 | 0.492 |
| 2010317e24rik | 0.492 |
| wee2 | 0.492 |
| usp28 | 0.492 |
| lrrn3 | 0.492 |
| dtx4 | 0.492 |
| ctnnal1 | 0.492 |
| msi2 | 0.492 |
| prr15 | 0.492 |
| zgpat | 0.492 |
| lrrc26 | 0.492 |
| sulf1 | 0.492 |
| tg(k6odctr)55tgo | 0.492 |
| mirn124a-3 | 0.491 |
| 1-Sep | 0.491 |
| bambi | 0.491 |
| zfp354a | 0.491 |
| 2010001j22rik | 0.491 |
| ptp4a2 | 0.491 |
| cdcp1 | 0.491 |
| 1700020c11rik | 0.491 |
| mtdh | 0.491 |
| wapal | 0.491 |
| igsf11 | 0.491 |
| rpl13a | 0.491 |
| zbtb38 | 0.491 |
| stard10 | 0.491 |
| bai1 | 0.491 |
| cables1 | 0.491 |
| git2 | 0.491 |
| igfbpl1 | 0.491 |
| dtl | 0.49 |
| trim69 | 0.49 |
| cep57 | 0.49 |
| nmyc2 | 0.49 |
| dazap2 | 0.49 |
| aktip | 0.49 |
| ing4 | 0.49 |
| dus2l | 0.49 |
| asprv1 | 0.49 |
| ddit4l | 0.49 |
| mirn200b | 0.49 |
| alpk3 | 0.489 |
| gpr4 | 0.489 |
| klhdc1 | 0.489 |
| ly16 | 0.489 |
| pa2g4 | 0.489 |
| fau | 0.489 |
| unc5d | 0.489 |
| rag1ap1 | 0.489 |
| tom1l1 | 0.489 |
| swap70 | 0.489 |
| cpsf3l | 0.489 |
| ints9 | 0.489 |
| tle6 | 0.489 |
| zfp287 | 0.489 |
| ly6k | 0.488 |
| zfp652 | 0.488 |
| bzw2 | 0.488 |
| eps8l1 | 0.488 |
| rp23-105o4.2 | 0.488 |
| ikzf2 | 0.488 |
| zfp395 | 0.488 |
| clca2 | 0.488 |
| 1500015o10rik | 0.488 |
