# Supplementary material for: GIT2 Acts as a Potential Keystone Protein in Functional Hypothalamic Networks Associated with Age-Related Phenotypic Changes in Rats
Source: PLoS One. 2012 May 14;7(5):e36975. doi: 10.1371/journal.pone.0036975 (PMC3351446; doi:10.1371/journal.pone.0036975)
Supplement: Table S25 — GeneIndexer latent semantic indexing (LSI) of significantly-regulated ‘Cytoskeletal protein binding’ GO term group. Using the GO term group ‘Cytoskeletal protein binding’ as an input term, a list of the top 1000 implicitly-correlated (LSI correlation score >0.1) was generated using a full genome background list. (DOC) [file pone.0036975.s029.doc]

**Table S25. GeneIndexer latent semantic indexing (LSI) of significantly-regulated ‘Cytoskeletal protein binding’ GO term group.** Using the GO term group ‘Cytoskeletal protein binding’ as an input term, a list of the top 1000 implicitly-correlated (LSI correlation score >0.1) was generated using a full genome background list.

| ***Cytoskeletal protein binding*** |  |
| --- | --- |
|  |  |
| **Protein Symbol** | **LSI correlation score** |
| twf2 | 0.697 |
| coro2b | 0.688 |
| twf1 | 0.683 |
| arpm1 | 0.667 |
| rhpn1 | 0.665 |
| 2310014h01rik | 0.662 |
| plek2 | 0.658 |
| e130112l23rik | 0.655 |
| stk35 | 0.65 |
| 1700007i08rik | 0.637 |
| klhdc1 | 0.635 |
| ablim2 | 0.635 |
| klhl5 | 0.63 |
| coro1c | 0.628 |
| csrp2bp | 0.628 |
| eps8l1 | 0.624 |
| svil | 0.622 |
| eps8l3 | 0.62 |
| macf1 | 0.619 |
| kif9 | 0.616 |
| fhod1 | 0.615 |
| actrt2 | 0.613 |
| eps8l2 | 0.613 |
| arhgap17 | 0.612 |
| fblim1 | 0.611 |
| tesk1 | 0.611 |
| mkln1 | 0.609 |
| nrbp1 | 0.607 |
| ccin | 0.606 |
| spire1 | 0.604 |
| layn | 0.603 |
| coro1b | 0.603 |
| armc8 | 0.602 |
| tesk2 | 0.601 |
| arhgap12 | 0.601 |
| arhgap9 | 0.6 |
| apbb1ip | 0.599 |
| arhgap21 | 0.599 |
| kank2 | 0.598 |
| fbf1 | 0.597 |
| afap1 | 0.597 |
| tsnaxip1 | 0.597 |
| 2610204m08rik | 0.597 |
| spire2 | 0.596 |
| arhgap24 | 0.596 |
| 2810003c17rik | 0.594 |
| arpc4 | 0.594 |
| actrt1 | 0.591 |
| coro2a | 0.591 |
| rhpn2 | 0.591 |
| tox4 | 0.591 |
| kank4 | 0.59 |
| kank3 | 0.59 |
| mphosph8 | 0.589 |
| ktn1 | 0.588 |
| bc060632 | 0.588 |
| ppp1r12c | 0.588 |
| nckap1l | 0.588 |
| cdgap | 0.586 |
| phactr3 | 0.586 |
| nckap1 | 0.586 |
| ai662250 | 0.581 |
| exosc8 | 0.581 |
| raver1 | 0.579 |
| epb4.1l2 | 0.579 |
| zfp7 | 0.578 |
| megf11 | 0.578 |
| scyl3 | 0.578 |
| ablim1 | 0.578 |
| rai14 | 0.576 |
| cdc42se1 | 0.575 |
| ints4 | 0.575 |
| 6430548m08rik | 0.575 |
| 7-Sep | 0.575 |
| klhl2 | 0.575 |
| memo1 | 0.575 |
| epb4.9 | 0.574 |
| d930014e17rik | 0.573 |
| ablim3 | 0.572 |
| trim67 | 0.572 |
| daam1 | 0.572 |
| pfn3 | 0.571 |
| eif3k | 0.571 |
| zyx | 0.571 |
| fmnl1 | 0.571 |
| p42pop | 0.57 |
| lrrc59 | 0.569 |
| 2310003c23rik | 0.569 |
| zfp622 | 0.569 |
| rab3ip | 0.568 |
| capzb | 0.567 |
| dennd4c | 0.567 |
| baiap2l1 | 0.566 |
| nckipsd | 0.565 |
| arpc5 | 0.564 |
| 4831426i19rik | 0.564 |
| lima1 | 0.564 |
| parvb | 0.564 |
| 10-Sep | 0.563 |
| parvg | 0.563 |
| trim46 | 0.563 |
| nexn | 0.562 |
| actr1b | 0.562 |
| cotl1 | 0.561 |
| rhot2 | 0.561 |
| lrrc16a | 0.561 |
| zfp655 | 0.56 |
| rhot1 | 0.558 |
| triobp | 0.557 |
| ddx54 | 0.557 |
| cdc42ep4 | 0.557 |
| aw554918 | 0.557 |
| actl7a | 0.557 |
| fchsd1 | 0.556 |
| gas2l2 | 0.556 |
| farp1 | 0.555 |
| rod1 | 0.555 |
| eml3 | 0.555 |
| ropn1 | 0.555 |
| capza1 | 0.555 |
| kank1 | 0.555 |
| klhl20 | 0.554 |
| ccdc88b | 0.553 |
| eml2 | 0.551 |
| mical1 | 0.551 |
| eml5 | 0.551 |
| d13mit91 | 0.551 |
| shroom4 | 0.551 |
| cdc42ep1 | 0.551 |
| evi5l | 0.55 |
| ankrd35 | 0.55 |
| eif3g | 0.55 |
| nebl | 0.55 |
| polr3e | 0.549 |
| ssna1 | 0.549 |
| tmod3 | 0.549 |
| arhgap4 | 0.549 |
| arhgap26 | 0.548 |
| spert | 0.547 |
| arpc2 | 0.547 |
| fchsd2 | 0.547 |
| zfp449 | 0.546 |
| centd3 | 0.546 |
| klhl17 | 0.546 |
| kndc1 | 0.545 |
| pip5kl1 | 0.544 |
| coro7 | 0.543 |
| bspry | 0.542 |
| cdc42ep3 | 0.542 |
| nip7 | 0.542 |
| mobkl3 | 0.542 |
| pfn2 | 0.542 |
| bin3 | 0.541 |
| kbtbd10 | 0.541 |
| 4432412l15rik | 0.541 |
| rab35 | 0.541 |
| kptn | 0.541 |
| prdm12 | 0.541 |
| naif1 | 0.54 |
| cdc42ep5 | 0.54 |
| whdc1 | 0.54 |
| fermt2 | 0.539 |
| ncdn | 0.539 |
| elmo2 | 0.539 |
| palld | 0.539 |
| spnb5 | 0.538 |
| 11-Sep | 0.538 |
| ccpg1 | 0.538 |
| dbnl | 0.538 |
| 4631416l12rik | 0.538 |
| c80913 | 0.538 |
| micalcl | 0.537 |
| pdlim1 | 0.537 |
| arhgap19 | 0.536 |
| ivns1abp | 0.536 |
| ankrd42 | 0.536 |
| trip6 | 0.536 |
| evl | 0.536 |
| fbxo10 | 0.536 |
| kif13b | 0.536 |
| 1-Sep | 0.536 |
| dennd3 | 0.535 |
| chchd1 | 0.535 |
| zbtb6 | 0.535 |
| phldb2 | 0.534 |
| arhgap15 | 0.534 |
| pfn4 | 0.534 |
| diap3 | 0.534 |
| sorbs3 | 0.533 |
| 1700016k13rik | 0.533 |
| fnbp1l | 0.533 |
| rbm12 | 0.533 |
| txndc9 | 0.533 |
| wdr1 | 0.532 |
| sfrs18 | 0.532 |
| ssh2 | 0.532 |
| arpc3 | 0.532 |
| zfp59 | 0.532 |
| 3110043j09rik | 0.532 |
| grwd1 | 0.531 |
| tnik | 0.531 |
| rhobtb3 | 0.531 |
| arhgap25 | 0.53 |
| zfml | 0.53 |
| mtss1 | 0.529 |
| rbm26 | 0.529 |
| 4930418g15rik | 0.529 |
| arl8a | 0.528 |
| rc3h2 | 0.528 |
| zfp207 | 0.528 |
| limk2 | 0.527 |
| fnbp1 | 0.527 |
| hnrnpul2 | 0.527 |
| ssbp3 | 0.527 |
| raph1 | 0.527 |
| arl8b | 0.526 |
| parva | 0.526 |
| pfdn2 | 0.526 |
| d10wsu52e | 0.525 |
| 6-Sep | 0.525 |
| pbxip1 | 0.525 |
| rufy2 | 0.525 |
| mpp1 | 0.525 |
| zfp458 | 0.525 |
| rnd2 | 0.524 |
| mtap1s | 0.524 |
| arid5a | 0.524 |
| rragc | 0.524 |
| eg208426 | 0.524 |
| pmv23 | 0.523 |
| pabpc4 | 0.523 |
| abi3 | 0.522 |
| ssh3 | 0.522 |
| f830045p16rik | 0.522 |
| cnpy2 | 0.521 |
| wbp1 | 0.521 |
| phactr1 | 0.521 |
| rsu1 | 0.521 |
| cnn3 | 0.521 |
| tdrd7 | 0.521 |
| mprip | 0.521 |
| sorbs2 | 0.521 |
| gspt2 | 0.521 |
| ttc35 | 0.521 |
| lasp1 | 0.521 |
| frmd4b | 0.52 |
| mical3 | 0.52 |
| edc3 | 0.52 |
| gins4 | 0.52 |
| maea | 0.519 |
| sh3bgrl2 | 0.519 |
| zfp661 | 0.519 |
| harbi1 | 0.518 |
| sgsm3 | 0.518 |
| tbca | 0.518 |
| zfand3 | 0.518 |
| wdr25 | 0.518 |
| actr1a | 0.517 |
| grit | 0.517 |
| fcho2 | 0.517 |
| diap2 | 0.517 |
| zc3h14 | 0.516 |
| zfpl1 | 0.516 |
| fhod3 | 0.516 |
| rnf10 | 0.516 |
| ostf1 | 0.515 |
| fgd3 | 0.515 |
| fmnl3 | 0.515 |
| spata13 | 0.515 |
| arpc1a | 0.515 |
| av249152 | 0.514 |
| arhgap22 | 0.514 |
| cfl2 | 0.514 |
| srgap3 | 0.514 |
| hebp2 | 0.513 |
| gas2l1 | 0.513 |
| arl6ip4 | 0.513 |
| rmnd5a | 0.513 |
| ubl7 | 0.513 |
| avil | 0.513 |
| wbp11 | 0.513 |
| sh3bp1 | 0.513 |
| actr10 | 0.513 |
| myo1e | 0.513 |
| arhgap10 | 0.512 |
| arpc1b | 0.512 |
| sec14l1 | 0.512 |
| tm9sf1 | 0.512 |
| clip3 | 0.512 |
| rhov | 0.512 |
| ftcd | 0.511 |
| d1mit330 | 0.511 |
| cap2 | 0.511 |
| wbp2 | 0.511 |
| shroom2 | 0.511 |
| ppfibp2 | 0.511 |
| rps6kc1 | 0.511 |
| rp23-157o10.7 | 0.51 |
| 8-Sep | 0.51 |
| wipf3 | 0.51 |
| rnf38 | 0.51 |
| ankrd7 | 0.51 |
| ppm1f | 0.51 |
| cnnm3 | 0.51 |
| pctk2 | 0.51 |
| mdn1 | 0.51 |
| vgll1 | 0.51 |
| zfp239 | 0.509 |
| baiap3 | 0.509 |
| 9030409g11rik | 0.509 |
| dbn1 | 0.509 |
| exoc2 | 0.509 |
| ankrd28 | 0.509 |
| trim7 | 0.509 |
| nif3l1 | 0.509 |
| iqub | 0.509 |
| klhdc2 | 0.508 |
| anks1 | 0.508 |
| ttc9 | 0.508 |
| trim39 | 0.508 |
| trappc4 | 0.508 |
| nfu1 | 0.507 |
| iapls3-31 | 0.507 |
| iapt11 | 0.507 |
| arid3c | 0.507 |
| centg3 | 0.507 |
| nat14 | 0.507 |
| ddx47 | 0.507 |
| nol8 | 0.507 |
| pef1 | 0.507 |
| rnf151 | 0.507 |
| shroom1 | 0.506 |
| ssx2ip | 0.506 |
| lancl2 | 0.506 |
| rgnef | 0.506 |
| au040829 | 0.506 |
| sdcbp2 | 0.506 |
| rnd1 | 0.506 |
| amotl2 | 0.506 |
| mybph | 0.506 |
| cct6b | 0.506 |
| pfdn1 | 0.505 |
| abra | 0.505 |
| mast1 | 0.505 |
| sergef | 0.505 |
| tbcb | 0.504 |
| trpd52l3 | 0.504 |
| habp4 | 0.504 |
| srp68 | 0.504 |
| d2mit496 | 0.504 |
| ldb2 | 0.503 |
| actr8 | 0.503 |
| mycbpap | 0.503 |
| tpm4 | 0.503 |
| strn3 | 0.503 |
| psd | 0.503 |
| wdr12 | 0.503 |
| rpl12 | 0.503 |
| zik1 | 0.502 |
| nol4 | 0.502 |
| zfyve1 | 0.502 |
| rnpc3 | 0.502 |
| ttc3 | 0.502 |
| itsn2 | 0.502 |
| rundc3a | 0.502 |
| synj2 | 0.502 |
| ubqln3 | 0.502 |
| tlk2 | 0.502 |
| tln2 | 0.501 |
| dctn4 | 0.501 |
| ccdc19 | 0.501 |
| vps26b | 0.501 |
| arhgap20 | 0.501 |
| rell1 | 0.501 |
| rell2 | 0.501 |
| cnnm1 | 0.501 |
| myo18a | 0.501 |
| plekho1 | 0.501 |
| wbp5 | 0.5 |
| d17mit196 | 0.5 |
| arhgef2 | 0.5 |
| 2-Sep | 0.5 |
| schip1 | 0.5 |
| rexo1 | 0.5 |
| brd1 | 0.499 |
| bcnp1 | 0.499 |
| arl6ip6 | 0.499 |
| igfn1 | 0.499 |
| arhgap29 | 0.499 |
| zfp113 | 0.499 |
| nrap | 0.498 |
| baiap2 | 0.498 |
| s100a16 | 0.498 |
| cdc42bpa | 0.498 |
| strn4 | 0.498 |
| saps3 | 0.498 |
| actr6 | 0.498 |
| gripap1 | 0.497 |
| ai427122 | 0.497 |
| cap1 | 0.497 |
| tex2 | 0.497 |
| centa1 | 0.497 |
| cdc42ep2 | 0.497 |
| rps21 | 0.497 |
| actr3b | 0.497 |
| matr3 | 0.497 |
| pick2 | 0.497 |
| pick5 | 0.497 |
| pick3 | 0.497 |
| pick4 | 0.497 |
| epb4.1l4b | 0.497 |
| zdhhc16 | 0.497 |
| gem | 0.497 |
| arhgdig | 0.497 |
| 100039796 | 0.496 |
| ppfia1 | 0.496 |
| srp54b | 0.496 |
| cnot3 | 0.496 |
| 6330417g02rik | 0.496 |
| rbm15b | 0.496 |
| zcchc17 | 0.495 |
| arhgap27 | 0.495 |
| pdlim4 | 0.495 |
| abi2 | 0.495 |
| yod1 | 0.495 |
| synpo2 | 0.495 |
| trip10 | 0.495 |
| cstf3 | 0.495 |
| lpxn | 0.495 |
| spef1 | 0.494 |
| pfdn4 | 0.494 |
| zbed4 | 0.494 |
| riok3 | 0.494 |
| myo16 | 0.494 |
| cabyr | 0.494 |
| bin2 | 0.494 |
| klhl31 | 0.494 |
| pdlim3 | 0.493 |
| ottmusg00000012291 | 0.493 |
| prpf40a | 0.493 |
| iqgap2 | 0.493 |
| bc017158 | 0.493 |
| ypel5 | 0.493 |
| s100z | 0.493 |
| pigy | 0.493 |
| ubxd5 | 0.493 |
| lnx2 | 0.492 |
| zcchc4 | 0.492 |
| lsm12 | 0.492 |
| zfp212 | 0.492 |
| myo10 | 0.491 |
| mylc2b | 0.491 |
| zfp472 | 0.491 |
| syx1 | 0.491 |
| syx2 | 0.491 |
| cep57 | 0.491 |
| ssh1 | 0.491 |
| trim3 | 0.491 |
| pkn3 | 0.49 |
| clmn | 0.49 |
| fscn3 | 0.49 |
| eral1 | 0.49 |
| pigyl | 0.49 |
| pfdn5 | 0.49 |
| rhbdl1 | 0.49 |
| cnbpl1 | 0.49 |
| emv24 | 0.49 |
| tbcd | 0.49 |
| gdpd1 | 0.49 |
| phactr2 | 0.489 |
| plek | 0.489 |
| stard10 | 0.489 |
| arl2bp | 0.489 |
| zfp300 | 0.489 |
| ppp1r16b | 0.489 |
| cnot1 | 0.489 |
| eif1ad | 0.489 |
| smek1 | 0.489 |
| rhobtb1 | 0.489 |
| cep250 | 0.489 |
| elmo1 | 0.488 |
| sdad1 | 0.488 |
| nob1 | 0.488 |
| jub | 0.488 |
| spag4 | 0.488 |
| lztr1 | 0.488 |
| unc84b | 0.488 |
| arl2 | 0.488 |
| cit | 0.488 |
| iapls3-28 | 0.488 |
| 12-Sep | 0.487 |
| zfp263 | 0.487 |
| prkcbp1 | 0.487 |
| anp32e | 0.487 |
| d8ertd82e | 0.487 |
| zfp639 | 0.487 |
| med28 | 0.487 |
| fsd1 | 0.487 |
| hnrpx-ps | 0.487 |
| zfp174 | 0.486 |
| trim45 | 0.486 |
| bnip2 | 0.486 |
| lrrfip1 | 0.486 |
| tuba8 | 0.486 |
| 2700060e02rik | 0.486 |
| cdc42bpb | 0.486 |
| trim14 | 0.485 |
| cpne6 | 0.485 |
| iapls1-19 | 0.485 |
| aup1 | 0.485 |
| ylpm1 | 0.485 |
| osbpl3 | 0.485 |
| nol10 | 0.485 |
| pacsin2 | 0.485 |
| cml3 | 0.485 |
| sgsm2 | 0.484 |
| pcdhb12 | 0.484 |
| wdr44 | 0.484 |
| esf1 | 0.484 |
| c1d | 0.484 |
| cd2bp2 | 0.484 |
| zbtb25 | 0.484 |
| smtnl1 | 0.484 |
| pgm5 | 0.484 |
| hnrnpm | 0.484 |
| arhgef6 | 0.483 |
| eif6 | 0.483 |
| rbm22 | 0.483 |
| cyfip2 | 0.483 |
| enah | 0.483 |
| pop5 | 0.483 |
| zfyve27 | 0.483 |
| usp6nl | 0.482 |
| saps1 | 0.482 |
| pcdh3 | 0.482 |
| lrba | 0.482 |
| hdgfl1 | 0.482 |
| rnu5g | 0.482 |
| ypel1 | 0.482 |
| ptpn14 | 0.482 |
| smad-ps1 | 0.481 |
| zc3h7b | 0.481 |
| ggnbp1 | 0.481 |
| fgd2 | 0.481 |
| bzrap1 | 0.481 |
| zfp593 | 0.481 |
| zhx3 | 0.481 |
| nsbp1 | 0.481 |
| trim36 | 0.48 |
| flii | 0.48 |
| clic5 | 0.48 |
| myom1 | 0.48 |
| rhou | 0.48 |
| cdk5rap1 | 0.48 |
| dennd4a | 0.479 |
| dcdc2b | 0.479 |
| exoc1 | 0.479 |
| crip2 | 0.479 |
| cpne3 | 0.479 |
| reps1 | 0.479 |
| bxdc1 | 0.479 |
| iars | 0.479 |
| ankrd44 | 0.479 |
| ficd | 0.479 |
| hdgfrp3 | 0.479 |
| ddx19b | 0.478 |
| eprs | 0.478 |
| rpl18a | 0.478 |
| rlf | 0.478 |
| aw125753 | 0.478 |
| afap1l2 | 0.478 |
| tes | 0.478 |
| cml1 | 0.478 |
| cml5 | 0.478 |
| map6d1 | 0.478 |
| 6530403a03rik | 0.478 |
| scamp4 | 0.478 |
| sgsm1 | 0.478 |
| gpn1 | 0.478 |
| loc384848 | 0.478 |
| b230208h17rik | 0.478 |
| dync1li2 | 0.478 |
| ard1b | 0.477 |
| rragd | 0.477 |
| pphln1 | 0.477 |
| mcf2l | 0.477 |
| lpp | 0.477 |
| ppfia4 | 0.477 |
| fhl3 | 0.477 |
| troap | 0.477 |
| sdccag8 | 0.477 |
| nol6 | 0.477 |
| mon1b | 0.477 |
| dnmlp1 | 0.477 |
| zfp330 | 0.477 |
| wdfy3 | 0.477 |
| 1700009n14rik | 0.476 |
| sacm1l | 0.476 |
| ropn1l | 0.476 |
| eif1b | 0.476 |
| iqsec2 | 0.476 |
| cnot6 | 0.476 |
| gprin2 | 0.476 |
| spnb3 | 0.476 |
| ccdc88a | 0.475 |
| dtd1 | 0.475 |
| cml2 | 0.475 |
| prrg2 | 0.475 |
| gulp1 | 0.475 |
| tm9sf3 | 0.475 |
| unc84a | 0.475 |
| dnttip1 | 0.475 |
| nostrin | 0.475 |
| rabgap1 | 0.474 |
| odf1 | 0.474 |
| d0wfb1e | 0.474 |
| tpt1-ps1 | 0.474 |
| tmem135 | 0.474 |
| ccdc88c | 0.474 |
| zbtb1 | 0.473 |
| cops7b | 0.473 |
| trappc1 | 0.473 |
| zfp457 | 0.473 |
| xpo6 | 0.473 |
| zmynd19 | 0.473 |
| zfp498 | 0.473 |
| tex21 | 0.473 |
| ottmusg00000004461 | 0.473 |
| centd1 | 0.473 |
| mrpl12 | 0.473 |
| sestd1 | 0.473 |
| mif4gd | 0.473 |
| thoc7 | 0.473 |
| pld4 | 0.472 |
| pacsin1 | 0.472 |
| 1700126l10rik | 0.472 |
| pdik1l | 0.472 |
| zbtb34 | 0.472 |
| clic3 | 0.472 |
| arhgef19 | 0.471 |
| pitpnm1 | 0.471 |
| sh3yl1 | 0.471 |
| pstpip1 | 0.471 |
| phactr4 | 0.471 |
| exoc8 | 0.471 |
| btbd1 | 0.471 |
| rhog | 0.471 |
| stard13 | 0.471 |
| rp23-100c5.8 | 0.471 |
| trio | 0.471 |
| ddef2 | 0.471 |
| 1700006a11rik | 0.471 |
| pir | 0.471 |
| poldip2 | 0.47 |
| plekhb1 | 0.47 |
| d10ertd610e | 0.47 |
| rab8b | 0.47 |
| zdhhc1 | 0.47 |
| rap2c | 0.47 |
| ttc1 | 0.47 |
| yaf2 | 0.47 |
| pin4 | 0.47 |
| a130090k04rik | 0.47 |
| tmem33 | 0.47 |
| csrnp2 | 0.47 |
| eg630579 | 0.47 |
| eppk1 | 0.469 |
| caskin1 | 0.469 |
| ptpn18 | 0.469 |
| snx21 | 0.469 |
| rgs22 | 0.469 |
| btbd2 | 0.469 |
| osgepl1 | 0.469 |
| cyfip1 | 0.469 |
| d11jkn1e | 0.469 |
| mycbp | 0.469 |
| pcnp | 0.469 |
| mki67ip | 0.469 |
| bud31 | 0.469 |
| tmod1 | 0.469 |
| nudt16l1 | 0.468 |
| pscdbp | 0.468 |
| oog1 | 0.468 |
| nck2 | 0.468 |
| lcp1 | 0.468 |
| zfp326 | 0.468 |
| phf5a | 0.468 |
| rimbp2 | 0.468 |
| zfp438 | 0.467 |
| pscd1 | 0.467 |
| ppfia3 | 0.467 |
| gtf3c6 | 0.467 |
| sdpr | 0.467 |
| gdpd2 | 0.467 |
| mapre3 | 0.467 |
| khdrbs2 | 0.467 |
| rtkn | 0.467 |
| hrbl | 0.467 |
| arhgef9 | 0.467 |
| anln | 0.467 |
| ythdc1 | 0.467 |
| mrps35 | 0.467 |
| cpne8 | 0.467 |
| shank2 | 0.467 |
| sh3pxd2a | 0.467 |
| son | 0.467 |
| tmod4 | 0.467 |
| epb4.1l1 | 0.466 |
| pcdhgc3 | 0.466 |
| osbpl6 | 0.466 |
| myg1 | 0.466 |
| gdpd4 | 0.466 |
| kalrn | 0.466 |
| fgd1 | 0.466 |
| nat5 | 0.466 |
| zfp382 | 0.466 |
| rufy3 | 0.466 |
| sh3bp4 | 0.466 |
| fuk | 0.466 |
| 4930526h21rik | 0.466 |
| iqsec3 | 0.466 |
| herc1 | 0.466 |
| wdr51b | 0.465 |
| wdr51a | 0.465 |
| u06147 | 0.465 |
| ankar | 0.465 |
| ggnbp2 | 0.465 |
| diap1 | 0.465 |
| sav1 | 0.465 |
| erc1 | 0.465 |
| mars | 0.465 |
| spryd4 | 0.465 |
| cnksr3 | 0.465 |
| ifi35 | 0.465 |
| arl5b | 0.464 |
| tiam2 | 0.464 |
| igfbp5-ip | 0.464 |
| zfp161 | 0.464 |
| syngr2 | 0.464 |
| akap2 | 0.464 |
| bc010304 | 0.464 |
| ppp1r16a | 0.464 |
| specc1 | 0.464 |
| lancl1 | 0.464 |
| git2 | 0.464 |
| rabif | 0.463 |
| trim41 | 0.463 |
| tex24 | 0.463 |
| herc3 | 0.463 |
| riok2 | 0.463 |
| palm | 0.463 |
| kif20b | 0.463 |
| brpf1 | 0.463 |
| stk38l | 0.463 |
| net1 | 0.463 |
| a430041b07rik | 0.463 |
| hook3 | 0.463 |
| cct3 | 0.463 |
| 2500003m10rik | 0.463 |
| arhgef4 | 0.463 |
| rraga | 0.463 |
| fgd4 | 0.463 |
| dync1i1 | 0.462 |
| mylip | 0.462 |
| zhx1 | 0.462 |
| 1500003o03rik | 0.462 |
| zmynd11 | 0.462 |
| srp19 | 0.462 |
| ptpn20 | 0.462 |
| plekhg5 | 0.462 |
| gtpbp2 | 0.462 |
| tomm22 | 0.462 |
| psmg1 | 0.462 |
| zfp187 | 0.462 |
| cnot8 | 0.462 |
| bicd1 | 0.462 |
| asxl3 | 0.462 |
| herc5 | 0.462 |
| riok1 | 0.462 |
| 9430023l20rik | 0.462 |
| ptpn21 | 0.462 |
| zfp641 | 0.462 |
| palm2-akap2 | 0.462 |
| tubgcp6 | 0.462 |
| d10mit260 | 0.462 |
| 4933407c03rik | 0.462 |
| mast4 | 0.461 |
| mterf | 0.461 |
| pfn1 | 0.461 |
| mapbpip | 0.461 |
| nfxl1 | 0.461 |
| snx26 | 0.461 |
| zfp606 | 0.461 |
| ppie | 0.461 |
| ttc4 | 0.461 |
| cenpk | 0.461 |
| capg | 0.461 |
| nbea | 0.461 |
| raver2 | 0.46 |
| srp14 | 0.46 |
| ddx41 | 0.46 |
| amotl1 | 0.46 |
| zfp667 | 0.46 |
| uxt | 0.46 |
| cttnbp2 | 0.46 |
| obsl1 | 0.46 |
| cecr2 | 0.46 |
| hook2 | 0.46 |
| tomm34 | 0.459 |
| sh2d3c | 0.459 |
| rpp21 | 0.459 |
| dock11 | 0.459 |
| usp52 | 0.459 |
| plrg1 | 0.459 |
| strn | 0.459 |
| stk10 | 0.459 |
| mast2 | 0.459 |
| wdr68 | 0.459 |
| wipf2 | 0.459 |
| ankhd1 | 0.459 |
| ccdc65 | 0.458 |
| pscd3 | 0.458 |
| tax1bp3 | 0.458 |
| 4121402d02rik | 0.458 |
| myom2 | 0.458 |
| ppl | 0.458 |
| nek9 | 0.458 |
| srp54a | 0.458 |
| zfp111 | 0.458 |
| obscn | 0.458 |
| gmfg | 0.458 |
| ube2q2 | 0.458 |
| edc4 | 0.458 |
| srgap2 | 0.458 |
| zbtb43 | 0.458 |
| cd302 | 0.458 |
| csrnp3 | 0.458 |
| mcf2 | 0.458 |
| mrps12 | 0.458 |
| wdr13 | 0.458 |
| rusc1 | 0.458 |
| repin1 | 0.457 |
| triml1 | 0.457 |
| dph3 | 0.457 |
| arhgap6 | 0.457 |
| myo9a | 0.457 |
| micall1 | 0.457 |
| 2700078k21rik | 0.457 |
| pcdh18 | 0.457 |
| gas8 | 0.457 |
| dock1 | 0.457 |
| rab6b | 0.457 |
| rnf6 | 0.457 |
| osbp2 | 0.457 |
| mtx3 | 0.457 |
| tomm70a | 0.457 |
| 4930455c21rik | 0.456 |
| trak1 | 0.456 |
| eif3d | 0.456 |
| zfp445 | 0.456 |
| dimt1 | 0.456 |
| farp2 | 0.456 |
| ubtd2 | 0.456 |
| iqgap3 | 0.456 |
| pgam1 | 0.456 |
| cdk5rap2 | 0.456 |
| nrsn2 | 0.456 |
| ulk2 | 0.456 |
| tfpt | 0.455 |
| tomm7 | 0.455 |
| pptc7 | 0.455 |
| shisa4 | 0.455 |
| syf2 | 0.455 |
| rshl2b | 0.455 |
| rassf9 | 0.455 |
| cdc42se2 | 0.455 |
| 2310043l02rik | 0.455 |
| rnf144a | 0.455 |
| tbc1d10a | 0.455 |
| nvl | 0.455 |
| ddef1 | 0.455 |
| map2k1ip1 | 0.455 |
| sbk1 | 0.454 |
| cpsf3l | 0.454 |
| ints9 | 0.454 |
| fbxo17 | 0.454 |
| arfip2 | 0.454 |
| stard9 | 0.454 |
| zfp74 | 0.454 |
| arhgef18 | 0.454 |
| npcd | 0.454 |
| arhgef7 | 0.454 |
| 5133400g04rik | 0.454 |
| svep1 | 0.454 |
| gtf3c3 | 0.454 |
| caprin1 | 0.453 |
| lmcd1 | 0.453 |
| pik3r4 | 0.453 |
| wac | 0.453 |
| rac3 | 0.453 |
| hepacam | 0.453 |
| dstn | 0.453 |
| pknox2 | 0.453 |
| golph3 | 0.453 |
| snrpe-ps1 | 0.453 |
| snrpe-ps2 | 0.453 |
| ctxn3 | 0.453 |
| ralgps2 | 0.453 |
| zfp418 | 0.453 |
| sugt1 | 0.453 |
| stmn3 | 0.453 |
| dcdc5 | 0.453 |
| myoz3 | 0.453 |
| slc41a3 | 0.453 |
| akap8l | 0.452 |
| d4mit110 | 0.452 |
| hist1h4a | 0.452 |
| bola1 | 0.452 |
| bola3 | 0.452 |
| spsb1 | 0.452 |
| odf3 | 0.452 |
| pdlim7 | 0.452 |
| slk | 0.452 |
| polr2c | 0.452 |
| amot | 0.452 |
| zfp386 | 0.452 |
| dullard | 0.452 |
| rap1gds1 | 0.452 |
| csmd2 | 0.452 |
| 1110005a03rik | 0.451 |
| gmip | 0.451 |
| cpxm2 | 0.451 |
| polr2j | 0.451 |
| osbpl7 | 0.451 |
| rhod | 0.451 |
| taok1 | 0.451 |
| midn | 0.451 |
| d030074e01rik | 0.451 |
| rab12 | 0.451 |
| tob2 | 0.451 |
| mettl1 | 0.451 |
| d5mit318 | 0.451 |
| sdccag3 | 0.45 |
| zfp322a | 0.45 |
| gnl3l | 0.45 |
| foxk2 | 0.45 |
| gnl2 | 0.45 |
| mrps5 | 0.45 |
| swap70 | 0.45 |
| mib2 | 0.45 |
| efha1 | 0.449 |
| unc45b | 0.449 |
| tln1 | 0.449 |
| zfp446 | 0.449 |
| rasa2 | 0.449 |
| ppfia2 | 0.449 |
| zfp628 | 0.449 |
| zfp536 | 0.449 |
| dock9 | 0.449 |
| vstm2l | 0.449 |
| rpp14 | 0.449 |
| shcbp1 | 0.449 |
| flnc | 0.449 |
| csdc2 | 0.449 |
| glipr2 | 0.449 |
| ranbp10 | 0.449 |
| rbms2 | 0.449 |
| ppil3 | 0.448 |
| tmem150 | 0.448 |
| lmo7 | 0.448 |
| shkbp1 | 0.448 |
| larp6 | 0.448 |
| shd | 0.448 |
| she | 0.448 |
| pitpnc1 | 0.448 |
| hnrpdl | 0.448 |
| zer1 | 0.448 |
| ajap1 | 0.448 |
| palmd | 0.448 |
| n4bp2 | 0.448 |
| spsb2 | 0.448 |
| cmya5 | 0.448 |
| wtip | 0.448 |
| chd6 | 0.448 |
| 1110006o17rik | 0.447 |
| cct8 | 0.447 |
| trappc5 | 0.447 |
| plekhm3 | 0.447 |
| hfm1 | 0.447 |
| gtpbp10 | 0.447 |
| gtpbp5 | 0.447 |
| 6720467c03rik | 0.447 |
| srfbp1 | 0.447 |
| lmbr1l | 0.447 |
| nme6 | 0.447 |
| rplp1 | 0.447 |
| pkn1 | 0.446 |
| pnrc1 | 0.446 |
| rragb | 0.446 |
| rusc2 | 0.446 |
