# Supplementary material for: GIT2 Acts as a Potential Keystone Protein in Functional Hypothalamic Networks Associated with Age-Related Phenotypic Changes in Rats
Source: PLoS One. 2012 May 14;7(5):e36975. doi: 10.1371/journal.pone.0036975 (PMC3351446; doi:10.1371/journal.pone.0036975)
Supplement: Table S26 — GeneIndexer latent semantic indexing (LSI) of significantly-regulated ‘Macromolecular complex’ GO term group. Using the GO term group ‘Macromolecular complex’ as an input term, a list of the top 1000 implicitly-correlated (LSI correlation score >0.1) was generated using a full genome background list. (DOC) [file pone.0036975.s030.doc]

**Table S26. GeneIndexer latent semantic indexing (LSI) of significantly-regulated ‘Macromolecular complex’ GO term group.** Using the GO term group ‘Macromolecular complex’ as an input term, a list of the top 1000 implicitly-correlated (LSI correlation score >0.1) was generated using a full genome background list.

| ***Macromolecular complex*** |  |
| --- | --- |
|  |  |
| **Protein Symbol** | **LSI correlation score** |
| d030074e01rik | 0.525 |
| cwc15 | 0.511 |
| trappc1 | 0.501 |
| zyg11b | 0.492 |
| gemin8 | 0.478 |
| gemin6 | 0.474 |
| psmd5 | 0.474 |
| trappc4 | 0.472 |
| gemin7 | 0.471 |
| 2410019a14rik | 0.468 |
| gins4 | 0.461 |
| cog4 | 0.46 |
| pih1d1 | 0.457 |
| sf3a1 | 0.456 |
| trappc3 | 0.455 |
| imp4 | 0.451 |
| nol5a | 0.449 |
| gins3 | 0.449 |
| lsm8 | 0.448 |
| mrto4 | 0.446 |
| nup35 | 0.446 |
| noc4l | 0.444 |
| nup133 | 0.438 |
| tmem48 | 0.437 |
| rpap2 | 0.436 |
| sf3a3 | 0.435 |
| srp19 | 0.434 |
| pop7 | 0.433 |
| gtf3c5 | 0.429 |
| gtf3c4 | 0.429 |
| d530033c11rik | 0.428 |
| snapc5 | 0.424 |
| seh1l | 0.424 |
| gtf3c6 | 0.424 |
| exosc4 | 0.423 |
| wdr57 | 0.423 |
| psmd12 | 0.421 |
| wdr61 | 0.421 |
| nup93 | 0.42 |
| 5730449l18rik | 0.418 |
| nup37 | 0.417 |
| nup43 | 0.417 |
| ppih | 0.417 |
| sf3b3 | 0.417 |
| bxdc1 | 0.416 |
| nsmce1 | 0.413 |
| nup107 | 0.413 |
| gemin5 | 0.413 |
| lsm5 | 0.412 |
| lsm3 | 0.412 |
| lsm6 | 0.412 |
| pop5 | 0.41 |
| cops7b | 0.409 |
| nup205 | 0.408 |
| cog5 | 0.408 |
| cog8 | 0.408 |
| cd2bp2 | 0.406 |
| 0610009d07rik | 0.405 |
| ppp4r2 | 0.404 |
| rpp14 | 0.404 |
| cog6 | 0.404 |
| exosc3 | 0.403 |
| snupn | 0.403 |
| cstf3 | 0.402 |
| cog3 | 0.402 |
| 2310003l22rik | 0.402 |
| txnl4a | 0.402 |
| aw554918 | 0.401 |
| ascc3l1 | 0.4 |
| pop4 | 0.4 |
| smndc1 | 0.399 |
| akap8 | 0.399 |
| armc8 | 0.398 |
| pomp | 0.398 |
| eif3j | 0.397 |
| polr2d | 0.397 |
| elp4 | 0.396 |
| sf3b4 | 0.396 |
| timm9 | 0.396 |
| cog7 | 0.394 |
| snrpf | 0.394 |
| psmd11 | 0.394 |
| srp54b | 0.394 |
| med7 | 0.393 |
| nol5 | 0.393 |
| rnuxa | 0.393 |
| med26 | 0.393 |
| lsm11 | 0.391 |
| uqcrh | 0.391 |
| nfu1 | 0.39 |
| ddx46 | 0.389 |
| snrpg | 0.389 |
| ndufaf1 | 0.388 |
| kti12 | 0.388 |
| cog2 | 0.388 |
| gemin4 | 0.387 |
| anapc13 | 0.386 |
| tomm7 | 0.386 |
| 1110019j04rik | 0.386 |
| snrpd2 | 0.385 |
| 6530403a03rik | 0.385 |
| rpo1-3 | 0.385 |
| nup188 | 0.385 |
| ankrd52 | 0.384 |
| nudt21 | 0.384 |
| srp68 | 0.384 |
| tox4 | 0.384 |
| eftud2 | 0.384 |
| usp39 | 0.384 |
| timm50 | 0.384 |
| sec13 | 0.383 |
| ddx23 | 0.383 |
| lsm10 | 0.383 |
| gtf3c1 | 0.383 |
| gtf3c2 | 0.383 |
| med6 | 0.382 |
| med8 | 0.382 |
| tomm22 | 0.381 |
| timm22 | 0.381 |
| trappc6a | 0.381 |
| gle1 | 0.381 |
| sf3b2 | 0.381 |
| gtf3c3 | 0.38 |
| nola1 | 0.38 |
| trappc5 | 0.38 |
| ankrd44 | 0.379 |
| wibg | 0.378 |
| nup160 | 0.378 |
| edc3 | 0.378 |
| eif3g | 0.378 |
| txnl4b | 0.378 |
| snrpd3 | 0.377 |
| eif5b | 0.377 |
| nup54 | 0.376 |
| wbp11 | 0.376 |
| dhx38 | 0.376 |
| ndufa11 | 0.375 |
| eif3c | 0.374 |
| psmc6 | 0.374 |
| pop1 | 0.374 |
| rtf1 | 0.373 |
| 0610038d11rik | 0.373 |
| cnot1 | 0.372 |
| prpf4 | 0.372 |
| trappc6b | 0.372 |
| med31 | 0.371 |
| gins2 | 0.371 |
| timm10 | 0.371 |
| mtx2 | 0.371 |
| cpsf6 | 0.371 |
| larp2 | 0.371 |
| thoc7 | 0.371 |
| lsm4 | 0.37 |
| srp54a | 0.37 |
| srp14 | 0.369 |
| akap6 | 0.369 |
| eif3d | 0.369 |
| mphosph10 | 0.369 |
| thoc2 | 0.369 |
| chrac1 | 0.369 |
| ndufs6 | 0.369 |
| rfxap | 0.368 |
| 6330439k17rik | 0.368 |
| dda1 | 0.367 |
| cops4 | 0.367 |
| sf3b1 | 0.367 |
| snrpd1 | 0.366 |
| cog1 | 0.366 |
| timm13 | 0.366 |
| dhx29 | 0.365 |
| smc2 | 0.365 |
| ncapd2 | 0.364 |
| zfp473 | 0.364 |
| vps29 | 0.364 |
| paf1 | 0.363 |
| isy1 | 0.363 |
| ap4b1 | 0.363 |
| rpp21 | 0.362 |
| exosc8 | 0.362 |
| pet112l | 0.362 |
| snrpb2 | 0.362 |
| 4632419k20rik | 0.361 |
| rexo1 | 0.361 |
| cdc26 | 0.36 |
| sec24b | 0.36 |
| cpsf2 | 0.36 |
| aa673488 | 0.36 |
| ncbp2 | 0.359 |
| wdr12 | 0.359 |
| akap8l | 0.359 |
| rpp38 | 0.359 |
| nhp2l1 | 0.359 |
| 4121402d02rik | 0.359 |
| elof1 | 0.359 |
| cops7a | 0.359 |
| kctd5 | 0.358 |
| snrpb | 0.358 |
| srrm2 | 0.358 |
| dhx40 | 0.358 |
| xpo6 | 0.357 |
| copz2 | 0.357 |
| ap4e1 | 0.357 |
| samm50 | 0.356 |
| nupl1 | 0.356 |
| vps26b | 0.356 |
| pole3 | 0.356 |
| orc5l | 0.355 |
| nat13 | 0.355 |
| psmg3 | 0.355 |
| obfc1 | 0.354 |
| 1500010j02rik | 0.354 |
| tram1 | 0.354 |
| med13 | 0.354 |
| polr3e | 0.354 |
| lsm7 | 0.354 |
| sec16a | 0.353 |
| wdr23 | 0.353 |
| spc25 | 0.353 |
| timm17a | 0.353 |
| ssr3 | 0.352 |
| caskin1 | 0.352 |
| psmd14 | 0.351 |
| sec24c | 0.351 |
| cpsf3 | 0.351 |
| lin52 | 0.35 |
| prpf6 | 0.35 |
| tgs1 | 0.35 |
| drap1 | 0.349 |
| polr3f | 0.349 |
| srp9 | 0.349 |
| copg | 0.349 |
| 2610528k11rik | 0.348 |
| cstf1 | 0.348 |
| psmc1 | 0.347 |
| utp18 | 0.347 |
| pcf11 | 0.347 |
| zfp259 | 0.347 |
| ndufs2 | 0.346 |
| baz1a | 0.346 |
| skiv2l2 | 0.346 |
| oxa1l | 0.346 |
| ddx19b | 0.345 |
| 2310005n01rik | 0.344 |
| au014645 | 0.344 |
| hcfc1r1 | 0.344 |
| eif3b | 0.344 |
| brpf3 | 0.344 |
| actr10 | 0.343 |
| rnpc3 | 0.343 |
| tyw3 | 0.343 |
| ddx20 | 0.343 |
| dpy30 | 0.343 |
| strap | 0.343 |
| wdr42a | 0.343 |
| rangrf | 0.342 |
| plrg1 | 0.342 |
| isg20l2 | 0.342 |
| ssr2 | 0.342 |
| sf3a2 | 0.342 |
| wbp4 | 0.341 |
| dctn3 | 0.341 |
| nmd3 | 0.341 |
| prpf18 | 0.341 |
| nploc4 | 0.341 |
| kif13b | 0.34 |
| phf5a | 0.34 |
| sec22b | 0.339 |
| mphosph8 | 0.339 |
| dctn5 | 0.339 |
| dr1 | 0.339 |
| rpo1-4 | 0.339 |
| cpsf1 | 0.338 |
| mett11d1 | 0.338 |
| vps41 | 0.338 |
| bc003885 | 0.338 |
| thoc6 | 0.338 |
| ap4s1 | 0.338 |
| tada1l | 0.338 |
| luc7l2 | 0.337 |
| sec23ip | 0.337 |
| rbm28 | 0.337 |
| zcchc4 | 0.336 |
| lsm12 | 0.336 |
| cenpt | 0.336 |
| ttc35 | 0.336 |
| bxdc2 | 0.335 |
| rpap3 | 0.335 |
| mtif3 | 0.335 |
| arcn1 | 0.335 |
| mcm10 | 0.335 |
| psmc4 | 0.335 |
| srrm1 | 0.335 |
| atp6v0e | 0.335 |
| p42pop | 0.334 |
| smc4 | 0.334 |
| cnot8 | 0.334 |
| pole2 | 0.334 |
| syce1 | 0.334 |
| nxt1 | 0.334 |
| cpsf4 | 0.334 |
| dscc1 | 0.333 |
| nutf2 | 0.333 |
| caskin2 | 0.333 |
| orc6l | 0.333 |
| polr2e | 0.333 |
| snapc3 | 0.333 |
| orc4l | 0.333 |
| psmb3 | 0.333 |
| 0610038f07rik | 0.333 |
| eif3eip | 0.333 |
| sec24a | 0.333 |
| mepce | 0.332 |
| zbtb43 | 0.332 |
| ssr1 | 0.332 |
| timm17b | 0.332 |
| rrp15 | 0.332 |
| zfp335 | 0.332 |
| lin7b | 0.332 |
| usp52 | 0.332 |
| lin7c | 0.331 |
| psmc2 | 0.331 |
| pfdn2 | 0.331 |
| asf1a | 0.331 |
| sip1 | 0.33 |
| naf1 | 0.33 |
| atp6v1d | 0.33 |
| sec11c | 0.33 |
| magoh | 0.33 |
| cpsf3l | 0.33 |
| ints9 | 0.33 |
| bdp1 | 0.33 |
| d12mit143 | 0.33 |
| med24 | 0.33 |
| tceb3 | 0.33 |
| ncaph | 0.329 |
| snrpe | 0.329 |
| orc3l | 0.329 |
| eny2 | 0.329 |
| mrps5 | 0.329 |
| mrps34 | 0.329 |
| arpc5 | 0.329 |
| u2af1 | 0.328 |
| tsen34 | 0.328 |
| rars | 0.328 |
| wdr77 | 0.328 |
| med4 | 0.328 |
| vps35 | 0.328 |
| exosc9 | 0.328 |
| dynlt3 | 0.328 |
| mpp2 | 0.328 |
| tomm20 | 0.327 |
| psma8 | 0.327 |
| inoc1 | 0.327 |
| copb2 | 0.327 |
| 1110007a13rik | 0.326 |
| rer1 | 0.326 |
| cnot4 | 0.326 |
| vgll1 | 0.326 |
| nup85 | 0.326 |
| med17 | 0.326 |
| psmg4 | 0.326 |
| snapc2 | 0.326 |
| lin7a | 0.326 |
| copz1 | 0.326 |
| vps36 | 0.326 |
| ubxd1 | 0.326 |
| akap1 | 0.326 |
| clpx | 0.325 |
| zer1 | 0.325 |
| iars | 0.325 |
| gatad2a | 0.325 |
| loc665506 | 0.325 |
| arpc4 | 0.325 |
| iws1 | 0.325 |
| rpp25 | 0.325 |
| ngly1 | 0.324 |
| pfdn1 | 0.324 |
| puf60 | 0.324 |
| ruvbl1 | 0.324 |
| 5730453i16rik | 0.323 |
| nsfl1c | 0.323 |
| cnot6 | 0.323 |
| wdr48 | 0.323 |
| cox18 | 0.322 |
| snrpc | 0.322 |
| atpaf2 | 0.322 |
| psma2 | 0.322 |
| akap2 | 0.322 |
| lyrm4 | 0.322 |
| etf1 | 0.322 |
| ai413782 | 0.322 |
| leo1 | 0.321 |
| d1mit213 | 0.321 |
| hist1h4a | 0.321 |
| elp2 | 0.321 |
| ndufs8 | 0.321 |
| d230025d16rik | 0.321 |
| ndufb10 | 0.32 |
| rpo1-1 | 0.32 |
| 2310003c23rik | 0.32 |
| poldip3 | 0.32 |
| ndufb7 | 0.32 |
| med20 | 0.32 |
| ssu72 | 0.32 |
| d19ertd721e | 0.32 |
| 6330569m22rik | 0.32 |
| d330017j20rik | 0.32 |
| thoc3 | 0.32 |
| lin54 | 0.32 |
| actl6a | 0.319 |
| chmp1b | 0.319 |
| psmc1-rs1 | 0.319 |
| elp3 | 0.319 |
| copa | 0.319 |
| yipf7 | 0.319 |
| mpp5 | 0.318 |
| srpr | 0.318 |
| psmb6 | 0.318 |
| polr2l | 0.318 |
| sfrs18 | 0.318 |
| cct2 | 0.318 |
| polrmt | 0.317 |
| nupl2 | 0.317 |
| thrap3 | 0.317 |
| xpot | 0.317 |
| nfs1 | 0.317 |
| prpf8 | 0.317 |
| f630043a04rik | 0.317 |
| polr3c | 0.317 |
| prim2 | 0.317 |
| nvl | 0.317 |
| mrpl12 | 0.316 |
| syce2 | 0.316 |
| akap9 | 0.316 |
| prpf40a | 0.316 |
| n6amt1 | 0.316 |
| 3110003a22rik | 0.316 |
| sfi1 | 0.316 |
| prpf3 | 0.316 |
| msl2l1 | 0.316 |
| bxdc5 | 0.315 |
| ropn1 | 0.315 |
| polr2c | 0.315 |
| 4930526h21rik | 0.315 |
| afg3l1 | 0.315 |
| ruvbl2 | 0.315 |
| btaf1 | 0.315 |
| rnu11 | 0.315 |
| gpn1 | 0.315 |
| srp72 | 0.314 |
| usp46 | 0.314 |
| polr2h | 0.314 |
| tbcd | 0.314 |
| chchd3 | 0.314 |
| orc1l | 0.314 |
| snrpa1 | 0.314 |
| uchl5ip | 0.314 |
| akap11 | 0.314 |
| u2af2 | 0.313 |
| hnrnpul2 | 0.313 |
| exosc7 | 0.313 |
| dhx8 | 0.313 |
| chmp2a | 0.313 |
| mcm9 | 0.313 |
| refbp2 | 0.313 |
| xpo4 | 0.313 |
| psmd8 | 0.313 |
| cops3 | 0.313 |
| tbca | 0.313 |
| hook2 | 0.313 |
| rp23-100c5.8 | 0.313 |
| ndufa12 | 0.313 |
| supt5h-rs1 | 0.312 |
| nolc1 | 0.312 |
| gabpb1 | 0.312 |
| bicd2 | 0.312 |
| psmd2 | 0.312 |
| trak2 | 0.312 |
| tmed3 | 0.312 |
| snrp2a | 0.312 |
| polr2b | 0.311 |
| clns1a | 0.311 |
| hcfc2 | 0.311 |
| cnot3 | 0.311 |
| actl6b | 0.311 |
| saps2 | 0.311 |
| psmd7 | 0.311 |
| polr2i | 0.311 |
| dync1li2 | 0.311 |
| ift80 | 0.311 |
| arpm1 | 0.311 |
| cnot2 | 0.311 |
| copg2 | 0.311 |
| chchd6 | 0.31 |
| urm1 | 0.31 |
| 5730427n09rik | 0.31 |
| rps7 | 0.31 |
| pan3 | 0.31 |
| eprs | 0.31 |
| dlgap1 | 0.31 |
| dars | 0.31 |
| yif1a | 0.31 |
| rfxank | 0.31 |
| emg1 | 0.31 |
| brpf1 | 0.31 |
| anapc10 | 0.309 |
| gspt2 | 0.309 |
| wdr82 | 0.309 |
| uso1 | 0.309 |
| c80913 | 0.309 |
| rbm15b | 0.309 |
| 7-Sep | 0.309 |
| psma3 | 0.309 |
| psmb7 | 0.309 |
| ahsa2 | 0.309 |
| nup155 | 0.309 |
| nup210 | 0.309 |
| tomm34 | 0.308 |
| prr6 | 0.308 |
| rae1 | 0.308 |
| chchd4 | 0.308 |
| psmd1 | 0.308 |
| ndufb5 | 0.308 |
| ndufc1 | 0.308 |
| dctn4 | 0.308 |
| gatad2b | 0.308 |
| eif4a3 | 0.308 |
| kif2b | 0.308 |
| scfd1 | 0.308 |
| snapc4 | 0.307 |
| golt1a | 0.307 |
| bcap29 | 0.307 |
| tomm70a | 0.307 |
| thoc4 | 0.307 |
| sdcbp2 | 0.306 |
| tbc1d13 | 0.306 |
| eif3k | 0.306 |
| rusc2 | 0.306 |
| esf1 | 0.306 |
| mpp3 | 0.306 |
| med23 | 0.306 |
| ndufa2 | 0.306 |
| esco1 | 0.306 |
| cask | 0.306 |
| mrps16 | 0.306 |
| cox19 | 0.306 |
| sco1 | 0.306 |
| nsmce4a | 0.306 |
| alg14 | 0.305 |
| 9430023l20rik | 0.305 |
| klc2 | 0.305 |
| psmb2 | 0.305 |
| rfc5 | 0.305 |
| zc3h3 | 0.305 |
| cep135 | 0.305 |
| pir | 0.305 |
| rrp9 | 0.305 |
| smg6 | 0.304 |
| eg636544 | 0.304 |
| sart3 | 0.304 |
| ncaph2 | 0.304 |
| btf3 | 0.304 |
| in(17)3t | 0.304 |
| in(17)2t | 0.304 |
| unc84a | 0.304 |
| gorasp2 | 0.303 |
| mapre2 | 0.303 |
| ift172 | 0.303 |
| cops8 | 0.303 |
| ndufs3 | 0.303 |
| nxf1 | 0.303 |
| anapc5 | 0.303 |
| golgb1 | 0.302 |
| cope | 0.302 |
| nbea | 0.302 |
| 1200014m14rik | 0.302 |
| inadl | 0.302 |
| cdc40 | 0.302 |
| 9130404d08rik | 0.302 |
| bc017158 | 0.302 |
| timm44 | 0.302 |
| hnrnpul1 | 0.302 |
| trip11 | 0.301 |
| ppp2r3a | 0.301 |
| mpp1 | 0.301 |
| ai837181 | 0.301 |
| ndufa6 | 0.301 |
| cct7 | 0.301 |
| mapre3 | 0.301 |
| psmc3 | 0.301 |
| yipf5 | 0.301 |
| rbm8a | 0.301 |
| dynll2 | 0.301 |
| exoc8 | 0.301 |
| pom121 | 0.3 |
| coro7 | 0.3 |
| wdhd1 | 0.3 |
| aa474455 | 0.3 |
| rpp30 | 0.3 |
| chaf1b | 0.3 |
| bloc1s1 | 0.3 |
| d6mit33 | 0.3 |
| eif5 | 0.3 |
| shank3 | 0.3 |
| snapc1 | 0.3 |
| vps18 | 0.299 |
| abcf1 | 0.299 |
| casc3 | 0.299 |
| nubp2 | 0.299 |
| zmynd11 | 0.299 |
| crnkl1 | 0.299 |
| shank1 | 0.299 |
| chd3 | 0.299 |
| ipo7 | 0.299 |
| tada2l | 0.299 |
| arpc3 | 0.299 |
| psmc5 | 0.299 |
| ncapg2 | 0.299 |
| hat1 | 0.298 |
| upf3a | 0.298 |
| stx5a | 0.298 |
| pfdn4 | 0.298 |
| fxc1 | 0.298 |
| 1700007i08rik | 0.298 |
| med27 | 0.298 |
| ubqln2 | 0.298 |
| d2ertd391e | 0.298 |
| vprbp | 0.298 |
| prim1 | 0.298 |
| sec24d | 0.298 |
| tceb2 | 0.298 |
| gatc | 0.298 |
| ears2 | 0.298 |
| qrsl1 | 0.298 |
| sec23a | 0.298 |
| lin37 | 0.298 |
| fsip2 | 0.297 |
| fsip1 | 0.297 |
| dctn2 | 0.297 |
| rplp1 | 0.297 |
| arpc1a | 0.297 |
| dcp1b | 0.297 |
| orc2l | 0.297 |
| lsm2 | 0.297 |
| mapbpip | 0.297 |
| ssbp3 | 0.297 |
| sap130 | 0.297 |
| grasp | 0.297 |
| ssna1 | 0.297 |
| cox11 | 0.297 |
| tmed2 | 0.297 |
| vps26a | 0.296 |
| med30 | 0.296 |
| mpp7 | 0.296 |
| uchl5 | 0.296 |
| asb10 | 0.296 |
| asb14 | 0.296 |
| asb16 | 0.296 |
| asb13 | 0.296 |
| asb18 | 0.296 |
| rint1 | 0.296 |
| nrm | 0.296 |
| polr2f | 0.296 |
| ahctf1 | 0.296 |
| riok2 | 0.296 |
| cript | 0.296 |
| pigk | 0.296 |
| nkain3 | 0.296 |
| nkain1 | 0.296 |
| rnu12 | 0.296 |
| mtrf1l | 0.296 |
| pigy | 0.296 |
| ppil1 | 0.296 |
| psmb1 | 0.296 |
| d130059p03rik | 0.295 |
| arfgef1 | 0.295 |
| ptcd3 | 0.295 |
| cct6a | 0.295 |
| pigs | 0.295 |
| smg7 | 0.295 |
| pmf1 | 0.295 |
| actr5 | 0.295 |
| atxn7l1 | 0.295 |
| atxn7l3 | 0.295 |
| exoc1 | 0.295 |
| clpp | 0.295 |
| dnajc19 | 0.295 |
| baz1b | 0.295 |
| supt7l | 0.295 |
| smg5 | 0.295 |
| nufip1 | 0.294 |
| prkar2a | 0.294 |
| trspap1 | 0.294 |
| cotl1 | 0.294 |
| pola2 | 0.294 |
| mcm8 | 0.294 |
| nup153 | 0.294 |
| atp5s | 0.294 |
| ndufs7 | 0.294 |
| coil | 0.294 |
| u2af1l4 | 0.294 |
| polr2g | 0.294 |
| mical3 | 0.294 |
| prmt5 | 0.293 |
| cops6 | 0.293 |
| stt3b | 0.293 |
| cnksr3 | 0.293 |
| upf3b | 0.293 |
| copb1 | 0.293 |
| pole4 | 0.293 |
| snf8 | 0.293 |
| vcpip1 | 0.293 |
| tmem149 | 0.293 |
| ncapg | 0.293 |
| oma1 | 0.292 |
| gins1 | 0.292 |
| psmb4 | 0.292 |
| uqcrc2 | 0.292 |
| cdc5l | 0.292 |
| 1110008l16rik | 0.292 |
| ndufaf2 | 0.292 |
| gtf2h2 | 0.292 |
| 2410018c20rik | 0.291 |
| ubr4 | 0.291 |
| vps25 | 0.291 |
| poldip2 | 0.291 |
| thex1 | 0.291 |
| taf2 | 0.291 |
| actr1a | 0.291 |
| xmv45 | 0.29 |
| 4930418g15rik | 0.29 |
| psme4 | 0.29 |
| epb4.1l2 | 0.29 |
| ranbp3 | 0.29 |
| nsl1 | 0.29 |
| mterf | 0.29 |
| gm1040 | 0.29 |
| gosr1 | 0.29 |
| cct3 | 0.29 |
| supv3l1 | 0.29 |
| d12mit156 | 0.29 |
| pigyl | 0.29 |
| ccdc101 | 0.29 |
| dhx15 | 0.289 |
| qars | 0.289 |
| 1110020p15rik | 0.289 |
| trpc4ap | 0.289 |
| tsfm | 0.289 |
| gm672 | 0.289 |
| ankrd28 | 0.289 |
| chaf1a | 0.289 |
| tax1bp3 | 0.288 |
| rcc1 | 0.288 |
| 2810452k22rik | 0.288 |
| gtf2e2 | 0.288 |
| av249152 | 0.288 |
| exoc2 | 0.288 |
| 2510003e04rik | 0.288 |
| ap4m1 | 0.288 |
| ipo9 | 0.288 |
| 1810035l17rik | 0.288 |
| tdrkh | 0.288 |
| cand1 | 0.288 |
| chtf18 | 0.288 |
| zc3h7b | 0.288 |
| sec22a | 0.287 |
| spg21 | 0.287 |
| vkorc1l1 | 0.287 |
| snrpa | 0.287 |
| trmt6 | 0.287 |
| dlg1 | 0.287 |
| zcchc6 | 0.287 |
| bet1 | 0.286 |
| arl2 | 0.286 |
| nup62 | 0.286 |
| sap18 | 0.286 |
| nxf7 | 0.286 |
| akap7 | 0.286 |
| ai314180 | 0.285 |
| tnpo3 | 0.285 |
| pold2 | 0.285 |
| mcm3 | 0.285 |
| cnksr2 | 0.285 |
| dlgap3 | 0.285 |
| chd1 | 0.285 |
| 2810433k01rik | 0.285 |
| oip5 | 0.285 |
| pigu | 0.285 |
| c8g | 0.285 |
| whdc1 | 0.285 |
| vps11 | 0.285 |
| ars2 | 0.284 |
| eif6 | 0.284 |
| tbkbp1 | 0.284 |
| cecr2 | 0.284 |
| ranbp1 | 0.284 |
| ankrd12 | 0.284 |
| atp6v1f | 0.284 |
| zw10 | 0.284 |
| akap4 | 0.284 |
| rpo1-2 | 0.284 |
| mobkl3 | 0.284 |
| gosr2 | 0.284 |
| trim67 | 0.284 |
| nup214 | 0.284 |
| ctr9 | 0.283 |
| 6720458f09rik | 0.283 |
| akap3 | 0.283 |
| trim46 | 0.283 |
| pmpca | 0.283 |
| dtnb | 0.283 |
| arfgef2 | 0.283 |
| rpa3 | 0.283 |
| snx2 | 0.283 |
| sec23b | 0.283 |
| tbcb | 0.283 |
| cenpn | 0.282 |
| dcp1a | 0.282 |
| ctdp1 | 0.282 |
| arl2bp | 0.282 |
| cdk8 | 0.282 |
| 8430415e04rik | 0.282 |
| rfc2 | 0.282 |
| kars | 0.282 |
| qsm | 0.282 |
| klhl17 | 0.282 |
| acbd3 | 0.282 |
| dsn1 | 0.282 |
| rpn1 | 0.282 |
| snrp70 | 0.282 |
| dlg3 | 0.282 |
| exoc4 | 0.281 |
| loc100043597 | 0.281 |
| dlg2 | 0.281 |
| ppme1 | 0.281 |
| sec61g | 0.281 |
| tufm | 0.281 |
| ppig | 0.281 |
| nxf2 | 0.281 |
| snora74a | 0.281 |
| cct6b | 0.281 |
| d7mit133 | 0.281 |
| 2700060e02rik | 0.281 |
| fars2 | 0.281 |
| trak1 | 0.281 |
| nek9 | 0.281 |
| mrrf | 0.281 |
| gorasp1 | 0.281 |
| wdr40a | 0.28 |
| cul2 | 0.28 |
| wac | 0.28 |
| tut1 | 0.28 |
| habp4 | 0.28 |
| saps3 | 0.28 |
| sipa1l1 | 0.28 |
| prpf19 | 0.28 |
| cct8 | 0.28 |
| med16 | 0.28 |
| setd1b | 0.28 |
| tfb1m | 0.28 |
| ndufa9 | 0.28 |
| gps1 | 0.28 |
| dmap1 | 0.279 |
| cox7b | 0.279 |
| rfc4 | 0.279 |
| golga4 | 0.279 |
| rnmt | 0.279 |
| cdc45l | 0.279 |
| rfc3 | 0.279 |
| lrrc1 | 0.279 |
| d14ertd436e | 0.279 |
| ipo13 | 0.279 |
| txndc9 | 0.279 |
| tnrc6b | 0.279 |
| cbx6 | 0.279 |
| nup50 | 0.278 |
| cmya5 | 0.278 |
| xpo5 | 0.278 |
| paip1 | 0.278 |
| jarid1a | 0.278 |
| ndufb4 | 0.278 |
| psma5 | 0.278 |
| rbm25 | 0.278 |
| det1 | 0.278 |
| saps1 | 0.278 |
| sec61b | 0.278 |
| immp1l | 0.277 |
| dctn6 | 0.277 |
| akap5 | 0.277 |
| mycbp | 0.277 |
| uqcrc1 | 0.277 |
| sf4 | 0.277 |
| ppp1r3f | 0.277 |
| nat5 | 0.277 |
| mtrf1 | 0.277 |
| 1700001g17rik | 0.277 |
| hist1h2bp | 0.277 |
| eaf1 | 0.276 |
| hcfc1 | 0.276 |
| exosc10 | 0.276 |
| klc3 | 0.276 |
| gopc | 0.276 |
| pdzd11 | 0.276 |
| 9130227c08rik | 0.276 |
| pbrm1 | 0.276 |
| fbl | 0.276 |
| baz2a | 0.276 |
| ddx11 | 0.276 |
| patl1 | 0.276 |
| patl2 | 0.276 |
| ylpm1 | 0.276 |
| tceb1 | 0.276 |
| rpl18a | 0.275 |
| tbcc | 0.275 |
| arid2 | 0.275 |
| golga5 | 0.275 |
| atpaf1 | 0.275 |
| psmg1 | 0.275 |
| supt6h | 0.275 |
| c8a | 0.275 |
| sfrs11 | 0.275 |
| mpp4 | 0.275 |
| ubxd2 | 0.275 |
| ddx54 | 0.275 |
| shank2 | 0.275 |
| taf1a | 0.275 |
| 1700021k19rik | 0.275 |
| shfm1 | 0.274 |
| iqsec2 | 0.274 |
| asb9 | 0.274 |
| smc5 | 0.274 |
| mtch1 | 0.274 |
| fkbp8 | 0.274 |
| hnrnpr | 0.274 |
| yaf2 | 0.274 |
| pld4 | 0.274 |
| zwint | 0.274 |
| thoc1 | 0.274 |
| sfrs2ip | 0.274 |
| mis12 | 0.274 |
| atad3a | 0.274 |
| ndufv1 | 0.274 |
| cops2 | 0.274 |
| upf2 | 0.274 |
| tmem151b | 0.273 |
| dync1i1 | 0.273 |
| cenpk | 0.273 |
| eif3e | 0.273 |
| polg2 | 0.273 |
| edc4 | 0.273 |
| setd1a | 0.273 |
| arpc1b | 0.273 |
| zbtb6 | 0.273 |
| nxf3 | 0.273 |
| d7mit357 | 0.273 |
| golga1 | 0.273 |
| lsm1 | 0.273 |
| 2600010e01rik | 0.273 |
| gspt1 | 0.273 |
| tor1aip1 | 0.273 |
| slbp | 0.273 |
| bloc1s2 | 0.273 |
| ppp4r1 | 0.273 |
| mpp6 | 0.272 |
| ppp1r8 | 0.272 |
| snora65 | 0.272 |
| snora70 | 0.272 |
| snora64 | 0.272 |
| ssr4 | 0.272 |
| begain | 0.272 |
| erbb2ip | 0.272 |
| wdr35 | 0.272 |
| vps16 | 0.272 |
| yipf1 | 0.272 |
| hirip3 | 0.272 |
| eif4enif1 | 0.272 |
| psme2 | 0.272 |
| tcea1 | 0.271 |
| papola | 0.271 |
| med21 | 0.271 |
| snora68 | 0.271 |
| nap1l1 | 0.271 |
| polr2j | 0.271 |
| mospd2 | 0.271 |
| mospd1 | 0.271 |
| smc6 | 0.271 |
| smarcc2 | 0.271 |
| apba1 | 0.271 |
| ap3s2 | 0.271 |
| sart1 | 0.271 |
| rpl23a | 0.271 |
| apba3 | 0.271 |
| gtf2e1 | 0.271 |
| atp6v1h | 0.271 |
| chd4 | 0.271 |
| polr3a | 0.271 |
| gcc1 | 0.271 |
| sntg1 | 0.27 |
| 2900092e17rik | 0.27 |
| homer3 | 0.27 |
