# Supplementary material for: GIT2 Acts as a Potential Keystone Protein in Functional Hypothalamic Networks Associated with Age-Related Phenotypic Changes in Rats
Source: PLoS One. 2012 May 14;7(5):e36975. doi: 10.1371/journal.pone.0036975 (PMC3351446; doi:10.1371/journal.pone.0036975)
Supplement: Table S27 — GeneIndexer latent semantic indexing (LSI) of significantly-regulated ‘Nitric oxide synthase activity’ GO term group. Using the GO term group ‘Nitric oxide synthase activity’ as an input term, a list of the top 1000 implicitly-correlated (LSI correlation score >0.1) was generated using a full genome background list. (DOC) [file pone.0036975.s031.doc]

**Table S27. GeneIndexer latent semantic indexing (LSI) of significantly-regulated ‘Nitric oxide synthase activity’ GO term group.** Using the GO term group ‘Nitric oxide synthase activity’ as an input term, a list of the top 1000 implicitly-correlated (LSI correlation score >0.1) was generated using a full genome background list.

| ***Nitric oxide synthase activity*** |  |
| --- | --- |
|  |  |
| **Protein Symbol** | **LSI correlation score** |
| 2610024g14rik | 0.764 |
| nosip | 0.681 |
| zdhhc23 | 0.533 |
| stzid2 | 0.508 |
| hph1 | 0.491 |
| arg2 | 0.487 |
| ddah2 | 0.485 |
| cypt13 | 0.479 |
| cypt14 | 0.479 |
| cypt15 | 0.479 |
| cypt8 | 0.479 |
| cypt9 | 0.479 |
| cypt10 | 0.479 |
| cypt11 | 0.479 |
| cypt7 | 0.479 |
| nostrin | 0.477 |
| ddah1 | 0.473 |
| gucy1b2 | 0.465 |
| hsp86-ps2 | 0.456 |
| slc7a2 | 0.454 |
| gucy1b3 | 0.446 |
| gucy1a3 | 0.443 |
| gucy1a2 | 0.43 |
| pgm2l1 | 0.428 |
| arg1 | 0.422 |
| stzid | 0.422 |
| d6mit291 | 0.413 |
| gchfr | 0.412 |
| ndufb4 | 0.411 |
| cmv3 | 0.407 |
| mirn146 | 0.395 |
| 2810405k02rik | 0.386 |
| gch1 | 0.382 |
| thnsl1 | 0.377 |
| nmral1 | 0.377 |
| slc7a1 | 0.376 |
| cyp2d12 | 0.376 |
| cyp2d13 | 0.376 |
| mgst3 | 0.371 |
| clec14a | 0.365 |
| ogdhl | 0.361 |
| d2ertd750e | 0.358 |
| samd8 | 0.357 |
| nanos2 | 0.352 |
| ass1 | 0.352 |
| trip13 | 0.351 |
| oxsm | 0.35 |
| pde5a | 0.349 |
| pgs1 | 0.342 |
| hmox2 | 0.338 |
| nanos3 | 0.337 |
| accs | 0.337 |
| d6mit135 | 0.337 |
| mgst2 | 0.332 |
| ahsa1 | 0.327 |
| pde2a | 0.326 |
| rab26 | 0.326 |
| ndor1 | 0.325 |
| prkg2 | 0.324 |
| smoc1 | 0.324 |
| prkg1 | 0.32 |
| d11nds1 | 0.319 |
| nans | 0.315 |
| tmem70 | 0.315 |
| asl | 0.311 |
| slc7a3 | 0.311 |
| pts | 0.306 |
| rlf | 0.305 |
| atg9b | 0.302 |
| pus3 | 0.3 |
| atp5j | 0.299 |
| nos1ap | 0.298 |
| sntg2 | 0.297 |
| myoz3 | 0.297 |
| mirn223 | 0.295 |
| ilvbl | 0.294 |
| thnsl2 | 0.292 |
| cnrip1 | 0.292 |
| ndufa6 | 0.29 |
| srm | 0.29 |
| centg3 | 0.288 |
| ate1 | 0.288 |
| ptgis | 0.286 |
| eg594843 | 0.285 |
| ndufa12 | 0.283 |
| rasd1 | 0.283 |
| xdh | 0.283 |
| zbtb7c | 0.281 |
| dpm2 | 0.281 |
| dynll1 | 0.276 |
| dhdds | 0.275 |
| spr | 0.272 |
| mb | 0.272 |
| trim7 | 0.269 |
| trub1 | 0.261 |
| trub2 | 0.261 |
| slc6a14 | 0.26 |
| snta1 | 0.258 |
| klk1b1 | 0.258 |
| lias | 0.257 |
| isyna1 | 0.255 |
| hnrnpl | 0.255 |
| pdss1 | 0.254 |
| dhps | 0.252 |
| sntb1 | 0.25 |
| mgea5 | 0.249 |
| dynll2 | 0.248 |
| nags | 0.248 |
| mrvi1 | 0.248 |
| uts2d | 0.247 |
| 1300018i17rik | 0.247 |
| adm2 | 0.246 |
| cpb1 | 0.246 |
| alas1 | 0.246 |
| gsbs | 0.245 |
| mocs3 | 0.245 |
| cdipt | 0.245 |
| cps1 | 0.244 |
| dpm3 | 0.244 |
| cs | 0.243 |
| cyp4a12a | 0.24 |
| sntb2 | 0.24 |
| aldh18a1 | 0.24 |
| kynu | 0.237 |
| idd4 | 0.237 |
| hmgcs1 | 0.236 |
| trem5 | 0.236 |
| trem4 | 0.236 |
| mpmv2 | 0.235 |
| aco2 | 0.233 |
| cct7 | 0.233 |
| svs4 | 0.231 |
| dohh | 0.231 |
| tabw2 | 0.23 |
| ppp2r5a | 0.23 |
| tst | 0.228 |
| hmgn1-rs18 | 0.228 |
| hmgn1-rs19 | 0.228 |
| synpr | 0.228 |
| b3gnt5 | 0.226 |
| ppp1r12b | 0.224 |
| dlgap2 | 0.224 |
| akr1c19 | 0.224 |
| atp5d | 0.222 |
| cyp2c44 | 0.222 |
| adra1d | 0.221 |
| cds2 | 0.221 |
| garnl4 | 0.22 |
| dpm1 | 0.219 |
| aasdhppt | 0.218 |
| ptges | 0.218 |
| ggps1 | 0.218 |
| cox5a | 0.218 |
| calr2 | 0.217 |
| noan1 | 0.216 |
| d5mit409 | 0.216 |
| noan2 | 0.216 |
| zit1 | 0.216 |
| far2 | 0.216 |
| far1 | 0.216 |
| uts2r | 0.215 |
| ly6g6d | 0.215 |
| cbs | 0.215 |
| alad | 0.215 |
| aass | 0.215 |
| pigq | 0.215 |
| d6mit59 | 0.214 |
| wdr68 | 0.214 |
| idi1 | 0.214 |
| nox1 | 0.213 |
| sms | 0.213 |
| dlgap1 | 0.212 |
| odc1 | 0.212 |
| ngb | 0.212 |
| 5430435g22rik | 0.212 |
| upb1 | 0.211 |
| mcat | 0.211 |
| ctps | 0.211 |
| pkig | 0.21 |
| adc | 0.21 |
| sod3 | 0.21 |
| atpif1 | 0.209 |
| rgn | 0.209 |
| stub1 | 0.208 |
| adcy10 | 0.208 |
| chsy1 | 0.207 |
| atp5e | 0.207 |
| pus1 | 0.207 |
| atg9a | 0.206 |
| zcchc8 | 0.206 |
| iapls2-5 | 0.205 |
| iapls3-12 | 0.205 |
| gatm | 0.205 |
| pcbd1 | 0.205 |
| sntg1 | 0.205 |
| mocs2 | 0.204 |
| alas2 | 0.204 |
| adnp | 0.204 |
| hbb | 0.203 |
| olr1 | 0.203 |
| prmt2 | 0.203 |
| ogdh | 0.203 |
| atp2b4 | 0.203 |
| rpp21 | 0.202 |
| cyp4a14 | 0.202 |
| lass3 | 0.202 |
| apln | 0.202 |
| smpd3 | 0.201 |
| dlgap4 | 0.201 |
| amd1 | 0.2 |
| ang2 | 0.2 |
| clip2 | 0.2 |
| agmat | 0.2 |
| centg2 | 0.2 |
| paox | 0.199 |
| ptgds2 | 0.199 |
| azin1 | 0.199 |
| atp5g2 | 0.198 |
| pigp | 0.198 |
| atp5l | 0.198 |
| nppc | 0.198 |
| 1700014n06rik | 0.198 |
| tbxas1 | 0.198 |
| mthfd1l | 0.198 |
| cyp4a12b | 0.197 |
| oaz2 | 0.197 |
| dtna | 0.196 |
| pmv12 | 0.196 |
| d11mit325 | 0.195 |
| cpd | 0.195 |
| 2010209o12rik | 0.195 |
| smox | 0.195 |
| ppp1r12a | 0.195 |
| tlcd2 | 0.194 |
| hba | 0.194 |
| cth | 0.194 |
| b4galt6 | 0.194 |
| atpaf2 | 0.193 |
| uxs1 | 0.193 |
| adsl | 0.193 |
| pigf | 0.192 |
| nfs1 | 0.192 |
| b3galt6 | 0.192 |
| nrf1 | 0.192 |
| indo | 0.191 |
| b3galt4 | 0.191 |
| zc3h12b | 0.191 |
| npr3 | 0.19 |
| dlg2 | 0.19 |
| atp5g3 | 0.19 |
| ptdss1 | 0.189 |
| ndufs8 | 0.189 |
| grina | 0.189 |
| agtrap | 0.188 |
| lass5 | 0.188 |
| uros | 0.187 |
| pkib | 0.187 |
| npr1 | 0.187 |
| d3mit106 | 0.187 |
| gulo | 0.187 |
| vip | 0.187 |
| a3galt2 | 0.186 |
| cav3 | 0.186 |
| sgms1 | 0.185 |
| gyg | 0.185 |
| urm1 | 0.185 |
| edf1 | 0.185 |
| cygb | 0.185 |
| gtpbp1 | 0.184 |
| atp5f1 | 0.184 |
| ptgs1 | 0.184 |
| sucla2 | 0.183 |
| prdx3 | 0.183 |
| slc5a2 | 0.183 |
| fech | 0.182 |
| sephs1 | 0.182 |
| mthfd2 | 0.182 |
| aif1 | 0.182 |
| camk1 | 0.182 |
| qdpr | 0.182 |
| dus2l | 0.182 |
| t(4;17)2lws | 0.181 |
| t(4;17)2lws | 0.181 |
| irf1 | 0.181 |
| alox15 | 0.181 |
| gnmt | 0.181 |
| pigc | 0.181 |
| hmgcs2 | 0.181 |
| gls | 0.181 |
| gpr182 | 0.181 |
| rock2 | 0.18 |
| b3galnt1 | 0.18 |
| dlgap3 | 0.179 |
| d12mit136 | 0.179 |
| aco1 | 0.179 |
| ncf1 | 0.179 |
| vps54 | 0.179 |
| mgst1 | 0.179 |
| oplah | 0.179 |
| sgms2 | 0.178 |
| ndufab1 | 0.178 |
| pmvk | 0.178 |
| gys2 | 0.178 |
| cox4i1 | 0.178 |
| nkrf | 0.178 |
| atpaf1 | 0.178 |
| cmpk2 | 0.177 |
| has3 | 0.177 |
| tyms-ps | 0.177 |
| atp5c1 | 0.177 |
| atp5s | 0.177 |
| lpsi2 | 0.177 |
| lpsi1 | 0.177 |
| oaz-ps | 0.177 |
| papss1 | 0.176 |
| cds1 | 0.176 |
| alox12 | 0.176 |
| slc11a1 | 0.176 |
| cyb5r3 | 0.176 |
| d13mit1 | 0.176 |
| dcps | 0.176 |
| agtr1b | 0.176 |
| atp5k | 0.176 |
| ephx2 | 0.175 |
| atp5o | 0.175 |
| ren2 | 0.175 |
| coasy | 0.175 |
| chpf | 0.175 |
| pus10 | 0.174 |
| irg1 | 0.174 |
| adcy6 | 0.174 |
| nrgn | 0.173 |
| agtr2 | 0.173 |
| gart | 0.173 |
| st6galnac5 | 0.173 |
| ptprn | 0.173 |
| nos1 | 0.173 |
| atp5a1 | 0.173 |
| dhodh | 0.172 |
| fstl1 | 0.172 |
| gclm | 0.172 |
| uts2 | 0.172 |
| ptdss2 | 0.172 |
| klf2 | 0.172 |
| agps | 0.172 |
| ndufs2 | 0.172 |
| nox4 | 0.172 |
| a4galt | 0.172 |
| mgll | 0.171 |
| atp5g1 | 0.171 |
| vasp | 0.171 |
| hsp90aa1 | 0.171 |
| aoc3 | 0.171 |
| htr1d | 0.171 |
| cdo1 | 0.17 |
| bhmt2 | 0.17 |
| atp5b | 0.17 |
| ppp1r3d | 0.169 |
| cybb | 0.169 |
| hmbs | 0.169 |
| ltc4s | 0.169 |
| slc7a8 | 0.169 |
| ppapdc2 | 0.169 |
| ppcdc | 0.169 |
| ly6g6c | 0.168 |
| adra1b | 0.168 |
| npr2 | 0.168 |
| lss | 0.168 |
| cmas | 0.167 |
| fdps | 0.167 |
| csgalnact1 | 0.167 |
| rln1 | 0.166 |
| mocs1 | 0.166 |
| add1 | 0.166 |
| tbxa2r | 0.165 |
| prkaa2 | 0.165 |
| prmt3 | 0.165 |
| cyba | 0.165 |
| rxfp4 | 0.165 |
| dock7 | 0.165 |
| d1mit293 | 0.165 |
| zc3h12c | 0.165 |
| mdh2 | 0.165 |
| pdlim3 | 0.165 |
| sgcb | 0.165 |
| ptges2 | 0.164 |
| amd2 | 0.164 |
| glrx5 | 0.164 |
| adm | 0.164 |
| sgcg | 0.164 |
| angptl6 | 0.164 |
| tuba1c | 0.164 |
| tyw3 | 0.163 |
| pycr1 | 0.163 |
| ppp1r3b | 0.163 |
| prkaa1 | 0.163 |
| sgcd | 0.163 |
| mpo | 0.163 |
| b3gnt6 | 0.163 |
| edn2 | 0.163 |
| tis | 0.163 |
| gys1 | 0.162 |
| dtymk | 0.162 |
| slc29a1 | 0.162 |
| pkia | 0.162 |
| el1 | 0.162 |
| oaz3 | 0.162 |
| syn3 | 0.162 |
| eif5a | 0.162 |
| rgs17 | 0.162 |
| dusp16 | 0.161 |
| abcb5 | 0.161 |
| dgat2l4 | 0.161 |
| mst1r | 0.16 |
| pik3c3 | 0.16 |
| mas1 | 0.16 |
| immp2l | 0.16 |
| decr1 | 0.16 |
| cpox | 0.16 |
| gsta4 | 0.16 |
| atp5j2 | 0.16 |
| tg(tek-cre)12flv | 0.16 |
| slc39a14 | 0.159 |
| d4mit289 | 0.159 |
| d4mit324 | 0.159 |
| ppcs | 0.159 |
| shmt2 | 0.158 |
| cyb5r4 | 0.157 |
| pygl | 0.157 |
| prmt7 | 0.157 |
| pepd | 0.157 |
| ncf2 | 0.157 |
| sgca | 0.157 |
| kcnn3 | 0.157 |
| cad | 0.156 |
| tyw1 | 0.156 |
| srxn1 | 0.156 |
| mosc1 | 0.156 |
| qtrt1 | 0.156 |
| sat2 | 0.156 |
| vac14 | 0.156 |
| prkab1 | 0.156 |
| oat | 0.155 |
| txn2 | 0.155 |
| crls1 | 0.155 |
| srr | 0.155 |
| hsd11b2 | 0.155 |
| grin2c | 0.155 |
| bcat2 | 0.155 |
| ren1 | 0.155 |
| has2 | 0.154 |
| frat2 | 0.154 |
| glul | 0.154 |
| kcnmb1 | 0.154 |
| ensmusg00000058357 | 0.154 |
| gapdh | 0.154 |
| cyp4f15 | 0.154 |
| ugcg | 0.154 |
| trim39 | 0.153 |
| gmps | 0.153 |
| abp1 | 0.153 |
| ppp1r3c | 0.153 |
| fdft1 | 0.153 |
| pde3a | 0.153 |
| adra1a | 0.153 |
| alox5 | 0.153 |
| has1 | 0.153 |
| oaz1 | 0.153 |
| igtp | 0.153 |
| prdx2 | 0.152 |
| evi2a | 0.152 |
| rxfp3 | 0.152 |
| wars | 0.152 |
| adss | 0.152 |
| sat1 | 0.152 |
| sptlc2 | 0.152 |
| grin2d | 0.152 |
| akr1b3 | 0.152 |
| lpo | 0.152 |
| dhfr | 0.152 |
| gclc | 0.152 |
| htr2b | 0.151 |
| akt2 | 0.151 |
| syn2 | 0.151 |
| hbegf | 0.151 |
| cab | 0.151 |
| eprs | 0.151 |
| edn3 | 0.151 |
| cyp11b2 | 0.15 |
| ifngr1 | 0.15 |
| fer1l3 | 0.15 |
| umps | 0.15 |
| dnajb9 | 0.15 |
| kmo | 0.15 |
| ece1 | 0.15 |
| irf2 | 0.15 |
| st3gal5 | 0.15 |
| ctps2 | 0.15 |
| sftpa1 | 0.15 |
| mosc2 | 0.15 |
| ccbl1 | 0.149 |
| vnn1 | 0.149 |
| fbxo32 | 0.149 |
| rcan1 | 0.149 |
| sdha | 0.149 |
| gja4 | 0.149 |
| trem3 | 0.149 |
| epx | 0.149 |
| a230051g13rik | 0.149 |
| tmem14c | 0.149 |
| duox1 | 0.149 |
| b3galt2 | 0.148 |
| cia8 | 0.148 |
| map3k5 | 0.148 |
| scl1 | 0.148 |
| tsp2 | 0.148 |
| tufm | 0.148 |
| bach1 | 0.148 |
| lass1 | 0.148 |
| bpgm | 0.148 |
| prss8 | 0.148 |
| gnb3 | 0.148 |
| nqo1 | 0.147 |
| akr1b7 | 0.147 |
| guk1 | 0.147 |
| calm3 | 0.147 |
| dlg3 | 0.147 |
| dnaic1 | 0.147 |
| slc18a3 | 0.147 |
| 4632434i11rik | 0.147 |
| appl1 | 0.147 |
| tgs1 | 0.146 |
| prdx6 | 0.146 |
| zc3h12d | 0.146 |
| dpysl3 | 0.146 |
| cd59a | 0.145 |
| pnmt | 0.145 |
| mvd | 0.145 |
| ppp1r3e | 0.145 |
| nfkbib | 0.145 |
| rhbdd2 | 0.145 |
| utrn | 0.145 |
| zbed3 | 0.145 |
| dlg4 | 0.145 |
| txnip | 0.145 |
| rtcd1 | 0.145 |
| pah | 0.144 |
| mpst | 0.144 |
| slc6a13 | 0.144 |
| coq2 | 0.144 |
| dtnb | 0.144 |
| fmo1 | 0.144 |
| ptgir | 0.144 |
| hspa4 | 0.144 |
| camk2d | 0.144 |
| gstm4 | 0.144 |
| ambp | 0.144 |
| dyrk2 | 0.143 |
| dusp1 | 0.143 |
| adh5 | 0.143 |
| hspd1 | 0.143 |
| atp2b2 | 0.143 |
| cox1 | 0.142 |
| lass6 | 0.142 |
| lass4 | 0.142 |
| p2ry1 | 0.142 |
| st8sia5 | 0.142 |
| dnajb1 | 0.142 |
| slc30a2 | 0.142 |
| slc6a11 | 0.142 |
| pde9a | 0.142 |
| ctxn3 | 0.142 |
| pla2g6 | 0.141 |
| acly | 0.141 |
| cat | 0.141 |
| bdkrb1 | 0.141 |
| nadsyn1 | 0.141 |
| alox5ap | 0.141 |
| wars2 | 0.141 |
| tdo2 | 0.141 |
| asah2 | 0.141 |
| elavl1 | 0.141 |
| usf2 | 0.141 |
| bcat1 | 0.14 |
| add3 | 0.14 |
| grin3b | 0.14 |
| suox | 0.14 |
| bgn | 0.14 |
| hmgb1-rs7 | 0.14 |
| dnaja1 | 0.14 |
| tars | 0.14 |
| gpd1 | 0.14 |
| gamt | 0.14 |
| otc | 0.139 |
| prl | 0.139 |
| mafg | 0.139 |
| haao | 0.139 |
| miox | 0.139 |
| st8sia1 | 0.139 |
| guca2b | 0.139 |
| ldb3 | 0.139 |
| sptlc1 | 0.139 |
| ptgds | 0.139 |
| ugp2 | 0.139 |
| kcnn4 | 0.139 |
| gstz1 | 0.139 |
| npl | 0.138 |
| rpp30 | 0.138 |
| rcl1 | 0.138 |
| tes3-ps | 0.138 |
| tgm2 | 0.138 |
| slc25a37 | 0.138 |
| rap2a | 0.138 |
| trib3 | 0.138 |
| d12mit37 | 0.138 |
| es3 | 0.138 |
| pln | 0.138 |
| mafa | 0.137 |
| crcp | 0.137 |
| mars2 | 0.137 |
| renbp | 0.137 |
| aplnr | 0.137 |
| nmt1 | 0.137 |
| bbox1 | 0.137 |
| trpm7 | 0.137 |
| ppox | 0.136 |
| adcyap1 | 0.136 |
| gstm2 | 0.136 |
| rxfp1 | 0.136 |
| fn3k | 0.136 |
| rsad2 | 0.136 |
| gsr | 0.136 |
| gramd1a | 0.136 |
| aacs | 0.136 |
| prmt6 | 0.136 |
| nlrp12 | 0.136 |
| alkbh6 | 0.136 |
| alkbh7 | 0.136 |
| alkbh4 | 0.136 |
| strn | 0.136 |
| rfk | 0.135 |
| camkk2 | 0.135 |
| nfatc3 | 0.135 |
| camk4 | 0.135 |
| nox3 | 0.135 |
| actb | 0.135 |
| st6galnac6 | 0.135 |
| eef1a1 | 0.135 |
| ube2d1 | 0.135 |
| esa4 | 0.134 |
| lyz2 | 0.134 |
| ilk | 0.134 |
| sub1 | 0.134 |
| uba1 | 0.134 |
| hrh1 | 0.134 |
| prdx1 | 0.134 |
| atp2a2 | 0.134 |
| scn11a | 0.134 |
| bhmt | 0.134 |
| slc3a1 | 0.133 |
| smpd2 | 0.133 |
| ace2 | 0.133 |
| nos2 | 0.133 |
| tg(k6odctr)55tgo | 0.133 |
| syn1 | 0.133 |
| epo | 0.132 |
| gkap1 | 0.132 |
| gfpt1 | 0.132 |
| gpsn2 | 0.132 |
| duox2 | 0.132 |
| g6pdx | 0.132 |
| acss2 | 0.132 |
| b3galt1 | 0.131 |
| ddx23 | 0.131 |
| pla2g4a | 0.131 |
| ube2e1 | 0.131 |
| nt5dc1 | 0.131 |
| rgs2 | 0.131 |
| hk3 | 0.131 |
| nars | 0.131 |
| sgcz | 0.131 |
| atp6v1a | 0.131 |
| hsf1 | 0.13 |
| mycn | 0.13 |
| bbs12 | 0.13 |
| nudt14 | 0.13 |
| eng | 0.13 |
| kcnma1 | 0.13 |
| umod | 0.13 |
| nr | 0.13 |
| pmv15 | 0.13 |
| smpd1 | 0.13 |
| pdxk | 0.13 |
| prps2 | 0.13 |
| amd-ps1 | 0.13 |
| odc-rs12 | 0.13 |
| hnrnpd | 0.13 |
| gy | 0.13 |
| mthfs | 0.129 |
| mtv35 | 0.129 |
| rock1 | 0.129 |
| itgb1bp2 | 0.129 |
| wisp1 | 0.129 |
| oprl1 | 0.129 |
| calm2 | 0.129 |
| sars | 0.129 |
| cxcl2 | 0.129 |
| txn1 | 0.129 |
| hint1 | 0.129 |
| adora2b | 0.129 |
| ppp1r2 | 0.129 |
| pisd | 0.129 |
| slc25a39 | 0.129 |
| trmt5 | 0.128 |
| cysltr2 | 0.128 |
| abat | 0.128 |
| gucy2g | 0.128 |
| glud1 | 0.128 |
| ogt | 0.128 |
| avp | 0.128 |
| dnahc5 | 0.128 |
| rars | 0.128 |
| gad2 | 0.128 |
| tim | 0.128 |
| nfatc4 | 0.128 |
| mat1a | 0.128 |
| enoph1 | 0.128 |
| glp2r | 0.128 |
| add2 | 0.127 |
| oxa1l | 0.127 |
| klf5 | 0.127 |
| hao1 | 0.127 |
| d6mit55 | 0.127 |
| nppa | 0.127 |
| pnpo | 0.127 |
| nos3 | 0.127 |
| impa1 | 0.127 |
| slc6a6 | 0.127 |
| bdkrb2 | 0.127 |
| hccs | 0.127 |
| csad | 0.127 |
| ihpk1 | 0.127 |
| nfkbia | 0.127 |
| irf8 | 0.127 |
| mlycd | 0.127 |
| pgls | 0.126 |
| tg(otc)94mori | 0.126 |
| spon1l | 0.126 |
| shmt1 | 0.126 |
| prmt1 | 0.126 |
| grin3a | 0.126 |
| ugdh | 0.126 |
| slc6a8 | 0.126 |
| cyp4f14 | 0.126 |
| prcp | 0.126 |
| pigg | 0.126 |
| slc7a5 | 0.126 |
| b4galnt1 | 0.126 |
| crip2 | 0.126 |
| tsn | 0.126 |
| lrrfip2 | 0.126 |
| xpnpep1 | 0.126 |
| aldh1l1 | 0.125 |
| cyp11b1 | 0.125 |
| cnga1 | 0.125 |
| slc30a1 | 0.125 |
| sspn | 0.125 |
| etohila | 0.125 |
| aadat | 0.125 |
| gss | 0.125 |
| ptafr | 0.125 |
| lta4h | 0.125 |
| tms | 0.125 |
| atp2a3 | 0.125 |
| 6530404n21rik | 0.125 |
| csgalnact2 | 0.125 |
| gnpat | 0.125 |
| pfn1 | 0.125 |
| idi2 | 0.125 |
| pgf | 0.125 |
| sardh | 0.125 |
| ltb4r2 | 0.125 |
| atic | 0.125 |
| d17wsu104e | 0.125 |
| pcx | 0.124 |
| begain | 0.124 |
| foxo3a | 0.124 |
| slc25a3 | 0.124 |
| cd47 | 0.124 |
| ednrb | 0.124 |
| jb | 0.124 |
| ramp2 | 0.124 |
| pdpk1 | 0.124 |
| mpdu1 | 0.124 |
| msr1 | 0.124 |
| nhlrc1 | 0.124 |
| tspo | 0.124 |
| cd200 | 0.124 |
| eif2ak4 | 0.124 |
| hsp90ab1 | 0.124 |
| hyi | 0.124 |
| nnmt | 0.124 |
| rgs5 | 0.124 |
| lpcat3 | 0.123 |
| taldo1 | 0.123 |
| coq3 | 0.123 |
| btg1 | 0.123 |
| mtap1b | 0.123 |
| hisppd2a | 0.123 |
| gnai1 | 0.123 |
| cnga2 | 0.123 |
| ezg | 0.123 |
| ppp3ca | 0.122 |
| ifitm2 | 0.122 |
| gstt2 | 0.122 |
| tyk2 | 0.122 |
| mcfd2 | 0.122 |
| mtrr | 0.122 |
| gphn | 0.122 |
| bnip3 | 0.122 |
| as3mt | 0.122 |
| mmab | 0.122 |
| ifngr2 | 0.122 |
| kcnk6 | 0.122 |
| guca2a | 0.122 |
| coq7 | 0.122 |
| eif2ak1 | 0.122 |
| gcat | 0.121 |
| kcnj8 | 0.121 |
| ptgfr | 0.121 |
| il24 | 0.121 |
| sftpd | 0.121 |
| irgm | 0.121 |
| calcb | 0.121 |
| angpt1 | 0.121 |
| papss2 | 0.121 |
| pdzk1 | 0.121 |
| alox12b | 0.121 |
| mat2a | 0.121 |
| mst1 | 0.121 |
| pygb | 0.121 |
| ppat | 0.121 |
| smtnl1 | 0.121 |
| urod | 0.121 |
| agl | 0.12 |
| nr3c2 | 0.12 |
| idh3g | 0.12 |
| casp4 | 0.12 |
| pdss2 | 0.12 |
| dolk | 0.12 |
| cyp2j6 | 0.12 |
| cox2 | 0.12 |
| acaca | 0.12 |
| adpgk | 0.12 |
| msra | 0.12 |
| dars | 0.12 |
| b3galt5 | 0.12 |
| mtr | 0.12 |
| hmgcl | 0.12 |
| gap43 | 0.12 |
| pcmt1 | 0.12 |
| pygm | 0.119 |
| klf4 | 0.119 |
| scly | 0.119 |
| bc032265 | 0.119 |
| hsd11b1 | 0.119 |
| uqcrfs1 | 0.119 |
| atp2a1 | 0.119 |
| hhip | 0.119 |
| agxt2 | 0.119 |
| slc25a11 | 0.119 |
| sirpa | 0.119 |
| ctsd | 0.119 |
| cox10 | 0.119 |
| acsm2 | 0.119 |
| fpgs | 0.119 |
| vegfb | 0.119 |
| tacr2 | 0.119 |
| rp23-395h4.4 | 0.119 |
| hrh2 | 0.119 |
| pnoc | 0.118 |
| ggta1 | 0.118 |
| rrm2b | 0.118 |
| slc7a11 | 0.118 |
| sgk1 | 0.118 |
| zfp668 | 0.118 |
| serpinb2 | 0.118 |
| scye1 | 0.118 |
| st8sia6 | 0.118 |
| camk2a | 0.118 |
| 4930570c03rik | 0.118 |
| magee1 | 0.118 |
| fosl1 | 0.118 |
| tbcb | 0.118 |
| gna12 | 0.118 |
| hdc | 0.118 |
| idh3b | 0.118 |
| chsy3 | 0.117 |
| chat | 0.117 |
| flad1 | 0.117 |
| isca1 | 0.117 |
| nagk | 0.117 |
| calb2 | 0.117 |
| cnr2 | 0.117 |
| ptgr1 | 0.117 |
| 677156 | 0.117 |
| ptk2b | 0.117 |
| myh14 | 0.117 |
| ehbp1l1 | 0.117 |
| ndufs1 | 0.117 |
| cbr4 | 0.117 |
| usf1 | 0.117 |
| loc100038882 | 0.117 |
| hisppd1 | 0.117 |
| cyp4a10 | 0.117 |
| bpnt1 | 0.117 |
| serpinb7 | 0.117 |
| b3gnt2 | 0.117 |
| sepsecs | 0.116 |
| sirt1 | 0.116 |
| trim32 | 0.116 |
| nt5e | 0.116 |
| slc25a21 | 0.116 |
| ntan1 | 0.116 |
| myoz1 | 0.116 |
| rtn3 | 0.116 |
| serpinc1 | 0.116 |
| ifi9.5 | 0.116 |
| vdac3 | 0.116 |
| pfkm | 0.116 |
| glyctk | 0.116 |
| slc39a10 | 0.116 |
| sds | 0.116 |
| degs2 | 0.116 |
| s100a10 | 0.116 |
| atp6ap2 | 0.116 |
| cyp51 | 0.116 |
| abcb7 | 0.115 |
| fkbp1a | 0.115 |
| gja5 | 0.115 |
| galk2 | 0.115 |
| rpe | 0.115 |
| gucy2c | 0.115 |
| ddo | 0.115 |
| dnm1l | 0.115 |
| retnla | 0.115 |
| lonp1 | 0.115 |
| cav1 | 0.115 |
| pldn | 0.115 |
| idh3a | 0.115 |
| nudt6 | 0.115 |
| gucy2d | 0.115 |
| ppp1r3a | 0.115 |
| dph2 | 0.115 |
| uck2 | 0.114 |
| alox12e | 0.114 |
| nmnat3 | 0.114 |
| hsp34 | 0.114 |
| scn2a1 | 0.114 |
| c730007p19rik | 0.114 |
| omp | 0.114 |
| grk4 | 0.114 |
| amelx | 0.114 |
| pask | 0.114 |
| cript | 0.114 |
| 4931406c07rik | 0.114 |
| edn1 | 0.114 |
| pde1b | 0.114 |
| ppp2r1a | 0.114 |
| coq4 | 0.114 |
| abcc5 | 0.114 |
| sdhc | 0.114 |
| ado | 0.114 |
| marco | 0.114 |
| shpk | 0.114 |
| por | 0.114 |
| oxt | 0.114 |
| oxtr | 0.113 |
| cyp2a5 | 0.113 |
| pank3 | 0.113 |
| mthfr-ps1 | 0.113 |
| elovl5 | 0.113 |
| prhoxnb | 0.113 |
| slc17a8 | 0.113 |
| tac1 | 0.113 |
| cyp2c29 | 0.113 |
| stk11 | 0.113 |
| rb(16.17)32lub | 0.113 |
| rb(16.17)32lub | 0.113 |
| gla | 0.113 |
| ptpn6 | 0.113 |
| gstk1 | 0.113 |
| vc | 0.113 |
| trnt1 | 0.113 |
| mapk7 | 0.113 |
| casp12 | 0.113 |
| ramp3 | 0.113 |
| d6mit183 | 0.113 |
| ddr1 | 0.112 |
| tmlhe | 0.112 |
