# Supplementary material for: GIT2 Acts as a Potential Keystone Protein in Functional Hypothalamic Networks Associated with Age-Related Phenotypic Changes in Rats
Source: PLoS One. 2012 May 14;7(5):e36975. doi: 10.1371/journal.pone.0036975 (PMC3351446; doi:10.1371/journal.pone.0036975)
Supplement: Table S28 — GeneIndexer latent semantic indexing (LSI) of significantly-regulated ‘Synapse’ GO term group. Using the GO term group ‘Synapse’ as an input term, a list of the top 1000 implicitly-correlated (LSI correlation score >0.1) was generated using a full genome background list. (DOC) [file pone.0036975.s032.doc]

**Table S28. GeneIndexer latent semantic indexing (LSI) of significantly-regulated ‘Synapse’ GO term group.** Using the GO term group ‘Synapse’ as an input term, a list of the top 1000 implicitly-correlated (LSI correlation score >0.1) was generated using a full genome background list.

| ***Synapse*** |  |
| --- | --- |
|  |  |
| **Protein Symbol** | **LSI correlation score** |
| lrrc4b | 0.572 |
| loc100040794 | 0.5 |
| unc13c | 0.5 |
| nlgn2 | 0.461 |
| erc2 | 0.459 |
| nlgn1 | 0.457 |
| nrxn3 | 0.443 |
| nrxn2 | 0.441 |
| pcdhga3 | 0.437 |
| nd | 0.435 |
| musk | 0.421 |
| bsn | 0.412 |
| frmpd4 | 0.412 |
| nrxn1 | 0.411 |
| dok7 | 0.41 |
| glud2 | 0.399 |
| rapsn | 0.397 |
| ppfia4 | 0.395 |
| cplx3 | 0.383 |
| rims1 | 0.383 |
| pclo | 0.382 |
| rp23-100c5.8 | 0.382 |
| nlgn3 | 0.38 |
| cplx4 | 0.376 |
| begain | 0.376 |
| sv2a | 0.375 |
| agrn | 0.367 |
| syp | 0.367 |
| sv2b | 0.366 |
| prr7 | 0.364 |
| grid2ip | 0.362 |
| dlgap3 | 0.36 |
| grip2 | 0.357 |
| loc667655 | 0.349 |
| ppfia3 | 0.344 |
| arhgef9 | 0.338 |
| dlgap2 | 0.338 |
| rims4 | 0.337 |
| shank1 | 0.336 |
| shank3 | 0.335 |
| dlgap4 | 0.335 |
| unc13a | 0.333 |
| prss12 | 0.331 |
| syn1 | 0.331 |
| unc13b | 0.329 |
| slc32a1 | 0.327 |
| dlgap1 | 0.326 |
| sv2c | 0.326 |
| phldb2 | 0.324 |
| dlg3 | 0.32 |
| slc17a7 | 0.319 |
| tprgl | 0.319 |
| shank2 | 0.318 |
| syn2 | 0.317 |
| fchsd1 | 0.314 |
| chrnd | 0.314 |
| 5930434b04rik | 0.313 |
| chrna1 | 0.312 |
| gphn | 0.31 |
| nlgn4l | 0.309 |
| ppfia2 | 0.308 |
| chrne | 0.307 |
| sipa1l1 | 0.307 |
| syt2 | 0.307 |
| fchsd2 | 0.303 |
| rims2 | 0.302 |
| syt12 | 0.3 |
| lphn3 | 0.298 |
| syt4 | 0.297 |
| grid1 | 0.297 |
| rimbp2 | 0.297 |
| hpc | 0.297 |
| igsf9b | 0.295 |
| syn3 | 0.295 |
| tmem163 | 0.292 |
| slc17a8 | 0.292 |
| cript | 0.292 |
| dlg2 | 0.29 |
| klhl17 | 0.289 |
| cask | 0.288 |
| trappc4 | 0.286 |
| dagla | 0.285 |
| t(x;4)7rl | 0.284 |
| t(x;4)8rl | 0.284 |
| t(x;4)8rl | 0.284 |
| t(x;4)7rl | 0.284 |
| lphn1 | 0.284 |
| rims3 | 0.283 |
| dlg4 | 0.283 |
| icam5 | 0.282 |
| ophn1 | 0.28 |
| cacng8 | 0.28 |
| syngap1 | 0.279 |
| dbnl | 0.278 |
| dbn1 | 0.277 |
| lrp4 | 0.277 |
| rph3a | 0.276 |
| d8mit77 | 0.274 |
| rab3a | 0.274 |
| magi2 | 0.273 |
| baiap2 | 0.273 |
| cacng2 | 0.272 |
| ppfia1 | 0.271 |
| mpp4 | 0.27 |
| grik4 | 0.27 |
| dmxl2 | 0.268 |
| caps2 | 0.266 |
| lrfn4 | 0.265 |
| lrfn3 | 0.265 |
| daglb | 0.265 |
| mnx1 | 0.264 |
| dlg1 | 0.263 |
| syt1 | 0.261 |
| lrrc7 | 0.261 |
| lin7c | 0.261 |
| d2mit152 | 0.261 |
| cbln3 | 0.26 |
| nptx1 | 0.258 |
| lin7b | 0.257 |
| cnih3 | 0.257 |
| igsf9 | 0.257 |
| colq | 0.256 |
| nova2 | 0.256 |
| slc17a6 | 0.256 |
| chrnb1 | 0.254 |
| mpp3 | 0.253 |
| rab3c | 0.252 |
| cpne6 | 0.251 |
| lrfn1 | 0.25 |
| chrng | 0.25 |
| synpr | 0.25 |
| lphn2 | 0.249 |
| rp23-157o10.7 | 0.248 |
| cabp4 | 0.246 |
| sema4g | 0.244 |
| doc2a | 0.244 |
| erc1 | 0.243 |
| snph | 0.242 |
| slc30a3 | 0.242 |
| rab3il1 | 0.241 |
| sorbs2 | 0.241 |
| nptx2 | 0.241 |
| cplx1 | 0.24 |
| syngr3 | 0.239 |
| d230025d16rik | 0.239 |
| dennd1a | 0.238 |
| slc18a3 | 0.238 |
| grid2 | 0.237 |
| iqsec2 | 0.237 |
| lrfn2 | 0.235 |
| caskin2 | 0.235 |
| nsg1 | 0.235 |
| apba1 | 0.234 |
| grik1 | 0.233 |
| dnm3 | 0.233 |
| exoc4 | 0.233 |
| cplx2 | 0.232 |
| mpp2 | 0.232 |
| cdc42bpg | 0.231 |
| caskin1 | 0.23 |
| usp14 | 0.23 |
| syt10 | 0.229 |
| cd247 | 0.229 |
| sntb2 | 0.229 |
| nckipsd | 0.229 |
| pogo | 0.229 |
| d3bwg0562e | 0.229 |
| sdcbp | 0.228 |
| fyb | 0.227 |
| grik5 | 0.227 |
| skts5 | 0.227 |
| bv | 0.227 |
| cit | 0.226 |
| grit | 0.225 |
| lin7a | 0.224 |
| sytl2 | 0.222 |
| shc4 | 0.222 |
| cadps2 | 0.222 |
| cnksr2 | 0.221 |
| syt5 | 0.221 |
| clrn2 | 0.221 |
| pdzrn3 | 0.221 |
| rab3gap2 | 0.221 |
| zap70 | 0.22 |
| sytl3 | 0.219 |
| exoc3 | 0.218 |
| pick1 | 0.218 |
| grip1 | 0.218 |
| wasl | 0.218 |
| pcdh20 | 0.218 |
| efnb3 | 0.217 |
| pld4 | 0.217 |
| arc | 0.217 |
| synj1 | 0.217 |
| zdhhc3 | 0.217 |
| dtna | 0.216 |
| gda | 0.216 |
| tbkbp1 | 0.215 |
| homer3 | 0.215 |
| rab3gap1 | 0.215 |
| epb4.1l1 | 0.215 |
| ddn | 0.214 |
| git2 | 0.214 |
| wipf1 | 0.213 |
| dynll2 | 0.213 |
| ppp1r9a | 0.213 |
| cd2 | 0.213 |
| wdr7 | 0.212 |
| ston2 | 0.211 |
| slc1a7 | 0.211 |
| kalrn | 0.211 |
| au041133 | 0.211 |
| klrb1f | 0.209 |
| loc641201 | 0.209 |
| pfn2 | 0.209 |
| slc6a12 | 0.208 |
| syt9 | 0.208 |
| iqub | 0.208 |
| syt3 | 0.207 |
| homer2 | 0.207 |
| skap1 | 0.207 |
| ctnnd2 | 0.206 |
| bc1 | 0.206 |
| rab3b | 0.205 |
| cd3e | 0.205 |
| tmc1 | 0.204 |
| 3-Sep | 0.204 |
| slc5a7 | 0.204 |
| ppp1r9b | 0.204 |
| akap5 | 0.203 |
| neto1 | 0.203 |
| prkcq | 0.203 |
| aa407270 | 0.202 |
| kirrel3 | 0.202 |
| homer1 | 0.202 |
| nova1 | 0.201 |
| adcy1 | 0.201 |
| loc677282 | 0.2 |
| grm7 | 0.2 |
| tmem16b | 0.2 |
| rph3al | 0.199 |
| slc6a5 | 0.199 |
| was | 0.199 |
| git1 | 0.199 |
| utrn | 0.198 |
| agtpbp1 | 0.198 |
| gria3 | 0.197 |
| scrib | 0.197 |
| grik3 | 0.197 |
| amph | 0.196 |
| unc119 | 0.196 |
| arhgap17 | 0.196 |
| cacna1b | 0.196 |
| 5730410e15rik | 0.196 |
| grasp | 0.196 |
| dag1 | 0.195 |
| arhgef7 | 0.195 |
| cadm2 | 0.194 |
| cm | 0.194 |
| epha6 | 0.193 |
| vstm2l | 0.193 |
| synpo | 0.192 |
| syt16 | 0.192 |
| gria1 | 0.192 |
| epha4 | 0.192 |
| mpp5 | 0.192 |
| sema4c | 0.191 |
| nxph1 | 0.19 |
| gria4 | 0.19 |
| chl1 | 0.19 |
| whrn | 0.19 |
| doc2g | 0.19 |
| mtap1a | 0.189 |
| slc1a6 | 0.189 |
| syt11 | 0.189 |
| pcdhga1 | 0.189 |
| sdk2 | 0.189 |
| brsk1 | 0.189 |
| cbln1 | 0.189 |
| hld | 0.189 |
| pfn1 | 0.188 |
| negr1 | 0.187 |
| tbc1d10a | 0.187 |
| slc6a17 | 0.186 |
| vav1 | 0.186 |
| cdk5r2 | 0.186 |
| ston1 | 0.185 |
| cnn3 | 0.185 |
| ephb3 | 0.185 |
| bzrap1 | 0.185 |
| grik2 | 0.184 |
| snap91 | 0.184 |
| mpp6 | 0.183 |
| nr | 0.183 |
| tln2 | 0.183 |
| itk | 0.183 |
| syngr1 | 0.183 |
| vsnl1 | 0.183 |
| clstn1 | 0.183 |
| 11-Sep | 0.182 |
| syt7 | 0.182 |
| sh3gl2 | 0.182 |
| clstn2 | 0.182 |
| pak3 | 0.182 |
| d12mit143 | 0.182 |
| sps | 0.181 |
| znrf2 | 0.181 |
| ephb2 | 0.181 |
| dnm1 | 0.181 |
| kif1b | 0.181 |
| wasf2 | 0.18 |
| cacna1f | 0.18 |
| otof | 0.18 |
| abl2 | 0.18 |
| bc | 0.18 |
| trim9 | 0.18 |
| tcrb-v3 | 0.18 |
| lsg1 | 0.18 |
| cacnb4 | 0.179 |
| gria2 | 0.179 |
| espn | 0.178 |
| txk | 0.178 |
| card11 | 0.178 |
| zdhhc23 | 0.178 |
| grin2c | 0.178 |
| chat | 0.177 |
| sytl1 | 0.177 |
| slc6a1 | 0.177 |
| bc060632 | 0.177 |
| chn1 | 0.177 |
| camk2n2 | 0.176 |
| gabrr3 | 0.176 |
| loc100042150 | 0.176 |
| exnm | 0.176 |
| lcp2 | 0.176 |
| spn | 0.176 |
| itsn2 | 0.176 |
| jakmip1 | 0.176 |
| sytl4 | 0.176 |
| cd3g | 0.176 |
| trat1 | 0.176 |
| glrb | 0.175 |
| synj2 | 0.175 |
| atcay | 0.175 |
| cbln4 | 0.175 |
| olfr17 | 0.175 |
| cacna1a | 0.175 |
| mpp1 | 0.174 |
| lrrc4c | 0.174 |
| neto2 | 0.174 |
| cd24a | 0.174 |
| syt14 | 0.174 |
| pcdha2 | 0.174 |
| prima1 | 0.174 |
| bc018242 | 0.173 |
| wasf1 | 0.173 |
| glra4 | 0.173 |
| nyx | 0.173 |
| sdk1 | 0.173 |
| bc046331 | 0.173 |
| lck | 0.173 |
| slc12a5 | 0.172 |
| t(x;7)2rl | 0.172 |
| t(x;7)2rl | 0.172 |
| t(x;7)5rl | 0.172 |
| t(x;7)5rl | 0.172 |
| t(x;7)6rl | 0.172 |
| t(x;4)1rl | 0.172 |
| t(x;4)1rl | 0.172 |
| t(x;7)6rl | 0.172 |
| pip5k1c | 0.172 |
| efna3 | 0.172 |
| cd28 | 0.172 |
| plcb4 | 0.171 |
| chrna9 | 0.171 |
| cacna1d | 0.171 |
| nck2 | 0.171 |
| syt6 | 0.171 |
| atp2b2 | 0.17 |
| chrna10 | 0.17 |
| freq | 0.17 |
| cd3d | 0.17 |
| gripap1 | 0.17 |
| dpy19l4 | 0.169 |
| epha7 | 0.169 |
| cir | 0.169 |
| klhl24 | 0.169 |
| cyfip1 | 0.169 |
| mtap6 | 0.169 |
| crtam | 0.169 |
| trim2 | 0.168 |
| slc6a9 | 0.168 |
| grm1 | 0.168 |
| mycbp2 | 0.168 |
| nrg1 | 0.168 |
| cnksr3 | 0.168 |
| clrn3 | 0.167 |
| cadps | 0.167 |
| cd6 | 0.167 |
| erbb4 | 0.167 |
| nrgn | 0.167 |
| kif13b | 0.167 |
| ephb1 | 0.167 |
| clec2i | 0.167 |
| abi1 | 0.167 |
| cyfip2 | 0.167 |
| dnajc5 | 0.166 |
| enah | 0.166 |
| nrn1 | 0.166 |
| snta1 | 0.166 |
| apba2 | 0.166 |
| cd244 | 0.166 |
| wipf3 | 0.165 |
| stxbp1 | 0.165 |
| gap43 | 0.165 |
| sema5b | 0.165 |
| cpeb1 | 0.165 |
| clstn3 | 0.165 |
| cadm1 | 0.164 |
| erbb2ip | 0.164 |
| ptpn4 | 0.164 |
| actr3 | 0.164 |
| myo5a | 0.163 |
| arpc2 | 0.163 |
| cacna1e | 0.163 |
| fnbp1l | 0.163 |
| basp1 | 0.162 |
| nxph3 | 0.162 |
| lime1 | 0.162 |
| syt8 | 0.162 |
| palm | 0.162 |
| ablim1 | 0.161 |
| cbln2 | 0.161 |
| slamf6 | 0.161 |
| grm6 | 0.161 |
| adcy8 | 0.161 |
| camk2b | 0.161 |
| isl2 | 0.161 |
| cadm3 | 0.161 |
| baiap3 | 0.16 |
| pak2 | 0.16 |
| abi2 | 0.16 |
| rab40c | 0.16 |
| slc1a2 | 0.16 |
| cntnap3 | 0.16 |
| d9ertd280e | 0.159 |
| arpc3 | 0.159 |
| brsk2 | 0.159 |
| cspg5 | 0.159 |
| t(12;16)1cje | 0.159 |
| lat | 0.158 |
| pma | 0.158 |
| neurod6 | 0.158 |
| cadm4 | 0.158 |
| etn1 | 0.158 |
| myo15 | 0.157 |
| nptxr | 0.157 |
| dnm2 | 0.157 |
| prkar1b | 0.157 |
| daam1 | 0.156 |
| 2300002m23rik | 0.156 |
| ntrk2 | 0.156 |
| hrs2 | 0.156 |
| arhgef6 | 0.156 |
| emx1 | 0.156 |
| efna5 | 0.156 |
| dgkb | 0.156 |
| efnb1 | 0.155 |
| cacna2d2 | 0.155 |
| mast1 | 0.155 |
| kif17 | 0.155 |
| sema4b | 0.154 |
| grin2a | 0.154 |
| nefh | 0.153 |
| lax1 | 0.153 |
| nipsnap1 | 0.153 |
| sit1 | 0.153 |
| bhlhb4 | 0.153 |
| d6mit33 | 0.153 |
| gabbr2 | 0.153 |
| unc13d | 0.153 |
| grm4 | 0.152 |
| lamb2 | 0.152 |
| def6 | 0.152 |
| srgap3 | 0.152 |
| gjd2 | 0.152 |
| pstpip1 | 0.152 |
| grm5 | 0.152 |
| cacna2d4 | 0.152 |
| itgal | 0.152 |
| cacng3 | 0.151 |
| pacsin1 | 0.151 |
| sema4f | 0.15 |
| d13mit249 | 0.15 |
| grm2 | 0.15 |
| scamp4 | 0.15 |
| grap2 | 0.15 |
| pcdhac2 | 0.15 |
| raver2 | 0.15 |
| myo6 | 0.149 |
| apba3 | 0.149 |
| d9mit339 | 0.149 |
| rab27a | 0.149 |
| syne1 | 0.148 |
| caly | 0.148 |
| svop | 0.148 |
| stau2 | 0.148 |
| slc6a7 | 0.148 |
| e430004n04rik | 0.148 |
| cdk5r1 | 0.147 |
| sh3glb1 | 0.147 |
| fgf22 | 0.147 |
| kif1a | 0.147 |
| grinl1a | 0.147 |
| astn1 | 0.147 |
| sez6 | 0.147 |
| grin2b | 0.147 |
| gabrr1 | 0.146 |
| grin3a | 0.146 |
| rufy3 | 0.146 |
| mynn | 0.146 |
| plekhb1 | 0.146 |
| kir3dl2 | 0.146 |
| baiap2l1 | 0.146 |
| arpc4 | 0.145 |
| 2810003c17rik | 0.145 |
| mtap1b | 0.145 |
| ezr | 0.144 |
| sbp | 0.144 |
| zdhhc21 | 0.144 |
| slc2a13 | 0.144 |
| ppp1r3d | 0.144 |
| d12mit146 | 0.144 |
| nma | 0.144 |
| evl | 0.144 |
| slc1a3 | 0.144 |
| mpp7 | 0.144 |
| ctnna2 | 0.144 |
| dach2 | 0.144 |
| epb4.1l2 | 0.144 |
| xmv6 | 0.143 |
| actr2 | 0.143 |
| epha5 | 0.143 |
| amigo1 | 0.143 |
| amigo3 | 0.143 |
| sntb1 | 0.143 |
| znrf1 | 0.143 |
| msn | 0.143 |
| cd47 | 0.143 |
| ntf3 | 0.142 |
| doc2b | 0.142 |
| klra15 | 0.142 |
| ap3m2 | 0.142 |
| cap1 | 0.142 |
| mpaps1 | 0.142 |
| cd226 | 0.142 |
| snap25 | 0.141 |
| slc1a1 | 0.141 |
| nefm | 0.141 |
| au040829 | 0.141 |
| lrfn5 | 0.14 |
| cdh8 | 0.14 |
| efna2 | 0.14 |
| omp | 0.14 |
| gad2 | 0.14 |
| cdk5 | 0.14 |
| ntf5 | 0.14 |
| cdc42se1 | 0.14 |
| cacng5 | 0.14 |
| exoc7 | 0.14 |
| d18mit164 | 0.139 |
| pcdhgc3 | 0.139 |
| mllt4 | 0.139 |
| ap4m1 | 0.139 |
| kcnn2 | 0.139 |
| map6d1 | 0.139 |
| cacng7 | 0.139 |
| slc17a1 | 0.139 |
| caln1 | 0.139 |
| slamf9 | 0.139 |
| sla | 0.138 |
| npas4 | 0.138 |
| iqsec3 | 0.138 |
| lrrtm1 | 0.138 |
| clrn1 | 0.138 |
| cblb | 0.138 |
| prickle3 | 0.138 |
| sirpa | 0.138 |
| grin2d | 0.138 |
| wld | 0.137 |
| wasf3 | 0.137 |
| gabbr1 | 0.137 |
| sh2d1a | 0.137 |
| ncam2 | 0.137 |
| cd48 | 0.137 |
| pi | 0.137 |
| ctl1 | 0.137 |
| 9130404h23rik | 0.137 |
| sypl | 0.137 |
| ntng2 | 0.136 |
| cd160 | 0.136 |
| pvalb | 0.136 |
| camk2a | 0.136 |
| gdi1 | 0.136 |
| gabarap | 0.136 |
| dvl1 | 0.135 |
| grin1 | 0.135 |
| kcna4 | 0.135 |
| d14mit95 | 0.135 |
| rora | 0.135 |
| pofut2 | 0.134 |
| grm3 | 0.134 |
| ptprcap | 0.134 |
| cd8b1 | 0.134 |
| ache | 0.134 |
| dner | 0.134 |
| sla2 | 0.134 |
| pcdh8 | 0.134 |
| nxph2 | 0.134 |
| hcst | 0.134 |
| 1700019h03rik | 0.134 |
| thy1 | 0.133 |
| tnr | 0.133 |
| phactr1 | 0.133 |
| snx9 | 0.133 |
| klrb1b | 0.133 |
| cd5 | 0.133 |
| itga7 | 0.133 |
| slc6a11 | 0.133 |
| cntnap2 | 0.133 |
| epha8 | 0.133 |
| pcdha1 | 0.133 |
| syngr2 | 0.133 |
| ush1c | 0.133 |
| itsn1 | 0.132 |
| nbea | 0.132 |
| dgkz | 0.132 |
| mn | 0.132 |
| nxph4 | 0.132 |
| h35 | 0.132 |
| gabrg1 | 0.131 |
| jph4 | 0.131 |
| dscaml1 | 0.131 |
| cdh15 | 0.131 |
| t(12;16)1cje | 0.131 |
| spg21 | 0.131 |
| wipf2 | 0.131 |
| dtnb | 0.131 |
| baf | 0.13 |
| d18mit65 | 0.13 |
| mea | 0.13 |
| amigo2 | 0.13 |
| bc016423 | 0.13 |
| abi3 | 0.13 |
| exph5 | 0.13 |
| nck1 | 0.13 |
| d18mit20 | 0.129 |
| hpcal4 | 0.129 |
| myrip | 0.129 |
| tex261 | 0.129 |
| grm8 | 0.129 |
| epha3 | 0.129 |
| sh3gl3 | 0.129 |
| nckap1 | 0.129 |
| srr | 0.129 |
| sdcbp2 | 0.129 |
| mpdz | 0.129 |
| ptprc | 0.129 |
| tk | 0.129 |
| memo1 | 0.129 |
| rnf39 | 0.128 |
| pja2 | 0.128 |
| epha10 | 0.128 |
| xmv24 | 0.128 |
| scrn2 | 0.128 |
| tulp1 | 0.128 |
| yb2 | 0.128 |
| kirrel | 0.128 |
| slmap | 0.128 |
| clec4b1 | 0.128 |
| cdc42 | 0.128 |
| mlph | 0.128 |
| vmn2r26 | 0.128 |
| lat2 | 0.127 |
| rab3d | 0.127 |
| myo3b | 0.127 |
| gabra1 | 0.127 |
| rdx | 0.127 |
| spata13 | 0.127 |
| fnbp1 | 0.127 |
| hnt | 0.127 |
| calb2 | 0.127 |
| magi1 | 0.126 |
| bin2 | 0.126 |
| rab35 | 0.126 |
| snapin | 0.126 |
| klk8 | 0.126 |
| olfr2 | 0.126 |
| dbndd1 | 0.126 |
| gr | 0.126 |
| 4930506m07rik | 0.126 |
| adam19 | 0.126 |
| aw209491 | 0.126 |
| mrpl32 | 0.126 |
| tw | 0.126 |
| 2900073g15rik | 0.125 |
| pdzd7 | 0.125 |
| pcdha4 | 0.125 |
| cabp1 | 0.125 |
| gopc | 0.125 |
| myo7a | 0.125 |
| icam2 | 0.125 |
| chn2 | 0.125 |
| gpr98 | 0.124 |
| olfr160 | 0.124 |
| c330002i19rik | 0.124 |
| dock2 | 0.124 |
| d17mit232.1 | 0.124 |
| rhof | 0.124 |
| gabra6 | 0.124 |
| dscam | 0.124 |
| gipc1 | 0.124 |
| pak1 | 0.124 |
| ap2m1 | 0.124 |
| st8sia2 | 0.124 |
| strn4 | 0.123 |
| marcks | 0.123 |
| syt15 | 0.123 |
| kif5b | 0.123 |
| gpm6a | 0.123 |
| fmr1 | 0.123 |
| stau1 | 0.122 |
| itpka | 0.122 |
| rftn1 | 0.122 |
| mtss1 | 0.122 |
| stu | 0.122 |
| 7-Sep | 0.121 |
| syt13 | 0.121 |
| gabpa | 0.121 |
| d2mit434 | 0.121 |
| nrn1l | 0.121 |
| coro1c | 0.121 |
| layn | 0.121 |
| efnb2 | 0.121 |
| klrc1 | 0.121 |
| sh2d1b2 | 0.121 |
| st8sia4 | 0.121 |
| dnali1 | 0.121 |
| d2mit436 | 0.121 |
| sema6c | 0.12 |
| myo3a | 0.12 |
| nckap1l | 0.12 |
| camkk2 | 0.12 |
| av249152 | 0.12 |
| d9mit73 | 0.12 |
| ncdn | 0.12 |
| glra3 | 0.12 |
| pvrl1 | 0.12 |
| kcnc3 | 0.12 |
| d2mit242 | 0.12 |
| klra1 | 0.12 |
| magee1 | 0.12 |
| bc005764 | 0.119 |
| rab27b | 0.119 |
| ephb6 | 0.119 |
| grina | 0.119 |
| dclk2 | 0.119 |
| cttn | 0.119 |
| pik3ap1 | 0.119 |
| olfr151 | 0.119 |
| kcnj6 | 0.119 |
| mneu | 0.119 |
| diap1 | 0.119 |
| slamf8 | 0.119 |
| pcp2 | 0.119 |
| ap3d1 | 0.119 |
| rhot1 | 0.118 |
| diap3 | 0.118 |
| gpr156 | 0.118 |
| gabrg2 | 0.118 |
| camk1g | 0.118 |
| slc18a2 | 0.118 |
| cd2ap | 0.118 |
| egr3 | 0.118 |
| rcn2 | 0.118 |
| plk2 | 0.118 |
| tyrobp | 0.118 |
| gabrd | 0.118 |
| slc6a13 | 0.118 |
| gad1 | 0.118 |
| vav2 | 0.117 |
| frmd4b | 0.117 |
| sh2d1b1 | 0.117 |
| inadl | 0.117 |
| tiam2 | 0.117 |
| klrk1 | 0.117 |
| lgals1 | 0.117 |
| ica1 | 0.117 |
| skap2 | 0.117 |
| khdrbs1 | 0.117 |
| gabrr2 | 0.117 |
| gabrb1 | 0.117 |
| mrpl20 | 0.117 |
| necab3 | 0.117 |
| ncr1 | 0.117 |
| hsh2d | 0.117 |
| mmp24 | 0.116 |
| ascl4 | 0.116 |
| macf1 | 0.116 |
| l1cam | 0.116 |
| d16h22s680e | 0.116 |
| olfr1507 | 0.116 |
| rasgrf1 | 0.116 |
| nfasc | 0.116 |
| strn | 0.116 |
| cacnb3 | 0.116 |
| nell2 | 0.116 |
| adam22 | 0.116 |
| plcl2 | 0.115 |
| cd80 | 0.115 |
| a630098a13rik | 0.115 |
| nsf | 0.115 |
| ctla4 | 0.115 |
| nptn | 0.115 |
| lrrc4 | 0.115 |
| cd209a | 0.115 |
| d3mit192 | 0.115 |
| efhb | 0.114 |
| 1700009n14rik | 0.114 |
| vav3 | 0.114 |
| cntn1 | 0.114 |
| siglec15 | 0.114 |
| cnih2 | 0.114 |
| eml1 | 0.114 |
| pacsin2 | 0.114 |
| cdc42se2 | 0.114 |
| fer1l4 | 0.114 |
| phactr2 | 0.113 |
| ptpn5 | 0.113 |
| cacng4 | 0.113 |
| arpc1b | 0.113 |
| dep | 0.113 |
| ap2a2 | 0.113 |
| cacnb2 | 0.113 |
| large | 0.113 |
| pde6b | 0.113 |
| mctp1 | 0.113 |
| camkv | 0.112 |
| chst10 | 0.112 |
| gabra3 | 0.112 |
| d1mit365 | 0.112 |
| ms4a4b | 0.112 |
| pcdha@ | 0.112 |
| slamf1 | 0.112 |
| ncam1 | 0.112 |
| epb4.1l5 | 0.112 |
| rua | 0.112 |
| sycn | 0.111 |
| pcdh15 | 0.111 |
| stk10 | 0.111 |
| sytl5 | 0.111 |
| mylip | 0.111 |
| sh3d2c1 | 0.111 |
| tmem37 | 0.111 |
| erbb3 | 0.111 |
| sh3kbp1 | 0.111 |
| dnaja3 | 0.111 |
| scamp1 | 0.111 |
| cdh2 | 0.111 |
| sirpb1 | 0.111 |
| cntnap1 | 0.111 |
| emb | 0.111 |
| gabrb2 | 0.11 |
| cd84 | 0.11 |
| klrc3 | 0.11 |
| d9mit304 | 0.11 |
| rabac1 | 0.11 |
| kcnd2 | 0.11 |
| klra16 | 0.11 |
| clec9a | 0.11 |
| olfr256 | 0.11 |
| necab1 | 0.11 |
| pscdbp | 0.109 |
| stx1a | 0.109 |
| 6330527o06rik | 0.109 |
| tiam1 | 0.109 |
| shroom4 | 0.109 |
| cd38 | 0.109 |
| gzmc | 0.109 |
| bai1 | 0.109 |
| klrg1 | 0.109 |
| spock1 | 0.109 |
| lzts1 | 0.109 |
| numbl | 0.109 |
| spnb4 | 0.109 |
| vamp1 | 0.108 |
| tg(bcl2l1)1cbt | 0.108 |
| el1 | 0.108 |
| klrd1 | 0.108 |
| bcas1 | 0.108 |
| mecp2 | 0.108 |
| snap29 | 0.108 |
| rab12 | 0.108 |
| synj2bp | 0.108 |
| mnm | 0.108 |
| exoc5 | 0.108 |
| cdh10 | 0.108 |
| camk2n1 | 0.108 |
| epn1 | 0.108 |
| pvrl3 | 0.108 |
| 2610204m08rik | 0.108 |
| cdkl2 | 0.107 |
| rps15a | 0.107 |
| lair1 | 0.107 |
| tln1 | 0.107 |
| tcrbe | 0.107 |
| d18mit24 | 0.107 |
| faim2 | 0.107 |
| gabrb3 | 0.107 |
| chrm1 | 0.107 |
| d10mit251 | 0.107 |
| b4galnt1 | 0.107 |
| necab2 | 0.107 |
| gabra4 | 0.107 |
| d14mit265 | 0.107 |
| clnk | 0.106 |
| cdc2b | 0.106 |
| gabra5 | 0.106 |
| grin3b | 0.106 |
| kif3c | 0.106 |
| wrmod1 | 0.106 |
| wdfy3 | 0.106 |
| d18mit66 | 0.106 |
| gtrgeo22 | 0.106 |
| prot | 0.106 |
| vcl | 0.106 |
| hist1h4a | 0.106 |
| d6mit300 | 0.106 |
| sema4a | 0.106 |
| ppt1 | 0.105 |
| sh3bp2 | 0.105 |
| slamf7 | 0.105 |
| vezt | 0.105 |
| syx1 | 0.105 |
| syx2 | 0.105 |
| ottmusg00000014994 | 0.105 |
| sema6a | 0.105 |
| lrrn4 | 0.105 |
| sh3bp1 | 0.105 |
| cacna1c | 0.105 |
| ush2a | 0.105 |
| scel | 0.105 |
| pgia6 | 0.105 |
| arhgap26 | 0.105 |
| cd86 | 0.105 |
| vmn2r1 | 0.105 |
| ly9 | 0.105 |
| plek2 | 0.105 |
| cdh23 | 0.105 |
| cd24c | 0.105 |
| cd24b | 0.105 |
| sh3pxd2a | 0.105 |
| 100043861 | 0.105 |
| eps8 | 0.105 |
| rimbp3 | 0.104 |
| pag1 | 0.104 |
| rac3 | 0.104 |
| icos | 0.104 |
| crb3 | 0.104 |
| camk4 | 0.104 |
| sync | 0.104 |
| hip1r | 0.104 |
| gdnfl | 0.104 |
| syce2 | 0.104 |
| pard6b | 0.104 |
| slc12a9 | 0.104 |
| ushbp1 | 0.104 |
| fkbp15 | 0.104 |
| emv35 | 0.104 |
| hug | 0.104 |
| rapgef4 | 0.104 |
| scamp5 | 0.104 |
| lama2 | 0.104 |
| triobp | 0.104 |
| d12ertd551e | 0.104 |
| cdc42bpa | 0.103 |
| ubxd5 | 0.103 |
| iqgap3 | 0.103 |
| actg1 | 0.103 |
| card14 | 0.103 |
| plekha2 | 0.103 |
| plcb1 | 0.103 |
| rnd1 | 0.103 |
| d9mit351 | 0.103 |
| exoc2 | 0.103 |
| tnk2 | 0.103 |
| ptprf | 0.103 |
| rhot2 | 0.103 |
| slc25a18 | 0.103 |
| afg3l2 | 0.103 |
