# Supplementary material for: GIT2 Acts as a Potential Keystone Protein in Functional Hypothalamic Networks Associated with Age-Related Phenotypic Changes in Rats
Source: PLoS One. 2012 May 14;7(5):e36975. doi: 10.1371/journal.pone.0036975 (PMC3351446; doi:10.1371/journal.pone.0036975)
Supplement: Table S29 — GeneIndexer latent semantic indexing (LSI) of significantly-regulated ‘Response to stress’ GO term group. Using the GO term group ‘Response to stress’ as an input term, a list of the top 1000 implicitly-correlated (LSI correlation score >0.1) was generated using a full genome background list. (DOC) [file pone.0036975.s033.doc]

**Table S29. GeneIndexer latent semantic indexing (LSI) of significantly-regulated ‘Response to stress’ GO term group.** Using the GO term group ‘Response to stress’ as an input term, a list of the top 1000 implicitly-correlated (LSI correlation score>0.1) was generated using a full genome background list.

| ***Response to stress*** |  |
| --- | --- |
|  |  |
| **Protein Symbol** | **LSI correlation score** |
| tmem161a | 0.636 |
| ai462493 | 0.589 |
| tmed4 | 0.565 |
| ern2 | 0.531 |
| ern1 | 0.517 |
| 2810022l02rik | 0.502 |
| sesn2 | 0.499 |
| eif2ak3 | 0.489 |
| 4632434i11rik | 0.488 |
| eif2a | 0.483 |
| myd116 | 0.478 |
| atf6 | 0.477 |
| dnajc3 | 0.469 |
| 1200003c05rik | 0.469 |
| tmem85 | 0.469 |
| ppp1r15b | 0.465 |
| herpud1 | 0.461 |
| hyou1 | 0.461 |
| 2010100o12rik | 0.455 |
| dnajb9 | 0.45 |
| ero1l | 0.449 |
| znhit1 | 0.445 |
| zfand2a | 0.44 |
| atf4 | 0.438 |
| eif2ak4 | 0.433 |
| scara3 | 0.431 |
| 3230401d17rik | 0.43 |
| creb3l1 | 0.429 |
| d3ucla1 | 0.428 |
| ccdc100 | 0.425 |
| ddit4l | 0.422 |
| ddit4 | 0.422 |
| chac1 | 0.417 |
| wdr26 | 0.417 |
| atf3 | 0.416 |
| xbp1 | 0.415 |
| casp12 | 0.414 |
| ddit3 | 0.414 |
| map3k6 | 0.413 |
| creb3l3 | 0.413 |
| dnajc10 | 0.411 |
| asns | 0.407 |
| a930001n09rik | 0.406 |
| trap1 | 0.402 |
| prkra | 0.399 |
| b230120h23rik | 0.398 |
| higd1a | 0.397 |
| stk35 | 0.396 |
| shisa5 | 0.393 |
| txn2 | 0.393 |
| sesn1 | 0.392 |
| taok3 | 0.391 |
| syvn1 | 0.391 |
| gcn1l1 | 0.391 |
| spcs1 | 0.39 |
| gadd45g | 0.39 |
| tmem132a | 0.387 |
| eif2s1 | 0.387 |
| hsph1 | 0.386 |
| cirbp | 0.384 |
| prodh2 | 0.383 |
| rps27l | 0.383 |
| 3930401k13rik | 0.383 |
| tabw2 | 0.381 |
| mirn208a | 0.379 |
| tg(bcl2l1)2cbt | 0.377 |
| hsl1 | 0.377 |
| lrdd | 0.376 |
| trib3 | 0.376 |
| ero1lb | 0.376 |
| msra | 0.375 |
| gadd45b | 0.374 |
| mprip | 0.374 |
| tegt | 0.373 |
| prdx3 | 0.373 |
| sec61a1 | 0.373 |
| mapk13 | 0.372 |
| 5730403b10rik | 0.372 |
| nol3 | 0.371 |
| tsc22d2 | 0.371 |
| 4930519f09rik | 0.371 |
| abra | 0.37 |
| qsox2 | 0.37 |
| creb3l2 | 0.369 |
| prkrir | 0.368 |
| prdx4 | 0.368 |
| sec61a2 | 0.367 |
| atf5 | 0.367 |
| telo2 | 0.366 |
| selk | 0.365 |
| crebl1 | 0.365 |
| trp53inp1 | 0.362 |
| srxn1 | 0.362 |
| prdx1 | 0.362 |
| stk25 | 0.362 |
| oxr1 | 0.362 |
| eif2ak1 | 0.361 |
| fkbpl | 0.361 |
| txnip | 0.361 |
| cirbp-rs3 | 0.359 |
| cirbp-rs1 | 0.359 |
| zmat3 | 0.357 |
| mrpl13 | 0.357 |
| trp53i11 | 0.356 |
| mrps11 | 0.356 |
| phlda1 | 0.355 |
| tipin | 0.355 |
| ier3 | 0.354 |
| 9630033f20rik | 0.353 |
| mapkapk5 | 0.352 |
| 1200002n14rik | 0.351 |
| lonp1 | 0.351 |
| 2700094k13rik | 0.351 |
| h47 | 0.35 |
| prdx2 | 0.349 |
| hspa5 | 0.349 |
| vrk2 | 0.348 |
| clspn | 0.348 |
| msrb2 | 0.347 |
| gadd45a | 0.347 |
| prdx5 | 0.346 |
| atmin | 0.345 |
| rnd3 | 0.345 |
| rrm2b | 0.345 |
| stk24 | 0.345 |
| plk3 | 0.344 |
| 2610207i05rik | 0.343 |
| plekhf2 | 0.342 |
| ppm1d | 0.342 |
| mapk11 | 0.341 |
| map3k5 | 0.34 |
| ftmt | 0.339 |
| ppp5c | 0.339 |
| arl6ip5 | 0.339 |
| gclc | 0.338 |
| sh3rf1 | 0.336 |
| 1110008f13rik | 0.336 |
| ccar1 | 0.336 |
| rbm3 | 0.334 |
| slk | 0.334 |
| aifm2 | 0.333 |
| dnajb5 | 0.333 |
| triap1 | 0.332 |
| dhcr24 | 0.332 |
| eef2 | 0.332 |
| 6620401k05rik | 0.331 |
| ier2 | 0.331 |
| isg20l1 | 0.331 |
| rbm42 | 0.331 |
| glrx | 0.33 |
| plk2 | 0.33 |
| crebzf | 0.329 |
| gsta4 | 0.329 |
| prdx6 | 0.329 |
| dusp2 | 0.329 |
| pcbp4 | 0.328 |
| glrx2 | 0.327 |
| cep164 | 0.326 |
| nfe2l1 | 0.326 |
| apold1 | 0.326 |
| pdia2 | 0.326 |
| zdhhc16 | 0.325 |
| rabepk | 0.325 |
| zfp420 | 0.325 |
| pmaip1 | 0.325 |
| taok1 | 0.325 |
| nupr1 | 0.324 |
| rdm1 | 0.324 |
| endog | 0.324 |
| arrdc2 | 0.323 |
| creb3 | 0.323 |
| slc6a6 | 0.323 |
| hsf1 | 0.323 |
| hsp84-2 | 0.322 |
| siva1 | 0.322 |
| bnip3l | 0.322 |
| l1md-a5 | 0.322 |
| hsp90b1 | 0.322 |
| trp53i13 | 0.321 |
| mapkapk2 | 0.32 |
| mbtps2 | 0.32 |
| pask | 0.32 |
| ifi202b | 0.32 |
| txn1 | 0.32 |
| nsmaf | 0.32 |
| map3k9 | 0.32 |
| nuak2 | 0.32 |
| usp28 | 0.319 |
| edem1 | 0.319 |
| apip | 0.319 |
| loc100046187 | 0.319 |
| fosl2 | 0.319 |
| aatf | 0.317 |
| ier3ip1 | 0.317 |
| map3k13 | 0.317 |
| ubxd2 | 0.317 |
| nfat5 | 0.317 |
| prdx6-rs2 | 0.317 |
| ints3 | 0.316 |
| 1110054o05rik | 0.316 |
| psmd1 | 0.316 |
| txnl1 | 0.316 |
| dusp4 | 0.316 |
| ccdc47 | 0.316 |
| gpx7 | 0.316 |
| rcsd1 | 0.316 |
| zfp385a | 0.315 |
| plagl2 | 0.315 |
| rpl26 | 0.315 |
| rhbdd2 | 0.315 |
| slc7a11 | 0.315 |
| gpr37 | 0.314 |
| 1500019g21rik | 0.314 |
| susp | 0.313 |
| hsp34 | 0.313 |
| sphkap | 0.313 |
| msrb3 | 0.313 |
| mtf1 | 0.313 |
| mknk2 | 0.312 |
| magea4 | 0.312 |
| dnajb11 | 0.312 |
| derl1 | 0.312 |
| zbtb4 | 0.311 |
| eef2k | 0.311 |
| jtv1 | 0.311 |
| bnip1 | 0.311 |
| topbp1 | 0.311 |
| 2900092e17rik | 0.311 |
| mapk6 | 0.31 |
| aifm3 | 0.31 |
| 1500041n16rik | 0.31 |
| alkbh8 | 0.31 |
| tg(myhca-cre)1abel | 0.31 |
| bach1 | 0.31 |
| rad9 | 0.31 |
| frag1 | 0.309 |
| 2310056p07rik | 0.309 |
| txndc4 | 0.309 |
| pdia6 | 0.309 |
| mirn7b | 0.308 |
| perp | 0.308 |
| obfc2b | 0.308 |
| mbip | 0.308 |
| ei24 | 0.307 |
| bc010304 | 0.307 |
| rb1cc1 | 0.307 |
| gclm | 0.307 |
| hus1 | 0.307 |
| rps6ka4 | 0.306 |
| jmy | 0.306 |
| dclre1a | 0.306 |
| foxred2 | 0.306 |
| mylk2 | 0.306 |
| loc100034726 | 0.306 |
| rfwd2 | 0.306 |
| ucn2 | 0.306 |
| siah2 | 0.305 |
| centg3 | 0.305 |
| gmfb | 0.305 |
| ier5 | 0.305 |
| gstp2 | 0.304 |
| txndc17 | 0.304 |
| rnf5 | 0.304 |
| sltm | 0.304 |
| inf2 | 0.303 |
| txndc12 | 0.303 |
| tlk1 | 0.303 |
| bag3 | 0.302 |
| ppm1l | 0.302 |
| bnip3 | 0.302 |
| taok2 | 0.302 |
| nrk | 0.302 |
| gpx6 | 0.301 |
| ubxd5 | 0.301 |
| dusp1 | 0.301 |
| pdia4 | 0.301 |
| sec61b | 0.301 |
| smpd4 | 0.3 |
| mtbp | 0.3 |
| il24 | 0.3 |
| map2k6 | 0.3 |
| smpd2 | 0.3 |
| dusp14 | 0.299 |
| erp29 | 0.299 |
| edem2 | 0.299 |
| paxip1 | 0.299 |
| npas4 | 0.299 |
| nrbp1 | 0.299 |
| kdelr1 | 0.299 |
| txnrd1 | 0.298 |
| ing4 | 0.298 |
| them4 | 0.298 |
| gramd4 | 0.298 |
| 4932409i22rik | 0.298 |
| eg244911 | 0.298 |
| 3300001a09rik | 0.298 |
| glrx5 | 0.297 |
| ddx47 | 0.297 |
| avil | 0.297 |
| egln3 | 0.297 |
| gmeb1 | 0.297 |
| dusp10 | 0.297 |
| rnf8 | 0.296 |
| cxxc5 | 0.296 |
| map2k7 | 0.296 |
| bach2 | 0.296 |
| bbc3 | 0.295 |
| mapk15 | 0.295 |
| dclre1b | 0.295 |
| tfpt | 0.295 |
| obfc2a | 0.294 |
| rgnef | 0.294 |
| dusp12 | 0.294 |
| dnajb1 | 0.294 |
| hspb6 | 0.294 |
| fhod1 | 0.293 |
| af366264 | 0.293 |
| ppif | 0.293 |
| mkl1 | 0.293 |
| nudt15 | 0.292 |
| trsp | 0.292 |
| dapk3 | 0.292 |
| clic4 | 0.291 |
| glrx3 | 0.291 |
| derl2 | 0.291 |
| txndc1 | 0.291 |
| rad17 | 0.291 |
| uaca | 0.29 |
| lias | 0.29 |
| txndc5 | 0.29 |
| map2k5 | 0.29 |
| dusp5 | 0.29 |
| aco2 | 0.29 |
| tfam | 0.29 |
| chek1 | 0.29 |
| eef1e1 | 0.29 |
| narfl | 0.29 |
| psmd8 | 0.29 |
| d3mit241 | 0.289 |
| sec61g | 0.289 |
| rb(16.17)32lub | 0.289 |
| rb(16.17)32lub | 0.289 |
| eif2ak2 | 0.289 |
| stk4 | 0.289 |
| hipk1 | 0.289 |
| errfi1 | 0.289 |
| pdrg1 | 0.289 |
| scpro5 | 0.288 |
| creb3l4 | 0.288 |
| idh2 | 0.288 |
| map3k1 | 0.288 |
| phb2 | 0.288 |
| atf2 | 0.288 |
| smpd3 | 0.288 |
| dus2l | 0.288 |
| mapkapk3 | 0.288 |
| ccdc88a | 0.288 |
| tmem173 | 0.288 |
| mirn34b | 0.288 |
| mirn34c | 0.288 |
| sil1 | 0.288 |
| map2k3 | 0.288 |
| hspb2 | 0.287 |
| hsbp1 | 0.287 |
| igtp | 0.287 |
| fastk | 0.287 |
| cdc42ep2 | 0.287 |
| jdp2 | 0.287 |
| rcan1 | 0.287 |
| cyct | 0.287 |
| ihpk2 | 0.287 |
| cdca7l | 0.287 |
| ecel1 | 0.286 |
| ldoc1 | 0.286 |
| ablim3 | 0.286 |
| gadd45gip1 | 0.286 |
| nadk | 0.286 |
| derl3 | 0.285 |
| syt17 | 0.285 |
| nrf1 | 0.285 |
| vnn1 | 0.285 |
| fosb | 0.285 |
| 1190002h23rik | 0.285 |
| mkl2 | 0.285 |
| mapk12 | 0.285 |
| map3k11 | 0.284 |
| mknk1 | 0.284 |
| hipk2 | 0.284 |
| zmiz1 | 0.284 |
| siah1b | 0.284 |
| aifm1 | 0.284 |
| dap3 | 0.284 |
| kdelc1 | 0.284 |
| pak4 | 0.284 |
| ube2j1 | 0.283 |
| ptplad1 | 0.283 |
| mterfd1 | 0.283 |
| hsp84-3 | 0.283 |
| svs4 | 0.283 |
| gtse1 | 0.282 |
| sirt3 | 0.282 |
| rhpn2 | 0.282 |
| mrpl11 | 0.282 |
| mylk3 | 0.282 |
| bat3 | 0.282 |
| arhgef18 | 0.282 |
| hspa4l | 0.282 |
| map3k12 | 0.282 |
| gas2 | 0.281 |
| mtfr1 | 0.281 |
| gpx3 | 0.281 |
| map3k4 | 0.281 |
| tmem55b | 0.28 |
| trim63 | 0.28 |
| net1 | 0.28 |
| jund | 0.28 |
| ppp1r12a | 0.28 |
| rabep2 | 0.28 |
| d10ertd610e | 0.28 |
| glo1 | 0.28 |
| 2310008h04rik | 0.28 |
| pdia3 | 0.28 |
| atr | 0.28 |
| akt1s1 | 0.279 |
| ifi47 | 0.279 |
| ksr1 | 0.279 |
| nisch | 0.279 |
| enc1 | 0.279 |
| akirin2 | 0.279 |
| pnpt1 | 0.279 |
| edem3 | 0.279 |
| map3k2 | 0.278 |
| cyb5r4 | 0.278 |
| cfl1 | 0.278 |
| tesk2 | 0.278 |
| klf2 | 0.277 |
| map3k3 | 0.277 |
| gpx2 | 0.277 |
| stk17b | 0.277 |
| nmral1 | 0.277 |
| ptrh2 | 0.277 |
| rhot2 | 0.276 |
| coq2 | 0.276 |
| bri3bp | 0.276 |
| egln2 | 0.276 |
| myocd | 0.276 |
| vrk1 | 0.276 |
| mdc1 | 0.276 |
| mapk8ip1 | 0.276 |
| zc3h12a | 0.276 |
| hspa4 | 0.276 |
| gpr39 | 0.275 |
| ir4 | 0.275 |
| lypd1 | 0.275 |
| map3k10 | 0.275 |
| klhl32 | 0.275 |
| gps2 | 0.275 |
| banp | 0.275 |
| bre | 0.275 |
| fbxo32 | 0.275 |
| phb | 0.275 |
| asna1 | 0.274 |
| dusp6 | 0.274 |
| foxo4 | 0.274 |
| rrm2 | 0.274 |
| tg(bcl2l1)1cbt | 0.274 |
| hisppd2a | 0.274 |
| zfp346 | 0.274 |
| aliq2 | 0.274 |
| mapk7 | 0.274 |
| arhgef15 | 0.274 |
| au041133 | 0.273 |
| loc384848 | 0.273 |
| rbm38 | 0.273 |
| d6mit183 | 0.273 |
| ahsa1 | 0.273 |
| pgrmc1 | 0.273 |
| plekho1 | 0.273 |
| zfp641 | 0.273 |
| fbxo45 | 0.273 |
| cad | 0.273 |
| bag1 | 0.273 |
| hbxip | 0.273 |
| slc25a27 | 0.273 |
| metap2 | 0.272 |
| trp53bp1 | 0.272 |
| mb | 0.272 |
| aco1 | 0.272 |
| tada3l | 0.272 |
| hsf4 | 0.272 |
| map4k3 | 0.272 |
| nhlrc1 | 0.272 |
| ucn3 | 0.271 |
| mirn320 | 0.271 |
| tram1l1 | 0.271 |
| mirn34a | 0.271 |
| elk1 | 0.271 |
| egln1 | 0.271 |
| dnajb2 | 0.271 |
| hisppd1 | 0.271 |
| trib2 | 0.27 |
| tnfrsf12a | 0.27 |
| rnf7 | 0.27 |
| dyrk1b | 0.27 |
| g3bp1 | 0.27 |
| rad1 | 0.27 |
| txnrd2 | 0.27 |
| ireb2 | 0.27 |
| aliq4 | 0.27 |
| cytb | 0.269 |
| 2810417h13rik | 0.269 |
| cradd | 0.269 |
| nps | 0.269 |
| usp3 | 0.269 |
| arl6ip1 | 0.269 |
| tnfaip8 | 0.269 |
| gpr22 | 0.269 |
| slc38a2 | 0.269 |
| prkd2 | 0.269 |
| cdc25a | 0.268 |
| atad5 | 0.268 |
| gpx4 | 0.268 |
| ripk5 | 0.268 |
| keap1 | 0.268 |
| ccdc85b | 0.268 |
| dnaja4 | 0.268 |
| gpr4 | 0.268 |
| dusp3 | 0.268 |
| ras | 0.268 |
| niban | 0.268 |
| stard4 | 0.267 |
| ascc1 | 0.267 |
| ndrg1 | 0.267 |
| ndufb4 | 0.267 |
| hmox2 | 0.267 |
| ksr2 | 0.267 |
| arhgap24 | 0.267 |
| antxr1 | 0.267 |
| elk3 | 0.267 |
| ifrd1 | 0.267 |
| park7 | 0.267 |
| tpp2 | 0.267 |
| ncoa7 | 0.267 |
| cdk5rap3 | 0.266 |
| gdf15 | 0.266 |
| ssh1 | 0.266 |
| rpl11 | 0.266 |
| rps6ka5 | 0.266 |
| rnd1 | 0.266 |
| naprt1 | 0.266 |
| arhgef2 | 0.266 |
| rpn2 | 0.266 |
| ifi27 | 0.266 |
| pak1ip1 | 0.265 |
| stra13 | 0.265 |
| cirbp-rs2 | 0.265 |
| ifi204 | 0.265 |
| zfp143 | 0.265 |
| sgms1 | 0.265 |
| casp4 | 0.265 |
| igh-pc | 0.265 |
| thrsp | 0.265 |
| sepw1 | 0.265 |
| sirt7 | 0.265 |
| mafg | 0.264 |
| mapk4 | 0.264 |
| rasgrf2 | 0.264 |
| aliq3 | 0.264 |
| xdh | 0.264 |
| nek11 | 0.264 |
| trex1 | 0.264 |
| stub1 | 0.264 |
| stc1 | 0.263 |
| atf1 | 0.263 |
| mapk8ip3 | 0.263 |
| hsf2 | 0.263 |
| ifi35 | 0.263 |
| rrn3 | 0.263 |
| sif3 | 0.263 |
| sif1 | 0.263 |
| sif2 | 0.263 |
| zscan4d | 0.263 |
| adnp | 0.263 |
| nptxr | 0.263 |
| nck2 | 0.263 |
| afap1 | 0.262 |
| map4k1 | 0.262 |
| 6030408c04rik | 0.262 |
| fosl1 | 0.262 |
| bag2 | 0.262 |
| traf7 | 0.262 |
| ddah1 | 0.262 |
| gfer | 0.262 |
| cyfip2 | 0.262 |
| ccng1 | 0.261 |
| palld | 0.261 |
| ankrd2 | 0.261 |
| nqo2 | 0.261 |
| nfyc | 0.261 |
| stk3 | 0.261 |
| tpm4 | 0.261 |
| vasp | 0.261 |
| sgpl1 | 0.261 |
| naif1 | 0.261 |
| sh3bgrl3 | 0.261 |
| cdc42se1 | 0.261 |
| ucn | 0.26 |
| armet | 0.26 |
| tial1 | 0.26 |
| fer | 0.26 |
| fhl3 | 0.26 |
| mpv17l | 0.26 |
| gna12 | 0.26 |
| mirn709 | 0.26 |
| tgfb1i1 | 0.26 |
| stk39 | 0.26 |
| dgka | 0.26 |
| crnn | 0.26 |
| mylk | 0.26 |
| irg1 | 0.259 |
| cfl2 | 0.259 |
| trim55 | 0.259 |
| zyx | 0.259 |
| gss | 0.259 |
| ndrg4 | 0.259 |
| pawr | 0.259 |
| dusp19 | 0.259 |
| mapk10 | 0.259 |
| apbb2 | 0.259 |
| crtc1 | 0.259 |
| ndufa13 | 0.259 |
| nfya | 0.259 |
| htatip2 | 0.259 |
| pank4 | 0.259 |
| 1700020c11rik | 0.259 |
| csrp3 | 0.259 |
| aven | 0.258 |
| hspa9 | 0.258 |
| def6 | 0.258 |
| atf7 | 0.258 |
| crhbp | 0.258 |
| hspb1 | 0.258 |
| zc3h15 | 0.258 |
| mus81 | 0.258 |
| trp53rk | 0.258 |
| vcp | 0.258 |
| dnm1l | 0.258 |
| gulo | 0.258 |
| crtc2 | 0.258 |
| acin1 | 0.258 |
| itgb1bp2 | 0.258 |
| rgs3 | 0.258 |
| btg1 | 0.257 |
| tmem49 | 0.257 |
| ankrd23 | 0.257 |
| bok | 0.257 |
| dusp16 | 0.257 |
| mycs | 0.257 |
| nr4a3 | 0.257 |
| dnaja3 | 0.257 |
| brap | 0.257 |
| sel1l | 0.257 |
| rhob | 0.257 |
| spred2 | 0.257 |
| abhd1 | 0.257 |
| elavl1 | 0.256 |
| nfatc4 | 0.256 |
| naca | 0.256 |
| oxsr1 | 0.256 |
| smox | 0.256 |
| spdya | 0.256 |
| d0h4s114 | 0.256 |
| pgam5 | 0.256 |
| 6330569m22rik | 0.256 |
| d330017j20rik | 0.256 |
| ppp1r12c | 0.255 |
| rchy1 | 0.255 |
| clpb | 0.255 |
| parg | 0.255 |
| pak7 | 0.255 |
| d2wsu81e | 0.255 |
| rpap3 | 0.255 |
| cebpz | 0.255 |
| mapkap1 | 0.255 |
| cdc42ep1 | 0.255 |
| mirn146 | 0.255 |
| tas2r107 | 0.254 |
| c230052i12rik | 0.254 |
| rhpn1 | 0.254 |
| ergic3 | 0.254 |
| stc2 | 0.254 |
| ndufb2 | 0.254 |
| dffa | 0.254 |
| rsad2 | 0.254 |
| bnip2 | 0.254 |
| alkbh1 | 0.254 |
| ir3 | 0.254 |
| fhl1 | 0.254 |
| c230093n12rik | 0.254 |
| gpr132 | 0.254 |
| zfand6 | 0.253 |
| ppp2r4 | 0.253 |
| alas1 | 0.253 |
| anp32b | 0.253 |
| nkap | 0.253 |
| trp53bp2 | 0.253 |
| secisbp2 | 0.253 |
| pak6 | 0.253 |
| lasp1 | 0.253 |
| rhod | 0.253 |
| htra2 | 0.253 |
| gsr | 0.253 |
| 1700007b13rik | 0.253 |
| rreb1 | 0.252 |
| wsb1 | 0.252 |
| ppm1k | 0.252 |
| tg(krt14-cre)1efu | 0.252 |
| ankrd1 | 0.252 |
| tnik | 0.252 |
| diap1 | 0.252 |
| 1200009f10rik | 0.252 |
| pdcd2l | 0.252 |
| pxdn | 0.252 |
| gsn | 0.251 |
| tesk1 | 0.251 |
| pkn1 | 0.251 |
| trip6 | 0.251 |
| prkrip1 | 0.251 |
| tpt1 | 0.251 |
| fem1b | 0.251 |
| shb | 0.251 |
| mafa | 0.251 |
| fkbp3 | 0.251 |
| rexo4 | 0.251 |
| nox4 | 0.251 |
| trappc9 | 0.251 |
| ddah2 | 0.251 |
| plrg1 | 0.251 |
| cyc1 | 0.251 |
| git2 | 0.251 |
| sertad3 | 0.25 |
| p4hb | 0.25 |
| maf1 | 0.25 |
| tmem166 | 0.25 |
| ripk3 | 0.25 |
| gimap5 | 0.25 |
| rbck1 | 0.25 |
| lancl2 | 0.25 |
| limk2 | 0.25 |
| tnni3k | 0.25 |
| arhgap1 | 0.25 |
| 4932441k18rik | 0.25 |
| cald1 | 0.25 |
| ppid | 0.25 |
| akirin1 | 0.25 |
| abl2 | 0.25 |
| amfr | 0.25 |
| hax1 | 0.25 |
| mrpl41 | 0.25 |
| zc3h12b | 0.25 |
| sdf2l1 | 0.25 |
| hpi2 | 0.25 |
| d5mit391 | 0.25 |
| hpi1 | 0.25 |
| d13mit17 | 0.25 |
| map2k4 | 0.25 |
| orf9 | 0.25 |
| zbtb17 | 0.249 |
| serpinh1 | 0.249 |
| sars2 | 0.249 |
| pdlim2 | 0.249 |
| lima1 | 0.249 |
| pcnp | 0.249 |
| tria1 | 0.249 |
| tria2 | 0.249 |
| avpr1b | 0.249 |
| mfn2 | 0.249 |
| d15mit100 | 0.249 |
| siah1a | 0.249 |
| dusp7 | 0.249 |
| arhgef1 | 0.249 |
| rybp | 0.249 |
| ulbp1 | 0.249 |
| hspa13 | 0.249 |
| grsf1 | 0.249 |
| mirn143 | 0.249 |
| daxx | 0.249 |
| bcap31 | 0.249 |
| kin | 0.248 |
| pdcd5 | 0.248 |
| sarm1 | 0.248 |
| coq7 | 0.248 |
| penk-rs | 0.248 |
| arhgef11 | 0.248 |
| arhgdia | 0.248 |
| mars | 0.248 |
| rasd1 | 0.248 |
| rhot1 | 0.248 |
| ciapin1 | 0.248 |
| dgkz | 0.248 |
| utp11l | 0.248 |
| dyrk2 | 0.248 |
| adnp2 | 0.248 |
| sirt2 | 0.248 |
| mirn21 | 0.248 |
| zfp148 | 0.248 |
| gna13 | 0.247 |
| crtc3 | 0.247 |
| bc067047 | 0.247 |
| actg2 | 0.247 |
| rgs16 | 0.247 |
| gper | 0.247 |
| ppm1f | 0.247 |
| aplnr | 0.247 |
| btg2 | 0.247 |
| akr1b3 | 0.247 |
| rps19bp1 | 0.247 |
| btbd14b | 0.247 |
| ifi205 | 0.247 |
| tnfrsf21 | 0.247 |
| ugcgl2 | 0.247 |
| cnksr1 | 0.247 |
| mfn1 | 0.247 |
| uimc1 | 0.247 |
| bag4 | 0.246 |
| eif2b5 | 0.246 |
| tmbim4 | 0.246 |
| d8ertd82e | 0.246 |
| pank3 | 0.246 |
| crhr2 | 0.246 |
| gucy1a3 | 0.246 |
| dusp26 | 0.246 |
| calr | 0.246 |
| cdc42bpa | 0.246 |
| cse1l | 0.246 |
| ifit3 | 0.246 |
| cyp2a5 | 0.246 |
| eif1 | 0.246 |
| dap | 0.246 |
| chmp1a | 0.245 |
| zfp36l2 | 0.245 |
| zfp383 | 0.245 |
| os9 | 0.245 |
| smek2 | 0.245 |
| ube2j2 | 0.245 |
| akap13 | 0.245 |
| t(2;5)43ad | 0.245 |
| t(2;5)43ad | 0.245 |
| map4k4 | 0.245 |
| rsu1 | 0.245 |
| dhrs2 | 0.245 |
| paox | 0.245 |
| cabin1 | 0.245 |
| retsat | 0.245 |
| cox2 | 0.245 |
| becn1 | 0.245 |
| nkrf | 0.245 |
| steap3 | 0.245 |
| mirn199a-1 | 0.245 |
| rgn | 0.245 |
| tnip2 | 0.245 |
| aa408296 | 0.244 |
| map4k2 | 0.244 |
| rhou | 0.244 |
| gas2l1 | 0.244 |
| zfand2b | 0.244 |
| hsh2d | 0.244 |
| dnaja1 | 0.244 |
| bc005685 | 0.244 |
| sdpr | 0.244 |
| arc | 0.244 |
| mtpn | 0.244 |
| 2010109i03rik | 0.244 |
| cdgap | 0.243 |
| cd8mts2 | 0.243 |
| als2cr2 | 0.243 |
| mrps35 | 0.243 |
| rpl23 | 0.243 |
| scye1 | 0.243 |
| prlh | 0.243 |
| top3b | 0.243 |
| pbk | 0.243 |
| cmpk2 | 0.243 |
| gtf2i | 0.243 |
| ccng2 | 0.243 |
| trim16 | 0.243 |
| psmb5 | 0.243 |
| itgb3bp | 0.243 |
| grlf1 | 0.243 |
| pdcd4 | 0.243 |
| slc25a14 | 0.243 |
| rnf34 | 0.243 |
| tnk1 | 0.242 |
| nlrx1 | 0.242 |
| rasgrp3 | 0.242 |
| cpeb1 | 0.242 |
| rtkn | 0.242 |
| hrsp12 | 0.242 |
| map2k1ip1 | 0.242 |
| scg2 | 0.242 |
| atg5 | 0.242 |
| creg1 | 0.242 |
| batf | 0.242 |
| akr1b7 | 0.242 |
| ottmusg00000005723 | 0.242 |
| olfr749 | 0.242 |
| ecsit | 0.242 |
| sgpp1 | 0.242 |
| sepx1 | 0.242 |
| ard1 | 0.242 |
| rpl32 | 0.242 |
| h2afx | 0.242 |
| htr2b | 0.242 |
| pacs2 | 0.242 |
| zfp91 | 0.242 |
| bhlhb2 | 0.242 |
| ysk4 | 0.241 |
| ing2 | 0.241 |
| gltscr2 | 0.241 |
| cycs | 0.241 |
| ddb2 | 0.241 |
| huwe1 | 0.241 |
| 15-Sep | 0.241 |
| txnrd3 | 0.241 |
| ea2 | 0.241 |
| serpina1a | 0.241 |
| wfs1 | 0.241 |
| prkab1 | 0.241 |
| eps8l2 | 0.241 |
| ptpn5 | 0.241 |
| aatk | 0.24 |
| pip5k1b | 0.24 |
| htatip | 0.24 |
| map3k7ip3 | 0.24 |
| arhgdib | 0.24 |
| sorbs3 | 0.24 |
| rhog | 0.24 |
| usp18 | 0.24 |
| retnla | 0.24 |
| madd | 0.24 |
| dapk1 | 0.24 |
| elk4 | 0.24 |
| 1300018i05rik | 0.24 |
| mcph1 | 0.24 |
| nrn1 | 0.24 |
| hnrnpul1 | 0.24 |
| rcan2 | 0.24 |
| kank1 | 0.24 |
| fbxo42 | 0.24 |
| srf | 0.24 |
| aliq1 | 0.24 |
| aoc3 | 0.24 |
| ubqln1 | 0.24 |
| casp2 | 0.239 |
| mlkl | 0.239 |
| dnajc1 | 0.239 |
| csrp2 | 0.239 |
| col4a3bp | 0.239 |
| fkbp8 | 0.239 |
| wee1 | 0.239 |
| mterfd3 | 0.239 |
| gpx5 | 0.239 |
| dnajc7 | 0.239 |
| faim | 0.239 |
| serhl | 0.239 |
| rnf103 | 0.239 |
| ube2d3 | 0.239 |
| fbxo6 | 0.239 |
| 2610301g19rik | 0.238 |
| sh2d2a | 0.238 |
| zfp36 | 0.238 |
| zfp36l1 | 0.238 |
| dusp22 | 0.238 |
| sphk2 | 0.238 |
| ufm1 | 0.238 |
| camk1d | 0.238 |
| trafd1 | 0.238 |
| use1 | 0.238 |
| pdyn | 0.238 |
| slfn2 | 0.238 |
| scin | 0.238 |
| snf1lk | 0.238 |
| srfbp1 | 0.238 |
| rnd2 | 0.238 |
| gpx1 | 0.238 |
| gripap1 | 0.238 |
| ilf3 | 0.238 |
| zhx2 | 0.238 |
| ndufs4 | 0.238 |
| rapop4 | 0.238 |
| sec11a | 0.238 |
| zfp187 | 0.238 |
| elf4 | 0.238 |
| irak1bp1 | 0.238 |
| prkcbp1 | 0.238 |
| gpr3 | 0.238 |
| fosr | 0.238 |
| btg3 | 0.237 |
| myct1 | 0.237 |
| il1 | 0.237 |
