# Supplementary material for: GIT2 Acts as a Potential Keystone Protein in Functional Hypothalamic Networks Associated with Age-Related Phenotypic Changes in Rats
Source: PLoS One. 2012 May 14;7(5):e36975. doi: 10.1371/journal.pone.0036975 (PMC3351446; doi:10.1371/journal.pone.0036975)
Supplement: Table S30 — GeneIndexer latent semantic indexing (LSI) of significantly-regulated ‘Intracellular membrane-bound organelle’ GO term group. Using the GO term group ‘Intracellular membrane-bound organelle’ as an input term, a list of the top 1000 implicitly-correlated (LSI correlation score >0.1) was generated using a full genome background list. (DOC) [file pone.0036975.s034.doc]

**Table S30. GeneIndexer latent semantic indexing (LSI) of significantly-regulated ‘Intracellular membrane-bound organelle’ GO term group.** Using the GO term group ‘Intracellular membrane-bound organelle’ as an input term, a list of the top 1000 implicitly-correlated (LSI correlation score >0.1) was generated using a full genome background list.

| ***Intracellular membrane bound organelle*** |  |
| --- | --- |
|  |  |
| **Protein Symbol** | **LSI correlation score** |
| slc9a7 | 0.649 |
| kdelr3 | 0.645 |
| yif1a | 0.617 |
| tm9sf2 | 0.615 |
| yipf1 | 0.612 |
| tmed3 | 0.611 |
| yipf7 | 0.598 |
| yipf5 | 0.597 |
| laptm4a | 0.594 |
| gcc1 | 0.593 |
| sec22a | 0.591 |
| sec16a | 0.59 |
| kdelr2 | 0.588 |
| snx10 | 0.586 |
| bet1 | 0.586 |
| cog3 | 0.584 |
| sec24a | 0.583 |
| rab6b | 0.58 |
| uso1 | 0.578 |
| trappc3 | 0.577 |
| 5830411g16rik | 0.574 |
| rusc2 | 0.573 |
| wdr44 | 0.572 |
| sec24c | 0.571 |
| golt1a | 0.571 |
| 4930417m19rik | 0.569 |
| sec31a | 0.568 |
| sec24b | 0.562 |
| vps29 | 0.558 |
| gcc2 | 0.557 |
| golgb1 | 0.556 |
| bicd2 | 0.556 |
| sec24d | 0.555 |
| hook3 | 0.554 |
| scamp1 | 0.553 |
| alpk1 | 0.551 |
| coro7 | 0.551 |
| tmed2 | 0.55 |
| gosr2 | 0.549 |
| hook2 | 0.548 |
| golga1 | 0.546 |
| golga2 | 0.546 |
| slc9a6 | 0.546 |
| vps41 | 0.546 |
| golim4 | 0.545 |
| plekhb2 | 0.545 |
| 3-Mar | 0.542 |
| tbc1d5 | 0.542 |
| rilp | 0.542 |
| rn18s-rs6 | 0.541 |
| vps26b | 0.54 |
| pi4k2a | 0.538 |
| sar1a | 0.537 |
| cog4 | 0.536 |
| vapa | 0.536 |
| cog5 | 0.534 |
| rab9 | 0.533 |
| cog2 | 0.533 |
| rab33b | 0.532 |
| gosr1 | 0.53 |
| bc002199 | 0.529 |
| sec22b | 0.528 |
| golga5 | 0.528 |
| scamp5 | 0.528 |
| sec11c | 0.528 |
| rab22a | 0.528 |
| scamp2 | 0.527 |
| cog8 | 0.526 |
| rab1b | 0.526 |
| 5730596k20rik | 0.525 |
| lman2 | 0.525 |
| bicd1 | 0.524 |
| bc025076 | 0.524 |
| sec22c | 0.524 |
| rab34 | 0.523 |
| rab18 | 0.523 |
| kdelr1 | 0.522 |
| sec31b | 0.522 |
| mical3 | 0.522 |
| vps35 | 0.518 |
| mtx2 | 0.518 |
| gorasp2 | 0.517 |
| bet1l | 0.517 |
| rab43 | 0.516 |
| rab14 | 0.516 |
| tmem32 | 0.516 |
| ap3s1 | 0.515 |
| bcap29 | 0.515 |
| rabac1 | 0.513 |
| golga3 | 0.513 |
| clip3 | 0.513 |
| copb2 | 0.513 |
| spnb3 | 0.512 |
| golga4 | 0.51 |
| bcap31 | 0.51 |
| rab6 | 0.51 |
| copb1 | 0.51 |
| chmp2a | 0.51 |
| rer1 | 0.509 |
| ergic3 | 0.509 |
| cog1 | 0.509 |
| rlbp1l2 | 0.508 |
| snx4 | 0.507 |
| plekha8 | 0.507 |
| rabgap1 | 0.507 |
| cog7 | 0.506 |
| rab20 | 0.506 |
| stx18 | 0.506 |
| 2400001e08rik | 0.504 |
| ai507611 | 0.504 |
| ottmusg00000005491 | 0.504 |
| 4933417m04rik | 0.504 |
| 4921509e07rik | 0.504 |
| 1700021p22rik | 0.504 |
| rabepk | 0.503 |
| tmed10 | 0.502 |
| rab24 | 0.501 |
| rab17 | 0.501 |
| cope | 0.501 |
| 2510003e04rik | 0.5 |
| snx15 | 0.499 |
| rab8b | 0.499 |
| lman2l | 0.499 |
| vps45 | 0.497 |
| chmp6 | 0.496 |
| pigs | 0.496 |
| erlin1 | 0.495 |
| ap3s2 | 0.494 |
| vps11 | 0.493 |
| stx5a | 0.492 |
| rab2a | 0.492 |
| vps16 | 0.491 |
| ccdc88b | 0.491 |
| zfyve27 | 0.49 |
| vti1b | 0.49 |
| scamp3 | 0.489 |
| pitpnc1 | 0.488 |
| ykt6 | 0.488 |
| copa | 0.488 |
| copg | 0.488 |
| pacs2 | 0.486 |
| rab11fip5 | 0.486 |
| sec13 | 0.485 |
| 0610010k06rik | 0.484 |
| vti1a | 0.483 |
| slc25a10 | 0.483 |
| eg636544 | 0.483 |
| ktn1 | 0.483 |
| tomm22 | 0.482 |
| nrm | 0.481 |
| osbpl1a | 0.481 |
| rab9b | 0.481 |
| pld4 | 0.481 |
| 3110003a22rik | 0.48 |
| rufy1 | 0.48 |
| golph3 | 0.48 |
| alg14 | 0.479 |
| dnajc14 | 0.479 |
| snx1 | 0.478 |
| slc25a24 | 0.477 |
| rab7 | 0.477 |
| copz2 | 0.477 |
| 4632419k20rik | 0.476 |
| slc25a17 | 0.475 |
| rab11fip2 | 0.475 |
| eea1 | 0.474 |
| sec23a | 0.474 |
| fis1 | 0.472 |
| trip11 | 0.472 |
| 2-Mar | 0.472 |
| lrba | 0.471 |
| exoc8 | 0.47 |
| trappc4 | 0.469 |
| stx6 | 0.469 |
| vps18 | 0.469 |
| pcdhga3 | 0.468 |
| stx12 | 0.468 |
| kif1c | 0.466 |
| arfgef2 | 0.466 |
| rp23-433p19.11 | 0.466 |
| zfyve1 | 0.465 |
| pi4kb | 0.465 |
| tmem163 | 0.465 |
| gorasp1 | 0.465 |
| ccdc47 | 0.465 |
| gbf1 | 0.464 |
| gopc | 0.463 |
| rab8a | 0.463 |
| kif16b | 0.463 |
| rab12 | 0.463 |
| olfr937 | 0.462 |
| zfyve20 | 0.462 |
| stx16 | 0.462 |
| ap1gbp1 | 0.462 |
| rab11b | 0.462 |
| timm17b | 0.462 |
| snx32 | 0.462 |
| snx29 | 0.462 |
| snx2 | 0.461 |
| rab1 | 0.461 |
| exoc1 | 0.461 |
| sec61b | 0.461 |
| golga7 | 0.461 |
| slc2a13 | 0.461 |
| dctn2 | 0.461 |
| sec23b | 0.46 |
| cog6 | 0.46 |
| vps39 | 0.46 |
| vps25 | 0.459 |
| mal2 | 0.459 |
| stard3nl | 0.458 |
| arcn1 | 0.458 |
| kif13b | 0.458 |
| myo5b | 0.458 |
| copz1 | 0.458 |
| tomm20 | 0.458 |
| ap4b1 | 0.457 |
| m6prbp1 | 0.457 |
| exoc7 | 0.456 |
| vamp4 | 0.455 |
| scyl2 | 0.455 |
| eg434077 | 0.454 |
| rab15 | 0.454 |
| tram1 | 0.454 |
| use1 | 0.453 |
| pdzd11 | 0.453 |
| chmp4b | 0.453 |
| rab11a | 0.453 |
| dnm1l | 0.452 |
| pigw | 0.452 |
| rab11fip3 | 0.452 |
| rab4a | 0.452 |
| ap4m1 | 0.451 |
| atp6v1d | 0.45 |
| alg2 | 0.449 |
| arfgef1 | 0.449 |
| osbpl7 | 0.448 |
| vps24 | 0.447 |
| wdfy3 | 0.447 |
| lctl | 0.447 |
| ap3d1 | 0.447 |
| wdr45l | 0.447 |
| arfgap1 | 0.447 |
| plekhm2 | 0.446 |
| rhot1 | 0.446 |
| slc25a23 | 0.446 |
| pex5l | 0.446 |
| ai314180 | 0.446 |
| pacs1 | 0.446 |
| trappc6a | 0.445 |
| kif5b | 0.445 |
| samm50 | 0.445 |
| arl6ip2 | 0.444 |
| git2 | 0.444 |
| ap1g1 | 0.443 |
| acaa2 | 0.443 |
| tmco1 | 0.443 |
| mon2 | 0.443 |
| rab5b | 0.442 |
| scfd1 | 0.442 |
| vps4b | 0.441 |
| sacm1l | 0.441 |
| ap4s1 | 0.441 |
| rab11fip1 | 0.441 |
| stx7 | 0.441 |
| spire1 | 0.44 |
| rabep1 | 0.44 |
| slc25a11 | 0.44 |
| sytl2 | 0.44 |
| arfgap3 | 0.44 |
| rab40c | 0.44 |
| ehd3 | 0.44 |
| vps33a | 0.44 |
| vta1 | 0.44 |
| mppe1 | 0.439 |
| slc4a1ap | 0.439 |
| ap3m1 | 0.438 |
| rab32 | 0.438 |
| 1500003o03rik | 0.438 |
| 6330527o06rik | 0.437 |
| rab35 | 0.437 |
| npal1 | 0.437 |
| trappc6b | 0.436 |
| rassf9 | 0.436 |
| chchd6 | 0.436 |
| olfr705 | 0.435 |
| gga2 | 0.435 |
| lman1l | 0.435 |
| trappc1 | 0.434 |
| arl1 | 0.434 |
| lrp10 | 0.433 |
| rin3 | 0.433 |
| txndc14 | 0.433 |
| 2210417d09rik | 0.433 |
| gga1 | 0.433 |
| exoc2 | 0.432 |
| lamp1 | 0.432 |
| pigt | 0.431 |
| slc35a1 | 0.431 |
| exoc3 | 0.431 |
| ubb1 | 0.43 |
| vapb | 0.43 |
| gabarapl1 | 0.43 |
| rab5a | 0.429 |
| trak2 | 0.429 |
| ehd1 | 0.429 |
| pign | 0.428 |
| exoc5 | 0.428 |
| arv1 | 0.428 |
| pex16 | 0.428 |
| rab21 | 0.428 |
| ap3b2 | 0.428 |
| chmp4c | 0.427 |
| arl6ip1 | 0.427 |
| pip5k3 | 0.427 |
| vps33b | 0.426 |
| ajap1 | 0.426 |
| rilpl2 | 0.425 |
| rilpl1 | 0.425 |
| kifc3 | 0.425 |
| rab5c | 0.425 |
| col4a3bp | 0.425 |
| acbd3 | 0.425 |
| caln1 | 0.425 |
| doc2b | 0.425 |
| chmp7 | 0.425 |
| snx17 | 0.424 |
| epb4.1l2 | 0.424 |
| snx27 | 0.424 |
| atp6v0c | 0.424 |
| pex19 | 0.424 |
| atp6v1f | 0.424 |
| stx17 | 0.424 |
| au040829 | 0.424 |
| osbpl2 | 0.424 |
| 4930471m23rik | 0.423 |
| vps4a | 0.423 |
| tm9sf3 | 0.422 |
| golt1b | 0.422 |
| osbpl6 | 0.422 |
| vps26a | 0.422 |
| 4933433k01rik | 0.421 |
| slc9a9 | 0.421 |
| rpn2 | 0.421 |
| arl4c | 0.421 |
| osbp | 0.421 |
| spnb5 | 0.421 |
| micall1 | 0.421 |
| ap1m2 | 0.421 |
| timm13 | 0.421 |
| rft1 | 0.42 |
| stx8 | 0.42 |
| clcc1 | 0.42 |
| mical1 | 0.42 |
| dctn4 | 0.42 |
| syt7 | 0.419 |
| ccdc91 | 0.419 |
| erlin2 | 0.419 |
| nbea | 0.419 |
| map6d1 | 0.419 |
| dnajc5b | 0.419 |
| dynlt1 | 0.418 |
| nsfl1c | 0.418 |
| pigl | 0.418 |
| nploc4 | 0.418 |
| napb | 0.417 |
| stard3 | 0.417 |
| slc25a42 | 0.417 |
| vamp7 | 0.417 |
| sec23ip | 0.417 |
| mff | 0.417 |
| snx3 | 0.416 |
| sec61a1 | 0.416 |
| slc35d2 | 0.416 |
| klc2 | 0.416 |
| arfrp1 | 0.416 |
| nagpa | 0.416 |
| pigb | 0.416 |
| chmp5 | 0.415 |
| gga3 | 0.415 |
| clint1 | 0.415 |
| gp2 | 0.415 |
| kif1a | 0.414 |
| asna1 | 0.414 |
| timm44 | 0.414 |
| napg | 0.414 |
| pdcd6 | 0.414 |
| pitpna | 0.414 |
| sarb | 0.413 |
| pigk | 0.413 |
| ankfy1 | 0.413 |
| whdc1 | 0.413 |
| ai413782 | 0.413 |
| stom | 0.412 |
| chmp1b | 0.412 |
| syt9 | 0.412 |
| slc25a18 | 0.411 |
| mfn1 | 0.411 |
| kifc2 | 0.41 |
| cybasc3 | 0.409 |
| slc25a25 | 0.409 |
| ugcgl2 | 0.409 |
| wdr45 | 0.409 |
| fig4 | 0.409 |
| duoxa1 | 0.408 |
| rab6ip1 | 0.408 |
| rab13 | 0.408 |
| stard5 | 0.408 |
| serinc5 | 0.407 |
| slc25a41 | 0.407 |
| spcs1 | 0.407 |
| lphn1 | 0.407 |
| txndc4 | 0.407 |
| arfgap2 | 0.406 |
| megf11 | 0.406 |
| fkbp2 | 0.406 |
| pitpnb | 0.406 |
| ank3 | 0.406 |
| wipi1 | 0.405 |
| d7mit357 | 0.405 |
| kifap3 | 0.405 |
| tmem74 | 0.405 |
| mapbpip | 0.405 |
| oxa1l | 0.405 |
| rab10 | 0.405 |
| rrbp1 | 0.405 |
| rab31 | 0.405 |
| npcd | 0.404 |
| pex3 | 0.404 |
| dynlt3 | 0.404 |
| inpp5e | 0.404 |
| timm23 | 0.403 |
| snx5 | 0.403 |
| hook1 | 0.402 |
| gde1 | 0.402 |
| ckap4 | 0.402 |
| cabp7 | 0.402 |
| rtn2 | 0.401 |
| pitpnm1 | 0.401 |
| ccdc88a | 0.401 |
| clic1 | 0.401 |
| gabarap | 0.401 |
| 4831426i19rik | 0.4 |
| doc2a | 0.4 |
| clic3 | 0.4 |
| rabgef1 | 0.4 |
| vamp5 | 0.399 |
| tom1 | 0.399 |
| rufy2 | 0.399 |
| pik3r4 | 0.398 |
| nucb1 | 0.398 |
| snapin | 0.398 |
| gpaa1 | 0.398 |
| 5730410e15rik | 0.398 |
| vps53 | 0.398 |
| plekhb1 | 0.398 |
| osbpl3 | 0.397 |
| fbxo10 | 0.397 |
| tomm70a | 0.397 |
| a430083b19rik | 0.396 |
| frag1 | 0.396 |
| tmem150 | 0.396 |
| myo5c | 0.395 |
| vps36 | 0.395 |
| vdac3 | 0.395 |
| d030074e01rik | 0.395 |
| kif5c | 0.395 |
| mtch1 | 0.394 |
| dctn1 | 0.394 |
| pi4k2b | 0.394 |
| trim72 | 0.394 |
| man2c1 | 0.394 |
| slc9a5 | 0.394 |
| sycn | 0.394 |
| inpp5b | 0.394 |
| pacsin2 | 0.394 |
| 2610528k11rik | 0.394 |
| 1700019h03rik | 0.394 |
| ap4e1 | 0.394 |
| syt5 | 0.393 |
| clstn1 | 0.393 |
| pxmp2 | 0.393 |
| sdccag3 | 0.393 |
| lmbr1l | 0.393 |
| nrsn2 | 0.393 |
| rab27b | 0.393 |
| gdi2 | 0.393 |
| exoc4 | 0.393 |
| atp6v0e | 0.392 |
| exoc3l | 0.392 |
| 1700040i03rik | 0.392 |
| tpcn2 | 0.392 |
| palm | 0.392 |
| atp6v1c1 | 0.392 |
| rcn2 | 0.392 |
| mcoln2 | 0.392 |
| srp68 | 0.391 |
| tbc1d15 | 0.391 |
| spaca4 | 0.391 |
| pex11b | 0.391 |
| d1mit365 | 0.391 |
| frmd4b | 0.391 |
| ddx41 | 0.391 |
| ptpn9 | 0.39 |
| tsnaxip1 | 0.39 |
| dync1li2 | 0.39 |
| 6620401m08rik | 0.39 |
| lamp2 | 0.39 |
| rab3b | 0.39 |
| ehd2 | 0.39 |
| pex5 | 0.389 |
| syt8 | 0.389 |
| abcb10 | 0.389 |
| plekhf2 | 0.389 |
| 4432412l15rik | 0.389 |
| mctp1 | 0.389 |
| erc1 | 0.389 |
| 11-Mar | 0.389 |
| nrbp1 | 0.389 |
| gltp | 0.388 |
| syngr3 | 0.388 |
| syt6 | 0.388 |
| tm9sf1 | 0.388 |
| ehd4 | 0.388 |
| cadps | 0.388 |
| tmem30a | 0.387 |
| rab38 | 0.387 |
| 4930579c15rik | 0.386 |
| smpd4 | 0.386 |
| epb4.1l1 | 0.386 |
| trak1 | 0.386 |
| tbc1d20 | 0.386 |
| mkln1 | 0.386 |
| gabarapl2 | 0.386 |
| atg10 | 0.385 |
| clic4 | 0.385 |
| ankrd27 | 0.385 |
| atp6v0a1 | 0.385 |
| sec61g | 0.385 |
| slc36a1 | 0.384 |
| rab25 | 0.384 |
| srp54b | 0.384 |
| mrap | 0.384 |
| d230025d16rik | 0.384 |
| 1700021k19rik | 0.384 |
| ssna1 | 0.383 |
| dnajc5 | 0.383 |
| micalcl | 0.383 |
| tgoln2 | 0.383 |
| oma1 | 0.383 |
| rab33a | 0.383 |
| chchd4 | 0.383 |
| d14ertd436e | 0.383 |
| ap1m1 | 0.383 |
| abcb6 | 0.382 |
| timm9 | 0.382 |
| tmem49 | 0.382 |
| tbc1d10a | 0.382 |
| ubl3 | 0.382 |
| srp54a | 0.382 |
| arf3 | 0.382 |
| wdfy2 | 0.382 |
| ssr1 | 0.381 |
| napa | 0.381 |
| tor1aip2 | 0.381 |
| cisd1 | 0.381 |
| myo1b | 0.381 |
| srprb | 0.381 |
| rint1 | 0.381 |
| rabif | 0.381 |
| clic5 | 0.381 |
| tgoln1 | 0.381 |
| sec61a2 | 0.38 |
| vcpip1 | 0.38 |
| loc100041194 | 0.38 |
| snx16 | 0.38 |
| centa1 | 0.38 |
| 10-Mar | 0.38 |
| arl2bp | 0.38 |
| actr1a | 0.379 |
| muted | 0.379 |
| surf4 | 0.379 |
| syt17 | 0.379 |
| arl8b | 0.379 |
| hps3 | 0.378 |
| kif13a | 0.378 |
| praf2 | 0.378 |
| d9mit73 | 0.378 |
| ap1g2 | 0.378 |
| d12ertd673e | 0.378 |
| ms4a14 | 0.378 |
| pscd1 | 0.377 |
| rgs7bp | 0.377 |
| antxr2 | 0.377 |
| centd2 | 0.376 |
| ap3b1 | 0.376 |
| rab3d | 0.376 |
| centb1 | 0.376 |
| dctn3 | 0.376 |
| vdac2 | 0.376 |
| tomm7 | 0.376 |
| zdhhc3 | 0.376 |
| pef1 | 0.375 |
| slc7a6 | 0.375 |
| flot1 | 0.375 |
| arf5 | 0.375 |
| mtmr7 | 0.375 |
| pigu | 0.375 |
| sidt1 | 0.375 |
| 2310007f21rik | 0.375 |
| ftcd | 0.374 |
| ap3m2 | 0.374 |
| 3000004c01rik | 0.374 |
| nradd | 0.374 |
| gpr172b | 0.374 |
| stam2 | 0.374 |
| pscd2 | 0.374 |
| sort1 | 0.374 |
| mcoln1 | 0.374 |
| cuta | 0.374 |
| mtmr6 | 0.374 |
| tor1aip1 | 0.374 |
| edem1 | 0.374 |
| slc24a1 | 0.374 |
| slc30a2 | 0.373 |
| vdac1 | 0.373 |
| snx6 | 0.373 |
| scarb2 | 0.373 |
| sytl1 | 0.373 |
| ssr3 | 0.373 |
| arf2 | 0.373 |
| plscr2 | 0.373 |
| slc30a7 | 0.373 |
| rabggta | 0.373 |
| plscr4 | 0.372 |
| rag1ap1 | 0.372 |
| akap9 | 0.372 |
| foxred2 | 0.372 |
| ppm1l | 0.372 |
| sh3bp4 | 0.372 |
| pex14 | 0.372 |
| olfr930 | 0.372 |
| d1mit488 | 0.372 |
| atp6v1g3 | 0.371 |
| gapvd1 | 0.371 |
| ttc35 | 0.371 |
| plekha2 | 0.371 |
| bloc1s2 | 0.371 |
| arf1 | 0.371 |
| pdzd3 | 0.37 |
| paqr7 | 0.37 |
| ulk1 | 0.37 |
| pitpnm2 | 0.37 |
| atp6v1e1 | 0.37 |
| pigv | 0.37 |
| atp6v1a | 0.37 |
| bc016423 | 0.369 |
| d4mit225 | 0.369 |
| nsg1 | 0.369 |
| arl8a | 0.369 |
| snap91 | 0.369 |
| os9 | 0.369 |
| sgsm2 | 0.369 |
| slc2a8 | 0.369 |
| mettl7b | 0.368 |
| uxs1 | 0.368 |
| rab28 | 0.368 |
| timm10 | 0.368 |
| plscr3 | 0.368 |
| slc29a3 | 0.368 |
| bloc1s3 | 0.368 |
| freq | 0.368 |
| pex13 | 0.367 |
| rpn1 | 0.367 |
| letm1 | 0.367 |
| aftph | 0.367 |
| vac14 | 0.367 |
| npc2 | 0.367 |
| atp6v0a2 | 0.366 |
| snx18 | 0.366 |
| pigx | 0.366 |
| timm22 | 0.366 |
| dynll2 | 0.366 |
| copg2 | 0.366 |
| klc1 | 0.365 |
| slc31a1 | 0.365 |
| sypl | 0.365 |
| pib5pa | 0.365 |
| zdhhc13 | 0.365 |
| tmem166 | 0.365 |
| dnahc1 | 0.365 |
| ugcgl1 | 0.365 |
| map1lc3a | 0.364 |
| dnmlp1 | 0.364 |
| vamp3 | 0.364 |
| nsf | 0.364 |
| 1300012g16rik | 0.364 |
| slc35a2 | 0.364 |
| myo1a | 0.364 |
| tram1l1 | 0.364 |
| m6pr | 0.363 |
| arfip2 | 0.363 |
| pigm | 0.363 |
| mreg | 0.363 |
| aqp11 | 0.363 |
| slc35b2 | 0.363 |
| scamp4 | 0.363 |
| tmed4 | 0.362 |
| myrip | 0.362 |
| rem2 | 0.362 |
| pscd3 | 0.362 |
| arl6ip6 | 0.362 |
| vps28 | 0.362 |
| mtvr2 | 0.362 |
| rtn3 | 0.362 |
| dner | 0.362 |
| layn | 0.361 |
| vps37b | 0.361 |
| vamp8 | 0.361 |
| lgtn | 0.361 |
| doc2g | 0.36 |
| prkcsh | 0.36 |
| d6wsu116e | 0.36 |
| trim23 | 0.36 |
| zfpl1 | 0.36 |
| sytl3 | 0.36 |
| ift20 | 0.359 |
| arf4 | 0.359 |
| stam | 0.359 |
| hgs | 0.359 |
| syne1 | 0.359 |
| arf6 | 0.359 |
| atp6v0d1 | 0.358 |
| ssr2 | 0.358 |
| atp6v1e2 | 0.358 |
| pigc | 0.358 |
| slc16a10 | 0.357 |
| jph1 | 0.357 |
| pex26 | 0.357 |
| rin2 | 0.357 |
| srp72 | 0.357 |
| kif20a | 0.357 |
| akap1 | 0.357 |
| atg4a | 0.357 |
| pip5k1c | 0.357 |
| rab3gap2 | 0.356 |
| dync2li1 | 0.356 |
| d1mit84 | 0.356 |
| rab27a | 0.356 |
| rhbg | 0.356 |
| cln3 | 0.356 |
| lphn3 | 0.356 |
| dnajc13 | 0.355 |
| dgkd | 0.355 |
| snf8 | 0.355 |
| sash3 | 0.355 |
| srpr | 0.355 |
| anxa6 | 0.355 |
| txndc1 | 0.355 |
| dync1i1 | 0.355 |
| centd3 | 0.354 |
| slc35b4 | 0.354 |
| kif5a | 0.354 |
| cyb5b | 0.354 |
| sdcbp2 | 0.354 |
| akap2 | 0.353 |
| centg2 | 0.353 |
| pcdhga1 | 0.353 |
| tmem201 | 0.353 |
| d9mit133 | 0.353 |
| d9mit259 | 0.353 |
| d9mit270 | 0.353 |
| d9mit261 | 0.353 |
| p42pop | 0.353 |
| bloc1s1 | 0.353 |
| mtmr3 | 0.353 |
| ngly1 | 0.353 |
| sdf4 | 0.352 |
| gdi1 | 0.352 |
| arl2 | 0.352 |
| ubxd4 | 0.352 |
| vps37a | 0.352 |
| sec63 | 0.352 |
| erc2 | 0.352 |
| agps | 0.351 |
| slc24a2 | 0.351 |
| lrrc59 | 0.351 |
| rcn1 | 0.351 |
| ocrl | 0.351 |
| ndufb10 | 0.351 |
| slc43a2 | 0.35 |
| cd63 | 0.35 |
| tmem38b | 0.35 |
| bin3 | 0.35 |
| spert | 0.35 |
| chmp1a | 0.35 |
| anxa13 | 0.35 |
| phldb2 | 0.35 |
| mrs2 | 0.35 |
| jph4 | 0.35 |
| coro1c | 0.35 |
| d3ucla1 | 0.35 |
| rab2b | 0.35 |
| atp8a1 | 0.35 |
| cyhr1 | 0.349 |
| rsc1a1 | 0.349 |
| cpd | 0.349 |
| spg21 | 0.349 |
| dpm2 | 0.349 |
| abcd3 | 0.349 |
| rab3c | 0.349 |
| pi4ka | 0.349 |
| clta | 0.349 |
| spag9 | 0.349 |
| pex12 | 0.349 |
| mpv17l | 0.349 |
| wdr69 | 0.348 |
| anxa4 | 0.348 |
| iigp1 | 0.348 |
| myo18a | 0.348 |
| sytl4 | 0.348 |
| bcnp1 | 0.348 |
| 5-Mar | 0.348 |
| plekhm1 | 0.348 |
| ap1s3 | 0.347 |
| akap7 | 0.347 |
| mmd | 0.347 |
| paqr5 | 0.347 |
| rtn1 | 0.347 |
| rabggtb | 0.347 |
| hip1r | 0.347 |
| smap2 | 0.347 |
| tuba4a | 0.346 |
| d19mit66 | 0.346 |
| d19mit24 | 0.346 |
| slc6a17 | 0.346 |
| snph | 0.346 |
| slc30a6 | 0.346 |
| pigg | 0.346 |
| 1700054f22rik | 0.346 |
| entpd4 | 0.346 |
| lancl2 | 0.346 |
| slc7a12 | 0.346 |
| ube2j2 | 0.346 |
| dync1i2 | 0.345 |
| cno | 0.345 |
| tmem50b | 0.345 |
| git1 | 0.345 |
| plekhf1 | 0.345 |
| kif3b | 0.345 |
| sntg1 | 0.345 |
| gipc1 | 0.345 |
| dnajc1 | 0.345 |
| apba3 | 0.344 |
| timm17a | 0.344 |
| blzf1 | 0.344 |
| cerk | 0.344 |
| rph3a | 0.344 |
| unc84b | 0.344 |
| flot2 | 0.344 |
| hps6 | 0.344 |
| pxmp4 | 0.344 |
| fer1l4 | 0.343 |
| 4933407n01rik | 0.343 |
| slc7a13 | 0.343 |
| gpr89 | 0.343 |
| baiap3 | 0.343 |
| creg2 | 0.343 |
| c330002i19rik | 0.343 |
| pip5k1b | 0.343 |
| myo1c | 0.343 |
| 9430023l20rik | 0.343 |
| slmap | 0.343 |
| clcn4-2 | 0.342 |
| 1110034a24rik | 0.342 |
| trappc5 | 0.342 |
| immt | 0.342 |
| ap1s2 | 0.342 |
| tmem33 | 0.342 |
| tomm34 | 0.342 |
| atp5o | 0.342 |
| kif17 | 0.341 |
| scp2 | 0.341 |
| pgap1 | 0.341 |
| atp6v0a4 | 0.341 |
| cabp1 | 0.341 |
| gucy2g | 0.341 |
| zw10 | 0.34 |
| hpcal1 | 0.34 |
| slc6a5 | 0.34 |
| clip1 | 0.34 |
| eml2 | 0.34 |
| man2a1 | 0.34 |
| rps6kc1 | 0.34 |
| rhot2 | 0.34 |
| rem1 | 0.34 |
| snx14 | 0.339 |
| clcn3 | 0.339 |
| derl2 | 0.339 |
| rab30 | 0.339 |
| ube2j1 | 0.339 |
| hps4 | 0.339 |
| tpcn1 | 0.339 |
| ottmusg00000004461 | 0.339 |
| map1lc3b | 0.339 |
| stab1 | 0.338 |
| gnpat | 0.338 |
| plekha3 | 0.338 |
| atpif1 | 0.338 |
| slc25a21 | 0.338 |
| arhgap17 | 0.338 |
| ggnbp1 | 0.338 |
| 4930553d19rik | 0.338 |
| bola1 | 0.337 |
| bola3 | 0.337 |
| bnip1 | 0.337 |
| slc36a2 | 0.337 |
| srp19 | 0.337 |
| cdipt | 0.337 |
| strn4 | 0.336 |
| 1810010m01rik | 0.336 |
| snx9 | 0.336 |
| abcd4 | 0.336 |
| dynlrb1 | 0.336 |
| plcd4 | 0.336 |
| stoml3 | 0.336 |
| defb29 | 0.336 |
| arl3 | 0.336 |
| atg7 | 0.336 |
| svs7 | 0.335 |
| asgr2 | 0.335 |
| jakmip1 | 0.335 |
| appl2 | 0.335 |
| zfyve16 | 0.335 |
| synj2 | 0.335 |
| atp5s | 0.335 |
| 2810003c17rik | 0.335 |
| fkbp7 | 0.335 |
| rab37 | 0.335 |
| fut11 | 0.335 |
| fut10 | 0.335 |
| atp6v0b | 0.335 |
| plcl2 | 0.335 |
| slc41a1 | 0.334 |
| tmem38a | 0.334 |
| rhoq | 0.334 |
| pip5k1a | 0.334 |
| naaladl1 | 0.334 |
| arfip1 | 0.334 |
| rgs19 | 0.334 |
| klc3 | 0.334 |
| sar1b | 0.334 |
| exoc6 | 0.334 |
| slc31a2 | 0.333 |
| uqcrc2 | 0.333 |
| necab3 | 0.333 |
| dnajc6 | 0.333 |
| hps1 | 0.333 |
| samd8 | 0.333 |
| hps5 | 0.333 |
| olfr410 | 0.333 |
| glg1 | 0.333 |
| clpp | 0.333 |
| rnf24 | 0.333 |
| cltb | 0.333 |
| plek | 0.333 |
| ank1 | 0.332 |
| adcy10 | 0.332 |
| myo10 | 0.332 |
| derl1 | 0.332 |
| ms6-4 | 0.332 |
| tex101 | 0.332 |
| syt13 | 0.332 |
| pacsin3 | 0.331 |
| slc30a5 | 0.331 |
| atp2b1 | 0.331 |
| col23a1 | 0.331 |
| 0610031j06rik | 0.331 |
| arl6ip5 | 0.331 |
| snx21 | 0.331 |
| atg4b | 0.331 |
| akap6 | 0.331 |
| tmem48 | 0.331 |
| ap1s1 | 0.33 |
| abcb9 | 0.33 |
| mtap1a | 0.33 |
| epb4.1 | 0.329 |
| ap2m1 | 0.329 |
| plb1 | 0.329 |
| rp23-136k12.4 | 0.329 |
| slc25a3 | 0.329 |
| necap1 | 0.329 |
| zdhhc21 | 0.329 |
| zdhhc16 | 0.329 |
| usp30 | 0.329 |
| hax1 | 0.329 |
| atp6v1c2 | 0.328 |
| mfsd10 | 0.328 |
| hpca | 0.328 |
| mlph | 0.328 |
