# Supplementary material for: GIT2 Acts as a Potential Keystone Protein in Functional Hypothalamic Networks Associated with Age-Related Phenotypic Changes in Rats
Source: PLoS One. 2012 May 14;7(5):e36975. doi: 10.1371/journal.pone.0036975 (PMC3351446; doi:10.1371/journal.pone.0036975)
Supplement: Table S31 — GeneIndexer latent semantic indexing (LSI) of significantly-regulated ‘Regulation of programmed cell death’ GO term group. Using the GO term group ‘Regulation of programmed cell death’ as an input term, a list of the top 1000 implicitly-correlated (LSI correlation score >0.1) was generated using a full genome background list. (DOC) [file pone.0036975.s035.doc]

**Table S31. GeneIndexer latent semantic indexing (LSI) of significantly-regulated ‘Regulation of programmed cell death’ GO term group.** Using the GO term group ‘Regulation of programmed cell death’ as an input term, a list of the top 1000 implicitly-correlated (LSI correlation score >0.1) was generated using a full genome background list.

| ***Regulation of programmed cell death*** |  |
| --- | --- |
|  |  |
| **Protein Symbol** | **LSI correlation score** |
| bc005685 | 0.769 |
| tmem166 | 0.709 |
| pogk | 0.703 |
| 4932432k03rik | 0.653 |
| 1110008f13rik | 0.653 |
| tmem85 | 0.651 |
| plekhf2 | 0.645 |
| dap | 0.643 |
| tmbim4 | 0.642 |
| spns1 | 0.637 |
| spin2 | 0.624 |
| plekhf1 | 0.618 |
| 2810002n01rik | 0.616 |
| ptrh2 | 0.611 |
| pdcl3 | 0.609 |
| faim2 | 0.6 |
| 5730403b10rik | 0.592 |
| zfp319 | 0.591 |
| rhbdd1 | 0.591 |
| faim | 0.59 |
| bhlhb9 | 0.587 |
| utp11l | 0.583 |
| d2wsu81e | 0.579 |
| bri3bp | 0.577 |
| siva1 | 0.577 |
| naif1 | 0.575 |
| bnipl | 0.573 |
| tfpt | 0.573 |
| faim3 | 0.568 |
| bok | 0.567 |
| acin1 | 0.567 |
| apip | 0.566 |
| moap1 | 0.566 |
| ldoc1 | 0.556 |
| ptchd2 | 0.554 |
| 1700020c11rik | 0.553 |
| mrpl41 | 0.553 |
| aifm2 | 0.552 |
| iap2 | 0.552 |
| cradd | 0.551 |
| pus10 | 0.551 |
| gramd4 | 0.549 |
| dedd2 | 0.548 |
| larp6 | 0.546 |
| loc100046187 | 0.542 |
| endog | 0.541 |
| ripk5 | 0.541 |
| trp53i11 | 0.54 |
| lrdd | 0.54 |
| dap3 | 0.539 |
| bcl2l13 | 0.539 |
| 1200002n14rik | 0.537 |
| wdr26 | 0.537 |
| tmem123 | 0.535 |
| chac1 | 0.535 |
| 2310056p07rik | 0.534 |
| ambra1 | 0.534 |
| ypel3 | 0.534 |
| higd1a | 0.529 |
| nol3 | 0.529 |
| bnip1 | 0.527 |
| aifm3 | 0.527 |
| bnip3l | 0.526 |
| 2010100o12rik | 0.525 |
| shisa5 | 0.524 |
| dffa | 0.524 |
| hrk | 0.523 |
| tnfaip8 | 0.522 |
| apol6 | 0.52 |
| trp53i13 | 0.52 |
| rbm45 | 0.52 |
| magea4 | 0.519 |
| sltm | 0.519 |
| 3230401d17rik | 0.518 |
| 5830417c01rik | 0.516 |
| khdc1b | 0.515 |
| cntd1 | 0.514 |
| tegt | 0.512 |
| fem1b | 0.512 |
| uaca | 0.512 |
| zdhhc16 | 0.511 |
| ppm1k | 0.511 |
| rhbdd3 | 0.51 |
| trim69 | 0.51 |
| phlda1 | 0.51 |
| atg4d | 0.509 |
| bcl2l10 | 0.509 |
| ghitm | 0.508 |
| 6720467c03rik | 0.508 |
| mtch1 | 0.505 |
| ei24 | 0.503 |
| slc37a3 | 0.502 |
| 4632434i11rik | 0.502 |
| pinc | 0.501 |
| d530049i02rik | 0.501 |
| isg20l1 | 0.499 |
| cideb | 0.499 |
| siah1b | 0.499 |
| birc6 | 0.499 |
| tg(bcl2l1)1cbt | 0.498 |
| qsox2 | 0.497 |
| dedd | 0.497 |
| bcl2l15 | 0.497 |
| arl6ip1 | 0.497 |
| ippk | 0.495 |
| ccdc86 | 0.494 |
| d15mit198 | 0.494 |
| mrpl36 | 0.494 |
| sav1 | 0.493 |
| pdcd5 | 0.493 |
| api5 | 0.492 |
| pdcd11 | 0.492 |
| zfp612 | 0.491 |
| perp | 0.491 |
| serinc3 | 0.491 |
| zmym4 | 0.49 |
| mrpl13 | 0.49 |
| pcnp | 0.49 |
| rnf34 | 0.49 |
| gnpnat1 | 0.487 |
| spns2 | 0.487 |
| eif3m | 0.486 |
| gimap8 | 0.486 |
| dffb | 0.486 |
| fastk | 0.485 |
| tnfrsf23 | 0.484 |
| tnfrsf21 | 0.484 |
| gimap4 | 0.484 |
| 2010109i03rik | 0.483 |
| plscr2 | 0.48 |
| efhd1 | 0.48 |
| rpap3 | 0.479 |
| smpd4 | 0.479 |
| dido1 | 0.479 |
| sdccag3 | 0.479 |
| them4 | 0.479 |
| traf3ip3 | 0.478 |
| 6030408c04rik | 0.478 |
| timm23 | 0.477 |
| bmf | 0.476 |
| cmtm8 | 0.475 |
| mycs | 0.474 |
| gltscr2 | 0.474 |
| cmtm5 | 0.473 |
| rhox3a | 0.473 |
| rhox2a | 0.473 |
| faf1 | 0.472 |
| madd | 0.472 |
| bcl2l14 | 0.472 |
| trim35 | 0.472 |
| naip4 | 0.472 |
| bik | 0.471 |
| etnk1 | 0.471 |
| dpf2 | 0.47 |
| dbndd2 | 0.47 |
| prodh2 | 0.468 |
| pea15b | 0.468 |
| a230083h22rik | 0.468 |
| eral1 | 0.466 |
| 2500003m10rik | 0.466 |
| dad1 | 0.466 |
| rnf122 | 0.466 |
| mageh1 | 0.466 |
| spata17 | 0.465 |
| tg(bcl2)1tsk | 0.465 |
| mllt11 | 0.465 |
| hsp84-3 | 0.464 |
| 2010001m09rik | 0.464 |
| gas2 | 0.464 |
| cstad | 0.463 |
| ciapin1 | 0.463 |
| bnip2 | 0.462 |
| sh3bgrl3 | 0.461 |
| d1mit65 | 0.461 |
| bclaf1 | 0.461 |
| gas5 | 0.461 |
| stk17b | 0.46 |
| scara3 | 0.46 |
| stk3 | 0.459 |
| ccdc85b | 0.459 |
| pxdn | 0.459 |
| triap1 | 0.459 |
| lcmt1 | 0.458 |
| med29 | 0.458 |
| serpina3g | 0.457 |
| zc3h15 | 0.457 |
| 1700081d17rik | 0.456 |
| rbm5 | 0.456 |
| cyc1 | 0.456 |
| ddit4l | 0.455 |
| tmem102 | 0.455 |
| ripk3 | 0.455 |
| aven | 0.455 |
| zmat3 | 0.454 |
| bcl2l2 | 0.453 |
| dnase1l3 | 0.453 |
| tnfaip8l2 | 0.45 |
| lats2 | 0.45 |
| pdcd2l | 0.45 |
| tmem161a | 0.448 |
| snora69 | 0.448 |
| gimap3 | 0.448 |
| vps53 | 0.447 |
| tcf25 | 0.447 |
| apitd1 | 0.446 |
| aifm1 | 0.446 |
| bnip3 | 0.446 |
| ube2q2 | 0.445 |
| thoc1 | 0.445 |
| 1200009f10rik | 0.445 |
| zbtb38 | 0.445 |
| casp2 | 0.445 |
| rassf6 | 0.444 |
| adnp2 | 0.444 |
| sec16b | 0.443 |
| wfdc5 | 0.443 |
| mrs2 | 0.443 |
| ngfrap1 | 0.443 |
| glipr1 | 0.443 |
| nrbp2 | 0.443 |
| frag1 | 0.443 |
| ihpk2 | 0.442 |
| pmaip1 | 0.442 |
| cirbp-rs3 | 0.442 |
| cirbp-rs1 | 0.442 |
| mul1 | 0.441 |
| casp8ap2 | 0.441 |
| rapop4 | 0.441 |
| birc1f | 0.441 |
| jmjd6 | 0.441 |
| tgm7 | 0.44 |
| naip2 | 0.44 |
| mtch2 | 0.44 |
| aatf | 0.439 |
| rps27l | 0.439 |
| dapk3 | 0.438 |
| znhit1 | 0.438 |
| khdc1a | 0.438 |
| nradd | 0.438 |
| cab39l | 0.438 |
| nkap | 0.437 |
| osgin1 | 0.437 |
| ddx18 | 0.437 |
| lypd1 | 0.436 |
| bbc3 | 0.436 |
| sesn2 | 0.436 |
| fis1 | 0.435 |
| dph5 | 0.434 |
| tg(bcl2l1)2cbt | 0.434 |
| spata3 | 0.434 |
| fau | 0.434 |
| rnf130 | 0.433 |
| trp53inp1 | 0.433 |
| wdr92 | 0.433 |
| tm2d2 | 0.433 |
| top1-rs1 | 0.432 |
| trib2 | 0.432 |
| naip3 | 0.432 |
| zc3h10 | 0.432 |
| dnm1l | 0.432 |
| zfp110 | 0.432 |
| tmem9 | 0.432 |
| 9630033f20rik | 0.432 |
| stk4 | 0.431 |
| 2810417h13rik | 0.431 |
| nsmaf | 0.431 |
| htra2 | 0.431 |
| birc5l | 0.431 |
| tmem49 | 0.43 |
| gimap7 | 0.43 |
| gimap9 | 0.43 |
| becn1 | 0.43 |
| zswim2 | 0.43 |
| ier3 | 0.43 |
| prdm4 | 0.43 |
| bag4 | 0.429 |
| cdr2 | 0.429 |
| rybp | 0.429 |
| carp1 | 0.429 |
| utp6 | 0.429 |
| sh3rf1 | 0.428 |
| bcl2a1a | 0.428 |
| letm1 | 0.428 |
| casz1 | 0.428 |
| casp12 | 0.427 |
| erdr1 | 0.427 |
| ctsq | 0.426 |
| casp14 | 0.426 |
| casp4 | 0.426 |
| ppp2r3c | 0.425 |
| gimap6 | 0.425 |
| dnaja3 | 0.425 |
| ints4 | 0.425 |
| 6430548m08rik | 0.425 |
| eamcd2 | 0.425 |
| apaf1 | 0.425 |
| plac8 | 0.425 |
| 6330500d04rik | 0.425 |
| mirn320 | 0.424 |
| rhbdl1 | 0.424 |
| unc5d | 0.423 |
| capop | 0.423 |
| ccar1 | 0.423 |
| aktip | 0.423 |
| bre | 0.423 |
| gas1 | 0.422 |
| msto1 | 0.422 |
| rbm10 | 0.422 |
| ppm1h | 0.422 |
| slfn5 | 0.422 |
| nle1 | 0.421 |
| bri3 | 0.421 |
| rffl | 0.42 |
| efhd2 | 0.42 |
| zfp385a | 0.42 |
| yb1a | 0.42 |
| tmed4 | 0.42 |
| tnfrsf25 | 0.42 |
| zc3h8 | 0.42 |
| zfp449 | 0.419 |
| slfn9 | 0.419 |
| slfn4 | 0.419 |
| rmst | 0.419 |
| ay074887 | 0.419 |
| tmem131 | 0.418 |
| hipk1 | 0.418 |
| mmd | 0.418 |
| st18 | 0.417 |
| rpl13a | 0.417 |
| slfn7 | 0.417 |
| slfn14 | 0.417 |
| slfn6 | 0.417 |
| ndufa13 | 0.417 |
| slfn8 | 0.417 |
| map3k9 | 0.416 |
| gimap1 | 0.416 |
| blcap | 0.416 |
| ifi205 | 0.415 |
| aatk | 0.415 |
| cyct | 0.415 |
| trp53bp2 | 0.415 |
| sall2 | 0.415 |
| dapk2 | 0.415 |
| stk38l | 0.414 |
| laptm5 | 0.414 |
| ubtd2 | 0.414 |
| slfn10 | 0.414 |
| apbb2 | 0.413 |
| bcap31 | 0.413 |
| plscr3 | 0.412 |
| taok3 | 0.412 |
| g0s2 | 0.412 |
| oxr1 | 0.412 |
| dus2l | 0.411 |
| ddx47 | 0.411 |
| trap1 | 0.411 |
| sgms1 | 0.411 |
| zfp395 | 0.411 |
| zfand2a | 0.411 |
| ifi202b | 0.411 |
| loc100034726 | 0.41 |
| ccdc72 | 0.41 |
| tpt1p | 0.41 |
| bag3 | 0.41 |
| 0610007c21rik | 0.41 |
| rbbp6 | 0.41 |
| gfral | 0.41 |
| nat13 | 0.409 |
| tnk1 | 0.409 |
| d14mit116 | 0.409 |
| iap5ra1 | 0.408 |
| anp32b | 0.408 |
| hsph1 | 0.408 |
| hmgb1-rs6 | 0.408 |
| tradd | 0.407 |
| rps13 | 0.407 |
| pnpt1 | 0.407 |
| bc010304 | 0.407 |
| rtkn2 | 0.407 |
| mff | 0.407 |
| lats1 | 0.406 |
| birc7 | 0.406 |
| lgals12 | 0.406 |
| cycs | 0.406 |
| stk24 | 0.406 |
| 9130213b05rik | 0.405 |
| slc22a17 | 0.405 |
| plk2 | 0.405 |
| bc048355 | 0.404 |
| alkbh8 | 0.404 |
| ensmusg00000069351 | 0.404 |
| fignl1 | 0.404 |
| ier2 | 0.404 |
| usp28 | 0.404 |
| tsc22d4 | 0.403 |
| nme3 | 0.403 |
| mmd2 | 0.403 |
| ars2 | 0.403 |
| dapk1 | 0.403 |
| trp53rk | 0.403 |
| eif3g | 0.403 |
| ergic2 | 0.403 |
| eg621324 | 0.403 |
| ppif | 0.402 |
| mterfd3 | 0.402 |
| 4931417g12rik | 0.402 |
| atg5 | 0.402 |
| mirn7b | 0.401 |
| pawr | 0.401 |
| csrnp3 | 0.401 |
| mirn34a | 0.401 |
| ints1 | 0.401 |
| plrg1 | 0.401 |
| gadd45g | 0.4 |
| 4930420k17rik | 0.4 |
| 4930471m23rik | 0.4 |
| cdc2l1 | 0.4 |
| bc063263 | 0.4 |
| plagl2 | 0.4 |
| pea15a | 0.4 |
| hm | 0.4 |
| prdx4 | 0.4 |
| parp4 | 0.4 |
| traip | 0.4 |
| thg1l | 0.399 |
| zfp668 | 0.399 |
| mobkl1b | 0.399 |
| trpd52l3 | 0.399 |
| traf4 | 0.399 |
| 1200015f23rik | 0.399 |
| zbtb4 | 0.399 |
| 1110006o17rik | 0.399 |
| gas4 | 0.399 |
| clic4 | 0.398 |
| dnase1l2 | 0.398 |
| sesn1 | 0.398 |
| shf | 0.398 |
| akirin2 | 0.398 |
| ern2 | 0.398 |
| npas4 | 0.397 |
| ern1 | 0.397 |
| sh3glb1 | 0.397 |
| card6 | 0.397 |
| clca2 | 0.397 |
| mkrn1 | 0.397 |
| hsh2d | 0.396 |
| agbl2 | 0.396 |
| stk33 | 0.396 |
| rhot2 | 0.396 |
| trim39 | 0.396 |
| smpd2 | 0.395 |
| myg1 | 0.395 |
| thap11 | 0.395 |
| steap3 | 0.395 |
| pptc7 | 0.395 |
| qars | 0.395 |
| olfml1 | 0.394 |
| gulp1 | 0.394 |
| gpr65 | 0.394 |
| mrps11 | 0.394 |
| tmem55b | 0.394 |
| diablo | 0.394 |
| gadd45gip1 | 0.394 |
| amigo2 | 0.394 |
| commd7 | 0.394 |
| ai462493 | 0.393 |
| ndufs5 | 0.393 |
| slc25a33 | 0.393 |
| relt | 0.393 |
| fbxo45 | 0.393 |
| nit1 | 0.393 |
| ift57 | 0.392 |
| casp6 | 0.392 |
| nupr1 | 0.392 |
| myct1 | 0.392 |
| pdcd2 | 0.392 |
| d9mit223 | 0.392 |
| nuak1 | 0.392 |
| kank4 | 0.391 |
| kank3 | 0.391 |
| kctd11 | 0.391 |
| itm2c | 0.391 |
| tax1bp1 | 0.391 |
| hbxip | 0.391 |
| gadd45b | 0.391 |
| atg7 | 0.391 |
| hax1 | 0.39 |
| htatip2 | 0.39 |
| rpl35a | 0.389 |
| fkbp8 | 0.389 |
| megf11 | 0.389 |
| gimap5 | 0.389 |
| trappc9 | 0.389 |
| rai12 | 0.389 |
| pdcd7 | 0.389 |
| ppp1r1c | 0.389 |
| nenf | 0.389 |
| mfn1 | 0.389 |
| cdca7l | 0.389 |
| gtse1 | 0.389 |
| pnma5 | 0.389 |
| pp11r | 0.388 |
| eg435755 | 0.388 |
| maged1 | 0.388 |
| gmppb | 0.388 |
| mobkl1a | 0.388 |
| dub2 | 0.388 |
| hyou1 | 0.388 |
| ier3ip1 | 0.388 |
| fastkd2 | 0.387 |
| zfp622 | 0.387 |
| ifi27 | 0.387 |
| tusc4 | 0.387 |
| slc35c2 | 0.387 |
| myd116 | 0.387 |
| mirn221 | 0.387 |
| mirn222 | 0.387 |
| map4k3 | 0.387 |
| gspt1 | 0.387 |
| ddit4 | 0.386 |
| ifi35 | 0.386 |
| melk | 0.386 |
| tgm6 | 0.386 |
| ing4 | 0.386 |
| gas2l1 | 0.385 |
| d9mit60 | 0.385 |
| d9mit61 | 0.385 |
| ndufb2 | 0.385 |
| 1110005a23rik | 0.385 |
| cse1l | 0.385 |
| tex261 | 0.385 |
| cdk2ap1 | 0.385 |
| d10mit69 | 0.385 |
| 6330569m22rik | 0.385 |
| d330017j20rik | 0.385 |
| zfp346 | 0.384 |
| ola1 | 0.384 |
| ing3 | 0.384 |
| hipk3 | 0.384 |
| caprin2 | 0.383 |
| zfand6 | 0.383 |
| banp | 0.383 |
| spin1 | 0.383 |
| rpl26 | 0.383 |
| vrk2 | 0.383 |
| kcmf1 | 0.383 |
| dhx32 | 0.383 |
| nptxr | 0.383 |
| rps3a | 0.383 |
| mrfap1 | 0.383 |
| fbxo33 | 0.383 |
| ottmusg00000008540 | 0.383 |
| birc1-rs1 | 0.383 |
| hsp84-2 | 0.383 |
| smpd3 | 0.382 |
| tcstv1 | 0.382 |
| zscan4f | 0.382 |
| tnfrsf22 | 0.382 |
| supv3l1 | 0.382 |
| fkbp3 | 0.382 |
| glcci1 | 0.381 |
| arid3b | 0.381 |
| tpd52l1 | 0.381 |
| ccdc100 | 0.381 |
| ddx41 | 0.381 |
| 6430517e21rik | 0.381 |
| pim3 | 0.381 |
| cxxc5 | 0.381 |
| top3b | 0.38 |
| caml | 0.38 |
| eaf2 | 0.38 |
| mtfr1 | 0.38 |
| srp72 | 0.38 |
| rprm | 0.38 |
| rtn3 | 0.38 |
| stra13 | 0.38 |
| ottmusg00000003456 | 0.38 |
| zfr | 0.379 |
| ppan | 0.379 |
| rbm3 | 0.379 |
| ing2 | 0.379 |
| gpr87 | 0.379 |
| wn | 0.379 |
| trim14 | 0.379 |
| nlrc3 | 0.379 |
| styk1 | 0.379 |
| itpk1 | 0.379 |
| sertad3 | 0.378 |
| ube4b | 0.378 |
| pdcd6 | 0.378 |
| bcas2 | 0.378 |
| spats2 | 0.378 |
| rbbp9 | 0.377 |
| siah2 | 0.377 |
| cks2 | 0.377 |
| zdhhc2 | 0.377 |
| ndrg4 | 0.377 |
| btbd10 | 0.377 |
| map3k13 | 0.377 |
| mirn21 | 0.377 |
| nr2c2ap | 0.377 |
| zfp383 | 0.376 |
| 2610018g03rik | 0.376 |
| b4galt2 | 0.376 |
| tpt1 | 0.376 |
| pak7 | 0.375 |
| trim16 | 0.375 |
| mib1 | 0.375 |
| cabc1 | 0.375 |
| csrnp2 | 0.375 |
| tmem77 | 0.375 |
| zc3h12a | 0.375 |
| rpl38 | 0.375 |
| cugbp2 | 0.375 |
| rp23-143a14.5 | 0.374 |
| txndc1 | 0.374 |
| vdac2 | 0.374 |
| ppil5 | 0.374 |
| thyn1 | 0.374 |
| mycbpap | 0.374 |
| rps5 | 0.374 |
| tulp3 | 0.373 |
| rps19bp1 | 0.373 |
| usp2 | 0.373 |
| tnfrsf26 | 0.373 |
| gdpd5 | 0.373 |
| gm1549 | 0.373 |
| lmtk3 | 0.373 |
| ak2 | 0.373 |
| psmd1 | 0.373 |
| prrxl1 | 0.373 |
| asah3l | 0.372 |
| bat3 | 0.372 |
| cdk5rap3 | 0.372 |
| 2900042b11rik | 0.372 |
| myt1 | 0.372 |
| sbno1 | 0.371 |
| e4f1 | 0.371 |
| letmd1 | 0.37 |
| zfhx2 | 0.37 |
| dnm3os | 0.37 |
| timm50 | 0.37 |
| phlda3 | 0.37 |
| tmem22 | 0.37 |
| vtcn1 | 0.37 |
| dnajc2 | 0.37 |
| 1200014m14rik | 0.37 |
| plekho1 | 0.37 |
| phf16 | 0.37 |
| 4-Sep | 0.37 |
| btbd14b | 0.37 |
| bai1 | 0.37 |
| spata2 | 0.37 |
| 2810022l02rik | 0.369 |
| rbm38 | 0.369 |
| lyrm1 | 0.369 |
| hdgf | 0.369 |
| cep110 | 0.369 |
| axud1 | 0.369 |
| ubash3b | 0.369 |
| slk | 0.369 |
| ddx19a | 0.368 |
| nuak2 | 0.368 |
| bptf | 0.368 |
| huwe1 | 0.368 |
| uxt | 0.368 |
| 1700047i17rik1 | 0.367 |
| toe1 | 0.367 |
| txnl1 | 0.367 |
| crebzf | 0.367 |
| rb1cc1 | 0.367 |
| nlrp5 | 0.367 |
| parp2 | 0.366 |
| aa408296 | 0.366 |
| cdc2l6 | 0.366 |
| ppp1r13b | 0.366 |
| slfn3 | 0.366 |
| ai132487 | 0.366 |
| serpinb9b | 0.366 |
| atg4c | 0.366 |
| gm347 | 0.366 |
| cirbp-rs2 | 0.366 |
| ep400 | 0.366 |
| sgpl1 | 0.366 |
| parp9 | 0.365 |
| eif4g2 | 0.365 |
| bid | 0.365 |
| alkbh1 | 0.365 |
| jtv1 | 0.365 |
| gripap1 | 0.365 |
| ethe1 | 0.365 |
| rpl4 | 0.365 |
| trim38 | 0.365 |
| armet | 0.365 |
| gprc5a | 0.365 |
| tg(trp53r172l)4491jmr | 0.365 |
| loc671535 | 0.364 |
| trp53inp2 | 0.364 |
| igfbp5-ip | 0.364 |
| morf4l2 | 0.364 |
| cgrrf1 | 0.364 |
| shkbp1 | 0.364 |
| asah1 | 0.364 |
| pdcd4 | 0.364 |
| stk40 | 0.364 |
| bex1 | 0.364 |
| tnip2 | 0.364 |
| chchd10 | 0.364 |
| d1mit415 | 0.364 |
| d1mit439 | 0.364 |
| amigo1 | 0.363 |
| amigo3 | 0.363 |
| grwd1 | 0.363 |
| srpx | 0.363 |
| cirbp | 0.363 |
| rapop1 | 0.363 |
| atad5 | 0.363 |
| cdca4 | 0.362 |
| ttll6 | 0.362 |
| luzp1 | 0.362 |
| sgp2 | 0.362 |
| ecel1 | 0.362 |
| emilin2 | 0.362 |
| usp30 | 0.362 |
| actr8 | 0.362 |
| crls1 | 0.362 |
| lztr1 | 0.362 |
| parg | 0.361 |
| itm2b | 0.361 |
| use1 | 0.361 |
| pacs2 | 0.361 |
| ptplb | 0.361 |
| 2610110g12rik | 0.361 |
| pim2 | 0.361 |
| gse1 | 0.361 |
| btg3 | 0.36 |
| casp7 | 0.36 |
| 2310047o13rik | 0.36 |
| zfp830 | 0.36 |
| ptdss2 | 0.36 |
| bin3 | 0.36 |
| mirn20a | 0.36 |
| idh2 | 0.36 |
| dkkl1 | 0.36 |
| mcts1 | 0.359 |
| epb4.1l4b | 0.359 |
| sertad2 | 0.359 |
| xaf1 | 0.359 |
| tnfrsf12a | 0.359 |
| nanos3 | 0.359 |
| eg433968 | 0.359 |
| zfp420 | 0.358 |
| fadd | 0.358 |
| dusp2 | 0.358 |
| bc021395 | 0.358 |
| lrrn1 | 0.358 |
| pcbp4 | 0.358 |
| anp32c | 0.358 |
| sdad1 | 0.358 |
| prelid1 | 0.357 |
| prdm14 | 0.357 |
| kank2 | 0.357 |
| e2f7 | 0.357 |
| trap1a | 0.357 |
| cgref1 | 0.357 |
| tsc22d2 | 0.357 |
| tmem132a | 0.357 |
| agmat | 0.357 |
| bysl | 0.357 |
| spdya | 0.357 |
| tcfap4 | 0.357 |
| prkrir | 0.357 |
| taf9b | 0.357 |
| agbl3 | 0.357 |
| b230208h17rik | 0.357 |
| eif1 | 0.357 |
| d11mit109 | 0.357 |
| d11mit205 | 0.357 |
| tatdn1 | 0.356 |
| wdr1 | 0.356 |
| oip5 | 0.356 |
| dbpht1 | 0.356 |
| map3k6 | 0.356 |
| aim2 | 0.356 |
| phf13 | 0.356 |
| 2610301g19rik | 0.356 |
| 2310003f16rik | 0.356 |
| rexo2 | 0.356 |
| c1d | 0.356 |
| tnni3k | 0.356 |
| atad3a | 0.356 |
| zfp91 | 0.355 |
| fbf1 | 0.355 |
| atf5 | 0.355 |
| trim45 | 0.355 |
| d9mit239 | 0.355 |
| orf9 | 0.355 |
| gas7 | 0.355 |
| unc5cl | 0.355 |
| ripk1 | 0.355 |
| bc029169 | 0.355 |
| sap30bp | 0.355 |
| jmy | 0.354 |
| xrra1 | 0.354 |
| ddx25 | 0.354 |
| ccdc7 | 0.354 |
| 1700126l10rik | 0.354 |
| bc016579 | 0.354 |
| dullard | 0.354 |
| zfp322a | 0.354 |
| map3k5 | 0.354 |
| ube4a | 0.354 |
| fem1a | 0.354 |
| rpl24 | 0.354 |
| pet1 | 0.354 |
| mirn335 | 0.354 |
| rps9 | 0.353 |
| tmem45a | 0.353 |
| lrrc18 | 0.353 |
| tm2d3 | 0.353 |
| narg2 | 0.353 |
| sidt1 | 0.353 |
| tcfl5 | 0.353 |
| tmem16j | 0.353 |
| ubb1 | 0.353 |
| ugcg | 0.353 |
| bcl6b | 0.353 |
| tex2 | 0.353 |
| mtbp | 0.353 |
| rb(6.16)24lub | 0.353 |
| rb(6.16)24lub | 0.353 |
| c80913 | 0.353 |
| slc25a37 | 0.353 |
| btla | 0.353 |
| eg637021 | 0.353 |
| snx14 | 0.353 |
| krt85 | 0.353 |
| stk38 | 0.353 |
| wsb1 | 0.352 |
| paox | 0.352 |
| als2cr2 | 0.352 |
| hint2 | 0.352 |
| cox4i2 | 0.352 |
| fndc3b | 0.352 |
| zfp641 | 0.352 |
| depdc1a | 0.352 |
| 1190002h23rik | 0.352 |
| ebf3 | 0.352 |
| rpl15 | 0.352 |
| plp2 | 0.352 |
| 4732474o15rik | 0.351 |
| kank1 | 0.351 |
| dazap2 | 0.351 |
| nrn1 | 0.351 |
| gzmk | 0.351 |
| 2310047d13rik | 0.351 |
| eg653016 | 0.351 |
| nav2 | 0.351 |
| krt23 | 0.351 |
| 4933424b01rik | 0.351 |
| mirn34b | 0.351 |
| mirn34c | 0.351 |
| ifi9.5 | 0.351 |
| vrk3 | 0.351 |
| d13mit114 | 0.35 |
| d13mit216 | 0.35 |
| rpl23 | 0.35 |
| gnl3 | 0.35 |
| dph1 | 0.35 |
| slfn1 | 0.35 |
| map3k10 | 0.35 |
| serinc5 | 0.35 |
| creg1 | 0.35 |
| mfn2 | 0.35 |
| ccng1 | 0.35 |
| eif2a | 0.35 |
| prkra | 0.35 |
| piwil2 | 0.35 |
| txn2 | 0.349 |
| ergic3 | 0.349 |
| magmas | 0.349 |
| eif3k | 0.349 |
| eif3s10 | 0.349 |
| specc1 | 0.349 |
| olfr805 | 0.349 |
| 9330133o14rik | 0.349 |
| naip1 | 0.349 |
| d6mit373 | 0.349 |
| 1110001a07rik | 0.349 |
| ankhd1 | 0.349 |
| lce3c | 0.348 |
| lass1 | 0.348 |
| pdrg1 | 0.348 |
| 2300002d11rik | 0.348 |
| btg4 | 0.348 |
| lass5 | 0.348 |
| uchl3 | 0.348 |
| atp13a3 | 0.348 |
| pbk | 0.348 |
| sirt7 | 0.348 |
| lims2 | 0.348 |
| npdc1 | 0.348 |
| tiaf2 | 0.348 |
| bbx | 0.348 |
| camk2n2 | 0.348 |
| ehf | 0.347 |
| lsg1 | 0.347 |
| centg1 | 0.347 |
| dyrk2 | 0.347 |
| 9130005n14rik | 0.347 |
| sgms2 | 0.347 |
| setd3 | 0.347 |
| 12-Sep | 0.347 |
| rrp15 | 0.347 |
| morc1 | 0.347 |
| ppm1l | 0.347 |
| sh3glb2 | 0.347 |
| gpr137b | 0.347 |
| gzmn | 0.347 |
| ivns1abp | 0.346 |
| bambi | 0.346 |
| rb(16.17)32lub | 0.346 |
| rb(16.17)32lub | 0.346 |
| dtd1 | 0.346 |
| rqcd1 | 0.346 |
| ccndbp1 | 0.346 |
| dhcr24 | 0.346 |
| zfp326 | 0.346 |
| avil | 0.346 |
| gfer | 0.345 |
| rbm4b | 0.345 |
| usp7 | 0.345 |
| zfat | 0.345 |
| bcl2l12 | 0.345 |
| cabin1 | 0.345 |
| ifitm1 | 0.345 |
| aw146242 | 0.345 |
| rsu1 | 0.345 |
| naip5 | 0.345 |
| gmds | 0.345 |
| btbd7 | 0.345 |
| spz1 | 0.345 |
| nat5 | 0.345 |
| lzts2 | 0.345 |
| mrpl43 | 0.344 |
| pa2g4 | 0.344 |
| dbc1 | 0.344 |
| birc2 | 0.344 |
| mprip | 0.344 |
| nmi | 0.344 |
| tnfrsf14 | 0.344 |
| cables1 | 0.344 |
| syvn1 | 0.344 |
| slfn2 | 0.344 |
| otud7b | 0.344 |
| rapop2 | 0.344 |
| dab2ip | 0.344 |
| mlkl | 0.344 |
| xrn1 | 0.344 |
| alg2 | 0.344 |
| rplp1 | 0.343 |
| akt1s1 | 0.343 |
| tnip3 | 0.343 |
| ctdsp1 | 0.343 |
| capns1 | 0.343 |
| mcrs1 | 0.343 |
| tm2d1 | 0.343 |
| degs1 | 0.343 |
| etnmg1 | 0.343 |
| ankrd32 | 0.343 |
| zc3h12b | 0.343 |
| klf10 | 0.343 |
| emb | 0.343 |
| prdm10 | 0.342 |
| ssbp2 | 0.342 |
| aym1 | 0.342 |
| anp32a | 0.342 |
| gpr132 | 0.342 |
| dgka | 0.342 |
| coq7 | 0.342 |
| neu3 | 0.342 |
| v165-d-j-c mu | 0.342 |
| trim27 | 0.342 |
| unc50 | 0.342 |
| zfp113 | 0.342 |
| psmg2 | 0.342 |
| rnf6 | 0.342 |
| ccdc115 | 0.342 |
| tmem74 | 0.341 |
| ppp1r10 | 0.341 |
| lrig3 | 0.341 |
| akirin1 | 0.341 |
| taf8 | 0.341 |
| d10wsu102e | 0.341 |
| snx31 | 0.341 |
| phca | 0.341 |
| mybl1 | 0.341 |
| pabpc4 | 0.341 |
| tsp1 | 0.341 |
| smpd1 | 0.341 |
